# Enantioselective Total Syntheses of Preussomerins: Control of Spiroacetal Stereogenicity by Photochemical Reaction of a Naphthoquinone through 1,6‐Hydrogen Atom Transfer

**DOI:** 10.1002/anie.202213682

**Published:** 2022-12-21

**Authors:** Yoshio Ando, Daichi Ogawa, Ken Ohmori, Keisuke Suzuki

**Affiliations:** ^1^ Department of Chemistry Tokyo Institute of Technology 2-12-1 O-okayama, Meguro-ku Tokyo 152-8551 Japan

**Keywords:** Photochemical Reactions, Preussomerins, Redox Chemistry, Stereospecificity, Total Synthesis

## Abstract

We report the enantioselective total syntheses of preussomerins EG_1_, EG_2_, and EG_3_. The key transformation is a stereospecific photochemical reaction involving 1,6‐hydrogen atom transfer to achieve *retentive* replacement of a C−H with a C−O bond, enabling otherwise‐difficult control of the spiroacetal stereogenic center.

## Graphical abstract

Photochemical reactions of naphthoquinones triggered by 1,6‐hydrogen atom transfer have been developed. This reaction proceeds in stereospecific manner without loss of the stereochemical information of the substrate, enabling the enantioselective total synthesis of preussomerin EGs. The constructed spiroacetal center is the sole stereogenic center in the product, which had been previously impossible to set by exploiting conventional methods.

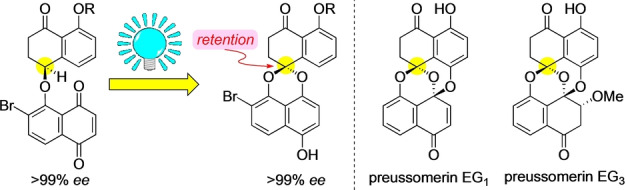

## Introduction

**Scheme 1:**
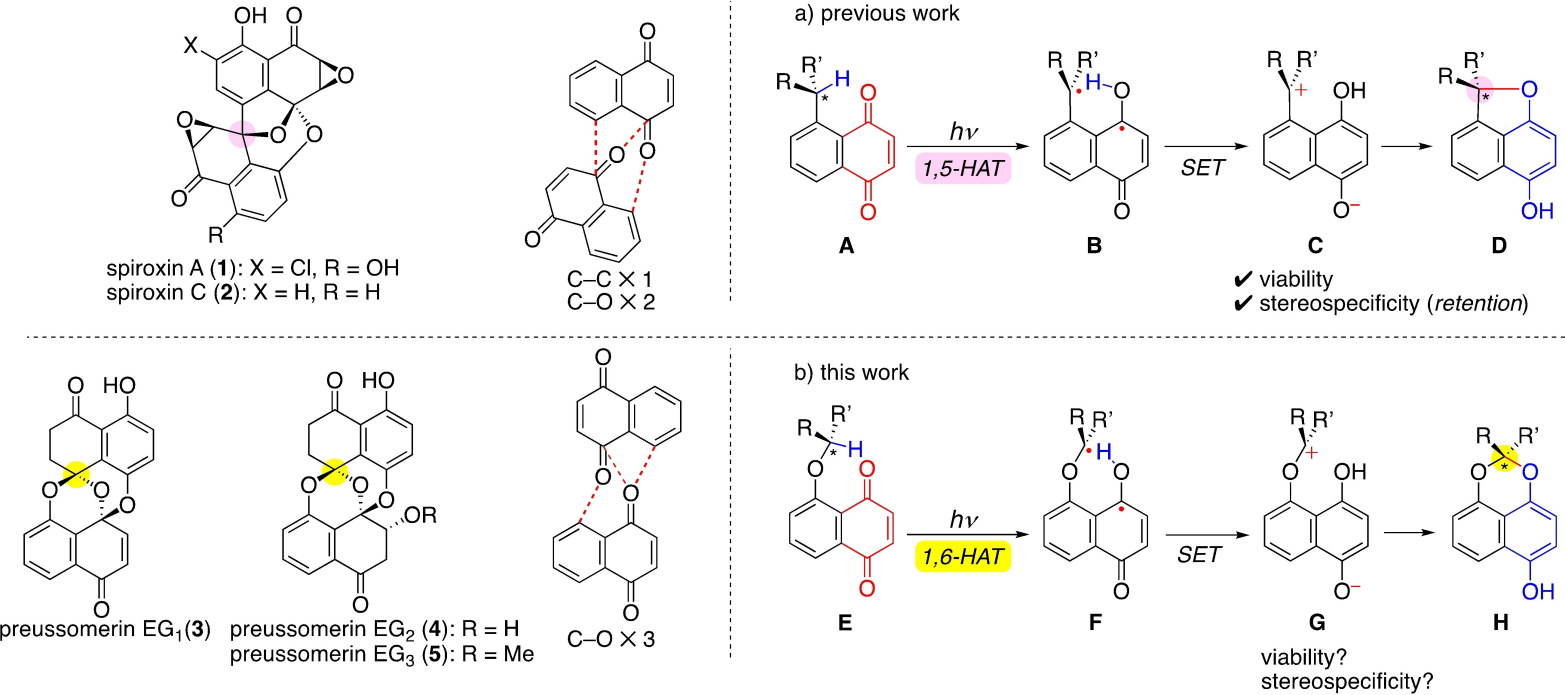
Photochemical approach to spiroxins and preussomerins: Stereospecific 1,5‐HAT (previous work) and 1,6‐HAT (this work).

Spiroxins **1** and **2** ^[1]^ and preussomerins **3**–**5**[^2^, ^3^] are the most elaborate members of the spirodioxynaphthalenes, a class of fungi‐derived, highly oxygenated naphthoquinone dimers with potent bioactivities (Scheme 1).^[4]^ Formidable synthetic challenges are provided by their molecular complexity due to the triply linked bis‐naphthoquinone scaffolds. Note that **1** and **2** are linked by a C−C bond and two C−O bonds, while **3**–**5** are linked by three C−O bonds.

In our study toward the enantioselective syntheses of these compounds, we previously reported the first enantioselective total syntheses of **1** and **2** ^[5]^ by exploiting a photochemical reaction of naphthoquinone **A** ^[6]^ that enabled us to control the spiroether stereogenic center (pink). The benzylic C−H bond in **A** is nicely replaced by an internal C−O bond in **D** with *retention* of the configuration by the following mechanism: Upon n‐π* photoexcitation of **A**, the subsequent processes are 1) 1,5‐hydrogen atom transfer (1,5‐HAT) to give biradical **B**, 2) single electron transfer (SET) to form zwitterion **C**, and 3) cyclization to cyclic ether **D**, which proceed rapidly enough relative to the internal C−C bond rotations to enable a stereospecific transformation. This process can be regarded as an intramolecular redox reaction, in which the benzylic oxidation level is increased and the quinone is reduced.

We turned our attention to preussomerins **3**–**5**, which are even more challenging targets in view of the obvious difficulty in controlling a spiroacetal stereogenic center (yellow).[^7^, ^8^] Faced with this problem, we focused again on the above‐mentioned photochemical reaction, but using naphthoquinone substrate **E** designed by inserting an oxygen atom into **A** en route to chiral, non‐racemic spiroacetal **H**.

Two concerns were foreseen:

1. Viability: Process **E**→**F** involves a 1,6‐hydrogen atom transfer (1,6‐HAT), known to be a less facile process than its 1,5‐counterpart.[^9^, ^10^, ^11^]
2. Stereospecificity: The anchoring oxygen atom in **E** adds conformational mobility (with respect to **A**) that may increase the chances for racemization during the course of the reaction.[^5^, ^12^]

Herein we describe the design of viable naphthoquinone substrates that allow the desired stereospecific photochemical reaction involving a 1,6‐HAT process, and the first enantioselective total syntheses of preussomerins EG_1_ (**3**), EG_2_ (**4**), and EG_3_ (**5**).

## Results and Discussion

The initial feasibility study is summarized in Table 1. We selected *O*‐benzyl juglone **6** as a substrate, which was irradiated with fluorescent light (CH_3_CN, RT, entry 1).^[6a]^ As the resulting product **7** was unstable to air, it was protected as a methyl ether. The fact that acetal **8** was obtained established the feasibility of the projected photochemical reaction, although the yield was low. Interestingly, this is the first report on the photochemical reaction of **6**, which had been long known, and must have previously been exposed to light.^[13]^ In addition, the dimeric product **12** was obtained in 17 % yield, which came from an oxidative dimerization of phenol **7** followed by methylation.^[14]^ Unfortunately, the reaction took a very long time (5.5 days), which stands in contrast to the cases with a 1,5‐HAT (Scheme 1),^[6a]^ suggesting the expected difficulty of the 1,6‐HAT.

**Table 1:** Initial feasibility study.

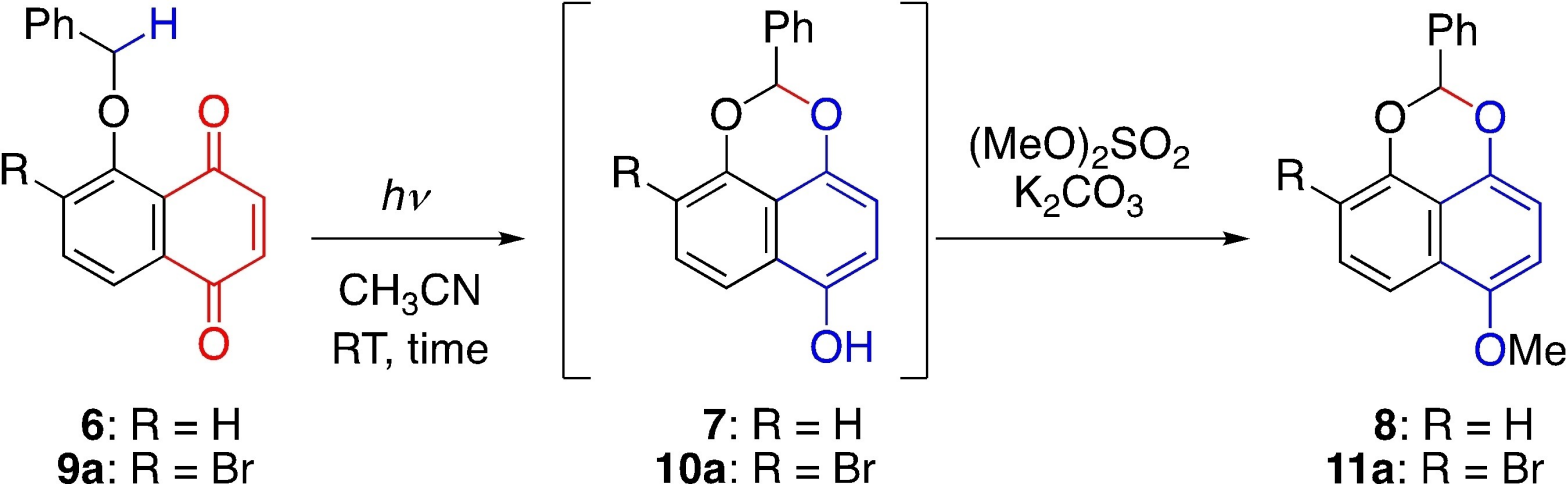				
Entry	Quinone	*hν*	*t*	Yield [%]
1	**6**	fluorescent light	5.5 days	15
2	**9 a**	fluorescent light	2 days	80
3	**9 a**	LED (448 nm)	20 min	84
4	**6**	LED (448 nm)	2 h	42
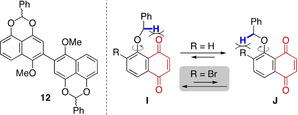				

At this juncture, we considered that the low efficiency of the photochemical reaction could be attributed to the scarcity of the reactive conformer **I** (R=H), in which the benzylic C−H bond is disposed near the quinone carbonyl. Note that **J** is an unreactive conformer: Even if photoactivated, the excited state derived from **J** would not be productive due to the spatially unfavorable arrangement, and would relax back to the ground state.

With the above consideration in mind, we came up with the idea of installing a bromine atom as in **9 a**, inspired by a report by Baldwin and Brown in 1969.^[15]^ Our hope was that the population of the reactive conformer **I** (R=Br) would be increased, providing an entropic advantage for the 1,6‐HAT to occur. Indeed, the irradiation of **9 a** with fluorescent light afforded acetal **11 a** in high yield (80 %), and the reaction time was shortened (2 days, entry 2).^[16]^ Moreover, the reaction time was further shortened (20 min) by using LED irradiation (448 nm, 680 mW), affording **11 a** in 84 % yield (entry 3). The LED light also shortened the reaction time of non‐bromo substrate **6** (2 h), although the yield was lower (42 %, entry 4).

Having obtained this promising result, we examined the substrate scope of the reaction by using bromonaphthoquinones **9 b**–**l** with various ether substituents (Table 2).^[14]^

**Table 2:** Photochemical reaction of bromonaphthoquinones **9 b**–**l**.

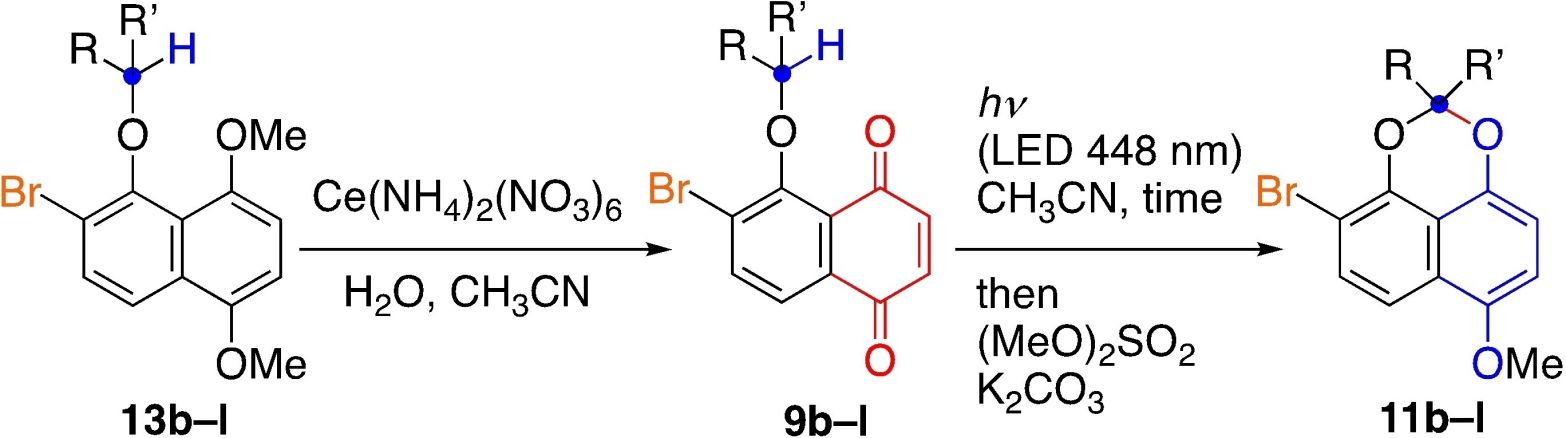					
Entry	Label	R	R′	*t* ^[a]^	Yield [%]^[b]^
1	**b**	CH=CH_2_	H	20 min	71
2	**c**	H	H	3.5 h	25
3	**d**	Me	H	1 h	56
4	**e**	Me	Me	15 min	78
5	**f**	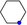		15 min	84
6	**g**	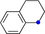		15 min	69
7	**h**	OMe	H	1 h	30
8	**i**	COOEt	H	1.5 h	27
9	**j**	CN	H	30 min	decomposed
10	**k**	CH_2_Ph	H	30 min	67
11	**l**	cyclopropyl	H	30 min	69
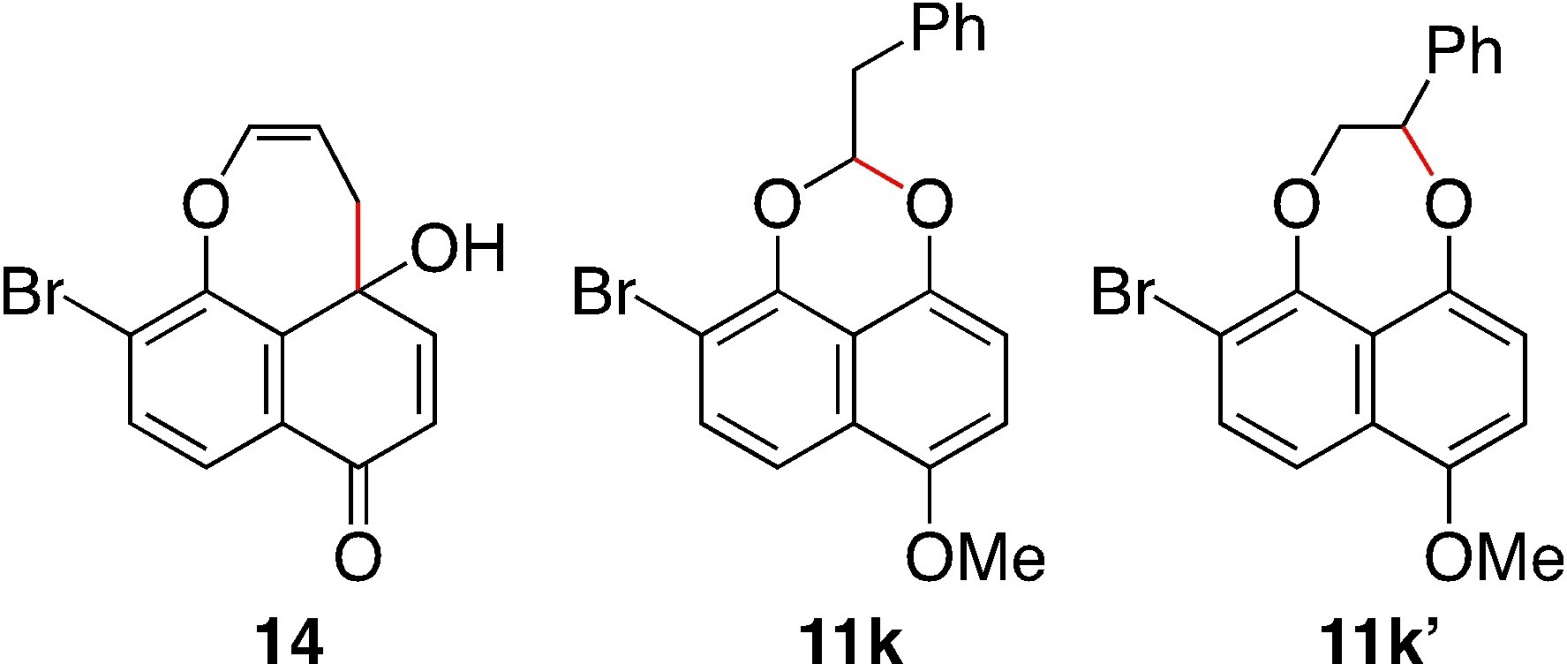					

[a] Time required for the complete consumption of **9**. [b] Two‐step yield from naphthalene **13**.

Two experimental precautions were implemented: 1) Considering the potential instability of bromonaphthoquinones **9 b**–**l** toward room light, they were prepared from the corresponding naphthalenes **13 b**–**l** immediately before the photoreactions, and 2) as the resulting products were unstable to air, they were isolated as the corresponding methyl ethers **11 b**–**l** by treatment with dimethyl sulfate (for the general procedure, see the Supporting Information).

Quinone **9 b** with an allylic C−H bond (R=CH=CH_2_, R′=H) was also a good substrate, giving the corresponding acetal **11 b** in 71 % yield, although Norrish–Yang product **14** was obtained as a minor product (entry 1). On the other hand, quinone **9 c** with a methyl group (R=R′=H) was a poor substrate, requiring a longer reaction time and giving only poor yield (entry 2). Better results were obtained with larger alkyl substituents. Quinone **9 d** with an ethyl group (R=Me, R′=H) underwent the reaction in a shorter time, giving an improved yield (entry 3). The improvement was even more prominent with quinone **9 e** with an isopropyl group (R=R′=Me, entry 4), showing that the dialkyl substitution leads to good substrates. Indeed, quinones **9 f** and **9 g** with cycloalkyl groups gave the corresponding products **11 f** and **11 g** with spiroacetal structures (entries 5 and 6), which were promising results for the synthesis of the preussomerins.

Given the reaction mechanism (Scheme 1), this trend could be rationalized by considering the substitution effect in terms of:

1. the bond dissociation energies of the reacting C−H bond
2. the electron‐donating ability to facilitate the SET step^[6]^

Along these lines, we expected quinone **9 h** (R=OMe, R′=H) would be a good substrate, as the methoxy group weakens the C−H bond and exerts an electron‐donating ability (entry 7). However, orthoester **11 h** was obtained in only 30 % yield, owing to the chemical lability of the methoxymethyl group, which was partially cleaved during the reaction.

Our related expectation for the electron‐withdrawing group R was that it would discourage the SET process. Indeed, quinone **9 i** (R=COOEt, R′=H) gave acetal **11 i** in low yield (entry 8), and **9 j** (R=CN, R′=H) gave an intractable mixture of unidentified products (entry 9).^[6a]^

The reaction of quinone **9 k** with a 2‐phenylethyl group (R=CH_2_Ph, R′=H) gave **11 k** in 67 % yield as the sole product arising from the expected 1,6‐HAT (entry 10). Our interest in this particular substrate **9 k** was that a 1,7‐hydrogen atom transfer (1,7‐HAT) may compete by participation of the distal C−H bond (a weak benzylic bond). As it turned out, none of **11 k′** expected from the 1,7‐HAT was detected (see the bottom of Table 2).

Quinone **9 l** with a cyclopropylmethyl group (R=cyclopropyl, R′=H) gave acetal **11 l** in 69 % yield (entry 11), suggesting that the SET process was much faster than the ring opening of the cyclopropylmethyl radical and/or the corresponding cationic species,[^17^, ^18^] as evidenced by NMR analysis of the crude products: the lack of vinyl protons suggested the absence of the ring‐opened products.

**Scheme 2:**
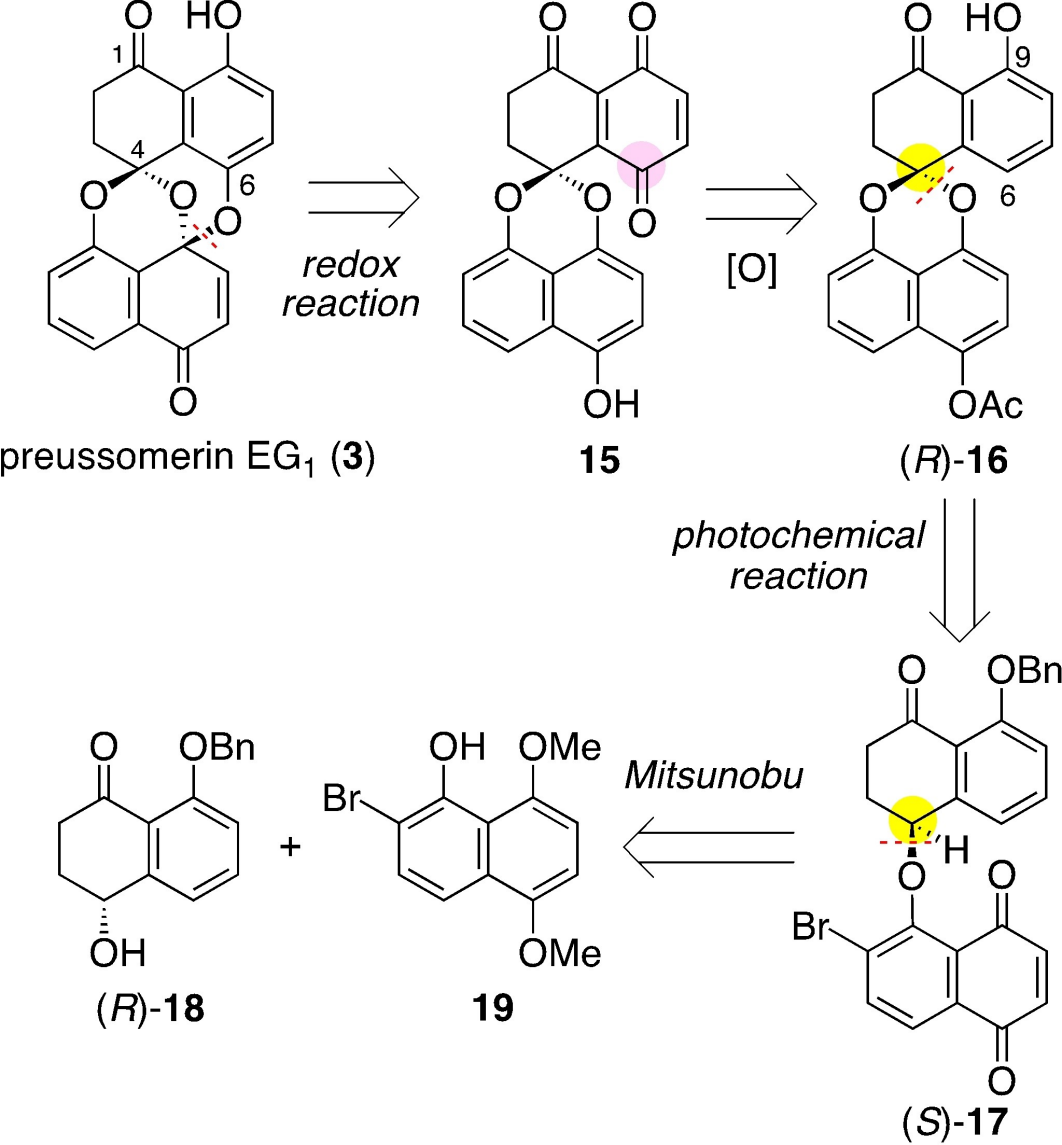
Retrosynthetic analysis.

Having studied the substrate scope, we proceeded to plan the synthesis of preussomerin EG_1_ (**3**), paying particular attention to the stereospecificity. Scheme 2 shows our retrosynthetic analysis. The bis(spiroacetal) structure in **3** would be derived from quinone **15** through an intramolecular quinone/hydroquinone redox reaction.[^5b^, ^7a^] Benzoquinone **15** could be derived from phenol (*R*)‐**16**, presuming the installation of an oxygen atom *para* to the C9 phenol. The key spiroacetal stereogenic center in (*R*)‐**16** would hopefully be constructed from chiral, non‐racemic naphthoquinone (*S*)‐**17** by a stereospecific photochemical reaction. This is the key step, and we envisioned it would proceed in a stereoretentive manner.[^5^, ^12^] Naphthoquinone (*S*)‐**17** could be derived from chiral, non‐racemic tetralone (*R*)‐**18** and the known bromonaphthol **19** ^[19]^ by Mitsunobu reaction. The starting material (*R*)‐**18** is the synthetic intermediate of our previous synthesis of the spiroxins.^[5]^

**Scheme 3:**
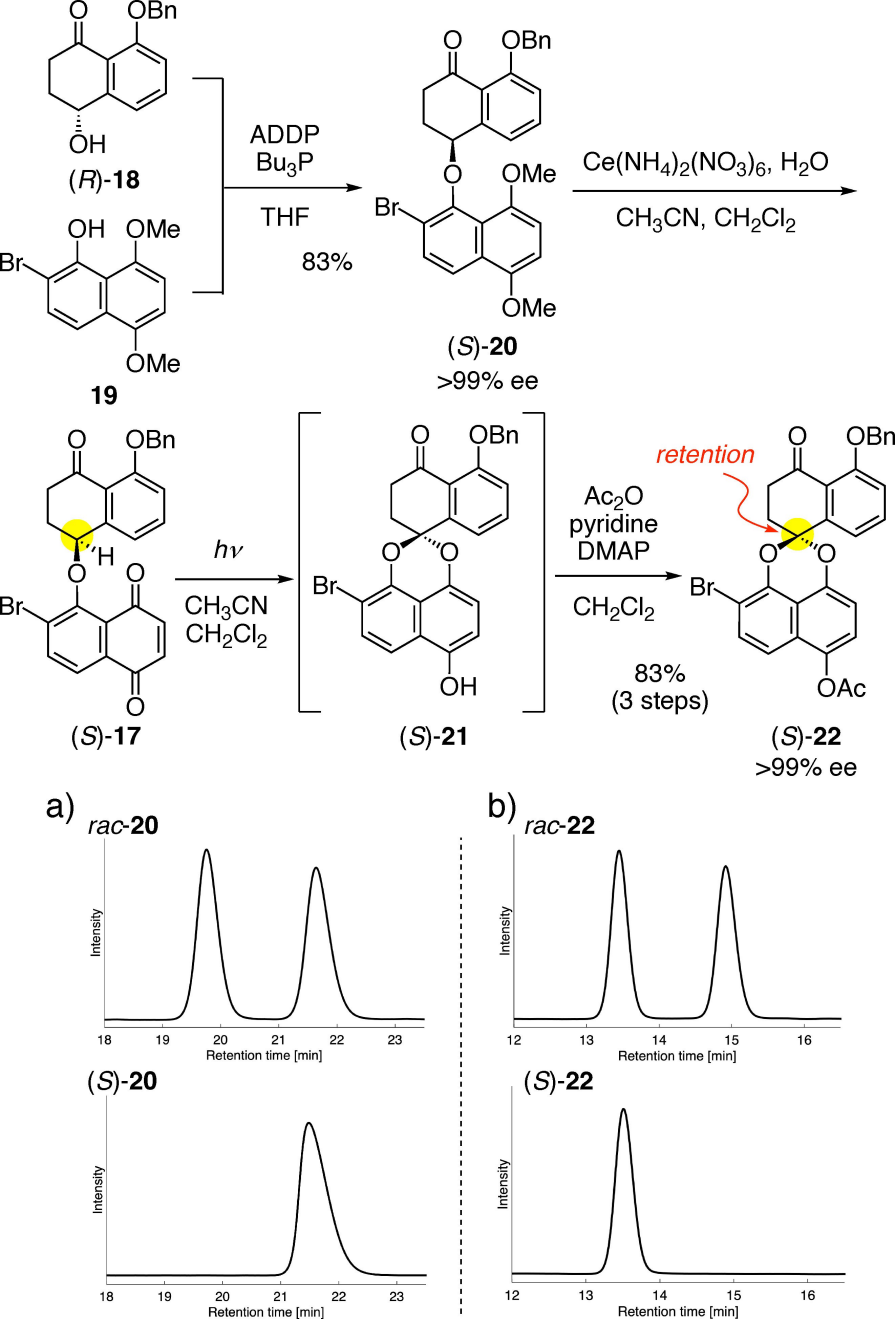
Substrate synthesis and stereospecific photochemical reaction. DMAP=4‐(dimethylamino)pyridine.

Scheme 3 shows the preparation of naphthoquinone **17** as the key stereodefined substrate of the photochemical reaction. Alcohol (*R*)‐**18** ^[20]^ and naphthol **19** were combined by a Mitsunobu reaction [1,1′‐(azodicarbonyl)dipiperidine (ADDP), Bu_3_P, THF, RT]^[21]^ to give tetralone (*S*)‐**20**. The product was recrystallized from CH_3_CN, giving enantiomerically pure material (*S*)‐**20** (>99 % *ee*, Scheme 3a). Following the developed procedure,^[16]^ (*S*)‐**20** was oxidized to naphthoquinone (*S*)‐**17**, which was irradiated with an LED light (448 nm) in CH_3_CN and CH_2_Cl_2_,^[22]^ giving the corresponding spiroacetal **21**. After removal of the volatile components of the mixture, one‐pot acetylation gave spiroacetal **22** in 83 % yield over the three steps from (*S*)‐**20**.

**Figure 1:**
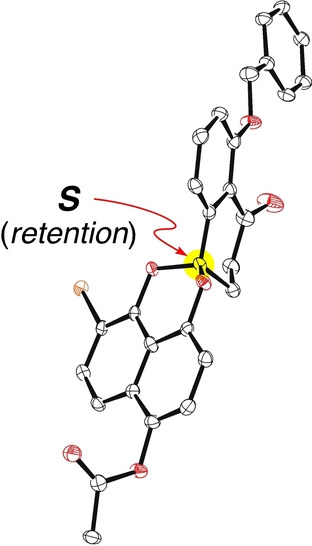
X‐ray crystal structure of spiroacetal (*S*)‐**22**.

We were pleased to realize that the reaction proceeded in a stereospecific manner (>99 % *ee*, Scheme 3b), as verified by HPLC analysis of **22** on a chiral stationary phase (CHIRALPAK® IB, eluent: hexane/EtOAc=80/20, flow rate: 1 mL min^−1^, 25 °C, retention time: 13.5 min for the *S* isomer, 14.9 min for the *R* isomer). The absolute configuration of the spiroacetal center in **22** was assigned as *S* by X‐ray diffraction analysis (Figure 1),^[23]^ verifying the stereoretentive nature of the key photochemical reaction.

We are pleased to document that even a photochemical process including 1,6‐HAT can nicely transmit stereochemical information to the product without any loss of the enantiomeric excess.^[24]^

**Scheme 4:**
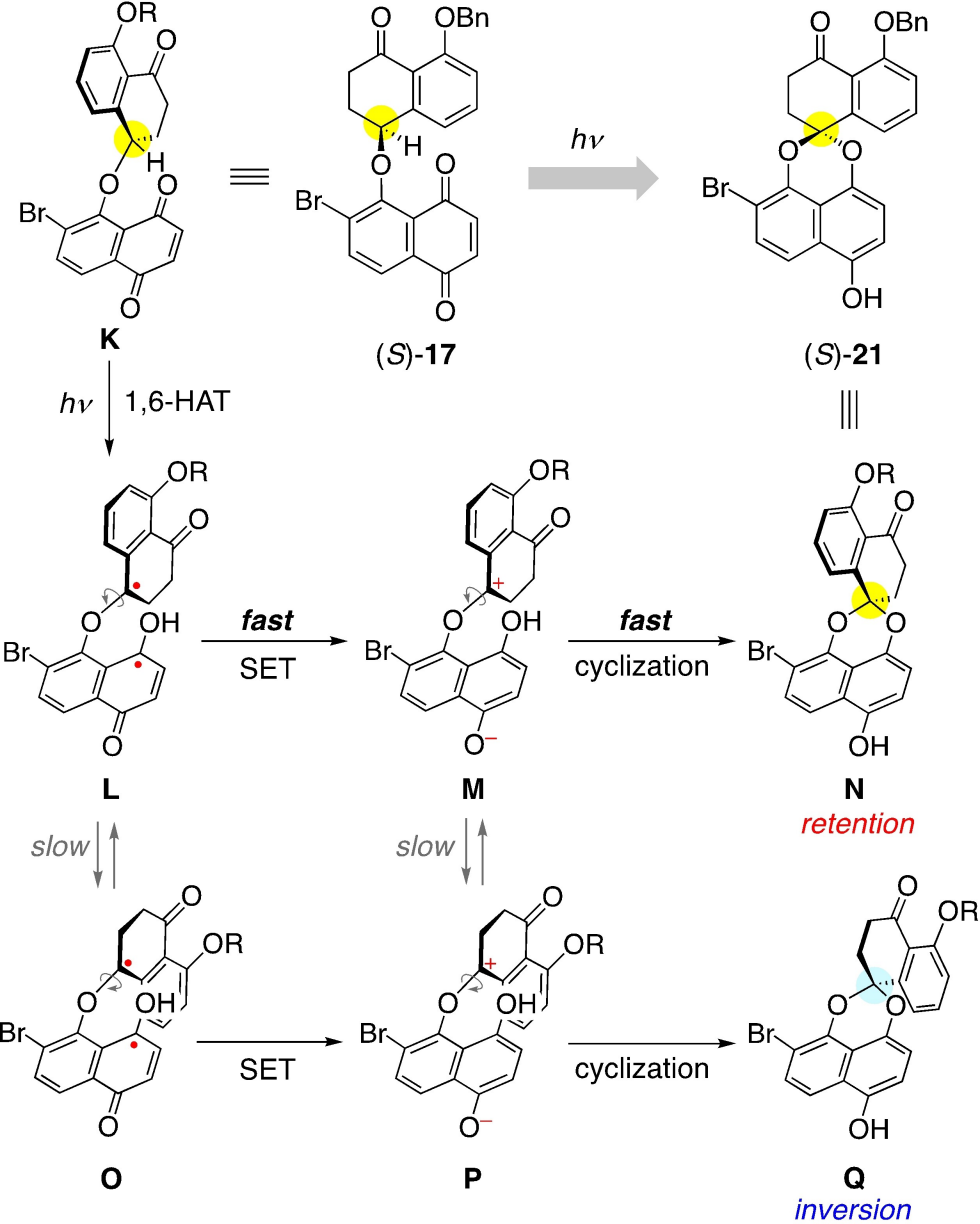
Rationale for the stereoretentive reaction.

Scheme 4 shows the rationale for the stereospecific conversion of (*S*)‐**17** into (*S*)‐**21** based on our working hypothesis.^[5]^ Among possible conformations of (*S*)‐**17**, the key conformer **K** has the hydrogen atom near the quinone carbonyl group. Upon photoexcitation, a 1,6‐HAT process occurs to generate biradical **L**, whose molecular shape reflects that of **K**, in which the stereochemical information of (*S*)‐**17** is retained as axial chirality.

Given the following processes **L**→**M**→**N** were fast enough relative to the conformational flipping of two intermediates **L** (to give **O**) and **M** (to give **P**), the final cyclization regenerates the central chirality in the product **N** [=(*S*)‐**21**] with retention of configuration, which is consistent with the present result.^[25]^

In some cases in our previous study partial stereomutation was observed, suggesting the intervention of such conformational changes of reactive intermediates, while the present case was not disturbed by such stereochemical deterioration.^[26]^

**Scheme 5:**
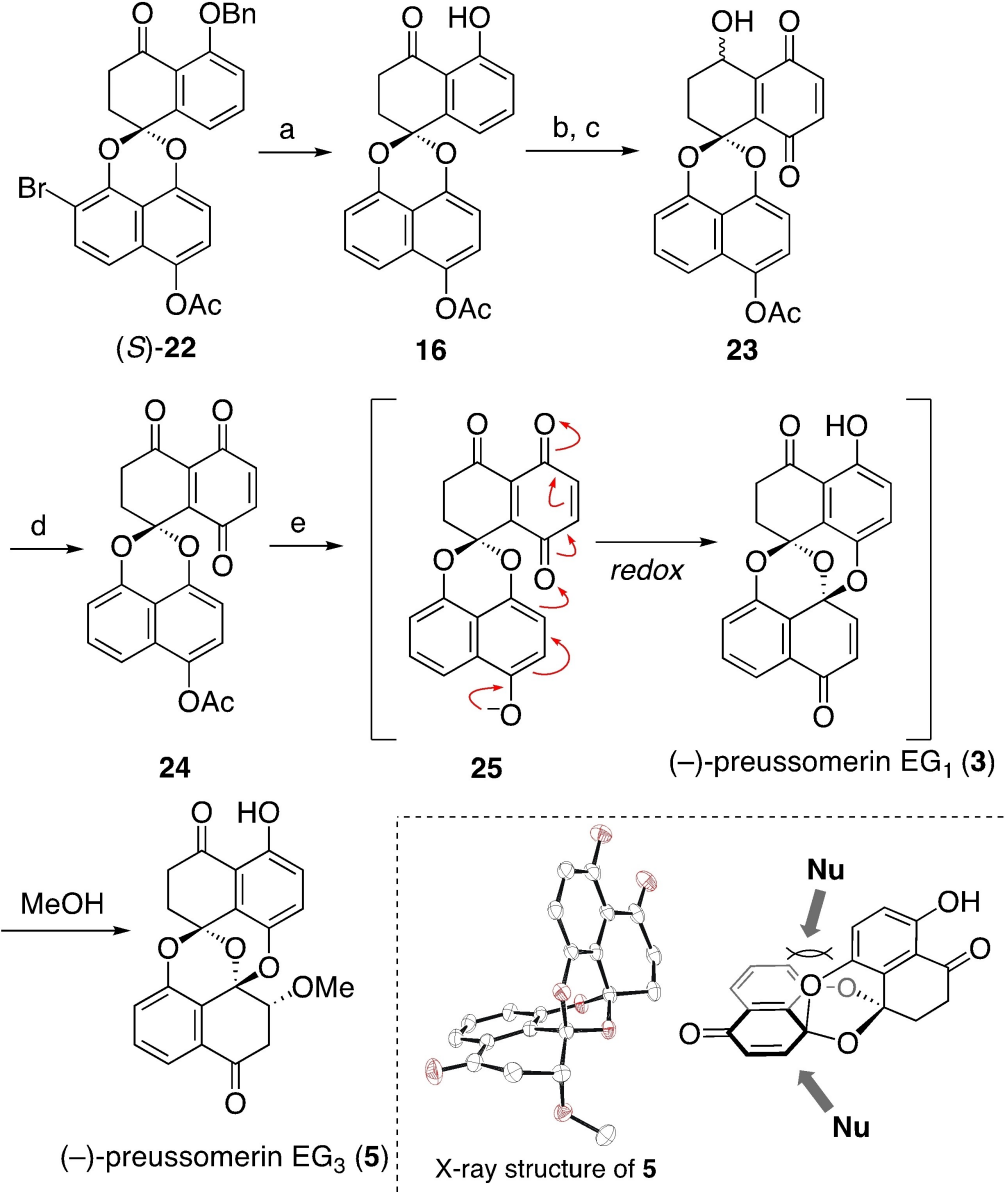
Total synthesis of (−)‐preussomerin EG_3_. a) H_2_, 10 % Pd/C, Et_3_N, THF, MeOH, RT, 94 %; b) NaBH_4_, THF, MeOH, RT, 90 %; c) PhI(OCOCF_3_)_2_, THF, H_2_O, RT, 41 %; d) Dess–Martin periodinane, CH_2_Cl_2_, RT; e) K_2_CO_3_, MeOH, 0 °C, 85 % (2 steps).

With enantiomerically pure (*S*)‐**22** in hand, the next task was its conversion into quinone **24**, the key intermediate toward the preussomerins (Scheme 5). Hydrogenolysis of spiroacetal (*S*)‐**22** removed the benzyl group and the bromine atom, giving phenol **16**. Addition of triethylamine was crucial for the debromination.^[27]^

Attempted oxidation of phenol **16** to quinone **24** did not proceed at all. We attributed this failure to the poor π‐electron density of phenol **16** by the presence of a carbonyl group. After considerable experimentation, we decided to transform the ketone into the corresponding alcohol. Ketone **16** was reduced with NaBH_4_, and the resulting alcohol was treated with PhI(OCOCF_3_)_2_ in wet THF to produce the desired quinone **23** in 41 % yield.^[7b]^ Although many other possibilities were examined, this was the optimum yield. A dilemma was that the application of stronger oxidants seemed to cause competitive oxidation of the naphthalene moiety.

Oxidation of alcohol **23** with Dess–Martin periodinane gave labile ketone **24**, which was used without silica gel chromatography for the next step in the synthesis of preussomerin EG_1_ (**3**). Acetate **24** was treated with K_2_CO_3_ in methanol (Scheme 5). Although the projected reaction (**24**→**3**) indeed proceeded, it was accompanied by the facile 1,4‐addition of methanol to give preussomerin EG_3_ (**5**) in 85 % yield. All the spectroscopic data of the synthetic material **5** matched with the reported data for the natural product.^[2]^ The structure was further verified by X‐ray diffraction analysis.^[23]^

The facile conversion of **24** into **5** could be explained as follows: Removal of the acetyl group in **24** gives phenolate **25**,^[28]^ which undergoes an internal redox reaction to give the bis(spiroacetal) structure of **3**.[^5b^, ^7a^] This process could be expressed as a formal intramolecular 1,6‐addition (see the electron arrows in **25**, Scheme 5). The α,β‐unsaturated system in **3** underwent facile attack of a methanol to afford **5** as a single stereoisomer.^[7a]^ To explain this rigorous stereoselectivity, we could conceive three relevant factors, albeit of unknown relative significance.

1. convex/concave (outside from the cage)
2. Felkin‐Anh (anti to the α‐oxygen)
3. axial attack (minimizing the torsional strain)

Seeking direct access to **3**, we screened several reaction parameters to suppress the extra 1,4‐addition to produce **5**, which unfortunately turned out to be unfruitful (Table 3). Entry 1 shows the result already presented in Scheme 5. Lowering the temperature (−20 and −40 °C) afforded **3** in low yield (entries 2 and 3). Changing the solvent (EtOH and *t*‐BuOH) resulted in a complex mixture and gave only a small amount of **3** without the corresponding adduct **27** and **28**, respectively (entries 4 and 5). When hydroxide was employed as a nucleophile, compound **3** was not obtained at all (entry 6). The major product was angular alcohol **29** formed in 43 % yield, which was generated by a 1,4‐addition of hydroxide to the highly electrophilic β‐position of acylquinone **24**.^[29]^

**Table 3:** Attempts at the direct synthesis of **3** from **24**.

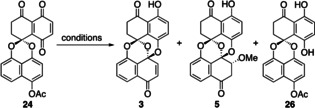							
Entry	Base^[a]^	Solvent^[b]^	*T* [°C]	*t* [h]	Yield [%]^[c]^		
					**3**	**5**	**26**
1	K_2_CO_3_	MeOH	0	1	–	85	–
2	K_2_CO_3_^[d]^	MeOH	−20	1	23	8	14
3	K_2_CO_3_^[d]^	MeOH	−40	6	41	1	6
4	K_2_CO_3_	EtOH	RT	3	2	–	20
5	K_2_CO_3_	*t*‐BuOH^[e]^ CH_2_Cl_2_	RT	4	–	–	12
6	LiOH⋅H_2_O	THF^[f]^ H_2_O	0	0.5	–	–	3
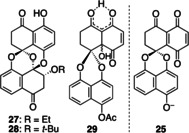							

[a] 2.0 equiv [b] 0.05 M. [c] two‐step yield from **23**. [d] 1.5 equiv [e] 0.025 M. [f] 0.02 M.

The essential issue was that the acetyl protecting group on the phenol was much more resistant to removal than expected. Namely, these conditions generated hydroquinone **26** bearing an acetyl group, which was derived from the reduction of **24**. The reductant would be phenolate **25**, functioning as an intermolecular two‐electron donor to the reactive electron acceptor **24**.^[30]^

**Scheme 6:**
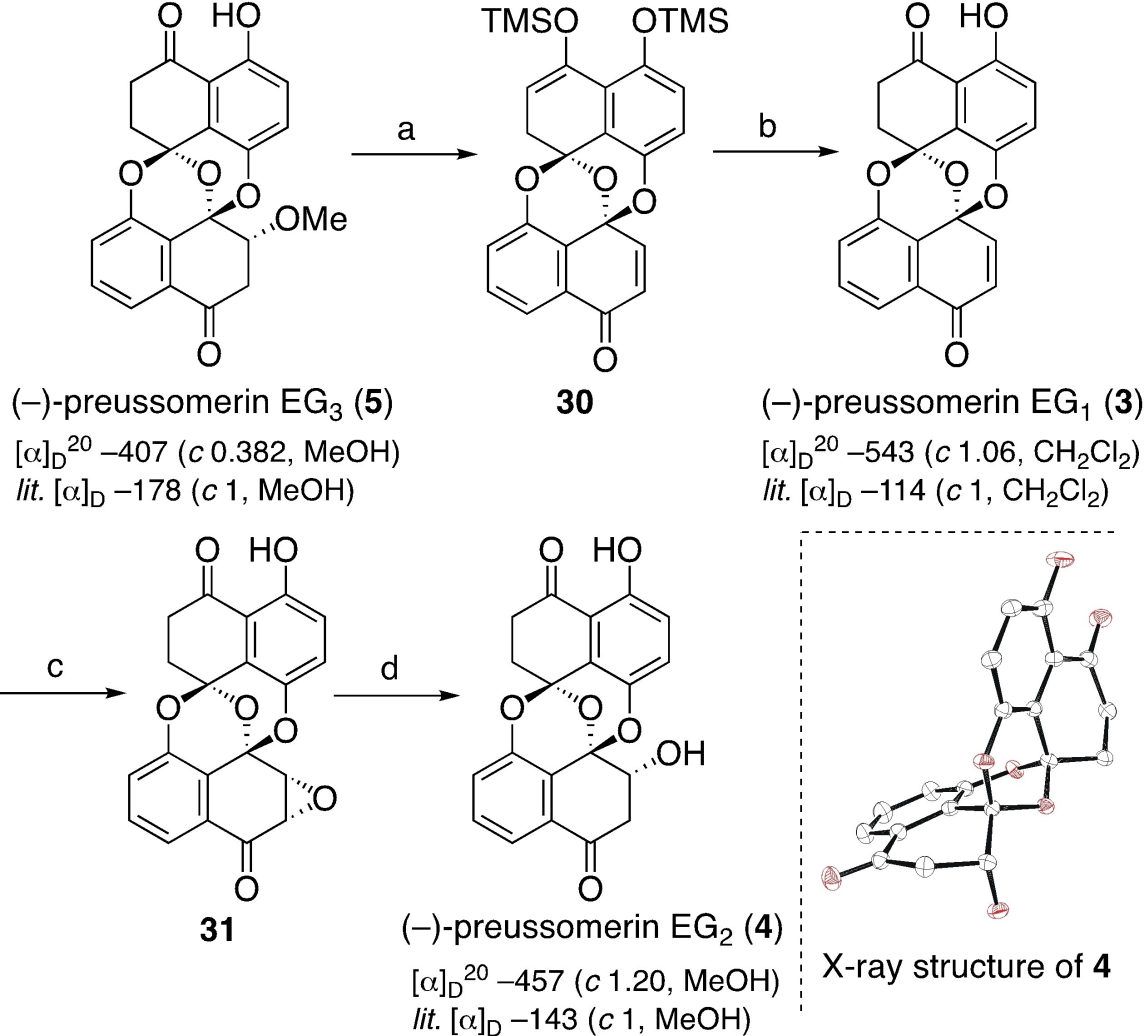
Total synthesis of (−)‐preussomerin EG_1_ and EG_2_. a) TMSOTf, Et_3_N, CH_2_Cl_2_; b) PPTS, THF, H_2_O, RT, 83 % (2 steps); c) *t*‐BuOOH, TBD, CH_2_Cl_2_, 0 °C; d) Zn, AcOH, THF, RT, 88 % (2 steps). TMSOTf=trimethylsilyl trifluoromethanesulfonate, PPTS=pyridinium *p*‐toluenesulfonate, TBD=1,5,7‐triazabicyclo[4.4.0]dec‐5‐ene.

With these results, we judged that the direct synthesis of **3** from **24** was not practical, and alternatively focused on the transformation of **5** into **3** (Scheme 6). Treatment of **5** with TMSOTf and Et_3_N caused the β‐elimination of methanol,^[7a]^ while the ketone and phenol in **3** were simultaneously transformed into the corresponding silyl ether to furnish **30**.^[31]^ Hydrolysis under weakly acidic conditions gave **3** in 83 % yield (2 steps).^[32]^ All the spectroscopic data of the synthetic material **3** were identical with those reported.^[2]^

For the synthesis of (−)‐preussomerin EG_2_ (**4**), the direct 1,4‐addition of H_2_O to **3** was difficult.^[33]^ On the other hand, a two‐step protocol—stereoselective epoxidation and reductive opening of the oxirane ring—nicely enabled the total synthesis of **4** in 88 % yield (2 steps).^[7d]^ All spectroscopic data of the synthetic material **4** were identical to those reported.^[2]^ The structure of **4** was further confirmed by X‐ray diffraction analysis.^[23]^

The [α]_D_ values of the synthetic materials **3**–**5** were the same in sign to those of the natural samples, while substantially different in magnitude. We are confident in the chemical purity and the [α]_D_ values of our synthetic materials, so the discrepancy may come from the low enantiomeric excess and/or insufficient chemical purity of the natural samples.^[34]^

## Conclusion

In conclusion, photochemical reactions of naphthoquinones involving 1,6‐HAT have been developed. The installation of a bromine atom *ortho* to the substituent on the naphthoquinones rendered the 1,6‐HAT facile by an entropic effect. The substrate scope and limitations have been described with respect to the substitution pattern. The photochemical reaction proceeded in a stereospecific manner (retention), which enabled the first enantioselective total syntheses of preussomerins EG_1_, EG_2_, and EG_3_.

## Conflict of interest

The authors declare no conflict of interest.

1

## Supporting information

As a service to our authors and readers, this journal provides supporting information supplied by the authors. Such materials are peer reviewed and may be re‐organized for online delivery, but are not copy‐edited or typeset. Technical support issues arising from supporting information (other than missing files) should be addressed to the authors.

Supporting Information

## Data Availability

The data that support the findings of this study are available in the Supporting Information of this article.
