# Supplementary material for: Enantioselective Total Syntheses of Preussomerins: Control of Spiroacetal Stereogenicity by Photochemical Reaction of a Naphthoquinone through 1,6‐Hydrogen Atom Transfer
Source: Angew Chem Int Ed Engl. 2022 Dec 21;62(5):e202213682. doi: 10.1002/anie.202213682 (PMC10107447; doi:10.1002/anie.202213682)
Supplement: Supplementary file 1 — Supporting Information [file ANIE-62-0-s001.pdf]

Angewandte Chemie
Eine Zeitschrift der Gesellschaft Deutscher Chemiker

Supporting Information

Enantioselective Total Syntheses of Preussomerins: Control of Spiroacetal Stereogenicity by Photochemical Reaction of a Naphthoquinone through 1,6-Hydrogen Atom Transfer

Y. Ando, D. Ogawa, K. Ohmori, K. Suzuki**

Table of contents

General experimental procedures S-2

Experimental procedures and characterization data S-3

NMR spectra S-56

General experimental procedure

All reactions were performed under an argon atmosphere unless otherwise stated. Ethereal solvents and CH_2Cl_2 (anhydrous; *Kanto Chemical Co., Inc.*) were purified under argon by using an Organic Solvent Pure Unit (*Wako Pure Chemical Industries, Ltd.*). DMF, CH_3CN, Et_3N, and TMSOTf were distilled prior to use according to the standard protocols. For thin-layer chromatography (TLC) analysis, Merck pre-coated plates (TLC silica gel 60 F_{254}, Art 5715, 0.25 mm) were used. Silica gel preparative thin-layer chromatography (PTLC) was performed using plates prepared from Merck silica gel 60 PF_{254} (Art 7747). For flash column chromatography, silica gel 60N (Spherical, neutral, 63–210 μm) from Kanto Chemical was used. Melting point (mp) determinations were performed using a METTLER TOLEDO MP 70 Melting Point System or a Yanaco MP-500 instrument or a METTLER TOLEDO MP 70 melting point system, and are uncorrected. ^{1}H- and ^{13}C-NMR were measured on a Bruker Avance III 600 (600 MHz) spectrometer in the solvent indicated; Chemical shifts (δ) are expressed in parts per million (ppm) downfield from internal standard (tetramethylsilane, 0.00 ppm, 7.26 ppm for $CDCl_3$ and 2.04 ppm for acetone-d_6), and coupling constants (J) are reported as hertz (Hz). Splitting patterns are indicated as follows: s = singlet, d = doublet, t = triplet, q = quartet, sept = septet, m = multiplet, br = broad. Infrared (IR) spectra were recorded on a Thermo Fisher SCIENTIFIC NICOLET iS5 FTIR spectrometer. Attenuated total reflectance Fourier-transform infrared (ATR-FTIR) spectra were recorded by using a Thermo Fisher SCIENTIFIC NICOLET iS5 FTIR spectrometer. High-resolution mass spectra (HRMS) were obtained with a Bruker micrOTOF–QII (ESI or APCI). Medium pressure liquid chromatography (MPLC) purifications were performed by using a Yamazen YFLC W–Prep 2XY (prep UV-254W for UV detector, Smart Flash ELSC-100X for ELSD detector). High-flash L column (silica gel 40 μm, 34 g, 26 × 100 mm), High-flash 2L column (silica gel, 40 μm, 45 g, 26 × 150 mm), High-flash 3L column (silica gel, 40 μm, 120 g, 46 × 130 mm) were used. High performance liquid chromatography (HPLC) analyses were performed by a LC-NetII/ADC for controller, a PU-2086 Plus for HPLC pump, a UV-2075 Plus for UV/Vis detector, and a CD-2095 Plus chiral detector. Photoreaction was performed using Techno Sigma PER-AMP (PER-448). UV–VIS spectra were recorded by using a JASCO V-650 spectrometer. X-ray crystallographic data were recorded with a Rigaku VM SPIDER Rigaku, or a Rigaku R-AXIS RAPID IP area detector system.

O-benzyljuglone **6**

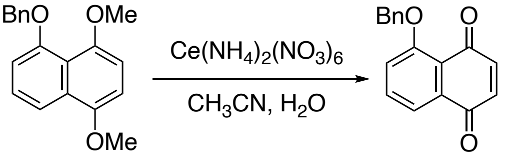

To a solution of **S1**[1] (303 mg, 1.03 mmol) in CH_3CN (30 mL) in a brown round bottom flask was added a solution of ammonium cerium(IV) nitrate (1.20 g, 2.19 mmol) in water (5 mL) dropwise at 0 ºC. After stirring for 5 min at the same temperature, the reaction was stopped by adding saturated aqueous $NaHCO_3$ at 0 ºC. The mixture was poured into water and extracted with EtOAc (×3). The combined organic extracts were washed with brine, dried (Na_2SO_4), and concentrated in vacuo. The residue was purified by MPLC (High-flash L column, hexane/EtOAc = 20/1 → 2.3/1) to afford *O*-benzyljuglone **6** (260 mg, 96%) as a yellow solid.[2]

6: R_f 0.49 (hexane/ Et_2O = 2/1); mp 112–114 °C; ^{1}H NMR (600 MHz, $CDCl_3$) δ 5.31 (s, 2H), 6.89 (s, 2H), 7.33 (t, 1H, *J* = 7.4 Hz), 7.35 (dd, 1H, *J* = 8.4, 1.0 Hz), 7.39–7.44 (m, 2H), 7.58 (br-d, 2H, *J* = 7.2 Hz), 7.65 (dd, 1H, *J* = 8.4, 7.6 Hz), 7.75 (dd, 1H, *J* = 7.6, 1.0 Hz); ^{13}C NMR (150 MHz, $CDCl_3$) δ 70.9, 119.5, 119.7, 120.3, 126.7, 128.0, 128.7, 134.1, 134.8, 136.0, 136.2, 140.9, 158.5, 184.1, 185.2; IR (ATR) 3067, 2906, 1654, 1613, 1580, 1500, 1470, 1442, 1335, 1305, 1283, 1253, 1167, 1120, 1051, 1024, 913, 851, 829, 772, 732, 698, 678 cm^{-1}; UV–Vis (CH_3CN) λ_{max} nm (ε) = 390 (3885), 245 (21279), 205 (32587); HRMS (ESI-TOF) calcd for $C_{17}H_{12}NaO_3$ ($[M+Na]^+$) *m/z* 287.0679, found *m/z* 287.0680.

bromonaphthol **19**[3]

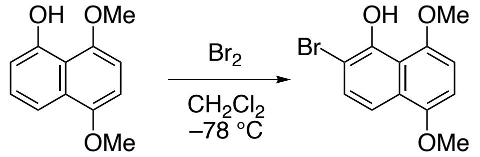

To a solution of naphthol **S2** (9.03 g, 44.2 mmol) in CH_2Cl_2 (400 mL) was added Br_2 (2.40 mL,

[1] Y. Ando, D. Tanaka, R. Sasaki, K. Ohmori, K. Suzuki, *Angew. Chem. Int. Ed.* **2019**, *58*, 12507–12513.

[2] H. Laatsch, *Liebigs Ann. Chem.* **1980**, 1321–1347.

[3] a) R. L. Vézouët, A. J. P. White, J. N. Burrows, A. G. M. Barrett, *Tetrahedron* **2006**, *62*, 12252–12263; b) H. Laatsch, *Liebigs Ann. Chem.* **1985**, 2420–2442.

47.1 mmol) in CH_2Cl_2 (30 mL) at –78 °C. After stirring for 30 min at –78 °C, the reaction was poured into stirred mixed solvent of saturated aqueous $NaHCO_3$ and 10% aqueous $Na_2S_2O_3$ at 0 °C. The mixture was extracted with CH_2Cl_2 (×3), and the combined organic extracts were washed with saturated aqueous $NaHCO_3$, 10% aqueous $Na_2S_2O_3$ and brine, dried (Na_2SO_4), and concentrated in vacuo. Reprecipitation with hexane and CH_2Cl_2 afforded **19** (1st crop: 6.40 g, 51%; 2nd crop: 2.52 g, 20%) as a pale green solid. The mother liquor was concentrated in vacuo, and the residue was purified by flash column chromatography (silica gel, hexane/EtOAc = 7/3) and reprecipitation with hexane and CH_2Cl_2 afforded additional **19** (3rd crop: 1.19 g, 10%; 4th crop: 1.05 g, 8%; 5th crop: 197 mg, 2%; 6th crop: 132 mg, 1%; total yield = 92%) as an off-white solid.

19: R_f 0.35 (hexane/ Et_2O = 1/1); mp 132–135 °C; ^1H NMR (600 MHz, $CDCl_3$) δ 3.94 (s, 3H), 4.03 (s, 3H), 6.67 (d, 1H, J = 8.4 Hz), 6.74 (d, 1H, J = 8.4 Hz), 7.55 (d, 1H, J = 9.0 Hz), 7.60 (d, 1H, J = 9.0 Hz), 10.18 (s, 1H, OH); ^{13}C NMR (150 MHz, $CDCl_3$) δ 55.7, 56.6, 103.4, 104.7, 105.4, 114.1, 116.1, 127.2, 130.8, 149.0, 150.2, 150.5; IR (ATR) 3314, 2945, 2836, 1716, 1608, 1505, 1454, 1378, 1354, 1296, 1251, 1169, 1127, 1087, 1050, 971, 903, 811, 799, 769, 723 cm^{-1}; HRMS (ESI-TOF) calcd for $C_{12}H_{11}BrNaO_3$ ($[M+Na]^+$) *m/z* 304.9784, found *m/z* 304.9777.

General procedure for the etherification of bromonaphthol 19:

To a solution of bromonaphthol **19** in DMF was added base and R–X at 0 °C. After stirring at room temperature until **19** was completely consumed (monitored by TLC), to the reaction was added Et_2NH at room temperature. After stirring for several hours, the mixture was poured into mixed solvent of 1 M aqueous HCl and EtOAc. (When Cs_2CO_3 was used as base, the mixture was filtered through a Celite® pad before pouring to the mixed solvent.) The mixture was extracted with EtOAc (×3), and the combined organic extracts were washed with 1 M aqueous HCl, water, saturated aqueous $NaHCO_3$, and brine, dried (Na_2SO_4), and concentrated in vacuo. The residue was purified to afford bromonaphthalene **13**.

bromonaphthalene **13a**

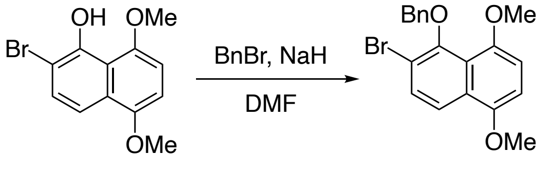

According to the general procedure, bromonaphthol **19** (500 mg, 1.77 mmol) in DMF (5.5 mL) was treated with NaH (63% dispersion in mineral oil, 94.0 mg, 2.46 mmol) and benzylbromide (220 μL, 1.85 mmol). After stirring for 2 h, the reaction was quenched and the product was extracted. The residue was purified by MPLC (High-flash 2L column, hexane/EtOAc = 20/1 → 5/1) to afford **13a** (545 mg, 82%) as a pale-yellow solid. Analytically pure sample was obtained by recrystallization (cold hexane) as pale-yellow needles.

13a: R_f 0.69 (hexane/EtOAc = 2/1); mp 105–106 °C (hexane); ^{1}H NMR (600 MHz, $CDCl_3$) δ 3.84 (s, 3H), 3.96 (s, 3H), 5.02 (s, 2H), 6.76 (d, 1H, *J* = 8.5 Hz), 6.83 (d, 1H, *J* = 8.5 Hz), 7.36 (t, 1H, *J* = 7.4 Hz), 7.43 (t, 2H, *J* = 7.4 Hz), 7.64 (d, 1H, *J* = 9.0 Hz), 7.66 (br-d, 2H, *J* = 7.4 Hz), 7.95 (d, 1H, *J* = 9.0 Hz); ^{13}C NMR (150 MHz, $CDCl_3$) δ 55.8, 57.0, 75.9, 104.3, 107.6, 116.7, 119.8, 122.5, 127.8, 128.0, 128.3, 128.3, 130.1, 137.7, 149.3, 149.8, 151.3; IR (ATR) 3031, 2992, 2956, 2935, 2869, 2833, 1618, 1577, 1498, 1448, 1410, 1338, 1315, 1260, 1091, 1139, 1091, 1051, 980, 916, 805, 777, 749, 728, 705, 696, 619 cm^{-1}; HRMS (ESI-TOF) calcd for $C_{19}H_{17}BrNaO_3$ ($[M+Na]^+$) *m/z* 395.0253, found *m/z* 395.0272.

bromonaphthalene **13b**

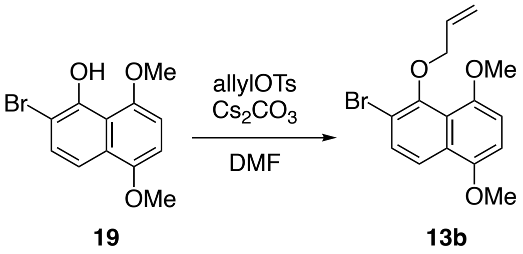

According to the general procedure, bromonaphthol **19** (302 mg, 1.07 mmol) in DMF (2 mL) was treated with Cs_2CO_3 (560 mg, 1.72 mmol) and allyltosylate (338 mg, 1.59 mmol). After stirring for 1.5 h, the reaction was quenched and the product was extracted. The residue was purified by MPLC (High-flash L column, hexane/EtOAc = 1/0 → 5/1) to afford **13b** (330 mg, 95%) as a pale-yellow oil.

13b: R_f 0.66 (hexane/EtOAc = 2/1); ^{1}H NMR (600 MHz, $CDCl_3$) δ 3.91 (s, 3H), 3.94 (s, 3H),

4.51 (ddd, 2H, J = 5.8, 1.6, 1.2 Hz), 5.31 (ddt, 1H, J = 10.4, 3.2, 1.2 Hz), 5.47 (ddt, 1H, J = 17.2, 3.2, 1.6 Hz), 6.21–6.31 (m, 1H), 6.73 (d, 1H, J = 8.5 Hz), 6.81 (d, 1H, J = 8.5 Hz), 7.60 (d, 1H, J = 9.1 Hz), 7.91 (d, 1H, J = 9.1 Hz); ^{13}C NMR (150 MHz, $CDCl_3$) δ 55.8, 57.1, 75.2, 104.2, 107.6, 116.5, 117.5, 119.6, 122.4, 127.9, 130.0, 134.1, 149.3, 149.7, 151.3; IR (neat) 3077, 2995, 2935, 2832, 1617, 1578, 1456, 1337, 1315, 1259, 1094, 1054, 990, 974, 927, 820, 804, 780, 729 cm^{-1}; HRMS (ESI-TOF) calcd for $C_{15}H_{15}BrNaO_3$ ([M+Na]$^+$) *m/z* 345.0097, found *m/z* 345.0104.

bromonaphthalene **13c**

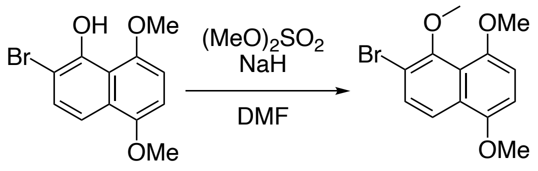

19 **13c**

According to the general procedure, bromonaphthol **19** (303 mg, 1.07 mmol) in DMF (3 mL) was treated with NaH (63% dispersion in mineral oil, 58.0 mg, 1.49 mmol) and dimethyl sulfate (120 μL, 1.27 mmol). After stirring for 30 min, the reaction was quenched and the product was extracted. The residue was purified by MPLC (High-flash L column, hexane/EtOAc = 1/0 → 5/1) to afford **13c** (303 mg, 96%) as a pale-yellow solid.

13c: R_f 0.55 (hexane/EtOAc = 2/1); mp 70–72 °C; ^{1}H NMR (600 MHz, $CDCl_3$) δ 3.89 (s, 3H), 3.942 (s, 3H), 3.944 (s, 3H), 6.74 (d, 1H, J = 8.4 Hz), 6.82 (d, 1H, J = 8.4 Hz), 7.59 (d, 1H, J = 9.0 Hz), 7.91 (d, 1H, J = 9.0 Hz); ^{13}C NMR (150 MHz, $CDCl_3$) δ 55.8, 57.2, 61.8, 104.3, 107.6, 116.3, 119.5, 122.3, 128.0, 130.0, 149.3, 149.8, 152.7; IR (ATR) 2991, 2938, 2832, 1619, 1578, 1454, 1405, 1340, 1313, 1258, 1138, 1093, 1054, 993, 971, 878, 817, 803, 781, 728, 695 cm^{-1}; HRMS (ESI-TOF) calcd for $C_{13}H_{13}BrNaO_3$ ([M+Na]$^+$) *m/z* 318.9940, found *m/z* 318.9942.

bromonaphthalene **13d**

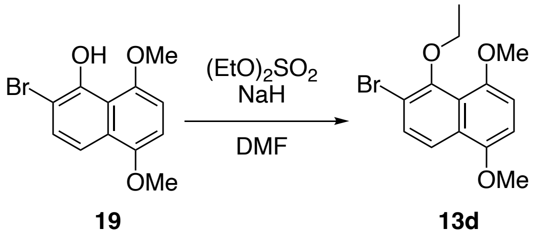

19 **13d**

According to the general procedure, bromonaphthol **19** (301 mg, 1.06 mmol) in DMF (3 mL) was

treated with NaH (63% dispersion in mineral oil, 59.0 mg, 1.55 mmol) and diethyl sulfate (166 μL, 1.27 mmol). After stirring for 6 h, the reaction was quenched and the product was extracted. The residue was purified by MPLC (High-flash L column, hexane/EtOAc = 1/0 → 5/1) to afford **13d** (302 mg, 92%) as a pale-yellow oil.

13d: R_f 0.55 (hexane/EtOAc = 2/1); ^{1}H NMR (600 MHz, $CDCl_3$) δ 1.52 (t, 3H, *J* = 7.0 Hz), 3.93 (s, 3H), 3.94 (s, 3H), 4.04 (q, 2H, *J* = 7.0 Hz), 6.73 (d, 1H, *J* = 8.5 Hz), 6.81 (d, 1H, *J* = 8.5 Hz), 7.60 (d, 1H, *J* = 9.1 Hz), 7.90 (d, 1H, *J* = 9.1 Hz); ^{13}C NMR (150 MHz, $CDCl_3$) δ 15.4, 55.8, 57.2, 70.3, 104.2, 107.9, 116.5, 119.3, 122.5, 128.0, 130.0, 149.4, 149.8, 151.7; IR (neat) 2977, 2935, 2832, 1617, 1578, 1454, 1410, 1339, 1259, 1094, 1055, 979, 820, 780, 728 cm^{-1}; HRMS (ESI-TOF) calcd for $C_{14}H_{15}BrNaO_3$ ([M+Na]$^+$) *m/z* 333.0097, found *m/z* 333.0097.

bromonaphthalene **13e**

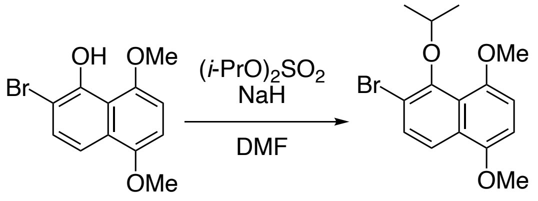

19 **13e**

According to the general procedure, bromonaphthol **19** (300 mg, 1.06 mmol) in DMF (3 mL) was treated with NaH (63% dispersion in mineral oil, 52.6 mg, 1.38 mmol) and diisopropyl sulfate (210 μL, 1.27 mmol). After stirring for 7 h, to the reaction mixture was added NaH (63% dispersion in mineral oil, 20.0 mg, 0.525 mmol) and diisopropyl sulfate (100 μL, 0.605 mmol). After stirring for 12 h, the reaction was quenched and the product was extracted. The residue was purified by MPLC (High-flash L column, hexane/EtOAc = 1/0 → 5/1) to afford **13e** (349 mg, quant.) as a pale-yellow oil.

13e: R_f 0.51 (hexane/EtOAc = 2/1); ^{1}H NMR (600 MHz, $CDCl_3$) δ 1.33 (d, 6H, *J* = 6.2 Hz), 3.91 (s, 3H), 3.94 (s, 3H), 4.53 (sept, 1H, *J* = 6.2 Hz), 6.72 (d, 1H, *J* = 8.5 Hz), 6.78 (d, 1H, *J* = 8.5 Hz), 7.60 (d, 1H, *J* = 9.0 Hz), 7.87 (d, 1H, *J* = 9.0 Hz); ^{13}C NMR (150 MHz, $CDCl_3$) δ 22.0, 55.8, 56.7, 77.9, 104.3, 107.3, 117.3, 118.8, 122.7, 128.0, 130.1, 149.3, 149.8, 150.2; IR (neat) 2976, 2934, 2833, 1617, 1576, 1454, 1408, 1328, 1259, 1136, 1106, 1092, 1053, 981, 940, 824, 803, 775, 729 cm^{-1}; HRMS (ESI-TOF) calcd for $C_{15}H_{17}BrNaO_3$ ([M+Na]$^+$) *m/z* 347.0253, found *m/z* 347.0250.

bromonaphthalene **13f**

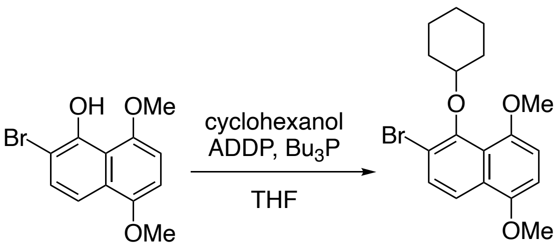

19 **13f**

To a solution of bromonaphthol **19** (296 mg, 1.16 mmol) and cyclohexanol (125 μL, 1.18 mmol) in THF (5.5 mL) was added 1,1'-(azodicarbonyl)dipiperidine (440 mg, 1.74 mmol) and tributylphosphine (430 μL, 1.74 mmol) at 0 °C. After stirring for 8.5 h at room temperature, to the reaction was further added cyclohexanol (60 μL, 0.568 mmol), 1,1'-(azodicarbonyl)dipiperidine (150 mg, 0.595 mmol), and tributylphosphine (150 μL, 0.608 mmol) at 0 °C. After stirring for 10 h at room temperature, the reaction was poured into mixed solvent of 1 M aqueous HCl and EtOAc. The mixture was extracted with EtOAc (×3), and the combined organic extracts were washed with water, saturated aqueous $NaHCO_3$, and brine, dried (Na_2SO_4), and concentrated in vacuo. The residue was purified by MPLC (High-flash L column, hexane/EtOAc = 1/0 → 5/1) to afford **13f** (348 mg, 82%) as a white solid.

13f: R_f 0.62 (hexane/EtOAc = 2/1); mp 106–108 °C; ^{1}H NMR (600 MHz, $CDCl_3$) δ 1.11–1.23 (m, 3H), 1.55–1.61 (m, 1H), 1.62–1.71 (m, 2H), 1.72–1.82 (m, 2H), 1.95–1.22 (m, 2H), 3.90 (s, 3H), 3.94 (s, 3H), 4.12 (tt, 1H, *J* = 10.8, 4.0 Hz), 6.73 (d, 1H, *J* = 8.5 Hz), 6.79 (d, 1H, *J* = 8.5 Hz), 7.59 (d, 1H, *J* = 9.0 Hz), 7.86 (d, 1H, *J* = 9.0 Hz); ^{13}C NMR (150 MHz, $CDCl_3$) δ 24.9, 25.7, 32.4, 55.8, 56.7, 84.3, 104.0, 107.3, 117.2, 118.7, 122.7, 128.0, 130.1, 149.4, 149.8, 150.1; IR (ATR) 2937, 2920, 2852, 2831, 1616, 1576, 1451, 1406, 1337, 1312, 1455, 1137, 1091, 1049, 1015, 979, 950, 900, 822, 805, 774, 730, 697 cm^{-1}; HRMS (ESI-TOF) calcd for $C_{18}H_{22}BrO_3$ ($[M+H]^+$) *m/z* 365.0747, found *m/z* 365.0739.

bromonaphthalene **13g**

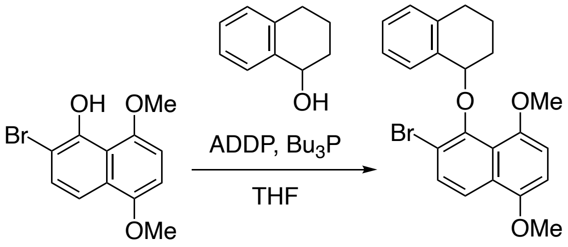

19 **13g**

To a solution of bromonaphthol **19** (300 mg, 1.18 mmol) and 1,2,3,4-tetrahydro-1-naphthol (270 mg, 1.83 mmol) in THF (6 mL) was added 1,1'-(azodicarbonyl)dipiperidine (465 mg, 1.84 mmol) and tributylphosphine (450 μL, 1.82 mmol) at 0 °C. After stirring for 12 h at room temperature, the reaction was poured into mixed solvent of 1 M aqueous HCl and EtOAc. The mixture was extracted with EtOAc (×3), and the combined organic extracts were washed with water, saturated aqueous $NaHCO_3$, and brine, dried (Na_2SO_4), and concentrated in vacuo. The residue was purified twice by MPLC (High-flash L column, first; hexane/EtOAc = 1/0 → 5/1, second; hexane/EtOAc = 1/0 → 5.6/1) to afford **13g** (136 mg, 28%) as a pale brown oil.

13g: R_f 0.58 (hexane/EtOAc = 2/1); ^{1}H NMR (600 MHz, $CDCl_3$) δ 1.81–1.95 (m, 2H), 2.39–2.49 (m, 1H), 2.49–2.56 (m, 1H), 2.83 (ddd, 1H, *J* = 16.9, 9.7, 6.0 Hz), 2.99 (dt, 1H, *J* = 16.9, 5.0 Hz), 3.80 (s, 3H), 3.96 (s, 3H), 5.24 (t, 1H, *J* = 4.1 Hz), 6.42 (d, 1H, *J* = 8.5 Hz), 6.71 (d, 1H, *J* = 8.5 Hz), 6.73 (d, 1H, *J* = 8.5 Hz), 6.81 (br-t, 1H, *J* = 8.5 Hz), 7.11 (d, 1H, *J* = 7.4 Hz), 7.15 (td, 1H, *J* = 7.4, 1.1 Hz), 7.50 (d, 1H, *J* = 9.0 Hz), 7.88 (d, 1H, *J* = 9.0 Hz); ^{13}C NMR (150 MHz, $CDCl_3$) δ 18.6, 29.2, 29.3, 55.7, 56.3, 80.4, 104.0, 106.4, 118.3, 118.8, 122.6, 124.6, 127.7, 127.9, 128.7, 129.4, 130.0, 134.5, 137.9, 149.2, 149.6, 149.6; IR (neat) 2998, 2938, 2832, 1616, 1576, 1455, 1409, 1352, 1334, 1310, 1259, 1093, 1051, 979, 948, 809, 803, 767, 729 cm^{-1}; HRMS (ESI-TOF) calcd for $C_{22}H_{21}BrNaO_3$ ([M+Na]$^+$) *m/z* 435.0566, found *m/z* 435.0577.

bromonaphthalene **13h**

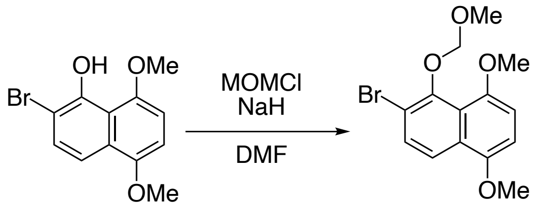

19 **13h**

According to the general procedure, bromonaphthol **19** (305 mg, 1.08 mmol) in DMF (3 mL) was treated with NaH (63% dispersion in mineral oil, 55.0 mg, 1.44 mmol) and MOMCl (100 μL, 1.32 mmol). After stirring for 30 min, the reaction was quenched and the product was extracted. The residue was purified by MPLC (High-flash L column, hexane/EtOAc = 1/0 → 5/1) to afford **13h** (352 mg, quant.) as a pale-yellow oil.

13h: R_f 0.43 (hexane/EtOAc = 2/1); ^{1}H NMR (600 MHz, $CDCl_3$) δ 3.75 (s, 3H), 3.92 (s, 3H), 3.94 (s, 3H), 5.12 (s, 2H), 6.74 (d, 1H, *J* = 8.5 Hz), 6.81 (d, 1H, *J* = 8.5 Hz), 7.61 (d, 1H, *J* = 9.1 Hz), 7.92 (d, 1H, *J* = 9.1 Hz); ^{13}C NMR (150 MHz, $CDCl_3$) δ 55.8, 56.9, 58.4, 101.0, 104.2, 107.5,

116.5, 119.8, 121.9, 128.0, 130.1, 148.9, 149.2, 149.8; IR (neat) 2994, 2937, 2833, 1617, 1579, 1456, 1142, 1342, 1259, 1160, 1138, 1094, 1045, 982, 949, 821, 805, 799, 728 cm^{-1}; HRMS (ESI-TOF) calcd for $C_{14}H_{15}BrNaO_4$ ($[M+Na]^+$) *m/z* 349.0046, found *m/z* 349.0039.

bromonaphthalene **13i**

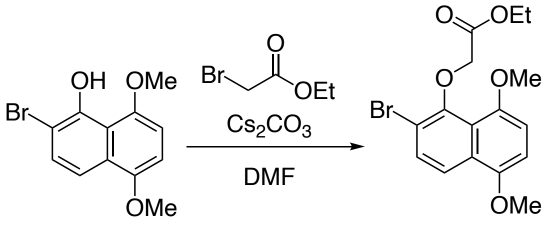

19 **13i**

According to the general procedure, bromonaphthol **19** (103 mg, 0.364 mmol) in DMF (1 mL) was treated with Cs_2CO_3 (345 mg, 1.06 mmol) and ethyl bromoacetate (80.0 μL, 0.721 mmol). After stirring for 20 min, the reaction was quenched and the product was extracted. The residue was purified by MPLC (High-flash L column, hexane/EtOAc = 1/0 → 3/1) to afford **13i** (129 mg, 96%) as a pale-yellow solid.

13i: R_f 0.38 (hexane/EtOAc = 2/1); mp 92–95 °C; ^1H NMR (600 MHz, $CDCl_3$) δ 1.37 (t, 3H, J = 7.1 Hz), 3.87 (s, 3H), 3.94 (s, 3H), 4.36 (q, 2H, J = 7.1 Hz), 4.60 (s, 2H), 6.74 (d, 1H, J = 8.5 Hz), 6.81 (d, 1H, J = 8.5 Hz), 7.58 (d, 1H, J = 9.1 Hz), 7.94 (d, 1H, J = 9.1 Hz); ^{13}C NMR (150 MHz, $CDCl_3$) δ 14.3, 55.8, 57.0, 61.0, 70.3, 104.4, 107.7, 116.3, 120.3, 121.9, 127.9, 129.9, 148.8, 149.7, 150.2, 168.9; IR (neat) 2976, 2938, 2904, 2834, 1762, 1732, 1618, 1580, 1456, 1411, 1344, 1260, 1197, 1098, 1071, 976, 822, 727 cm^{-1}; HRMS (ESI-TOF) calcd for $C_{16}H_{17}BrNaO_5$ ($[M+Na]^+$) *m/z* 391.0152, found *m/z* 391.0152.

bromonaphthalene **13j**

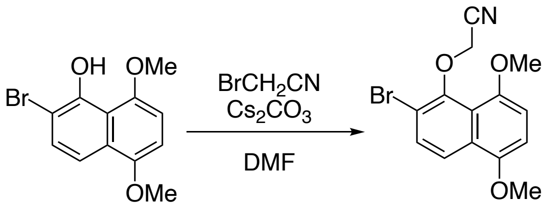

19 **13j**

According to the general procedure, bromonaphthol **19** (101 mg, 0.357 mmol) in DMF (1 mL) was treated with Cs_2CO_3 (345 mg, 1.06 mmol) and bromoacetonitrile (49.0 μL, 0.703 mmol).

After stirring for 2.5 h, the reaction was quenched and the product was extracted. The residue was purified by MPLC (High-flash L column, hexane/EtOAc = 9/1 → 3/1) to afford **13j** (107 mg, 93%) as a white solid.

13j: R$_f$ 0.38 (hexane/Et_2O = 2/1); mp 167–169 °C; ^{1}H NMR (600 MHz, $CDCl_3$) δ 3.95 (s, 3H), 3.99 (s, 3H), 4.78 (s, 2H), 6.78 (d, 1H, *J* = 8.5 Hz), 6.87 (d, 1H, *J* = 8.5 Hz), 7.59 (d, 1H, *J* = 9.1 Hz), 7.99 (d, 1H, *J* = 9.1 Hz); ^{13}C NMR (150 MHz, $CDCl_3$) δ 55.8, 56.9, 58.2, 104.7, 108.0, 115.7, 116.4, 121.3, 121.4, 127.9, 129.8, 148.4, 149.5, 149.7; IR (ATR) 2937, 2834, 1620, 1582, 1459, 1413, 1341, 1258, 1140, 1096, 1058, 1019, 976, 821, 797, 777, 727, 631 cm^{-1}; HRMS (ESI-TOF) calcd for $C_{14}H_{12}BrNNaO_3$ ([M+Na]$^+$) *m/z* 343.9893, found *m/z* 343.9893.

bromonaphthalene **13k**

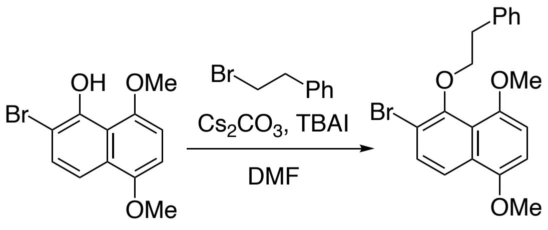

19 **13k**

According to the general procedure, bromonaphthol **19** (169 mg, 0.597 mmol) in DMF (2 mL) was treated with Cs_2CO_3 (345 mg, 1.06 mmol), TBAI (15.0 mg, 0.0406 mmol), and phenethyl bromide (96.0 μL, 0.716 mmol). After stirring for 14 h, the reaction was quenched and the product was extracted. The residue was purified by MPLC (High-flash L column, hexane/EtOAc = 1/0 → 7/1) to afford **13k** (202 mg, 87%) as a pale-yellow oil.

13k: R$_f$ 0.55 (hexane/Et_2O = 2/1); ^{1}H NMR (600 MHz, $CDCl_3$) δ 3.27 (t, 2H, *J* = 7.4 Hz), 3.81 (s, 3H), 3.94 (s, 3H), 4.17 (t, 2H, *J* = 7.4 Hz), 6.72 (d, 1H, *J* = 8.5 Hz), 6.79 (d, 1H, *J* = 8.5 Hz), 7.23 (br-tt, 1H, *J* = 7.3, 1.3 Hz), 7.29–7.33 (m, 2H), 7.35 (br-d, 2H, *J* = 6.9 Hz), 7.59 (d, 1H, *J* = 9.0 Hz), 7.90 (d, 1H, *J* = 9.0 Hz); ^{13}C NMR (150 MHz, $CDCl_3$) δ 36.7, 55.8, 57.0, 75.1, 104.2, 107.5, 116.5, 119.5, 122.4, 126.2, 127.9, 128.3, 129.2, 130.0, 138.6, 149.3, 149.7, 151.6; IR (neat) 3061, 3026, 2937, 2832, 1617, 1578, 1497, 1454, 1410, 1341, 1314, 1259, 1138, 1095, 1055, 1002, 977, 819, 804, 783, 749, 728, 700 cm^{-1}; HRMS (ESI-TOF) calcd for $C_{20}H_{20}BrO_3$ ([M+H]$^+$) *m/z* 387.0590, found *m/z* 387.0578.

bromonaphthalene **13l**

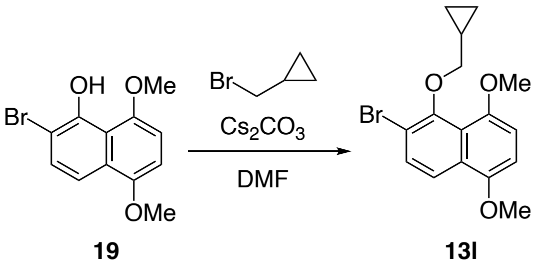

According to the general procedure, bromonaphthol **19** (304 mg, 1.07 mmol) in DMF (3 mL) was treated with Cs_2CO_3 (828 mg, 2.54 mmol) and cyclopropylmethyl bromide (125 μL, 1.29 mmol). After stirring for 15 h, the reaction was quenched and the product was extracted. The residue was purified by MPLC (High-flash 2L column, hexane/EtOAc = 1/0 → 7/1) to afford **13l** (301 mg, 95%) as a pale-yellow solid.

13l: R_f 0.62 (hexane/Et_2O = 2/1); mp 87–88 °C; ^{1}H NMR (600 MHz, $CDCl_3$) δ 0.38–0.47 (m, 2H), 0.61–0.71 (m, 2H), 1.42–1.52 (m, 1H), 3.81 (d, 2H, *J* = 7.1 Hz), 3.93 (s, 3H), 3.94 (s, 3H), 6.73 (d, 1H, *J* = 8.5 Hz), 6.81 (d, 1H, *J* = 8.5 Hz), 7.59 (d, 1H, *J* = 9.1 Hz), 7.89 (d, 1H, *J* = 9.1 Hz); ^{13}C NMR (150 MHz, $CDCl_3$) δ 3.3, 10.9, 55.8, 57.2, 79.3, 104.2, 107.7, 116.7, 119.4, 122.5, 128.0, 130.1, 149.4, 149.8, 151.4; IR (neat) 3080, 3001, 2937, 2832, 1617, 1578, 1455, 1411, 1353, 1332, 1259, 1138, 1094, 1054, 992, 973, 819, 804, 728 cm^{-1}; HRMS (ESI-TOF) calcd for $C_{16}H_{17}BrNaO_3$ ($[M+Na]^+$) *m/z* 359.0253, found *m/z* 359.0251.

acetal **8** (fluorescent light irradiation)

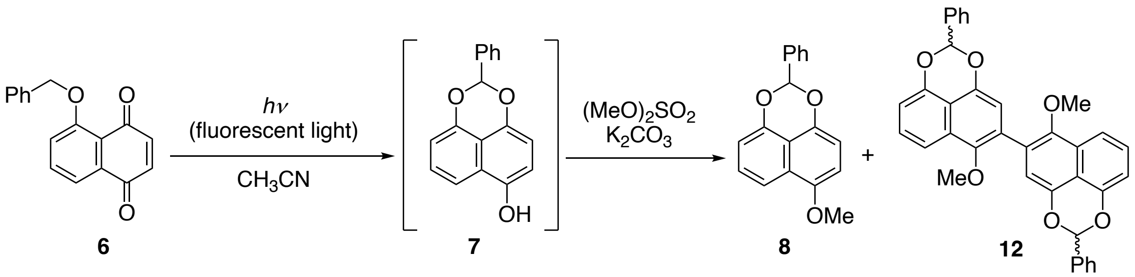

To a solution of naphthoquinone **6** (24.2 mg, 0.0914 mmol) in CH_3CN (9.1 mL) was placed in a pyrex® two necked round bottom flask, and degassed three times by purging with argon under sonication (×3). The solution was irradiated by fluorescent light (Panasonic FHF32EX-D-H, 32W; distance from flask: ca. 1 m; see figure S1a) at room temperature for 5.5 days. Then, to the solution was added dimethyl sulfate (40.0 μL, 0.423 mmol) and K_2CO_3 (100 mg, 0.724 mmol) at 0 °C. After stirring for 6 h, to the reaction was added Et_2NH (ca. 0.5 mL) at room temperature.

After stirring for 12 h, the mixture was filtered through Celite® pad and poured into water. The mixture was extracted with EtOAc (×3), and the combined organic extracts were washed with brine, dried (Na_2SO_4), and concentrated in vacuo. The residue was purified twice by PTLC (silica gel, 1st: hexane/EtOAc = 2/1, 2nd: hexane/EtOAc = 4/1) to afford acetal **8** (3.9 mg; 15%) as a white solid and dimeric acetal **12** (4.2 mg, 17%) as a colorless oil.

8: R_f 0.61 (hexane/EtOAc = 2/1); mp 151–154 °C; ^{1}H NMR (600 MHz, $CDCl_3$) δ 3.99 (s, 3H), 6.21 (s, 1H), 6.76 (d, 1H, J = 8.2 Hz), 6.89 (d, 1H, J = 8.2 Hz), 7.03 (dd, 1H, J = 7.6, 0.7 Hz), 7.44 (dd, 1H, J = 8.5, 7.6 Hz), 7.46–7.53 (m, 3H), 7.71–7.77 (m, 2H), 7.84 (dd, 1H, J = 8.5, 0.7 Hz); ^{13}C NMR (150 MHz, $CDCl_3$) δ 55.8, 99.6, 104.7, 107.8, 109.5, 115.2, 115.7, 126.4, 126.5, 126.8, 128.6, 129.9, 136.1, 144.0, 150.3, 150.4; IR (ATR) 3062, 3000, 1615, 1507, 1467, 1456, 1443, 1413, 1381, 1270, 1244, 1215, 1077, 1056, 1036, 1003, 963, 922, 912, 805, 772, 755, 714, 700 cm^{-1}; HRMS (ESI-TOF) calcd for $C_{18}H_{15}O_3$ ([M+H]$^+$) *m/z* 279.1074, found *m/z* 279.1001.

It was difficult to obtain a pure sample of dimer ***12****, and only the major peaks were identified because of its lability. Therefore, impure material was analyzed for the structure elucidation as shown below.*

Dimer 12 (mixture of diastereomers = 1:1): R_f 0.56 (hexane/EtOAc = 2/1); ^{1}H NMR (600 MHz, $CDCl_3$) δ 3.64 (s, 3H), 3.65 (s, 3H), 6.31 (s, 1H), 6.32 (s, 1H), 7.06 (d, 2H, J = 7.6 Hz), 7.05 (s, 1H), 7.09 (s, 1H), 7.47–7.55 (m, 8H), 7.74–7.78 (m, 4H), 7.84 (d, 2H, J = 8.4 Hz); ^{13}C NMR (150 MHz, $CDCl_3$) δ 61.7, 99.7, 109.3, 111.1, 115.2, 116.3, 126.8, 127.5, 128.1, 128.2, 128.7, 129.3, 129.3, 130.0, 135.9, 146.1, 148.2, 150.8; IR (neat) 3067, 2930, 2852, 1615, 1417, 1379, 1354, 1260, 1237, 1098, 1053, 1022, 961, 911, 758, 733, 697 cm^{-1}; HRMS (ESI-TOF) calcd for $C_{36}H_{27}O_6$ ([M+H]$^+$) *m/z* 555.1802, found *m/z* 555.1787.

key HMBC correlations

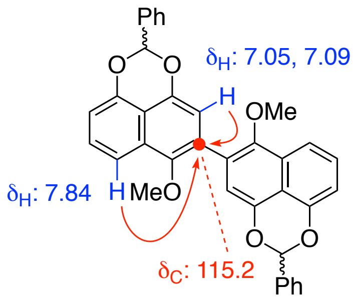

acetal **8** (LED irradiation)

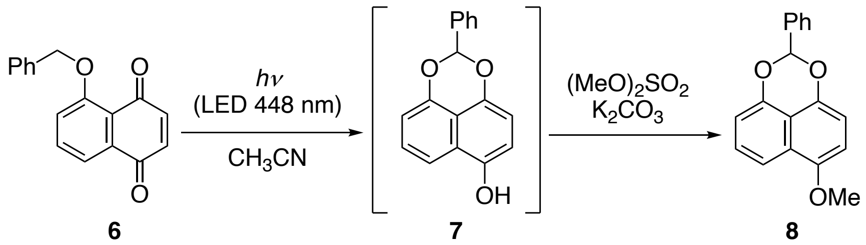

To a solution of naphthoquinone **6** (27.8 mg, 0.105 mmol) in CH_3CN (10 mL) was placed in a pyrex® two necked round bottom flask, and degassed three times by purging with argon under sonication. An LED lamp (Techno Sigma PER-AMP, PAR-448, 680 mW; see figure S1b) was inserted into the solution, and the flask was covered by an aluminum foil. The solution was irradiated at room temperature for 2 h. Then, to the solution was added dimethyl sulfate (50.0 μL, 0.528 mmol) and K_2CO_3 (145 mg, 1.05 mmol) at room temperature. After stirring for 15 h, to the reaction was added Et_2NH (ca. 0.5 mL) at room temperature. After stirring for 2 h, the mixture was filtered through Celite® pad and poured into mixed solvent of saturated aqueous NH_4Cl and EtOAc. The mixture was extracted with EtOAc (×3), and the combined organic extracts were washed with saturated aqueous NH_4Cl and brine, dried (Na_2SO_4), and concentrated in vacuo. The residue was purified twice by PTLC (silica gel, CH_2Cl_2 only) to afford acetal **8** (12.3 mg, 42%) as an off-white solid and dimeric acetal **12** (3.2 mg: impure, ca. 11%) as a pale-yellow oil.

a) b)

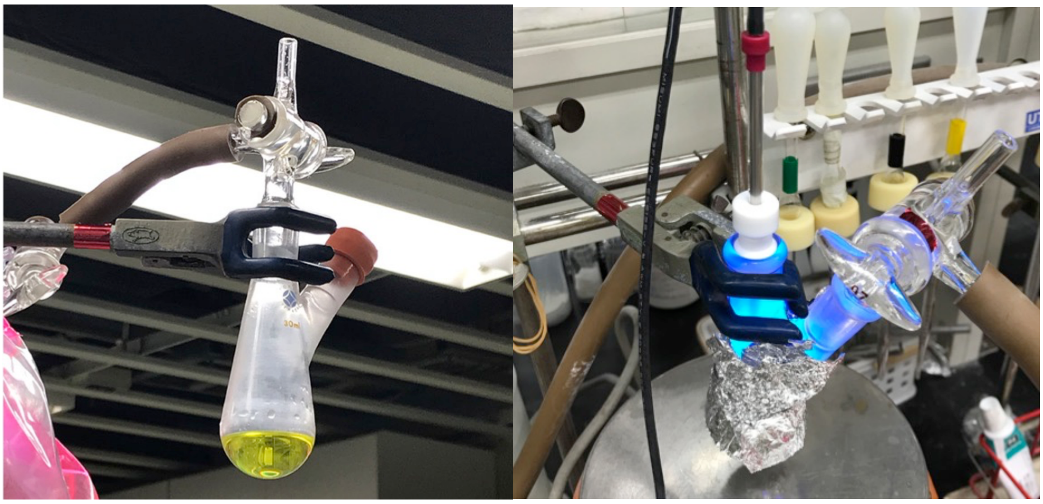

Figure S1

acetal **11a**

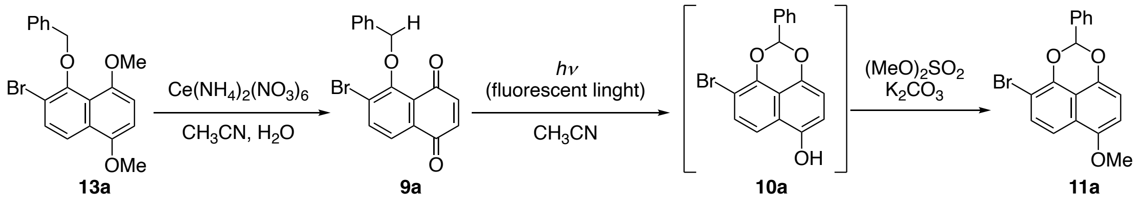

To a solution of **13a** (28.3 mg, 0.0758 mmol) in CH_3CN (3 mL) in a brown round bottom flask was added a solution of ammonium cerium(IV) nitrate (89.6 mg, 0.163 mmol) in water (0.7 mL) dropwise at 0 ºC. After stirring for 5 min at the same temperature, the reaction was stopped by adding saturated aqueous $NaHCO_3$ at 0 ºC. The mixture was poured into water and extracted with EtOAc (×3). The combined organic extracts were washed with brine, dried (Na_2SO_4), and concentrated in vacuo. To a solution of this crude material, including naphthoquinone **9a**, in CH_3CN (7.5 mL) was placed in a pyrex® two necked round bottom flask, and degassed three times by purging with argon under sonication. The solution was irradiated by fluorescent light (Panasonic FHF32EX-D-H, 32W; distance from flask: ca. 1 m; see figure S1a) at room temperature for 2 days. Then, to the solution was added dimethyl sulfate (36.0 μL, 0.379 mmol) and K_2CO_3 (105 mg, 0.758 mmol) at room temperature. After stirring for 15 h, to the reaction was added Et_2NH (ca. 0.5 mL) at room temperature. After stirring for 1 h, the mixture was filtered through Celite® pad and poured into mixed solvent of saturated aqueous NH_4Cl and EtOAc. The mixture was extracted with EtOAc (×3), and the combined organic extracts were washed with saturated aqueous NH_4Cl and brine, dried (Na_2SO_4), and concentrated in vacuo. The residue was purified twice by PTLC (silica gel, CH_2Cl_2 only) to afford acetal **11a** (21.6 mg, 80%) as a white solid.

Naphthoquinone 9a (crude): R_f 0.60 (hexane/EtOAc = 2/1); ^{1}H NMR (600 MHz, $CDCl_3$) δ 5.05 (s, 2H), 6.91 (d, 1H, *J* = 10.3 Hz), 6.94 (d, 1H, *J* = 10.3 Hz), 7.38 (br-t, 1H, *J* = 7.3 Hz), 7.44 (br-dd, 2H, *J* = 7.3, 7.2 Hz), 7.70 (br-d, 2H, *J* = 7.2 Hz), 7.82 (d, 1H, *J* = 8.3 Hz), 7.99 (d, 1H, *J* = 8.3 Hz); ^{13}C NMR (150 MHz, $CDCl_3$) δ 75.6, 124.1, 125.8, 128.0, 128.5 (2C), 128.9, 133.0, 136.1, 136.9, 138.6, 140.1, 155.2, 183.5, 184.2; IR (ATR) 3067, 2941, 2890, 1662, 1615, 1567, 1447, 1414, 1381, 1360, 1312, 1282, 1260, 1221, 1105, 970, 904, 834, 805, 749, 697, 658 cm^{-1}; UV–Vis (CH_3CN) λ_{max} nm (ε) = 358 (4155), 250 (20818), 207 (33591); HRMS (ESI-TOF) calcd for $C_{17}H_{11}BrNaO_3$ ([M+Na]$^+$) *m/z* 364.9784, found *m/z* 364.9771.

11a: R_f 0.55 (hexane/EtOAc = 2/1); mp 140–141 °C; ^{1}H NMR (600 MHz, $CDCl_3$) δ 3.97 (s, 3H), 6.28 (s, 1H), 6.77 (d, 1H, *J* = 8.3 Hz), 6.95 (d, 1H, *J* = 8.3 Hz), 7.45–7.57 (m, 3H), 7.57 (d, 1H, *J* = 9.0 Hz), 7.72 (d, 1H, *J* = 9.0 Hz), 7.74–7.78 (m, 2H); ^{13}C NMR (150 MHz, $CDCl_3$) δ 55.8, 99.7,

103.5, 105.0, 109.1, 116.0, 116.9, 125.1, 126.8, 128.6, 129.9, 130.0, 135.6, 142.8, 146.9, 150.4; IR (ATR) 3076, 2934, 2835, 1634, 1607, 1586, 1499, 1463, 1413, 1383, 1367, 1353, 1321, 1260, 1243, 1218, 1140, 1055, 1009, 951, 920, 820, 804, 777, 765, 741, 714, 696, 665, 639 cm^{-1}; HRMS (ESI-TOF) calcd for $C_{18}H_{14}BrO_3$ ($[M+H]^+$) *m/z* 357.0121, found *m/z* 357.0117.

General procedure for the oxidation of bromonaphthalene 13 followed by photochemical reaction of naphthoquinone 9 and methylation.

To a solution of bromonaphthalene **13** in CH_3CN in a brown round bottom flask was added ammonium cerium(IV) nitrate in water dropwise at 0 °C. After stirring for 5 min at 0 °C, the reaction was quenched by adding saturated aqueous $NaHCO_3$ and poured into mixed solvent of water and EtOAc. The mixture was extracted with EtOAc (×3), using a brown separatory funnel, and the combined organic extracts were washed with brine, dried (Na_2SO_4), and concentrated in vacuo using a brown round bottom flask. [Note: Since the naphthoquinone is labile under ambient light, light irradiation should be avoided by using a brown flask or an aluminum foil as a cover for a normal flask before photoreaction. Data for naphthoquinones were obtained by analyzing the crude material with careful manipulation.] To a solution of this crude material, including naphthoquinone **9**, in CH_3CN was placed in a pyrex® two necked round bottom flask, and degassed three times by purging with argon under sonication. An LED lamp (Techno Sigma PER-AMP, PAR-448, 680 mW; see figure S1) was inserted into the solution, and the flask was covered by an aluminum foil. The solution was irradiated at room temperature until the naphthoquinone was completely consumed. Then, to the solution was added dimethyl sulfate and K_2CO_3 at room temperature. After the phenol was completely consumed, to the reaction was added Et_2NH at room temperature. After several hours, the mixture was filtered through Celite® pad and poured into mixed solvent of saturated aqueous NH_4Cl and EtOAc. The mixture was extracted with EtOAc (×3), and the combined organic extracts were washed with saturated aqueous NH_4Cl and brine, dried (Na_2SO_4), and concentrated in vacuo. The residue was purified twice by PTLC to afford acetal **11**.

Note: In all experiments, the corresponding dimers were detected in ca. <5% yield, and the structures of dimers were not fully verified due to difficulty to obtain as pure form (perhaps its lability).

acetal **11a**

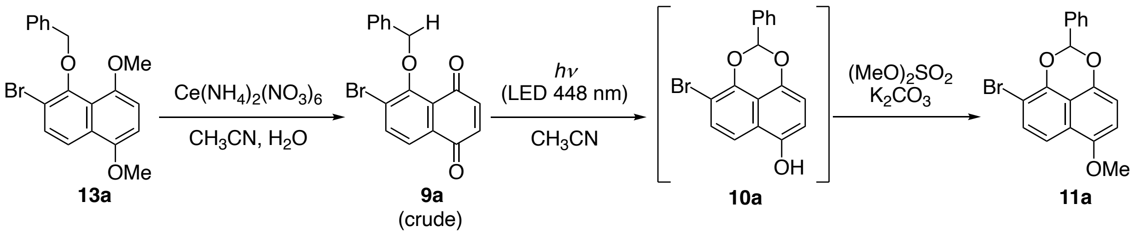

According to the general procedure, bromonaphthalene **13a** (28.2 mg, 0.0756 mmol) in CH_3CN (3 mL) was treated with ammonium cerium(IV) nitrate (91.6 mg, 0.167 mmol) in water (0.7 mL). Obtained naphthoquinone **9a** in CH_3CN (7.5 mL) was irradiated for 20 min and then added dimethyl sulfate (36.0 μL, 0.380 mmol) and K_2CO_3 (104 mg, 0.756 mmol). After stirring for 5.5 h, to the reaction was added Et_2NH (ca. 0.5 mL). After workup, the residue was purified by PTLC (silica gel, CH_2Cl_2 only) to afford **11a** (22.7 mg, 84%) as a white solid.

acetal **11b**

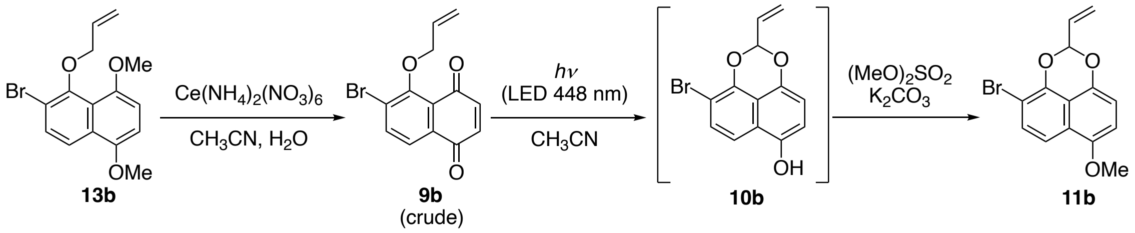

According to the general procedure, bromonaphthalene **13b** (45.7 mg, 0.141 mmol) in CH_3CN (3 mL) was treated with ammonium cerium(IV) nitrate (170 mg, 0.310 mmol) in water (1 mL). Obtained naphthoquinone **9b** in CH_3CN (14 mL) was irradiated for 20 min and then added dimethyl sulfate (66.0 μL, 0.697 mmol) and K_2CO_3 (195 mg, 1.41 mmol). After stirring for 16 h, to the reaction was added Et_2NH (ca. 0.5 mL). After workup, the residue was purified by PTLC (silica gel, CH_2Cl_2 only) to afford **7** (30.7 mg, 71%) as a white solid and alcohol **11b** (3.7 mg, impure, ca. 9%) as a pale brown oil.

Naphthoquinone 9b: R_f 0.53 (hexane/EtOAc = 2/1); ^{1}H NMR (600 MHz, $CDCl_3$) δ 4.57 (d, 2H, *J* = 5.9 Hz), 5.33 (dd, 1H, *J* = 10.3, 0.8 Hz), 5.47 (dd, 1H, *J* = 17.2, 1.4 Hz), 6.20–6.30 (m, 1H), 6.89 (d, 1H, *J* = 10.3 Hz), 6.93 (d, 1H, *J* = 10.3 Hz), 7.79 (d, 1H, *J* = 8.3 Hz), 7.96 (d, 1H, *J* = 8.3 Hz); ^{13}C NMR (150 MHz, $CDCl_3$) δ 75.2, 119.0, 123.9, 125.5, 127.9, 132.9, 133.0, 136.8, 138.5, 140.1, 155.4, 183.5, 184.2; IR (ATR) 3051, 2979, 2940, 1667, 1654, 1608, 1566, 1550, 1448, 1403, 1352, 1322, 1281, 1220, 1121, 1106, 983, 976, 925, 886, 866, 839, 809, 797, 654 cm^{-1};

UV–Vis (CH_3CN) λ_{max} nm (ε) = 359 (4512), 250 (21404), 207 (31339); HRMS (ESI-TOF) calcd for $C_{13}H_9BrNaO_3$ ($[M+Na]^+$) *m/z* 314.9627, found *m/z* 314.9609.

11b: R_f 0.55 (hexane/EtOAc = 2/1); mp 83–86 °C; ^{1}H NMR (600 MHz, $CDCl_3$) δ 3.96 (s, 3H), 5.60 (dt, 1H, *J* = 10.7, 1.0 Hz), 5.74 (dt, 1H, *J* = 4.6, 1.0 Hz), 5.83 (dt, 1H, *J* = 17.4, 1.0 Hz), 6.47 (ddd, 1H, *J* = 17.4, 10.7, 4.6 Hz), 6.75 (d, 1H, *J* = 8.2 Hz), 6.89 (d, 1H, *J* = 8.2 Hz), 7.54 (d, 1H, *J* = 9.0 Hz), 7.69 (d, 1H, *J* = 9.0 Hz); ^{13}C NMR (150 MHz, $CDCl_3$) δ 55.8, 98.9, 103.2, 105.0, 108.9, 115.9, 116.8, 121.7, 125.1, 130.0, 132.0, 142.4, 146.5, 150.4; IR (ATR) 2986, 2936, 2829, 1608, 1583, 1500, 1460, 1441, 1416, 1368, 1355, 1256, 1237, 1166, 1125, 1069, 1058, 946, 926, 841, 799, 765, 715, 659 cm^{-1}; HRMS (ESI-TOF) calcd for $C_{14}H_{12}BrO_3$ ($[M+H]^+$) *m/z* 306.9964, found *m/z* 306.9958.

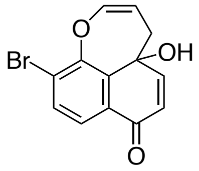

14

14: R_f 0.20 (hexane/EtOAc = 2/1); ^{1}H NMR (600 MHz, $CDCl_3$) δ 2.51 (dt, 1H, *J* = 17.9, 2.6 Hz), 2.71 (ddd, 1H, *J* = 17.9, 6.0, 1.5 Hz), 4.97 (ddd, 1H, *J* = 7.0, 6.0, 2.6 Hz), 6.40 (d, 1H, *J* = 10.3, Hz), 6.71(ddd, 1H, *J* = 7.0, 2.6, 1.5 Hz), 6.92 (d, 1H, *J* = 10.3 Hz), 7.70 (d, 1H, *J* = 8.4 Hz), 7.80 (d, 1H, *J* = 8.4 Hz); ^{13}C NMR (150 MHz, $CDCl_3$) δ 39.0, 69.3, 106.7, 121.8, 123.9, 127.4, 139.5, 133.3, 137.2, 142.1, 150.8, 153.3, 182.8; IR (ATR) 3387, 2924, 1668, 1630, 1579, 1559, 1456, 1421, 1392, 1299, 1218, 1117, 1087, 1041, 1013, 854, 811, 736 cm^{-1}; HRMS (ESI-TOF) calcd for $C_{13}H_{10}BrO_3$ ($[M+H]^+$) *m/z* 292.9808, found *m/z* 292.9794.

key HMBC correlations

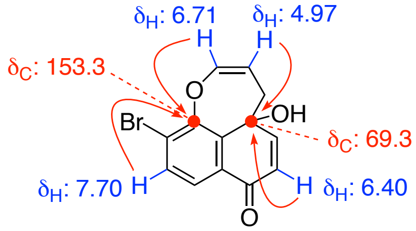

acetal **11c**

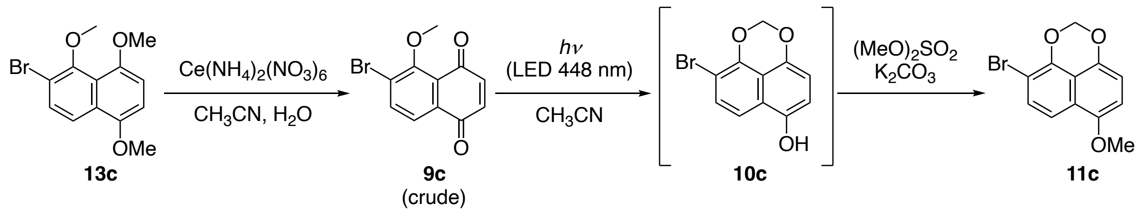

According to the general procedure, bromonaphthalene **13c** (27.6 mg, 0.0935 mmol) in CH_3CN (2 mL) was treated with ammonium cerium(IV) nitrate (109 mg, 0.199 mmol) in water (0.7 mL). Obtained naphthoquinone **9c** in CH_3CN (9.4 mL) was irradiated for 3.5 h and then added dimethyl sulfate (45.0 μL, 0.476 mmol) and K_2CO_3 (129 mg, 0.935 mmol). After stirring for 14.5 h, to the reaction was added Et_2NH (ca. 0.5 mL). After workup, the residue was purified by PTLC (silica gel, CH_2Cl_2 only) to afford **11c** (6.7 mg, 25%) as a white solid.

Naphthoquinone 9c: R_f 0.50 (hexane/EtOAc = 2/1); ^{1}H NMR (600 MHz, $CDCl_3$) δ 3.95 (s, 3H), 6.90 (d, 1H, J = 10.3 Hz), 6.93 (d, 1H, J = 10.3 Hz), 7.79 (d, 1H, J = 8.3 Hz), 7.97 (d, 1H, J = 8.3 Hz); ^{13}C NMR (150 MHz, $CDCl_3$) δ 61.7, 123.8, 125.3, 127.6, 133.0, 136.8, 138.5, 140.1, 156.8, 183.4, 184.2; IR (ATR) 3071, 2947, 1659, 1610, 1565, 1454, 1396, 1365, 1315, 1283, 1266, 1226, 1127, 1106, 1002, 899, 839, 800, 738, 638 cm^{-1}; UV–Vis (CH_3CN) λ_{max} nm (ε) = 357 (4038), 250 (18792), 207 (28653); HRMS (ESI-TOF) calcd for $C_{11}H_8BrO_3$ ($[M+H]^+$) *m/z* 266.9651, found *m/z* 266.9645.

11c: R_f 0.55 (hexane/EtOAc = 2/1); mp 118–122 °C; ^{1}H NMR (600 MHz, $CDCl_3$) δ 3.96 (s, 3H), 5.58 (s, 2H), 6.74 (d, 1H, J = 8.2 Hz), 6.87 (d, 1H, J = 8.2 Hz), 7.54 (d, 1H, J = 9.0 Hz), 7.70 (d, 1H, J = 9.0 Hz); ^{13}C NMR (150 MHz, $CDCl_3$) δ 55.8, 91.6, 103.0, 105.0, 108.8, 116.2, 117.0, 125.3, 129.9, 142.4, 146.6, 150.4; IR (ATR) 2935, 2835, 1633, 1609, 1584, 1502, 1462, 1443, 1416, 1358, 1338, 1260, 1244, 1159, 1117, 1064, 1050, 997, 972, 845, 808, 773, 730, 701, 661, 630 cm^{-1}; HRMS (ESI-TOF) calcd for $C_{12}H_{10}BrO_3$ ($[M+H]^+$) *m/z* 280.9808, found *m/z* 280.9800.

acetal **11d**

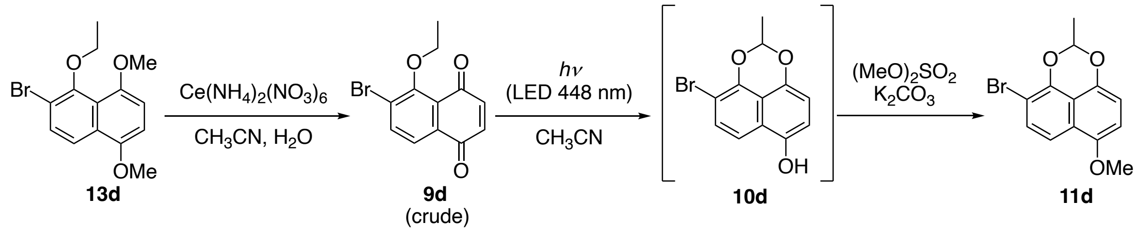

According to the general procedure, bromonaphthalene **13d** (23.3 mg, 0.0749 mmol) in CH_3CN (1.5 mL) was treated with ammonium cerium(IV) nitrate (91.8 mg, 0.167 mmol) in water (0.5 mL). Obtained naphthoquinone **9d** in CH_3CN (7.5 mL) was irradiated for 1 h and then added dimethyl sulfate (35.5 μL, 0.375 mmol) and K_2CO_3 (104 mg, 0.750 mmol). After stirring for 3.5 h, to the reaction was added Et_2NH (ca. 0.5 mL). After workup, the residue was purified twice by PTLC (silica gel, CH_2Cl_2 only) to afford **11d** (12.4 mg, 56%) as a white solid.

Naphthoquinone 9d: R_f 0.46 (hexane/EtOAc = 2/1); ^{1}H NMR (600 MHz, $CDCl_3$) δ 1.56 (t, 3H, J = 7.0 Hz), 4.08 (q, 2H, J = 7.0 Hz), 6.88 (d, 1H, J = 10.3 Hz), 6.92 (d, 1H, J = 10.3 Hz), 7.77 (d, 1H, J = 8.3 Hz), 7.96 (d, 1H, J = 8.3 Hz); ^{13}C NMR (150 MHz, $CDCl_3$) δ 15.4, 70.7, 123.7, 125.4, 127.9, 133.0, 136.8, 138.4, 140.2, 155.9, 183.5, 184.3; IR (ATR) 3066, 2977, 1661, 1612, 1566, 1439, 1418, 1386, 1313, 1286, 1260, 1213, 1163, 1103, 954, 859, 841, 807, 799, 733, 646 cm^{-1}; UV–Vis (CH_3CN) λ_{max} nm (ε) = 359 (3854), 250 (17818), 207 (27663); HRMS (ESI-TOF) calcd for $C_{12}H_9BrNaO_3$ ($[M+Na]^+$) *m/z* 302.9627, found *m/z* 302.9626.

11d: R_f 0.58 (hexane/EtOAc = 2/1); mp 123.7–124.4 °C; ^{1}H NMR (600 MHz, $CDCl_3$) δ 1.83 (d, 3H, J = 5.1 Hz), 3.95 (s, 3H), 5.43 (t, 1H, J = 5.1 Hz), 6.74 (d, 1H, J = 8.3 Hz), 6.83 (d, 1H, J = 8.3 Hz), 7.53 (d, 1H, J = 9.0 Hz), 7.67 (d, 1H, J = 9.0 Hz); ^{13}C NMR (150 MHz, $CDCl_3$) δ 20.3, 55.8, 98.3, 102.8, 105.0, 108.5, 115.8, 116.6, 125.1, 130.0, 143.0, 147.1, 150.2; IR (ATR) 2940, 2834, 1632, 1612, 1586, 1503, 1467, 1414, 1359, 1247, 1153, 1082, 1043, 923, 906, 866, 839, 801, 765, 741, 674, 514 cm^{-1}; HRMS (ESI-TOF) calcd for $C_{13}H_{12}BrO_3$ ($[M+H]^+$) *m/z* 294.9964, found *m/z* 294.9954.

acetal **11e**

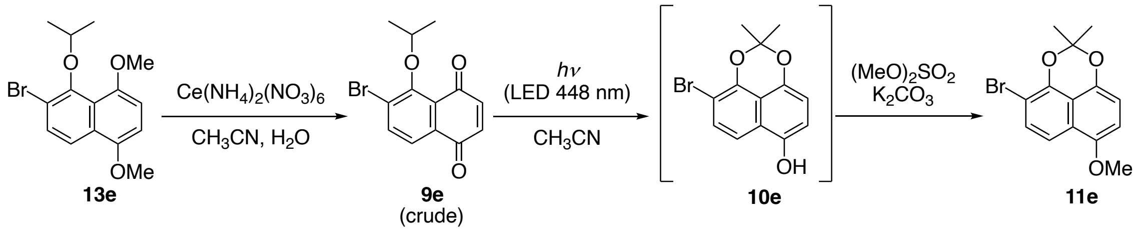

According to the general procedure, bromonaphthalene **13e** (24.4 mg, 0.0750 mmol) in CH_3CN (1.5 mL) was treated with ammonium cerium(IV) nitrate (89.0 mg, 0.162 mmol) in water (0.5 mL). Obtained naphthoquinone **9e** in CH_3CN (7.5 mL) was irradiated for 15 min and then added dimethyl sulfate (35.5 μL, 0.375 mmol) and K_2CO_3 (104 mg, 0.750 mmol). After stirring for 19 h, to the reaction was added Et_2NH (ca. 0.5 mL). After workup, the residue was purified twice by

PTLC (silica gel, CH_2Cl_2 only) to afford **11e** (18.2 mg, 78%) as a pale-yellow oil.

Naphthoquinone 9e: R_f 0.51 (hexane/EtOAc = 2/1); ^{1}H NMR (600 MHz, $CDCl_3$) δ 1.40 (d, 6H, J = 6.1 Hz), 4.51 (sept, 1H, J = 6.1 Hz), 6.87 (d, 1H, J = 10.3 Hz), 6.91 (d, 1H, J = 10.3 Hz), 7.74 (d, 1H, J = 8.3 Hz), 7.96 (d, 1H, J = 8.3 Hz); ^{13}C NMR (150 MHz, $CDCl_3$) δ 22.4, 79.4, 123.2, 125.4, 128.5, 133.2, 136.6, 138.5, 140.3, 154.9, 183.6, 184.4; IR (ATR) 3073, 2980, 2934, 1656, 1617, 1566, 1442, 1403, 1370, 1313, 1280, 1261, 1217, 1163, 1122, 1095, 961, 895, 864, 845, 805, 795, 640 cm^{-1}; UV–Vis (CH_3CN) λ_{max} nm (ε) = 368 (4289), 251 (20251), 210 (29937); HRMS (ESI-TOF) calcd for $C_{13}H_{11}BrNaO_3$ ($[M+Na]^+$) *m/z* 316.9784, found *m/z* 316.9777.

11e: R_f 0.50 (hexane/EtOAc = 2/1); ^{1}H NMR (600 MHz, $CDCl_3$) δ 1.67 (s, 6H), 3.95 (s, 3H), 6.74 (d, 1H, J = 8.2 Hz), 6.80 (d, 1H, J = 8.2 Hz), 7.53 (d, 1H, J = 9.0 Hz), 7.65 (d, 1H, J = 9.0 Hz); ^{13}C NMR (150 MHz, $CDCl_3$) δ 25.2, 55.7, 102.5, 103.8, 105.3, 109.3, 114.8, 116.1, 124.7, 130.2, 140.5, 144.8, 150.0; IR (neat) 2996, 2940, 2835, 1630, 1606, 1585, 1500, 1416, 1387, 1365, 1258, 1245, 1201, 1108, 1060, 966, 918, 852, 816, 806, 771, 742 cm^{-1}; HRMS (ESI-TOF) calcd for $C_{14}H_{14}BrO_3$ ($[M+H]^+$) *m/z* 309.0121, found *m/z* 309.0113.

acetal **11f**

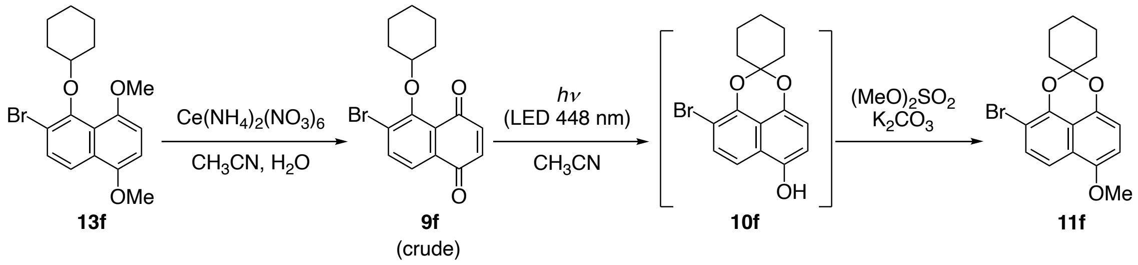

According to the general procedure, bromonaphthalene **13f** (35.0 mg, 0.0958 mmol) in CH_3CN (4 mL) was treated with ammonium cerium(IV) nitrate (122 mg, 0.223 mmol) in water (0.5 mL). Obtained naphthoquinone **9f** in CH_3CN (9.6 mL) was irradiated for 15 min and then added dimethyl sulfate (45.0 μL, 0.476 mmol) and K_2CO_3 (132 mg, 0.955 mmol). After stirring for 4 h, to the reaction was added Et_2NH (ca. 0.5 mL). After workup, the residue was purified by PTLC (silica gel, hexane/EtOAc = 7/3) to afford **11f** (28.0 mg, 84%) as a pale brown oil.

Naphthoquinone 9f: R_f 0.74 (hexane/EtOAc = 2/1); ^{1}H NMR (600 MHz, $CDCl_3$) δ 1.15–1.28 (m, 3H), 1.54–1.62 (m, 1H), 1.63–1.73 (m, 2H), 1.76–1.85 (m, 2H), 2.03–2.11 (m, 2H), 4.12 (tt, 1H, J = 10.5, 4.1 Hz), 6.87 (d, 1H, J = 10.2 Hz), 6.91 (d, 1H, J = 10.2 Hz), 7.73 (d, 1H, J = 8.3 Hz), 7.95 (d, 1H, J = 8.3 Hz); ^{13}C NMR (150 MHz, $CDCl_3$) δ 24.7, 25.3, 32.6, 85.3, 123.1, 125.4,

128.4, 133.2, 136.6, 138.4, 140.4, 154.9, 183.6, 184.4; IR (ATR) 2925, 2845, 1660, 1614, 1567, 1444, 1407, 1364, 1314, 1267, 1222, 1124, 1108, 1007, 973, 911, 834, 809, 791, 725, 650 cm^{-1}; UV–Vis (CH_3CN) λ_{max} nm (ε) = 371 (3227), 251 (15526), 211 (23253); HRMS (ESI-TOF) calcd for $C_{16}H_{15}BrNaO_3$ ($[M+Na]^+$) *m/z* 357.0097, found *m/z* 357.0093.

11f: R_f 0.65 (hexane/EtOAc = 2/1); ^1H NMR (600 MHz, $CDCl_3$) δ 1.41–1.51 (m, 1H), 1.53–1.66 (m, 1H), 1.66–1.79 (m, 4H), 1.79–1.86 (m, 2H), 1.95–2.02 (m, 2H), 3.94 (s, 3H), 6.73 (d, 1H, *J* = 8.2 Hz), 6.81 (d, 1H, *J* = 8.2 Hz), 7.52 (d, 1H, *J* = 9.0 Hz), 7.64 (d, 1H, *J* = 9.0 Hz); ^{13}C NMR (150 MHz, $CDCl_3$) δ 22.4, 25.1, 33.8, 55.7, 103.2, 103.9, 105.3, 109.2, 115.2, 116.1, 124.6, 130.1, 140.3, 144.5, 149.9; IR (neat) 2937, 2861, 1607, 1585, 1499, 1459, 1445, 1415, 1373, 1260, 1237, 1126, 1085, 1057, 929, 811, 789, 769, 743 cm^{-1}; HRMS (ESI-TOF) calcd for $C_{17}H_{18}BrO_3$ ($[M+H]^+$) *m/z* 349.0434, found *m/z* 349.0418.

acetal **11g**

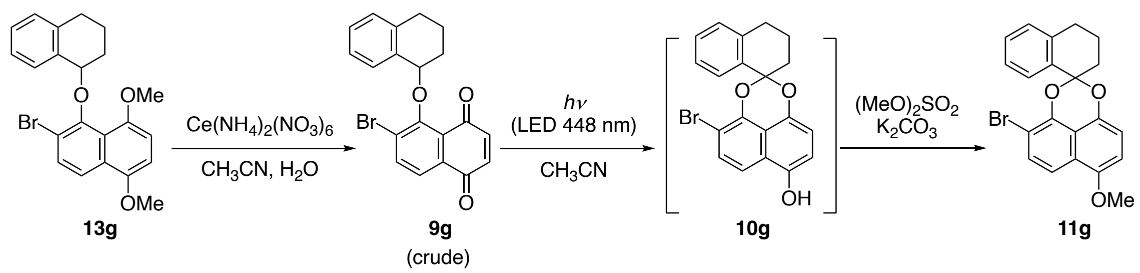

According to the general procedure, bromonaphthalene **13g** (32.4 mg, 0.0784 mmol) in CH_3CN (4 mL) was treated with ammonium cerium(IV) nitrate (97.3 mg, 0.177 mmol) in water (0.5 mL). Obtained naphthoquinone **9g** in CH_3CN (7.8 mL) was irradiated for 15 min and then added dimethyl sulfate (37.0 μL, 0.391 mmol) and K_2CO_3 (108 mg, 0.784 mmol). After stirring for 2.5 h, to the reaction was added Et_2NH (ca. 0.5 mL). After workup, the residue was purified by PTLC (silica gel, CH_2Cl_2 only) to afford a mixture of **11g** and the corresponding dimer (23.7 mg) as a white solid (**11g**:dimer = 10:1, **11g**; 69%, dimer; 7% (NMR yield)). Analytically pure sample was obtained by trituration with EtOAc.

Naphthoquinone 9g: R_f 0.55(hexane/EtOAc = 2/1); ^1H NMR (600 MHz, $CDCl_3$) δ 1.85–2.00 (m, 2H), 2.44–2.55 (m, 1H), 2.60–2.68 (m, 1H), 2.82 (ddd, 1H, *J* = 17.0, 10.5, 6.1 Hz), 2.98–3.05 (m, 1H), 5.20 (dd, 1H, *J* = 3.7, 3.5 Hz), 6.56 (d, 1H, *J* = 7.5 Hz), 6.73 (d, 1H, *J* = 10.3 Hz), 6.87 (d, 1H, *J* = 10.3 Hz), 6.88 (t, 1H, *J* = 7.5 Hz), 7.13 (d, 1H, *J* = 7.6 Hz), 7.18 (ddd, 1H, *J* = 7.6, 7.5, 1.1 Hz), 7.75 (d, 1H, *J* = 8.2 Hz), 7.88 (d, 1H, *J* = 8.2 Hz); ^{13}C NMR (150 MHz, $CDCl_3$) δ 18.1,

28.9, 29.3, 81.4, 123.4, 124.8, 126.1, 128.4, 129.2, 129.3, 129.4, 132.9, 133.5, 136.5, 138.3, 138.5, 140.3, 154.3, 183.3, 184.4; IR (neat) 2937, 1667, 1615, 1566, 1443, 1403, 1311, 1276, 1263, 1218, 1124, 1106, 957, 842, 804, 770, 731 cm^{-1}; UV–Vis (CH_3CN) λ_{max} nm (ε) = 371 (3116), 251 (16101), 210 (30237); HRMS (ESI-TOF) calcd for $C_{20}H_{15}BrNaO_3$ ($[M+Na]^+$) *m/z* 405.0097, found *m/z* 405.0093.

11g: R_f 0.63 (hexane/EtOAc = 2/1); mp 191–192 °C; ^1H NMR (600 MHz, $CDCl_3$) δ 1.88–2.01 (m, 2H), 2.05–2.15 (m, 2H), 2.87–3.00 (m, 2H), 3.97 (s, 3H), 6.77 (d, 1H, *J* = 8.2 Hz), 6.87 (d, 1H, *J* = 8.2 Hz), 7.22 (dd, 1H, *J* = 7.3, 1.5 Hz), 7.34 (td, 1H, *J* = 7.3, 1.5 Hz), 7.37 (td, 1H, *J* = 7.3, 1.6 Hz), 7.56 (d, 1H, *J* = 9.0 Hz), 7.70 (d, 1H, *J* = 9.0 Hz), 7.85 (dd, 1H, *J* = 7.3, 1.6 Hz); ^{13}C NMR (150 MHz, $CDCl_3$) δ 19.6, 29.2, 30.9, 55.8, 101.5, 104.3, 105.3, 109.8, 114.9, 116.4, 124.6, 126.6, 127.6, 128.6, 129.4, 130.3, 134.9, 138.1, 140.8, 145.3, 150.2; IR (ATR) 2959, 2931, 2831, 1607, 1586, 1499, 1454, 1416, 1365, 1318, 1254, 1233, 1125, 1107, 1060, 1009, 925, 909, 853, 820, 804, 765, 756, 734, 668, 608 cm^{-1}; HRMS (ESI-TOF) calcd for $C_{21}H_{18}BrO_3$ ($[M+H]^+$) *m/z* 397.0434, found *m/z* 397.0425.

acetal **11h**

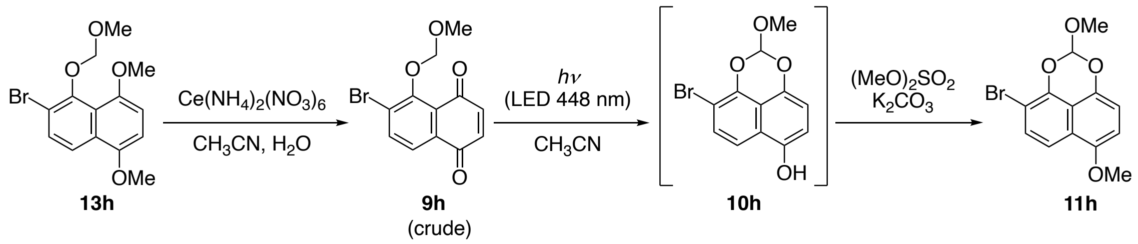

According to the general procedure, bromonaphthalene **13h** (27.6 mg, 0.0935 mmol) in CH_3CN (2 mL) was treated with ammonium cerium(IV) nitrate (109 mg, 0.199 mmol) in water (0.7 mL). Obtained naphthoquinone **9h** in CH_3CN (9.4 mL) was irradiated for 3.5 h and then added dimethyl sulfate (45.0 μL, 0.476 mmol) and K_2CO_3 (129 mg, 0.935 mmol). After stirring for 14.5 h, to the reaction was added Et_2NH (ca. 0.5 mL). After workup, the residue was purified by PTLC (silica gel, CH_2Cl_2 only) to afford **11h** (6.7 mg, 25%) as a white solid.

Naphthoquinone 9h: R_f 0.49 (hexane/EtOAc = 2/1); ^1H NMR (600 MHz, acetone-d_6) δ 3.63 (s, 3H), 5.16 (s, 2H), 6.95 (d, 1H, *J* = 10.3 Hz), 6.99 (d, 1H, *J* = 10.3 Hz), 7.76 (d, 1H, *J* = 8.3 Hz), 8.11 (d, 1H, *J* = 8.3 Hz); ^{13}C NMR (150 MHz, acetone-d_6) δ 57.8, 102.0, 123.7, 125.1, 126.5, 133.9, 137.0, 138.6, 140.8, 154.6, 183.8, 184.3; IR (ATR) 3054, 2957, 2835, 1668, 1655, 1611, 1565, 1449, 1393, 1369, 1317, 1288, 1272, 1211, 1169, 1117, 1081, 953, 930, 884, 860, 837, 808,

793, 761, 728, 618 cm^{-1}; UV–Vis (CH_3CN) λ_{max} nm (ε) = 366 (3782), 251 (16685), 209 (26654); HRMS (ESI-TOF) calcd for $C_{12}H_9BrNaO_4$ ($[M+Na]^+$) *m/z* 318.9576, found *m/z* 318.9568.

11h: R_f 0.54 (hexane/EtOAc = 2/1); ^1H NMR (600 MHz, acetone-d_6) δ 3.54 (s, 3H), 3.97 (s, 3H), 6.31 (s, 1H), 6.93 (d, 1H, *J* = 8.3 Hz), 6.95 (d, 1H, *J* = 8.3 Hz), 7.60 (d, 1H, *J* = 9.1 Hz), 7.69 (d, 1H, *J* = 9.1 Hz); ^{13}C NMR (150 MHz, acetone-d_6) δ 52.9, 55.7, 103.4, 106.1, 109.0, 110.1, 115.7, 117.3, 124.9, 130.4, 139.5, 144.1, 150.8; IR (neat) 3002, 2956, 2839, 1633, 1511, 1586, 1501, 1463, 1444, 1417, 1367, 1340, 1244, 1172, 1108, 1083, 1057, 1015, 952, 812, 797, 769, 716 cm^{-1}; HRMS (ESI-TOF) calcd for $C_{13}H_{12}BrO_4$ ($[M+H]^+$) *m/z* 310.9914, found *m/z* 310.9921.

acetal **11i**

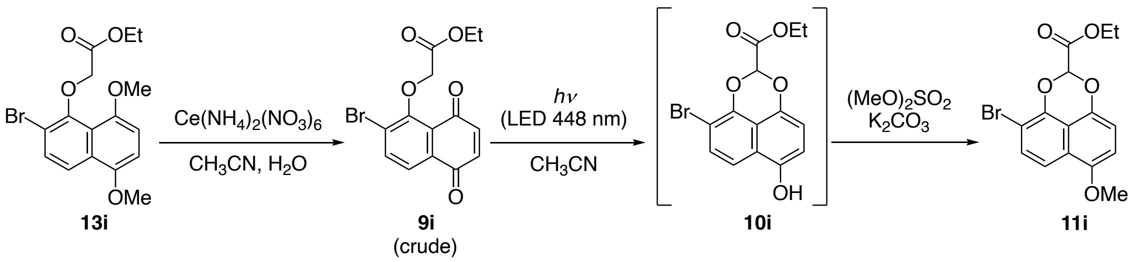

According to the general procedure, bromonaphthalene **13i** (26.0 mg, 0.0704 mmol) in CH_3CN (2 mL) was treated with ammonium cerium(IV) nitrate (81.1 mg, 0.148 mmol) in water (0.7 mL). Obtained naphthoquinone **9i** in CH_3CN (7.0 mL) was irradiated for 1.5 h and then added dimethyl sulfate (33.0 μL, 0.348 mmol) and K_2CO_3 (97.3 mg, 0.704 mmol). After stirring for 18 h, to the reaction was added Et_2NH (ca. 0.5 mL). After workup, the residue was purified by PTLC (silica gel, CH_2Cl_2 only) to afford **11i** (6.6 mg, 27%) as a colorless oil.

Naphthoquinone 9i: R_f 0.37 (hexane/EtOAc = 2/1); ^1H NMR (600 MHz, $CDCl_3$) δ 1.35 (t, 3H, *J* = 7.1 Hz), 4.35 (q, 2H, *J* = 7.1 Hz), 4.66 (s, 2H), 6.90 (d, 1H, *J* = 10.3 Hz), 6.93 (d, 1H, *J* = 10.3 Hz), 7.82 (d, 1H, *J* = 8.3 Hz), 7.97 (d, 1H, *J* = 8.3 Hz); ^{13}C NMR (150 MHz, $CDCl_3$) δ 14.2, 61.4, 69.8, 124.5, 125.4, 127.3, 132.8, 137.0, 138.6, 140.0, 154.2, 168.0, 183.4, 183.69; IR (ATR) 3072, 2987, 1747, 1661, 1611, 1566, 1450, 1412, 1383, 1319, 1276, 1248, 1210, 1129, 1107, 1070, 1049, 841, 802, 747 cm^{-1}; UV–Vis (CH_3CN) λ_{max} nm (ε) = 354 (3756), 251 (18847), 206 (27810); HRMS (ESI-TOF) calcd for $C_{14}H_{11}BrNaO_5$ ($[M+Na]^+$) *m/z* 360.9682, found *m/z* 360.9675.

11i: R_f 0.48 (hexane/EtOAc = 2/1); ^1H NMR (600 MHz, $CDCl_3$) δ 1.30 (t, 3H, *J* = 7.1 Hz), 3.96 (s, 3H), 4.26–4.36 (m, 2H), 5.85 (s, 1H), 6.76 (d, 1H, *J* = 8.3 Hz), 6.97 (d, 1H, *J* = 8.3 Hz), 7.55 (d, 1H, *J* = 9.0 Hz), 7.72 (d, 1H, *J* = 9.0 Hz); ^{13}C NMR (150 MHz, $CDCl_3$) δ 14.0, 55.8, 62.6, 93.6, 103.8, 105.0, 109.7, 115.4, 117.5, 124.9, 130.1, 140.5, 144.9, 150.8, 164.6; IR (neat) 2982,

2936, 2840, 1756, 1610, 1585, 1499, 1461, 1416, 1366, 1245, 1220, 1094, 1056, 1033, 810 cm^{-1}; HRMS (ESI-TOF) calcd for $C_{15}H_{13}BrNaO_5$ ($[M+Na]^+$) *m/z* 374.9839, found *m/z* 374.9838.

naphthoquinone **9j**

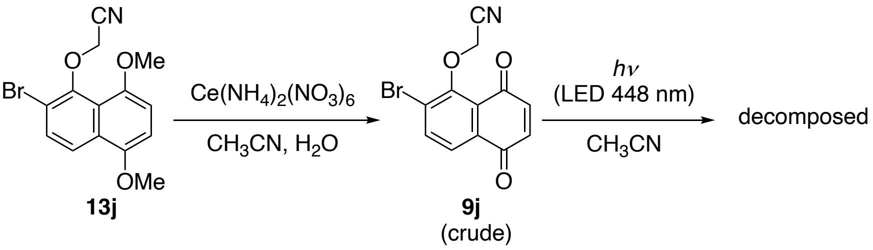

According to the general procedure, bromonaphthalene **13j** (17.5 mg, 0.0543 mmol) in CH_3CN (2 mL) was treated with ammonium cerium(IV) nitrate (62.5 mg, 0.114 mmol) in water (0.3 mL). Obtained naphthoquinone **9j** in CH_3CN (8.5 mL) was irradiated for 45 min and then added dimethyl sulfate (26.0 μL, 0.272 mmol) and K_2CO_3 (75.0 mg, 0.543 mmol). After stirring for 22 h, to the reaction was added Et_2NH (ca. 0.5 mL). After workup, the residue was obtained as an intractable mixture.

Naphthoquinone 9j: R_f 0.48 (hexane/EtOAc = 2/1); ^{1}H NMR (600 MHz, $CDCl_3$) δ 4.92 (s, 2H), 6.94 (d, 1H, *J* = 10.3 Hz), 6.99 (d, 1H, *J* = 10.3 Hz), 7.90 (d, 1H, *J* = 8.3 Hz), 8.03 (d, 1H, *J* = 8.3 Hz); ^{13}C NMR (150 MHz, $CDCl_3$) δ 58.1, 114.7, 125.48, 125.54, 127.31, 132.9, 137.3, 134.0, 139.9, 152.8, 183.4, 183.5; IR (ATR) 3068, 2999, 1662, 1650, 1606, 1564, 1446, 1410, 1357, 1321, 1293, 1282, 1261, 1226, 1170, 1129, 1111, 1015, 917, 899, 869, 842, 803, 763, 633, 620 cm^{-1}; UV–Vis (CH_3CN) λ_{max} nm (ε) = 350 (3728), 251 (20508), 205 (28446); HRMS (ESI-TOF) calcd for $C_{12}H_7BrNO_3$ ($[M+H]^+$) *m/z* 291.9604, found *m/z* 291.9593.

acetal **11k**

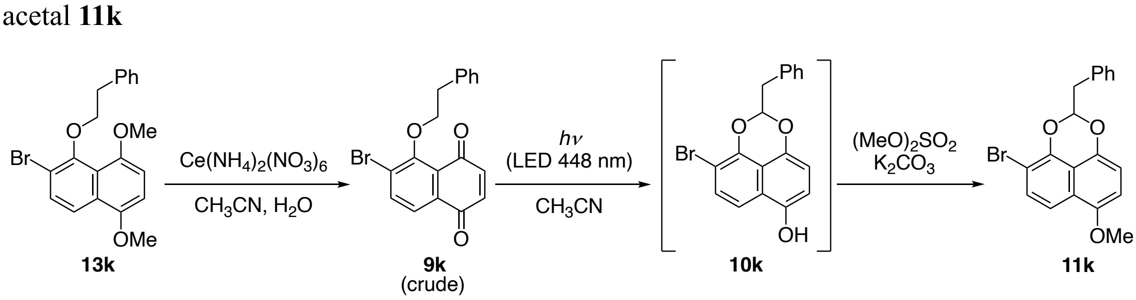

According to the general procedure, bromonaphthalene **13k** (26.0 mg, 0.0671 mmol) in CH_3CN (2 mL) was treated with ammonium cerium(IV) nitrate (80.0 mg, 0.146 mmol) in water (0.7 mL).

Obtained naphthoquinone **9k** in CH_3CN (6.7 mL) was irradiated for 30 min and then added dimethyl sulfate (32.0 μL, 0.338 mmol) and K_2CO_3 (94.0 mg, 0.680 mmol). After stirring for 16 h, to the reaction was added Et_2NH (ca. 0.5 mL). After workup, the residue was purified by PTLC (silica gel, CH_2Cl_2 only) to afford **11k** (16.7 mg, 67%) as a colorless oil.

Naphthoquinone 9k: R_f 0.50 (hexane/EtOAc = 2/1); ^1H NMR (600 MHz, $CDCl_3$) δ 3.33 (t, 2H, J = 7.5 Hz), 4.21 (t, 2H, J = 7.5 Hz), 6.89 (d, 1H, J = 10.3 Hz), 6.92 (d, 1H, J = 10.3 Hz), 7.23 (t, 1H, J = 7.2 Hz), 7.31 (dd, 2H, J = 7.3, 7.2 Hz), 7.35 (d, 2H, J = 7.3 Hz), 7.77 (d, 1H, J = 8.3 Hz), 7.95 (d, 1H, J = 8.3 Hz); ^{13}C NMR (150 MHz, $CDCl_3$) δ 36.4, 75.0, 123.8, 125.4, 126.5, 127.7, 128.4, 129.2, 133.0, 136.8, 137.7, 138.5, 140.2, 155.7, 183.5, 184.2; IR (ATR) 3058, 2942, 2888, 1665, 1614, 1566, 1441, 1413, 1362, 1307, 1282, 1220, 1120, 1106, 993, 925, 839, 804, 754, 732, 655 cm^{-1}; UV–Vis (CH_3CN) λ_{max} nm (ε) = 360 (4951), 250 (23940), 210 (34642); HRMS (ESI-TOF) calcd for $C_{18}H_{13}BrNaO_3$ ($[M+Na]^+$) *m/z* 378.9940, found *m/z* 378.9941.

11k: R_f 0.58 (hexane/EtOAc = 2/1); ^1H NMR (600 MHz, $CDCl_3$) δ 3.39 (dd, 1H, J = 14.3, 5.0 Hz), 3.47 (dd, 1H, J = 14.3, 5.3 Hz), 3.94 (s, 3H), 5.44 (dd, 1H, J = 5.3, 5.0 Hz), 6.72 (d, 1H, J = 8.3 Hz), 6.83 (d, 1H, J = 8.3 Hz), 7.29 (br-t, 1H, J = 7.4 Hz), 7.37 (br-dd, 2H, J = 7.7, 7.4 Hz), 7.48 (br-d, 2H, J = 7.7 Hz), 7.51 (d, 1H, J = 9.0 Hz), 7.66 (d, 1H, J = 9.0 Hz); ^{13}C NMR (150 MHz, $CDCl_3$) δ 40.5, 55.7, 100.8, 103.0, 105.0, 108.7, 115.9, 116.7, 125.1, 127.0, 128.4, 129.9, 130.2, 134.7, 142.7, 146.8, 150.3; IR (neat) 3029, 2932, 2832, 1632, 1609, 1584, 1499, 1460, 1444, 1414, 1371, 1357, 1257, 1244, 1115, 1048, 983, 922, 809, 738, 700 cm^{-1}; HRMS (ESI-TOF) calcd for $C_{19}H_{16}BrO_3$ ($[M+H]^+$) *m/z* 371.0277, found *m/z* 371.0267.

acetal **11l**

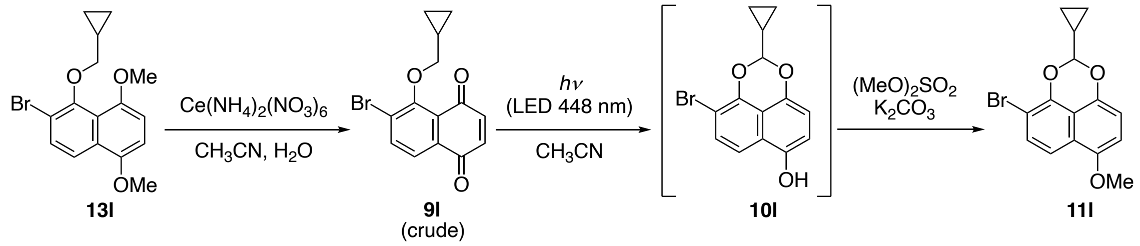

According to the general procedure, bromonaphthalene **13l** (24.8 mg, 0.0735 mmol) in CH_3CN (1.5 mL) was treated with ammonium cerium(IV) nitrate (84.4 mg, 0.154 mmol) in water (0.5 mL). Obtained naphthoquinone **9l** in CH_3CN (7.4 mL) was irradiated for 30 min and then added dimethyl sulfate (35.0 μL, 0.370 mmol) and K_2CO_3 (102 mg, 0.735 mmol). After stirring for 16 h, to the reaction was added Et_2NH (ca. 0.5 mL). After workup, the residue was purified twice by

PTLC (silica gel, 1st; hexane/EtOAc = 2/1, 2nd; CH_2Cl_2 only) to afford **11l** (16.4 mg, 69%) as a pale-yellow solid.

Naphthoquinone 9l: R_f 0.44 (hexane/EtOAc = 2/1); ^{1}H NMR (600 MHz, $CDCl_3$) δ 0.43–0.50 (m, 2H), 0.61–0.71 (m, 2H), 1.47–1.56 (m, 1H), 3.87 (d, 2H, J = 7.3 Hz), 6.87 (d, 1H, J = 10.3 Hz), 6.92 (d, 1H, J = 10.3 Hz), 7.77 (d, 1H, J = 8.3 Hz), 7.95 (d, 1H, J = 8.3 Hz); ^{13}C NMR (150 MHz, $CDCl_3$) δ 3.4, 10.8, 79.5, 123.7, 125.4, 128.0, 133.0, 136.8, 138.4, 140.2, 155.6, 183.5, 184.3; IR (ATR) 3066, 3004, 2949, 1660, 1614, 1567, 1448, 1395, 1314, 1285, 1260, 1212, 1164, 1101, 1025, 989, 865, 842, 816, 799, 731, 653 cm^{-1}; UV–Vis (CH_3CN) λ_{max} nm (ε) = 361 (3126), 250 (14581), 207 (22987); HRMS (ESI-TOF) calcd for $C_{14}H_{11}BrNaO_3$ ([M+Na]$^+$) m/z 328.9784, found m/z 328.9772.

11l: R_f 0.58 (hexane/EtOAc = 2/1); mp 94–96 °C; ^{1}H NMR (600 MHz, $CDCl_3$) δ 0.75–0.85 (m, 4H), 1.55–1.64 (m, 1H), 3.95 (s, 3H), 4.98 (d, 1H, J = 5.4 Hz), 6.73 (d, 1H, J = 8.3 Hz), 6.85 (d, 1H, J = 8.3 Hz), 7.52 (d, 1H, J = 9.0 Hz), 7.66 (d, 1H, J = 9.0 Hz); ^{13}C NMR (150 MHz, $CDCl_3$) δ 1.6, 1.7, 13.8, 55.7, 102.3, 102.9, 105.0, 108.5, 115.9, 116.6, 125.1, 130.0, 143.0, 147.1, 150.2; IR (neat) 3088, 3010, 2935, 2835, 1631, 1608, 1585, 1500, 1460, 1415, 1367, 1256, 1243, 1211, 1127, 1072, 971, 952, 927, 807, 741, 708 cm^{-1}; HRMS (ESI-TOF) calcd for $C_{15}H_{14}BrO_3$ ([M+H]$^+$) m/z 321.0121, found m/z 321.0115.

Modified synthesis of tetralone (R)-**18**[4]

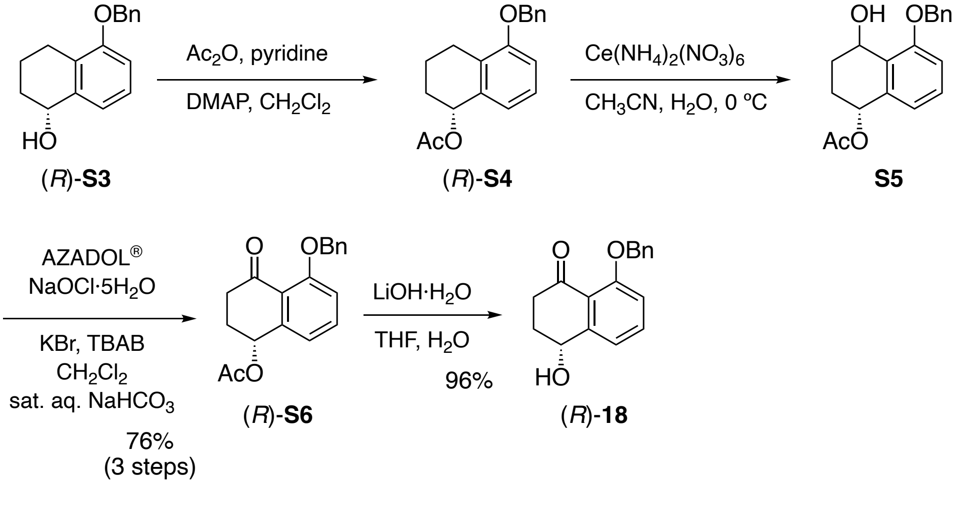

To a solution of alcohol (R)-**S3**[4] (5.00 g, 19.7 mmol) in CH_2Cl_2 (40 mL) was added pyridine (3.8

[4] Y. Ando, A. Hanaki, R. Sasaki, K. Ohmori, K. Suzuki, *Angew. Chem. Int. Ed.* **2017**, *68*, 11460–11465.

mL, 47.0 mmol), Ac_2O (2.20 mL, 23.3 mmol), and DMAP (120 mg, 0.985 mmol) at 0 °C. After stirring for 2.5 h at room temperature, the reaction was poured into mixed solvent of 1 M aqueous HCl and EtOAc. The mixture was extracted with EtOAc (×3), and the combined organic extracts were washed with 1 M aqueous HCl, water, saturated aqueous $NaHCO_3$, and brine, dried (Na_2SO_4), and concentrated in vacuo. This crude material was employed in the next experiment without further purification.

To a solution of this crude material, including (*R*)-**S4**, in CH_3CN (100 mL) was added $Ce(NH_4)_2(NO_3)_6$ (37.7 g, 68.9 mmol) in H_2O (50 mL) at 0 °C. After stirring for 40 min at 0 °C, the reaction was poured into mixed solvent of saturated aqueous $NaHCO_3$, 10% aqueous $Na_2S_2O_3$, and EtOAc at 0 °C. The mixture was extracted with EtOAc (×3), and the combined organic extracts were washed with brine, dried (Na_2SO_4), and concentrated in vacuo. This crude material was employed in the next experiment without further purification.

To a solution of this crude material, including **S5**, in CH_2Cl_2 (60 mL) was added TBAB (318 mg, 0.985 mmol), KBr (117 mg, 0.985 mmol), and AZADOL®[5] (151 mg, 0.985 mmol) at room temperature. To the stirred solution was added saturated aqueous $NaHCO_3$ (30 mL) and $NaOCl \cdot 5H_2O$ (4.87 g, 29.6 mmol) at 0 °C. After stirring for 30 min at room temperature, the reaction was poured into mixed solvent of saturated aqueous $NaHCO_3$, 10% aqueous $Na_2S_2O_3$, and EtOAc. The mixture was extracted with EtOAc (×3), and the combined organic extracts were washed with brine, dried (Na_2SO_4), and concentrated in vacuo. The residue was purified by MPLC (High-flash 3L column, hexane/EtOAc = 5/1 → 3/2) to afford tetralone (*R*)-**S6** with some impurity. Recrystallization from hexane and EtOAc (10/1) afforded (*R*)-**S6** (1st crop: 4.14 g, 68%; 2nd crop: 496 mg, 8%; total yield = 76% from (*R*)-**S3**) as colorless needles.

All data was identified with those reported.[4]

To a solution of tetralone (*R*)-**S6** (2.93 g, 9.44 mmol) in THF (48 mL) was added $LiOH \cdot H_2O$ (793 mg, 18.9 mmol) in H_2O (10 mL) at room temperature. After stirring for 4.6 h at 50 °C, the reaction was poured into water. The mixture was extracted with EtOAc (×3), and the combined organic extracts were washed with brine, dried over Na_2SO_4, and concentrated in vacuo. Recrystallization from hexane and EtOAc (5/2) to afforded (*R*)-**18** (1st crop: 2.05 g, 81%; 2nd crop: 269 mg, 11%; 3rd crop: 102 mg, 4%; total yield = 96%) as a pale pink solid.

---

[5] M. Shibuya, M. Tomizawa, I. Suzuki, Y. Iwabuchi, *J. Am. Chem. Soc.* **2006**, *128*, 8412–8413.

The enantiomeric excess of the alcohol was assessed by HPLC analysis with a chiral stationary phase [DAICEL CHIRALPAK® IF column (0.46 cm ϕ × 25 cm), eluent: hexane/*i*-PrOH = 80/20, 1 mL/min, temperature: 25 °C, detector: UV 254 nm, retention time: 11.6 min for the (*R*)-isomer, 18.1 min for the (*S*)-isomer].

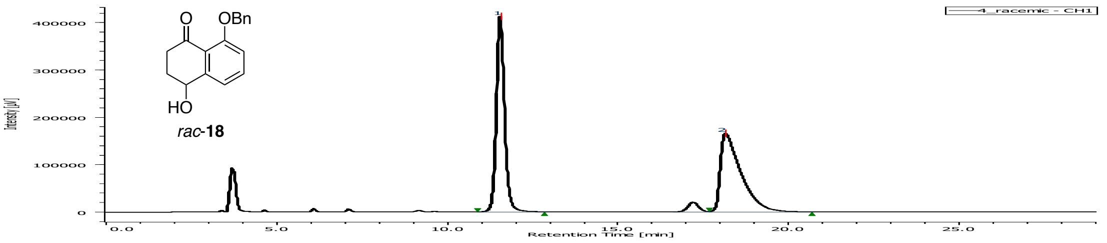

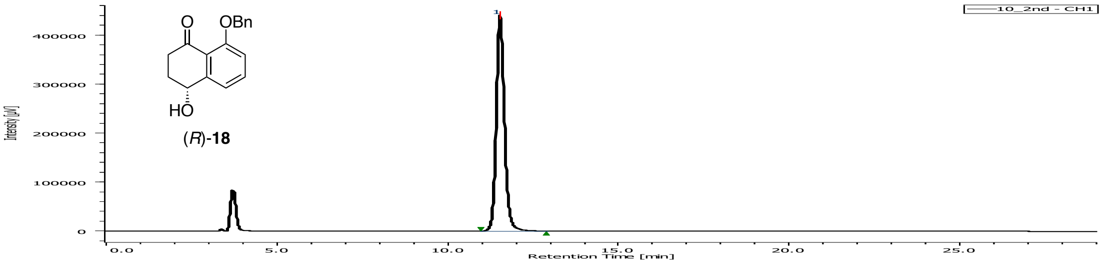

tetralone (*S*)-**20**

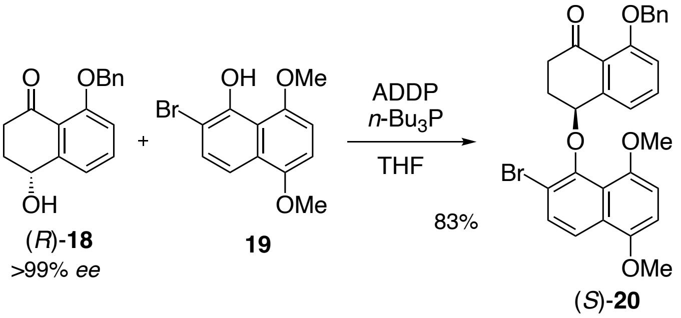

To a solution of tetralone (*R*)-**18** (995 mg, 3.71 mmol) and bromonaphthol **19** (1.00 g, 3.53 mmol) in THF (14 mL) was added 1,1'-(azodicarbonyl)dipiperidine (1.34 g, 5.30 mmol) and tributylphosphine (1.40 mL, 5.67 mmol) at 0 °C. After stirring for 3.5 h at room temperature, the reaction was poured into mixed solvent of 1 M aqueous HCl and EtOAc at 0 °C. The mixture was extracted with EtOAc (×3), and the combined organic extracts were washed with water, saturated aqueous $NaHCO_3$, and brine, dried (Na_2SO_4), and concentrated in vacuo. The residue was purified by flash column

chromatography (silica gel, hexane/EtOAc = 7/3) to afford (*S*)-**20** with some impurity. Recrystallization from CH_3CN afforded (*S*)-**20** (1st crop: 1.07 g, 57%; 2nd crop: 391 mg, 21%) as an olive green solid. The mother liquor was concentrated in vacuo, and the residue was purified by flash column chromatography (silica gel, hexane/EtOAc = 7/3). The obtained material was further purified by recrystallization from CH_3CN to afford (*S*)-**20** (3rd crop: 86.1 mg, 5%; total yield = 83%, >99% *ee*) as an olive green solid.

Note: After the recrystallization, the mother liquor was concentrated in vacuo, and the residue was purified by MPLC (High-flash L column, CH_2Cl_2) to afford (*S*)-**20** (116 mg, 6%, 86% *ee*) as an olive green solid. It showed that the racemization slightly proceeded in the Mitsunobu reaction (less than 1% *ee*). This product with lower enantiomeric excess was not used for the next reaction.

For the preparative scale (3 g) synthesis

To a solution of tetralone (*R*)-**18** (3.13 g, 11.7 mmol) and bromonaphthol **19** (3.00 g, 10.6 mmol) in THF (43 mL) was added 1,1'-(azodicarbonyl)dipiperidine (4.01 g, 15.9 mmol) and tributylphosphine (4.00 mL, 16.2 mmol) at 0 °C. After stirring for 3.3 h at room temperature, the reaction was poured into mixed solvent of 1 M aqueous HCl and EtOAc at 0 °C. The mixture was extracted with EtOAc (×3), and the combined organic extracts were washed with water, saturated aqueous $NaHCO_3$, and brine, dried (Na_2SO_4), and concentrated in vacuo. The residue was purified by flash column chromatography (silica gel, hexane/EtOAc = 7/3) to afford (*S*)-**20** with some impurity. Recrystallization from CH_3CN afforded (*S*)-**20** (1st crop: 3.48 g, 62%; 2nd crop: 978 mg, 17%; total yield = 79%, >99% *ee*) as an olive green solid.

Note: As the same above, the mother liquor was concentrated in vacuo, and the residue was purified by MPLC (High-flash 2L column, CH_2Cl_2) to afford (*S*)-**20** (313 mg, 6%, 91% *ee*) as an olive green solid. This product with lower enantiomeric excess was not used for the next reaction.

(*S*)-**20**: R_f 0.42 (toluene/EtOAc = 9/1); mp 113–119 °C (CH_3CN); $[\alpha]_D^{20}$ –306 (*c* 0.985, $CHCl_3$); ^1H NMR (600 MHz, $CDCl_3$) δ 2.30–2.36 (m, 1H), 2.65 (dt, 1H, *J* = 17.3, 5.0 Hz), 2.71–2.76 (m, 1H), 3.33 (ddd, 1H, *J* = 17.3, 11.0, 5.0 Hz), 3.76 (s, 3H), 3.94 (s, 3H), 5.19 (d, 1H, *J* = 12.4 Hz), 5.23 (d, 1H, *J* = 12.4 Hz), 5.28 (dd, 1H, *J* = 5.1, 3.2 Hz), 6.30 (d, 1H, *J* = 7.4 Hz), 6.71 (s, 2H), 6.95 (d, 1H, *J* = 8.3 Hz), 7.12 (dd, 1H, *J* = 8.3, 7.4 Hz), 7.30 (t, 1H, *J* = 7.4 Hz), 7.39 (dd, 2H, *J* = 7.4, 7.3 Hz), 7.49 (d, 1H, *J* = 9.0 Hz), 7.58 (d, 2H, *J* = 7.4 Hz), 7.88 (d, 1H, *J* = 9.0 Hz); ^{13}C NMR (150 MHz, $CDCl_3$) δ 28.7, 35.9, 55.7, 56.4, 70.6, 80.1, 104.2, 106.7, 114.5, 117.8, 119.4, 121.3, 122.16, 122.23, 126.7, 127.6, 127.8, 128.5, 129.9, 133.3, 136.8, 143.1, 148.9, 149.1, 149.6, 158.8, 196.8; IR (ATR) 2939,

1673, 1614, 1594, 1575, 1448, 1405, 1307, 1277, 1260, 1246, 1089, 1047, 1035, 976, 804, 728, 696 cm^{-1}; HRMS (ESI-TOF) calcd for $C_{29}H_{25}BrNaO_5$ ($[M+Na]^+$) *m/z* 555.0778, found *m/z* 555.0758.

Crystallographic data: $C_{29}H_{25}BrO_5$, Formula Weight = 533.40, 0.300 ×0.300 ×0.250 mm, triclinic, space group *P*1, Z = 2, T = 293 K, a = 9.6743(3), b = 10.9125(3), c = 12.3660(3) Å, V = 1216.25(6) $Å^3$, λ (CuKα) = 1.54184 Å, μ = 2.622 mm^{-1} Intensity data were collected on Rigaku XtaLAB Synergy-R/DW. The structure was solved by direct methods and refined by the full-matrix least-squares on F^2 (SHELXL-2018/3). A total of 32460 reflections were measured and 9041 were independent. Final $R1$ = 0.0755, $wR2$ = 0.1913 (7987 refs; $I > 2s(I)$), and GOF = 1.021 (for all data, $R1$ = 0.0796, $wR2$ = 0.1999). Flack Parameter = –0.02 (2).

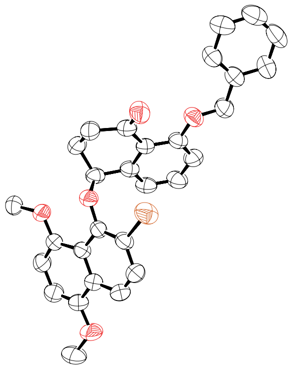

X-ray structure of (*S*)-**20**

(CCDC 2177099)

The enantiomeric excess of the tetralone (*S*)-**20** was assessed by HPLC analysis with a chiral stationary phase [DAICEL CHIRALPAK® IB column (0.46 cm ϕ × 25 cm), eluent: hexane/EtOAc = 85/15, 1 mL/min, temperature: 25 °C, detector: UV 254 nm, retention time: 19.8 min for the (*R*)-isomer, 21.6 min for the (*S*)-isomer].

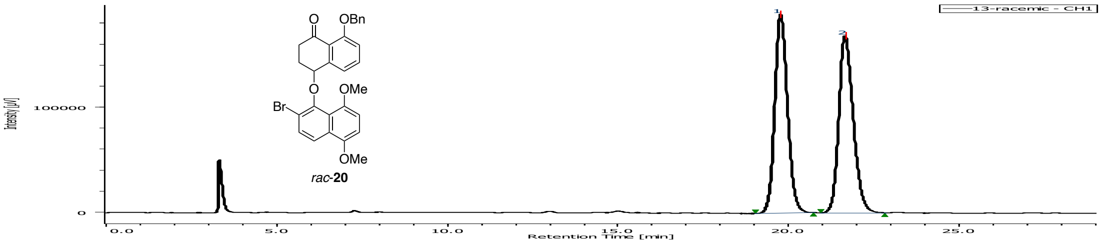

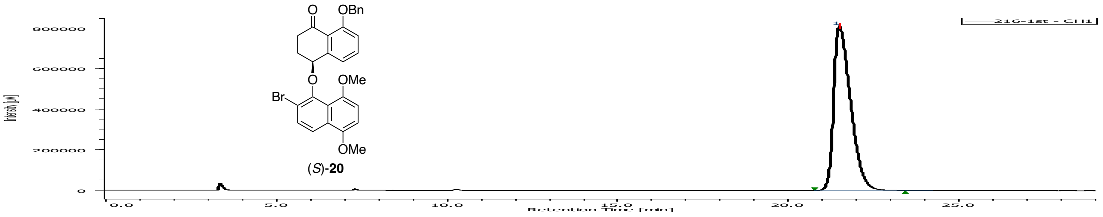

spiroacetal (*S*)-**22**

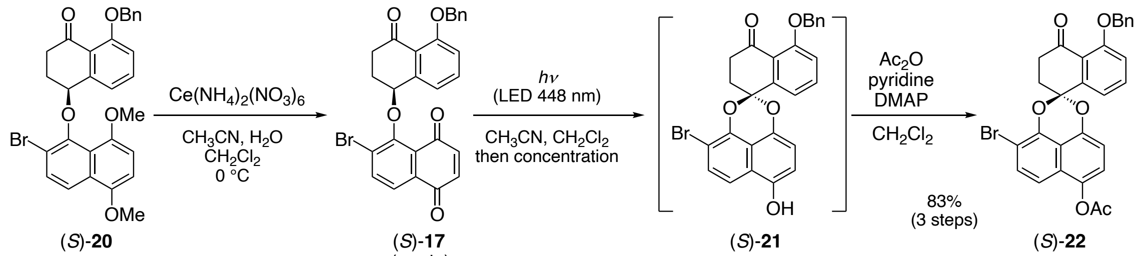

(*S*)-**20** >99% *ee* (*S*)-**17** (crude) (*S*)-**21** (*S*)-**22** >99% *ee*

To a solution of tetralone (*S*)-**20** (1.50 g, 2.81 mmol) in CH_3CN (56 mL) and CH_2Cl_2 (20 mL) in a brown round bottom flask was added ammonium cerium(IV) nitrate (3.40 g, 6.20 mmol) in H_2O (20 mL) dropwise at 0 °C. After stirring for 12 min at 0 °C, the reaction was quenched by adding saturated aqueous $NaHCO_3$ and poured into mixed solvent of brine and CH_2Cl_2. The mixture was extracted with CH_2Cl_2 using a brown separatory funnel, and aqueous phase was filtered through Celite® pad (washed with CH_2Cl_2). The mixture was extracted with CH_2Cl_2 (×2), and the combined organic extracts were washed with brine, dried (Na_2SO_4), and concentrated in vacuo using an aluminum foil as a cover for a normal flask. This crude material was used for the next experiment without further purification. A small portion was purified by PTLC (silica gel, toluene/acetone = 10/1) to give analytically pure sample.

A solution of this crude material, including (*S*)-**17** in CH_3CN (187 mL) and CH_2Cl_2 (94 mL) was placed in a five necked round bottom flask, and degassed three times by purging with argon under sonication. The solution was irradiated by using four tubes of LED light (Techno Sigma PER-AMP, PAR-448, 680 mW; see figure S2) for 1.5 h at room temperature. The reaction was concentrated in vacuo. To a solution of this crude material, including (*S*)-**21**, in CH_2Cl_2 (5.6 mL) was added pyridine (4.55 mL, 56.4 mmol), Ac_2O (2.65 mL, 28.0 mmol), and DMAP (34.2 mg, 0.280 mmol) at 0 °C. After stirring for 55 min at room temperature, the reaction was poured into mixed solvent of 1 M aqueous HCl and EtOAc. The mixture was extracted with EtOAc (×3), and the combined organic extracts were washed with 1 M aqueous HCl, water, saturated aqueous $NaHCO_3$, and brine, dried over Na_2SO_4, and concentrated in vacuo. The residue was purified by flash column chromatography (silica gel, toluene/acetone = 30/1) to afford spiroacetal (*S*)-**22** (1.28 g, 83% from (*S*)-**20**) as a sun yellow solid and dimeric spiroacetal **S7** (152 mg, impure). A small portion of impure **S7** was further purified by PTLC (silica gel, hexane/EtOAc = 1/2) to give analytical sample.

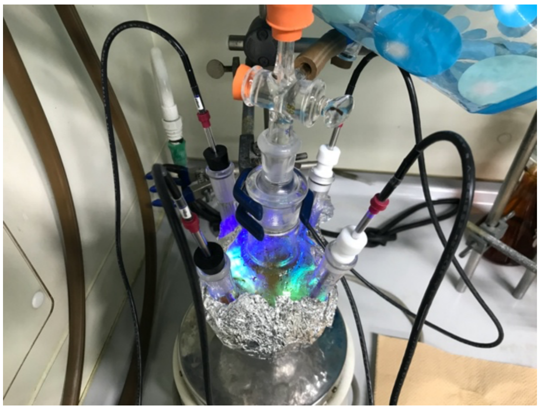 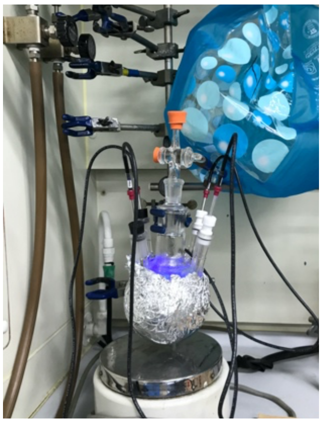

Figure S2 (top and side views)

Naphthoquinone (*S*)-17: R_f 0.32 (toluene/acetone = 10/1); $[\alpha]_D^{20}$ –442 (*c* 0.500, $CHCl_3$); ^{1}H NMR (600 MHz, $CDCl_3$) δ 2.35–2.41 (m, 1H), 2.72 (dt, 1H, *J* = 17.5, 4.7 Hz), 2.95 (ddd, 1H, *J* = 13.9, 9.5, 4.7 Hz), 3.37 (ddd, 1H, *J* = 17.5, 9.5, 5.2 Hz), 5.21 (d, 1H, *J* = 12.5 Hz), 5.25 (d, 1H, *J* = 12.5 Hz), 5.20–5.26 (m, 1H), 6.40 (d, 1H, *J* = 7.4 Hz), 6.82 (d, 1H, *J* = 10.2 Hz), 6.92 (d, 1H, *J* = 10.2 Hz), 7.02 (d, 1H, *J* = 8.2 Hz), 7.23 (dd, 1H, *J* = 8.2, 7.4 Hz), 7.30 (t, 1H, *J* = 7.4 Hz), 7.39 (dd, 2H, *J* = 7.4, 7.3 Hz), 7.57 (d, 2H, *J* = 7.3 Hz), 7.76 (d, 1H, *J* = 8.3 Hz), 7.85 (d, 1H, *J* = 8.3 Hz); ^{13}C NMR (150 MHz, $CDCl_3$) δ 28.7, 35.4, 70.6, 81.2, 115.3, 121.1, 122.4, 123.8, 125.4, 126.7, 127.7, 128.5, 128.9, 133.1, 133.5, 136.6, 136.7, 138.5, 140.2, 142.1, 153.7, 159.1, 183.4, 184.1, 196.3; IR (ATR) 3073, 1682, 1667, 1658, 1617, 1592, 1567, 1504, 1469 1448, 1437, 1405, 1310, 1278, 1265, 1248, 1218, 1101, 1010, 973, 841, 795, 740 cm^{-1}; UV–Vis (CH_3CN) λ_{max} nm (ε) = 354 (4046), 304 (6318), 239 (25320), 208 (36617); HRMS (ESI-TOF) calcd for $C_{27}H_{19}BrNaO_5$ ($[M+Na]^+$) *m/z* 525.0308, found *m/z* 525.0303.

(*S*)-**22**: R_f 0.52 (toluene/acetone = 10/1); mp 138–140 °C (CH_3CN/MeOH); $[\alpha]_D^{20}$ –41.8 (*c* 1.02, $CHCl_3$); ^{1}H NMR (600 MHz, $CDCl_3$) δ 2.43–2.52 (m, 2H), 2.45 (s, 3H), 2.77 (ddd, 1H, *J* = 17.4, 6.8, 5.8 Hz), 2.84 (ddd, 1H, *J* = 17.4, 8.4, 5.8 Hz), 5.26 (d, 1H, *J* = 12.9 Hz), 5.29 (d, 1H, *J* = 12.9 Hz), 7.00 (d, 1H, *J* = 8.0 Hz), 7.19 (dd, 1H, *J* = 8.3, 0.8 Hz), 7.23 (d, 1H, *J* = 8.0 Hz), 7.32 (t, 1H, *J* = 7.6 Hz), 7.36 (d, 1H, *J* = 9.0 Hz), 7.42 (dd, 2H, *J* = 7.8, 7.6 Hz), 7.57 (dd, 1H, *J* = 7.8, 0.8 Hz), 7.59–7.60 (m, 2H), 7.61–7.63 (m, 2H); ^{13}C NMR (150 MHz, $CDCl_3$) δ 20.9, 29.4, 35.4, 70.8, 100.2, 104.3, 110.1, 114.5, 115.7, 116.1, 118.1, 120.1, 121.5, 126.2, 126.7, 127.8, 128.6, 131.8, 134.8, 136.4, 140.9, 141.9, 144.5, 144.7, 158.6, 169.5, 194.5; IR (ATR) 1760, 1683, 1596, 1498, 1446, 1414, 1362, 1318, 1275, 1250, 1191, 1130, 1079, 1014, 983, 913, 801, 732, 696 cm^{-1}; HRMS (ESI-TOF) calcd for $C_{29}H_{22}BrO_6$ ($[M+H]^+$) *m/z* 545.0594, found *m/z* 545.0587.

The enantiomeric excess of the spiroacetal (*S*)-**22** was assessed by HPLC analysis with a chiral stationary phase [DAICEL CHIRALPAK® IB column (0.46 cm ϕ × 25 cm), eluent: hexane/EtOAc = 80/20, 1 mL/min, temperature: 25 °C, detector: UV 254 nm, retention time: 13.5 min for the (*S*)-isomer, 14.9 min for the (*R*)-isomer].

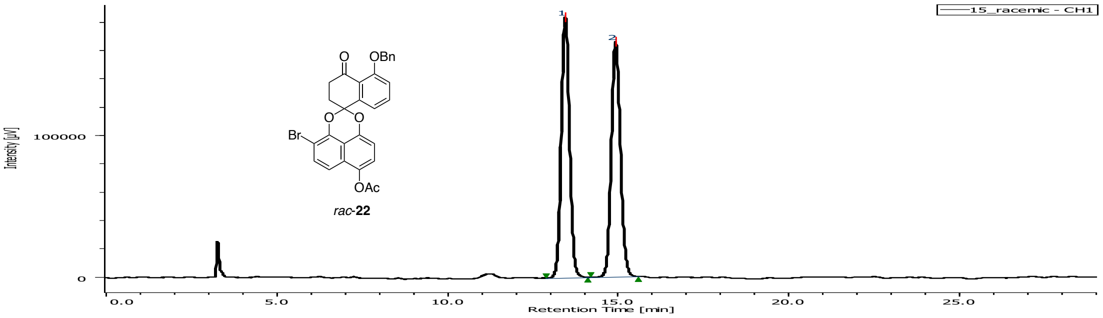

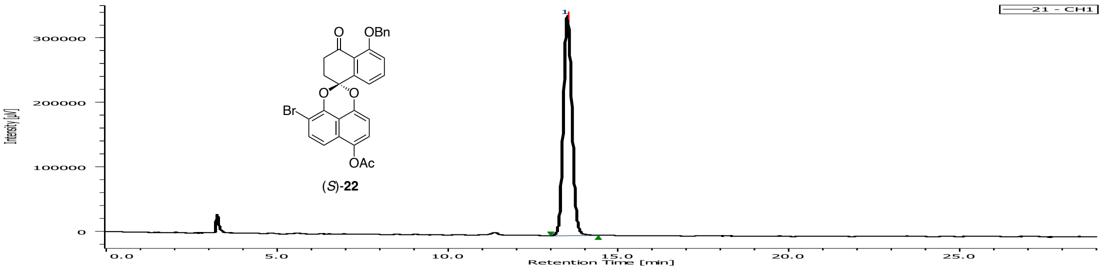

Crystallographic data: $C_{29}H_{21}BrO_6$, Formula Weight = 545.36, 0.05 ×0.21 ×0.35 mm, monoclinic, space group $P2_1$, Z = 4, T = 93 K, *a* = 17.6204(2), *b* = 5.3237(1), *c* = 25.4256(3) Å, V = 2358.97(6) Å^3, λ (CuKα) = 1.54184 Å, μ = 2.755 mm^{-1} Intensity data were collected on Rigaku XtaLAB Synergy-R/DW. The structure was solved by direct methods and refined by the full-matrix least-squares on F^2 (SHELXL-2018/1). A total of 8406 reflections were measured and 7790 were independent. Final *R*1 = 0.0354, *wR*2 = 0.0995 (6844 refs; *I* > 2s(*I*)), and GOF = 1.051 (for all data, *R*1 = 0.0409, *wR*2 = 0.1030). Flack Parameter = –0.011 (9).

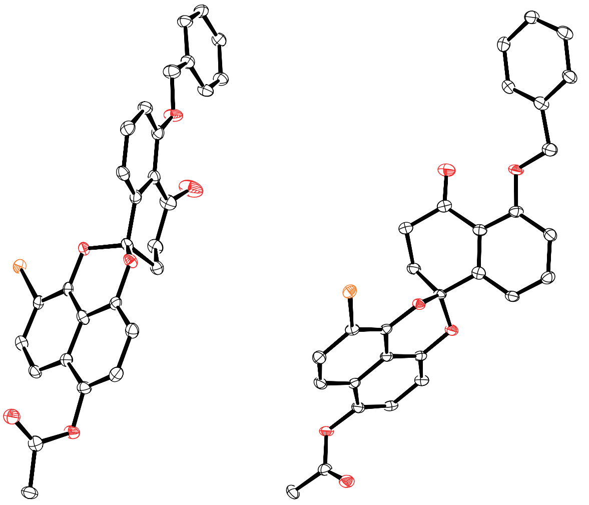

X-ray structure of (*S*)-**22** (front and side views)

(CCDC 2177100)

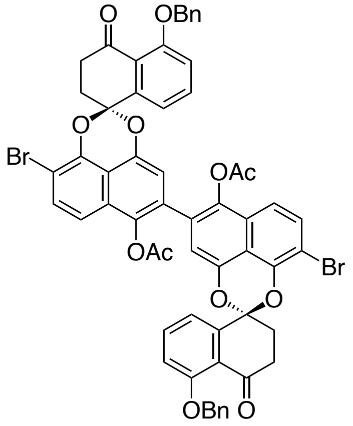

Dimeric spiroacetal S7: R_f 0.41 (toluene/acetone = 10/1); ^{1}H NMR (600 MHz, $CDCl_3$) δ 2.15 (s, 6H), 2.49–2.53 (m, 2H), 2.56–2.60 (m, 2H), 2.78 (dt, 2H, *J* = 17.3, 6.0 Hz), 2.86–2.89 (m, 2H), 5.27 (d, 2H, *J* = 12.5Hz), 5.29 (d, 2H, *J* = 12.5Hz), 7.02 (s, 2H), 7.20 (d, 2H, *J* = 8.3 Hz), 7.31 (d, 2H, *J* = 8.3 Hz), 7.32 (t, 2H, *J* = 7.5 Hz), 7.41 (dd, 4H, *J* = 7.8, 7.5 Hz), 7.56–7.63 (m, 8H), 7.67 (d, 2H, *J* = 9.0 Hz); ^{13}C NMR (150 MHz, $CDCl_3$) δ 20.5, 29.5, 35.3, 70.8, 100.4, 104.8, 112.1, 114.3, 115.8, 116.8, 118.0, 121.5, 126.5, 126.7, 127.8, 128.6, 128.8, 132.4, 134.8, 136.4, 138.5, 141.7, 144.4, 144.8, 158.7, 169.0, 194.3; IR (neat) 1768, 1688, 1605, 1385, 1367, 1317, 1192, 1041, 1014, 913, 733 cm^{-1}; HRMS (ESI-TOF) calcd for $C_{58}H_{41}Br_2O_{12}$ ([M+H]$^+$) *m/z* 1087.0959, found *m/z* 1087.0929.

key HMBC correlations

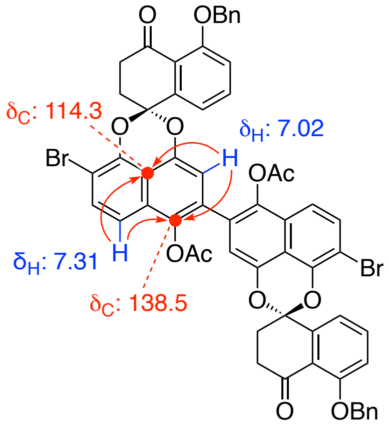

Synthesis of S8 for the photochemical reaction without a bromo substituent

tetralone **S8**

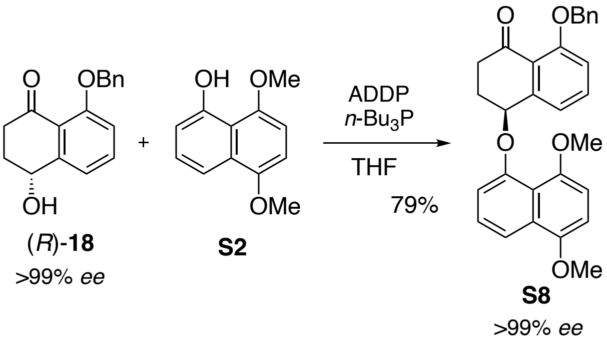

To a solution of tetralone (*R*)-**18** (144 mg, 0.537 mmol) and naphthol **S2** (101 mg, 0.495 mmol) in THF (2.0 mL) was added 1,1'-(azodicarbonyl)dipiperidine (185 mg, 0.733 mmol) and tributylphosphine (180 µL, 0.730 mmol) at 0 °C. After stirring for 3.5 h at room temperature, the reaction was poured into mixed solvent of 1 M aqueous HCl and EtOAc at 0 °C. The mixture was extracted with EtOAc (×3), and the combined organic extracts were washed with water, saturated aqueous $NaHCO_3$, and brine, dried (Na_2SO_4), and concentrated in vacuo. The residue was purified by flash column chromatography (silica gel, toluene/EtOAc = 33/1 → 20/1) to afford **S8** (177 mg, 79%, >99% *ee*) as an off-white solid.

S8: R_f 0.44 (toluene/EtOAc = 9/1); $[\alpha]_D^{20}$ –173 (*c* 1.06, $CHCl_3$); ^1H NMR (600 MHz, $CDCl_3$) δ 2.35–2.40 (m, 1H), 2.49–2.55 (m, 1H), 2.63 (dt, 1H, *J* = 17.8, 6.0 Hz), 3.14 (ddd, 1H, *J* = 17.8, 9.1, 5.4 Hz), 3.79 (s, 3H), 3.94 (s, 3H), 5.20 (d, 1H, *J* = 12.4 Hz), 5.24 (d, 1H, *J* = 12.4 Hz), 5.41 (dd, 1H, *J* = 6.3, 3.0 Hz), 6.71 (d, 1H, *J* = 8.6 Hz), 6.72 (d, 1H, *J* = 8.6 Hz), 6.92 (d, 1H, *J* = 7.6 Hz), 6.97 (d, 1H, *J* = 7.5 Hz), 7.00 (d, 1H, *J* = 8.3 Hz), 7.29–7.35 (m, 3H), 7.40 (dd, 2H, *J* = 7.8, 7.5 Hz), 7.59 (d, 2H, *J* =

7.5 Hz), 7.95 (d, 1H, J = 8.3 Hz); ^{13}C NMR (150 MHz, $CDCl_3$) δ 27.8, 35.7, 55.8, 56.6, 70.6, 77.3, 104.0, 105.9, 114.1, 116.0, 116.8, 120.2, 120.9, 121.8, 125.5, 126.7, 127.6, 128.5, 129.0, 133.7, 136.7, 144.6, 149.5, 150.3, 153.6, 158.8, 196.4; IR (ATR) 3063, 2995, 2962, 2934, 2887, 2854, 2834, 1684, 1621, 1596, 1584, 1515, 1499, 1451, 1409, 1381, 1264, 1174, 1074, 1044, 981, 910, 808, 752, 740, 724 cm^{-1}; HRMS (ESI-TOF) calcd for $C_{29}H_{26}NaO_5$ ([M+Na]$^+$) *m/z* 477.1673, found *m/z* 477.1670.

The enantiomeric excess of the tetralone **S8** was assessed by HPLC analysis with a chiral stationary phase [DAICEL CHIRALPAK® IB column (0.46 cm ϕ × 25 cm), eluent: hexane/EtOAc = 80/20, 1 mL/min, temperature: 25 °C, detector: UV 254 nm, retention time: 19.5 min for the (*R*)-isomer, 22.7 min for the (*S*)-isomer].

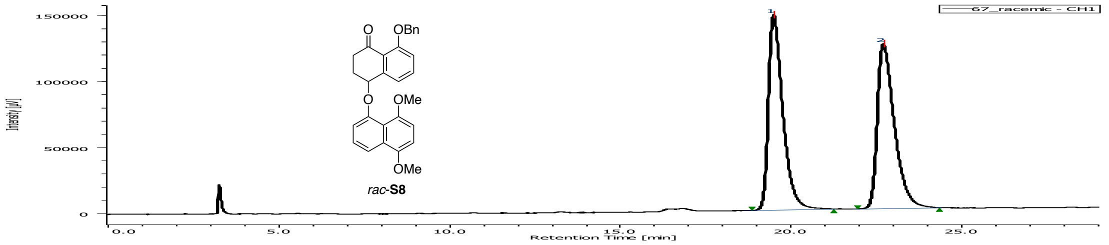

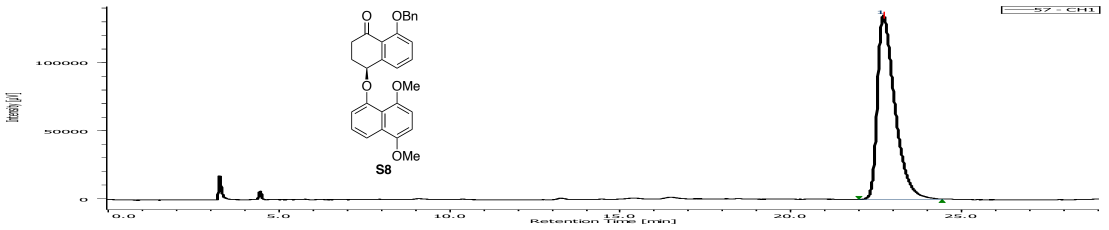

Photochemical reaction of naphthoquinone S8 without a bromo substituent

spiroacetal **S11**

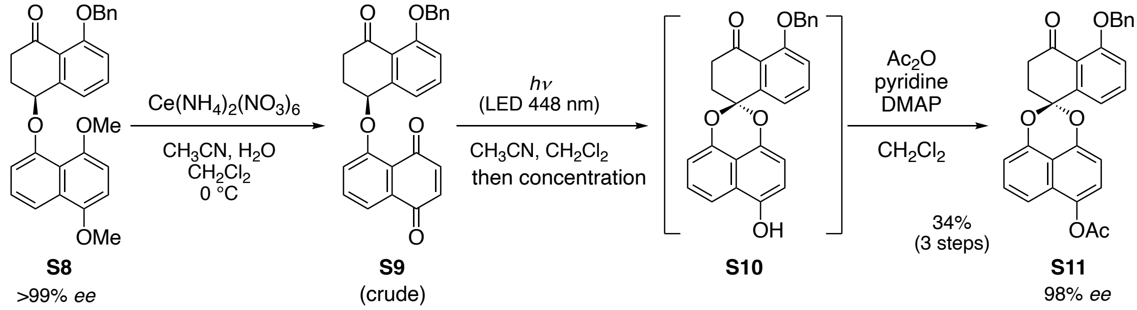

To a solution of tetralone **S8** (50.7 mg, 0.112 mmol) in CH_3CN (2.2 mL) and CH_2Cl_2 (0.75 mL) in a brown round bottom flask was added ammonium cerium(IV) nitrate (136 mg, 0.248 mmol) in H_2O (0.75 mL) dropwise at 0 °C. After stirring for 10 min at 0 °C, the reaction was quenched by adding

saturated aqueous $NaHCO_3$ and poured into mixed solvent of brine and CH_2Cl_2. The mixture was extracted with CH_2Cl_2 (×3), and the combined organic extracts were washed with brine, dried (Na_2SO_4), and concentrated in vacuo using a brown round bottom flask. This crude material was used for the next experiment without further purification. A small portion was purified by PTLC (silica gel, hexane/EtOAc = 1/2) to give analytically pure sample.

A solution of this crude material, including **S9**, in CH_3CN (7.4 mL) and CH_2Cl_2 (3.7 mL) was placed in a five necked round bottom flask, and degassed three times by purging with argon under sonication (×3). The solution was irradiated by using a tube of LED light (Techno Sigma PER-AMP, PAR-448, 680 mW; see figure S2) for 2.5 h at room temperature. The reaction was concentrated in vacuo. To a solution of this crude material, including **S10**, in CH_2Cl_2 (1.1 mL) was added pyridine (180 μL, 2.23 mmol), Ac_2O (105 μL, 1.11 mmol), and DMAP (1.4 mg, 0.0115 mmol) at 0 °C. After stirring for 2.0 h at room temperature, the reaction was poured into mixed solvent of 1 M aqueous HCl and EtOAc. The mixture was extracted with EtOAc (×3), and the combined organic extracts were washed with 1 M aqueous HCl, water, saturated aqueous $NaHCO_3$, and brine, dried over Na_2SO_4, and concentrated in vacuo. The residue was purified by PTLC (silica gel, toluene/EtOAc = 10/3) to afford spiroacetal **S11** (17.7 mg, 34% from **S8**) as a pale-yellow oil, dimeric spiroacetal **S12** (1.1 mg, ca. 2% from **S8**) as a pale pink solid and diacetate **S13** (8.6 mg, 15% from **S8**) as a pale-yellow oil.

Naphthoquinone S9: R_f 0.50 (toluene/EtOAc = 10/3); $[\alpha]_D^{20}$ –79.4 (*c* 0.432, $CHCl_3$); ^1H NMR (600 MHz, $CDCl_3$) δ 2.40–2.45 (m, 1H), 2.52–2.58 (m, 1H), 2.69 (ddd, 1H, *J* = 17.8, 7.0, 5.7 Hz), 3.17 (ddd, 1H, *J* = 17.8, 8.4, 5.5 Hz), 5.21 (d, 1H, *J* = 12.3 Hz), 5.24 (d, 1H, *J* = 12.3 Hz), 5.52 (dd, 1H, *J* = 7.0, 3.1 Hz), 6.84 (d, 1H, *J* = 10.3 Hz), 6.88 (d, 1H, *J* = 10.3 Hz), 7.05 (d, 1H, *J* = 8.2 Hz), 7.12 (d, 1H, *J* = 7.6 Hz), 7.299 (dd, 1H, *J* = 8.3, 0.9 Hz), 7.302 (t, 1H, *J* = 7.3 Hz), 7.39 (t, 2H, *J* = 7.3 Hz), 7.43 (dd, 1H, *J* = 8.2, 7.6 Hz), 7.58 (d, 2H, *J* = 7.3 Hz), 7.63 (dd, 1H, *J* = 8.3, 7.6 Hz), 7.80 (dd, 1H, *J* = 7.6, 0.9); ^{13}C NMR (150 MHz, $CDCl_3$) δ 28.0, 35.7, 70.6, 76.7, 114.5, 120.1, 120.9, 121.6, 121.9, 123.8, 126.6, 127.7, 128.5, 134.2, 134.3, 134.5, 136.3, 136.5, 140.7, 143.4, 157.2, 158.9, 183.9, 185.0, 195.5; IR (DR) 1660, 1612, 1593, 1584, 1462, 1451, 1328, 1293, 1273, 1248, 1107, 1093, 1041, 1022, 971, 851 749 cm^{-1}; UV–Vis (CH_3CN) λ_{max} nm (ε) = 386 (2615), 313 (6071), 246 (23092), 212 (34010); HRMS (ESI-TOF) calcd for $C_{27}H_{20}NaO_5$ ($[M+Na]^+$) *m/z* 447.1203, found *m/z* 447.1201.

S11: R_f 0.66 (toluene/EtOAc = 10/3); $[\alpha]_D^{20}$ +13.9 (*c* 1.01, $CHCl_3$); ^1H NMR (600 MHz, $CDCl_3$) δ 2.45 (s, 3H), 2.50 (t, 2H, *J* = 6.8 Hz), 2.78 (t, 2H, *J* = 6.8 Hz), 5.27 (s, 2H), 6.94 (d, 1H, *J* = 8.1 Hz), 7.00 (dd, 1H, *J* = 7.1, 1.1 Hz), 7.16 (dd, 1H, *J* = 8.2, 1.0 Hz), 7.21 (d, 1H, *J* = 8.1 Hz), 7.32 (t, 1H, *J* = 7.4 Hz), 7.41 (dd, 2H, *J* = 7.8, 7.4 Hz), 7.46 (dd, 1H, *J* = 8.5, 1.1 Hz), 7.49 (dd, 1H, *J* = 8.5, 7.1 Hz),

7.55 (dd, 1H, J = 7.7, 1.0 Hz), 7.58–7.61 (m, 3H); ^{13}C NMR (150 MHz, $CDCl_3$) δ 20.9, 29.4, 35.4, 70.8, 99.2, 108.9, 110.2, 113.9, 114.9, 115.6, 118.1, 119.7, 121.5, 126.7, 127.3, 127.8, 128.2, 128.6, 134.8, 136.5, 140.8, 142.5, 145.3, 147.8, 158.5, 169.6, 194.8; IR (neat) 3064, 1764, 1686, 1610, 1595, 1418, 1378, 1319, 1270, 1200, 1046, 1016, 985, 916, 734 cm^{-1}; HRMS (ESI-TOF) calcd for $C_{29}H_{23}O_6$ ([M+H]$^+$) *m/z* 467.1489, found *m/z* 467.1495.

The enantiomeric excess of the spiroacetal **S11** was assessed by HPLC analysis with a chiral stationary phase [DAICEL CHIRALPAK® IA column (0.46 cm ϕ × 25 cm), eluent: hexane/*i*-PrOH = 80/20, 1 mL/min, temperature: 25 °C, detector: UV 254 nm, retention time: 15.3 min for the (*S*)-isomer, 17.2 min for the (*R*)-isomer].

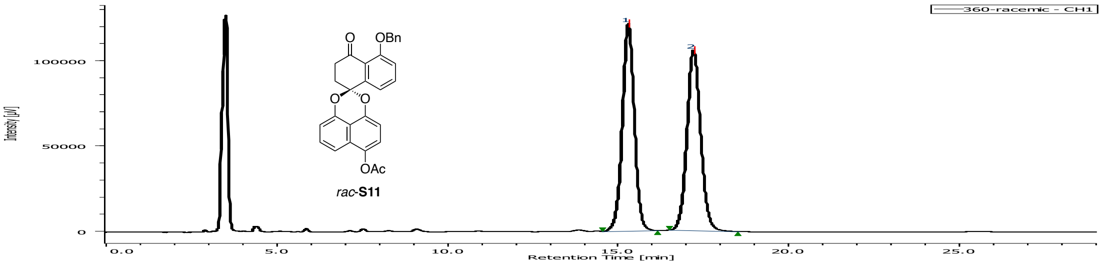

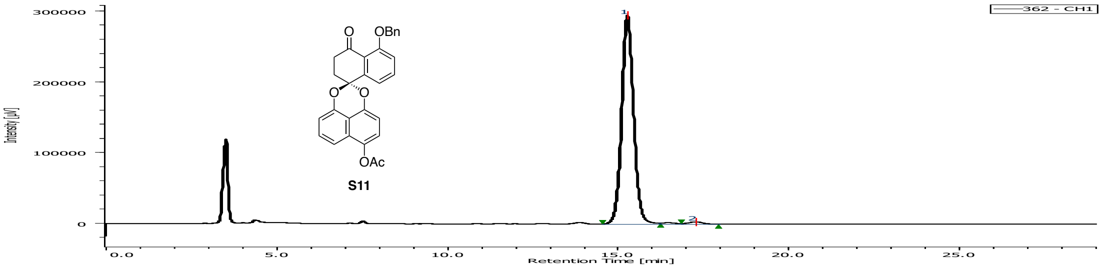

	tR /min	peak area	peak area /%
peak 1	15.267	7117620	98.748
peak 2	17.267	90258	1.252

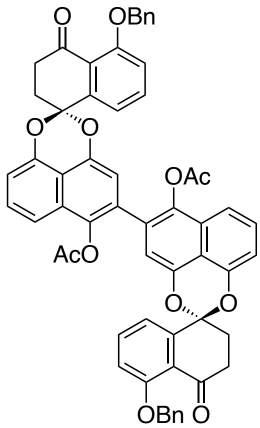

S12

Dimeric spiroacetal S12: R*f* 0.55 (toluene/EtOAc = 10/3); ^{1}H NMR (600 MHz, $CDCl_3$) δ 2.16 (s, 6H), 2.52–2.62 (m, 4H), 2.77–2.85 (m, 4H), 5.27 (s, 4H), 6.98 (s, 2H), 7.05 (dd, 2H, *J* = 7.6, 0.5 Hz), 7.18 (dd, 2H, *J* = 8.2, 1.0 Hz), 7.32 (t, 2H, *J* = 7.4 Hz), 7.40–7.43 (m, 6H), 7.53 (dd, 2H *J* = 8.3, 7.6 Hz), 7.56–7.60 (m, 6H), 7.61 (dd, 2H, *J* = 7.9, 7.6 Hz); ^{13}C NMR (150 MHz, $CDCl_3$) δ 20.6, 29.5, 35.3, 70.9, 99.4, 110.5, 111.1, 113.6, 115.6, 115.7, 118.1, 121.5, 126.7, 127.7, 127.8, 128.6 (2C), 128.7, 134.8, 136.5, 138.4, 142.3, 145.2, 147.9, 158.6, 169.2, 194.7; IR (neat) 3066, 2928, 1766, 1688, 1683, 1614, 1595, 1448, 1418, 1385, 1368, 1318, 1265, 1246, 1202, 1046, 1014, 985, 914, 801, 734 cm^{-1}; HRMS (ESI-TOF) calcd for $C_{58}H_{43}O_{12}$ ($[M+H]^+$) *m/z* 931.2749, found *m/z* 931.2731.

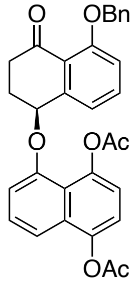

S13

Diacetate S13: R*f* 0.47 (toluene/EtOAc = 10/3); ^{1}H NMR (600 MHz, $CDCl_3$) δ 1.59 (s, 3H), 2.40–2.47 (m, 1H), 2.46 (s, 3H), 2.48–2.54 (m, 1H), 2.62 (ddd, 1H, *J* = 17.0, 7.7, 4.9), 2.95 (ddd, 1H, *J* = 17.0, 8.9, 4.9 Hz), 5.25 (d, 1H, *J* = 12.4 Hz), 5.28 (d, 1H, *J* = 12.4 Hz), 5.67 (dd, 1H, *J* = 6.5, 3.5), 6.99 (d, 1H, *J* = 8.2 Hz), 7.07 (d, 1H, *J* = 7.6 Hz), 7.08 (d, 1H, *J* = 8.0 Hz), 7.18 (d, 1H, *J* = 7.6 Hz), 7.22 (d, 1H, *J* = 8.2 Hz), 7.31 (t, 1H, *J* = 7.4 Hz), 7.40 (dd, 2H, *J* = 7.4, 7.3 Hz), 7.45 (dd, 1H, *J* = 8.3, 7.6 Hz), 7.515 (dd, 1H, *J* = 8.0, 7.6 Hz), 7.522 (d, 1H, *J* = 8.3 Hz), 7.58 (d, 2H, *J* = 7.3 Hz); ^{13}C NMR (150 MHz, $CDCl_3$) δ 20.3, 21.0, 26.5, 36.1, 70.6, 74.3, 108.8, 114.3, 114.8, 118.7, 119.1, 120.9, 121.0, 121.8, 126.7, 127.0, 127.8, 128.6, 130.1, 134.6, 136.5, 143.8, 144.3, 144.4, 153.4, 158.8, 169.3, 170.1, 195.7; IR (neat) 3065, 2936, 1760, 1682, 1595, 1583, 1511, 1451, 1414, 1368, 1264, 1223, 1196, 1041,

967, 909, 801, 735 cm^{-1}; HRMS (ESI-TOF) calcd for $C_{31}H_{26}NaO_7$ ([M+Na]$^+$) *m/z* 533.1571, found *m/z* 533.1559.

phenol **16**

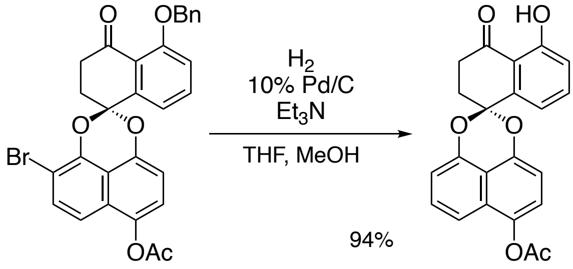

(*S*)-**22** >99% *ee* **16** >99% *ee*

A flask, thoroughly purged with argon, was added a solution of (*S*)-**22** (1.27 g, 2.33 mmol) in THF (64 mL) and MeOH (6.4 mL), to which was added 10% Pd/C (129 mg) and Et_3N (1.65 mL, 11.9 mmol). The atmosphere was changed from argon to H_2 (1 atm). After stirring for 3.5 h at room temperature, the atmosphere was changed from H_2 to argon, the mixture was filtrated through a Celite® pad (rinsed with EtOAc) and concentrated in vacuo. The residue was purified by flash column chromatography (silica gel, hexane/EtOAc = 85/15) to afford **16** (817 mg, 94%) as a pale-yellow oil.

16: R_f 0.64 (hexane/EtOAc = 1/1); $[\alpha]_D^{20}$ +28.2 (*c* 0.990, $CHCl_3$); ^{1}H NMR (600 MHz, $CDCl_3$) δ 2.46 (s, 3H), 2.50 (t, 2H, *J* = 6.5 Hz), 2.83 (t, 2H, *J* = 6.5 Hz), 6.95 (d, 1H, *J* = 8.1 Hz), 7.01 (dd, 1H, *J* = 7.0, 1.2 Hz), 7.10 (dd, 1H, *J* = 8.0, 0.9 Hz), 7.21 (d, 1H, *J* = 8.1 Hz), 7.43 (dd, 1H, *J* = 8.0, 0.9 Hz), 7.47 (dd, 1H, *J* = 8.5, 1.2 Hz), 7.50 (dd, 1H, *J* = 8.5, 7.0 Hz), 7.62 (t, 1H, *J* = 8.0 Hz), 12.43 (s, 1H, OH); ^{13}C NMR (150 MHz, $CDCl_3$) δ 20.9, 29.2, 34.0, 98.7, 109.0, 110.2, 113.7, 115.0, 115.3, 116.6, 119.7, 119.8, 127.3, 128.2, 137.2, 140.5, 140.9, 145.1, 147.6, 162.4, 169.6, 203.1; IR (neat) 3059, 2966, 1765, 1643, 1610, 1456, 1219, 1379, 1331, 1269, 1237, 1201, 1107, 952, 922, 888, 733 cm^{-1}; HRMS (ESI-TOF) calcd for $C_{22}H_{17}O_6$ ([M+H]$^+$) *m/z* 377.1020, found *m/z* 377.1027.

The enantiomeric excess of the phenol **16** was assessed by HPLC analysis with a chiral stationary phase [DAICEL CHIRALPAK® IB column (0.46 cm ϕ × 25 cm), eluent: hexane/EtOAc = 90/10, 1 mL/min, temperature: 25 °C, detector: UV 254 nm, retention time: 14.8 min for the (*S*)-isomer, 16.5 min for the (*R*)-isomer].

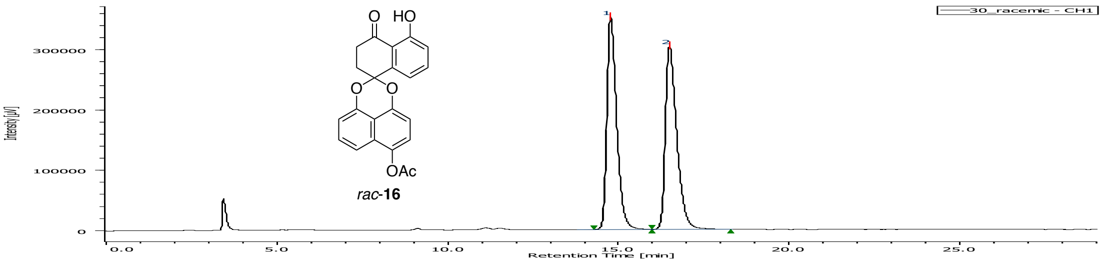

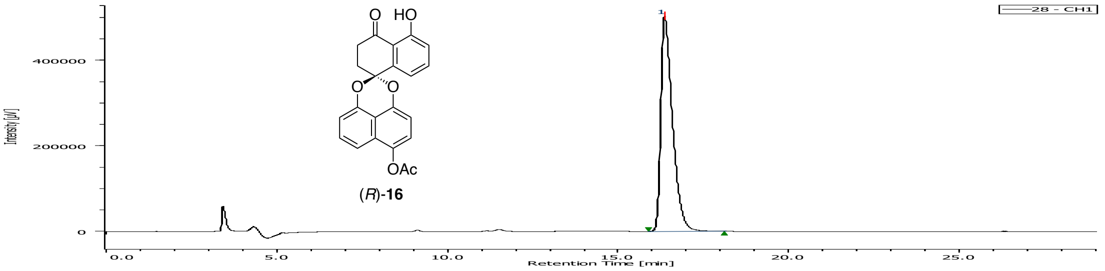

*To verify the absolute stereochemistry of **S11**, transformation to phenol **16** from **S11** was performed, and it was identical with phenol **16** from (S)-**22** by HPLC analysis on a chiral stationary phase. Thus, the stereochemistry of the spiroacetal center in **S11** was assigned as R configuration.*

phenol **16** from **S11**

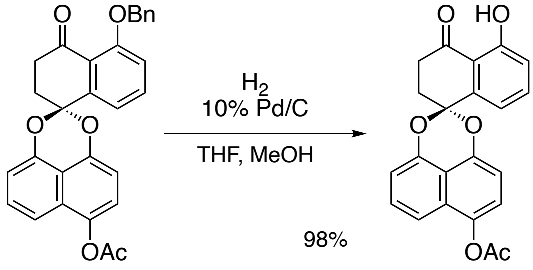

S11 98% *ee* **16** 98% *ee*

A flask, thoroughly purged with argon, was added a solution of **S11** (15.3 mg, 0.0328 mmol) in THF (0.90 mL) and MeOH (0.09 mL), to which was added 10% Pd/C (8.2 mg). The atmosphere was changed from argon to H_2 (1 atm). After stirring for 1.0 h at room temperature, the atmosphere was changed from H_2 to argon, the mixture was filtrated through a Celite® pad (rinsed with EtOAc) and concentrated in vacuo. The residue was purified by PTLC (silica gel, hexane/EtOAc = 3/2) to afford **16** (12.1 mg, 98%, 98% *ee*) as a pale-yellow oil.

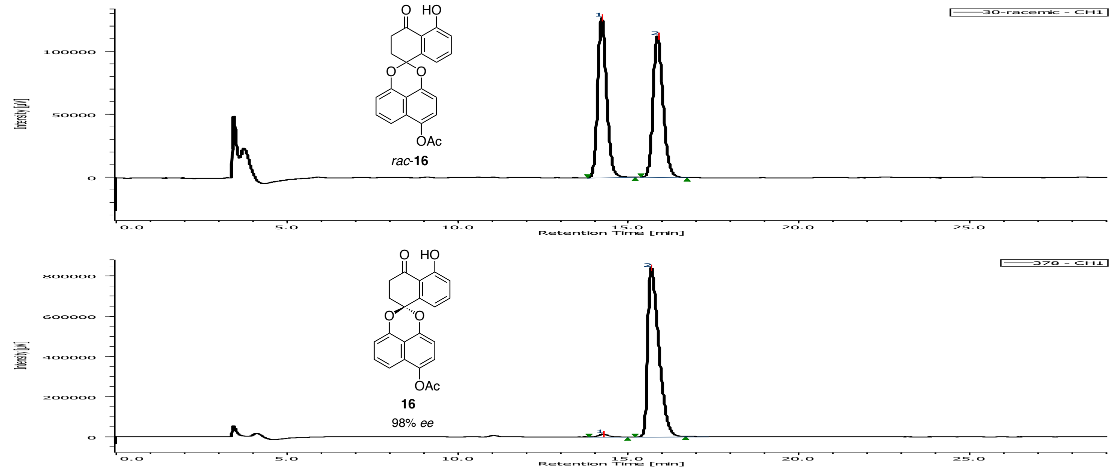

	tR /min	peak area	peak area /%
peak 1	14.267	237957	1.243
peak 2	15.667	18901109	98.757

alcohol **S14**

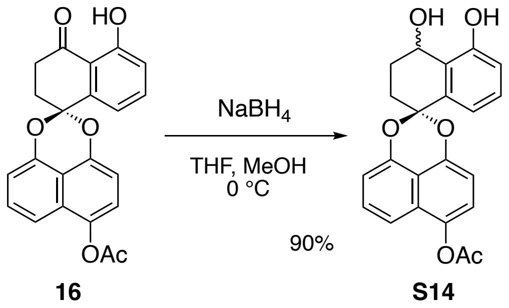

To a solution of **16** (817 mg, 2.17 mmol) in THF (41 mL) and MeOH (15 mL) was added $NaBH_4$ (82.2 mg, 2.17 mmol) at 0 °C. After stirring for 10 min at the same temperature, the reaction was stopped by adding saturated aqueous NH_4Cl. The mixture was poured into EtOAc and H_2O, and extracted with EtOAc (×3), and the combined organic extracts were washed with brine, dried (Na_2SO_4), and concentrated in vacuo. The residue was purified by flash column chromatography (silica gel, CH_2Cl_2/acetone = 50/1) to afford **S14** (738 mg, 90%, d.r. = 1:1) as a colorless solid.

S14 (mixture of diastereomers = 1:1): R*f* 0.41 (hexane/EtOAc = 1/1); $[\alpha]_D^{20}$ +22.3 (*c* 0.985, $CHCl_3$); ^1H NMR (600 MHz, $CDCl_3$) δ 1.89–1.96 (m, 1H), 1.98–2.06 (m, 1H), 2.15–2.21 (m, 1H), 2.32–2.40 (m, 1H), 2.45 (s, 3H), 2.68–2.73 (m, 1H, OH), 5.07–5.12 (m, 1H), 6.85 (d, 0.5H, *J* = 8.1 Hz), 6.89 (d, 0.5H, *J* = 8.2 Hz), 6.92 (d, 0.5H, *J* = 7.1 Hz), 6.95 (d, 0.5H, *J* = 7.9 Hz), 6.96 (d, 1H, *J* = 7.4 Hz), 7.17 (d, 0.5H, *J* = 7.1 Hz), 7.18 (d, 0.5H, *J* = 8.2 Hz), 7.33 (dd, 1H, *J* = 7.7, 7.4 Hz), 7.39 (d, 1H, *J* = 7.7 Hz), 7.42–7.48 (m, 2H), 7.97 (s, 0.5H, OH), 8.01 (s, 0.5H, OH); ^{13}C NMR (150 MHz, $CDCl_3$) δ 20.9, 27.79, 27.80, 28.59, 28.62, 67.6, 67.7, 99.9, 108.8, 108.9, 110.15, 110.20, 113.9, 114.53, 114.57, 117.77, 117.80, 118.8, 119.59, 119.64, 124.2, 127.2, 128.1, 128.2, 129.9, 135.7, 140.49, 140.53, 145.6, 145.7, 148.0, 148.1, 155.71, 155.74, 170.0; IR (neat) 3321, 2939, 1759, 1609, 1594, 1463, 1418, 1378, 1323, 1270, 1204, 1106, 1051, 1013, 953, 909, 731 cm^{-1}; HRMS (ESI-TOF) calcd for $C_{22}H_{18}NaO_6$ ($[M+Na]^+$) *m/z* 401.0996, found *m/z* 401.0993.

benzoquinone **23**

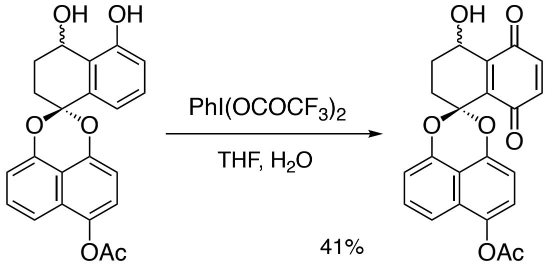

S14
dr = 1:1
23
d.r. = 1:1

To a solution of **S14** (476 mg, 1.26 mmol) in THF (28.5 mL) and H_2O (3.2 mL) was added $PhI(OCOCF_3)_2$ (1.15 mg, 2.67 mmol) at 0 °C. After stirring for 4.5 h at room temperature, the reaction was stopped by adding saturated aqueous $NaHCO_3$ at 0 °C. The mixture was extracted with EtOAc (×3), and the combined organic extracts were washed with brine, dried (Na_2SO_4), and concentrated in vacuo. The residue was purified by MPLC (High-flash 2L column, toluene/acetone = 32/1 → 8/1, detector; ESLD) to afford **23** (132 mg, 27%, d.r. = 1:1) as a red oil and mixture (139 mg) including **23**. The mixture was purified again by MPLC (High-flash L column, toluene/acetone = 32/1 → 8/1, detector; ELSD) to afford pure **23** (42.7 mg, 9%, d.r. = 1:1) as a red oil and mixture (38.7 mg) including **23**. Further purification of the mixture was performed by PTLC (silica gel, toluene/acetone = 3/1) to give **23** (22.3 mg, 5%, d.r. = 1:1; total yield = 41%) as a red oil.

23 (mixture of diastereomers = 1:1): R_f 0.44 (toluene/acetone = 3/1); $[\alpha]_D^{20}$ +62.2 (*c* 0.325, $CHCl_3$); ^1H NMR (600 MHz, $CDCl_3$) δ 1.95–2.00 (m, 1H), 2.03–2.12 (m, 2H), 2.28–2.34 (m, 1H), 2.45 (s, 3H), 3.09 (d, 0.5H, *J* = 2.9 Hz, OH), 3.12 (d, 0.5H, *J* = 2.9 Hz, OH), 4.89–4.91 (m, 1H), 6.81 (d, 1H, *J* = 10.2 Hz), 6.83 (d, 0.5H, *J* = 8.2 Hz), 6.85 (d, 1H, *J* = 10.2 Hz), 6.88 (d, 0.5H, *J* = 8.2 Hz), 6.90 (dd, 0.5H, *J* = 6.7, 1.7 Hz), 6.95 (dd, 0.5H, *J* = 7.3, 1.1 Hz), 7.18 (t, 1H, *J* = 8.2 Hz), 7.42–7.48 (m, 2H); ^{13}C NMR (150 MHz, $CDCl_3$) δ 20.9, 25.4, 27.4, 27.5, 62.5, 62.6, 98.72, 98.74, 108.6, 108.8, 109.9, 110.1, 113.2, 113.3, 114.6, 114.7, 119.5, 119.6, 127.3, 127.9, 128.1, 135.1, 135.6, 138.2, 140.57, 140.64, 144.2, 144.3, 144.4, 146.7, 146.9, 169.7, 183.50, 183.51, 188.98, 189.02; IR (neat) 3514, 3062, 2937, 1760, 1661, 1609, 1420, 1377, 1271, 1200, 1113, 974, 952, 732 cm^{-1}; HRMS (ESI-TOF) calcd for $C_{22}H_{17}O_7$ ($[M+H]^+$) *m/z* 393.0969, found *m/z* 393.0959.

preussomerin EG_3 (**5**)

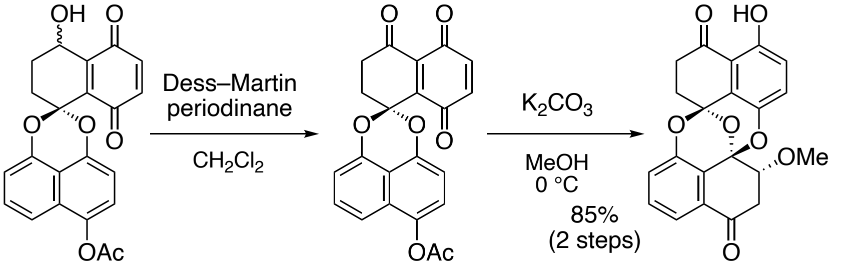

23 **24** (crude) preussomerin EG_3 (**5**)

To a solution of **23** (30.6 mg, 0.0780 mmol) in CH_2Cl_2 (3.9 mL) was added Dess–Martin periodinane (99.2 mg, 0.234 mmol) at 0 °C. After stirring for 1.0 h at room temperature, the reaction was stopped by adding saturated aqueous $NaHCO_3$ at 0 °C. The mixture was extracted with EtOAc (×3) and the combined organic extracts were washed with brine, dried (Na_2SO_4), and concentrated in vacuo. This crude material was used for the next experiment without further purification.

To a solution of this crude material, including **24** in MeOH (1.6 mL) was added K_2CO_3 (22.1 mg, 0.160 mmol) at 0 °C. After stirring for 1.0 h at the same temperature, the reaction was diluted with EtOAc and stopped by adding saturated aqueous NH_4Cl. The mixture was extracted with EtOAc (×3), and the combined organic extracts were washed with brine, dried (Na_2SO_4), and concentrated in vacuo. The residue was purified by flash column chromatography (silica gel, CH_2Cl_2) to afford **5** (25.3 mg, 85% from **23**) as a yellow solid.

benzoquinone 24 (crude): R_f 0.55 (diol silica gel, toluene/acetone = 3/1); ^{1}H NMR (600 MHz, $CDCl_3$) δ 2.46 (s, 3H), 2.51–2.59 (m, 2H), 2.75–2.81 (m, 2H), 6.82 (d, 1H, *J* = 10.3 Hz), 6.87 (d, 1H, *J* = 10.3 Hz), 6.91 (d, 1H, *J* = 8.2 Hz), 6.96–7.00 (m, 1H), 7.21 (d, 1H, *J* = 8.2 Hz), 7.47–7.51 (m, 2H); ^{13}C NMR (150 MHz, $CDCl_3$) δ 20.9, 31.0, 36.0, 98.0, 109.0, 110.2, 112.9, 115.3, 119.7, 127.4, 128.1, 130.2, 135.7, 137.0, 141.0, 141.4, 143.6, 146.1, 169.6, 184.0, 184.5, 194.5; HRMS (ESI-TOF) calcd for $C_{22}H_{14}NaO_7$ ($[M+Na]^+$) *m/z* 413.0632, found *m/z* 413.0636.

preussomerin EG₃ (5): R_f 0.30 (CH_2Cl_2); mp 198.8–199.4 °C (hexane/EtOAc); $[\alpha]_D^{20}$ –407 (*c* 0.382, MeOH); ^{1}H NMR (600 MHz, $CDCl_3$) δ 2.50 (ddd, 1H, *J* = 13.4, 13.4, 5.2 Hz), 2.80 (ddd, 1H, *J* = 13.4, 5.6, 1.7 Hz), 2.90 (ddd, 1H, *J* = 18.6, 5.2, 1.7 Hz), 3.08 (dd, 1H, *J* = 18.2, 2.7 Hz), 3.38 (ddd, 1H, *J* = 18.6, 13.4, 5.6 Hz), 3.39 (dd, 1H, *J* = 18.2, 3.0 Hz), 3.51 (s, 3H), 4.27 (dd, 1H, *J* = 3.0, 2.7 Hz), 6.92 (d, 1H, *J* = 9.1 Hz), 7.02 (d, 1H, *J* = 9.1 Hz), 7.08 (dd, 1H, *J* = 8.1, 0.8 Hz), 7.39 (dd, 1H, *J* = 8.1, 7.9 Hz), 7.63 (dd, 1H, *J* = 7.9, 0.8 Hz), 11.69 (s, 1H, OH); ^{13}C NMR (150 MHz, $CDCl_3$) δ 32.8, 33.8, 40.4, 59.2, 79.3, 93.1, 94.4, 112.9, 119.9, 120.2 (2C), 120.7, 121.6, 126.0, 130.9 (2C), 140.9, 150.6, 157.3, 193.8, 202.2; IR (ATR) 3294, 2917, 2849, 1704, 1689, 1647, 1596, 1471, 1330, 1287, 1104, 980 cm^{-1}; UV–Vis (MeOH) λ_{max} nm (ε) = 345 (4339), 305 (3675), 245 (14659), 207 (25496); HRMS (ESI-TOF) calcd for $C_{21}H_{17}O_7$ ($[M+H]^+$) *m/z* 381.0969, found *m/z* 381.0951.

Crystallographic data: $C_{21}H_{16}O_7$, Formula Weight = 380.34, 0.216 ×0.161 ×0.015 mm, monoclinic, space group $P2_1$, Z = 4, T = 106 K, *a* = 8.64837(16), *b* = 8.92177(16), *c* = 22.0206(4) Å, V = 1690.69(5) $Å^3$, λ (CuKα) = 1.54186 Å, μ = 0.953 mm^{-1} Intensity data were collected on Rigaku R-AXIS RAPID AUTO. The structure was solved by direct methods and refined by the full-matrix least-squares on F^2 (SHELXL-2018/3). A total of 31696 reflections were measured and 5898 were independent. Final *R*1 = 0.0423, *wR*2 = 0.1014 (5165 refs; *I* > 2s(*I*)), and GOF = 1.093 (for all data, *R*1 = 0.0495, *wR*2 = 0.1091). Flack Parameter = 0.03(9).

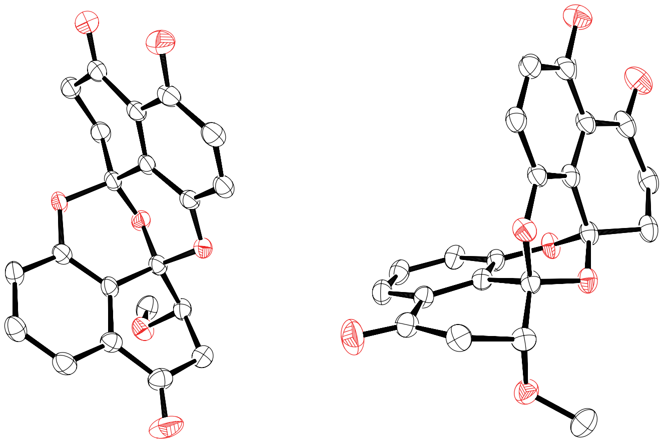

X-ray structure of **5** (front and side views)

(CCDC 2177101)

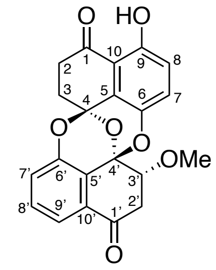

preussomerin EG_3 (**5**)

^1H NMR

natural (500 MHz, $CDCl_3$)*	synthetic (600 MHz, $CDCl_3$)
δ 2.50 (ddd, 1H, *J* = 13.5, 13.5, 5.5 Hz, 3a)	δ 2.50 (ddd, 1H, *J* = 13.4, 13.4, 5.2 Hz, 3a)
δ 2.80 (ddd, 1H, *J* = 13.5, 5.5, 2.0 Hz, 3b)	δ 2.80 (ddd, 1H, *J* = 13.4, 5.6, 1.7 Hz, 3b)
δ 2.90 (ddd, 1H, *J* = 18.6, 5.5, 2.0 Hz, 2a)	δ 2.90 (ddd, 1H, *J* = 18.6, 5.2, 1.7 Hz, 2a)
δ 3.08 (dd, 1H, *J* = 18.0, 3.0 Hz, 2'a)	δ 3.08 (dd, 1H, *J* = 18.2, 2.7 Hz, 2'a)
δ 3.37 (ddd, 1H, *J* = 18.6, 13.5, 5.5 Hz, 2b)	δ 3.38 (ddd, 1H, *J* = 18.6, 13.4, 5.6 Hz, 2b)
δ 3.39 (dd, 1H, *J* = 18.0, 3.0 Hz, 2'b)	δ 3.39 (dd, 1H, *J* = 18.2, 3.0 Hz, 2'b)
δ 3.51 (s, 3H, CH_3O-3')	δ 3.51 (s, 3H, CH_3O-3')
δ 4.27 (dd, 1H, *J* = 3.0, 3.0 Hz, 3')	δ 4.27 (dd, 1H, *J* = 3.0, 2.7 Hz, 3')
δ 6.92 (d, 1H, *J* = 9.0 Hz, 8)	δ 6.92 (d, 1H, *J* = 9.1 Hz, 8)
δ 7.02 (d, 1H, *J* = 9.0 Hz, 7)	δ 7.02 (d, 1H, *J* = 9.1 Hz, 7)
δ 7.08 (dd, 1H, *J* = 8.0, 1.0 Hz, 7')	δ 7.08 (dd, 1H, *J* = 8.1, 0.8 Hz, 7')
δ 7.38 (dd, 1H, *J* = 8.0, 8.0 Hz, 8')	δ 7.39 (dd, 1H, *J* = 8.1, 7.9 Hz, 8')
δ 7.63 (dd, 1H, *J* = 8.0, 1.0 Hz, 9')	δ 7.63 (dd, 1H, *J* = 7.9, 0.8 Hz, 9')
δ 11.71 (s, 1H, OH-9)	δ 11.69 (s, 1H, OH-9)

*A. L. Anaya *et al., Phytochemistry* **2008**, *69*, 1185.

^{13}C NMR

natural* (125 MHz, $CDCl_3$)	synthetic (150 MHz, $CDCl_3$)	natural* (125 MHz, $CDCl_3$)	synthetic (150 MHz, $CDCl_3$)
δ 32.8 (3)	δ 32.8 (3)	δ 120.7 (8)	δ 120.7 (8)
δ 33.8 (2)	δ 33.8 (2)	δ 121.6 (7')	δ 121.6 (7')
δ 40.4 (2')	δ 40.4 (2')	δ 126.0 (7)	δ 126.0 (7)
δ 59.2 (CH_3O-3')	δ 59.2 (CH_3O-3')	δ 130.9 (8', 10')	δ 130.9 (8', 10')
δ 79.3 (3')	δ 79.3 (3')	δ 140.9 (6)	δ 140.9 (6)
δ 93.2 (4)	δ 93.1 (4)	δ 150.6 (6')	δ 150.6 (6')
δ 94.4 (4')	δ 94.4 (4')	δ 157.3 (9)	δ 157.3 (9)
δ 113.0 (10)	δ 112.9 (10)	δ 193.7 (1')	δ 193.8 (1')
δ 119.9 (5')	δ 119.9 (5')	δ 202.2 (1)	δ 202.2 (1)
δ 120.3 (5, 9')	δ 120.2 (5, 9')		

*A. L. Anaya *et al., Phytochemistry* **2008**, *69*, 1185.

alcohol **29**

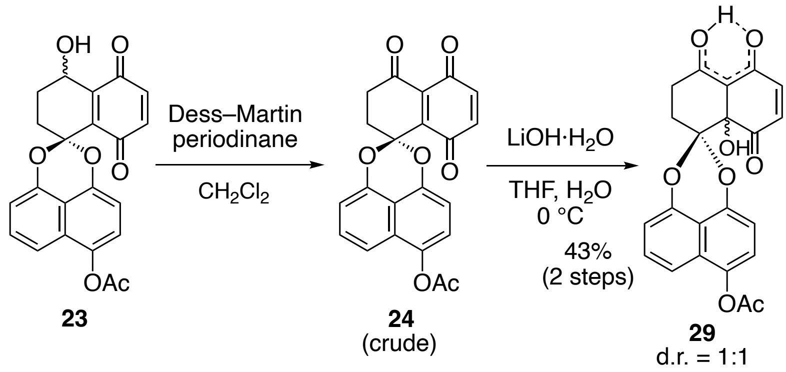

To a solution of **23** (14.2 mg, 0.0362 mmol) in CH_2Cl_2 (1.8 mL) was added Dess–Martin periodinane (46.1 mg, 0.109 mmol) at 0 °C. After stirring for 1.0 h at room temperature, the reaction was stopped by adding saturated aqueous $NaHCO_3$ at 0 °C. The mixture was extracted with EtOAc (×3) and the combined organic extracts were washed with brine, dried (Na_2SO_4), and concentrated in vacuo. This crude material was used for the next experiment without further purification.

To a solution of this crude material, including **24** in THF (1.5 mL) and H_2O (0.35 mL) was added LiOH·H_2O (3.2 mg, 0.0763 mmol) at 0 °C. After stirring for 0.5 h at the same temperature, the reaction was stopped by adding saturated aqueous NH_4Cl. The mixture was extracted with EtOAc (×3), and the combined organic extracts were washed with brine, dried (Na_2SO_4), and concentrated in vacuo. The residue was purified by PTLC (diol silica gel, CH_2Cl_2/acetone = 25/1) to afford **29** (6.3 mg, 43% from **23**, d.r. = 1:1) as a yellow solid and **26** (0.4 mg, 3% from **23**) as a yellow oil.

29 (mixture of diastereomers = 1:1): R_f 0.60 (diol silica gel, CH_2Cl_2/acetone = 25/1); ^1H NMR (600 MHz, $CDCl_3$) δ 2.21 (ddd, 1H, J = 14.4, 6.5, 3.4 Hz), 2.40–2.46 (m, 4H), 2.53–2.66 (m, 2H), 3.81 (s, 1H, OH), 6.54 (d, 0.5H, J = 10.3 Hz), 6.55 (d, 0.5H, J = 10.3 Hz), 6.84 (d, 0.5H, J = 8.2 Hz), 6.861 (d, 0.5H, J = 10.3 Hz), 6.862 (d, 0.5H, J = 10.3 Hz), 6.90 (dd, 0.5H, J = 4.4, 4.1 Hz), 6.97 (d, 0.5H, J = 8.2 Hz), 7.04 (d, 0.5H, J = 7.6 Hz), 7.16 (d, 0.5H, J = 8.2 Hz), 7.21 (d, 0.5H, J = 8.2 Hz), 7.44 (m, 1.5H), 7.49 (dd, 0.5H, J = 8.3, 7.6 Hz), 15.55 (s, 1H, OH); ^{13}C NMR (150 MHz, $CDCl_3$) δ 20.9, 24.66, 24.71, 28.7, 72.2, 101.66, 101.70, 107.25, 107.26, 109.0, 109.1, 110.2, 110.3, 113.5, 114.9, 115.3, 119.6, 119.7, 127.37, 127.40, 128.1, 128.2, 136.75, 136.76, 138.71, 138.73, 140.8, 141.0, 143.76, 143.81, 146.26, 146.30, 169.6, 182.3, 188.25, 188.27, 192.6; IR (neat) 3437, 3062, 2938, 1760, 1704, 1634, 1611, 1420, 1371, 1268, 1201, 1124, 1042, 1012, 908, 731 cm^{-1}; HRMS (ESI-TOF) calcd for $C_{22}H_{17}O_8$ ($[M+H]^+$) m/z 409.0918, found m/z 409.0909.

hydroquinone **26**

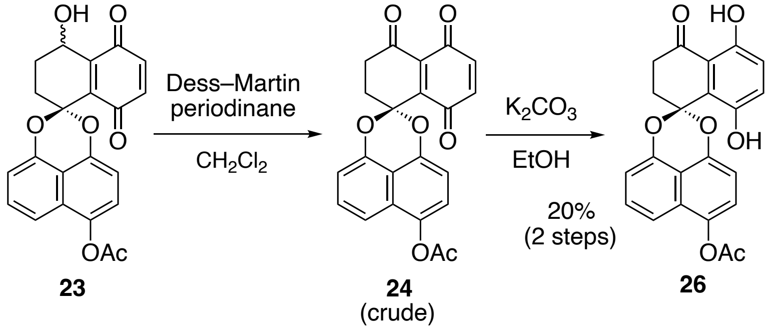

To a solution of **23** (16.4 mg, 0.0418 mmol) in CH_2Cl_2 (2.1 mL) was added Dess–Martin periodinane (53.7 mg, 0.127 mmol) at 0 °C. After stirring for 45 min at room temperature, the reaction was stopped by adding saturated aqueous $NaHCO_3$ at 0 °C. The mixture was extracted with EtOAc (×3) and the combined organic extracts were washed with brine, dried (Na_2SO_4), and concentrated in vacuo. This crude material was used for the next experiment without further purification.

To a solution of this crude material, including **24** in EtOH (0.85 mL) was added K_2CO_3 (11.7 mg, 0.0847 mmol) at 0 °C. After stirring for 1.3 h at room temperature, the reaction was stopped by adding saturated aqueous NH_4Cl at 0 °C. The mixture was extracted with EtOAc (×3), and the combined organic extracts were washed with brine, dried (Na_2SO_4), and concentrated in vacuo. The residue was purified by PTLC (silica gel, CH_2Cl_2 only) to afford **26** (3.2 mg, 20% from **23**) as a yellow oil and **3** (0.3 mg: impure, ca. 2%) as a colorless oil.

26: R*f* 0.41 (hexane/EtOAc = 2/1); $[\alpha]_D^{20}$ +15.4 (*c* 0.85, $CHCl_3$); ^{1}H NMR (600 MHz, acetone-d_6) δ 2.45 (s, 3H), 2.56 (t, 2H, *J* = 6.6 Hz), 2.78 (t, 2H, *J* = 6.6 Hz), 7.08 (d, 1H, *J* = 9.2 Hz), 7.10 (d, 1H, *J* = 8.1 Hz), 7.15 (dd, 1H, *J* = 7.0, 1.2 Hz), 7.29 (d, 1H, *J* = 9.2 Hz), 7.31 (d, 1H, *J* = 8.1 Hz), 7.58 (dd, 1H, *J* = 8.3, 7.0 Hz), 7.61 (dd, 1H, *J* = 8.3, 1.1 Hz), 8.09 (s, 1H, OH), 12.39 (s, 1H, OH); ^{1}H NMR (600 MHz, $CDCl_3$) δ 2.47 (s, 3H), 2.53 (dd, 2H, *J* = 6.8, 6.3 Hz), 2.76 (dd, 2H, *J* = 6.8, 6.3 Hz), 7.04 (d, 1H, *J* = 8.2 Hz), 7.08 (d, 1H, *J* = 9.2 Hz), 7.10 (dd, 1H, *J* = 6.7, 1.7 Hz), 7.25 (d, 1H, *J* = 8.2 Hz), 7.26 (d, 1H, *J* = 9.2 Hz), 7.53 (dd, 1H, *J* = 8.5, 6.7 Hz), 7.55 (dd, 1H, *J* = 8.5, 1.7 Hz), 7.52–7.56 (m, 1H, OH) 12.36 (s, 1H, OH); ^{13}C NMR (150 MHz, acetone-d_6) δ 20.8, 29.5–30.2(1C), 34.5, 102.5, 110.7, 111.8, 115.0, 115.6, 116.4, 120.9, 121.4, 122.2, 128.4, 129.1, 129.7, 142.4, 145.5, 148.2, 149.0, 157.5, 170.1, 204.1; ^{13}C NMR (150 MHz, $CDCl_3$) δ 20.9, 28.9, 33.8, 102.3, 110.1, 111.3, 114.0, 114.4, 116.1, 119.5, 119.9, 122.0, 127.4, 128.3, 128.9, 141.6, 143.9, 146.5, 147.6, 157.2, 169.6, 202.3; IR (neat) 3471, 3061, 2972, 1766, 1703, 1645, 1613, 1594, 1477, 1419, 1378, 1335, 1314, 1266, 1235, 1201, 1164, 1092, 1079, 1035, 1013, 952, 910, 875 cm^{-1}; HRMS (ESI-TOF) calcd for $C_{22}H_{17}O_7$ ($[M+H]^+$) *m/z* 393.0969, found *m/z* 393.0966.

preussomerin EG_1 (**3**)

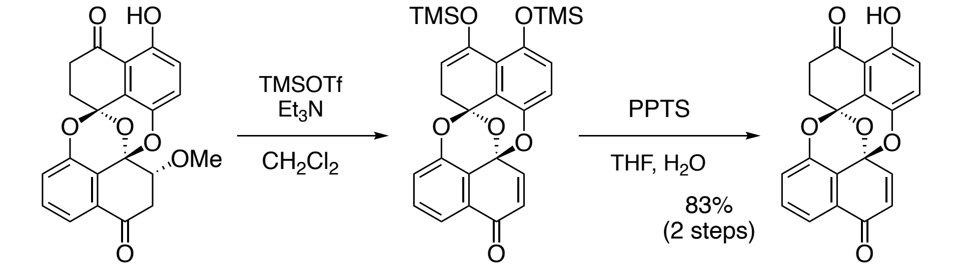

preussomerin EG_3 (**5**) **30** (crude) preussomerin EG_1 (**3**)

To a solution of **5** (23.7 mg, 0.0623 mmol) in CH_2Cl_2 (3.0 mL) was added Et_3N (87.5 µL, 0.631 mmol), TMSOTf (115 µL, 0.636 mmol) at 0 °C. After stirring for 1.0 h at room temperature, the reaction was stopped by adding saturated aqueous $NaHCO_3$ at 0 °C. The mixture was extracted with EtOAc (×3), and the combined organic extracts were washed with brine, dried (Na_2SO_4), and concentrated in vacuo. This crude material was used for the next experiment without further purification.

To a solution of this crude material, including **30** in THF (1.5 mL) and H_2O (0.15 mL) was added pyridinium *p*-toluenesulfonate (1.6 mg, 6.37 µmol) at 0 °C. After stirring for 2.8 h at room temperature, the reaction was stopped by adding saturated aqueous $NaHCO_3$ at 0 °C. The mixture was extracted with EtOAc (×3), and the combined organic extracts were washed with brine, dried (Na_2SO_4), and

concentrated in vacuo. The residue was purified by PTLC (silica gel, CH_2Cl_2) to afford **3** (18.0 mg, 83%) as a pale-yellow solid.

Enone 30 (crude): R_f 0.73 (CH_2Cl_2); ^1H NMR (600 MHz, $CDCl_3$) δ 0.20 (s, 9H), 0.22 (s, 9H), 2.87 (d, 2H, *J* = 4.9 Hz), 5.25 (t, 1H, *J* = 4.9 Hz), 6.53 (d, 1H, *J* = 10.0 Hz), 6.62 (d, 1H, *J* = 8.9 Hz), 6.69 (d, 1H, *J* = 8.9 Hz), 6.94 (dd, 1H, *J* = 8.2, 0.7 Hz), 7.17 (d, 1H, *J* = 10.0 Hz), 7.34 (dd, 1H, *J* = 8.2, 7.6 Hz), 7.54 (dd, 1H, *J* = 7.6, 0.7 Hz); ^{13}C NMR (150 MHz, $CDCl_3$) δ 0.024, 0.17, 29.7, 32.8, 89.5, 95.3, 103.3, 116.7, 117.1, 119.8, 120.5, 120.9, 122.8, 124.8, 130.6, 133.1, 141.9, 143.6, 145.9, 148.2, 150.7, 184.5; HRMS (ESI-TOF) calcd for $C_{26}H_{29}O_6Si_2$ ($[M+H]^+$) *m/z* 493.1497, found *m/z* 493.1491.

preussomerin EG₁ (3): R_f 0.47 (CH_2Cl_2); mp 227–229 °C (hexane/EtOAc, decomp.); $[\alpha]_D^{20}$ –543 (*c* 1.06, CH_2Cl_2); ^1H NMR (600 MHz, $CDCl_3$) δ 2.53 (ddd, 1H, *J* = 13.5, 13.5, 5.1 Hz), 2.75 (ddd, 1H, *J* = 13.5, 5.6, 1.7 Hz), 2.91 (ddd, 1H, *J* = 19.0, 5.1, 1.7 Hz), 3.36 (ddd, 1H, *J* = 19.0, 13.5, 5.6 Hz) , 6.57 (d, 1H, *J* = 10.0 Hz), 6.92 (d, 1H, *J* = 9.1 Hz), 7.02 (d, 1H, *J* = 9.1 Hz), 7.04 (d, 1H, *J* = 8.0 Hz), 7.18 (d, 1H, *J* = 10.0 Hz), 7.39 (dd, 1H, *J* = 8.0, 7.8 Hz), 7.60 (d, 1H, *J* = 7.8 Hz), 11.72 (s, 1H, OH); ^{13}C NMR (150 MHz, $CDCl_3$) δ 32.6, 33.7, 89.6, 93.7 112.6, 119.5, 120.4, 120.5, 120.6 120.7, 126.2, 130.4, 130.8, 133.3, 141.2, 141.4, 149.5, 157.2, 184.1, 202.0; IR (ATR) 1677, 1642, 1590, 1471, 1292, 1273, 1231, 1179, 1141, 1048, 1013, 947, 904, 833, 786, 762 cm^{-1}; UV–Vis (CH_2Cl_2) λ_{max} nm (ε) = 339 (5446), 255 (8549), 221 (21618); HRMS (ESI-TOF) calcd for $C_{20}H_{13}O_6$ ($[M+H]^+$) *m/z* 349.0707, found *m/z* 349.0703.

key HMBC correlations

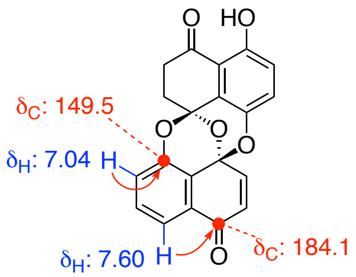

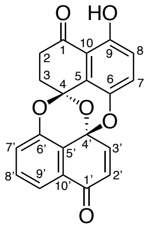

preussomerin EG₁ (**3**)

^{1}H NMR[6]

natural (500 MHz, $CDCl_3$)*	synthetic (600 MHz, $CDCl_3$)
δ 2.53 (ddd, 1H, *J* = 13.5, 13.5, 5.5 Hz, 3a)	δ 2.53 (ddd, 1H, *J* = 13.5, 13.5, 5.1 Hz, 3a)
δ 2.74 (ddd, 1H, *J* = 13.5, 5.5, 2.0 Hz, 3b)	δ 2.75 (ddd, 1H, *J* = 13.5, 5.6, 1.7 Hz, 3b)
δ 2.90 (ddd, 1H, *J* = 19.0, 5.5, 2.0 Hz, 2a)	δ 2.91 (ddd, 1H, *J* = 19.0, 5.1, 1.7 Hz, 2a)
δ 3.35 (ddd, 1H, *J* = 19.0, 13.5, 5.5 Hz, 2b)	δ 3.36 (ddd, 1H, *J* = 19.0, 13.5, 5.6 Hz, 2b)
δ 6.56 (d, 1H, *J* = 9.5 Hz, 2')	δ 6.57 (d, 1H, *J* = 10.0 Hz, 2')
δ 6.91 (d, 1H, *J* = 9.0 Hz, 8)	δ 6.92 (d, 1H, *J* = 9.1 Hz, 8)
δ 7.01 (d, 1H, *J* = 9.0 Hz, 7)	δ 7.02 (d, 1H, *J* = 9.1 Hz, 7)
δ 7.03 (dd, 1H, *J* = 8.0, 1.0 Hz, **9'**)	δ 7.04 (d, 1H, *J* = 8.0 Hz, **7'**)
δ 7.17 (d, 1H, *J* = 9.5 Hz, 3')	δ 7.18 (d, 1H, *J* = 10.0 Hz, 3')
δ 7.38 (dd, 1H, *J* = 8.0, 7.5 Hz, 8')	δ 7.39 (dd, 1H, *J* = 8.0, 7.8 Hz, 8')
δ 7.59 (dd, 1H, *J* = 7.5, 1.0 Hz, **7'**)	δ 7.60 (d, 1H, *J* = 7.8 Hz, **9'**)
δ 11.71 (s, 1H, OH-9)	δ 11.72 (s, 1H, OH)

*A. L. Anaya *et al., Phytochemistry* **2008**, *69*, 1185.

^{13}C NMR[6]

natural* (125 MHz, $CDCl_3$)	synthetic (150 MHz, $CDCl_3$)	natural* (125 MHz, $CDCl_3$)	synthetic (150 MHz, $CDCl_3$)
δ 32.6 (3)	δ 32.6 (3)	δ 126.2 (7)	δ 126.2 (7)
δ 33.7 (2)	δ 33.7 (2)	δ 130.4 (10')	δ 130.4 (10')
δ 89.6 (4')	δ 89.6 (4')	δ 130.8 (8')	δ 130.8 (8')
δ 93.7 (4)	δ 93.7 (4)	δ 133.3 (2')	δ 133.3 (2')
δ 112.6 (10)	δ 112.6 (10)	δ 141.2 (3')	δ 141.2 (3')
δ 119.5 (5)	δ 119.5 (5)	δ 141.4 (6)	δ 141.4 (6)
δ 120.4 **(7')**	δ 120.4 **(9')**	δ 149.5 (6')	δ 149.5 (6')
δ 120.6 (5', **9'**)	δ 120.5 (5')	δ 157.3 (9)	δ 157.2 (9)
	δ 120.6 **(7')**	δ 184.1 (1')	δ 184.1 (1')
δ 120.7 (8)	δ 120.7 (8)	δ 201.9 (1)	δ 202.0 (1)

*A. L. Anaya *et al., Phytochemistry* **2008**, *69*, 1185.

6 We unambiguously assigned the all signals in the ^{1}H and the ^{13}C NMR based on the extensive 2D NMR analyses (COSY, HMBC, HSQC), in which misassigns were found in the literature (indicated by red characters).

preussomerin EG_2 (**4**)

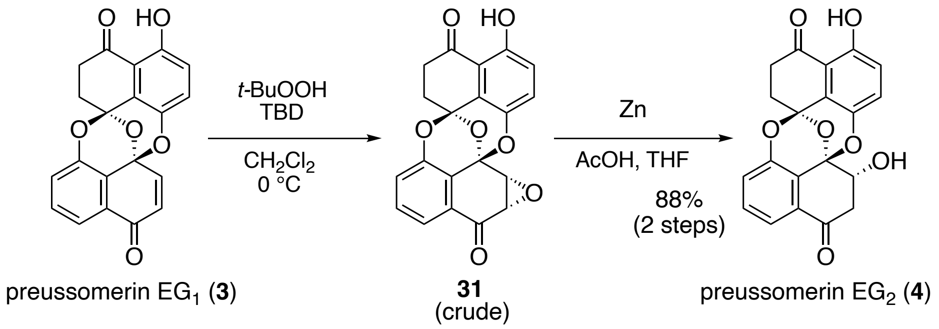

To a solution of **3** (11.2 mg, 0.0322 mmol) in CH_2Cl_2 (0.7 mL) was added *t*-BuOOH (13.0 μL, 0.0715 mmol, 5.5 M in decane), and 1,5,7-triazabicyclo[4,4,0]dec-5-ene (2.3 mg, 0.0165 mmol) at 0 °C. After stirring for 1.5 h at the same temperature, the reaction was stopped by adding 10% aqueous $Na_2S_2O_3$ at 0 °C. The mixture was extracted with EtOAc (×3), and the combined organic extracts were washed with brine, dried (Na_2SO_4), and concentrated in vacuo. This crude material was used for the next experiment without further purification. A small portion was purified by PTLC (silica gel, CH_2Cl_2) to give analytically pure sample.

To a solution of this crude material, including **31** in THF (0.4 mL) and AcOH (0.4 mL) was added Zn dust (22.0 mg, 0.337 mmol) at room temperature. After stirring for 18 h at the same temperature, the mixture was filtrated through a Celite® pad (rinsed with EtOAc), concentrated to half its volume in vacuo and quenched by adding saturated aqueous $NaHCO_3$ at 0 °C. The mixture was extracted with EtOAc (×3), and the combined organic extracts were washed with saturated aqueous $NaHCO_3$, and brine, dried (Na_2SO_4), and concentrated in vacuo. The residue was purified by PTLC (silica gel, hexane/EtOAc = 1/1) to afford **4** (10.4 mg, 88% from **3**) as a beige solid.

Epoxide 31: R_f 0.53 (CH_2Cl_2); $[\alpha]_D^{20}$ –575 (*c* 0.885, $CHCl_3$); ^{1}H NMR (600 MHz, $CDCl_3$) δ 2.53 (ddd, 1H, *J* = 13.4, 13.4, 5.2 Hz), 2.81 (ddd, 1H, *J* = 13.4, 5.6, 1.8 Hz), 2.92 (ddd, 1H, *J* = 18.7, 5.2, 1.8 Hz), 3.36 (ddd, 1H, *J* = 18.7, 13.4, 5.6 Hz), 3.87 (d, 1H, *J* = 4.1 Hz), 4.21 (d, 1H, *J* = 4.1 Hz), 6.93 (d, 1H, *J* = 9.2 Hz), 7.01 (d, 1H, *J* = 9.2 Hz), 7.10 (dd, 1H, *J* = 7.8, 1.4 Hz), 7.42 (dd, 1H, *J* = 7.8, 7.6 Hz), 7.45 (dd, 1H, *J* = 7.6, 1.4 Hz), 11.71 (s, 1H, OH); ^{13}C NMR (150 MHz, $CDCl_3$) δ 32.5, 33.7, 52.3, 53.4, 93.4, 93.5, 112.8, 117.5, 119.9, 120.5, 120.9, 121.9, 125.9, 128.7, 131.7, 140.5, 150.4, 157.4, 192.3, 201.9; IR (neat) 1704, 1650, 1594, 1470, 1329, 1301, 1270, 1101, 1001, 987, 959, 915, 879, 761, 731cm^{-1}; HRMS (ESI-TOF) calcd for $C_{20}H_{13}O_7$ ($[M+H]^+$) *m/z* 365.0656, found *m/z* 365.0658.

preussomerin EG_2 (**4**): R_f 0.41 (hexane/EtOAc = 1/1); mp 248–249 °C (hexane/EtOAc, partially decomp.); $[\alpha]_D^{20}$ –457 (*c* 1.20, MeOH); ^{1}H NMR (600 MHz, $CDCl_3$) δ 2.41–2.43 (m, 1H, OH), 2.50

(ddd, 1H, J = 13.4, 13.4, 5.2 Hz), 2.76 (ddd, 1H, J = 13.4, 5.5, 1.5 Hz), 2.91 (ddd, 1H, J = 18.6, 5.2, 1.5 Hz), 3.02 (dd, 1H, J = 18.4, 2.5 Hz), 3.37 (ddd, 1H, J = 18.6, 13.4, 5.5 Hz), 3.38 (dd, 1H, J = 18.4, 5.4 Hz), 4.71 (dd, 1H, J = 5.4, 2.5 Hz), 6.94 (d, 1H, J = 9.2 Hz), 7.04 (d, 1H, J = 9.2 Hz), 7.09 (d, 1H, J = 8.2 Hz), 7.41 (dd, 1H, J = 8.2, 7.7 Hz), 7.65 (d, 1H, J = 7.7 Hz), 11.71 (s, 1H, OH); ^{13}C NMR (150 MHz, $CDCl_3$) δ 32.6, 33.7, 41.3, 70.2, 93.3, 93.9, 112.9, 119.2, 120.1, 120.4, 120.8, 121.5, 126.0, 130.8, 131.1, 140.9, 150.7, 157.4, 193.6, 202.0; IR (neat) 3463, 2917, 1693, 1650, 1594, 1471, 1326, 1288, 1090, 1051, 988, 913, 733 cm^{-1}; UV–Vis (MeOH) λ_{max} nm (ε) = 347(4354), 306(3634), 246(14867), 209(27971); HRMS (ESI-TOF) calcd for $C_{20}H_{15}O_7$ ([M+H]$^+$) *m/z* 367.0823, found *m/z* 367.0814.

Crystallographic data: $C_{24}H_{22}O_9$, Formula Weight = 454.41, 0.249 ×0.080 ×0.052 mm, monoclinic, space group $P2_1$, Z = 2, T = 105 K, a = 8.2978(2), b = 15.2225(3), c =8.3959(2) Å, V = 1047.47(4) Å^3, λ (CuKα) = 1.54186 Å, μ = 0.937 mm^{-1} Intensity data were collected on Rigaku R-AXIS RAPID AUTO. The structure was solved by direct methods and refined by the full-matrix least-squares on F^2 (SHELXL-2018/1). A total of 26966 reflections were measured and 3788 were independent. Final $R1$ = 0.0395, $wR2$ = 0.0983 (3507 refs; $I > 2s(I)$), and GOF = 1.088 (for all data, $R1$ = 0.0428, $wR2$ = 0.1020). Flack Parameter = –0.05 (7).

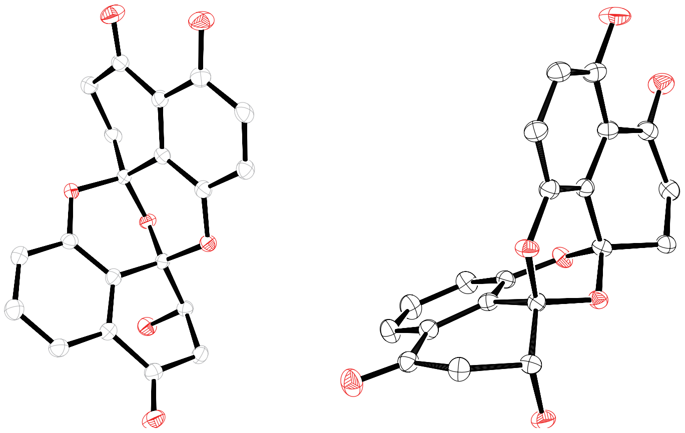

X-ray structure of **4** (front and side views)

(CCDC 2177102)

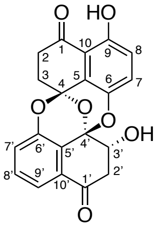

preussomerin EG_2 (**4**)

^{1}H NMR

natural (500 MHz, $CDCl_3$)	synthetic (600 MHz, $CDCl_3$)
δ 2.38 (1H, OH-3')	δ 2.41–2.43 (m, 1H, OH-3')
δ 2.50 (ddd, 1H, *J* = 13.5, 13.5, 5.5 Hz, 3a)	δ 2.50 (ddd, 1H, *J* = 13.4, 13.4, 5.2 Hz, 3a)
δ 2.77 (ddd, 1H, *J* = 13.5, 5.5, 2.0 Hz, 3b)	δ 2.76 (ddd, 1H, *J* = 13.4, 5.5, 1.5 Hz, 3b)
δ 2.91 (ddd, 1H, *J* = 18.8, 13.5, 5.5 Hz, 2a)	δ 2.91 (ddd, 1H, *J* = 18.6, 5.2, 1.5 Hz, 2a)
δ 3.02 (dd, 1H, *J* = 18.3, 3.0 Hz, 2a')	δ 3.02 (dd, 1H, *J* = 18.4, 2.5 Hz, 2'a)
δ 3.37 (ddd, 1H, *J* = 18.8, 5.5, 2.0 Hz, 2b)	δ 3.37 (ddd, 1H, *J* = 18.6, 13.4, 5.5 Hz, 2b)
δ 3.38 (dd, 1H, *J* = 18.3, 3.0 Hz, 2b')	δ 3.38 (dd, 1H, *J* = 18.4, 5.4 Hz, 2'b)
δ 4.71 (dd, 1H, *J* = 3.0, 3.0 Hz, 3')	δ 4.71 (dd, 1H, *J* = 5.4, 2.5 Hz, 3')
δ 6.94 (d, 1H, *J* = 9.0 Hz, 8)	δ 6.94 (d, 1H, *J* = 9.2 Hz, 8)
δ 7.04 (d, 1H, *J* = 9.0 Hz, 7)	δ 7.04 (d, 1H, *J* = 9.2 Hz, 7)
δ 7.09 (dd, 1H, *J* = 8.0, 1.0 Hz, 7')	δ 7.09 (d, 1H, *J* = 8.2 Hz, 7')
δ 7.41 (dd, 1H, *J* = 8.0, 8.0 Hz, 8')	δ 7.41 (dd, 1H, *J* = 8.2, 7.7 Hz, 8')
δ 7.66 (dd, 1H, *J* = 8.0, 1.0 Hz, 9')	δ 7.65 (d, 1H, *J* = 7.7 Hz, 9')
δ 11.71 (s, 1H, OH-9)	δ 11.71 (s, 1H, OH-9)

A. L. Anaya *et al., Phytochemistry* **2008**, *69*, 1185.

^{13}C NMR

natural (125 MHz, $CDCl_3$)	synthetic (150 MHz, $CDCl_3$)	natural (125 MHz, $CDCl_3$)	synthetic (150 MHz, $CDCl_3$)
δ 32.7 (3)	δ 32.6 (3)	δ 120.9 (8)	δ 120.8 (8)
δ 33.7 (2)	δ 33.7 (2)	δ 121.5 (7')	δ 121.5 (7')
δ 41.3 (2')	δ 41.3 (2')	δ 126.0 (7)	δ 126.0 (7)
δ 70.3 (3')	δ 70.2 (3')	δ 130.8 (10')	δ 130.8 (10')
δ 93.4 (4)	δ 93.3 (4)	δ 131.1 (8')	δ 131.1 (8')
δ 94.0 (4')	δ 93.9 (4')	δ 140.9 (6)	δ 140.9 (6)
δ 113.0 (10)	δ 112.9 (10)	δ 150.8 (6')	δ 150.7 (6')
δ 119.3 (5')	δ 119.2 (5')	δ 157.5 (9)	δ 157.4 (9)
δ 120.1 (5)	δ 120.1 (5)	δ 193.6 (1')	δ 193.6 (1')
δ 120.4 (9')	δ 120.4 (9')	δ 202.0 (1)	δ 202.0 (1)

A. L. Anaya *et al., Phytochemistry* **2008**, *69*, 1185.

^1H NMR (600 MHz, $CDCl_3$)

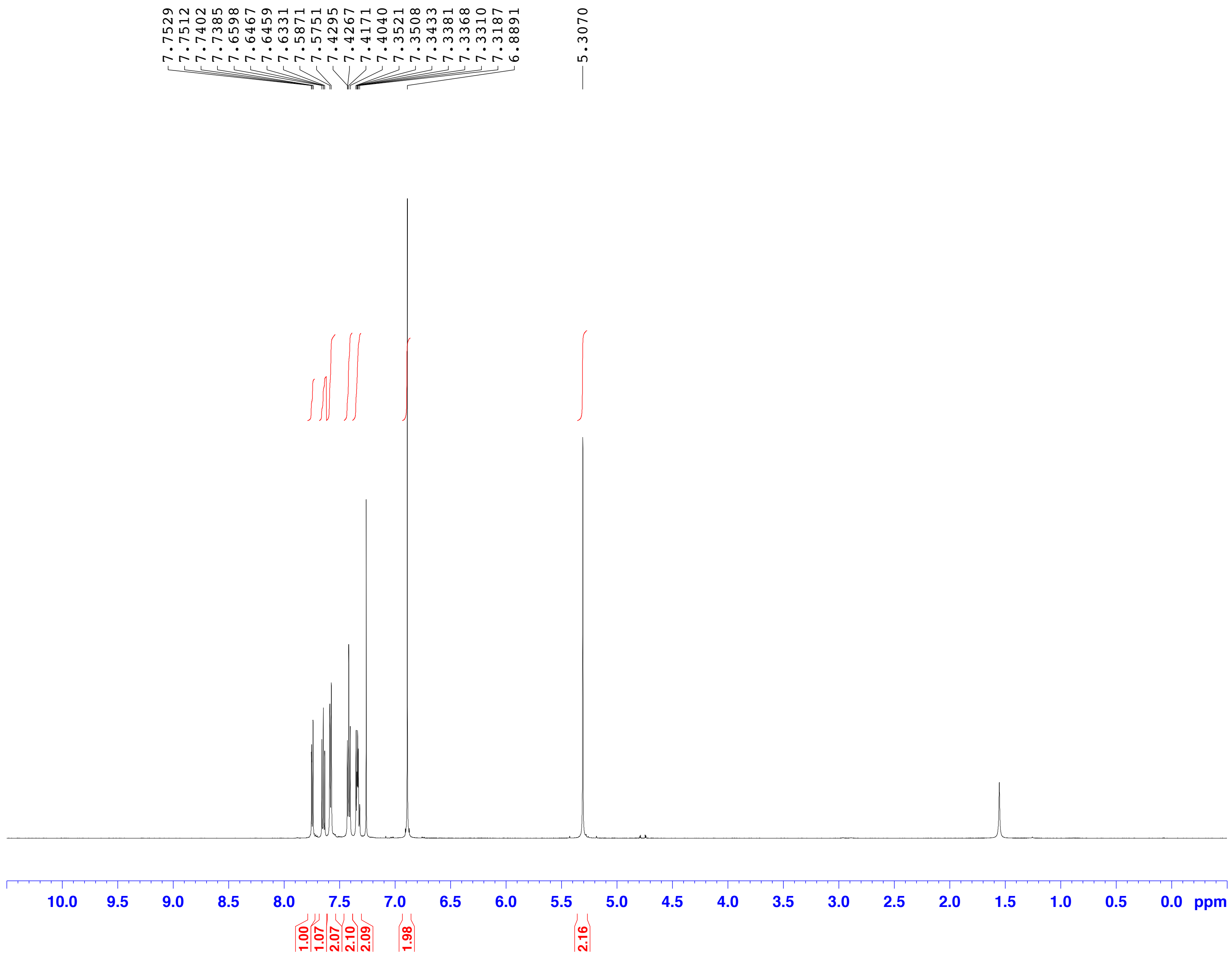


```
Current Data Parameters
NAME             YA1-1881-1
EXPNO                    40
PROCNO                    1

F2 - Acquisition Parameters
Date_              20210114
Time                  16.46
INSTRUM               spect
PROBHD     5 mm CPPBBO BB
PULPROG                zg30
TD                    65536
SOLVENT               CDCl3
NS                       16
DS                        2
SWH              12019.230 Hz
FIDRES            0.183399 Hz
AQ               2.7262976 sec
RG                    31.94
DW                   41.600 use
DE                    10.00 use
TE                    298.2 K
D1              1.00000000 sec
TD0                       1

======== CHANNEL f1 =======
SFO1         600.1337060 MHz
NUC1                     1H
P1                    12.00 use
PLW1            21.00000000 W

F2 - Processing parameters
SI                    65536
SF           600.1300148 MHz
WDW                      EM
SSB       0
LB                     0.30 Hz
GB        0
PC                     1.00
```

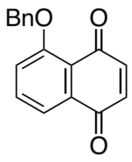

6

^{13}C NMR (150 MHz, $CDCl_3$)

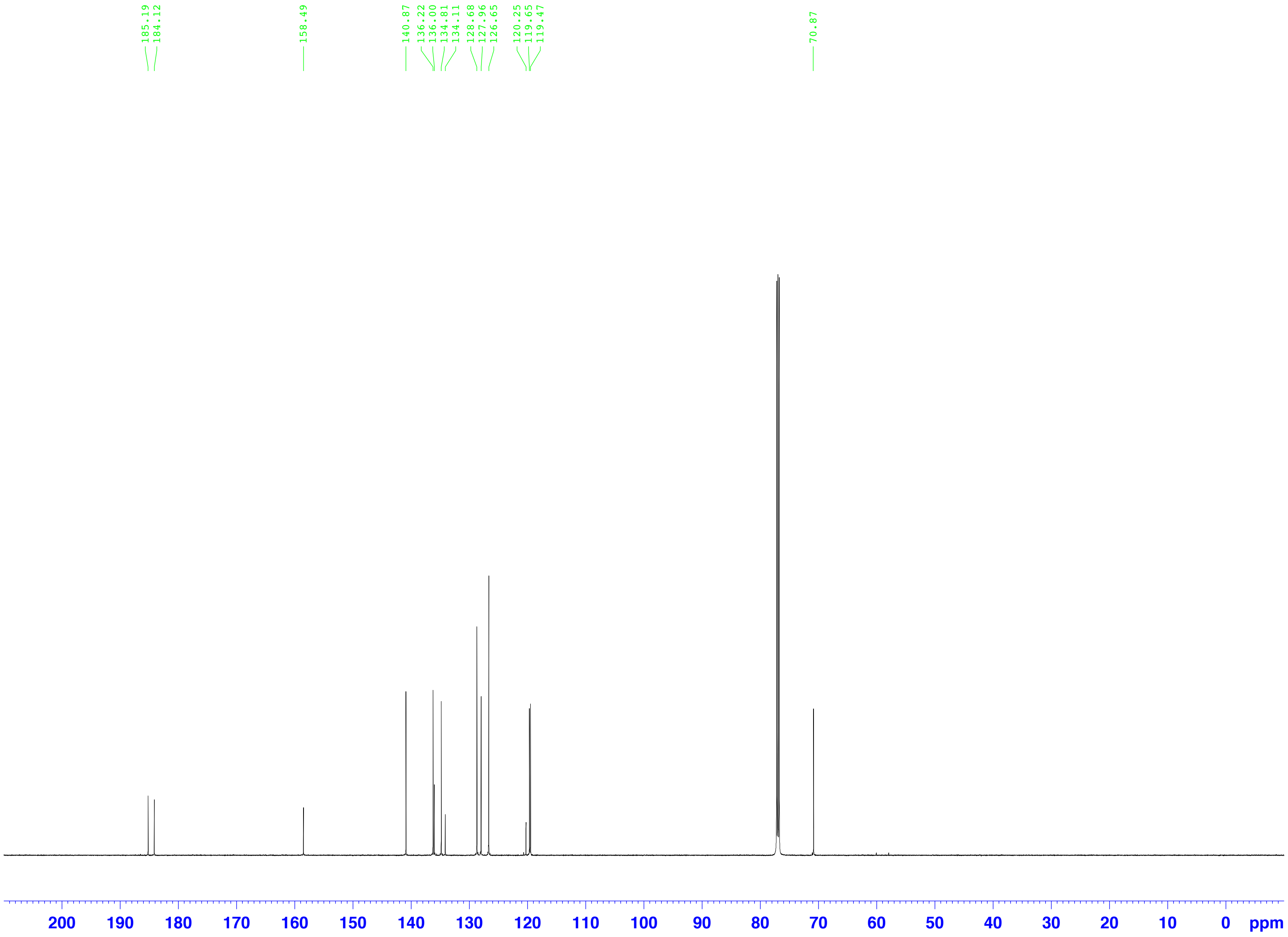


```
Current Data Parameters
NAME          YA1-1881-1
EXPNO                 11
PROCNO                 1

F2 - Acquisition Parameters
Date_           20210115
Time                0.01
INSTRUM            spect
PROBHD   5 mm CPPBBO BB
PULPROG          zgpg30
TD                 65536
SOLVENT            CDCl3
NS                  2400
DS                     4
SWH           36057.691 Hz
FIDRES         0.550197 Hz
AQ            0.9087659 sec
RG                175.56
DW                13.867 use
DE                 18.00 use
TE                 298.2 K
D1            2.00000000 sec
D11           0.03000000 sec
TD0                    1

======== CHANNEL f1 =======
SFO1        150.9178981 MHz
NUC1                 13C
P1                 10.00 use
PLW1         80.00000000 W

======== CHANNEL f2 =======
SFO2        600.1324005 MHz
NUC2                  1H
CPDPRG[2         waltz16
PCPD2              70.00 use
PLW2         13.43999958 W
PLW12         0.61714000 W
PLW13         0.31042001 W

F2 - Processing parameters
SI                 32768
SF          150.9028146 MHz
WDW                   EM
SSB       0
LB                  1.00 Hz
GB        0
PC                  1.40
```

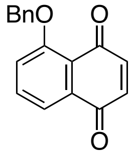

6

^{1}H NMR (600 MHz, $CDCl_3$)

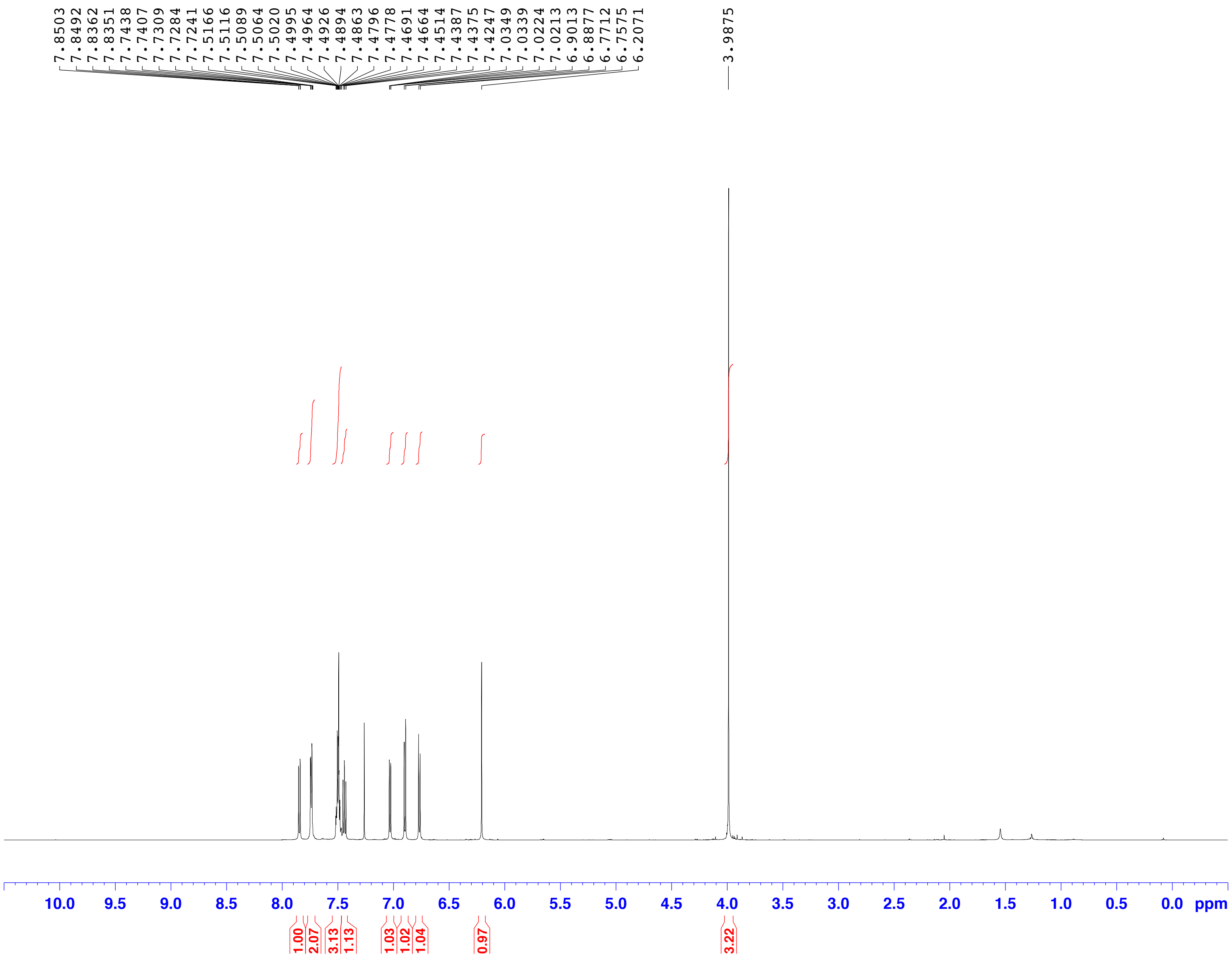

BRUKER

Current Data Parameters
NAME YA1-1883-1
EXPNO 10
PROCNO 1

F2 - Acquisition Parameters
Date_ 20210120
Time 15.57
INSTRUM spect
PROBHD 5 mm CPPBBO BB
PULPROG zg30
TD 65536
SOLVENT CDCl3
NS 16
DS 2
SWH 12019.230 Hz
FIDRES 0.183399 Hz
AQ 2.7262976 sec
RG 31.94
DW 41.600 use
DE 10.00 use
TE 298.2 K
D1 1.00000000 sec
TD0 1

======== CHANNEL f1 =======
SFO1 600.1337060 MHz
NUC1 1H
P1 12.00 use
PLW1 21.00000000 W

F2 - Processing parameters
SI 65536
SF 600.1300154 MHz
WDW EM
SSB 0
LB 0.30 Hz
GB 0
PC 1.00

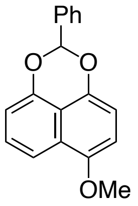

8

^{13}C NMR (150 MHz, $CDCl_3$)

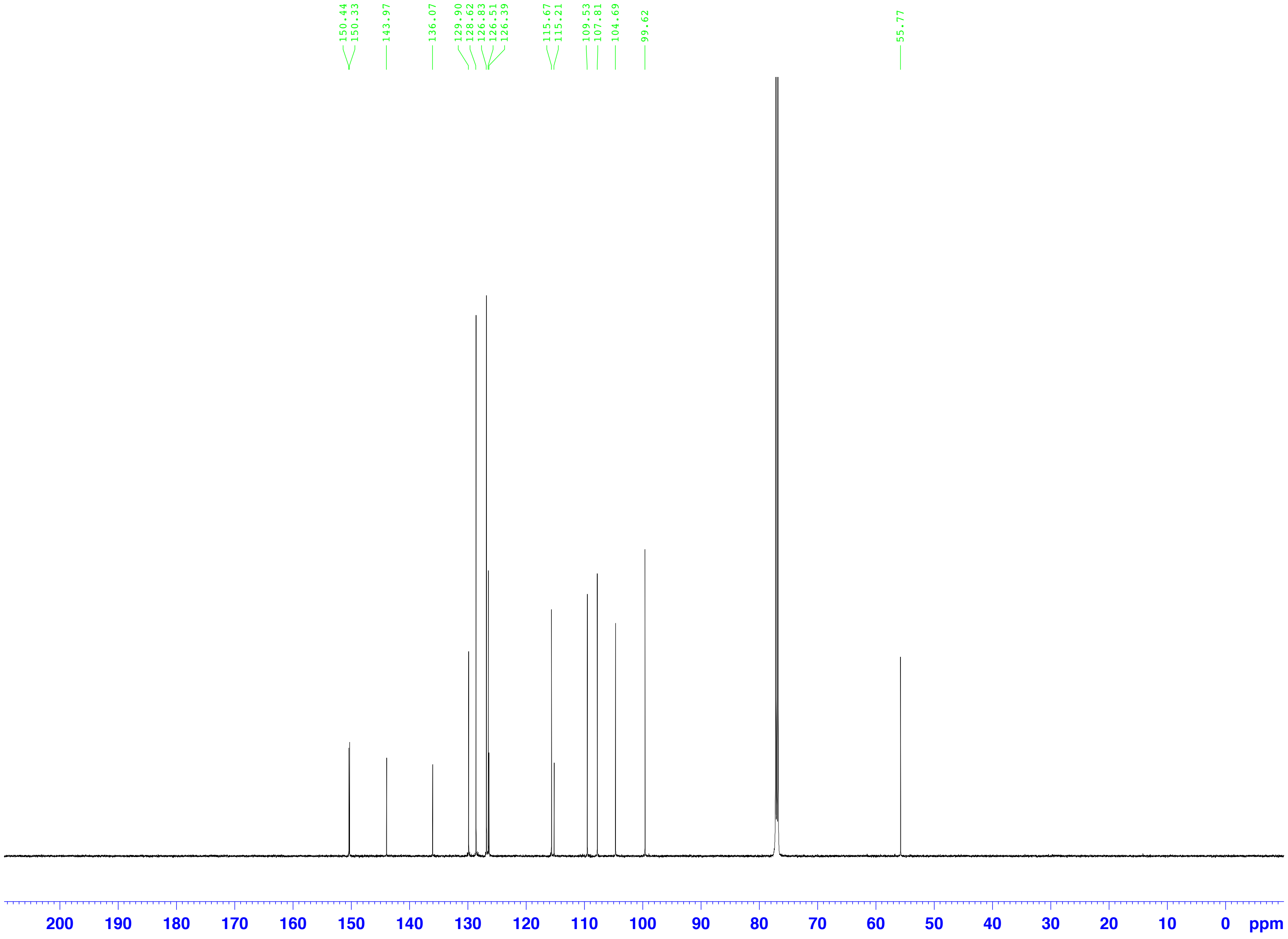


```
Current Data Parameters
NAME            YA1-1883-1
EXPNO                   11
PROCNO                   1

F2 - Acquisition Parameters
Date_             20210121
Time                 22.01
INSTRUM              spect
PROBHD    5 mm CPPBBO BB
PULPROG            zgpg30
TD                   65536
SOLVENT              CDCl3
NS                    2400
DS                       4
SWH             36057.691 Hz
FIDRES           0.550197 Hz
AQ              0.9087659 sec
RG                  175.56
DW                  13.867 use
DE                   18.00 use
TE                   298.2 K
D1             2.00000000 sec
D11            0.03000000 sec
TD0                      1

======== CHANNEL f1 =======
SFO1          150.9178981 MHz
NUC1                  13C
P1                  10.00 use
PLW1          80.00000000 W

======== CHANNEL f2 =======
SFO2          600.1324005 MHz
NUC2                   1H
CPDPRG[2          waltz16
PCPD2               70.00 use
PLW2          13.43999958 W
PLW12          0.61714000 W
PLW13          0.31042001 W

F2 - Processing parameters
SI                   32768
SF             150.9028135 MHz
WDW                     EM
SSB       0
LB                    1.00 Hz
GB        0
PC                    1.40
```

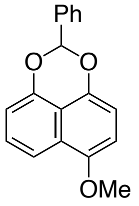

8

^{1}H NMR (600 MHz, $CDCl_3$)

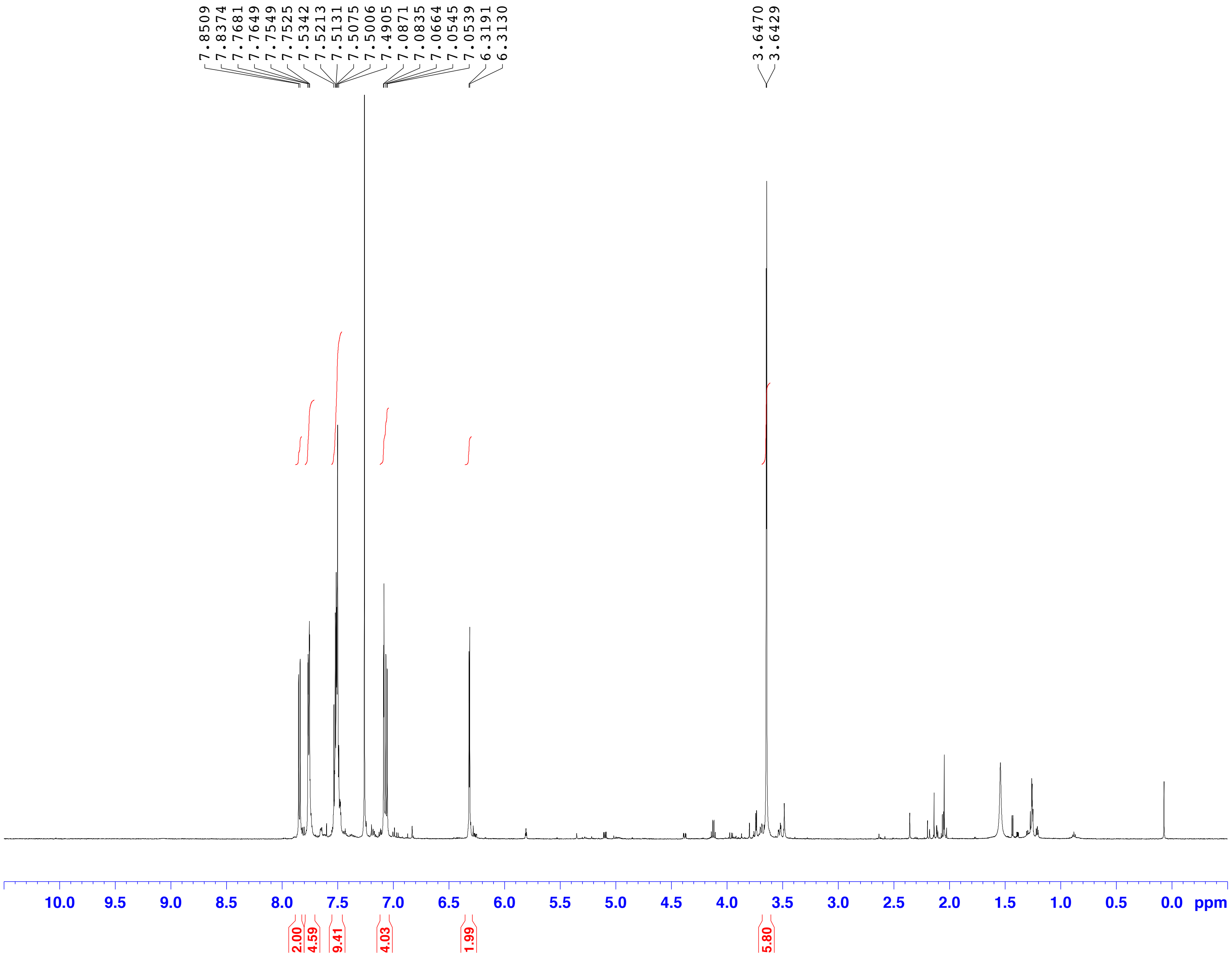

BRUKER

Current Data Parameters
NAME YA1-1883-2
EXPNO 10
PROCNO 1

F2 - Acquisition Parameters
Date_ 20210120
Time 16.00
INSTRUM spect
PROBHD 5 mm CPPBBO BB
PULPROG zg30
TD 65536
SOLVENT CDCl3
NS 16
DS 2
SWH 12019.230 Hz
FIDRES 0.183399 Hz
AQ 2.7262976 sec
RG 31.94
DW 41.600 use
DE 10.00 use
TE 298.2 K
D1 1.00000000 sec
TD0 1

======== CHANNEL f1 =======
SFO1 600.1337060 MHz
NUC1 1H
P1 12.00 use
PLW1 21.00000000 W

F2 - Processing parameters
SI 65536
SF 600.1300150 MHz
WDW EM
SSB 0
LB 0.30 Hz
GB 0
PC 1.00

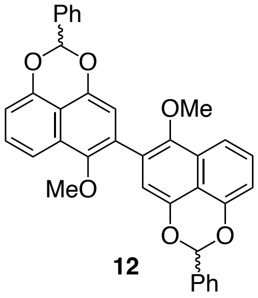

12

^{13}C NMR (150 MHz, $CDCl_3$)

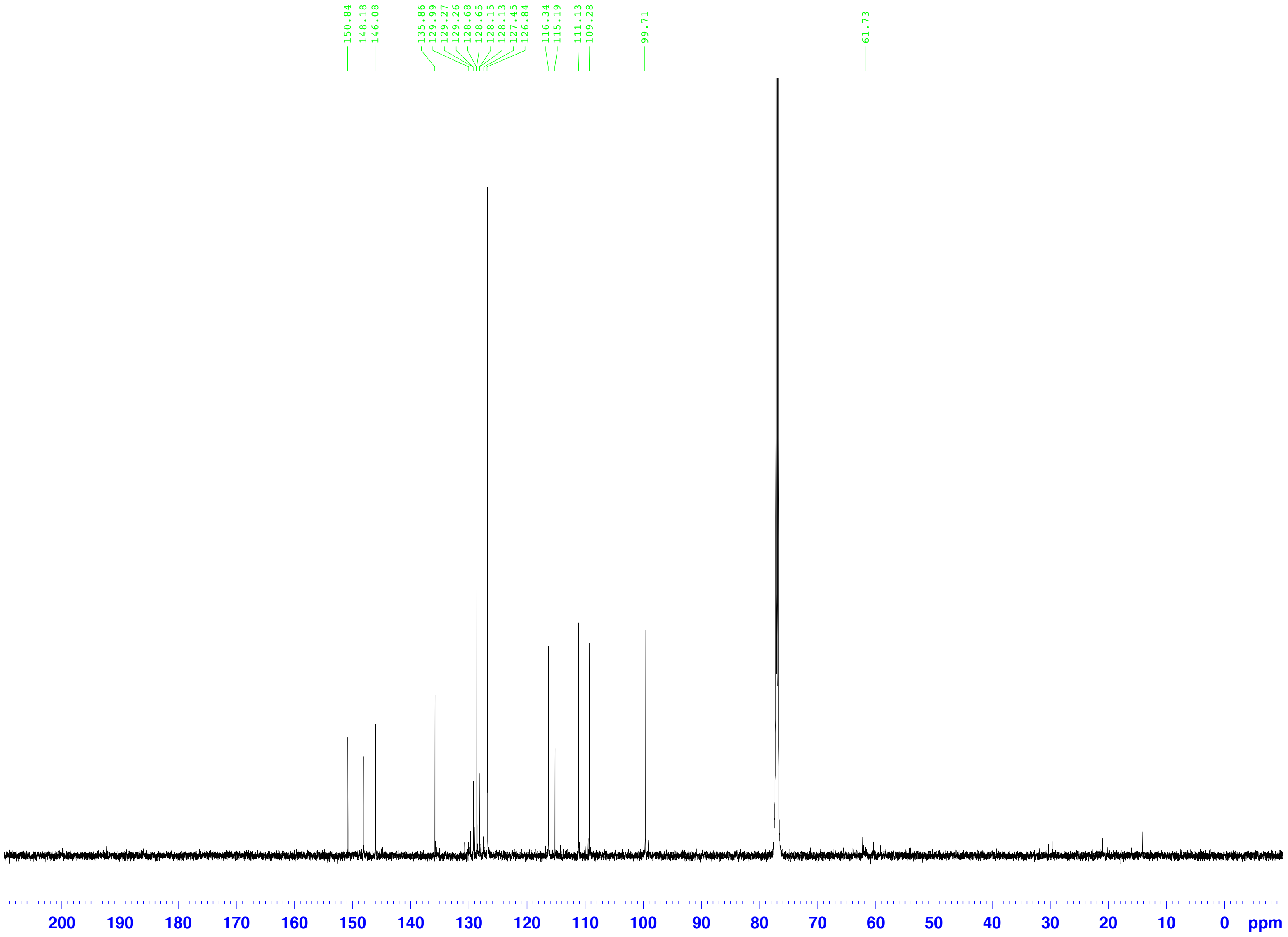


```
Current Data Parameters
NAME               YA1-1883-2
EXPNO                      11
PROCNO                      1

F2 - Acquisition Parameters
Date_                20210122
Time                     2.41
INSTRUM                 spect
PROBHD     5 mm CPPBBO BB
PULPROG               zgpg30
TD                      65536
SOLVENT                 CDCl3
NS                       3600
DS                          4
SWH                 36057.691 Hz
FIDRES               0.550197 Hz
AQ                  0.9087659 sec
RG                     175.56
DW                     13.867 use
DE                      18.00 use
TE                      298.2 K
D1                 2.00000000 sec
D11                0.03000000 sec
TD0                         1

======== CHANNEL f1 =======
SFO1              150.9178981 MHz
NUC1                      13C
P1                      10.00 use
PLW1              80.00000000 W

======== CHANNEL f2 =======
SFO2              600.1324005 MHz
NUC2                       1H
CPDPRG[2              waltz16
PCPD2                   70.00 use
PLW2              13.43999958 W
PLW12              0.61714000 W
PLW13              0.31042001 W

F2 - Processing parameters
SI                      32768
SF                150.9028123 MHz
WDW                        EM
SSB       0
LB                       1.00 Hz
GB        0
PC                       1.40
```

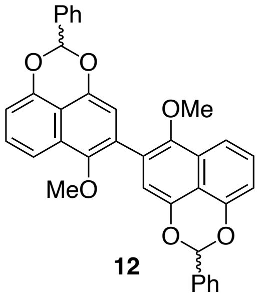

12

HSQC ($CDCl_3$)

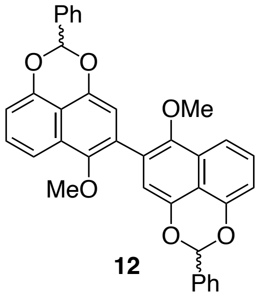

12

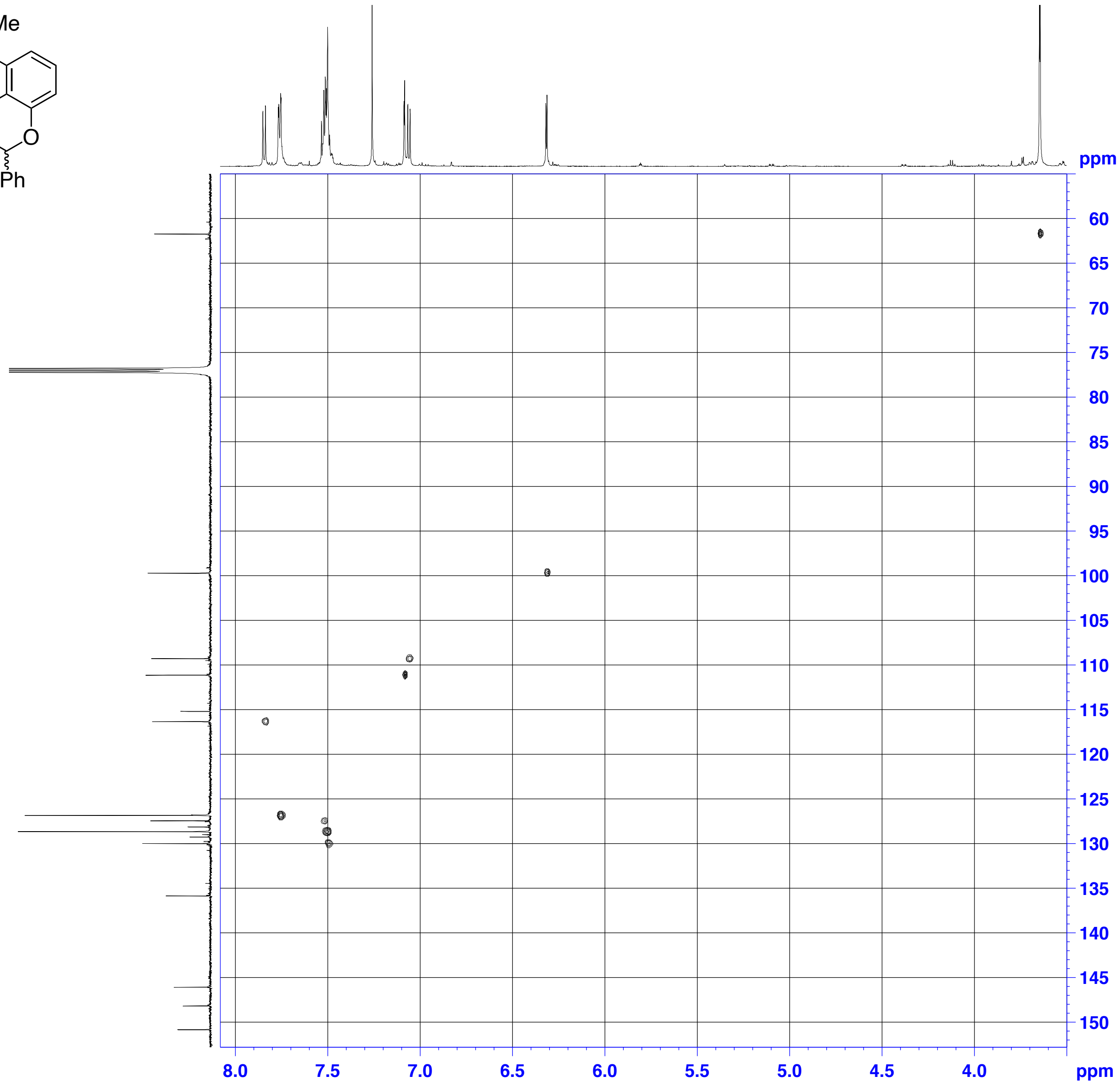


```
Current Data Parameters
NAME          YA1-1883-2
EXPNO                 13
PROCNO                 1

F2 - Acquisition Parameters
Date_           20210122
Time                2.58
INSTRUM            spect
PROBHD   5 mm CPPBBO BB
PULPROG  hsqcedetgpsisp2.3
TD                  2048
SOLVENT            CDCl3
NS                     4
DS                    32
SWH             7211.539 Hz
FIDRES          3.521259 Hz
AQ             0.1419947 sec
RG                 61.57
DW                69.333 usec
DE                 10.00 usec
TE                 298.2 K
CNST2        145.0000000
CNST17        -0.5000000
D0            0.00000300 sec
D1            2.00000000 sec
D4            0.00172414 sec
D11           0.03000000 sec
D16           0.00020000 sec
D21           0.00360000 sec
D24           0.00089000 sec
IN0           0.00002000 sec

======== CHANNEL f1 ========
SFO1         600.1328224 MHz
NUC1                  1H
P1                 12.00 usec
P2                 24.00 usec
P28      0 usec
PLW1         21.00000000 W

======== CHANNEL f2 ========
SFO2         150.9133710 MHz
NUC2                 13C
CPDPRG[2            garp
P3                 10.00 usec
P14               500.00 usec
P24              2000.00 usec
P31              1730.00 usec
PCPD2              60.00 usec
PLW0     0 W
PLW2         80.00000000 W
PLW12         2.22219992 W
SPNAM[3] Crp60,0.5,20.1
SPOAL3             0.500
SPOFFS3  0 Hz
SPW3         12.22299957 W
SPNAM[7]    Crp60comp.4
SPOAL7             0.500
SPOFFS7  0 Hz
SPW7         12.22299957 W
SPNAM[18  Crp60_xfilt.2
SPOAL18            0.500
SPOFFS18 0 Hz
SPW18         3.53270006 W

====== GRADIENT CHANNEL =====
GPNAM[1]     SMSQ10.100
GPNAM[2]     SMSQ10.100
GPNAM[3]     SMSQ10.100
GPNAM[4]     SMSQ10.100
GPZ1               80.00 %
GPZ2               20.10 %
GPZ3               11.00 %
GPZ4               -5.00 %
P16              1000.00 usec
P19               600.00 usec

F1 - Acquisition parameters
TD                   256
SFO1            150.9134 MHz
FIDRES         97.656250 Hz
SW               165.658 ppm
FnMODE     Echo-Antiecho

F2 - Processing parameters
SI                  1024
SF           600.1300150 MHz
WDW                QSINE
SSB                    2
LB       0 Hz
GB       0
PC                  1.40

F1 - Processing parameters
SI                  1024
MC2        echo-antiecho
SF           150.9028123 MHz
WDW                QSINE
SSB                    2
LB       0 Hz
GB       0
```

HMBC ($CDCl_3$)

key HMBC correlations

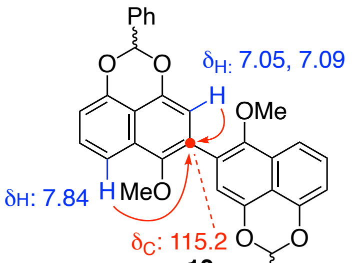

12

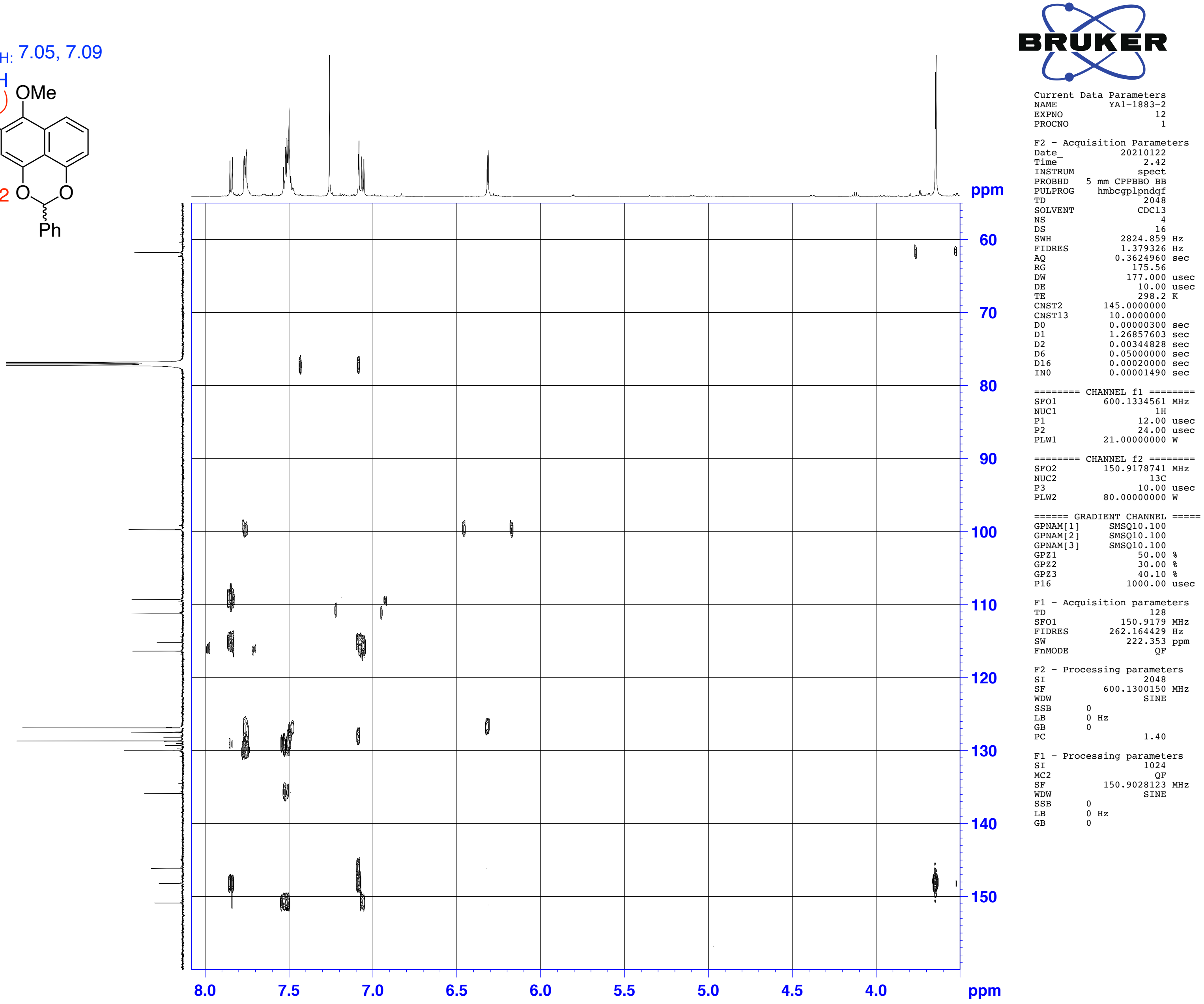

^{1}H NMR (600 MHz, $CDCl_3$)

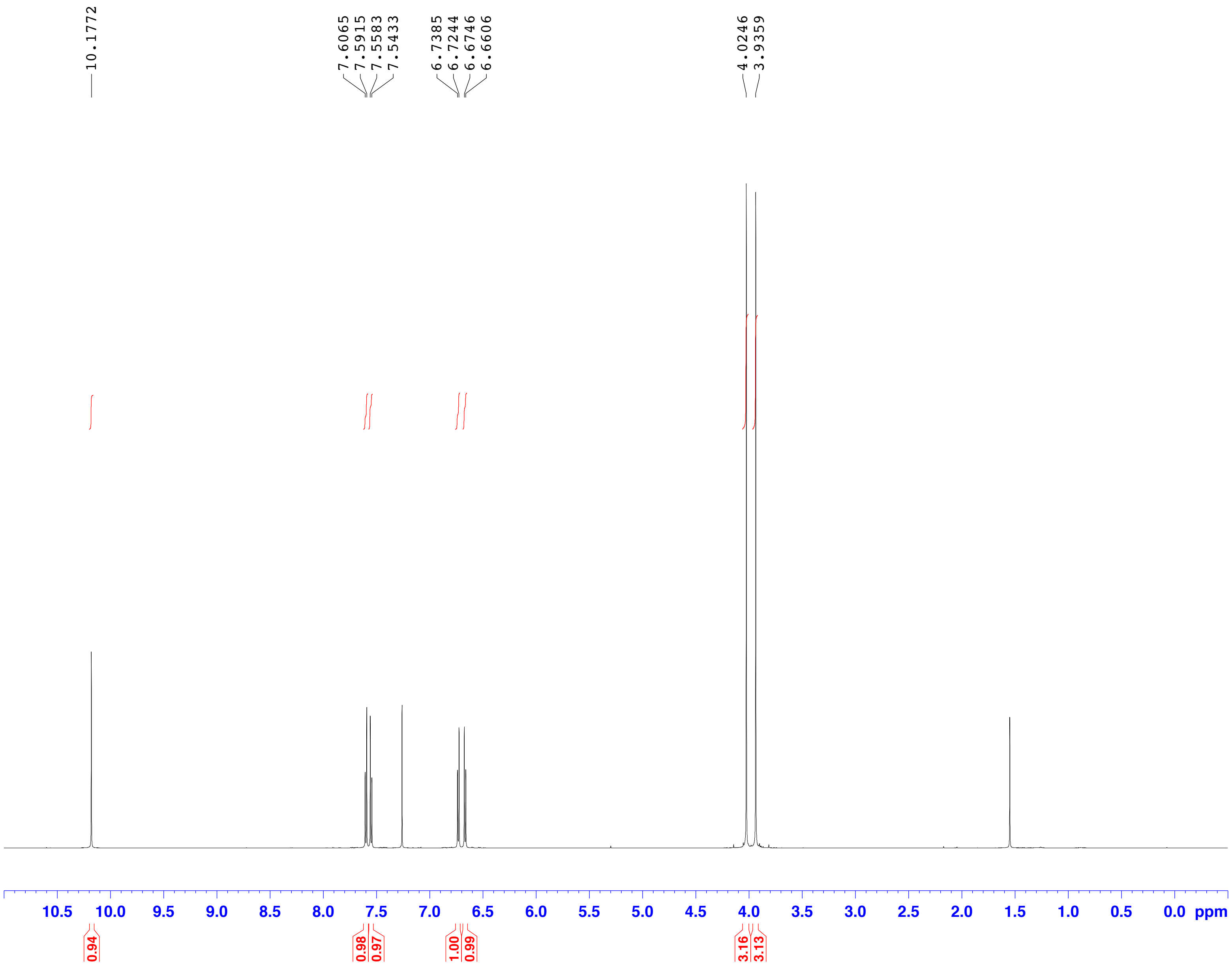


```
Current Data Parameters
NAME              YA1-1737-3
EXPNO                     20
PROCNO                     1

F2 - Acquisition Parameters
Date_               20191225
Time                   17.59
INSTRUM                spect
PROBHD    5 mm CPPBBO BB
PULPROG                 zg30
TD                     65536
SOLVENT                CDCl3
NS                        16
DS                         2
SWH                12019.230 Hz
FIDRES              0.183399 Hz
AQ                 2.7262976 sec
RG                     31.94
DW                    41.600 use
DE                     10.00 use
TE                     298.2 K
D1                1.00000000 sec
TD0                        1

======== CHANNEL f1 =======
SFO1             600.1337060 MHz
NUC1                      1H
P1                     12.00 use
PLW1             21.00000000 W

F2 - Processing parameters
SI                     65536
SF               600.1300147 MHz
WDW                       EM
SSB       0
LB                      0.30 Hz
GB        0
PC                      1.00
```

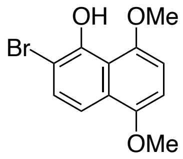

19

^{13}C NMR (150 MHz, $CDCl_3$)

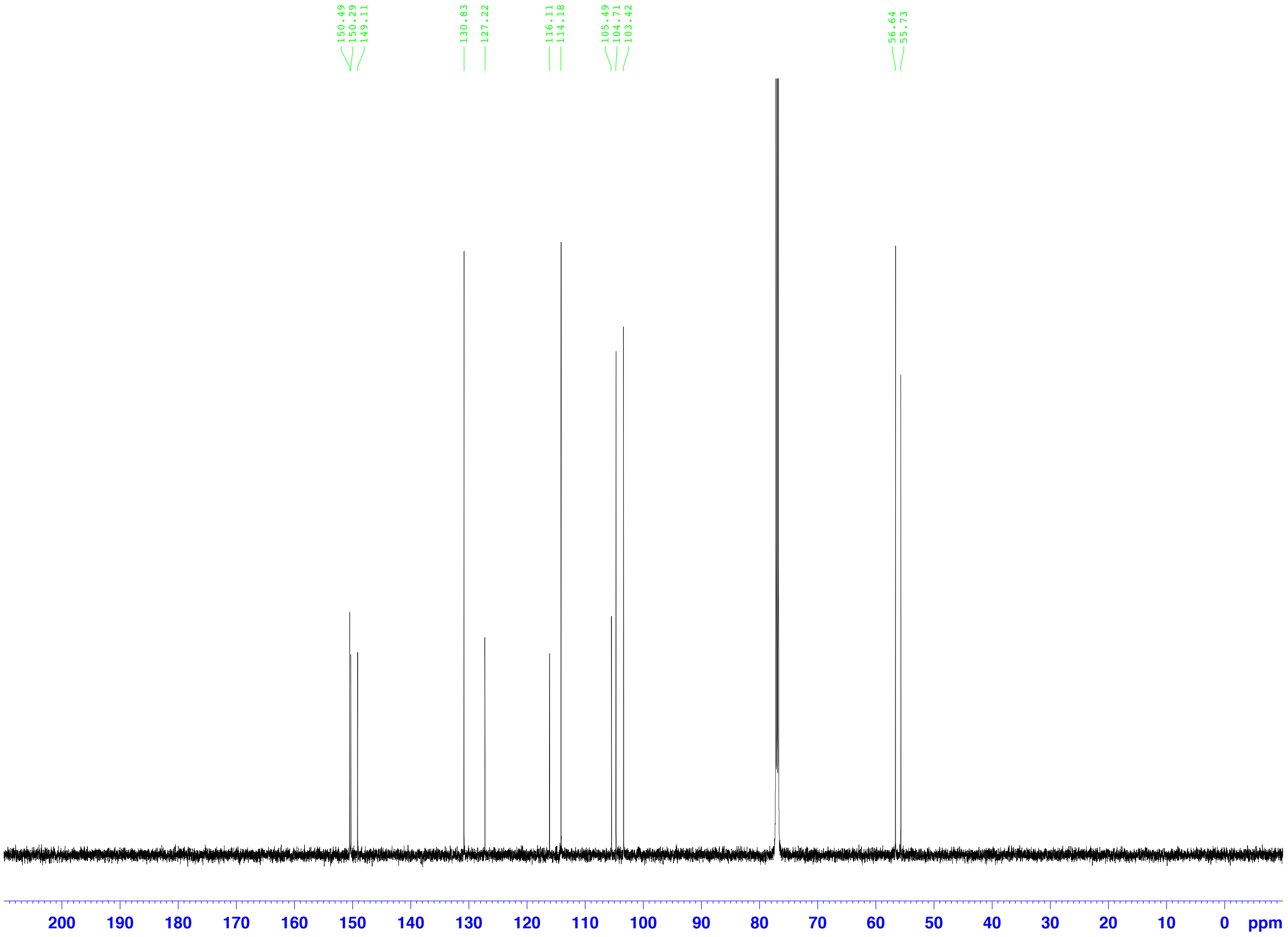

BRUKER

```
Current Data Parameters
NAME               YA1-1737-3
EXPNO                      21
PROCNO                      1

F2 - Acquisition Parameters
Date_              20191226
Time                   0.53
INSTRUM               spect
PROBHD   5 mm CPPBBO BB
PULPROG             zgpg30
TD                   65536
SOLVENT              CDCl3
NS                    1024
DS                       4
SWH             36057.691 Hz
FIDRES           0.550197 Hz
AQ              0.9087659 sec
RG                  175.56
DW                  13.867 use
DE                   18.00 use
TE                   298.2 K
D1              2.00000000 sec
D11             0.03000000 sec
TD0                      1

======== CHANNEL f1 =======
SFO1          150.9178981 MHz
NUC1                  13C
P1                  10.00 use
PLW1          80.00000000 W

======== CHANNEL f2 =======
SFO2          600.1324005 MHz
NUC2                   1H
CPDPRG[2          waltz16
PCPD2               70.00 use
PLW2          13.43999958 W
PLW12          0.61714000 W
PLW13          0.31042001 W

F2 - Processing parameters
SI                  32768
SF            150.9028135 MHz
WDW                    EM
SSB      0
LB                   1.00 Hz
GB       0
PC                   1.40
```

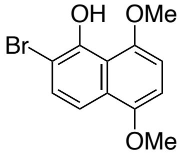

19

^{1}H NMR (600 MHz, $CDCl_3$)

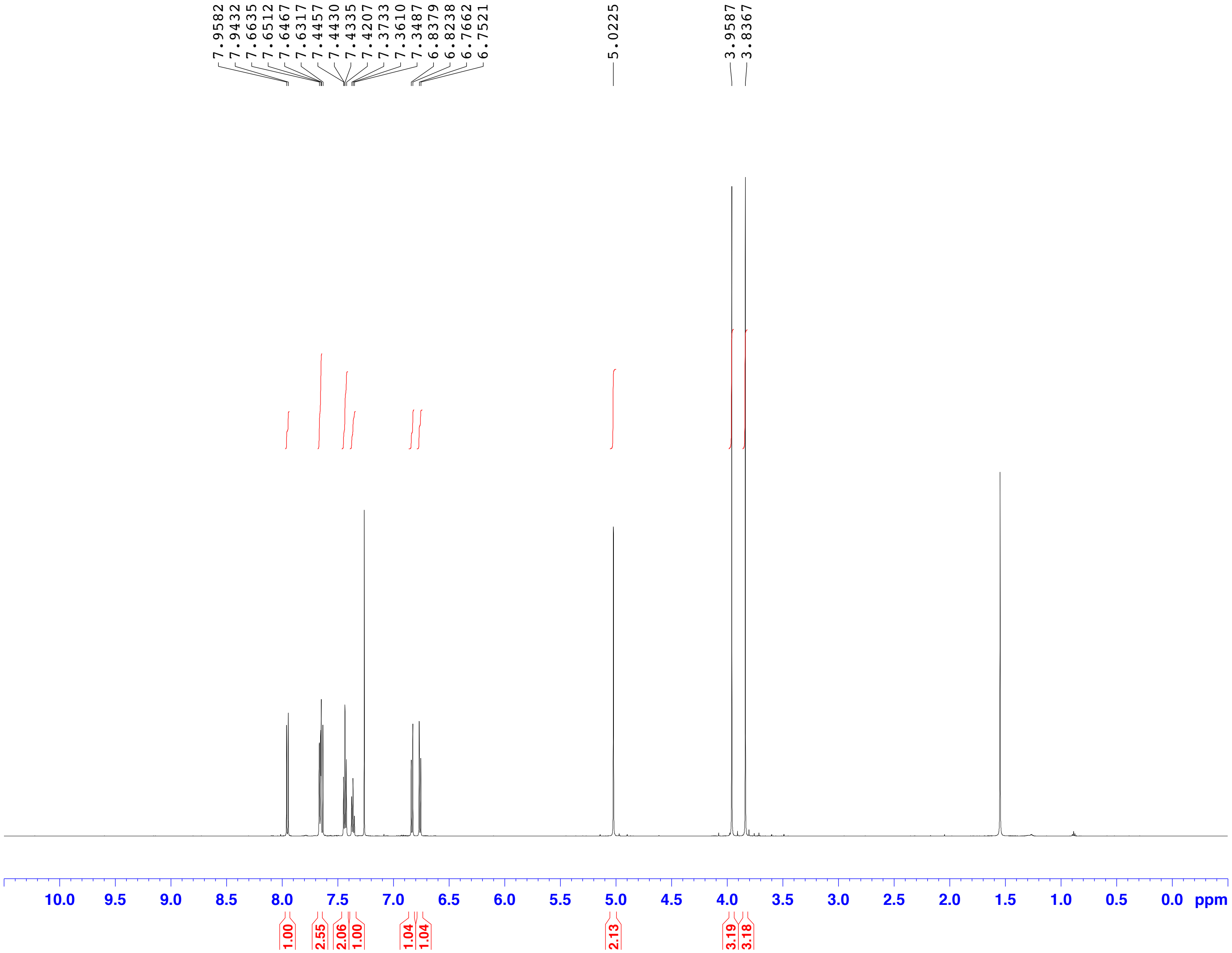

BRUKER

```
Current Data Parameters
NAME              YA1-1555-2
EXPNO                     10
PROCNO                     1

F2 - Acquisition Parameters
Date_               20171118
Time                   17.11
INSTRUM                spect
PROBHD    5 mm CPPBBO BB
PULPROG                 zg30
TD                     65536
SOLVENT                CDCl3
NS                        16
DS                         2
SWH             12019.230 Hz
FIDRES           0.183399 Hz
AQ              2.7262976 sec
RG                      17.5
DW                  41.600 use
DE                   10.00 use
TE                   300.1 K
D1             1.00000000 sec
TD0                        1

======== CHANNEL f1 =======
SFO1          600.1337060 MHz
NUC1                      1H
P1                   12.00 use
PLW1           23.00000000 W

F2 - Processing parameters
SI                     65536
SF            600.1300143 MHz
WDW                       EM
SSB       0
LB                    0.30 Hz
GB        0
PC                      1.00
```

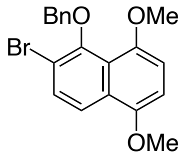

13a

^{13}C NMR (150 MHz, $CDCl_3$)

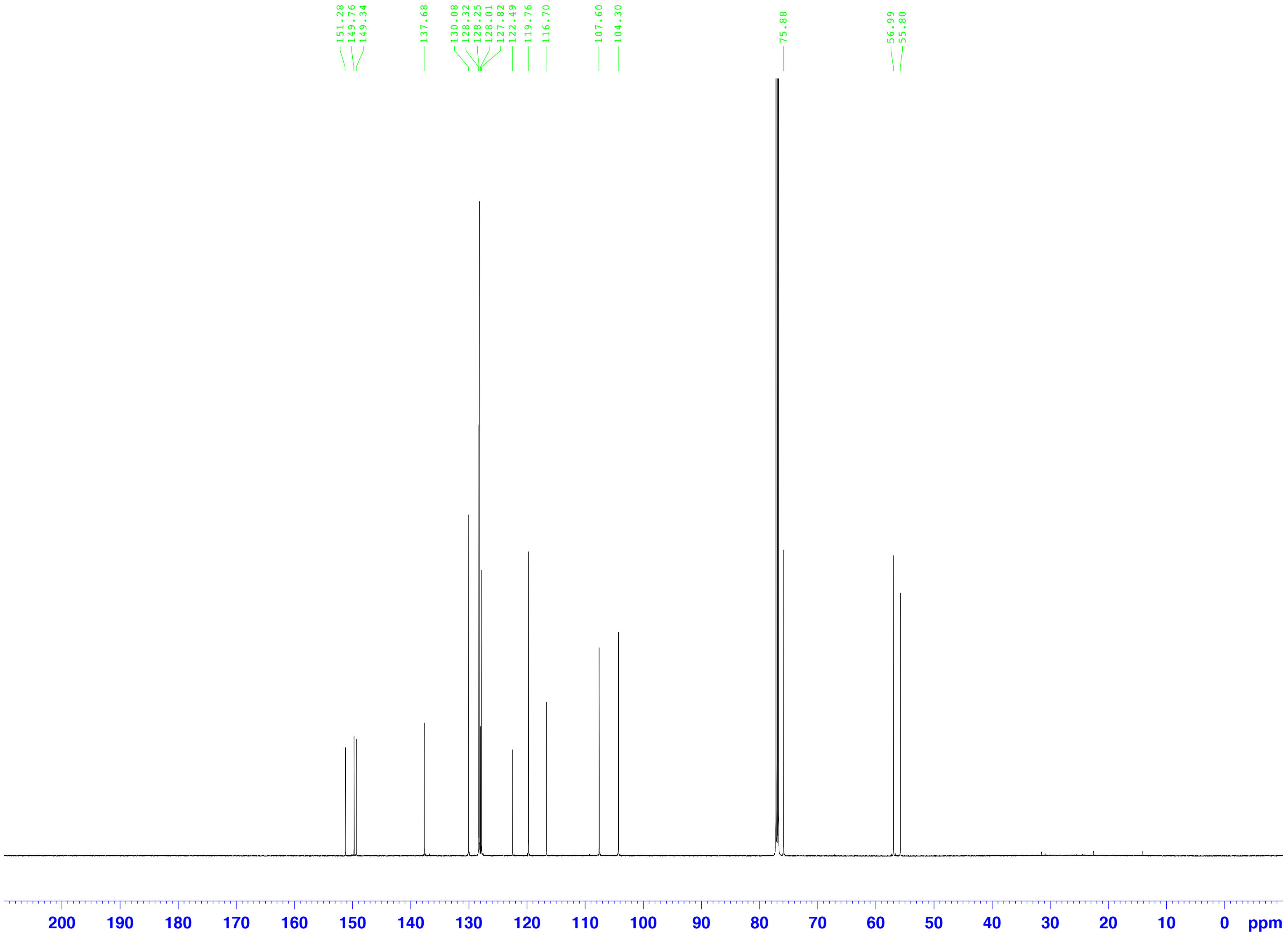


```
Current Data Parameters
NAME          YA1-1553-13C
EXPNO                   11
PROCNO                   1

F2 - Acquisition Parameters
Date_             20171121
Time                  3.00
INSTRUM              spect
PROBHD     5 mm CPPBBO BB
PULPROG            zgpg30
TD                   65536
SOLVENT              CDCl3
NS                    3600
DS                       4
SWH              36057.691 Hz
FIDRES            0.550197 Hz
AQ               0.9087659 sec
RG                  175.56
DW                  13.867 use
DE                   18.00 use
TE                   300.0 K
D1              2.00000000 sec
D11             0.03000000 sec
TD0                      1

======== CHANNEL f1 =======
SFO1           150.9178981 MHz
NUC1                   13C
P1                   10.00 use
PLW1           70.00000000 W

======== CHANNEL f2 =======
SFO2           600.1324005 MHz
NUC2                    1H
CPDPRG[2           waltz16
PCPD2                70.00 use
PLW2           14.00000000 W
PLW12           0.64286000 W
PLW13           0.32335001 W

F2 - Processing parameters
SI                   32768
SF             150.9028156 MHz
WDW                     EM
SSB      0
LB                    1.00 Hz
GB       0
PC                    1.40
```

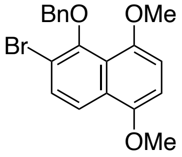

13a

^{1}H NMR (600 MHz, $CDCl_3$)

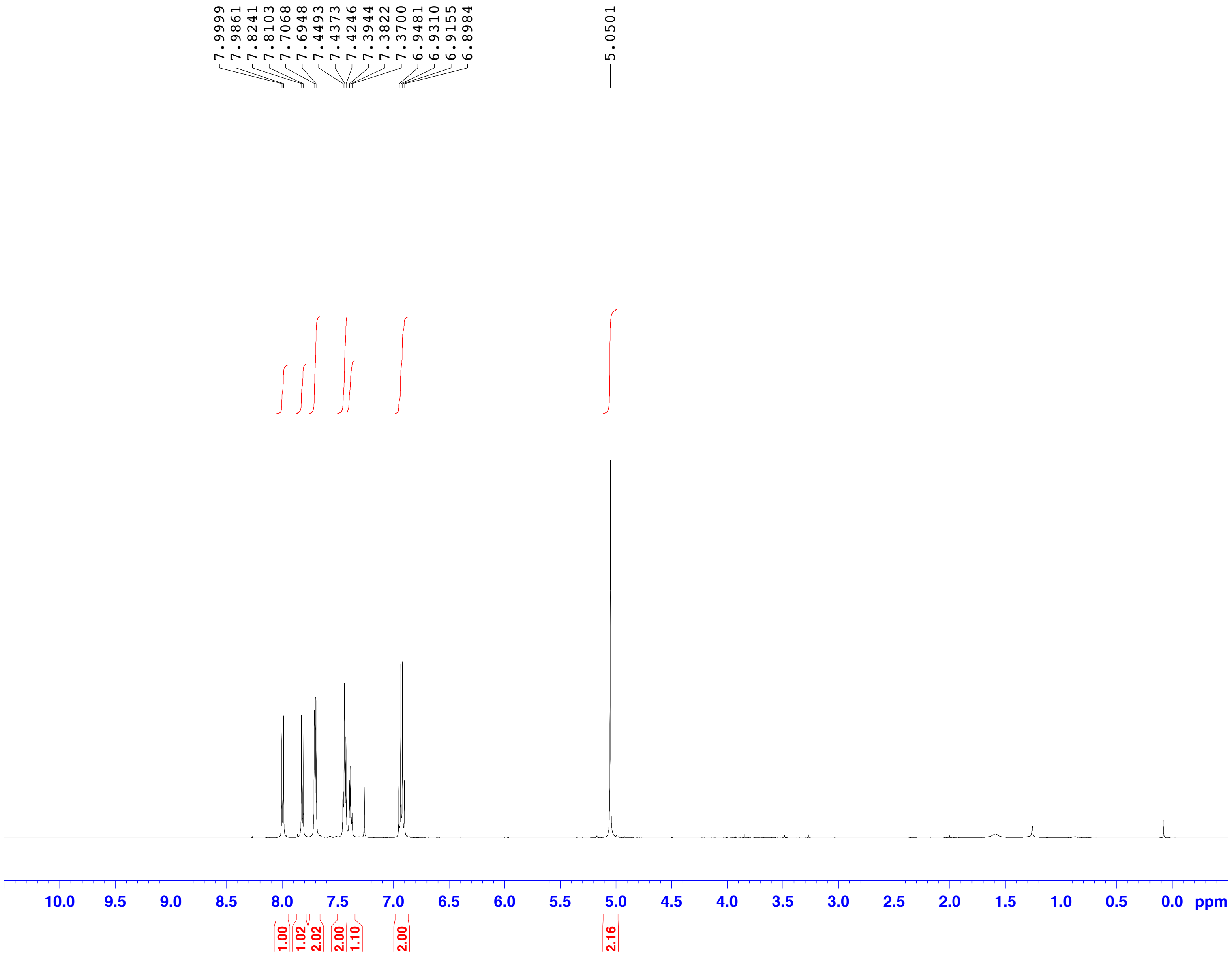


```
Current Data Parameters
NAME      YA1-1877-crude
EXPNO                 10
PROCNO                 1

F2 - Acquisition Parameters
Date_           20210106
Time               20.38
INSTRUM            spect
PROBHD    5 mm CPPBBO BB
PULPROG             zg30
TD                 65536
SOLVENT            CDCl3
NS                    16
DS                     2
SWH            12019.230 Hz
FIDRES          0.183399 Hz
AQ             2.7262976 sec
RG                 31.94
DW                41.600 use
DE                 10.00 use
TE                 298.2 K
D1            1.00000000 sec
TD0                    1

======== CHANNEL f1 =======
SFO1         600.1337060 MHz
NUC1                  1H
P1                 12.00 use
PLW1         21.00000000 W

F2 - Processing parameters
SI                 65536
SF           600.1300148 MHz
WDW                   EM
SSB       0
LB                  0.30 Hz
GB        0
PC                  1.00
```

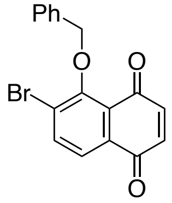

9a
(crude)

^{13}C NMR (150 MHz, $CDCl_3$)

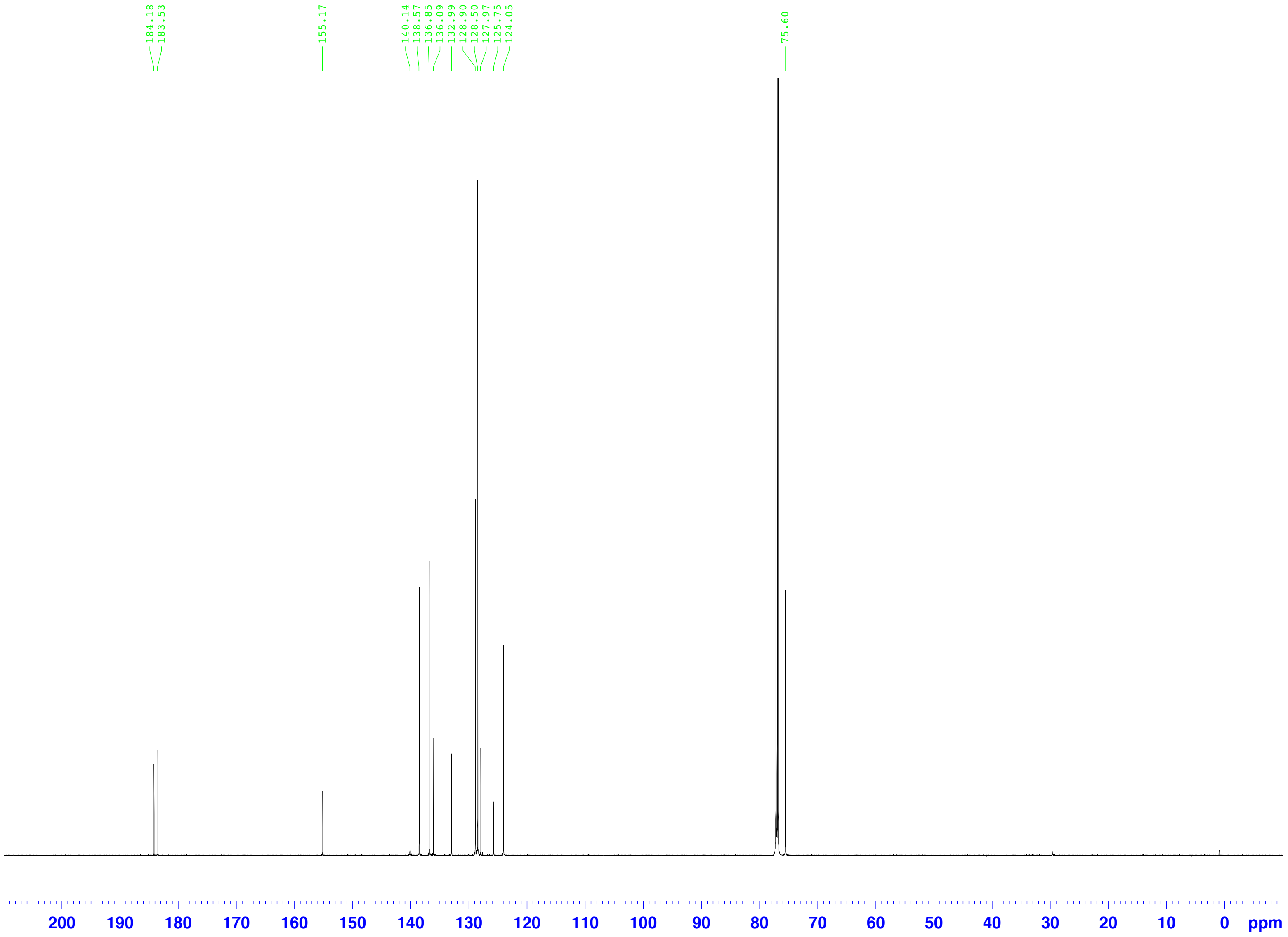


```
Current Data Parameters
NAME      YA1-1877-crude
EXPNO                 11
PROCNO                 1

F2 - Acquisition Parameters
Date_           20210107
Time                1.01
INSTRUM            spect
PROBHD   5 mm CPPBBO BB
PULPROG          zgpg30
TD                 65536
SOLVENT            CDCl3
NS                  2400
DS                     4
SWH           36057.691 Hz
FIDRES         0.550197 Hz
AQ            0.9087659 sec
RG                175.56
DW                13.867 use
DE                 18.00 use
TE                 298.2 K
D1            2.00000000 sec
D11           0.03000000 sec
TD0                    1

======== CHANNEL f1 =======
SFO1         150.9178981 MHz
NUC1                 13C
P1                 10.00 use
PLW1         80.00000000 W

======== CHANNEL f2 =======
SFO2         600.1324005 MHz
NUC2                  1H
CPDPRG[2         waltz16
PCPD2              70.00 use
PLW2         13.43999958 W
PLW12         0.61714000 W
PLW13         0.31042001 W

F2 - Processing parameters
SI                 32768
SF           150.9028156 MHz
WDW                   EM
SSB      0
LB                  1.00 Hz
GB       0
PC                  1.40
```

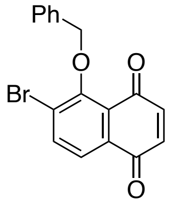

9a
(crude)

^{1}H NMR (600 MHz, $CDCl_3$)

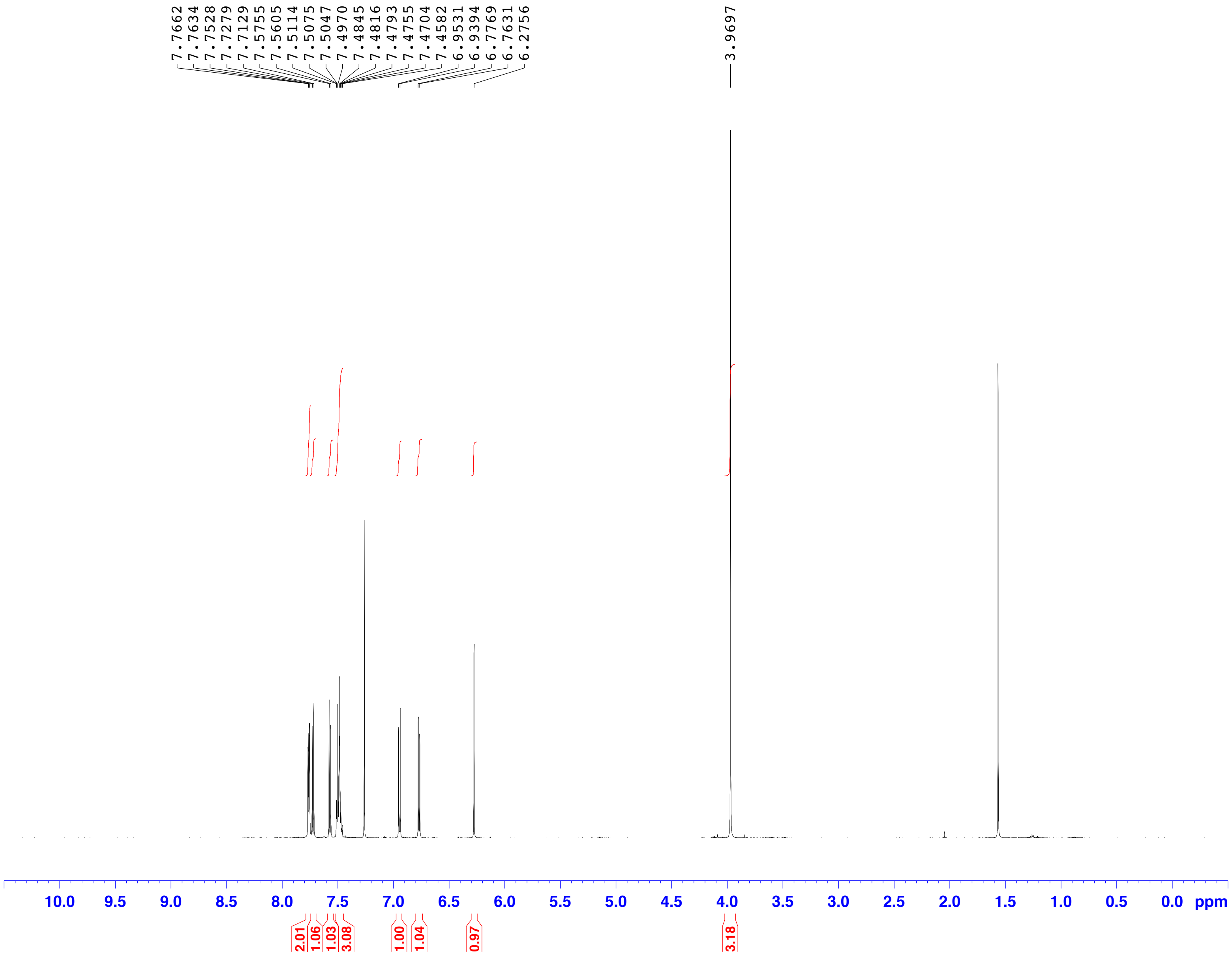

BRUKER

```
Current Data Parameters
NAME            YA1-1703-1
EXPNO                   26
PROCNO                   1

F2 - Acquisition Parameters
Date_             20191003
Time                 16.58
INSTRUM              spect
PROBHD    5 mm CPPBBO BB
PULPROG               zg30
TD                   65536
SOLVENT              CDCl3
NS                      16
DS                       2
SWH              12019.230 Hz
FIDRES            0.183399 Hz
AQ               2.7262976 sec
RG                   31.94
DW                  41.600 use
DE                   10.00 use
TE                   290.8 K
D1              1.00000000 sec
TD0                      1

======== CHANNEL f1 =======
SFO1          600.1337060 MHz
NUC1                    1H
P1                   12.00 use
PLW1           21.00000000 W

F2 - Processing parameters
SI                   65536
SF            600.1300149 MHz
WDW                     EM
SSB      0
LB                    0.30 Hz
GB       0
PC                    1.00
```

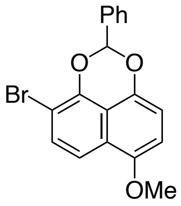

11a

^{13}C NMR (150 MHz, $CDCl_3$)

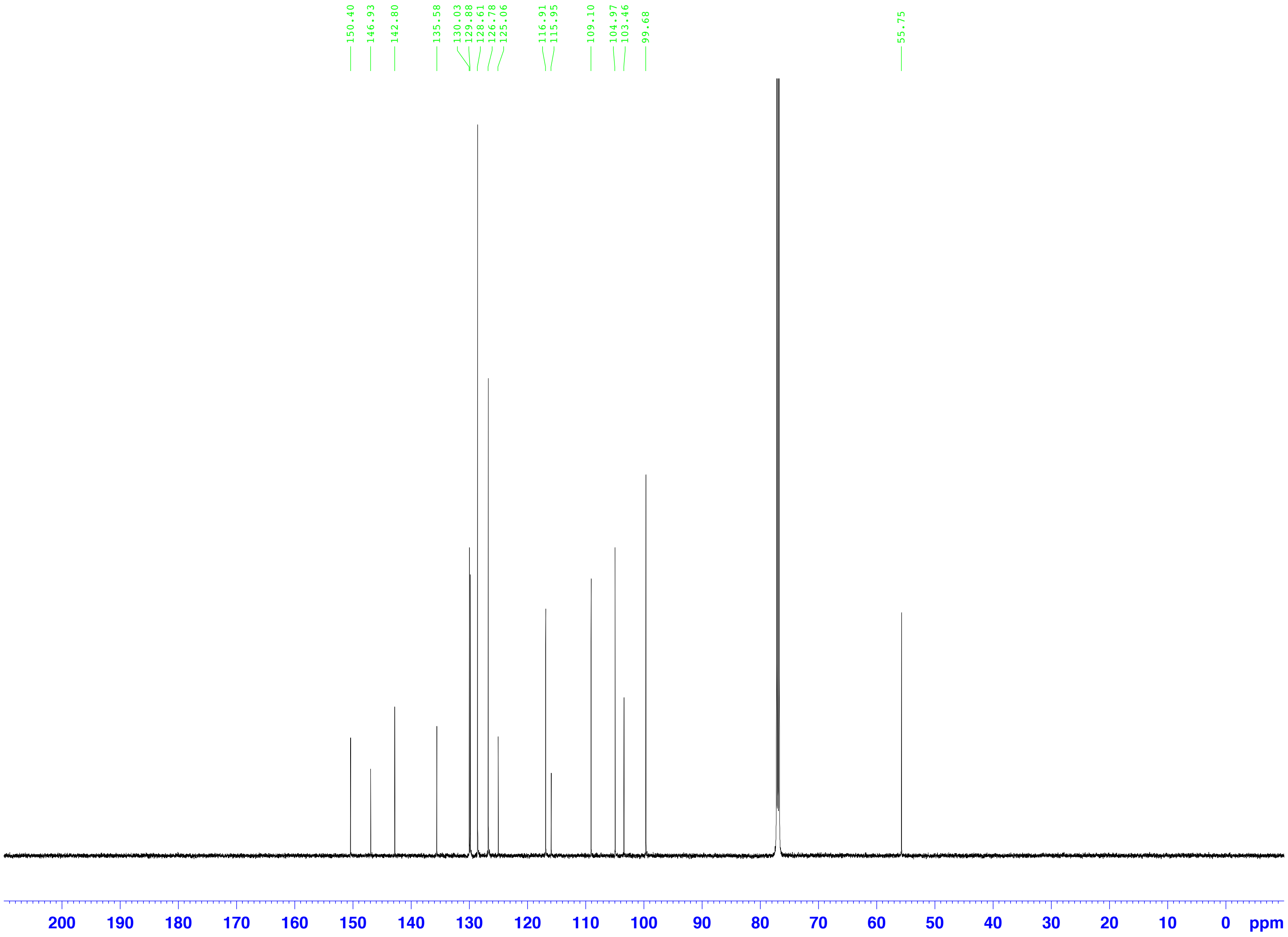


```
Current Data Parameters
NAME            YA1-1703-1
EXPNO                   22
PROCNO                   1

F2 - Acquisition Parameters
Date_             20191005
Time                  2.41
INSTRUM              spect
PROBHD     5 mm CPPBBO BB
PULPROG            zgpg30
TD                   65536
SOLVENT              CDCl3
NS                    3200
DS                       4
SWH              36057.691 Hz
FIDRES            0.550197 Hz
AQ               0.9087659 sec
RG                  175.56
DW                  13.867 use
DE                   18.00 use
TE                   290.8 K
D1              2.00000000 sec
D11             0.03000000 sec
TD0                      1

======== CHANNEL f1 =======
SFO1           150.9178981 MHz
NUC1                   13C
P1                   10.00 use
PLW1           80.00000000 W

======== CHANNEL f2 =======
SFO2           600.1324005 MHz
NUC2                    1H
CPDPRG[2           waltz16
PCPD2                70.00 use
PLW2           13.43999958 W
PLW12           0.61714000 W
PLW13           0.31042001 W

F2 - Processing parameters
SI                   32768
SF             150.9028158 MHz
WDW                     EM
SSB      0
LB                    1.00 Hz
GB       0
PC                    1.40
```

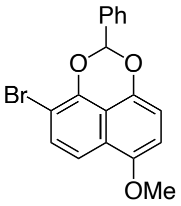

11a

^{1}H NMR (600 MHz, $CDCl_3$)

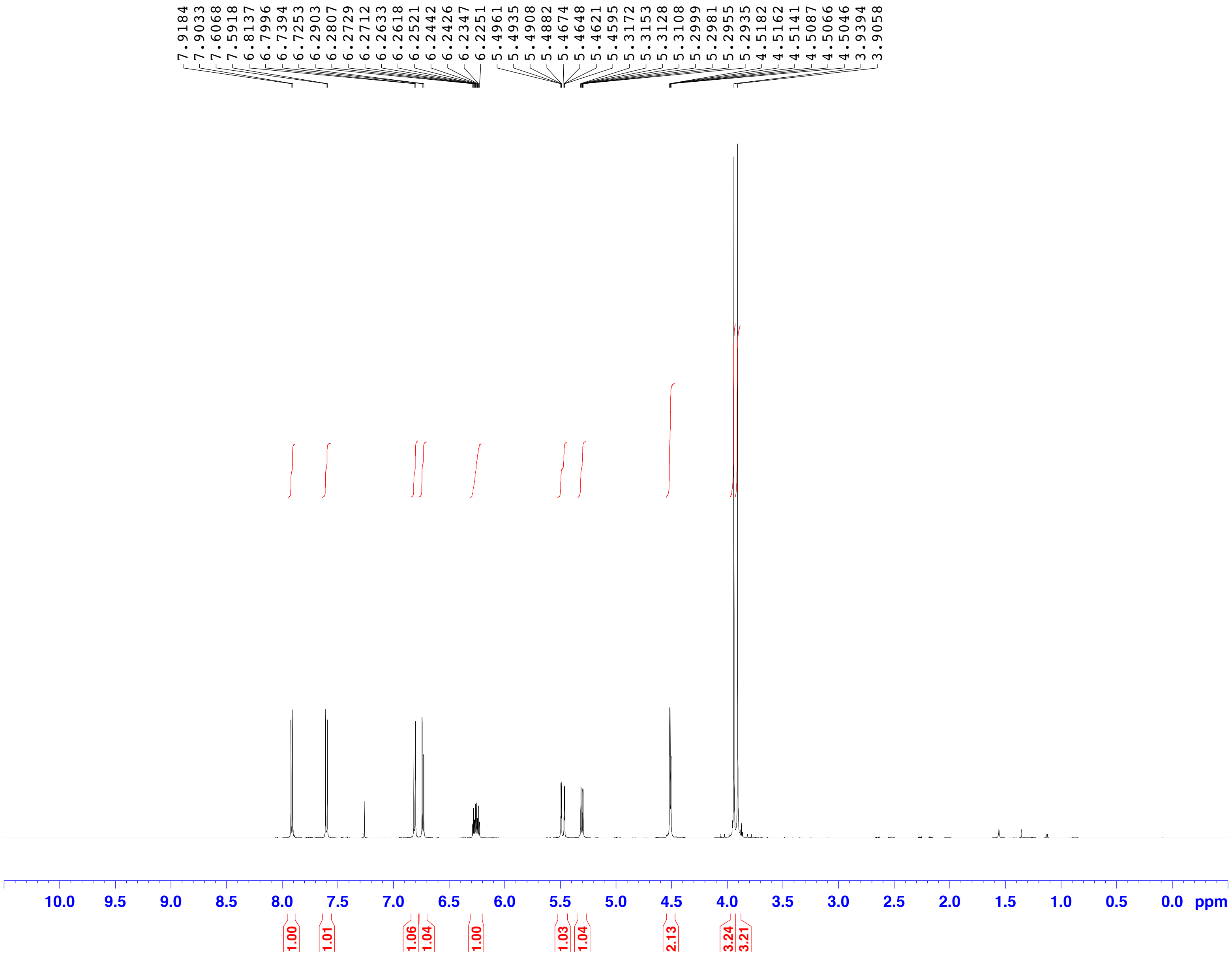

BRUKER

```
Current Data Parameters
NAME            YA1-1862-1
EXPNO                   20
PROCNO                   1

F2 - Acquisition Parameters
Date_             20201128
Time                 17.35
INSTRUM              spect
PROBHD    5 mm CPPBBO BB
PULPROG               zg30
TD                   65536
SOLVENT              CDCl3
NS                      16
DS                       2
SWH              12019.230 Hz
FIDRES            0.183399 Hz
AQ               2.7262976 sec
RG                    17.5
DW                  41.600 use
DE                   10.00 use
TE                   298.2 K
D1              1.00000000 sec
TD0                      1

======== CHANNEL f1 =======
SFO1         600.1337060 MHz
NUC1                    1H
P1                   12.00 use
PLW1         21.00000000 W

F2 - Processing parameters
SI                   65536
SF           600.1300148 MHz
WDW                     EM
SSB      0
LB                    0.30 Hz
GB       0
PC                    1.00
```

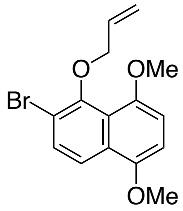

13b

^{13}C NMR (150 MHz, $CDCl_3$)

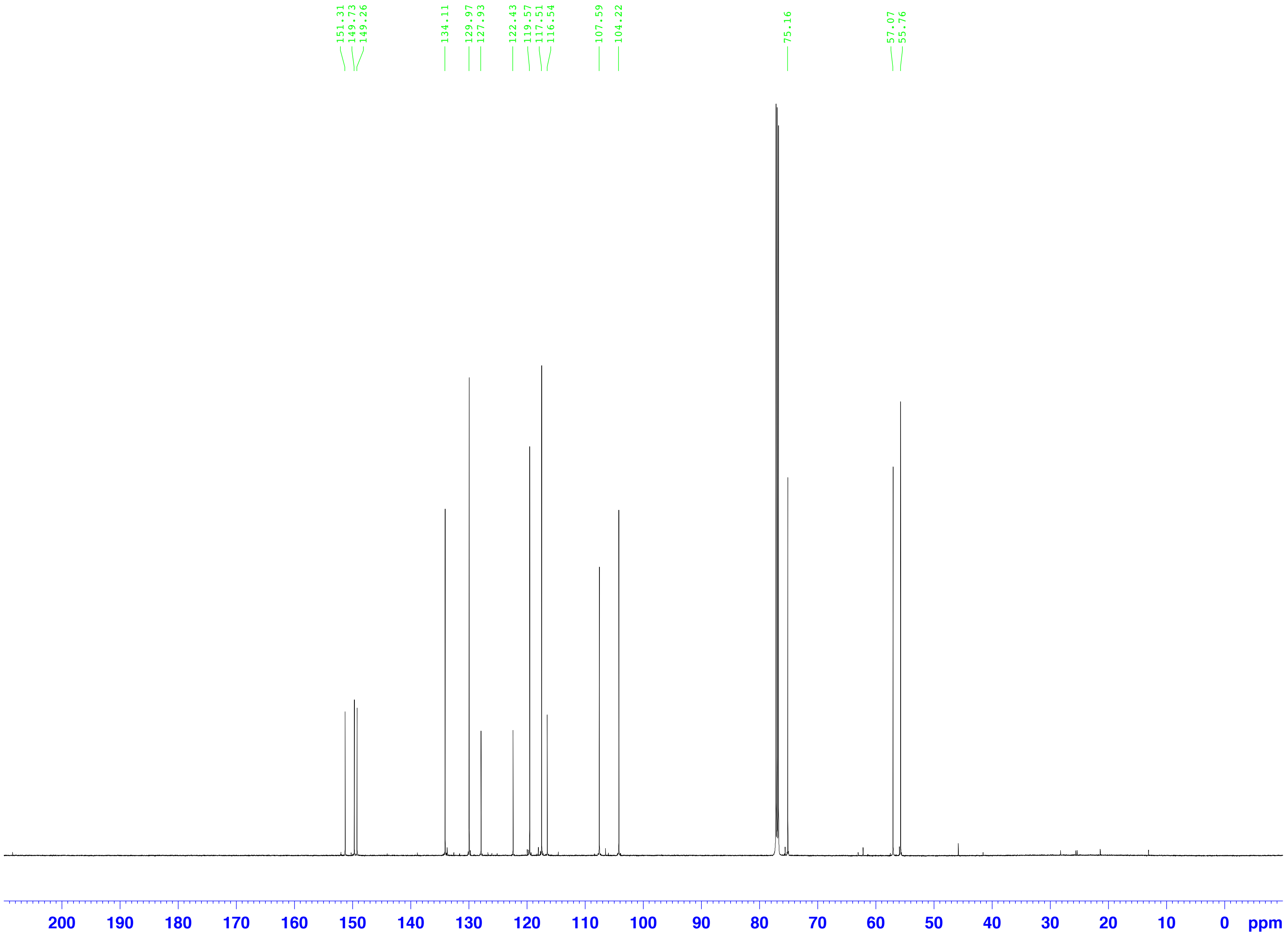


```
Current Data Parameters
NAME              YA1-1862-1
EXPNO                     21
PROCNO                     1

F2 - Acquisition Parameters
Date_               20201129
Time                   16.00
INSTRUM                spect
PROBHD   5 mm CPPBBO BB
PULPROG              zgpg30
TD                     65536
SOLVENT                CDCl3
NS                      3600
DS                         4
SWH               36057.691 Hz
FIDRES             0.550197 Hz
AQ                0.9087659 sec
RG                    175.56
DW                    13.867 use
DE                     18.00 use
TE                     298.2 K
D1                2.00000000 sec
D11               0.03000000 sec
TD0                        1

======== CHANNEL f1 =======
SFO1          150.9178981 MHz
NUC1                     13C
P1                     10.00 use
PLW1          80.00000000 W

======== CHANNEL f2 =======
SFO2          600.1324005 MHz
NUC2                      1H
CPDPRG[2             waltz16
PCPD2                  70.00 use
PLW2          13.43999958 W
PLW12          0.61714000 W
PLW13          0.31042001 W

F2 - Processing parameters
SI                     32768
SF            150.9028167 MHz
WDW                       EM
SSB       0
LB                      1.00 Hz
GB        0
PC                      1.40
```

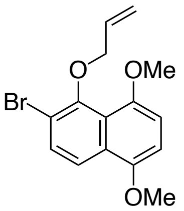

13b

^{1}H NMR (600 MHz, $CDCl_3$)

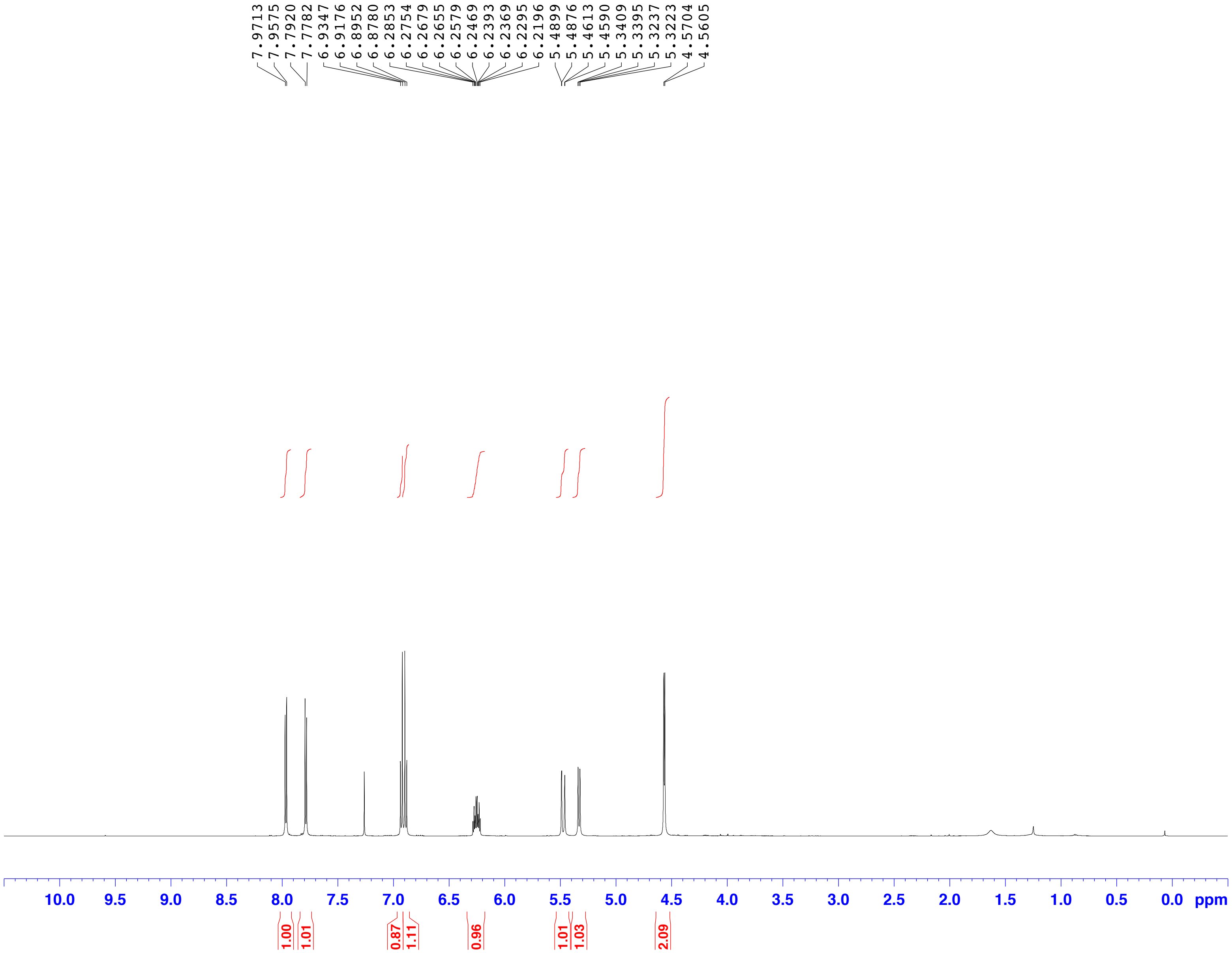

Current Data Parameters
NAME YA1-1865-crude
EXPNO 10
PROCNO 1

F2 - Acquisition Parameters
Date_ 20201207
Time 19.12
INSTRUM spect
PROBHD 5 mm CPPBBO BB
PULPROG zg30
TD 65536
SOLVENT CDCl3
NS 16
DS 2
SWH 12019.230 Hz
FIDRES 0.183399 Hz
AQ 2.7262976 sec
RG 31.94
DW 41.600 use
DE 10.00 use
TE 298.2 K
D1 1.00000000 sec
TD0 1

======== CHANNEL f1 =======
SFO1 600.1337060 MHz
NUC1 1H
P1 12.00 use
PLW1 21.00000000 W

F2 - Processing parameters
SI 65536
SF 600.1300147 MHz
WDW EM
SSB 0
LB 0.30 Hz
GB 0
PC 1.00

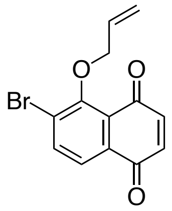

9b
(crude)

^{13}C NMR (150 MHz, $CDCl_3$)

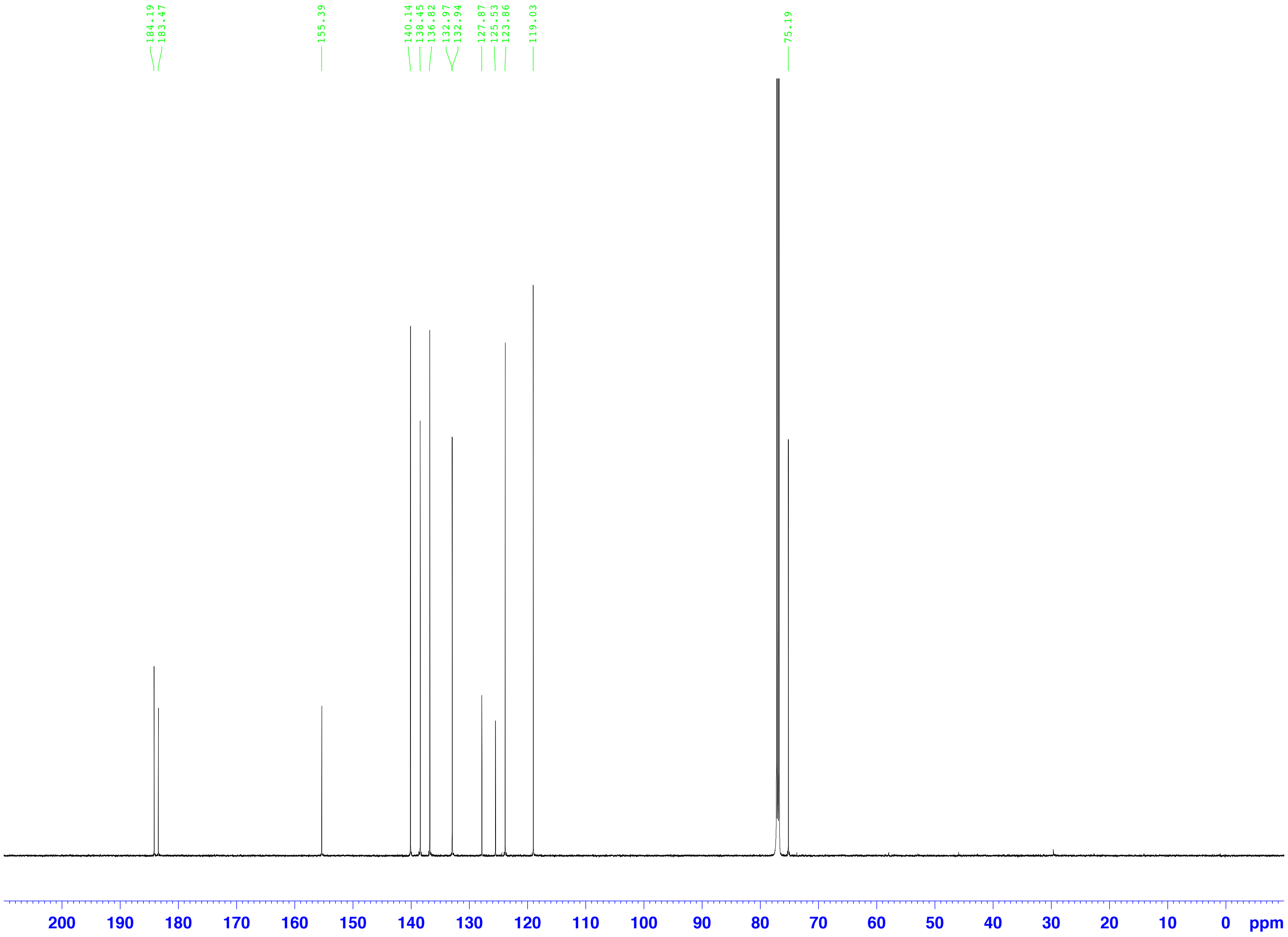


```
Current Data Parameters
NAME       YA1-1865-crude
EXPNO                  11
PROCNO                  1

F2 - Acquisition Parameters
Date_            20201208
Time                 0.32
INSTRUM             spect
PROBHD    5 mm CPPBBO BB
PULPROG           zgpg30
TD                 65536
SOLVENT            CDCl3
NS                  3000
DS                     4
SWH           36057.691 Hz
FIDRES         0.550197 Hz
AQ            0.9087659 sec
RG               175.56
DW               13.867 use
DE                18.00 use
TE                298.1 K
D1           2.00000000 sec
D11          0.03000000 sec
TD0                    1

======== CHANNEL f1 =======
SFO1        150.9178981 MHz
NUC1                 13C
P1                 10.00 use
PLW1         80.00000000 W

======== CHANNEL f2 =======
SFO2        600.1324005 MHz
NUC2                  1H
CPDPRG[2          waltz16
PCPD2              70.00 use
PLW2         13.43999958 W
PLW12         0.61714000 W
PLW13         0.31042001 W

F2 - Processing parameters
SI                 32768
SF          150.9028145 MHz
WDW                   EM
SSB      0
LB                  1.00 Hz
GB       0
PC                  1.40
```

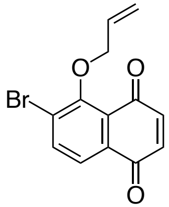

9b
(crude)

^{1}H NMR (600 MHz, $CDCl_3$)

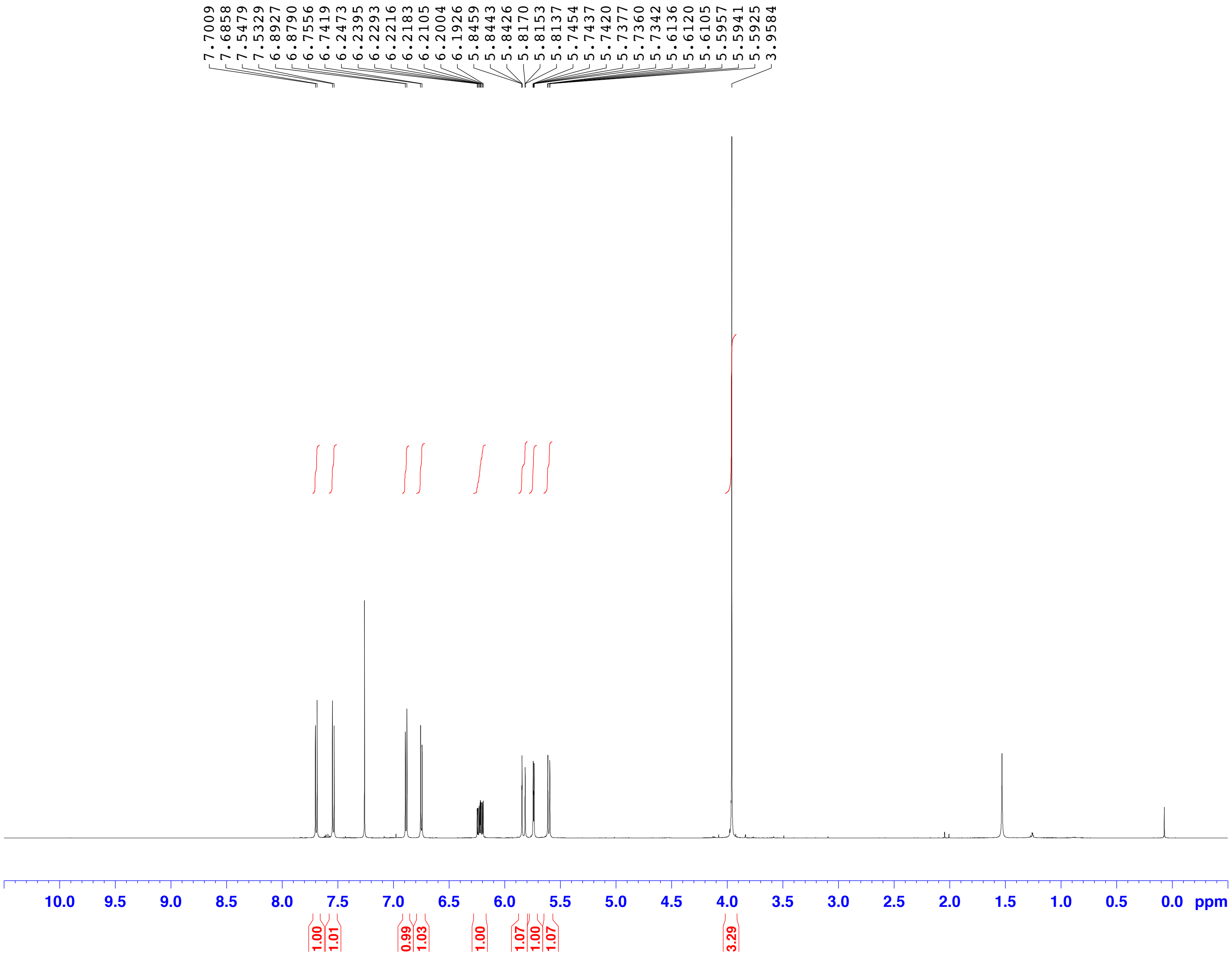


```
Current Data Parameters
NAME              YA1-1866-1
EXPNO                     10
PROCNO                     1

F2 - Acquisition Parameters
Date_               20201210
Time                   16.42
INSTRUM                spect
PROBHD    5 mm CPPBBO BB
PULPROG                 zg30
TD                     65536
SOLVENT                CDCl3
NS                        16
DS                         2
SWH               12019.230 Hz
FIDRES             0.183399 Hz
AQ                2.7262976 sec
RG                     31.94
DW                    41.600 use
DE                     10.00 use
TE                     298.2 K
D1               1.00000000 sec
TD0                        1

======== CHANNEL f1 =======
SFO1          600.1337060 MHz
NUC1                      1H
P1                     12.00 use
PLW1             21.00000000 W

F2 - Processing parameters
SI                     65536
SF            600.1300149 MHz
WDW                       EM
SSB         0
LB                      0.30 Hz
GB          0
PC                      1.00
```

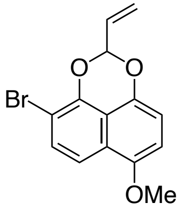

11b

^{13}C NMR (150 MHz, $CDCl_3$)

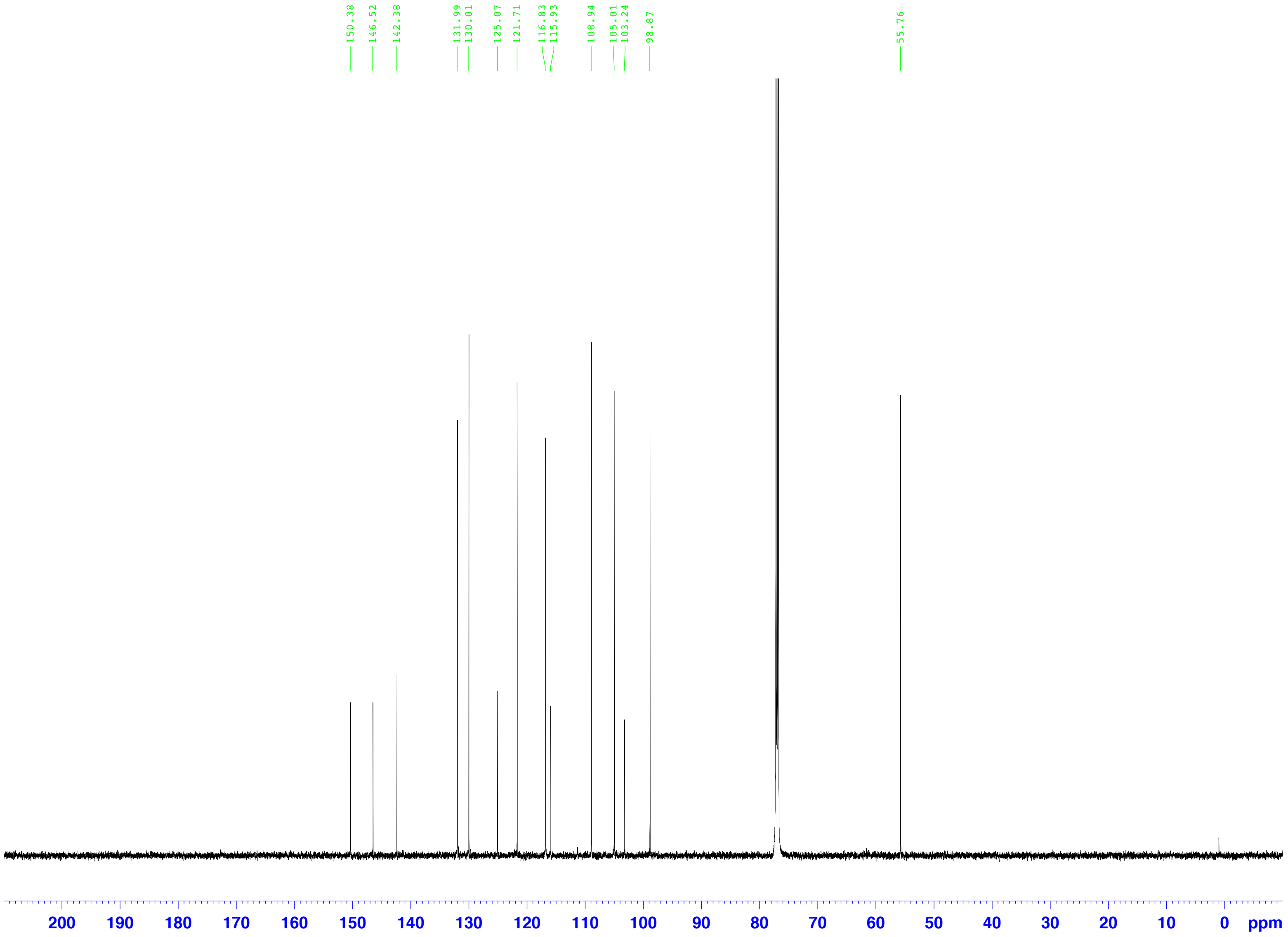

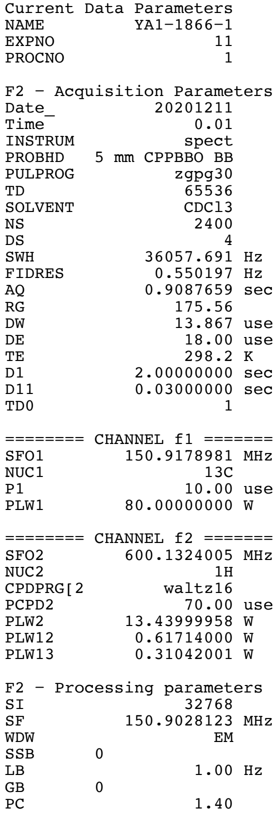

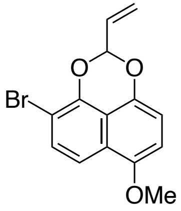

11b

^{1}H NMR (600 MHz, $CDCl_3$)

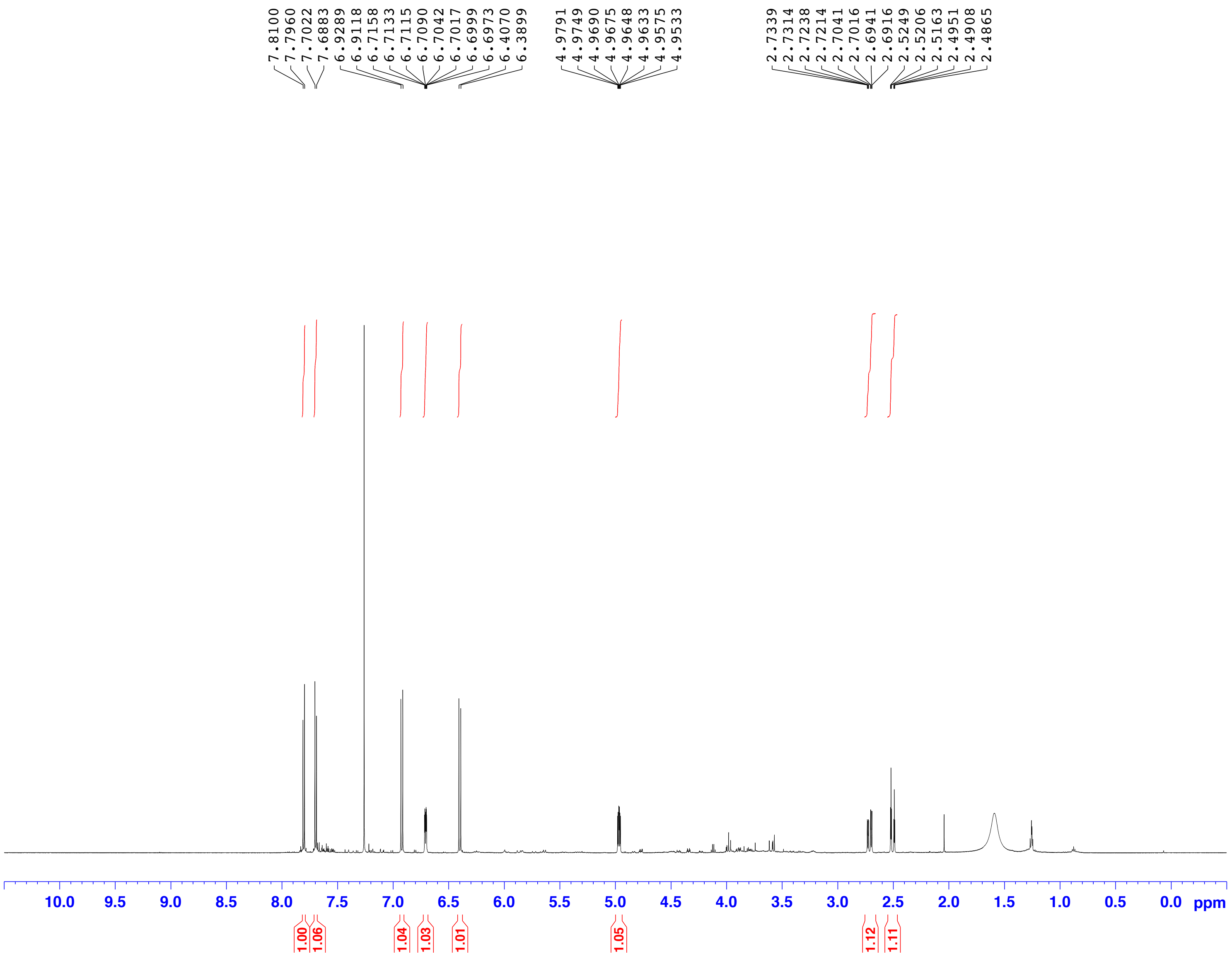


```
Current Data Parameters
NAME            YA1-1987-2
EXPNO                   10
PROCNO                   1

F2 - Acquisition Parameters
Date_             20210904
Time                 15.11
INSTRUM              spect
PROBHD   5 mm CPPBBO BB
PULPROG               zg30
TD                   65536
SOLVENT              CDCl3
NS                      16
DS                       2
SWH          12019.230 Hz
FIDRES        0.183399 Hz
AQ           2.7262976 sec
RG                   31.94
DW              41.600 usec
DE               10.00 usec
TE                 298.1 K
D1          1.00000000 sec
TD0                      1

======== CHANNEL f1 ========
SFO1       600.1337060 MHz
NUC1                    1H
P1               12.00 usec
PLW1         21.00000000 W

F2 - Processing parameters
SI                   65536
SF         600.1300143 MHz
WDW                     EM
SSB                      0
LB                 0.30 Hz
GB                       0
PC                    1.00
```

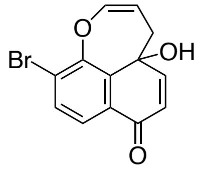

14

^{13}C NMR (150 MHz, $CDCl_3$)

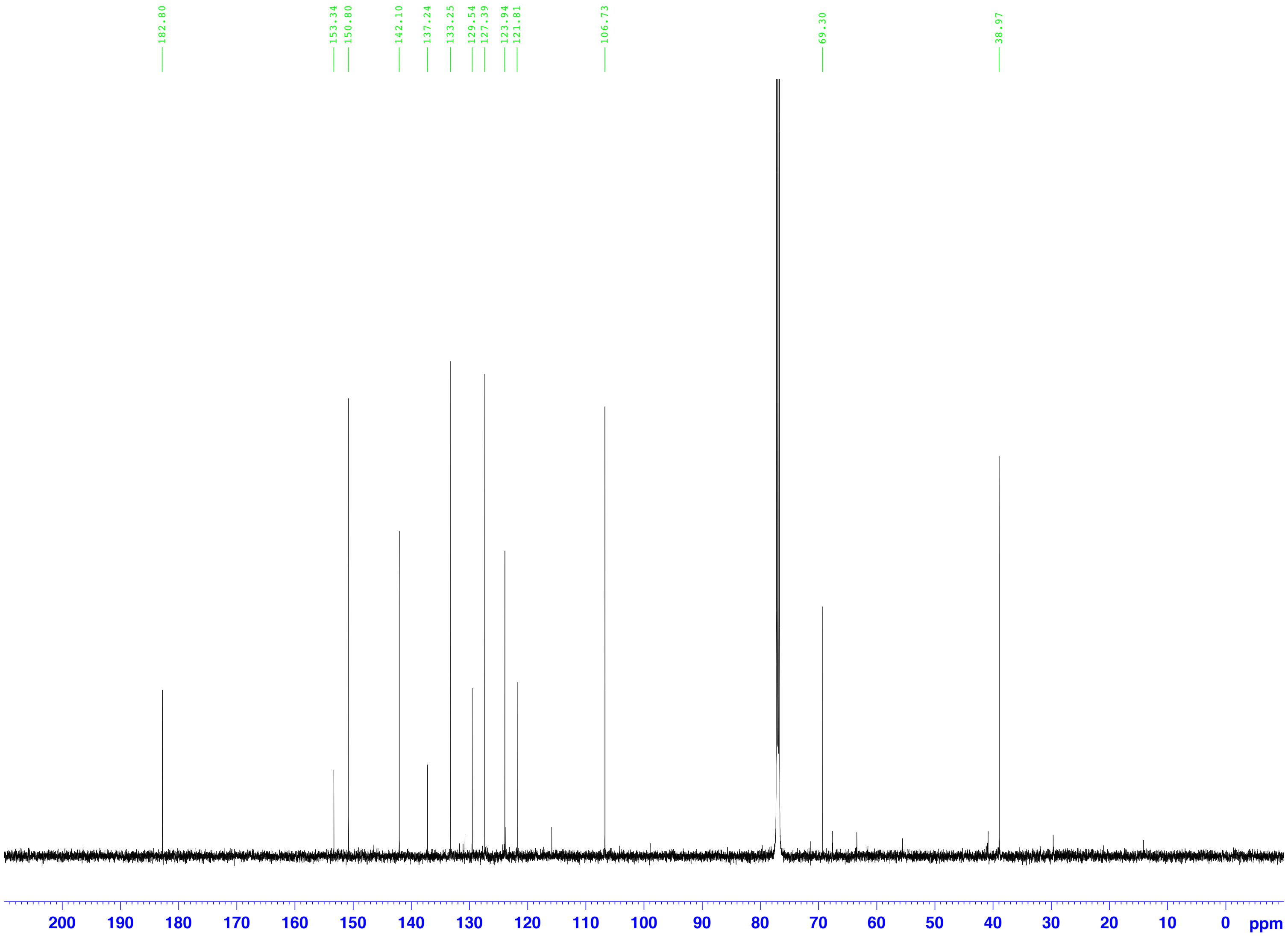

Current Data Parameters
NAME YA1-1987-2
EXPNO 12
PROCNO 1

F2 - Acquisition Parameters
Date_ 20210905
Time 13.01
INSTRUM spect
PROBHD 5 mm CPPBBO BB
PULPROG zgpg30
TD 65536
SOLVENT CDCl3
NS 2400
DS 4
SWH 36057.691 Hz
FIDRES 0.550197 Hz
AQ 0.9087659 sec
RG 175.56
DW 13.867 usec
DE 18.00 usec
TE 298.2 K
D1 2.00000000 sec
D11 0.03000000 sec
TD0 1

======== CHANNEL f1 ========
SFO1 150.9178981 MHz
NUC1 13C
P1 10.00 usec
PLW1 80.00000000 W

======== CHANNEL f2 ========
SFO2 600.1324005 MHz
NUC2 1H
CPDPRG[2 waltz16
PCPD2 70.00 usec
PLW2 13.43999958 W
PLW12 0.61714000 W
PLW13 0.31042001 W

F2 - Processing parameters
SI 32768
SF 150.9028123 MHz
WDW EM
SSB 0
LB 1.00 Hz
GB 0
PC 1.40

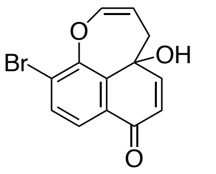

14

DEPT 135 (150 MHz, $CDCl_3$)

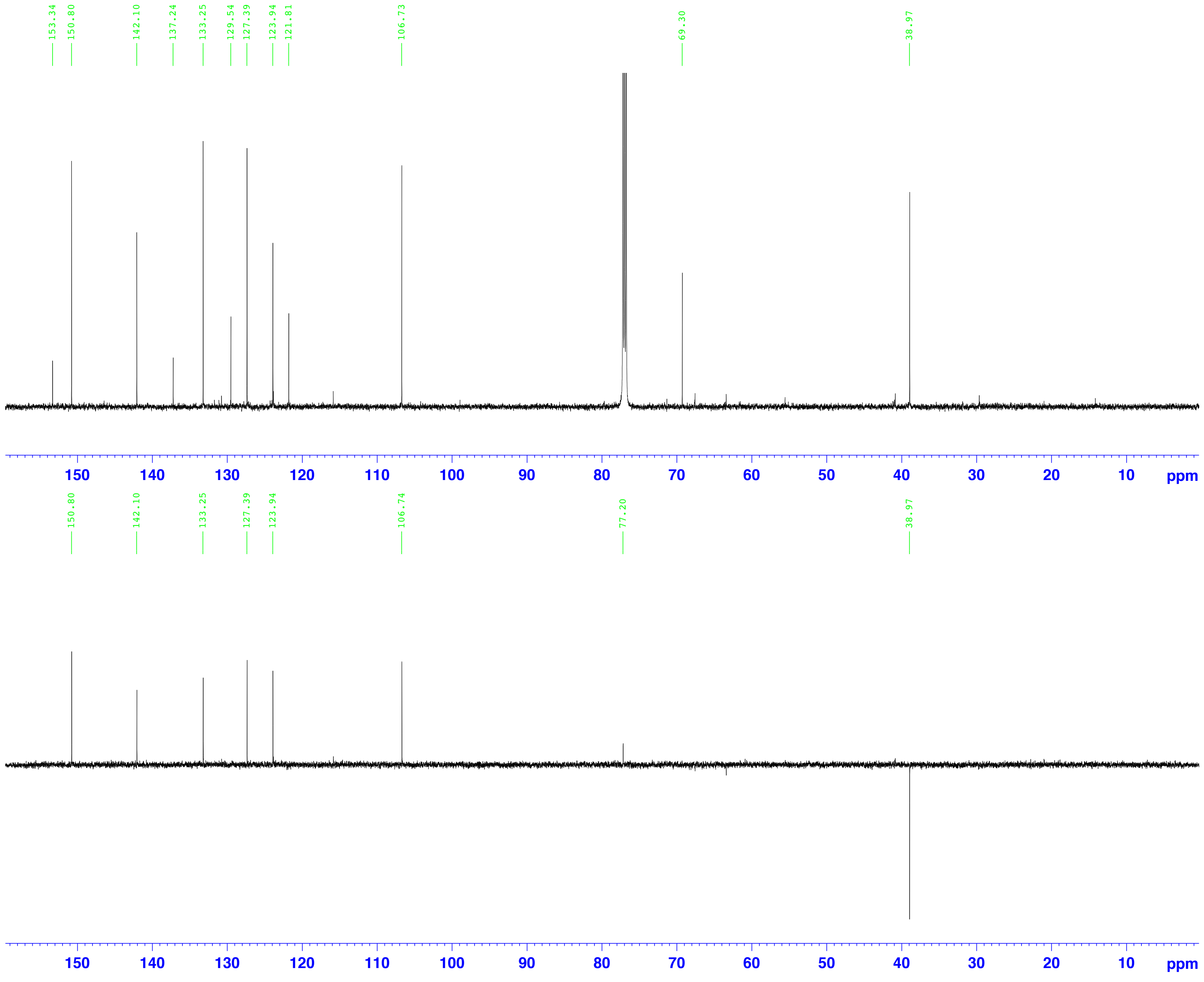


```
Current Data Parameters
NAME              YA1-1987-2
EXPNO                     16
PROCNO                     1

F2 - Acquisition Parameters
Date_               20210906
Time                    9.55
INSTRUM                spect
PROBHD      5 mm CPPBBO BB
PULPROG           deptsp135
TD                     65536
SOLVENT                CDCl3
NS                       256
DS                         4
SWH                24038.461 Hz
FIDRES              0.366798 Hz
AQ                 1.3631488 sec
RG                    175.56
DW                    20.800 usec
DE                     18.00 usec
TE                     298.2 K
CNST2            145.0000000
D1                2.00000000 sec
D2                0.00344828 sec
D12               0.00002000 sec
TD0                        1

======== CHANNEL f1 ========
SFO1             150.9148800 MHz
NUC1                     13C
P1                     10.00 usec
P13                  2000.00 usec
PLW0                       0 W
PLW1             80.00000000 W
SPNAM[5]        Crp60comp.4
SPOAL5                 0.500
SPOFFS5                    0 Hz
SPW5             12.22299957 W

======== CHANNEL f2 ========
SFO2             600.1319193 MHz
NUC2                      1H
CPDPRG[2             waltz16
P3                     15.00 usec
P4                     30.00 usec
PCPD2                  70.00 usec
PLW2             13.43999958 W
PLW12             0.61714000 W

F2 - Processing parameters
SI                     32768
SF               150.9028118 MHz
WDW                       EM
SSB                        0
LB                      1.00 Hz
GB                         0
PC                      1.40
```

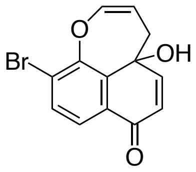

14

HSQC ($CDCl_3$)

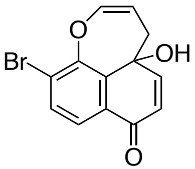

14

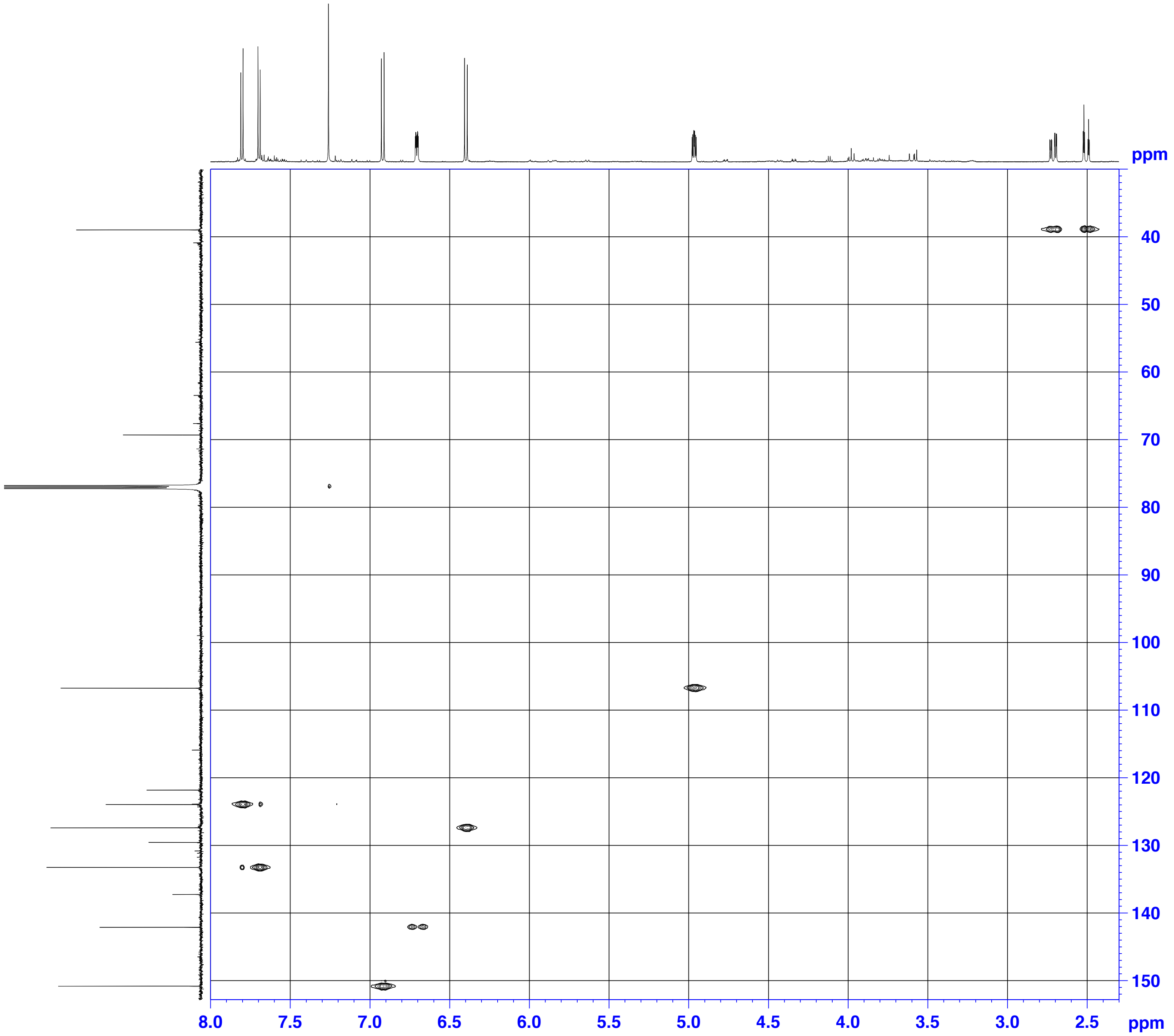

Current Data Parameters
NAME YA1-1987-2
EXPNO 17
PROCNO 1

F2 - Acquisition Parameters
Date_ 20210907
Time 1.34
INSTRUM spect
PROBHD 5 mm CPPBBO BB
PULPROG hsqcedetgpsisp2.3
TD 2048
SOLVENT CDCl3
NS 4
DS 32
SWH 7211.539 Hz
FIDRES 3.521259 Hz
AQ 0.1419947 sec
RG 175.56
DW 69.333 usec
DE 10.00 usec
TE 298.2 K
CNST2 145.0000000
CNST17 -0.5000000
D0 0.00000300 sec
D1 2.00000000 sec
D4 0.00172414 sec
D11 0.03000000 sec
D16 0.00020000 sec
D21 0.00360000 sec
D24 0.00089000 sec
IN0 0.00002000 sec

======== CHANNEL f1 ========
SFO1 600.1328224 MHz
NUC1 1H
P1 12.00 usec
P2 24.00 usec
P28 0 usec
PLW1 21.00000000 W

======== CHANNEL f2 ========
SFO2 150.9133710 MHz
NUC2 13C
CPDPRG[2 garp
P3 10.00 usec
P14 500.00 usec
P24 2000.00 usec
P31 1730.00 usec
PCPD2 60.00 usec
PLW0 0 W
PLW2 80.00000000 W
PLW12 2.22219992 W
SPNAM[3] Crp60,0.5,20.1
SPOAL3 0.500
SPOFFS3 0 Hz
SPW3 12.22299957 W
SPNAM[7] Crp60comp.4
SPOAL7 0.500
SPOFFS7 0 Hz
SPW7 12.22299957 W
SPNAM[18 Crp60_xfilt.2
SPOAL18 0.500
SPOFFS18 0 Hz
SPW18 3.53270006 W

====== GRADIENT CHANNEL =====
GPNAM[1] SMSQ10.100
GPNAM[2] SMSQ10.100
GPNAM[3] SMSQ10.100
GPNAM[4] SMSQ10.100
GPZ1 80.00 %
GPZ2 20.10 %
GPZ3 11.00 %
GPZ4 -5.00 %
P16 1000.00 usec
P19 600.00 usec

F1 - Acquisition parameters
TD 256
SFO1 150.9134 MHz
FIDRES 195.312500 Hz
SW 165.658 ppm
FnMODE Echo-Antiecho

F2 - Processing parameters
SI 1024
SF 600.1300143 MHz
WDW QSINE
SSB 2
LB 0 Hz
GB 0
PC 1.40

F1 - Processing parameters
SI 1024
MC2 echo-antiecho
SF 150.9028123 MHz
WDW QSINE
SSB 2
LB 0 Hz
GB 0

HMBC ($CDCl_3$)

key HMBC correlations

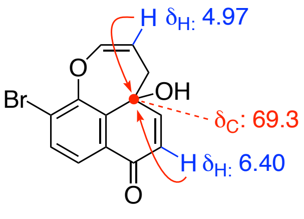

14

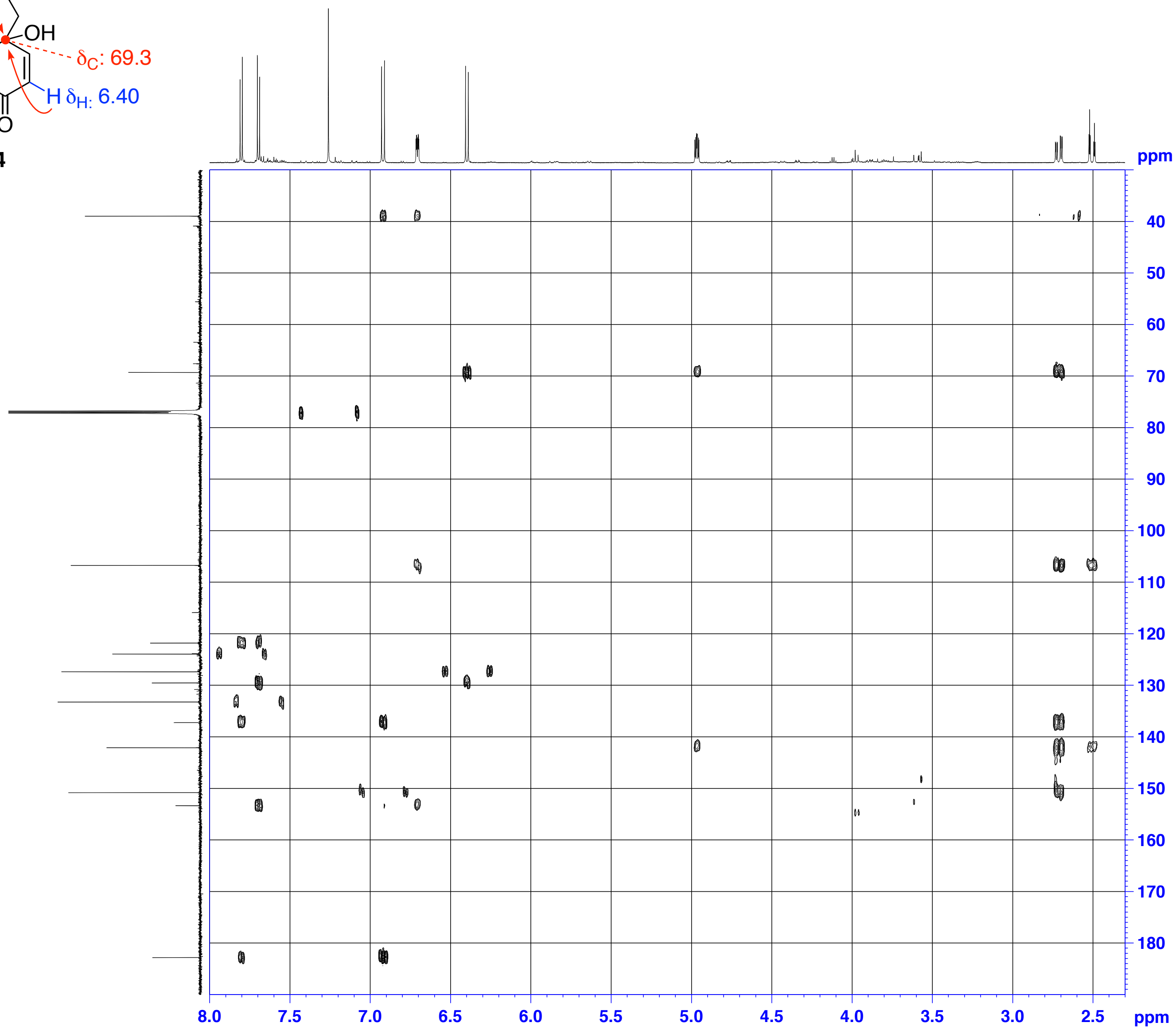

Current Data Parameters
NAME YA1-1987-2
EXPNO 13
PROCNO 1

F2 - Acquisition Parameters
Date_ 20210905
Time 13.02
INSTRUM spect
PROBHD 5 mm CPPBBO BB
PULPROG hmbcgplpndqf
TD 2048
SOLVENT CDCl3
NS 4
DS 16
SWH 4807.692 Hz
FIDRES 2.347506 Hz
AQ 0.2129920 sec
RG 175.56
DW 104.000 usec
DE 10.00 usec
TE 298.2 K
CNST2 145.0000000
CNST13 10.0000000
D0 0.00000300 sec
D1 1.41807997 sec
D2 0.00344828 sec
D6 0.05000000 sec
D16 0.00020000 sec
IN0 0.00001490 sec

======== CHANNEL f1 ========
SFO1 600.1326119 MHz
NUC1 1H
P1 12.00 usec
P2 24.00 usec
PLW1 21.00000000 W

======== CHANNEL f2 ========
SFO2 150.9178741 MHz
NUC2 13C
P3 10.00 usec
PLW2 80.00000000 W

====== GRADIENT CHANNEL =====
GPNAM[1] SMSQ10.100
GPNAM[2] SMSQ10.100
GPNAM[3] SMSQ10.100
GPZ1 50.00 %
GPZ2 30.00 %
GPZ3 40.10 %
P16 1000.00 usec

F1 - Acquisition parameters
TD 128
SFO1 150.9179 MHz
FIDRES 524.328857 Hz
SW 222.353 ppm
FnMODE QF

F2 - Processing parameters
SI 2048
SF 600.1300143 MHz
WDW SINE
SSB 0
LB 0 Hz
GB 0
PC 1.40

F1 - Processing parameters
SI 1024
MC2 QF
SF 150.9028123 MHz
WDW SINE
SSB 0
LB 0 Hz
GB 0

^{1}H NMR (600 MHz, $CDCl_3$)

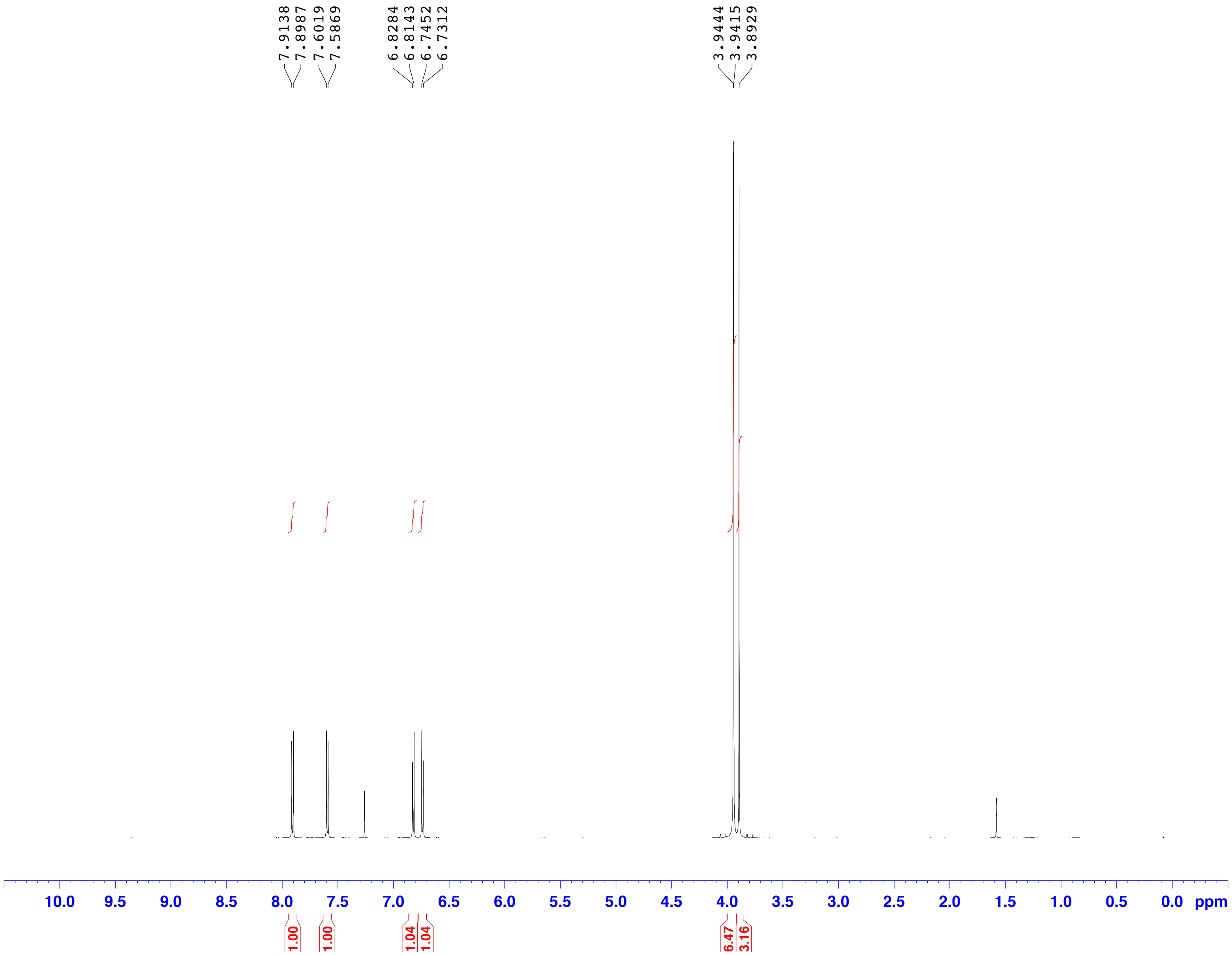

BRUKER

```
Current Data Parameters
NAME             YA1-1741-1
EXPNO                    10
PROCNO                    1

F2 - Acquisition Parameters
Date_              20200116
Time                  22.02
INSTRUM               spect
PROBHD    5 mm CPPBBO BB
PULPROG                zg30
TD                    65536
SOLVENT               CDCl3
NS                       16
DS                        2
SWH               12019.230 Hz
FIDRES             0.183399 Hz
AQ                2.7262976 sec
RG                     17.5
DW                   41.600 use
DE                    10.00 use
TE                    298.2 K
D1               1.00000000 sec
TD0                       1

======== CHANNEL f1 =======
SFO1            600.1337060 MHz
NUC1                     1H
P1                    12.00 use
PLW1            21.00000000 W

F2 - Processing parameters
SI                    65536
SF              600.1300148 MHz
WDW                      EM
SSB       0
LB                     0.30 Hz
GB        0
PC                     1.00
```

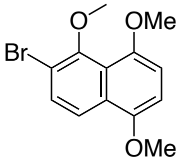

13c

^{13}C NMR (150 MHz, $CDCl_3$)

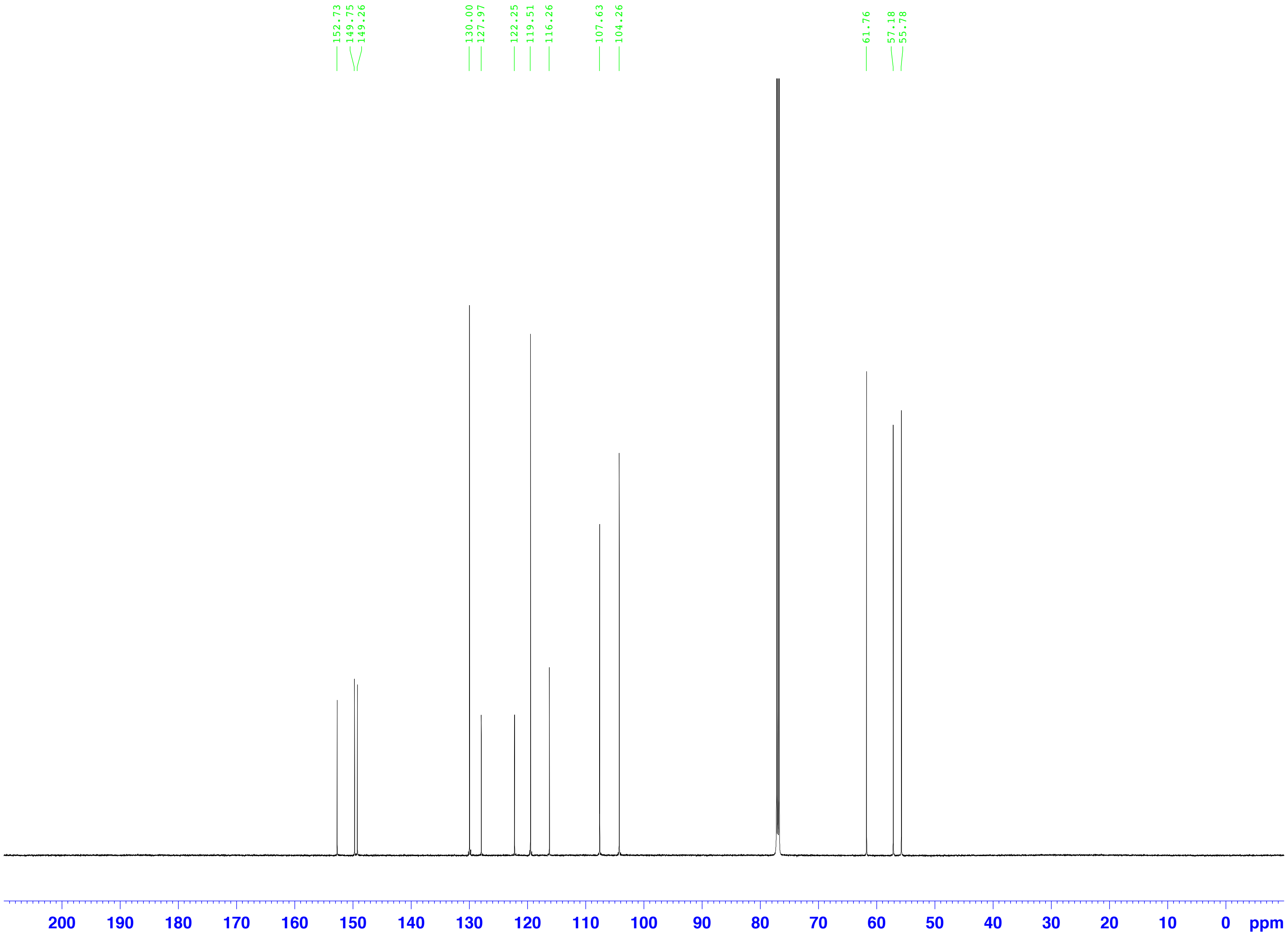


```
Current Data Parameters
NAME              YA1-1741-1
EXPNO                     11
PROCNO                     1

F2 - Acquisition Parameters
Date_               20200118
Time                    8.46
INSTRUM                spect
PROBHD    5 mm CPPBBO BB
PULPROG              zgpg30
TD                     65536
SOLVENT                CDCl3
NS                      3000
DS                         4
SWH               36057.691 Hz
FIDRES             0.550197 Hz
AQ                0.9087659 sec
RG                    175.56
DW                    13.867 use
DE                     18.00 use
TE                     298.2 K
D1                2.00000000 sec
D11               0.03000000 sec
TD0                        1

======== CHANNEL f1 =======
SFO1           150.9178981 MHz
NUC1                     13C
P1                     10.00 use
PLW1             80.00000000 W

======== CHANNEL f2 =======
SFO2           600.1324005 MHz
NUC2                      1H
CPDPRG[2             waltz16
PCPD2                  70.00 use
PLW2             13.43999958 W
PLW12             0.61714000 W
PLW13             0.31042001 W

F2 - Processing parameters
SI                     32768
SF             150.9028154 MHz
WDW                       EM
SSB      0
LB                      1.00 Hz
GB       0
PC                      1.40
```

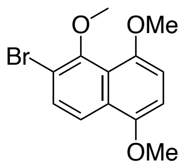

13c

^{1}H NMR (600 MHz, $CDCl_3$)

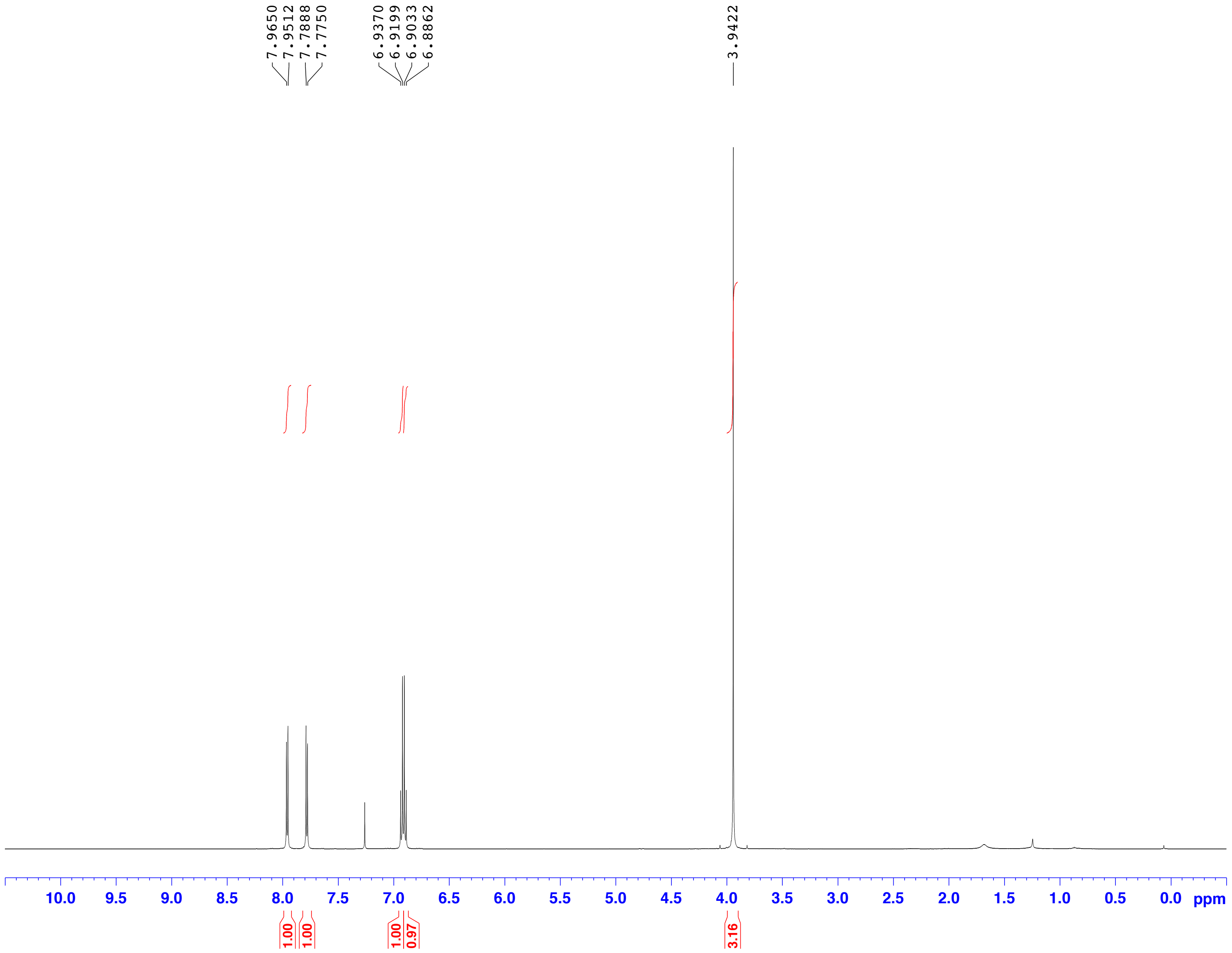


```
Current Data Parameters
NAME      YA1-1856-crude
EXPNO                 10
PROCNO                 1

F2 - Acquisition Parameters
Date_           20201118
Time               16.57
INSTRUM            spect
PROBHD   5 mm CPPBBO BB
PULPROG             zg30
TD                 65536
SOLVENT            CDCl3
NS                    16
DS                     2
SWH            12019.230 Hz
FIDRES          0.183399 Hz
AQ             2.7262976 sec
RG                  17.5
DW                41.600 usec
DE                 10.00 usec
TE                 298.2 K
D1            1.00000000 sec
TD0                    1

======== CHANNEL f1 ========
SFO1         600.1337060 MHz
NUC1                  1H
P1                 12.00 usec
PLW1         21.00000000 W

F2 - Processing parameters
SI                 65536
SF           600.1300145 MHz
WDW                   EM
SSB                    0
LB                  0.30 Hz
GB                     0
PC                  1.00
```

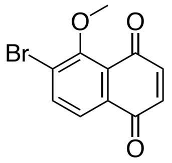

9c
(crude)

^{13}C NMR (150 MHz, $CDCl_3$)

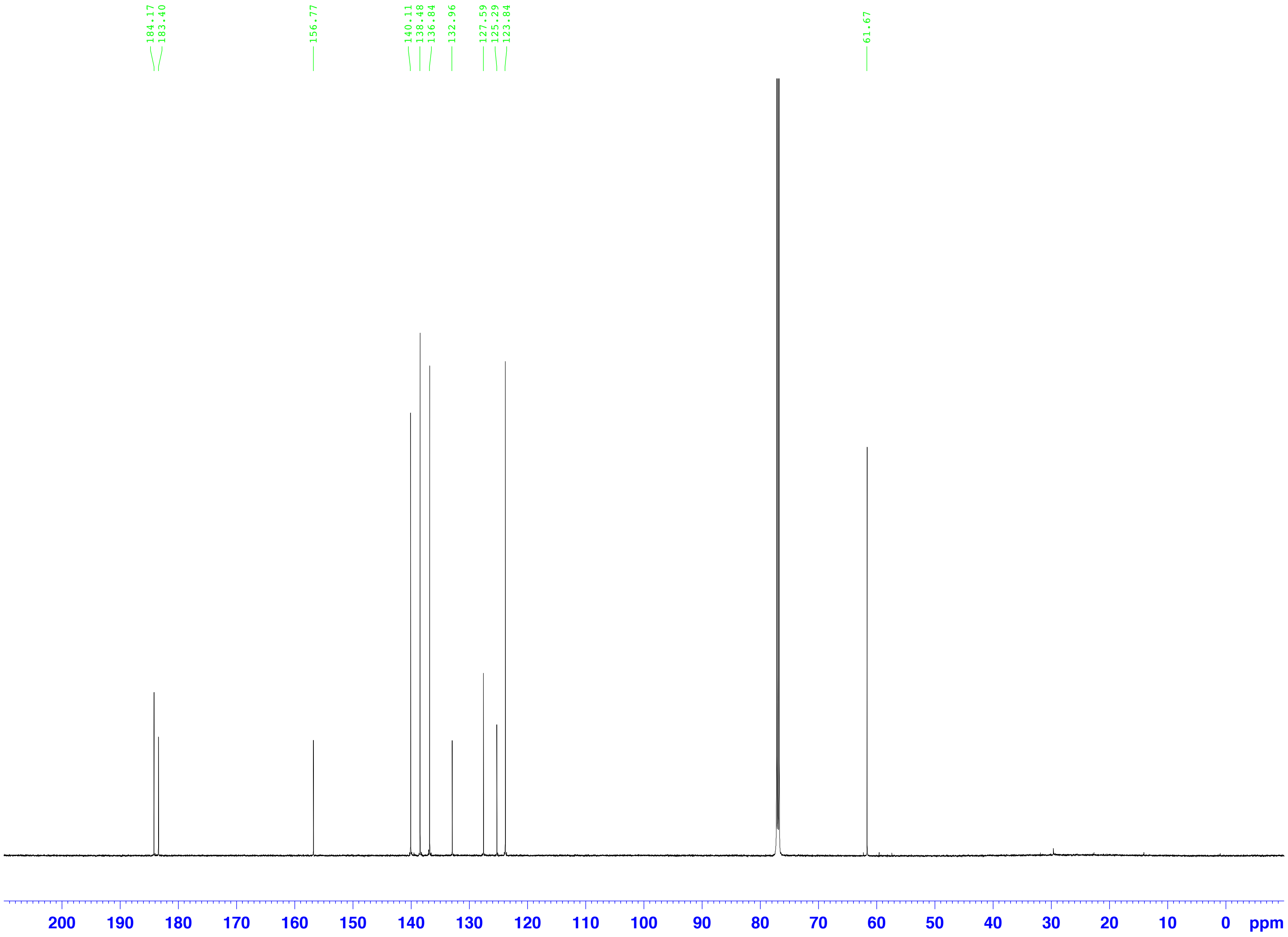


```
Current Data Parameters
NAME      YA1-1856-crude
EXPNO                 11
PROCNO                 1

F2 - Acquisition Parameters
Date_           20201119
Time                0.01
INSTRUM            spect
PROBHD    5 mm CPPBBO BB
PULPROG          zgpg30
TD                 65536
SOLVENT            CDCl3
NS                  2400
DS                     4
SWH            36057.691 Hz
FIDRES          0.550197 Hz
AQ             0.9087659 sec
RG                175.56
DW                13.867 use
DE                 18.00 use
TE                 298.2 K
D1            2.00000000 sec
D11           0.03000000 sec
TD0                    1

======== CHANNEL f1 =======
SFO1         150.9178981 MHz
NUC1                 13C
P1                 10.00 use
PLW1         80.00000000 W

======== CHANNEL f2 =======
SFO2         600.1324005 MHz
NUC2                  1H
CPDPRG[2         waltz16
PCPD2              70.00 use
PLW2         13.43999958 W
PLW12         0.61714000 W
PLW13         0.31042001 W

F2 - Processing parameters
SI                 32768
SF           150.9028149 MHz
WDW                   EM
SSB       0
LB                  1.00 Hz
GB        0
PC                  1.40
```

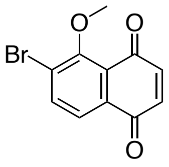

9c
(crude)

^{1}H NMR (600 MHz, $CDCl_3$)

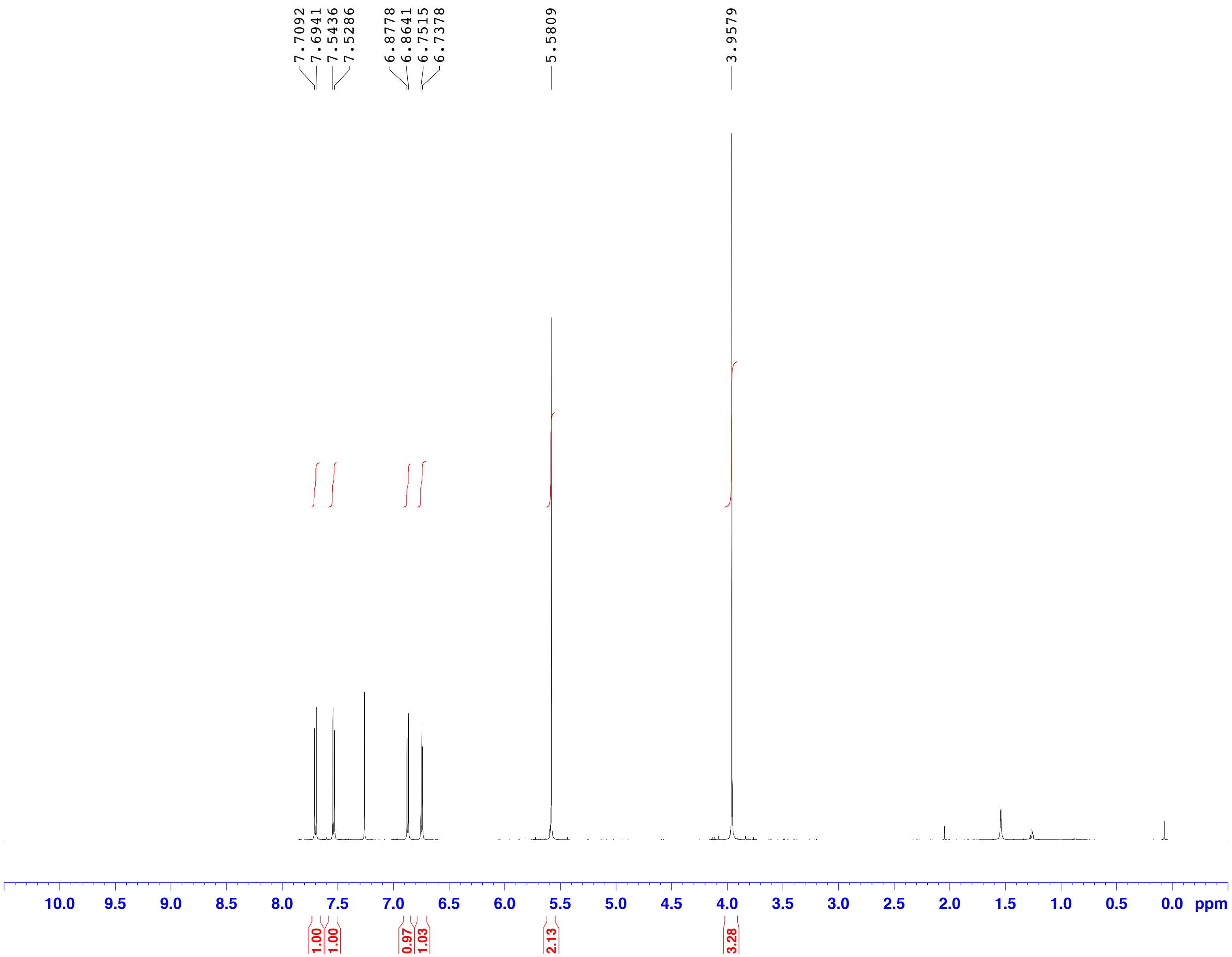


```
Current Data Parameters
NAME              YA1-1857-1
EXPNO                     10
PROCNO                     1

F2 - Acquisition Parameters
Date_               20201201
Time                   15.20
INSTRUM                spect
PROBHD    5 mm CPPBBO BB
PULPROG                 zg30
TD                     65536
SOLVENT                CDCl3
NS                        16
DS                         2
SWH             12019.230 Hz
FIDRES           0.183399 Hz
AQ              2.7262976 sec
RG                     31.94
DW                41.600 use
DE                 10.00 use
TE                  298.2 K
D1            1.00000000 sec
TD0                        1

======== CHANNEL f1 =======
SFO1         600.1337060 MHz
NUC1                      1H
P1                 12.00 use
PLW1          21.00000000 W

F2 - Processing parameters
SI                     65536
SF           600.1300149 MHz
WDW                       EM
SSB       0
LB                   0.30 Hz
GB        0
PC                      1.00
```

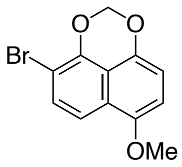

11c

^{13}C NMR (150 MHz, $CDCl_3$)

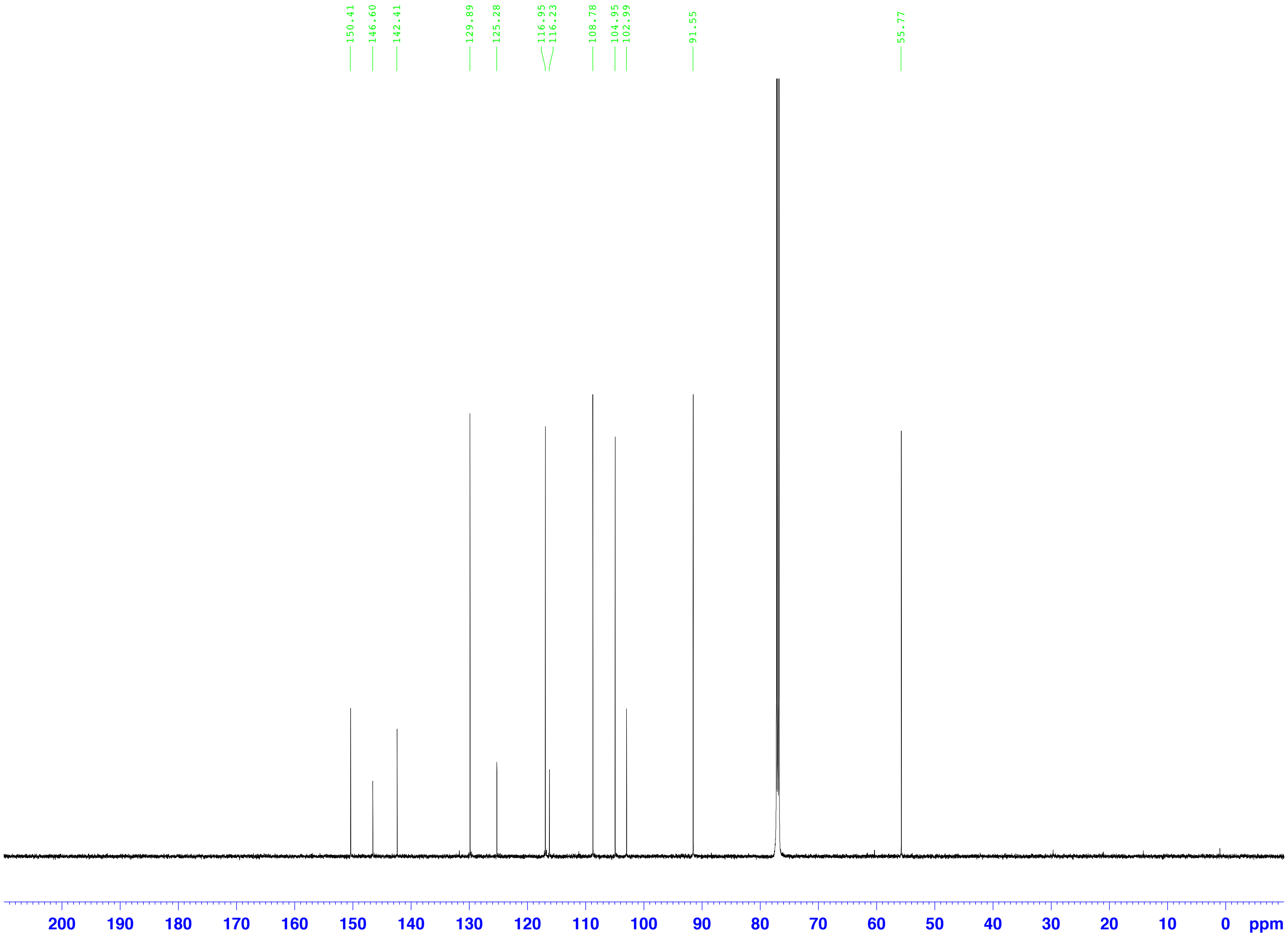


```
Current Data Parameters
NAME              YA1-1857-1
EXPNO                     11
PROCNO                     1

F2 - Acquisition Parameters
Date_               20201202
Time                    6.31
INSTRUM                spect
PROBHD     5 mm CPPBBO BB
PULPROG              zgpg30
TD                     65536
SOLVENT                CDCl3
NS                      2400
DS                         4
SWH               36057.691 Hz
FIDRES             0.550197 Hz
AQ                0.9087659 sec
RG                    175.56
DW                    13.867 use
DE                     18.00 use
TE                     298.1 K
D1               2.00000000 sec
D11              0.03000000 sec
TD0                        1

======== CHANNEL f1 =======
SFO1           150.9178981 MHz
NUC1                     13C
P1                     10.00 use
PLW1           80.00000000 W

======== CHANNEL f2 =======
SFO2           600.1324005 MHz
NUC2                      1H
CPDPRG[2             waltz16
PCPD2                  70.00 use
PLW2           13.43999958 W
PLW12           0.61714000 W
PLW13           0.31042001 W

F2 - Processing parameters
SI                     32768
SF             150.9028123 MHz
WDW                       EM
SSB       0
LB                      1.00 Hz
GB        0
PC                      1.40
```

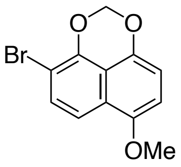

11c

^1H NMR (600 MHz, $CDCl_3$)

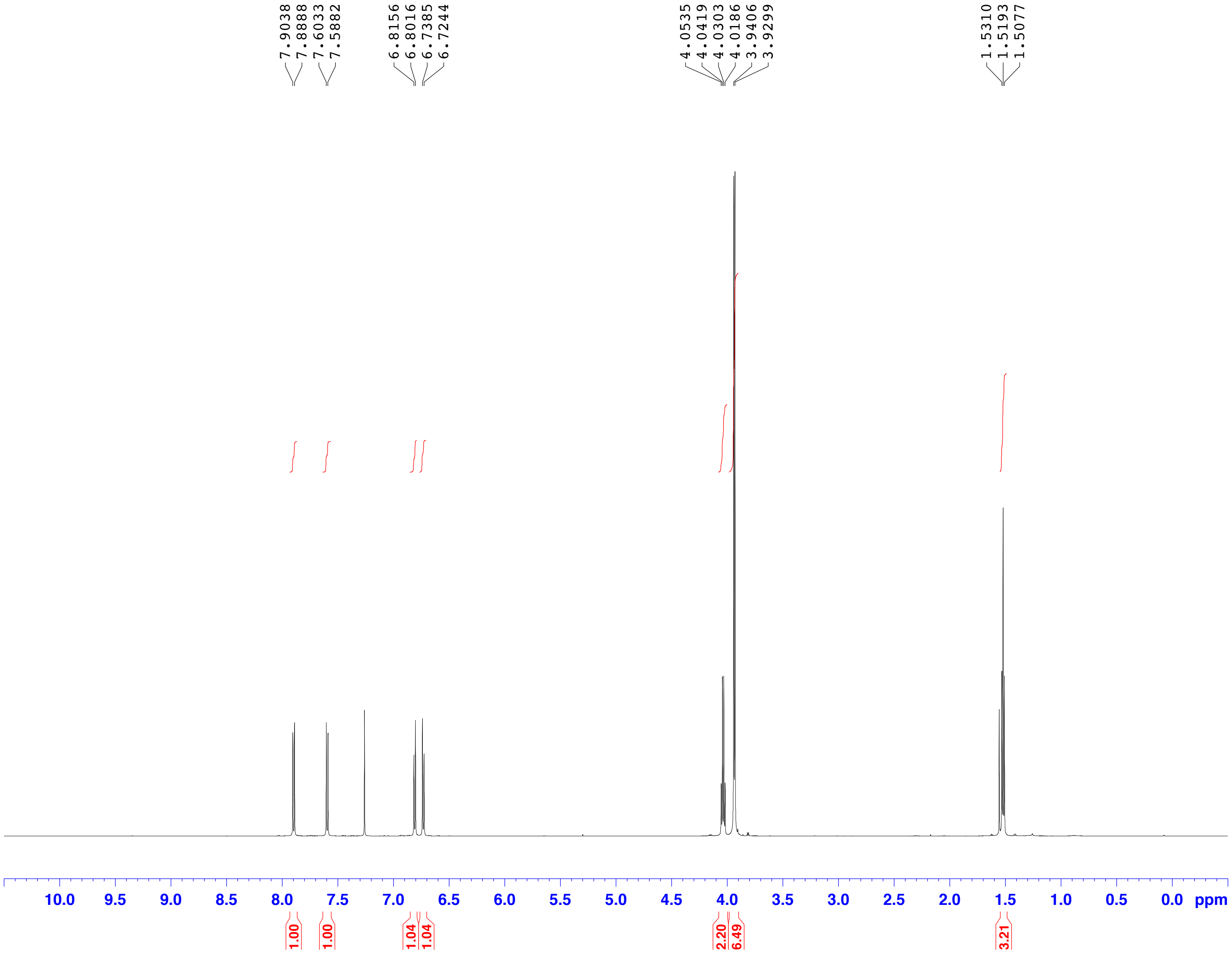

Current Data Parameters
NAME YA1-1742-1
EXPNO 10
PROCNO 1

F2 - Acquisition Parameters
Date_ 20200116
Time 22.06
INSTRUM spect
PROBHD 5 mm CPPBBO BB
PULPROG zg30
TD 65536
SOLVENT CDCl3
NS 16
DS 2
SWH 12019.230 Hz
FIDRES 0.183399 Hz
AQ 2.7262976 sec
RG 31.94
DW 41.600 use
DE 10.00 use
TE 298.2 K
D1 1.00000000 sec
TD0 1

======== CHANNEL f1 =======
SFO1 600.1337060 MHz
NUC1 1H
P1 12.00 use
PLW1 21.00000000 W

F2 - Processing parameters
SI 65536
SF 600.1300148 MHz
WDW EM
SSB 0
LB 0.30 Hz
GB 0
PC 1.00

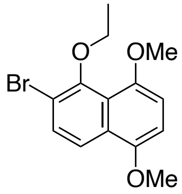

13d

^{13}C NMR (150 MHz, $CDCl_3$)

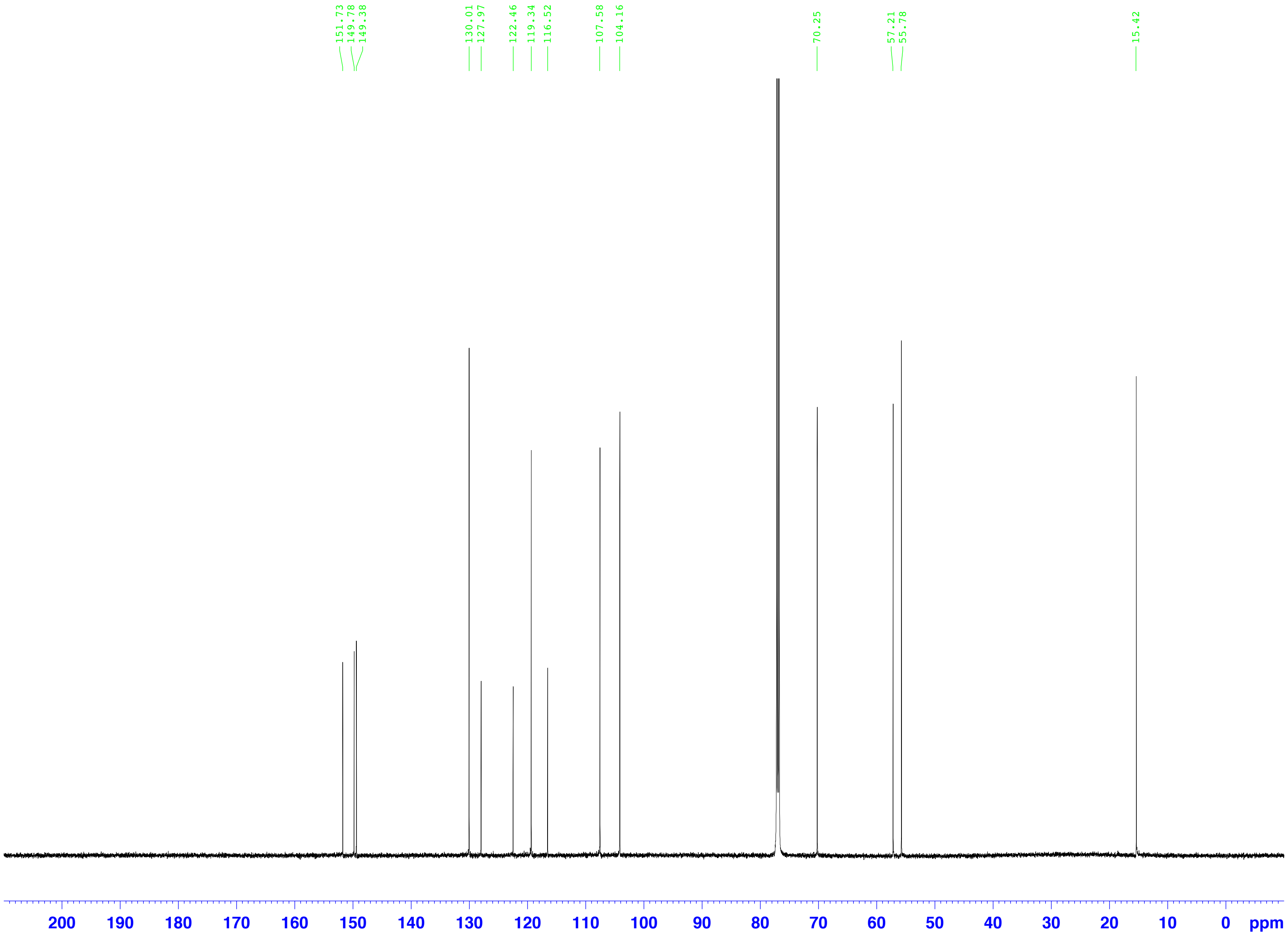


```
Current Data Parameters
NAME            YA1-1742-1
EXPNO                   11
PROCNO                   1

F2 - Acquisition Parameters
Date_             20200118
Time                 11.46
INSTRUM              spect
PROBHD   5 mm CPPBBO BB
PULPROG            zgpg30
TD                   65536
SOLVENT              CDCl3
NS                    3000
DS                       4
SWH             36057.691 Hz
FIDRES           0.550197 Hz
AQ              0.9087659 sec
RG                  175.56
DW                  13.867 use
DE                   18.00 use
TE                   298.2 K
D1              2.00000000 sec
D11             0.03000000 sec
TD0                      1

======== CHANNEL f1 =======
SFO1          150.9178981 MHz
NUC1                   13C
P1                   10.00 use
PLW1           80.00000000 W

======== CHANNEL f2 =======
SFO2          600.1324005 MHz
NUC2                    1H
CPDPRG[2           waltz16
PCPD2                70.00 use
PLW2           13.43999958 W
PLW12           0.61714000 W
PLW13           0.31042001 W

F2 - Processing parameters
SI                   32768
SF            150.9028135 MHz
WDW                     EM
SSB       0
LB                    1.00 Hz
GB        0
PC                    1.40
```

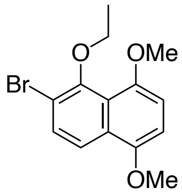

13d

^1H NMR (600 MHz, $CDCl_3$)

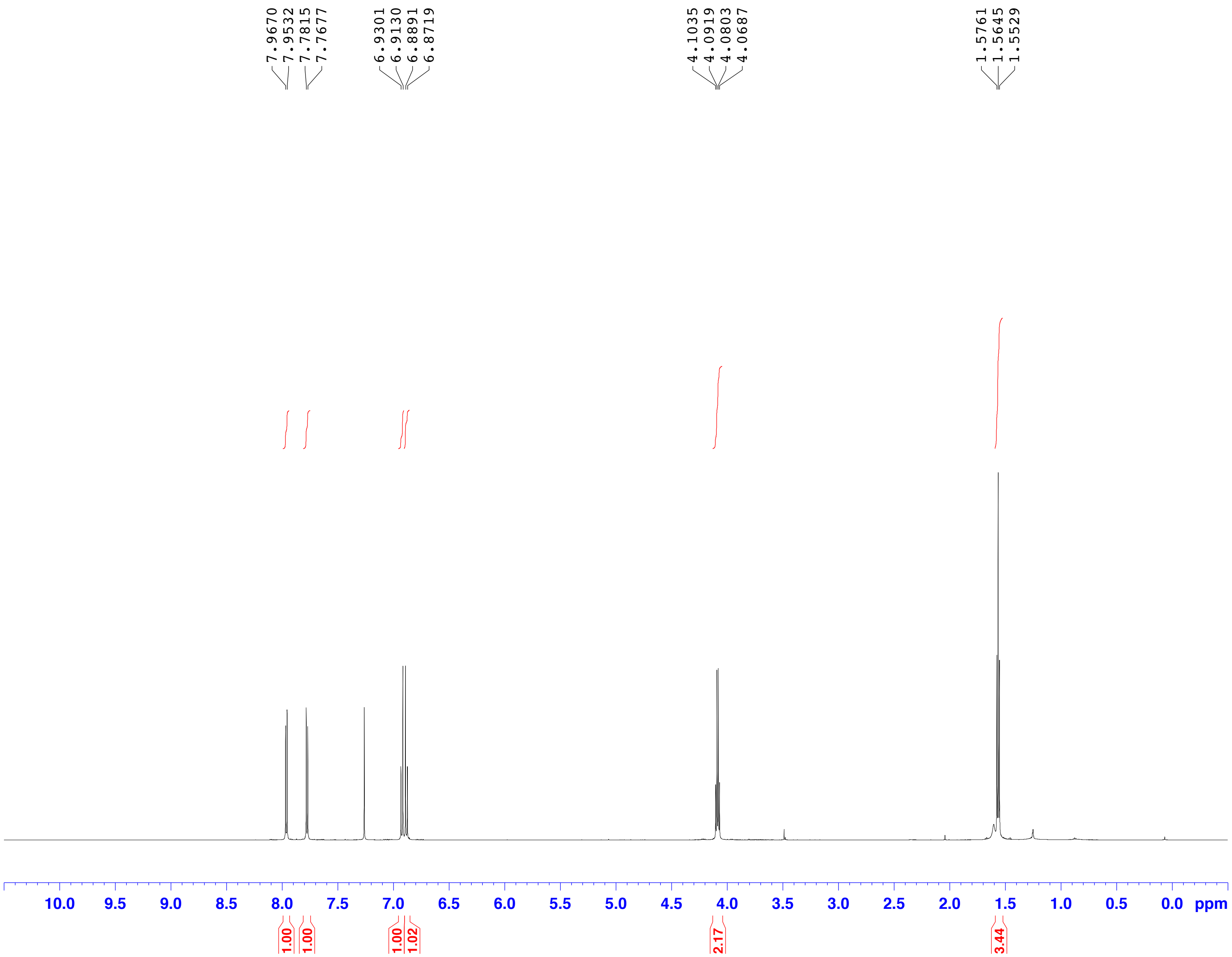


```
Current Data Parameters
NAME      YA1-1858-crude
EXPNO                 10
PROCNO                 1

F2 - Acquisition Parameters
Date_           20201118
Time               17.01
INSTRUM            spect
PROBHD   5 mm CPPBBO BB
PULPROG             zg30
TD                 65536
SOLVENT            CDCl3
NS                    16
DS                     2
SWH            12019.230 Hz
FIDRES          0.183399 Hz
AQ             2.7262976 sec
RG                 31.94
DW                41.600 use
DE                 10.00 use
TE                 298.2 K
D1            1.00000000 sec
TD0                    1

======== CHANNEL f1 =======
SFO1         600.1337060 MHz
NUC1                  1H
P1                 12.00 use
PLW1         21.00000000 W

F2 - Processing parameters
SI                 65536
SF           600.1300146 MHz
WDW                   EM
SSB      0
LB                  0.30 Hz
GB       0
PC                  1.00
```

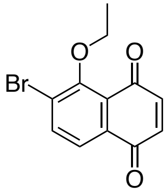

9d
(crude)

^{13}C NMR (150 MHz, $CDCl_3$)

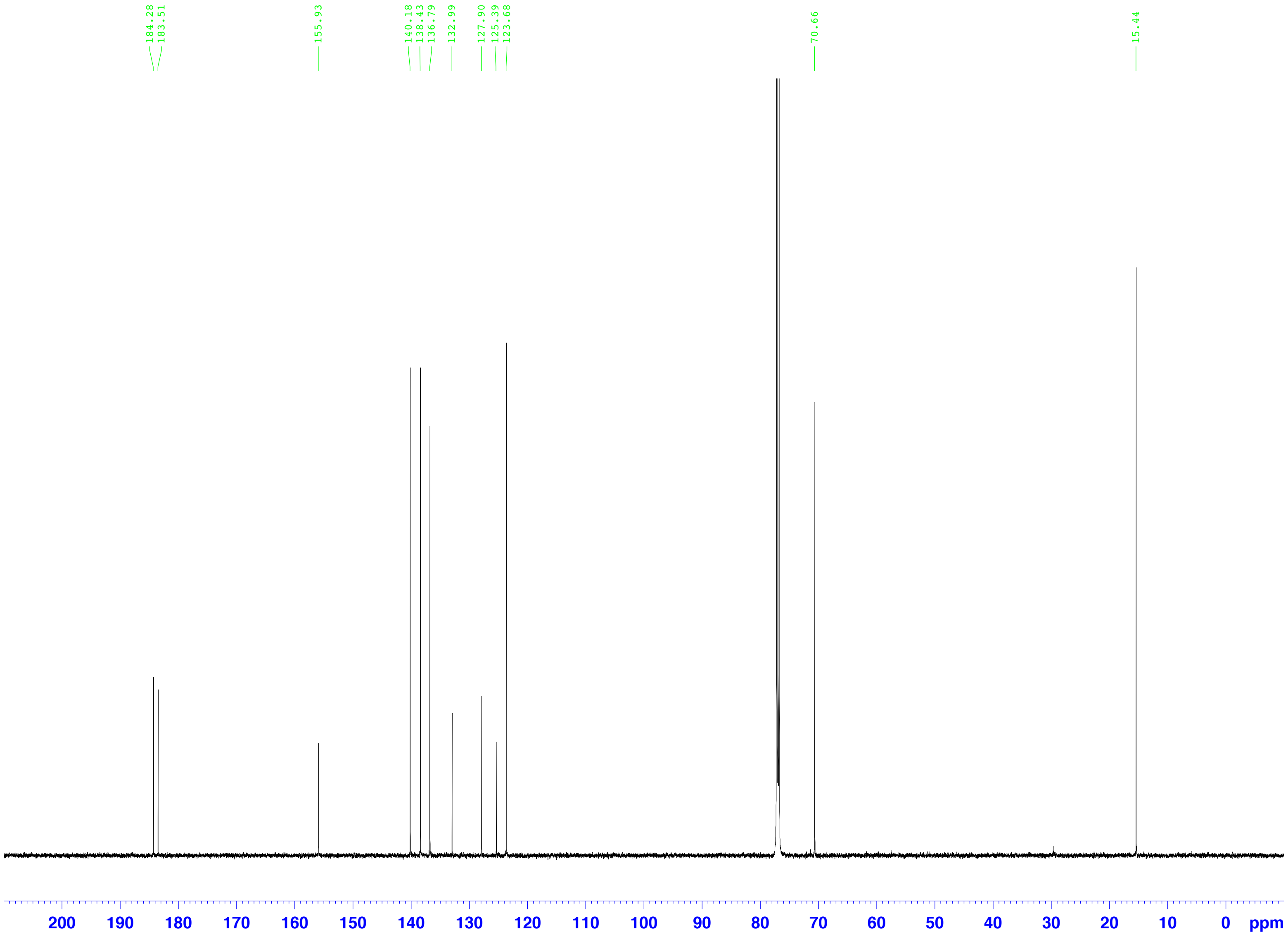


```
Current Data Parameters
NAME      YA1-1858-crude
EXPNO                 11
PROCNO                 1

F2 - Acquisition Parameters
Date_           20201119
Time                2.57
INSTRUM            spect
PROBHD    5 mm CPPBBO BB
PULPROG          zgpg30
TD                 65536
SOLVENT            CDCl3
NS                  2400
DS                     4
SWH            36057.691 Hz
FIDRES          0.550197 Hz
AQ             0.9087659 sec
RG                175.56
DW                13.867 use
DE                 18.00 use
TE                 298.2 K
D1            2.00000000 sec
D11           0.03000000 sec
TD0                    1

======== CHANNEL f1 =======
SFO1         150.9178981 MHz
NUC1                 13C
P1                 10.00 use
PLW1         80.00000000 W

======== CHANNEL f2 =======
SFO2         600.1324005 MHz
NUC2                  1H
CPDPRG[2          waltz16
PCPD2              70.00 use
PLW2         13.43999958 W
PLW12         0.61714000 W
PLW13         0.31042001 W

F2 - Processing parameters
SI                 32768
SF           150.9028123 MHz
WDW                   EM
SSB       0
LB                  1.00 Hz
GB        0
PC                  1.40
```

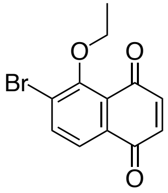

9d
(crude)

^1H NMR (600 MHz, $CDCl_3$)

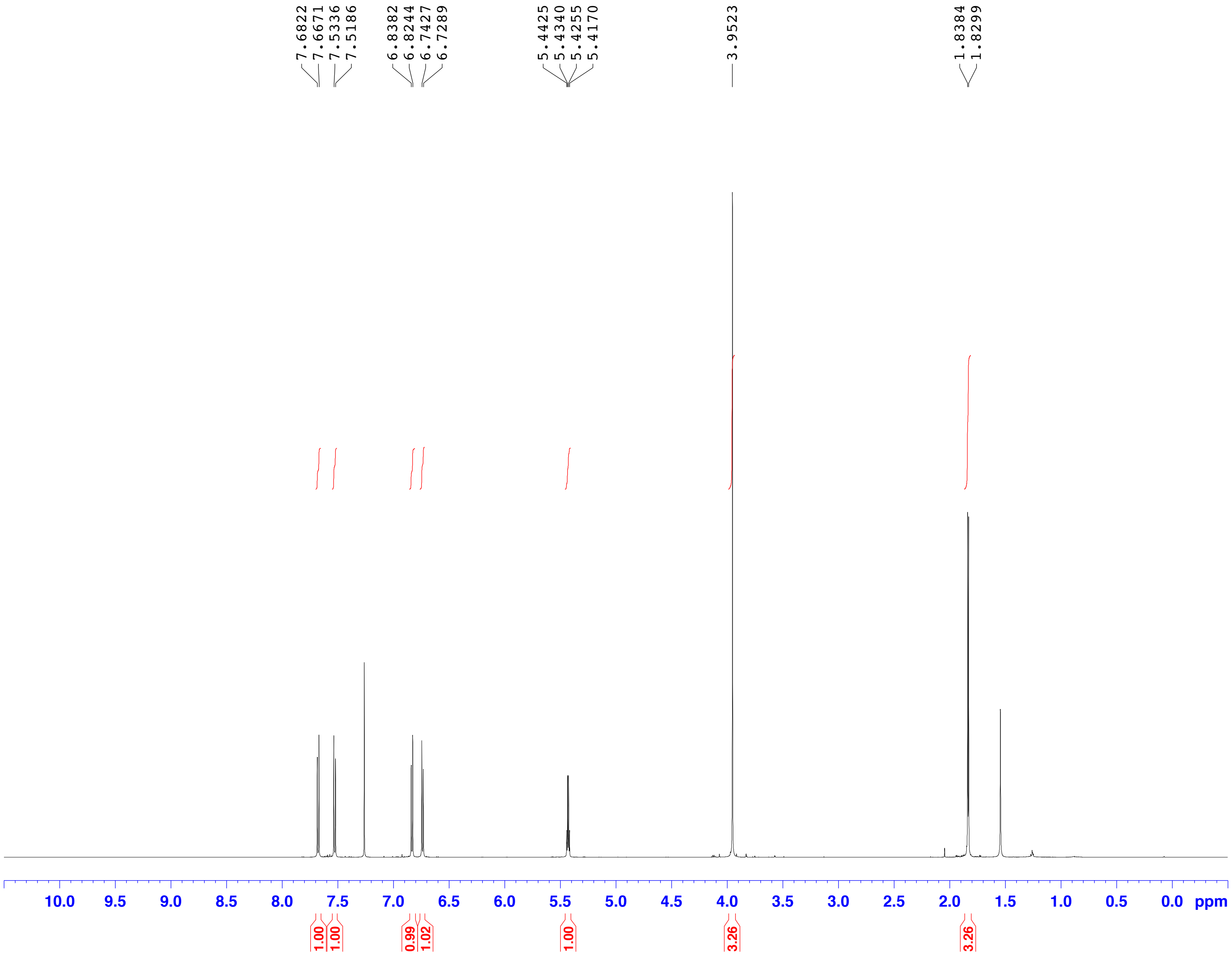


```
Current Data Parameters
NAME              YA1-1758-a
EXPNO                     10
PROCNO                     1

F2 - Acquisition Parameters
Date_               20200129
Time                   17.40
INSTRUM                spect
PROBHD    5 mm CPPBBO BB
PULPROG                 zg30
TD                     65536
SOLVENT                CDCl3
NS                        16
DS                         2
SWH              12019.230 Hz
FIDRES            0.183399 Hz
AQ               2.7262976 sec
RG                     31.94
DW                    41.600 use
DE                     10.00 use
TE                     298.0 K
D1              1.00000000 sec
TD0                        1

======== CHANNEL f1 =======
SFO1           600.1337060 MHz
NUC1                      1H
P1                     12.00 use
PLW1          21.00000000 W

F2 - Processing parameters
SI                     65536
SF             600.1300148 MHz
WDW                       EM
SSB       0
LB                      0.30 Hz
GB        0
PC                      1.00
```

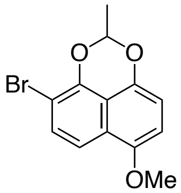

11d

^{13}C NMR (150 MHz, $CDCl_3$)

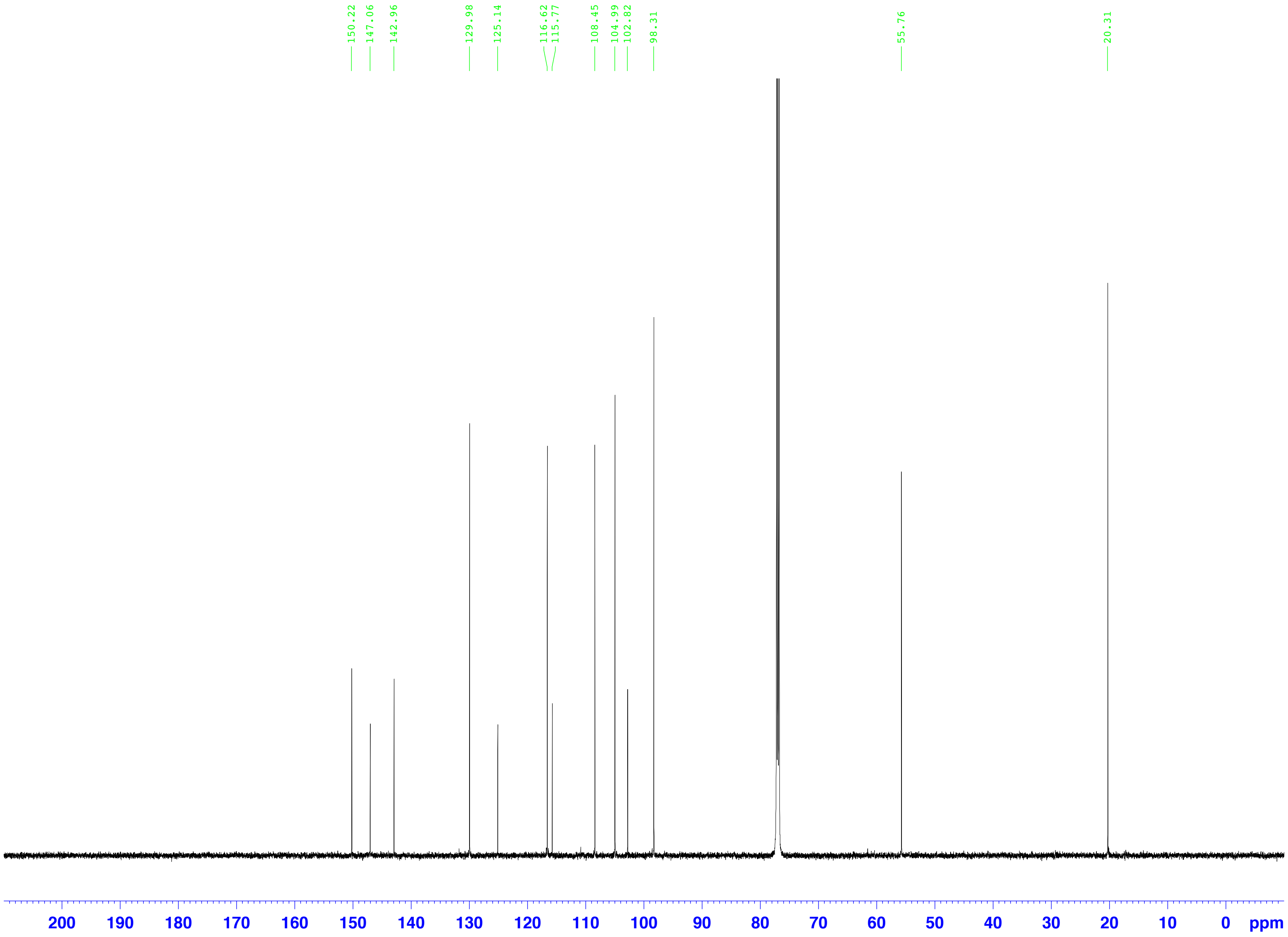


```
Current Data Parameters
NAME          YA1-1758-a
EXPNO                 11
PROCNO                 1

F2 - Acquisition Parameters
Date_           20200130
Time                5.31
INSTRUM            spect
PROBHD   5 mm CPPBBO BB
PULPROG          zgpg30
TD                65536
SOLVENT           CDCl3
NS                 3000
DS                    4
SWH          36057.691 Hz
FIDRES        0.550197 Hz
AQ           0.9087659 sec
RG              175.56
DW              13.867 use
DE               18.00 use
TE               298.2 K
D1          2.00000000 sec
D11         0.03000000 sec
TD0                  1

======== CHANNEL f1 =======
SFO1       150.9178981 MHz
NUC1               13C
P1               10.00 use
PLW1       80.00000000 W

======== CHANNEL f2 =======
SFO2       600.1324005 MHz
NUC2                1H
CPDPRG[2         waltz16
PCPD2            70.00 use
PLW2       13.43999958 W
PLW12       0.61714000 W
PLW13       0.31042001 W

F2 - Processing parameters
SI                32768
SF         150.9028121 MHz
WDW                  EM
SSB      0
LB                1.00 Hz
GB       0
PC                1.40
```

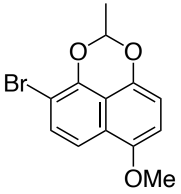

11d

^{1}H NMR (600 MHz, $CDCl_3$)

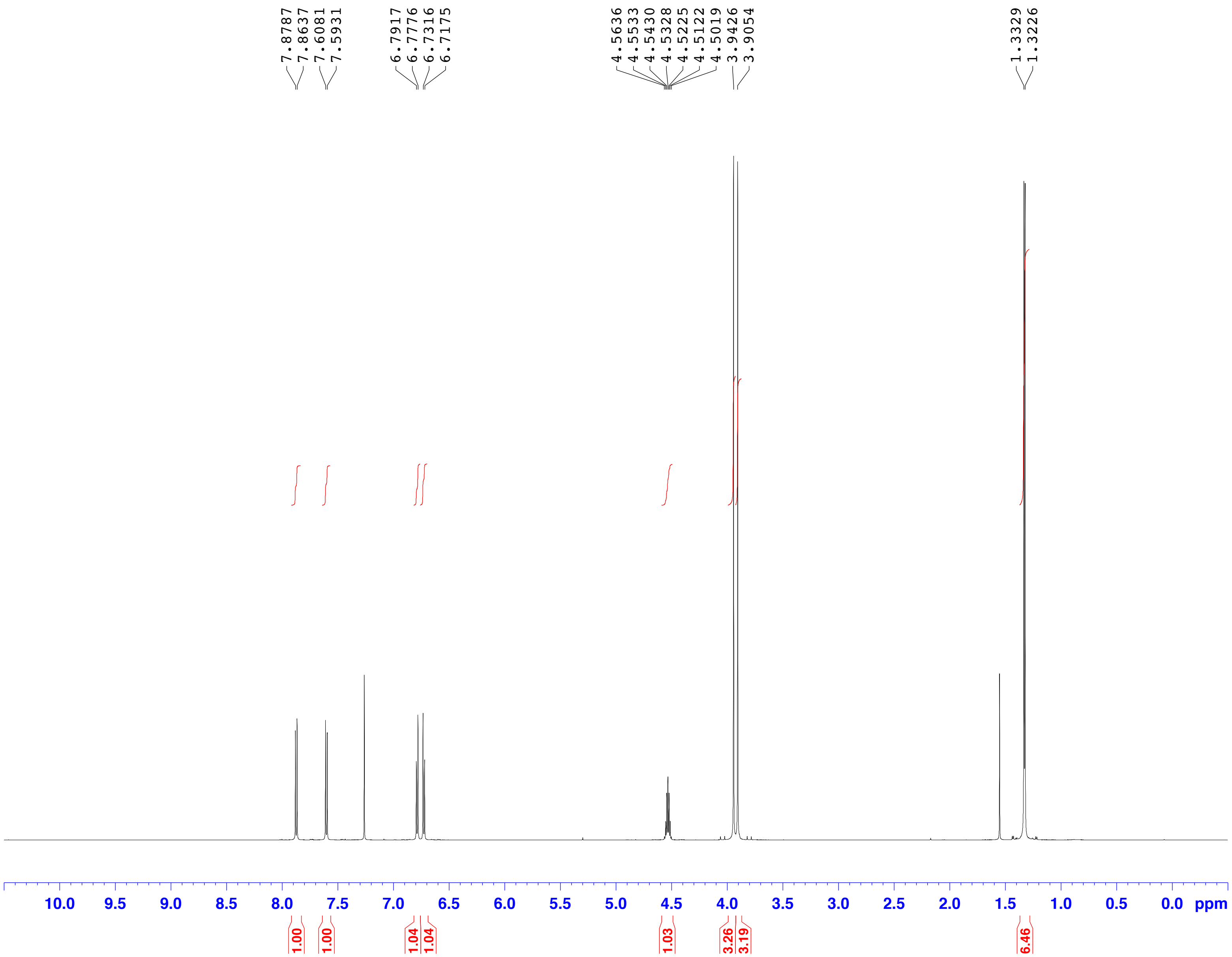

BRUKER

```
Current Data Parameters
NAME              YA1-1743-1
EXPNO                     10
PROCNO                     1

F2 - Acquisition Parameters
Date_               20200117
Time                   13.49
INSTRUM                spect
PROBHD     5 mm CPPBBO BB
PULPROG                 zg30
TD                     65536
SOLVENT                CDCl3
NS                        16
DS                         2
SWH                12019.230 Hz
FIDRES              0.183399 Hz
AQ                 2.7262976 sec
RG                      17.5
DW                    41.600 use
DE                     10.00 use
TE                     298.2 K
D1                1.00000000 sec
TD0                        1

======== CHANNEL f1 =======
SFO1             600.1337060 MHz
NUC1                      1H
P1                     12.00 use
PLW1             21.00000000 W

F2 - Processing parameters
SI                     65536
SF               600.1300148 MHz
WDW                       EM
SSB      0
LB                      0.30 Hz
GB       0
PC                      1.00
```

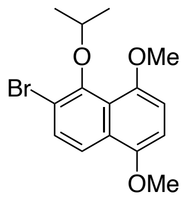

13e

^{13}C NMR (150 MHz, $CDCl_3$)

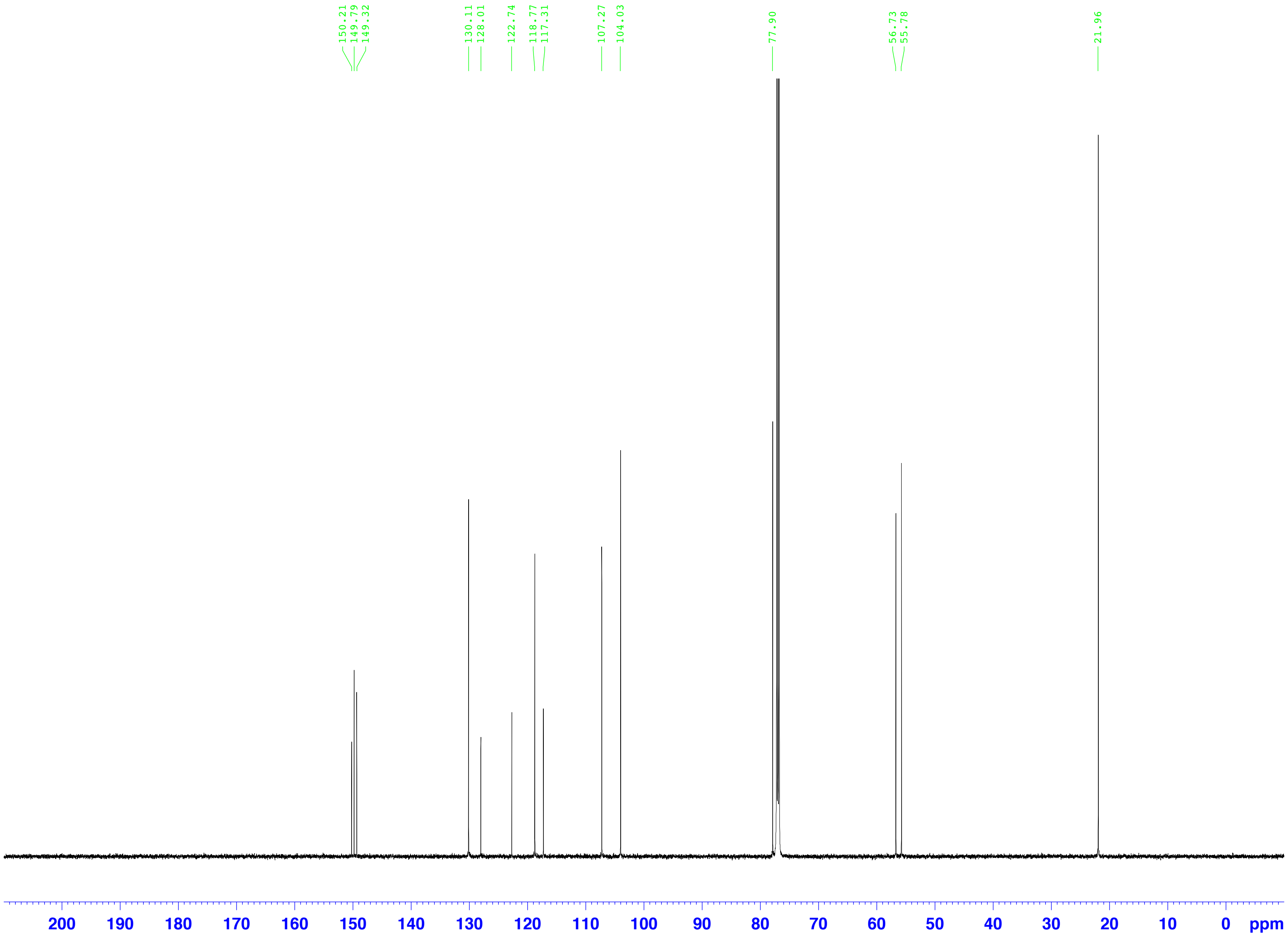

BRUKER

```
Current Data Parameters
NAME              YA1-1743-1
EXPNO                     11
PROCNO                     1

F2 - Acquisition Parameters
Date_               20200118
Time                   14.47
INSTRUM                spect
PROBHD     5 mm CPPBBO BB
PULPROG              zgpg30
TD                     65536
SOLVENT                CDCl3
NS                      3000
DS                         4
SWH               36057.691 Hz
FIDRES             0.550197 Hz
AQ                0.9087659 sec
RG                    175.56
DW                    13.867 use
DE                     18.00 use
TE                     298.2 K
D1                2.00000000 sec
D11               0.03000000 sec
TD0                        1

======== CHANNEL f1 =======
SFO1            150.9178981 MHz
NUC1                     13C
P1                     10.00 use
PLW1             80.00000000 W

======== CHANNEL f2 =======
SFO2            600.1324005 MHz
NUC2                      1H
CPDPRG[2             waltz16
PCPD2                  70.00 use
PLW2             13.43999958 W
PLW12             0.61714000 W
PLW13             0.31042001 W

F2 - Processing parameters
SI                     32768
SF              150.9028125 MHz
WDW                       EM
SSB       0
LB                      1.00 Hz
GB        0
PC                      1.40
```

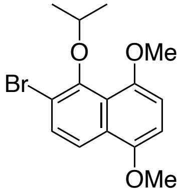

13e

^{1}H NMR (600 MHz, $CDCl_3$)

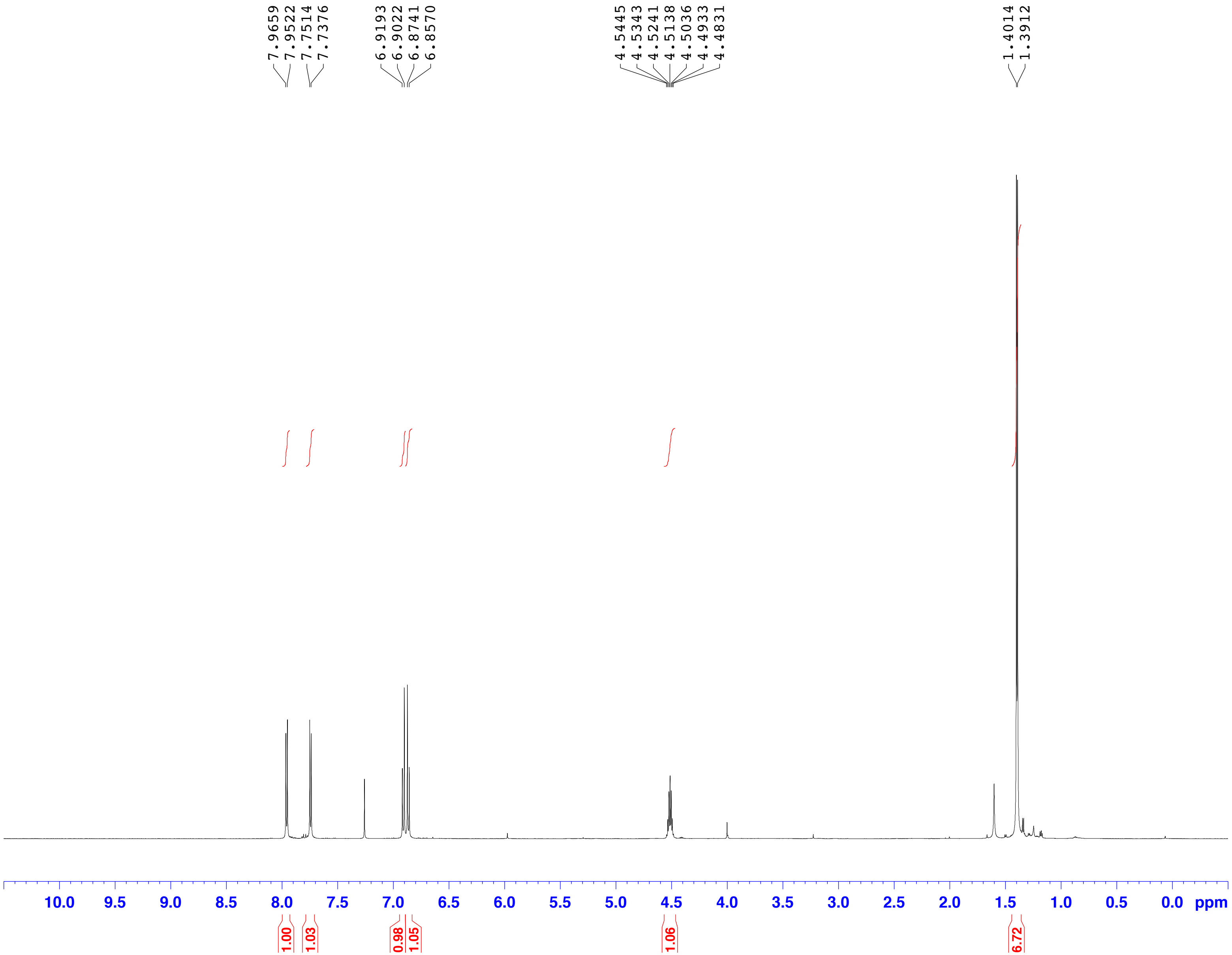


```
Current Data Parameters
NAME       YA1-1873-crude
EXPNO                  10
PROCNO                  1

F2 - Acquisition Parameters
Date_            20201217
Time                21.17
INSTRUM             spect
PROBHD    5 mm CPPBBO BB
PULPROG              zg30
TD                  65536
SOLVENT             CDCl3
NS                     16
DS                      2
SWH             12019.230 Hz
FIDRES           0.183399 Hz
AQ              2.7262976 sec
RG                  31.94
DW                 41.600 use
DE                  10.00 use
TE                  298.2 K
D1             1.00000000 sec
TD0                     1

======== CHANNEL f1 =======
SFO1          600.1337060 MHz
NUC1                   1H
P1                  12.00 use
PLW1          21.00000000 W

F2 - Processing parameters
SI                  65536
SF            600.1300153 MHz
WDW                    EM
SSB       0
LB                   0.30 Hz
GB        0
PC                   1.00
```

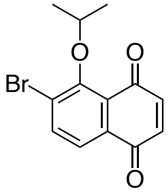

9e
(crude)

^{13}C NMR (150 MHz, $CDCl_3$)

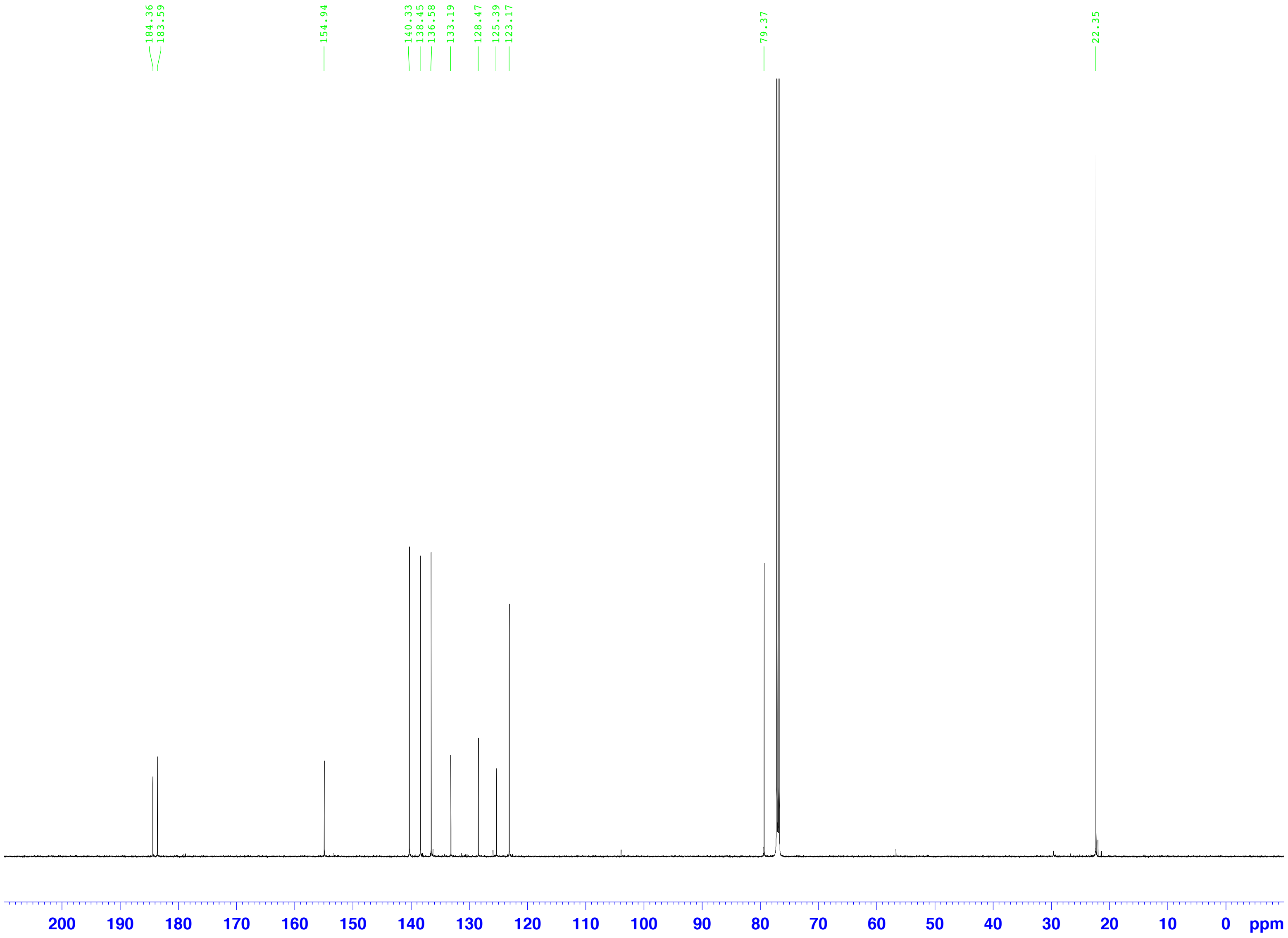


```
Current Data Parameters
NAME      YA1-1873-crude
EXPNO                 11
PROCNO                 1

F2 - Acquisition Parameters
Date_           20201218
Time                0.01
INSTRUM            spect
PROBHD    5 mm CPPBBO BB
PULPROG          zgpg30
TD                 65536
SOLVENT            CDCl3
NS                  2400
DS                     4
SWH            36057.691 Hz
FIDRES          0.550197 Hz
AQ             0.9087659 sec
RG                175.56
DW                13.867 use
DE                 18.00 use
TE                 298.0 K
D1            2.00000000 sec
D11           0.03000000 sec
TD0                    1

======== CHANNEL f1 =======
SFO1         150.9178981 MHz
NUC1                 13C
P1                 10.00 use
PLW1         80.00000000 W

======== CHANNEL f2 =======
SFO2         600.1324005 MHz
NUC2                  1H
CPDPRG[2         waltz16
PCPD2              70.00 use
PLW2         13.43999958 W
PLW12         0.61714000 W
PLW13         0.31042001 W

F2 - Processing parameters
SI                 32768
SF           150.9028140 MHz
WDW                   EM
SSB       0
LB                  1.00 Hz
GB        0
PC                  1.40
```

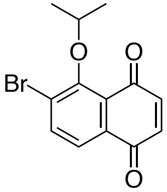

9e
(crude)

^{1}H NMR (600 MHz, $CDCl_3$)

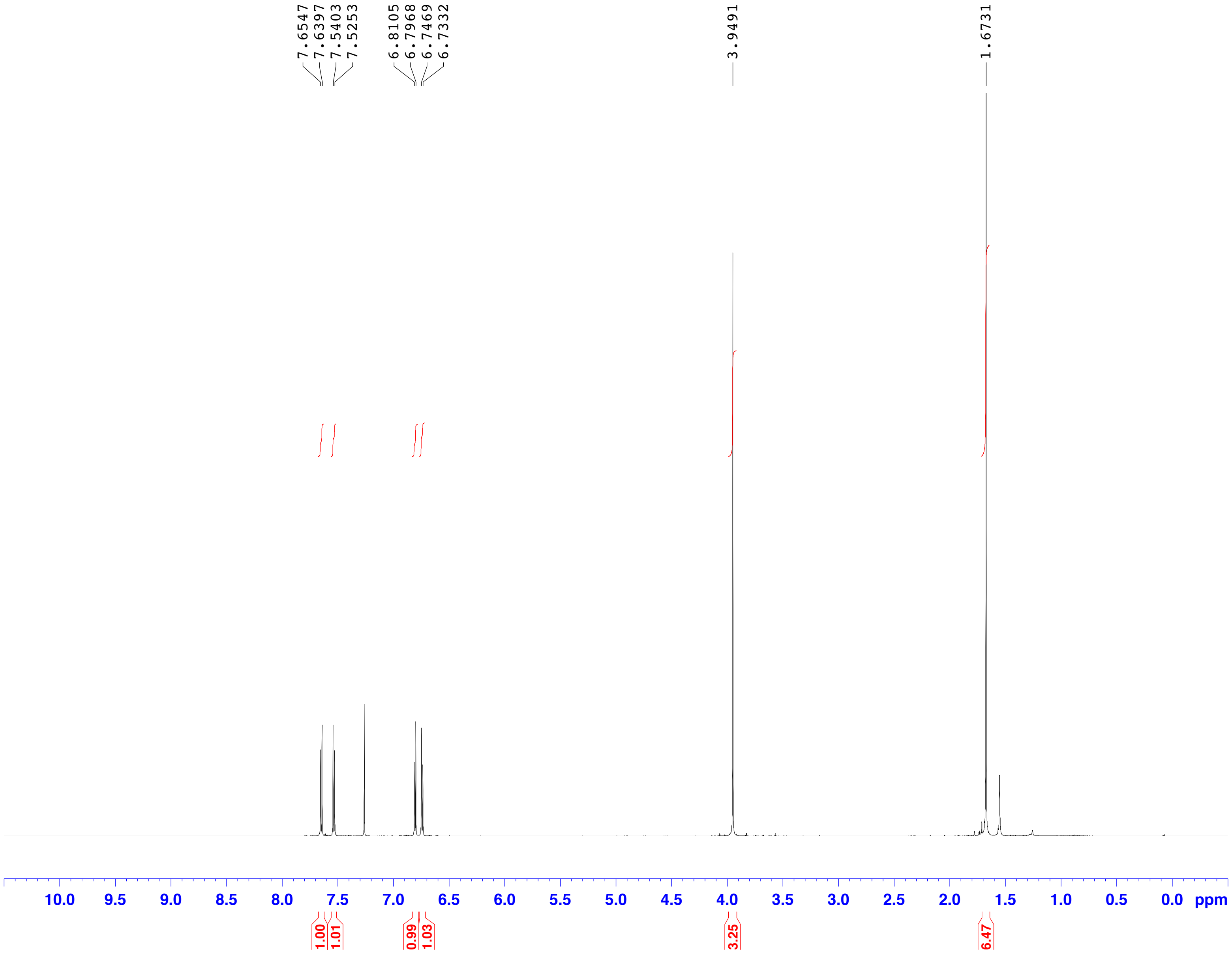

BRUKER

```
Current Data Parameters
NAME          YA1-1756-1-1
EXPNO                   10
PROCNO                   1

F2 - Acquisition Parameters
Date_             20200129
Time                 14.33
INSTRUM              spect
PROBHD    5 mm CPPBBO BB
PULPROG               zg30
TD                   65536
SOLVENT              CDCl3
NS                      16
DS                       2
SWH              12019.230 Hz
FIDRES            0.183399 Hz
AQ               2.7262976 sec
RG                   31.94
DW                  41.600 use
DE                   10.00 use
TE                   298.2 K
D1              1.00000000 sec
TD0                      1

======== CHANNEL f1 =======
SFO1           600.1337060 MHz
NUC1                    1H
P1                   12.00 use
PLW1           21.00000000 W

F2 - Processing parameters
SI                   65536
SF             600.1300148 MHz
WDW                     EM
SSB       0
LB                    0.30 Hz
GB        0
PC                    1.00
```

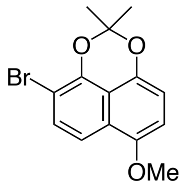

11e

^{13}C NMR (150 MHz, $CDCl_3$)

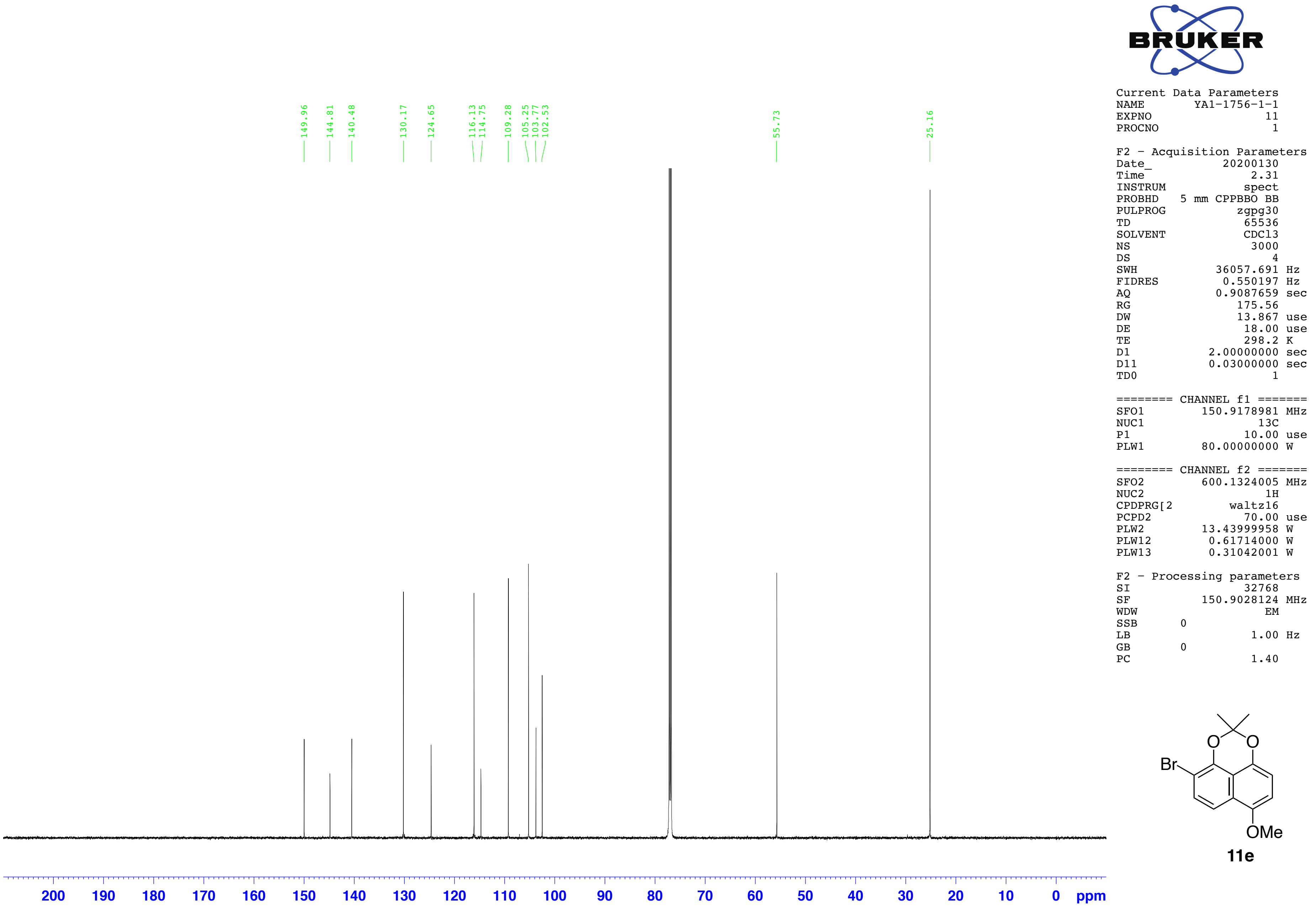

11e

^{1}H NMR (600 MHz, $CDCl_3$)

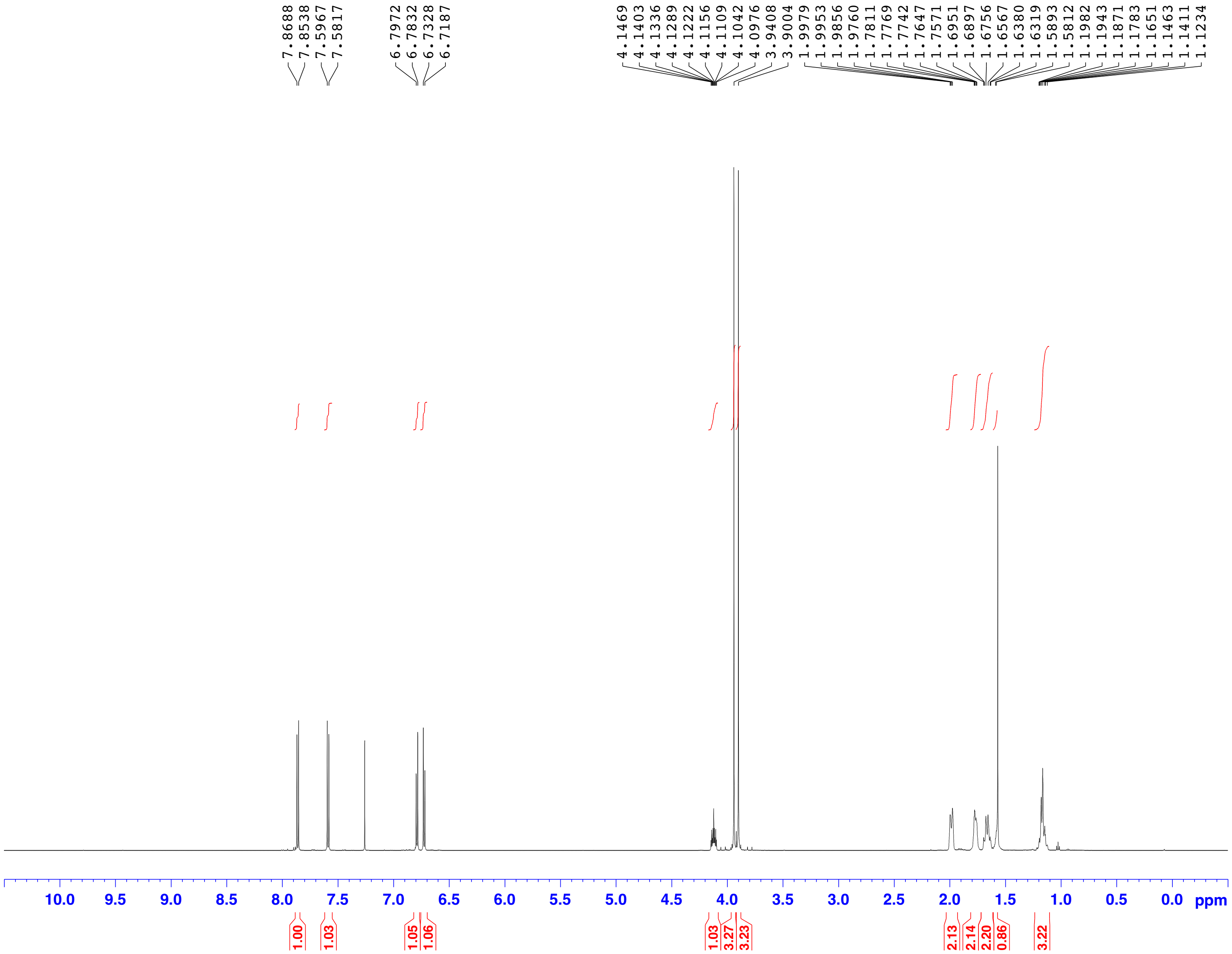


```
Current Data Parameters
NAME              YA1-2064-1
EXPNO                     31
PROCNO                     1

F2 - Acquisition Parameters
Date_               20220613
Time                   20.57
INSTRUM                spect
PROBHD   5 mm CPPBBO BB
PULPROG                 zg30
TD                     65536
SOLVENT                CDCl3
NS                        16
DS                         2
SWH                12019.230 Hz
FIDRES              0.183399 Hz
AQ                 2.7262976 sec
RG                     18.96
DW                    41.600 usec
DE                     10.00 usec
TE                     298.3 K
D1                1.00000000 sec
TD0                        1

======== CHANNEL f1 ========
SFO1             600.1337060 MHz
NUC1                      1H
P1                     12.00 usec
PLW1             21.00000000 W

F2 - Processing parameters
SI                     65536
SF               600.1300142 MHz
WDW                       EM
SSB                        0
LB                      0.30 Hz
GB                         0
PC                      1.00
```

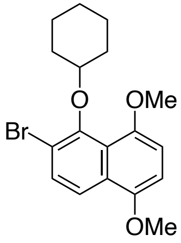

13f

^{13}C NMR (150 MHz, $CDCl_3$)

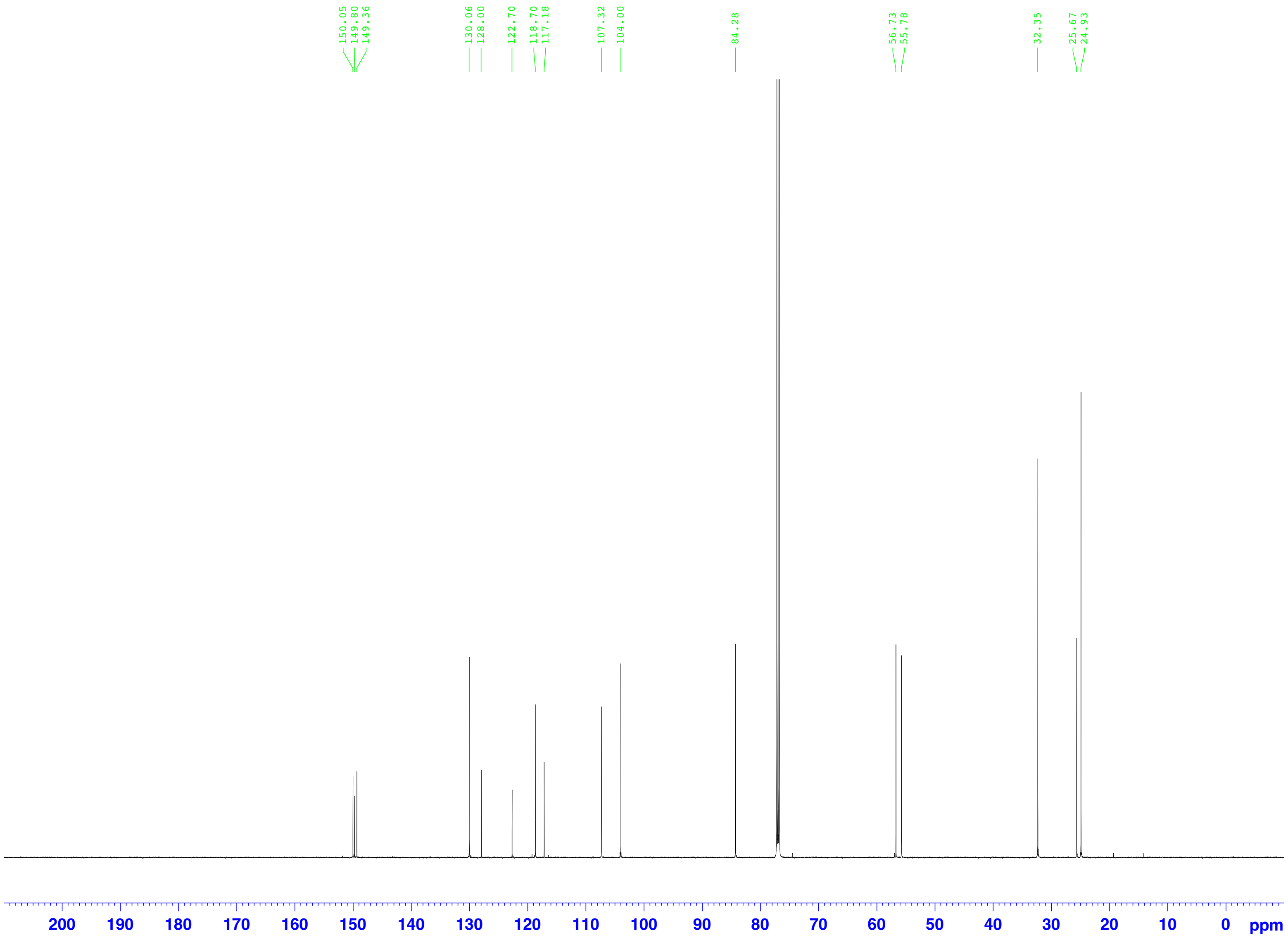

BRUKER

```
Current Data Parameters
NAME            YA1-2064-1
EXPNO                   41
PROCNO                   1

F2 - Acquisition Parameters
Date_             20220615
Time                  1.00
INSTRUM              spect
PROBHD    5 mm CPPBBO BB
PULPROG             zgpg30
TD                   65536
SOLVENT              CDCl3
NS                    3600
DS                       4
SWH             36057.691 Hz
FIDRES           0.550197 Hz
AQ              0.9087659 sec
RG                  175.56
DW                  13.867 usec
DE                   18.00 usec
TE                   298.2 K
D1             2.00000000 sec
D11            0.03000000 sec
TD0                      1

======== CHANNEL f1 ========
SFO1         150.9178981 MHz
NUC1                   13C
P1                   10.00 usec
PLW1          80.00000000 W

======== CHANNEL f2 ========
SFO2         600.1324005 MHz
NUC2                    1H
CPDPRG[2           waltz16
PCPD2                70.00 usec
PLW2          13.43999958 W
PLW12          0.61714000 W
PLW13          0.31042001 W

F2 - Processing parameters
SI                   32768
SF           150.9028133 MHz
WDW                     EM
SSB                      0
LB                    1.00 Hz
GB                       0
PC                    1.40
```

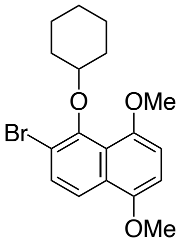

13f

^{1}H NMR (600 MHz, $CDCl_3$)

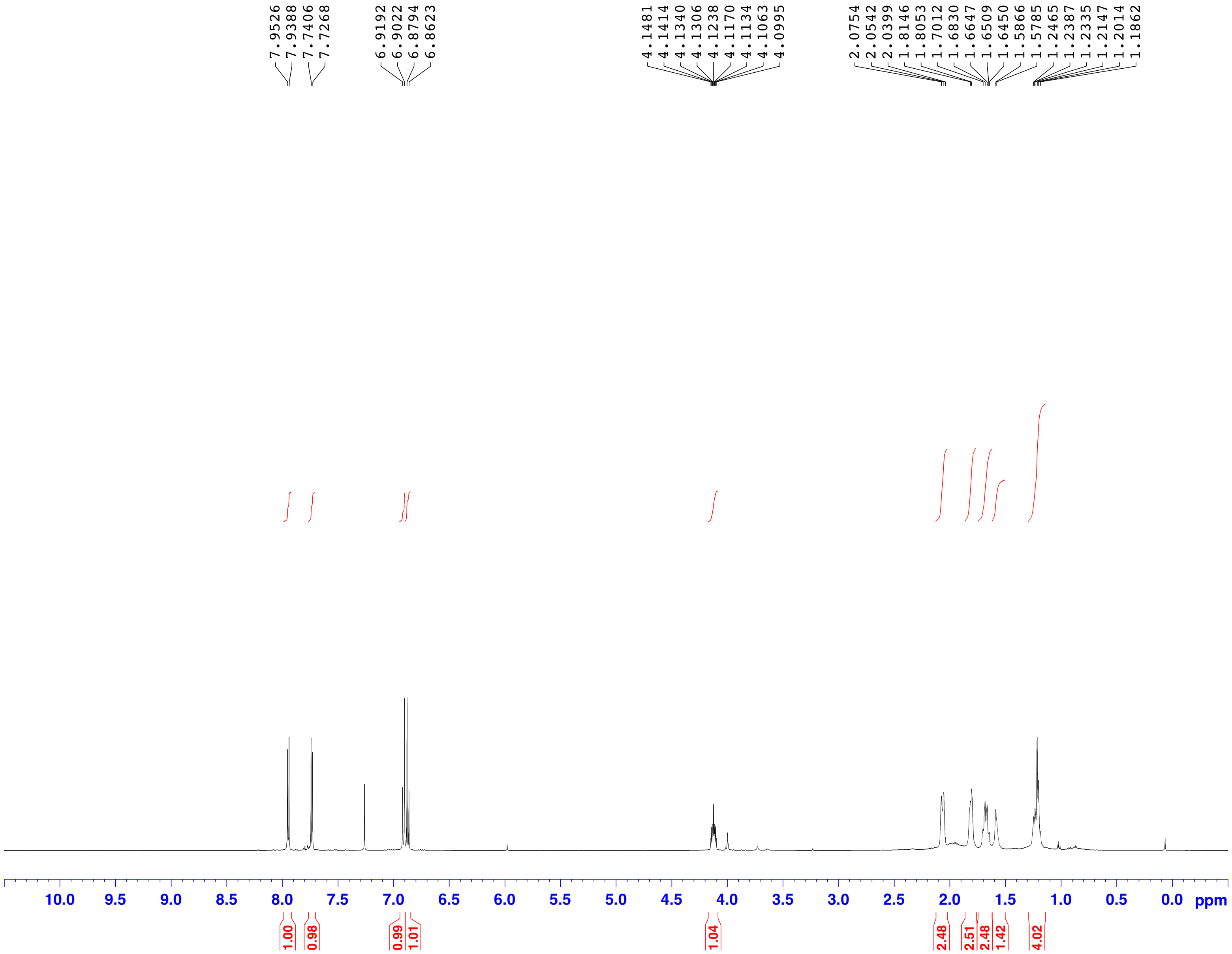


```
Current Data Parameters
NAME      YA1-2070-crude
EXPNO                 10
PROCNO                 1

F2 - Acquisition Parameters
Date_           20220618
Time               17.04
INSTRUM            spect
PROBHD    5 mm CPPBBO BB
PULPROG             zg30
TD                 65536
SOLVENT            CDCl3
NS                    16
DS                     2
SWH            12019.230 Hz
FIDRES          0.183399 Hz
AQ             2.7262976 sec
RG                 31.94
DW                41.600 usec
DE                 10.00 usec
TE                 298.1 K
D1            1.00000000 sec
TD0                    1

======== CHANNEL f1 ========
SFO1         600.1337060 MHz
NUC1                  1H
P1                 12.00 usec
PLW1         21.00000000 W

F2 - Processing parameters
SI                 65536
SF           600.1300149 MHz
WDW                   EM
SSB                    0
LB                  0.30 Hz
GB                     0
PC                  1.00
```

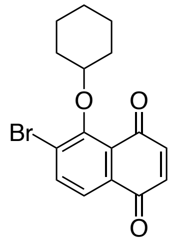

9f
(crude)

^{13}C NMR (150 MHz, $CDCl_3$)

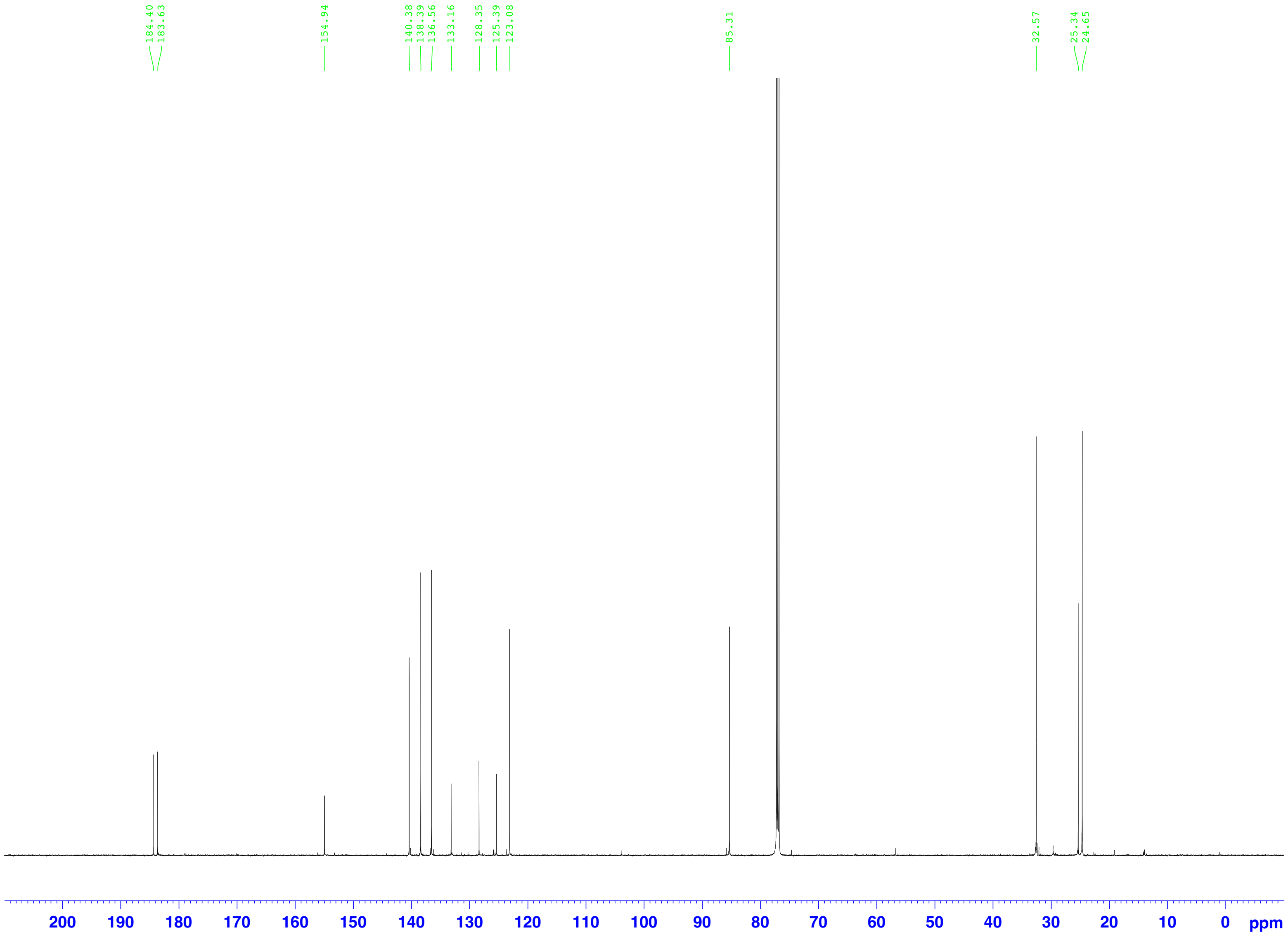


```
Current Data Parameters
NAME      YA1-2070-crude
EXPNO                 12
PROCNO                 1

F2 - Acquisition Parameters
Date_           20220619
Time                1.01
INSTRUM            spect
PROBHD    5 mm CPPBBO BB
PULPROG           zgpg30
TD                 65536
SOLVENT            CDCl3
NS                  3600
DS                     4
SWH            36057.691 Hz
FIDRES          0.550197 Hz
AQ             0.9087659 sec
RG                175.56
DW                13.867 usec
DE                 18.00 usec
TE                 298.2 K
D1            2.00000000 sec
D11           0.03000000 sec
TD0                    1

======== CHANNEL f1 ========
SFO1         150.9178981 MHz
NUC1                 13C
P1                 10.00 usec
PLW1         80.00000000 W

======== CHANNEL f2 ========
SFO2         600.1324005 MHz
NUC2                  1H
CPDPRG[2          waltz16
PCPD2              70.00 usec
PLW2         13.43999958 W
PLW12         0.61714000 W
PLW13         0.31042001 W

F2 - Processing parameters
SI                 32768
SF           150.9028136 MHz
WDW                   EM
SSB                    0
LB                  1.00 Hz
GB                     0
PC                  1.40
```

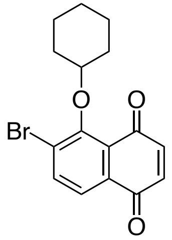

9f
(crude)

^{1}H NMR (600 MHz, $CDCl_3$)

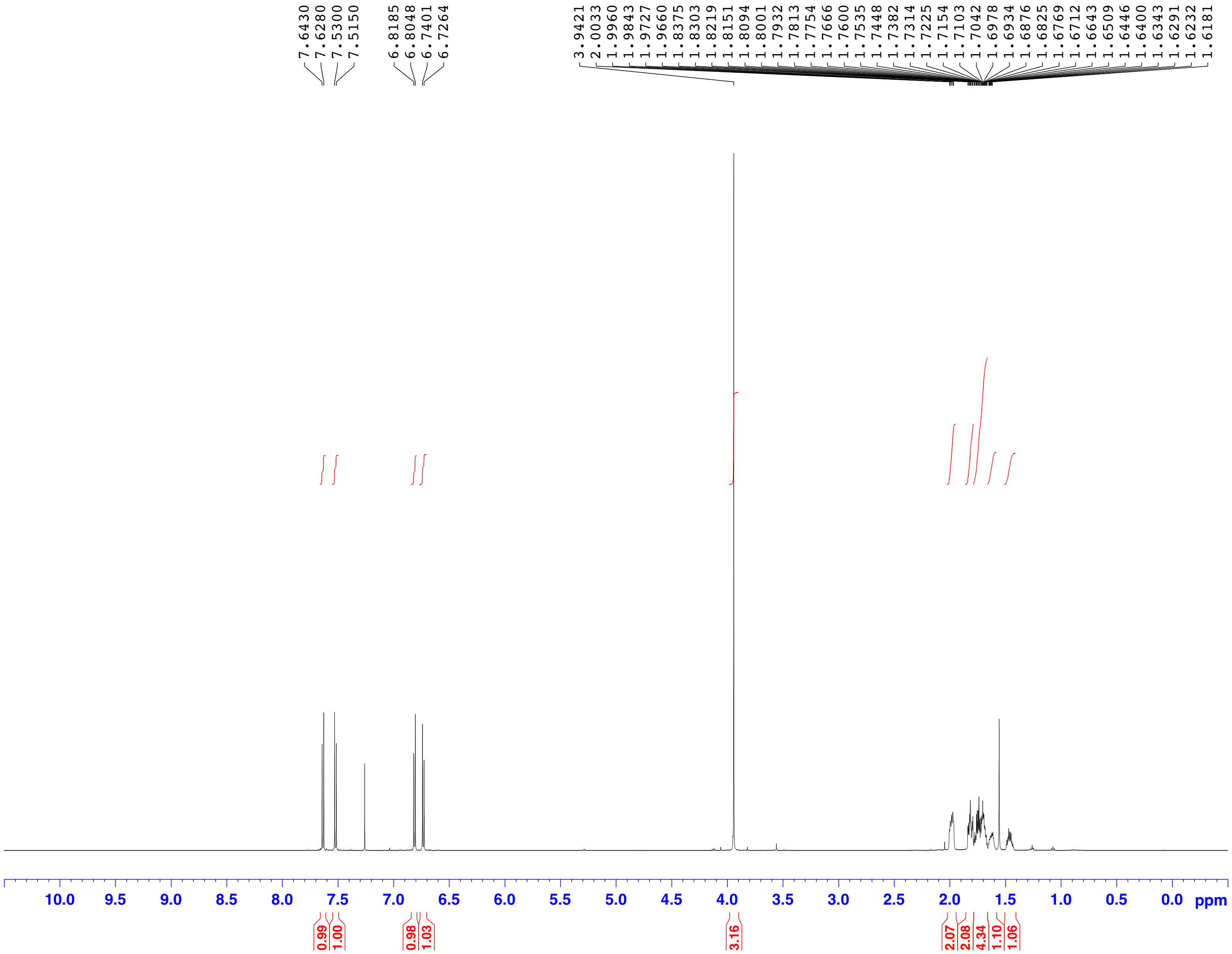

Current Data Parameters
NAME YA1-2068-1-2
EXPNO 10
PROCNO 1

F2 - Acquisition Parameters
Date_ 20220615
Time 16.30
INSTRUM spect
PROBHD 5 mm CPPBBO BB
PULPROG zg30
TD 65536
SOLVENT CDCl3
NS 16
DS 2
SWH 12019.230 Hz
FIDRES 0.183399 Hz
AQ 2.7262976 sec
RG 31.94
DW 41.600 usec
DE 10.00 usec
TE 298.3 K
D1 1.00000000 sec
TD0 1

======== CHANNEL f1 ========
SFO1 600.1337060 MHz
NUC1 1H
P1 12.00 usec
PLW1 21.00000000 W

F2 - Processing parameters
SI 65536
SF 600.1300144 MHz
WDW EM
SSB 0
LB 0.30 Hz
GB 0
PC 1.00

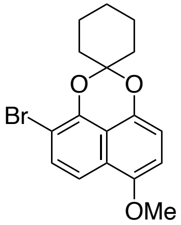

11f

^{13}C NMR (150 MHz, $CDCl_3$)

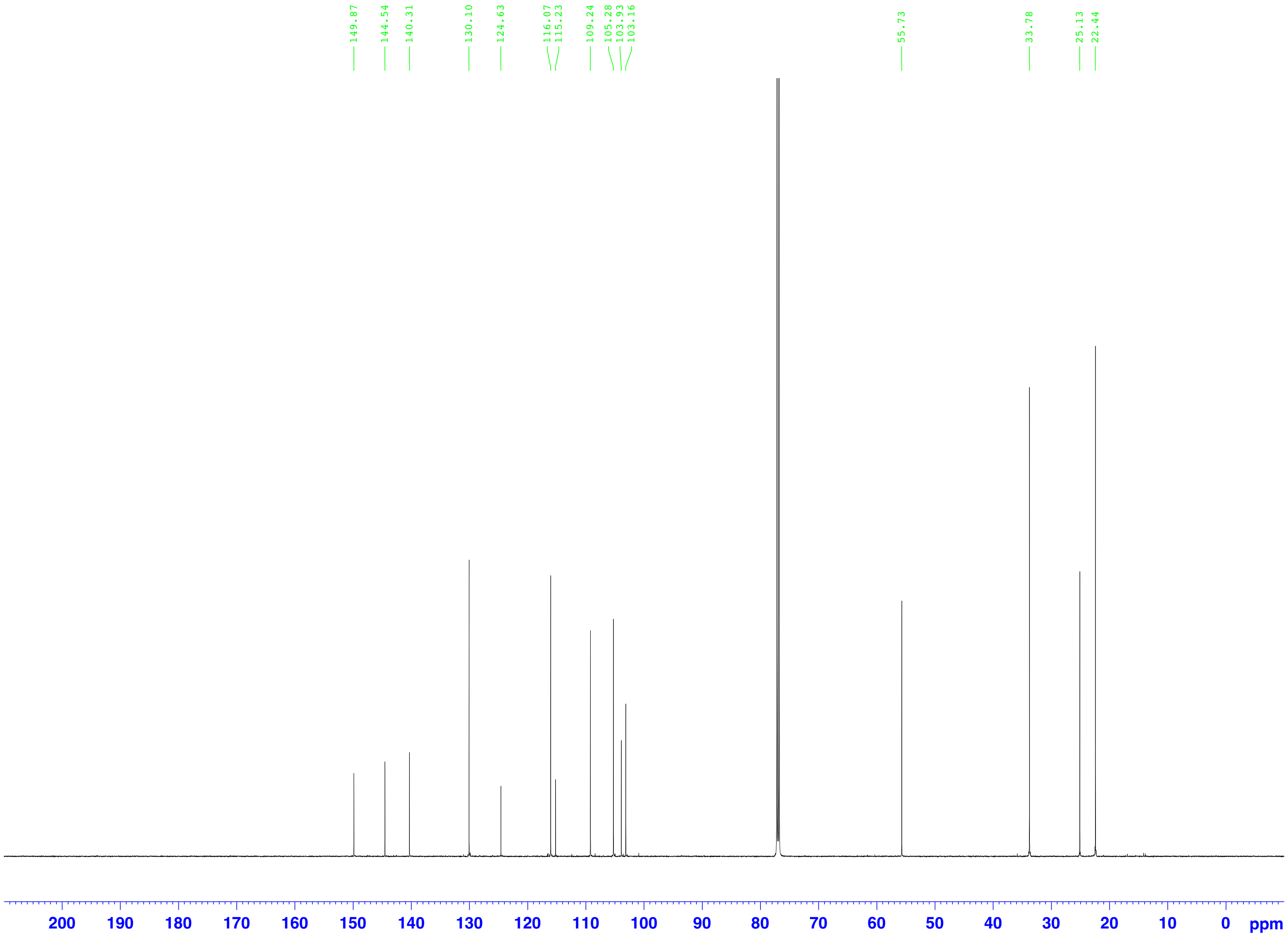


```
Current Data Parameters
NAME          YA1-2068-1-2
EXPNO                   11
PROCNO                   1

F2 - Acquisition Parameters
Date_             20220616
Time                  4.00
INSTRUM              spect
PROBHD    5 mm CPPBBO BB
PULPROG            zgpg30
TD                  65536
SOLVENT             CDCl3
NS                   3600
DS                      4
SWH             36057.691 Hz
FIDRES           0.550197 Hz
AQ              0.9087659 sec
RG                 175.56
DW                 13.867 usec
DE                  18.00 usec
TE                  298.2 K
D1             2.00000000 sec
D11            0.03000000 sec
TD0                     1

======== CHANNEL f1 ========
SFO1          150.9178981 MHz
NUC1                  13C
P1                  10.00 usec
PLW1          80.00000000 W

======== CHANNEL f2 ========
SFO2          600.1324005 MHz
NUC2                   1H
CPDPRG[2          waltz16
PCPD2               70.00 usec
PLW2          13.43999958 W
PLW12          0.61714000 W
PLW13          0.31042001 W

F2 - Processing parameters
SI                  32768
SF            150.9028133 MHz
WDW                    EM
SSB                     0
LB                   1.00 Hz
GB                      0
PC                   1.40
```

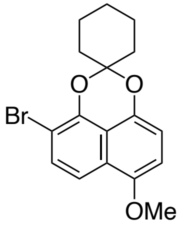

11f

^{1}H NMR (600 MHz, $CDCl_3$)

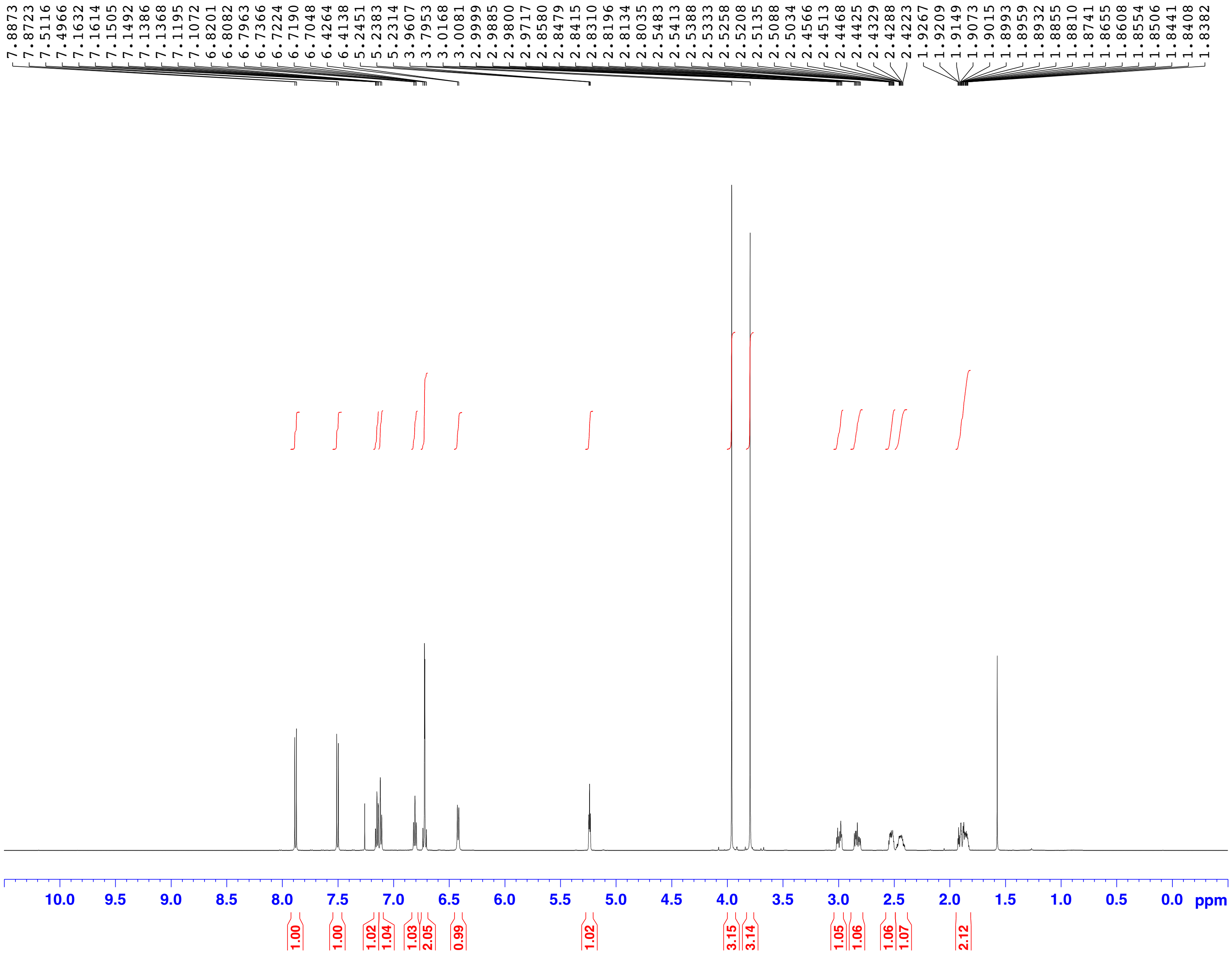


```
Current Data Parameters
NAME          YA1-2069-2-1
EXPNO                   20
PROCNO                   1

F2 - Acquisition Parameters
Date_             20220617
Time                 13.13
INSTRUM              spect
PROBHD     5 mm CPPBBO BB
PULPROG               zg30
TD                   65536
SOLVENT              CDCl3
NS                      16
DS                       2
SWH              12019.230 Hz
FIDRES            0.183399 Hz
AQ               2.7262976 sec
RG                    17.5
DW                  41.600 usec
DE                   10.00 usec
TE                   298.3 K
D1              1.00000000 sec
TD0                      1

======== CHANNEL f1 ========
SFO1           600.1337060 MHz
NUC1                    1H
P1                   12.00 usec
PLW1           21.00000000 W

F2 - Processing parameters
SI                   65536
SF             600.1300144 MHz
WDW                     EM
SSB                      0
LB                    0.30 Hz
GB                       0
PC                    1.00
```

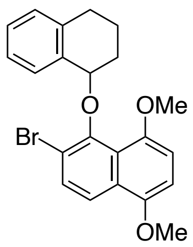

13g

^{13}C NMR (150 MHz, $CDCl_3$)

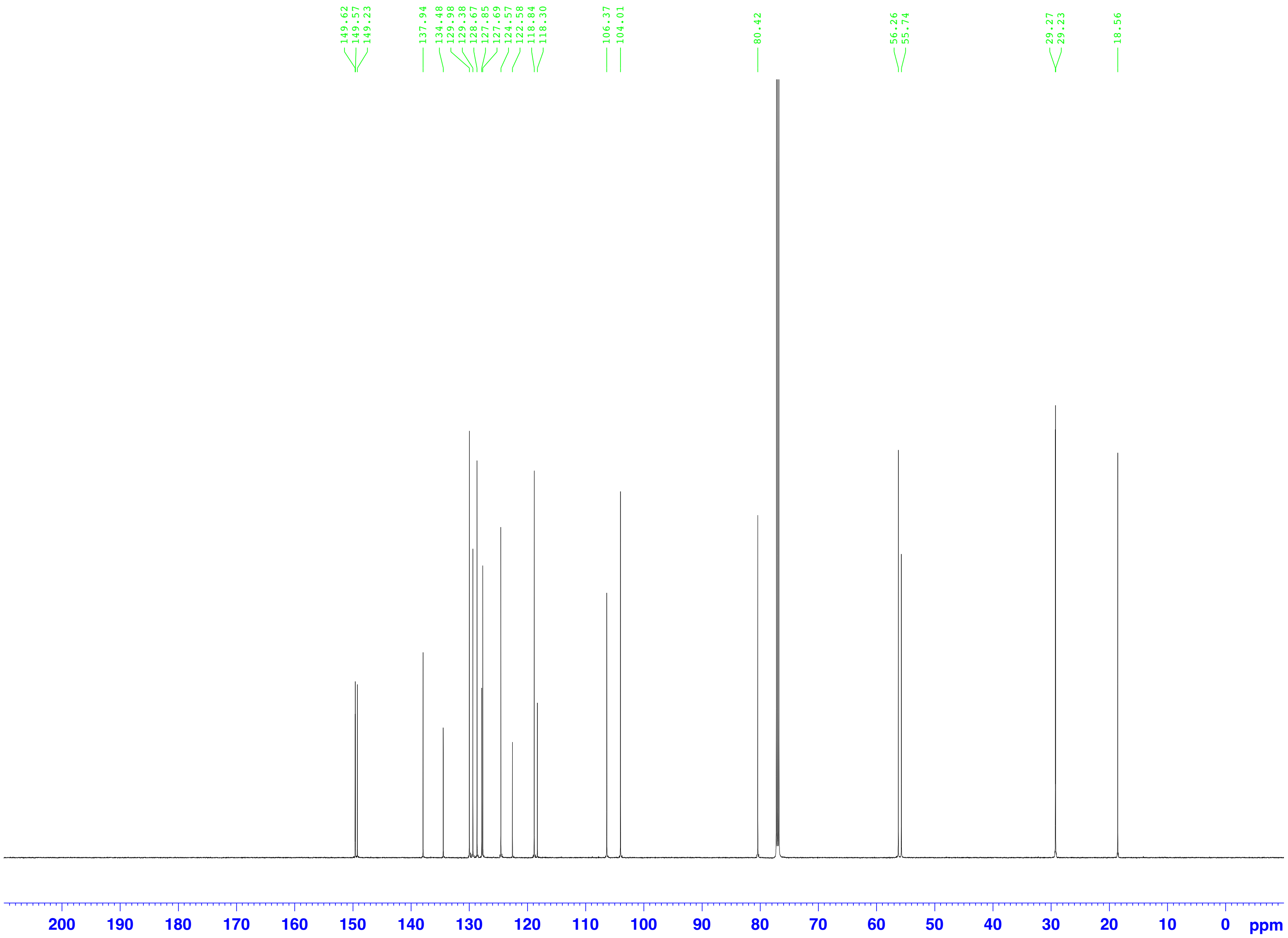


```
Current Data Parameters
NAME          YA1-2069-2-1
EXPNO                   22
PROCNO                   1

F2 - Acquisition Parameters
Date_             20220619
Time                  5.41
INSTRUM              spect
PROBHD    5 mm CPPBBO BB
PULPROG            zgpg30
TD                  65536
SOLVENT             CDCl3
NS                   3600
DS                      4
SWH             36057.691 Hz
FIDRES           0.550197 Hz
AQ              0.9087659 sec
RG                 175.56
DW                 13.867 usec
DE                  18.00 usec
TE                  298.3 K
D1             2.00000000 sec
D11            0.03000000 sec
TD0                     1

======== CHANNEL f1 ========
SFO1          150.9178981 MHz
NUC1                  13C
P1                  10.00 usec
PLW1          80.00000000 W

======== CHANNEL f2 ========
SFO2          600.1324005 MHz
NUC2                   1H
CPDPRG[2          waltz16
PCPD2               70.00 usec
PLW2          13.43999958 W
PLW12          0.61714000 W
PLW13          0.31042001 W

F2 - Processing parameters
SI                  32768
SF            150.9028156 MHz
WDW                    EM
SSB                     0
LB                   1.00 Hz
GB                      0
PC                   1.40
```

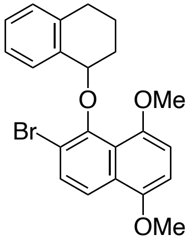

13g

^{1}H NMR (600 MHz, $CDCl_3$)

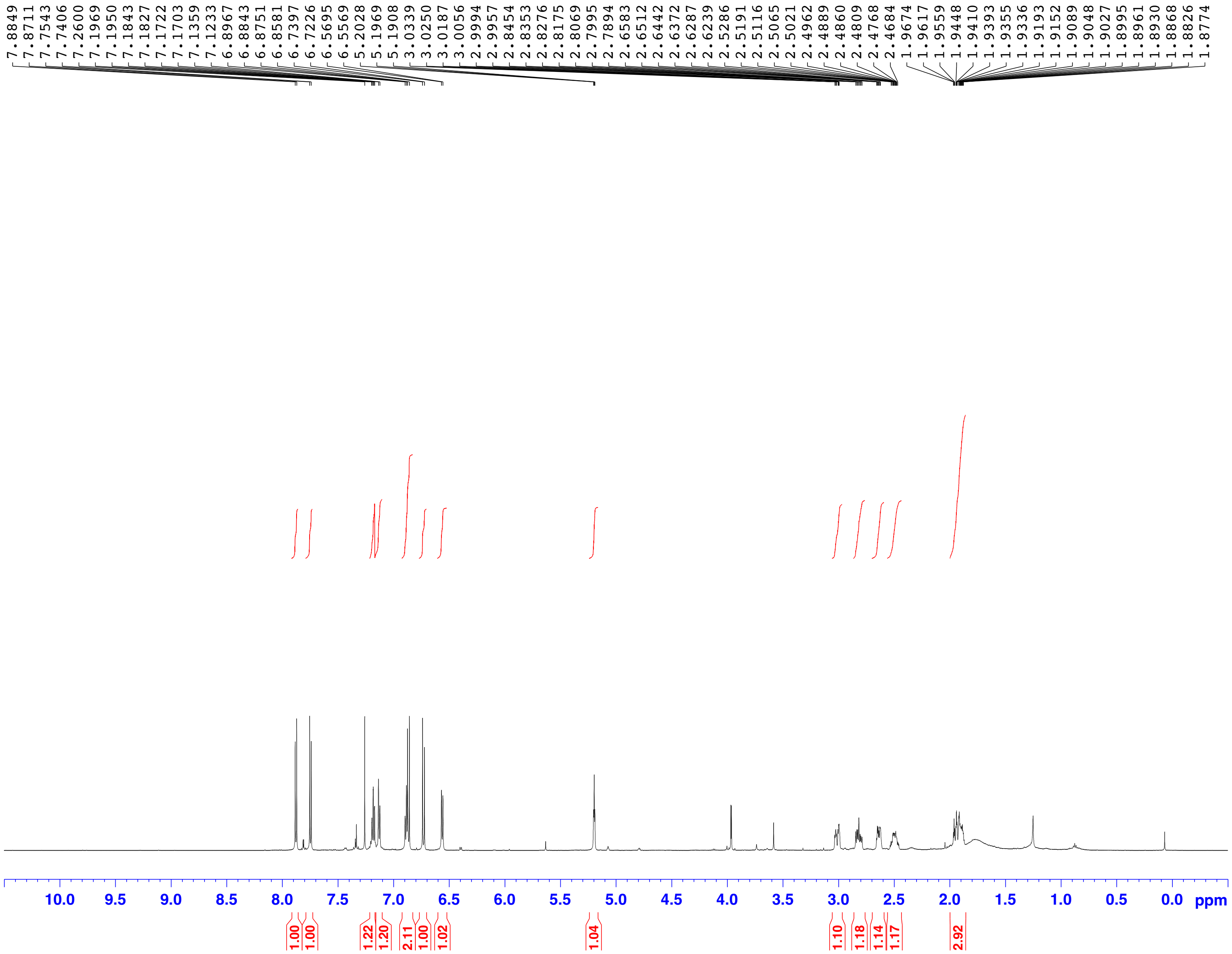

BRUKER

```
Current Data Parameters
NAME        YA1-2077-crude
EXPNO                   10
PROCNO                   1

F2 - Acquisition Parameters
Date_             20220622
Time                 17.15
INSTRUM              spect
PROBHD    5 mm CPPBBO BB
PULPROG               zg30
TD                   65536
SOLVENT              CDCl3
NS                      16
DS                       2
SWH              12019.230 Hz
FIDRES            0.183399 Hz
AQ               2.7262976 sec
RG                   31.94
DW                  41.600 usec
DE                   10.00 usec
TE                   298.1 K
D1              1.00000000 sec
TD0                      1

======== CHANNEL f1 ========
SFO1           600.1337060 MHz
NUC1                    1H
P1                   12.00 usec
PLW1           21.00000000 W

F2 - Processing parameters
SI                   65536
SF             600.1300148 MHz
WDW                     EM
SSB                      0
LB                    0.30 Hz
GB                       0
PC                    1.00
```

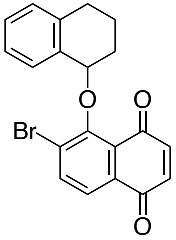

9g
(crude)

^{13}C NMR (150 MHz, $CDCl_3$)

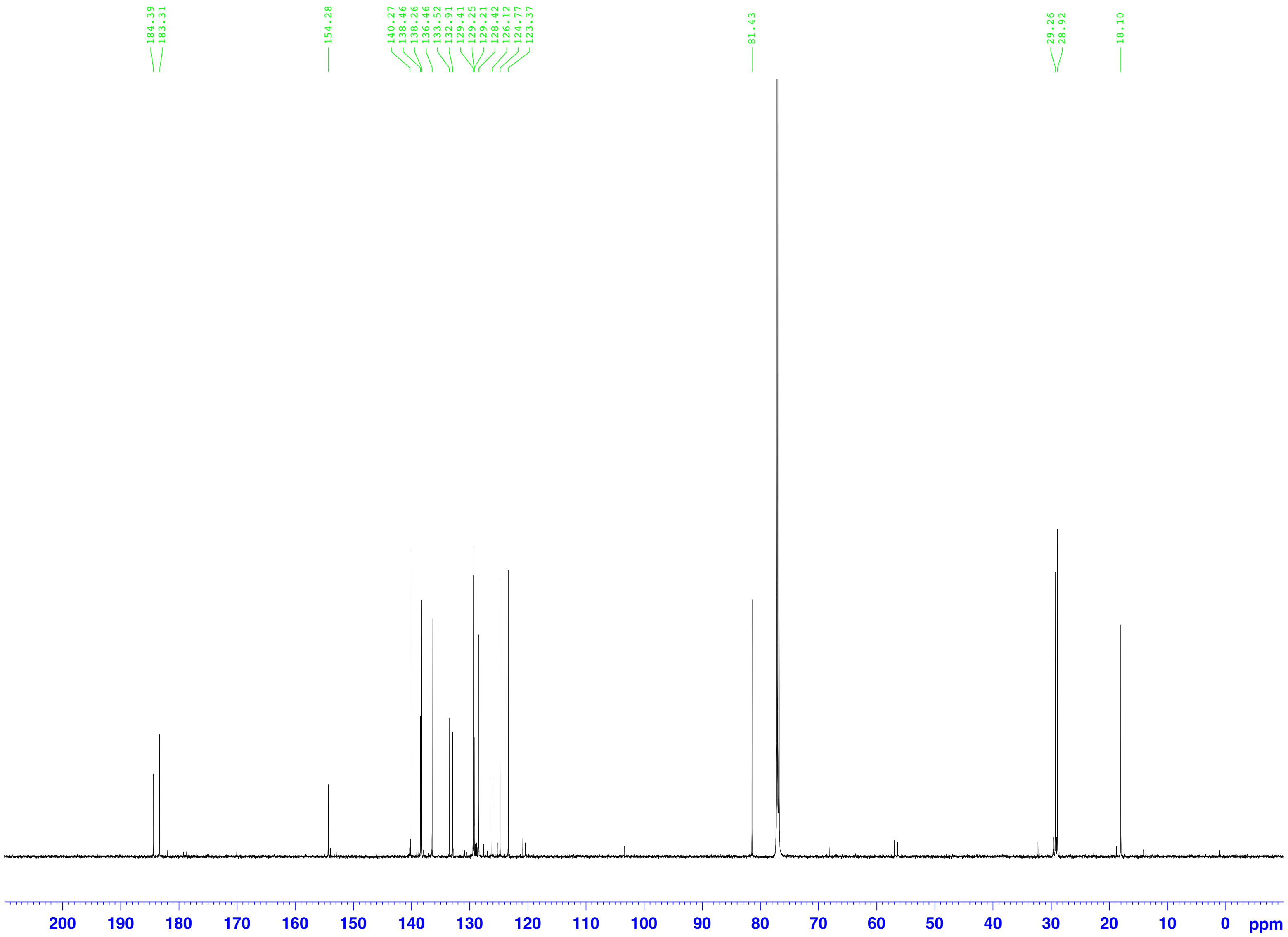


```
Current Data Parameters
NAME       YA1-2077-crude
EXPNO                  12
PROCNO                  1

F2 - Acquisition Parameters
Date_            20220623
Time                 0.01
INSTRUM             spect
PROBHD     5 mm CPPBBO BB
PULPROG           zgpg30
TD                  65536
SOLVENT             CDCl3
NS                   2400
DS                      4
SWH             36057.691 Hz
FIDRES           0.550197 Hz
AQ              0.9087659 sec
RG                 175.56
DW                 13.867 usec
DE                  18.00 usec
TE                  298.1 K
D1             2.00000000 sec
D11            0.03000000 sec
TD0                     1

======== CHANNEL f1 ========
SFO1          150.9178981 MHz
NUC1                  13C
P1                  10.00 usec
PLW1          80.00000000 W

======== CHANNEL f2 ========
SFO2          600.1324005 MHz
NUC2                   1H
CPDPRG[2          waltz16
PCPD2               70.00 usec
PLW2          13.43999958 W
PLW12          0.61714000 W
PLW13          0.31042001 W

F2 - Processing parameters
SI                  32768
SF            150.9028133 MHz
WDW                    EM
SSB                     0
LB                   1.00 Hz
GB                      0
PC                   1.40
```

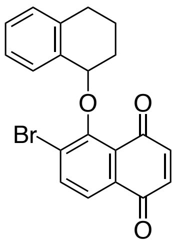

9g
(crude)

^{1}H NMR (600 MHz, $CDCl_3$)

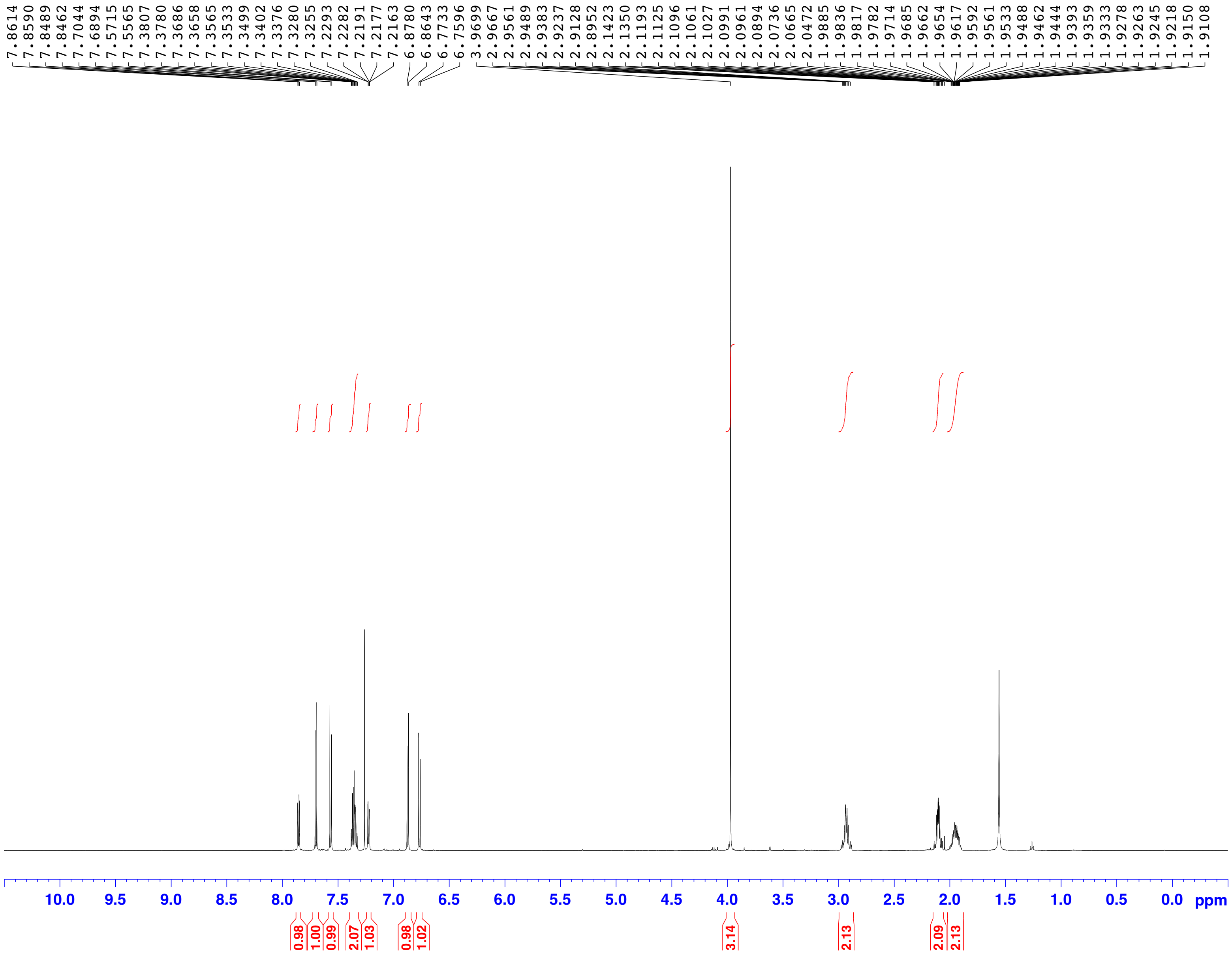

BRUKER

Current Data Parameters
NAME YA1-2072-t-1
EXPNO 20
PROCNO 1

F2 - Acquisition Parameters
Date_ 20220622
Time 17.12
INSTRUM spect
PROBHD 5 mm CPPBBO BB
PULPROG zg30
TD 65536
SOLVENT CDCl3
NS 16
DS 2
SWH 12019.230 Hz
FIDRES 0.183399 Hz
AQ 2.7262976 sec
RG 31.94
DW 41.600 usec
DE 10.00 usec
TE 298.3 K
D1 1.00000000 sec
TD0 1

======== CHANNEL f1 ========
SFO1 600.1337060 MHz
NUC1 1H
P1 12.00 usec
PLW1 21.00000000 W

F2 - Processing parameters
SI 65536
SF 600.1300143 MHz
WDW EM
SSB 0
LB 0.30 Hz
GB 0
PC 1.00

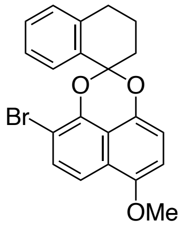

11g

^{13}C NMR (150 MHz, $CDCl_3$)

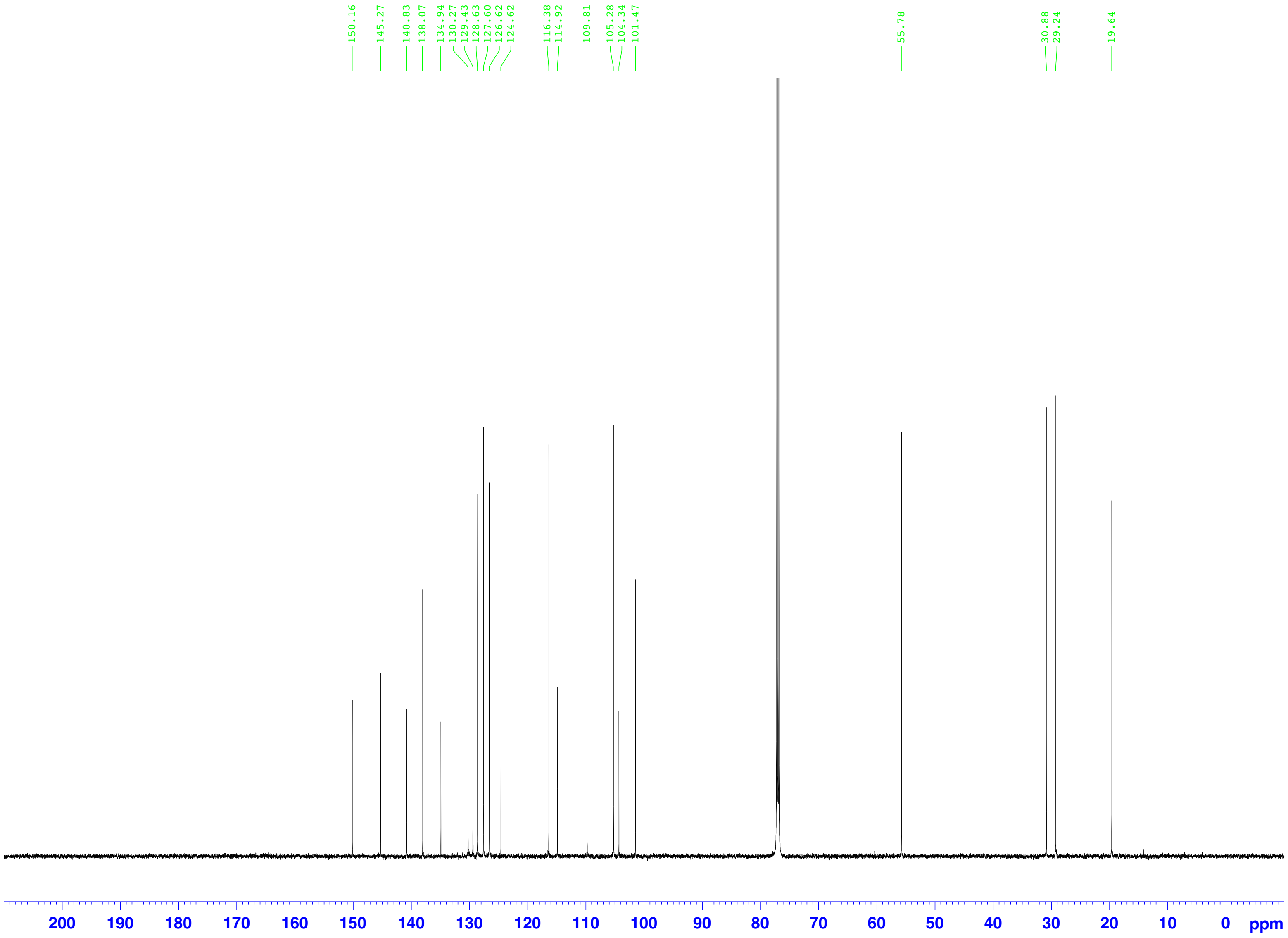


```
Current Data Parameters
NAME         YA1-2072-t-1
EXPNO                  22
PROCNO                  1

F2 - Acquisition Parameters
Date_            20220623
Time                 4.40
INSTRUM             spect
PROBHD    5 mm CPPBBO BB
PULPROG           zgpg30
TD                  65536
SOLVENT             CDCl3
NS                   3600
DS                      4
SWH             36057.691 Hz
FIDRES           0.550197 Hz
AQ              0.9087659 sec
RG                 175.56
DW                 13.867 usec
DE                  18.00 usec
TE                  298.1 K
D1             2.00000000 sec
D11            0.03000000 sec
TD0                     1

======== CHANNEL f1 ========
SFO1          150.9178981 MHz
NUC1                  13C
P1                  10.00 usec
PLW1          80.00000000 W

======== CHANNEL f2 ========
SFO2          600.1324005 MHz
NUC2                   1H
CPDPRG[2          waltz16
PCPD2               70.00 usec
PLW2          13.43999958 W
PLW12          0.61714000 W
PLW13          0.31042001 W

F2 - Processing parameters
SI                  32768
SF            150.9028132 MHz
WDW                    EM
SSB                     0
LB                   1.00 Hz
GB                      0
PC                   1.40
```

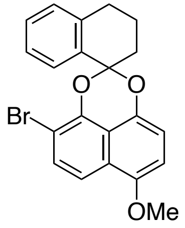

11g

^{1}H NMR (600 MHz, $CDCl_3$)

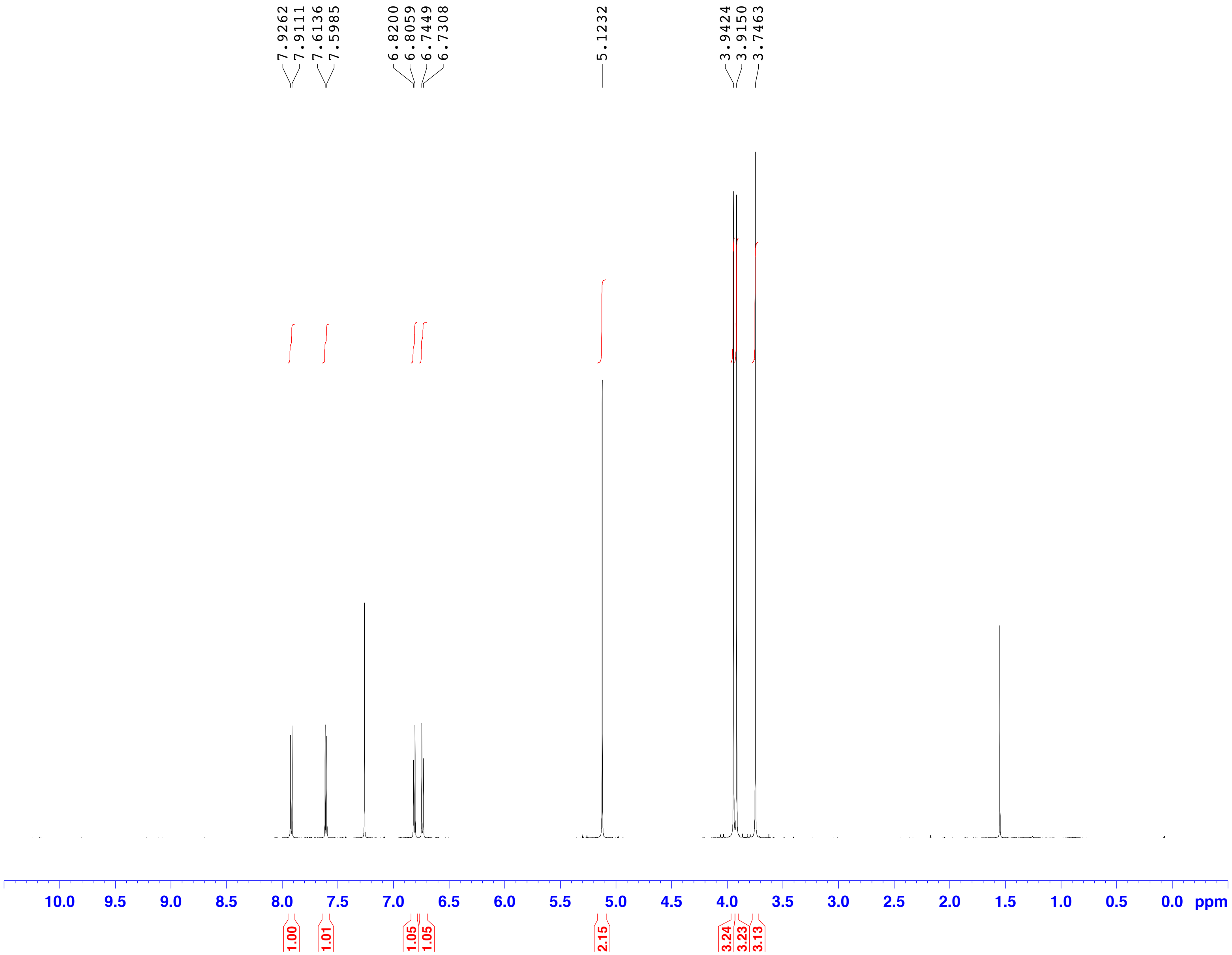

BRUKER

```
Current Data Parameters
NAME              YA1-1740-1
EXPNO                     10
PROCNO                     1

F2 - Acquisition Parameters
Date_               20200116
Time                   19.26
INSTRUM                spect
PROBHD    5 mm CPPBBO BB
PULPROG                 zg30
TD                     65536
SOLVENT                CDCl3
NS                        16
DS                         2
SWH            12019.230 Hz
FIDRES          0.183399 Hz
AQ             2.7262976 sec
RG                     31.94
DW                41.600 use
DE                 10.00 use
TE                 298.2 K
D1            1.00000000 sec
TD0                        1

======== CHANNEL f1 =======
SFO1         600.1337060 MHz
NUC1                      1H
P1                 12.00 use
PLW1         21.00000000 W

F2 - Processing parameters
SI                     65536
SF           600.1300150 MHz
WDW                       EM
SSB       0
LB                  0.30 Hz
GB        0
PC                     1.00
```

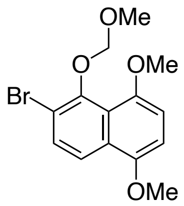

13h

^{13}C NMR (150 MHz, $CDCl_3$)

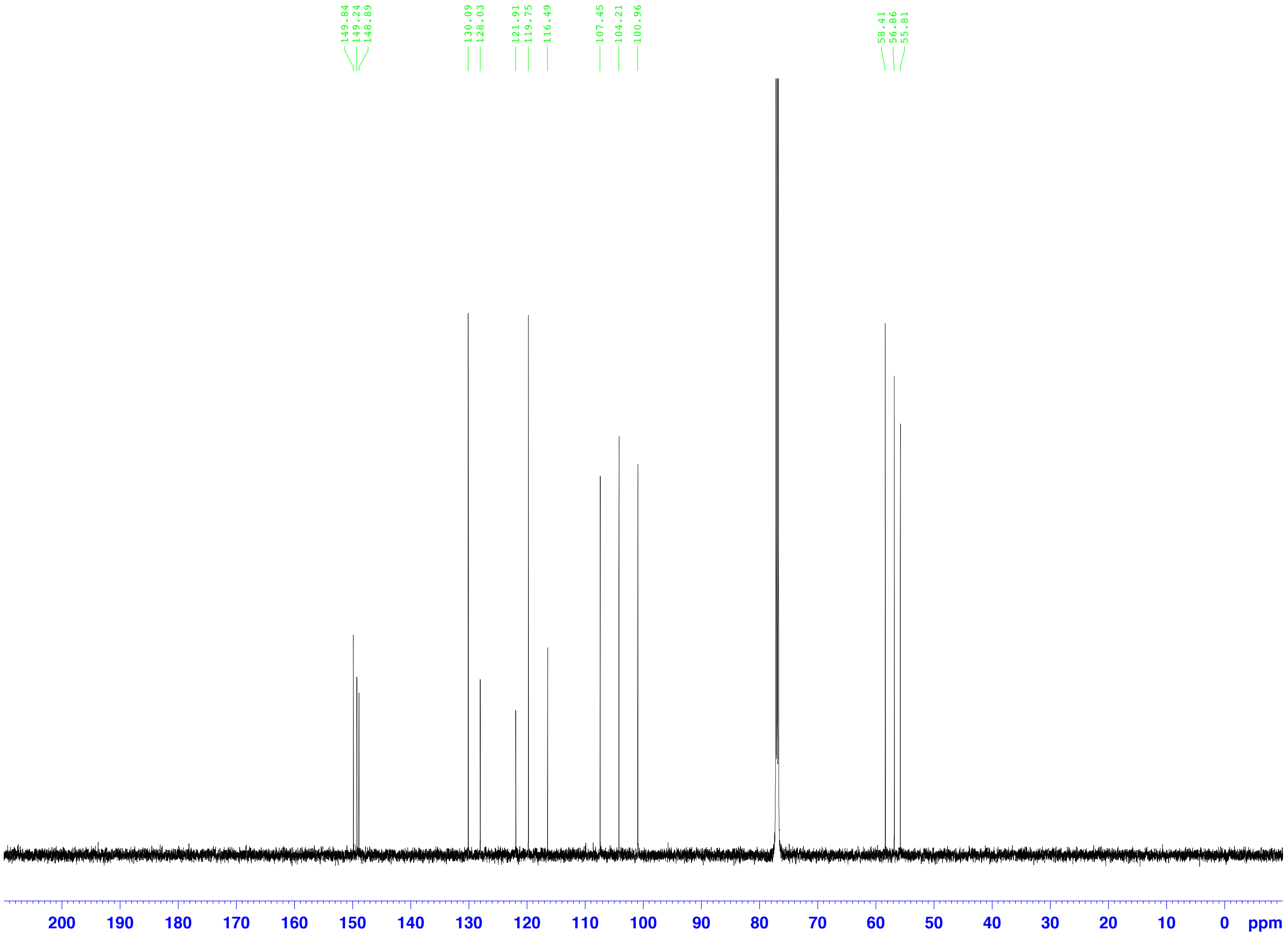


```
Current Data Parameters
NAME            YA1-1740-1
EXPNO                   12
PROCNO                   1

F2 - Acquisition Parameters
Date_             20200118
Time                  3.54
INSTRUM              spect
PROBHD   5 mm CPPBBO BB
PULPROG             zgpg30
TD                   65536
SOLVENT              CDCl3
NS                    1024
DS                       4
SWH              36057.691 Hz
FIDRES            0.550197 Hz
AQ               0.9087659 sec
RG                  175.56
DW                  13.867 use
DE                   18.00 use
TE                   298.2 K
D1              2.00000000 sec
D11             0.03000000 sec
TD0                      1

======== CHANNEL f1 =======
SFO1           150.9178981 MHz
NUC1                   13C
P1                   10.00 use
PLW1           80.00000000 W

======== CHANNEL f2 =======
SFO2           600.1324005 MHz
NUC2                    1H
CPDPRG[2           waltz16
PCPD2                70.00 use
PLW2           13.43999958 W
PLW12           0.61714000 W
PLW13           0.31042001 W

F2 - Processing parameters
SI                   32768
SF             150.9028124 MHz
WDW                     EM
SSB      0
LB                    1.00 Hz
GB       0
PC                    1.40
```

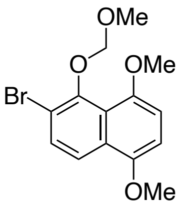

13h

^{1}H NMR (600 MHz, acetone-d_6)

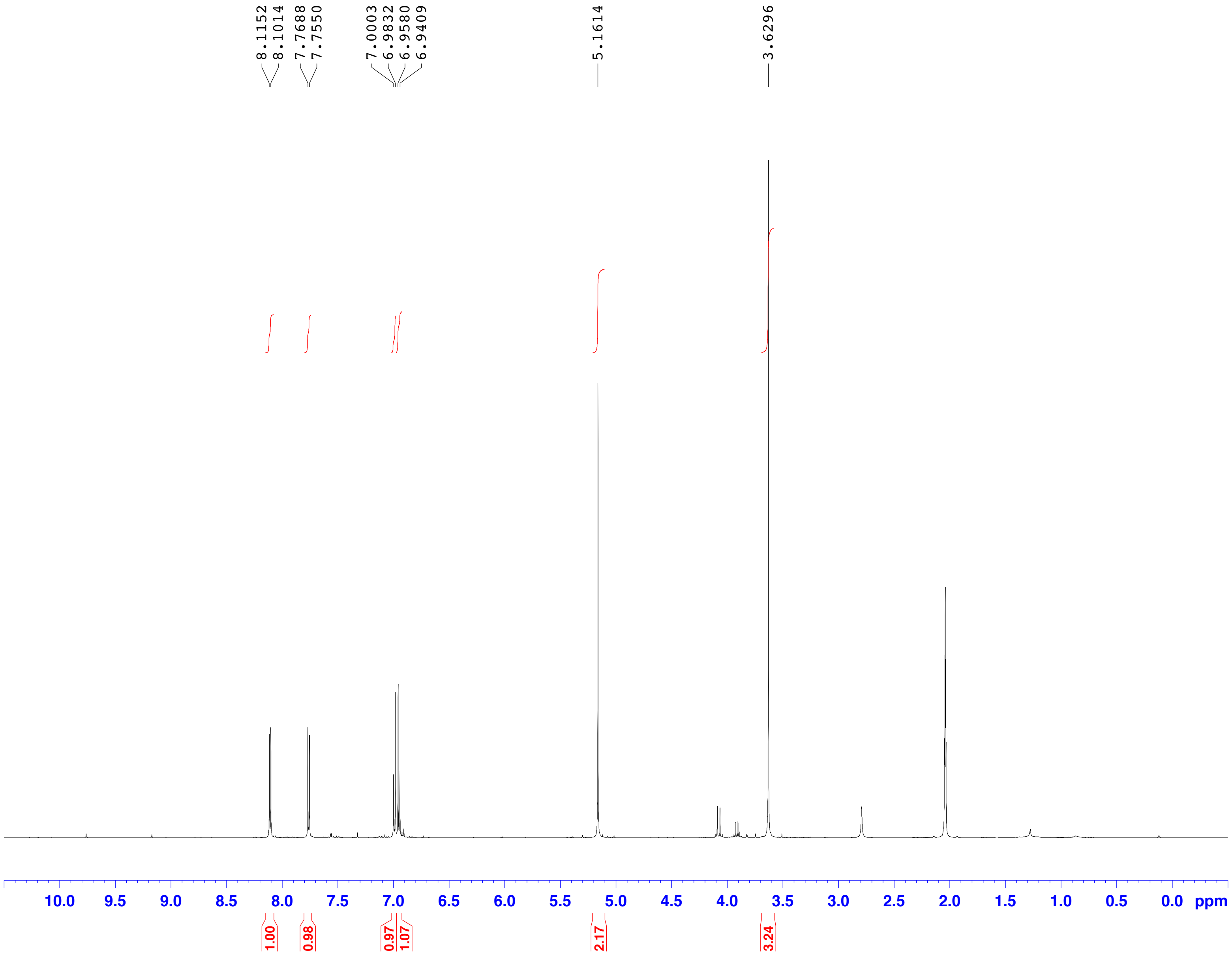

BRUKER

```
Current Data Parameters
NAME       YA1-1875-crude
EXPNO                  10
PROCNO                  1

F2 - Acquisition Parameters
Date_            20201224
Time                17.00
INSTRUM             spect
PROBHD    5 mm CPPBBO BB
PULPROG              zg30
TD                  65536
SOLVENT           Acetone
NS                     16
DS                      2
SWH             12019.230 Hz
FIDRES           0.183399 Hz
AQ              2.7262976 sec
RG                  31.94
DW                 41.600 use
DE                  10.00 use
TE                  298.2 K
D1             1.00000000 sec
TD0                     1

======== CHANNEL f1 =======
SFO1          600.1337060 MHz
NUC1                   1H
P1                  12.00 use
PLW1          21.00000000 W

F2 - Processing parameters
SI                  65536
SF            600.1300163 MHz
WDW                    EM
SSB       0
LB                   0.30 Hz
GB        0
PC                   1.00
```

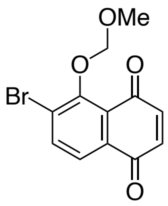

9h
(crude)

^{13}C NMR (150 MHz, acetone-d_6)

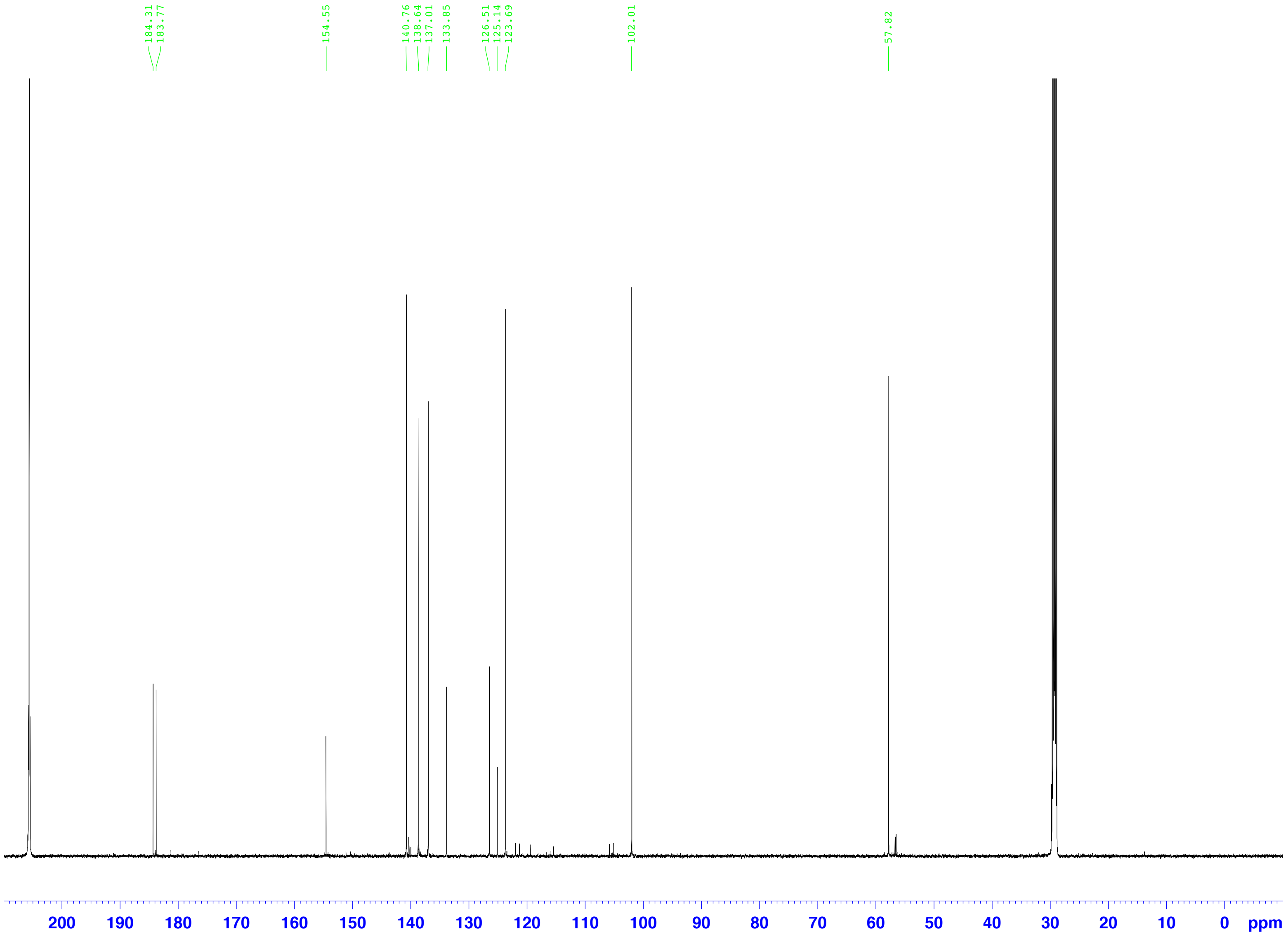


```
Current Data Parameters
NAME        YA1-1875-crude
EXPNO                   11
PROCNO                   1

F2 - Acquisition Parameters
Date_             20201225
Time                  5.02
INSTRUM              spect
PROBHD    5 mm CPPBBO BB
PULPROG            zgpg30
TD                  65536
SOLVENT            Acetone
NS                   2400
DS                      4
SWH            36057.691 Hz
FIDRES          0.550197 Hz
AQ             0.9087659 sec
RG                 175.56
DW                 13.867 use
DE                  18.00 use
TE                  298.0 K
D1             2.00000000 sec
D11            0.03000000 sec
TD0                     1

======== CHANNEL f1 =======
SFO1          150.9178981 MHz
NUC1                  13C
P1                  10.00 use
PLW1          80.00000000 W

======== CHANNEL f2 =======
SFO2          600.1324005 MHz
NUC2                   1H
CPDPRG[2          waltz16
PCPD2               70.00 use
PLW2          13.43999958 W
PLW12          0.61714000 W
PLW13          0.31042001 W

F2 - Processing parameters
SI                  32768
SF            150.9027567 MHz
WDW                    EM
SSB      0
LB                   1.00 Hz
GB       0
PC                   1.40
```

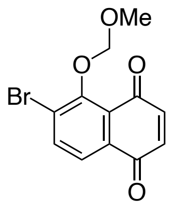

9h
(crude)

^{1}H NMR (600 MHz, acetone-d_6)

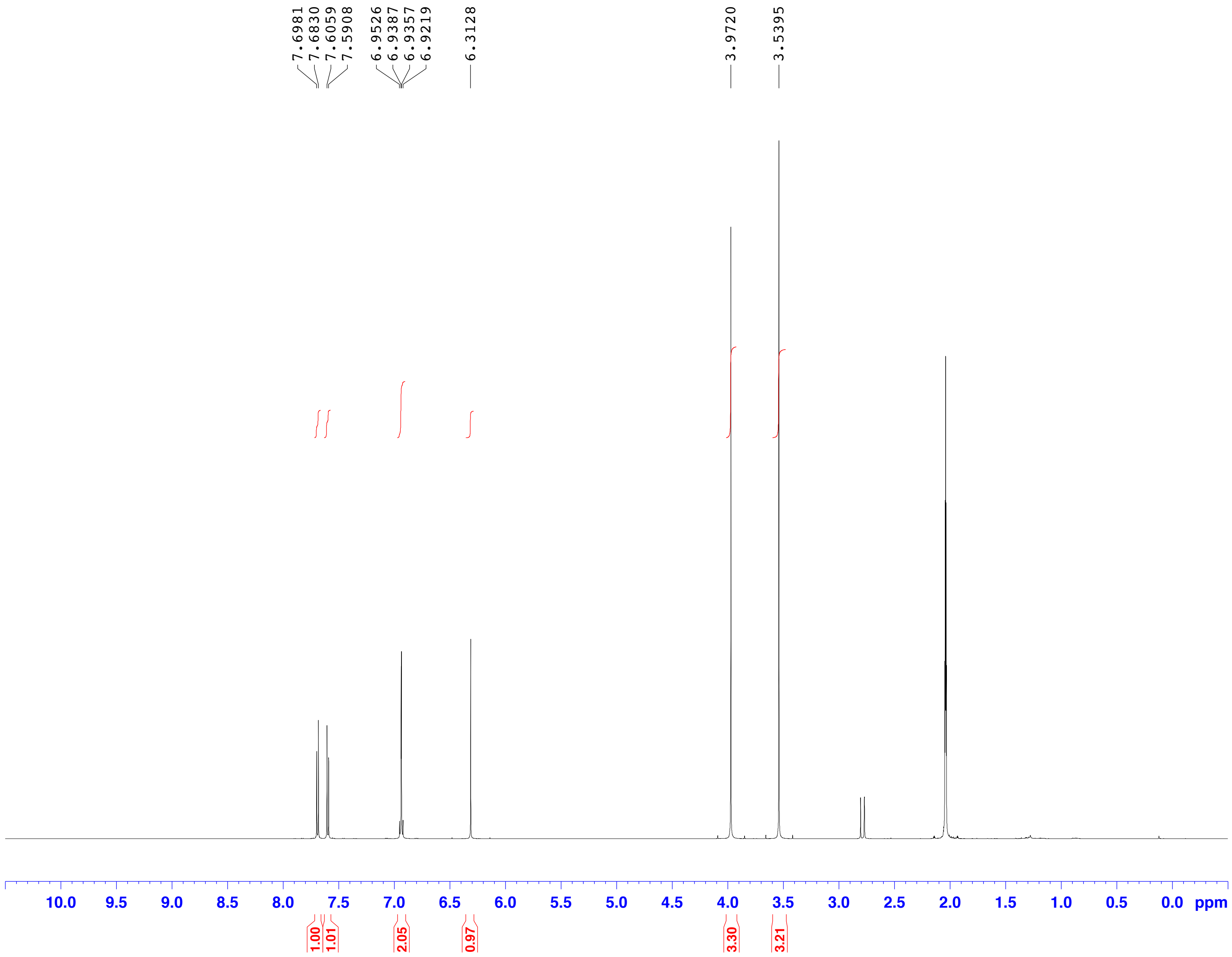


```
Current Data Parameters
NAME              YA1-1870-1
EXPNO                     10
PROCNO                     1

F2 - Acquisition Parameters
Date_               20201223
Time                   14.47
INSTRUM                spect
PROBHD   5 mm CPPBBO BB
PULPROG                 zg30
TD                     65536
SOLVENT              Acetone
NS                        16
DS                         2
SWH               12019.230 Hz
FIDRES             0.183399 Hz
AQ                2.7262976 sec
RG                     31.94
DW                    41.600 use
DE                     10.00 use
TE                     298.1 K
D1               1.00000000 sec
TD0                        1

======== CHANNEL f1 =======
SFO1            600.1337060 MHz
NUC1                      1H
P1                     12.00 use
PLW1            21.00000000 W

F2 - Processing parameters
SI                     65536
SF              600.1300163 MHz
WDW                       EM
SSB       0
LB                      0.30 Hz
GB        0
PC                      1.00
```

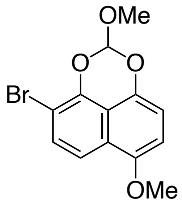

11h

^{13}C NMR (150 MHz, acetone-d_6)

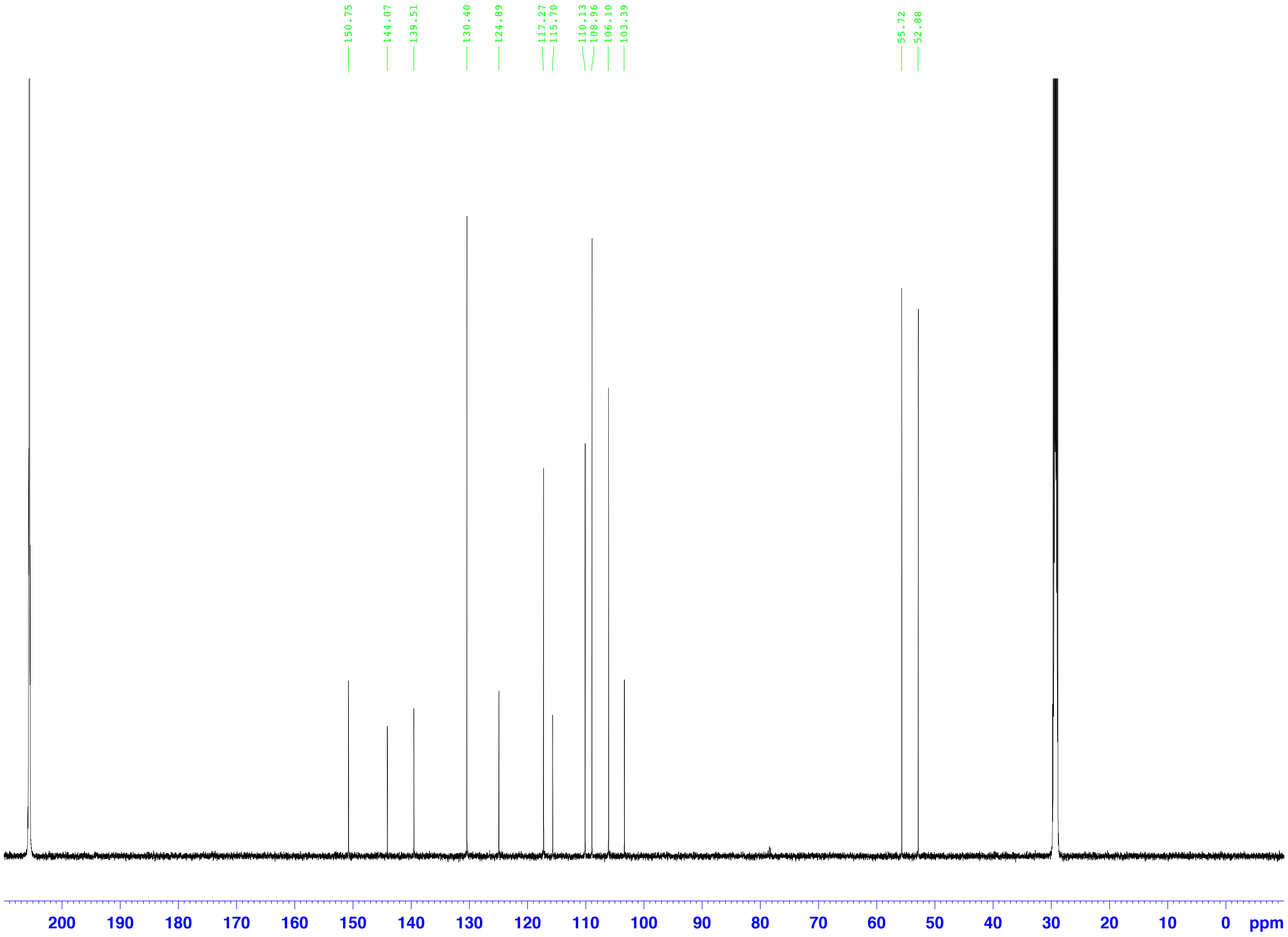


```
Current Data Parameters
NAME            YA1-1870-1
EXPNO                   11
PROCNO                   1

F2 - Acquisition Parameters
Date_             20201224
Time                  0.03
INSTRUM              spect
PROBHD   5 mm CPPBBO BB
PULPROG            zgpg30
TD                  65536
SOLVENT            Acetone
NS                   2400
DS                      4
SWH             36057.691 Hz
FIDRES           0.550197 Hz
AQ              0.9087659 sec
RG                 175.56
DW                 13.867 use
DE                  18.00 use
TE                  298.2 K
D1             2.00000000 sec
D11            0.03000000 sec
TD0                     1

======== CHANNEL f1 =======
SFO1         150.9178981 MHz
NUC1                 13C
P1                 10.00 use
PLW1         80.00000000 W

======== CHANNEL f2 =======
SFO2         600.1324005 MHz
NUC2                  1H
CPDPRG[2         waltz16
PCPD2              70.00 use
PLW2         13.43999958 W
PLW12         0.61714000 W
PLW13         0.31042001 W

F2 - Processing parameters
SI                 32768
SF           150.9027552 MHz
WDW                   EM
SSB      0
LB                  1.00 Hz
GB       0
PC                  1.40
```

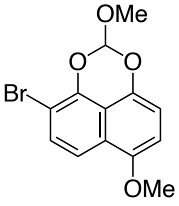

11h

^{1}H NMR (600 MHz, $CDCl_3$)

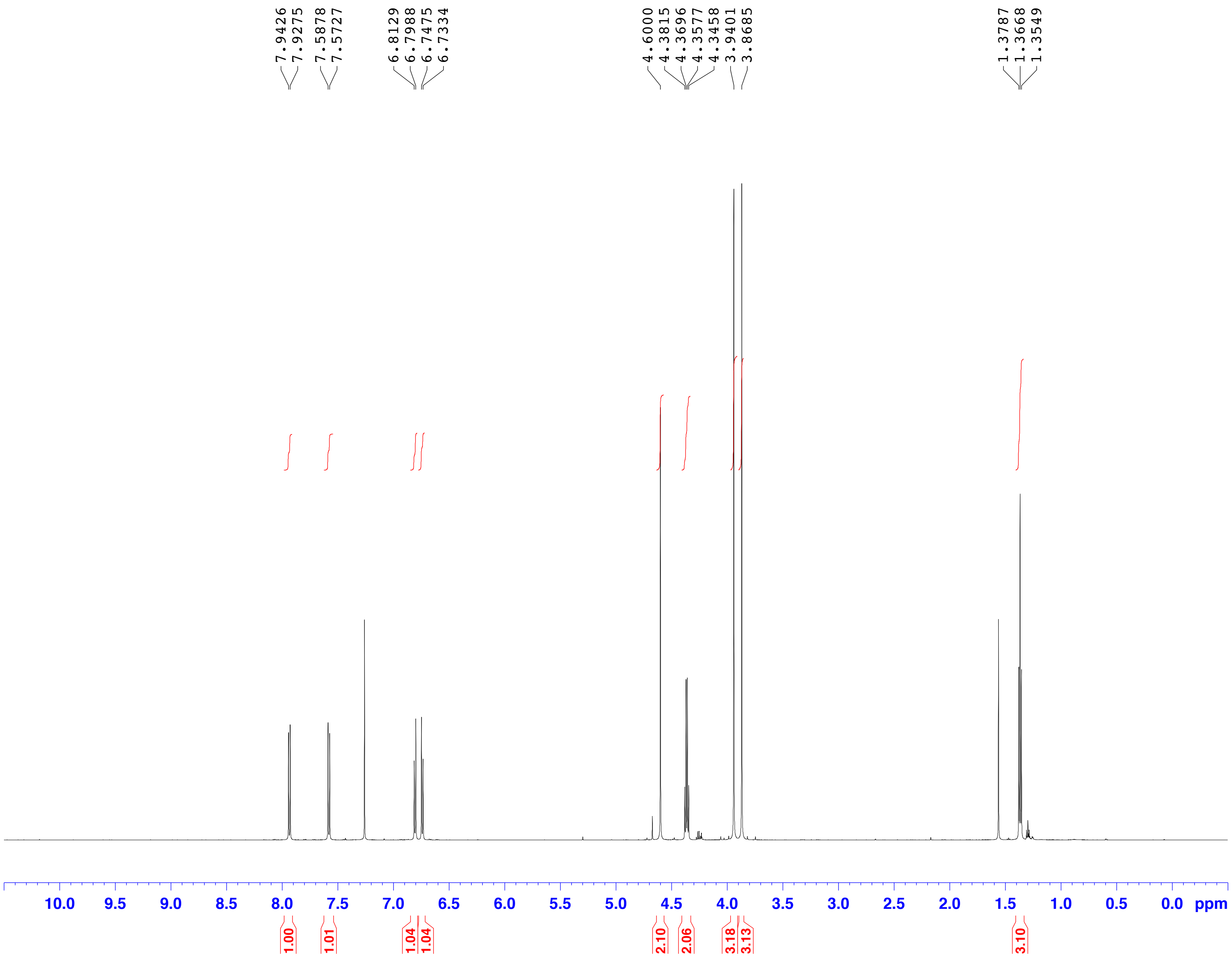


```
Current Data Parameters
NAME             YA1-1744-1
EXPNO                    10
PROCNO                    1

F2 - Acquisition Parameters
Date_              20200117
Time                  13.53
INSTRUM               spect
PROBHD    5 mm CPPBBO BB
PULPROG                zg30
TD                    65536
SOLVENT               CDCl3
NS                       16
DS                        2
SWH              12019.230 Hz
FIDRES            0.183399 Hz
AQ               2.7262976 sec
RG                     17.5
DW                   41.600 use
DE                    10.00 use
TE                    298.2 K
D1              1.00000000 sec
TD0                       1

======== CHANNEL f1 =======
SFO1           600.1337060 MHz
NUC1                     1H
P1                    12.00 use
PLW1            21.00000000 W

F2 - Processing parameters
SI                    65536
SF             600.1300148 MHz
WDW                      EM
SSB      0
LB                     0.30 Hz
GB       0
PC                     1.00
```

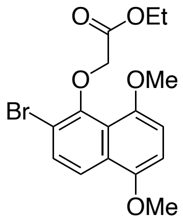

13i

^{13}C NMR (150 MHz, $CDCl_3$)

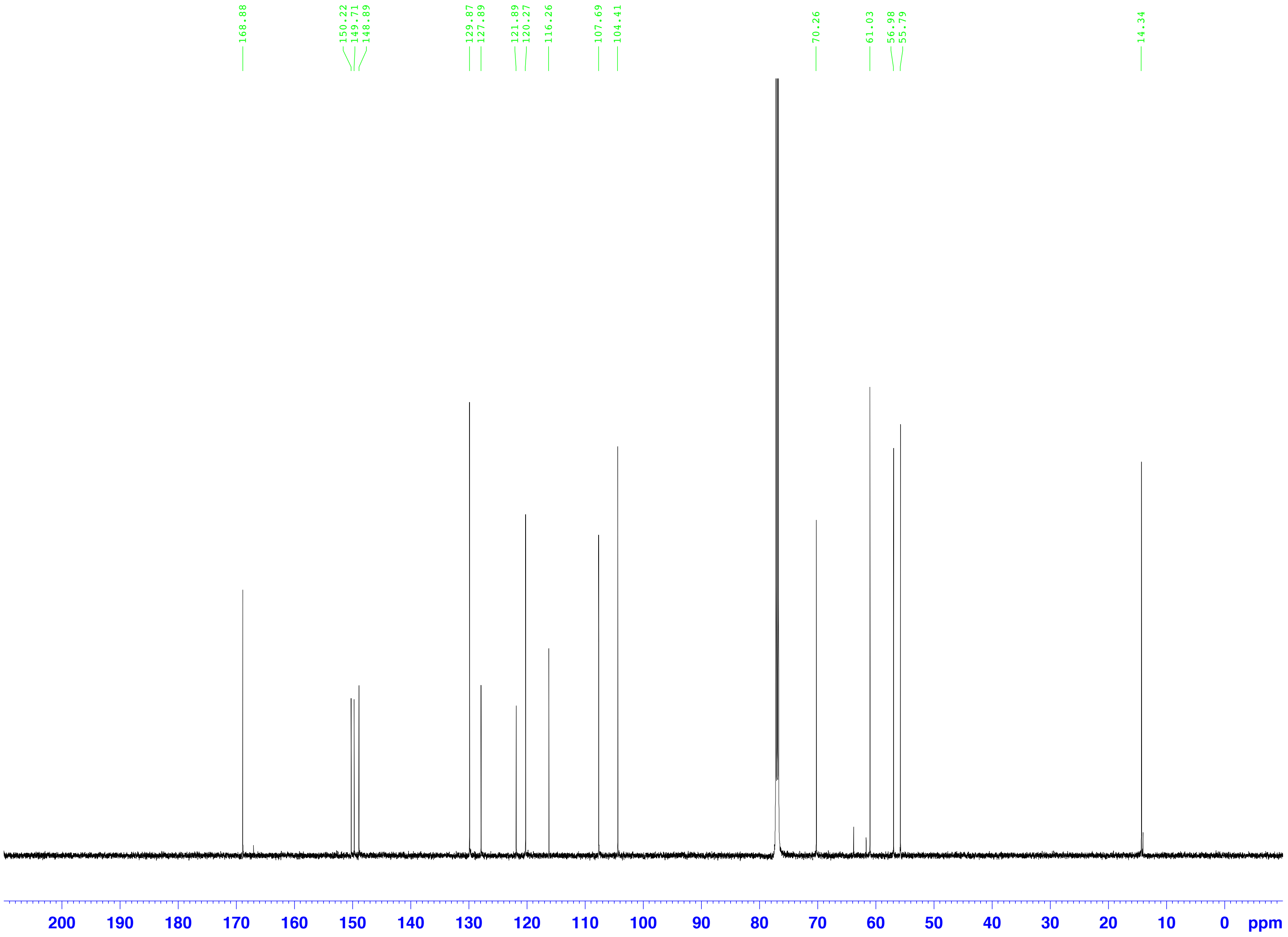


```
Current Data Parameters
NAME            YA1-1744-1
EXPNO                   11
PROCNO                   1

F2 - Acquisition Parameters
Date_             20200118
Time                 19.47
INSTRUM              spect
PROBHD    5 mm CPPBBO BB
PULPROG            zgpg30
TD                  65536
SOLVENT             CDCl3
NS                   3000
DS                      4
SWH             36057.691 Hz
FIDRES           0.550197 Hz
AQ              0.9087659 sec
RG                 175.56
DW                 13.867 use
DE                  18.00 use
TE                  298.2 K
D1             2.00000000 sec
D11            0.03000000 sec
TD0                     1

======== CHANNEL f1 =======
SFO1          150.9178981 MHz
NUC1                  13C
P1                   10.00 use
PLW1          80.00000000 W

======== CHANNEL f2 =======
SFO2          600.1324005 MHz
NUC2                   1H
CPDPRG[2          waltz16
PCPD2                70.00 use
PLW2          13.43999958 W
PLW12          0.61714000 W
PLW13          0.31042001 W

F2 - Processing parameters
SI                  32768
SF            150.9028124 MHz
WDW                    EM
SSB      0
LB                   1.00 Hz
GB       0
PC                   1.40
```

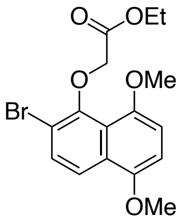

13i

^{1}H NMR (600 MHz, $CDCl_3$)

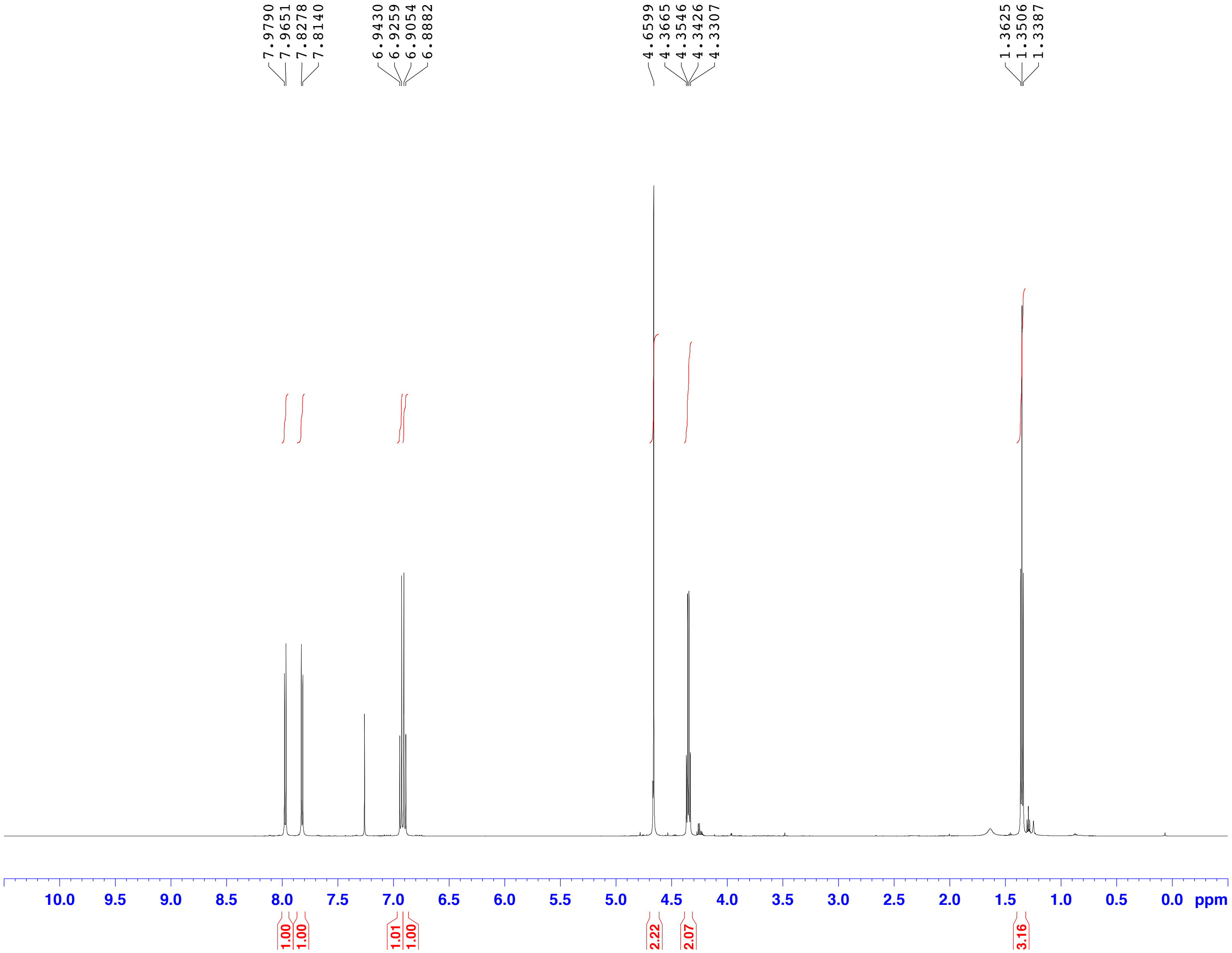


```
Current Data Parameters
NAME      YA1-1863-crude
EXPNO                 10
PROCNO                 1

F2 - Acquisition Parameters
Date_           20201130
Time               19.55
INSTRUM            spect
PROBHD   5 mm CPPBBO BB
PULPROG             zg30
TD                 65536
SOLVENT            CDCl3
NS                    16
DS                     2
SWH            12019.230 Hz
FIDRES          0.183399 Hz
AQ             2.7262976 sec
RG                 31.94
DW                41.600 use
DE                 10.00 use
TE                 298.0 K
D1            1.00000000 sec
TD0                    1

======== CHANNEL f1 =======
SFO1         600.1337060 MHz
NUC1                  1H
P1                 12.00 use
PLW1         21.00000000 W

F2 - Processing parameters
SI                 65536
SF           600.1300148 MHz
WDW                   EM
SSB       0
LB                  0.30 Hz
GB        0
PC                  1.00
```

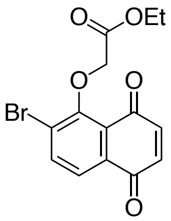

9i
(crude)

^{13}C NMR (150 MHz, $CDCl_3$)

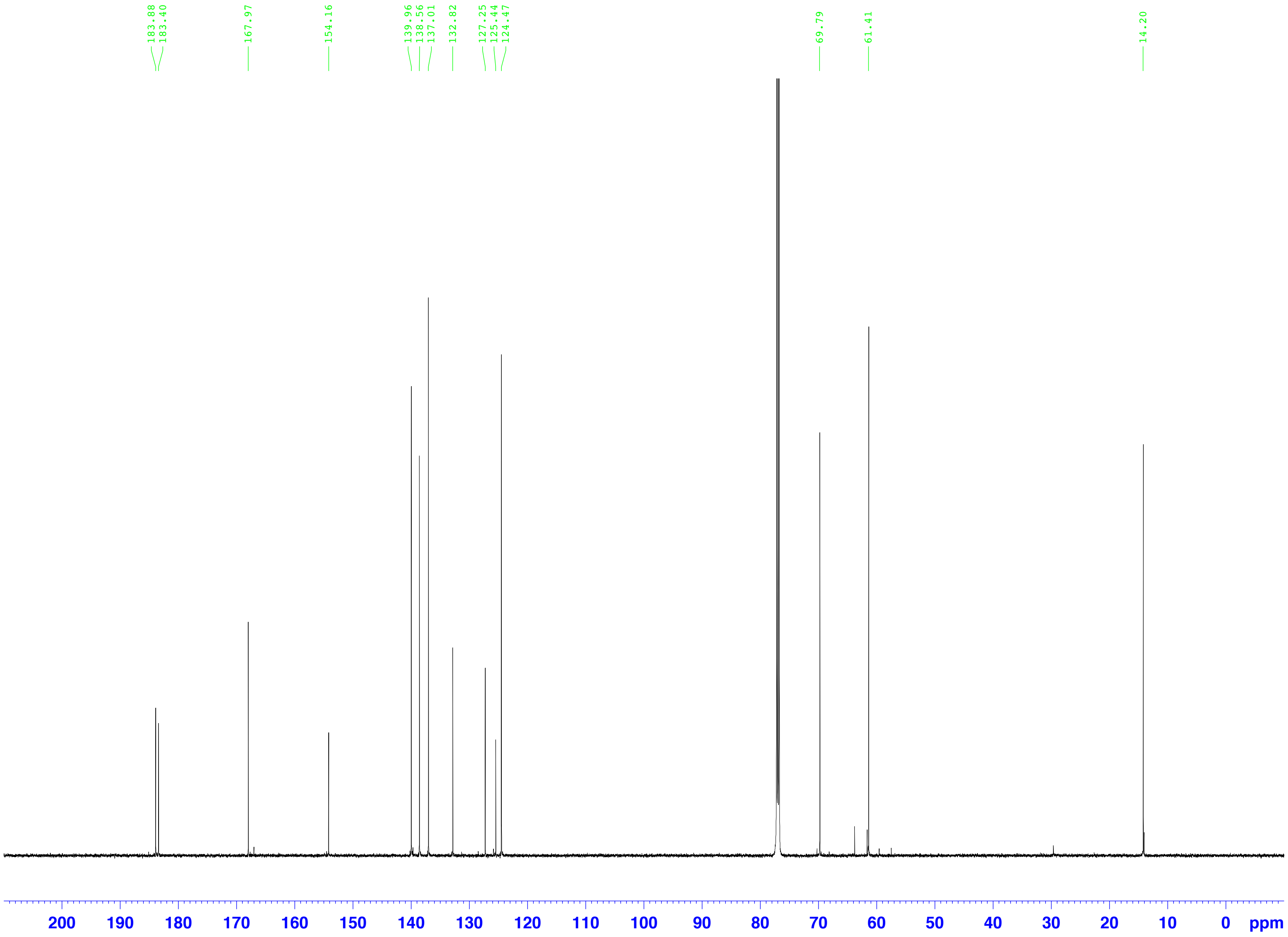


```
Current Data Parameters
NAME      YA1-1863-crude
EXPNO                 11
PROCNO                 1

F2 - Acquisition Parameters
Date_           20201201
Time                0.02
INSTRUM            spect
PROBHD    5 mm CPPBBO BB
PULPROG          zgpg30
TD                 65536
SOLVENT            CDCl3
NS                  2400
DS                     4
SWH            36057.691 Hz
FIDRES          0.550197 Hz
AQ             0.9087659 sec
RG                175.56
DW                13.867 use
DE                 18.00 use
TE                 298.2 K
D1            2.00000000 sec
D11           0.03000000 sec
TD0                    1

======== CHANNEL f1 =======
SFO1         150.9178981 MHz
NUC1                 13C
P1                 10.00 use
PLW1         80.00000000 W

======== CHANNEL f2 =======
SFO2         600.1324005 MHz
NUC2                  1H
CPDPRG[2         waltz16
PCPD2              70.00 use
PLW2         13.43999958 W
PLW12         0.61714000 W
PLW13         0.31042001 W

F2 - Processing parameters
SI                 32768
SF           150.9028146 MHz
WDW                   EM
SSB       0
LB                  1.00 Hz
GB        0
PC                  1.40
```

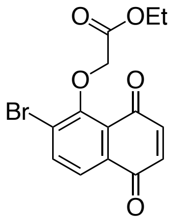

9i
(crude)

^{1}H NMR (600 MHz, $CDCl_3$)

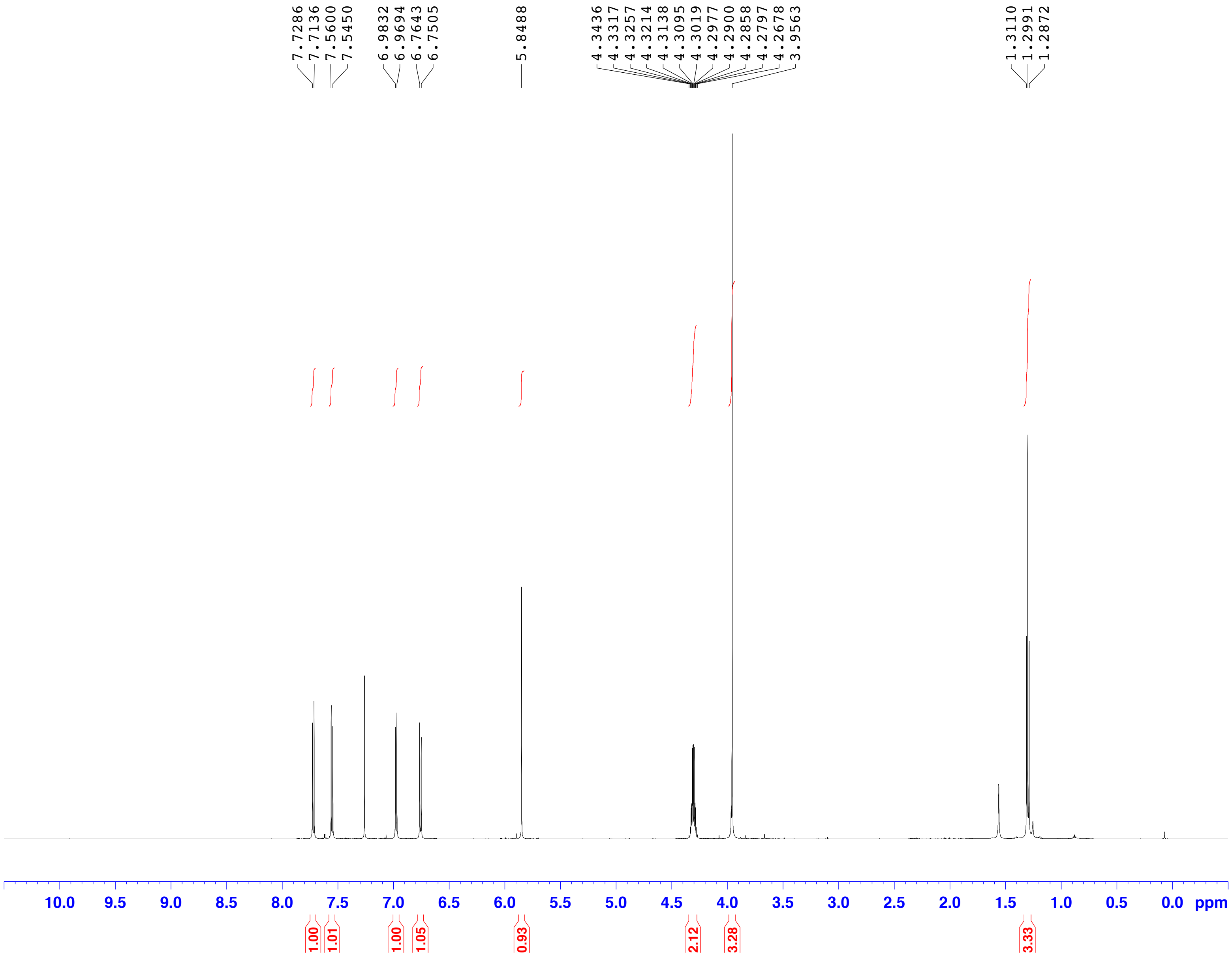


```
Current Data Parameters
NAME              YA1-1864-1
EXPNO                     11
PROCNO                     1

F2 - Acquisition Parameters
Date_               20201202
Time                   15.16
INSTRUM                spect
PROBHD    5 mm CPPBBO BB
PULPROG                 zg30
TD                     65536
SOLVENT                CDCl3
NS                        16
DS                         2
SWH             12019.230 Hz
FIDRES           0.183399 Hz
AQ              2.7262976 sec
RG                     31.94
DW                41.600 use
DE                 10.00 use
TE                 298.2 K
D1            1.00000000 sec
TD0                        1

======== CHANNEL f1 =======
SFO1          600.1337060 MHz
NUC1                      1H
P1                 12.00 use
PLW1         21.00000000 W

F2 - Processing parameters
SI                     65536
SF            600.1300147 MHz
WDW                       EM
SSB       0
LB                  0.30 Hz
GB        0
PC                      1.00
```

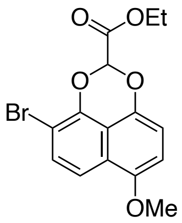

11i

^{13}C NMR (150 MHz, $CDCl_3$)

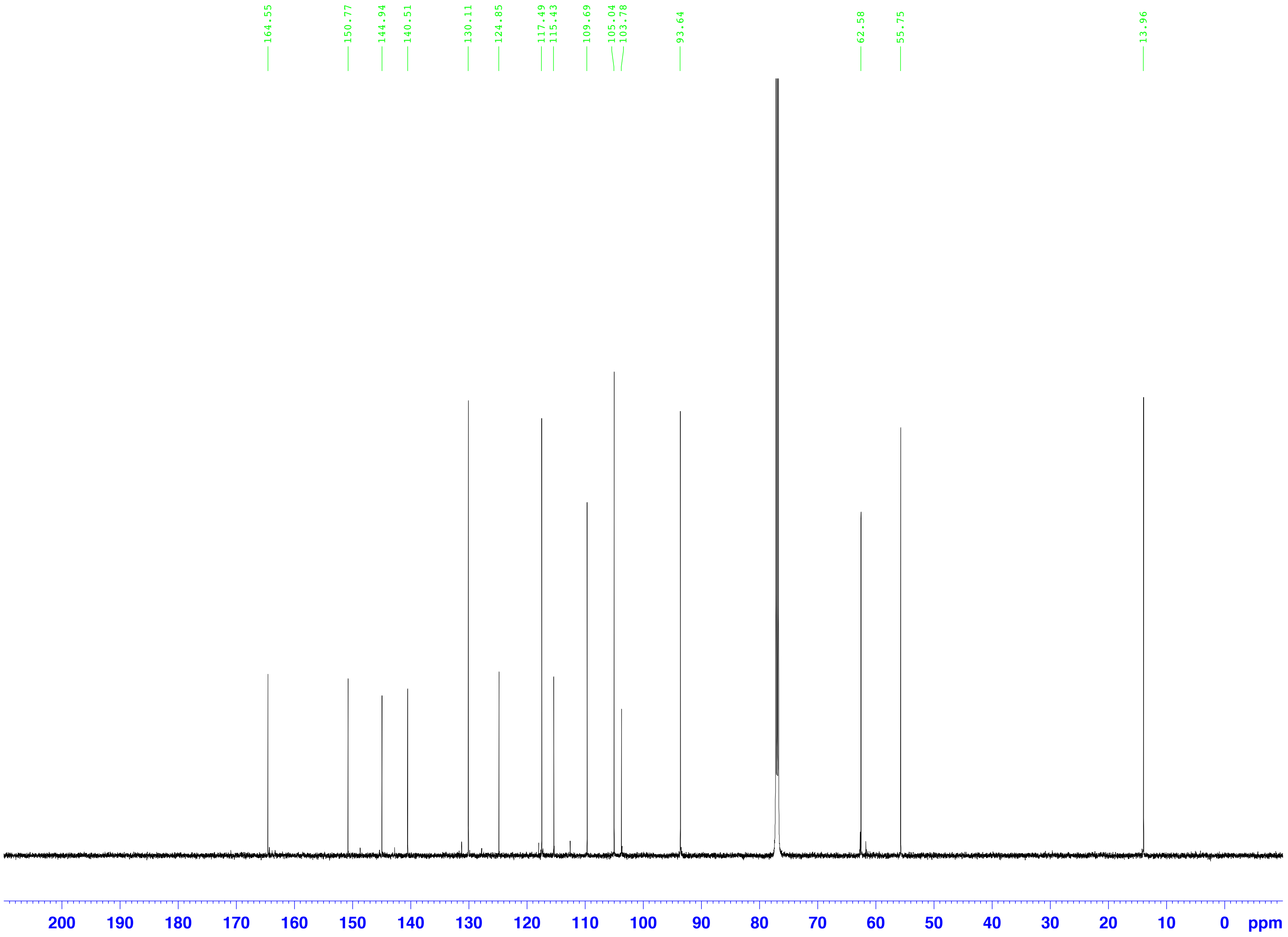


```
Current Data Parameters
NAME              YA1-1760-1
EXPNO                     11
PROCNO                     1

F2 - Acquisition Parameters
Date_               20200201
Time                    2.31
INSTRUM                spect
PROBHD    5 mm CPPBBO BB
PULPROG              zgpg30
TD                     65536
SOLVENT                CDCl3
NS                      3000
DS                         4
SWH               36057.691 Hz
FIDRES             0.550197 Hz
AQ                0.9087659 sec
RG                    175.56
DW                    13.867 use
DE                     18.00 use
TE                     298.2 K
D1                2.00000000 sec
D11               0.03000000 sec
TD0                        1

======== CHANNEL f1 =======
SFO1            150.9178981 MHz
NUC1                     13C
P1                     10.00 use
PLW1             80.00000000 W

======== CHANNEL f2 =======
SFO2            600.1324005 MHz
NUC2                      1H
CPDPRG[2             waltz16
PCPD2                  70.00 use
PLW2             13.43999958 W
PLW12             0.61714000 W
PLW13             0.31042001 W

F2 - Processing parameters
SI                     32768
SF              150.9028126 MHz
WDW                       EM
SSB       0
LB                      1.00 Hz
GB        0
PC                      1.40
```

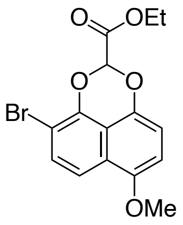

11i

^{1}H NMR (600 MHz, $CDCl_3$)

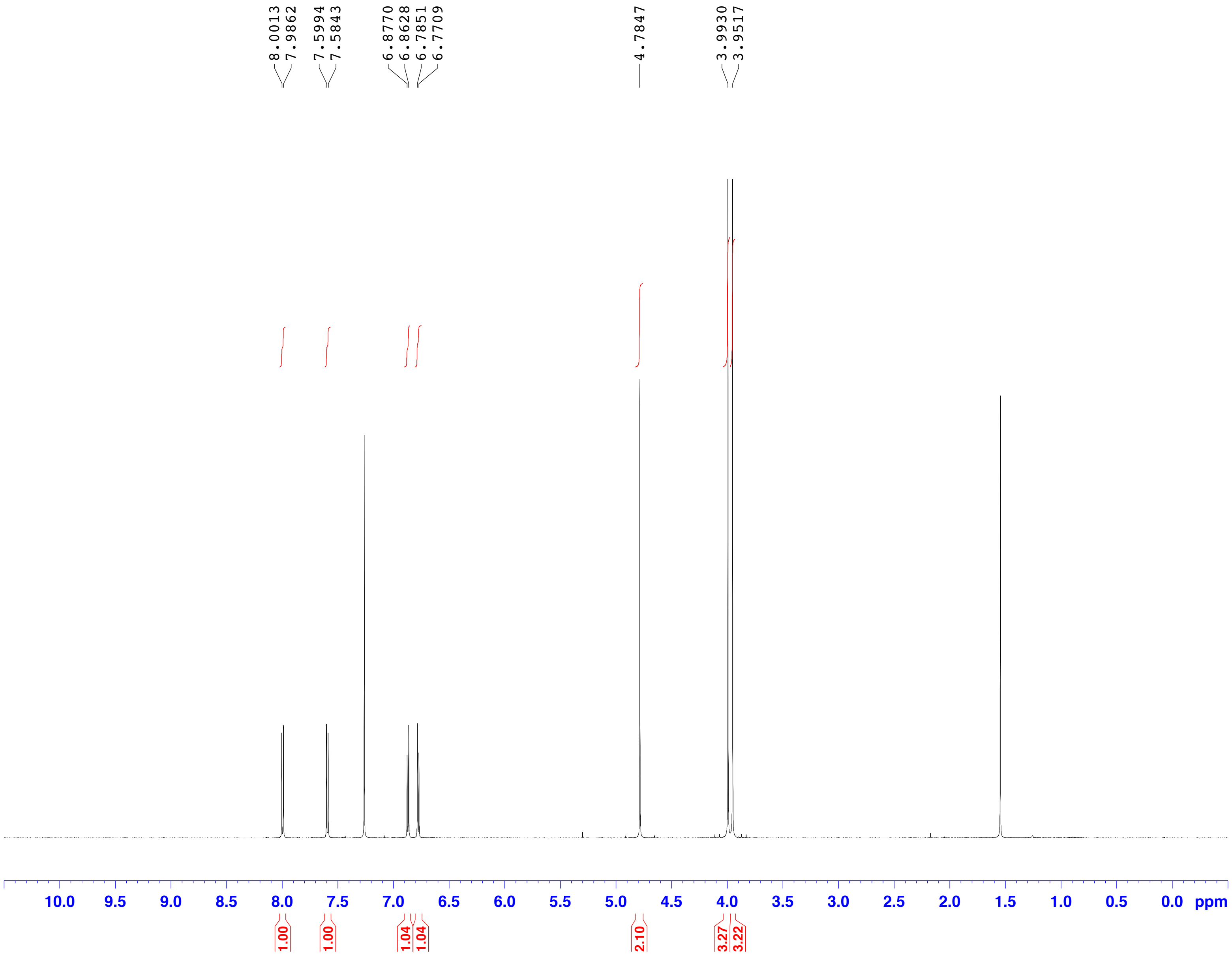


```
Current Data Parameters
NAME            YA1-1745-1
EXPNO                   10
PROCNO                   1

F2 - Acquisition Parameters
Date_             20200117
Time                 13.57
INSTRUM              spect
PROBHD   5 mm CPPBBO BB
PULPROG               zg30
TD                   65536
SOLVENT              CDCl3
NS                      16
DS                       2
SWH             12019.230 Hz
FIDRES           0.183399 Hz
AQ              2.7262976 sec
RG                    17.5
DW                  41.600 use
DE                   10.00 use
TE                   298.2 K
D1             1.00000000 sec
TD0                      1

======== CHANNEL f1 =======
SFO1        600.1337060 MHz
NUC1                    1H
P1                   12.00 use
PLW1         21.00000000 W

F2 - Processing parameters
SI                   65536
SF          600.1300149 MHz
WDW                     EM
SSB       0
LB                    0.30 Hz
GB        0
PC                    1.00
```

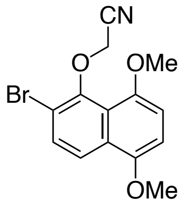

13j

^{13}C NMR (150 MHz, $CDCl_3$)

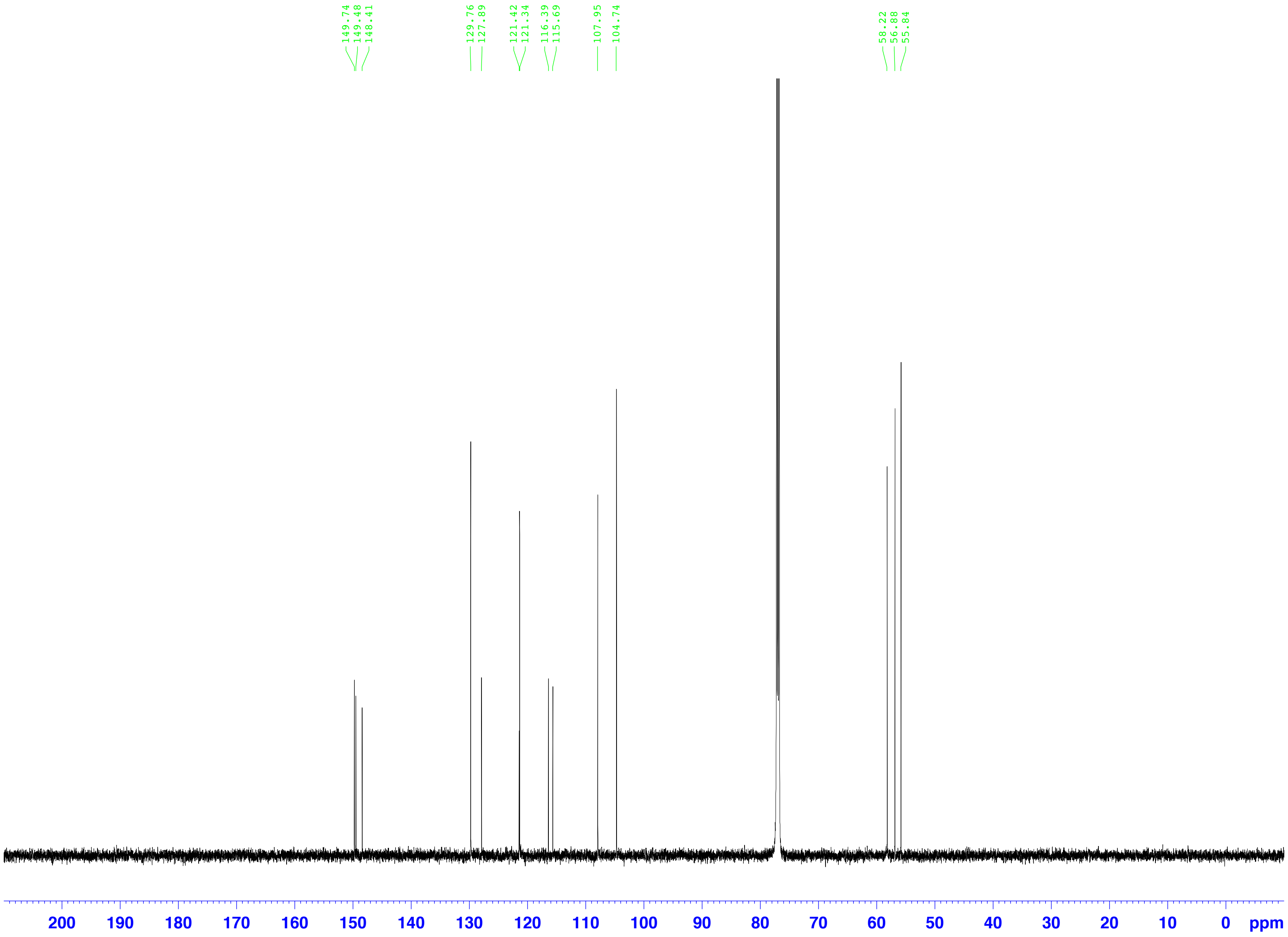


```
Current Data Parameters
NAME             YA1-1745-1
EXPNO                    11
PROCNO                    1

F2 - Acquisition Parameters
Date_              20200118
Time                  22.50
INSTRUM               spect
PROBHD    5 mm CPPBBO BB
PULPROG             zgpg30
TD                    65536
SOLVENT               CDCl3
NS                     3000
DS                        4
SWH              36057.691 Hz
FIDRES            0.550197 Hz
AQ               0.9087659 sec
RG                   175.56
DW                   13.867 use
DE                    18.00 use
TE                    298.2 K
D1               2.00000000 sec
D11              0.03000000 sec
TD0                       1

======== CHANNEL f1 =======
SFO1           150.9178981 MHz
NUC1                    13C
P1                    10.00 use
PLW1            80.00000000 W

======== CHANNEL f2 =======
SFO2           600.1324005 MHz
NUC2                     1H
CPDPRG[2            waltz16
PCPD2                 70.00 use
PLW2            13.43999958 W
PLW12            0.61714000 W
PLW13            0.31042001 W

F2 - Processing parameters
SI                    32768
SF             150.9028124 MHz
WDW                      EM
SSB      0
LB                     1.00 Hz
GB       0
PC                     1.40
```

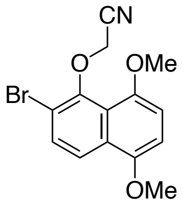

13j

^{1}H NMR (600 MHz, $CDCl_3$)

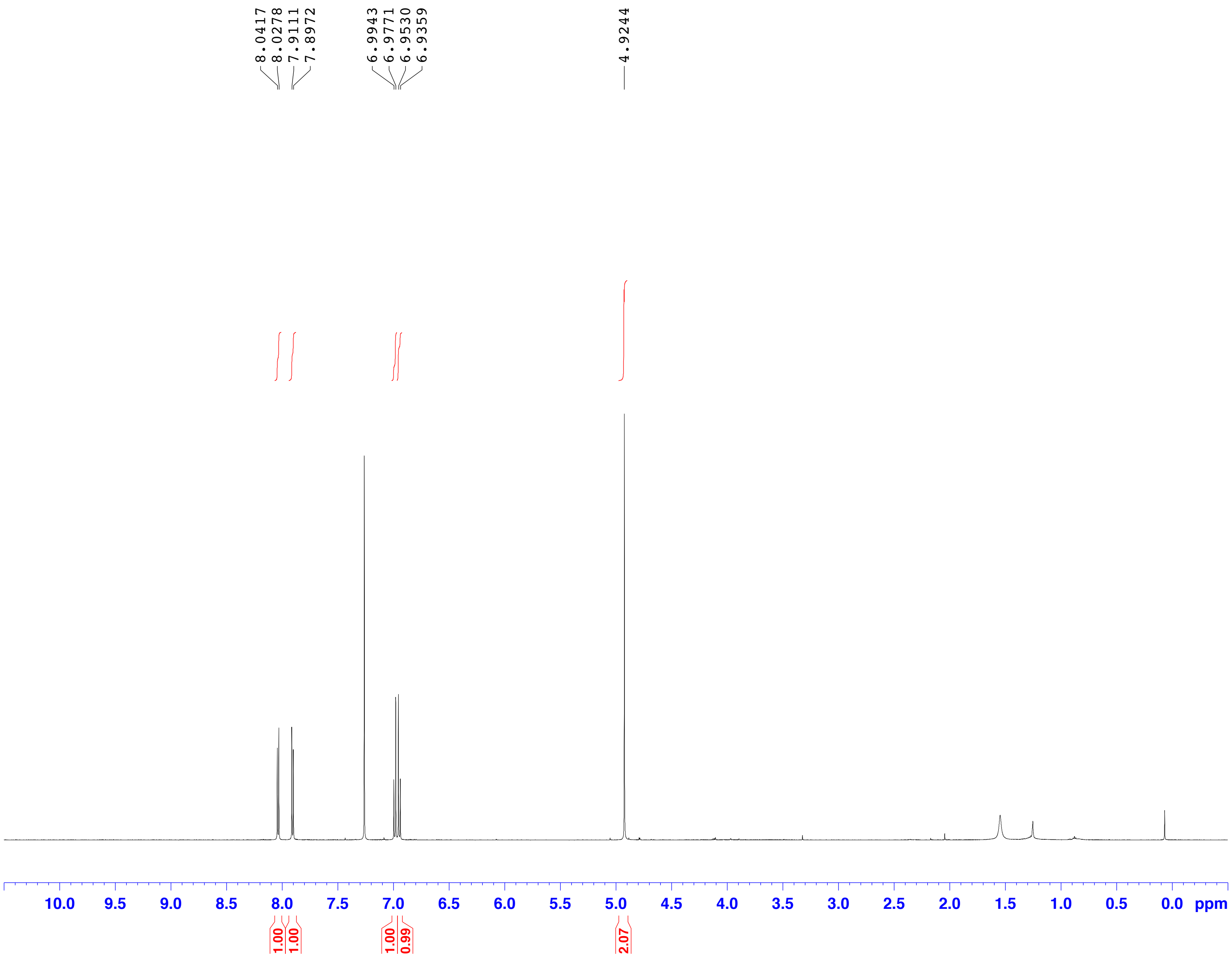

BRUKER

Current Data Parameters
NAME YA1-1761-crude
EXPNO 10
PROCNO 1

F2 - Acquisition Parameters
Date_ 20200131
Time 19.25
INSTRUM spect
PROBHD 5 mm CPPBBO BB
PULPROG zg30
TD 65536
SOLVENT CDCl3
NS 16
DS 2
SWH 12019.230 Hz
FIDRES 0.183399 Hz
AQ 2.7262976 sec
RG 31.94
DW 41.600 use
DE 10.00 use
TE 298.2 K
D1 1.00000000 sec
TD0 1

======== CHANNEL f1 =======
SFO1 600.1337060 MHz
NUC1 1H
P1 12.00 use
PLW1 21.00000000 W

F2 - Processing parameters
SI 65536
SF 600.1300150 MHz
WDW EM
SSB 0
LB 0.30 Hz
GB 0
PC 1.00

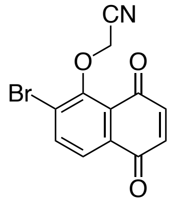

9j
(crude)

^{13}C NMR (150 MHz, $CDCl_3$)

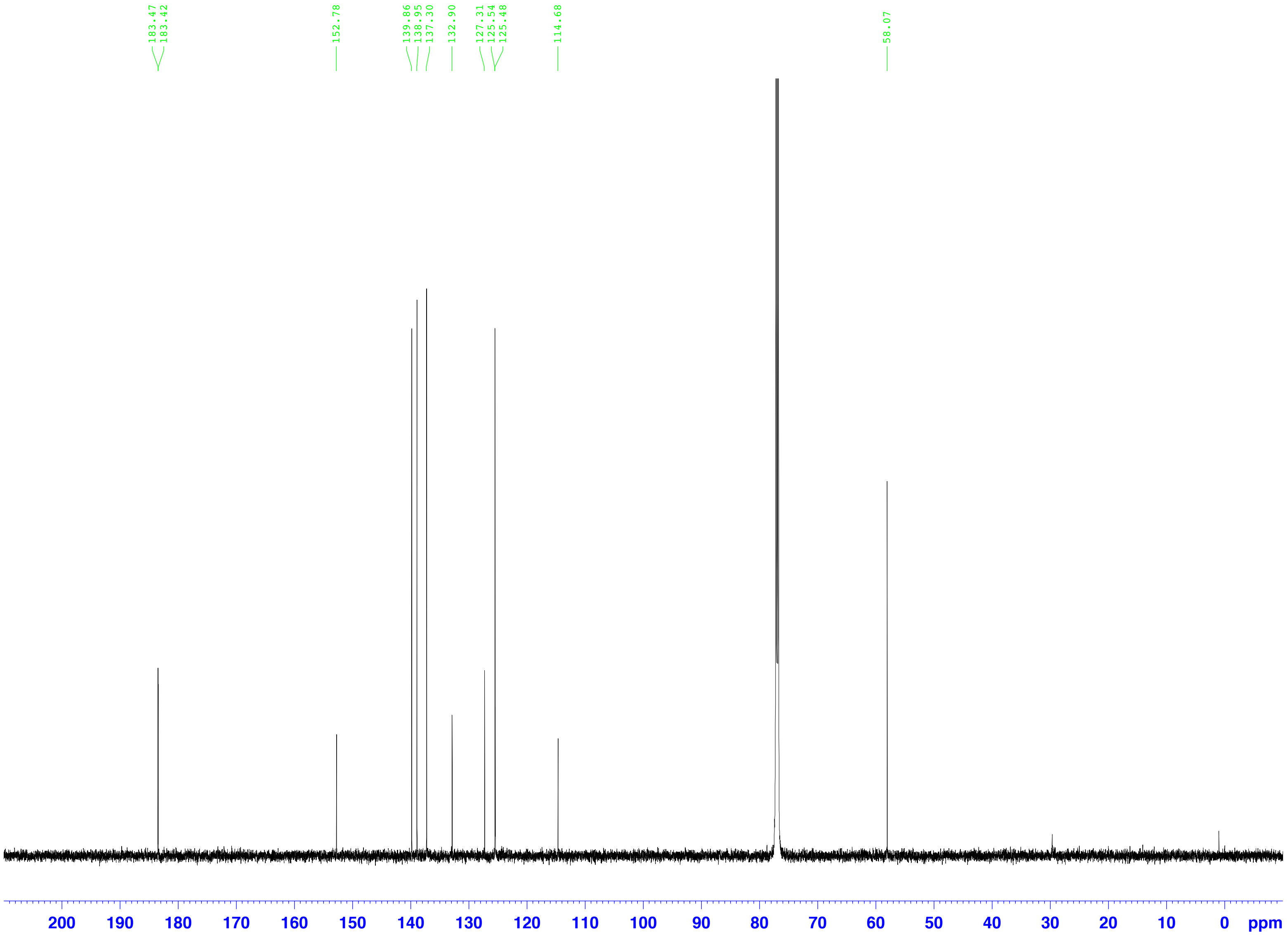


```
Current Data Parameters
NAME       YA1-1761-crude
EXPNO                  11
PROCNO                  1

F2 - Acquisition Parameters
Date_            20200201
Time                 6.28
INSTRUM             spect
PROBHD    5 mm CPPBBO BB
PULPROG           zgpg30
TD                 65536
SOLVENT            CDCl3
NS                  3600
DS                     4
SWH            36057.691 Hz
FIDRES          0.550197 Hz
AQ             0.9087659 sec
RG                175.56
DW                13.867 use
DE                 18.00 use
TE                 298.2 K
D1            2.00000000 sec
D11           0.03000000 sec
TD0                    1

======== CHANNEL f1 =======
SFO1         150.9178981 MHz
NUC1                 13C
P1                 10.00 use
PLW1         80.00000000 W

======== CHANNEL f2 =======
SFO2         600.1324005 MHz
NUC2                  1H
CPDPRG[2         waltz16
PCPD2              70.00 use
PLW2         13.43999958 W
PLW12         0.61714000 W
PLW13         0.31042001 W

F2 - Processing parameters
SI                 32768
SF           150.9028121 MHz
WDW                   EM
SSB      0
LB                  1.00 Hz
GB       0
PC                  1.40
```

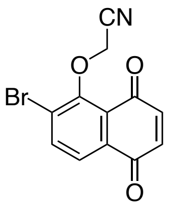

9j
(crude)

^{1}H NMR (600 MHz, $CDCl_3$)

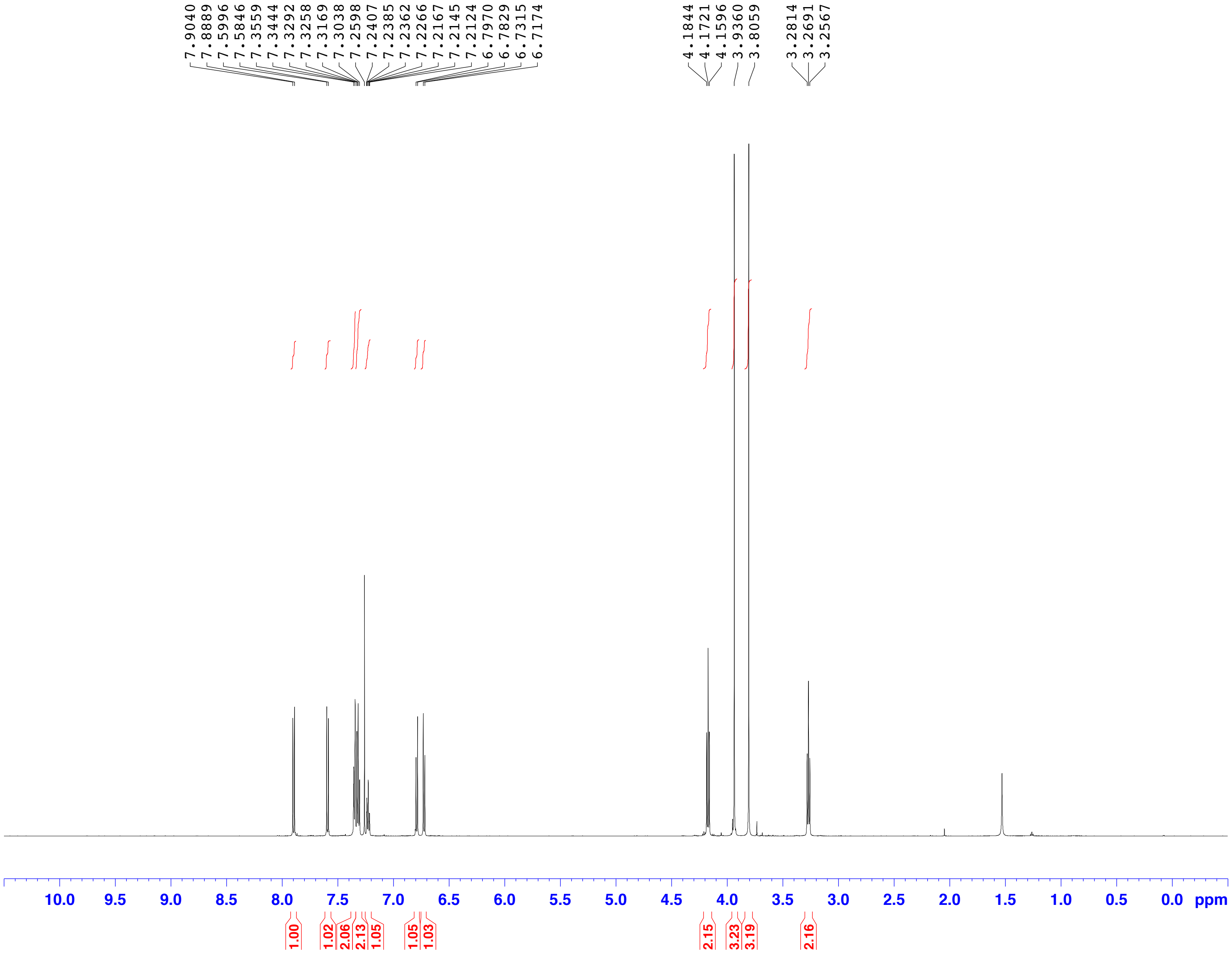


```
Current Data Parameters
NAME              YA1-1769-1
EXPNO                     10
PROCNO                     1

F2 - Acquisition Parameters
Date_               20200215
Time                   16.49
INSTRUM                spect
PROBHD     5 mm CPPBBO BB
PULPROG                 zg30
TD                     65536
SOLVENT                CDCl3
NS                        16
DS                         2
SWH               12019.230 Hz
FIDRES             0.183399 Hz
AQ                2.7262976 sec
RG                      17.5
DW                    41.600 use
DE                     10.00 use
TE                     298.2 K
D1               1.00000000 sec
TD0                        1

======== CHANNEL f1 =======
SFO1            600.1337060 MHz
NUC1                      1H
P1                     12.00 use
PLW1            21.00000000 W

F2 - Processing parameters
SI                     65536
SF              600.1300149 MHz
WDW                       EM
SSB       0
LB                      0.30 Hz
GB        0
PC                      1.00
```

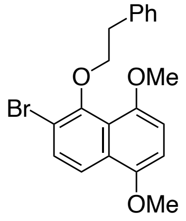

13k

^{13}C NMR (150 MHz, $CDCl_3$)

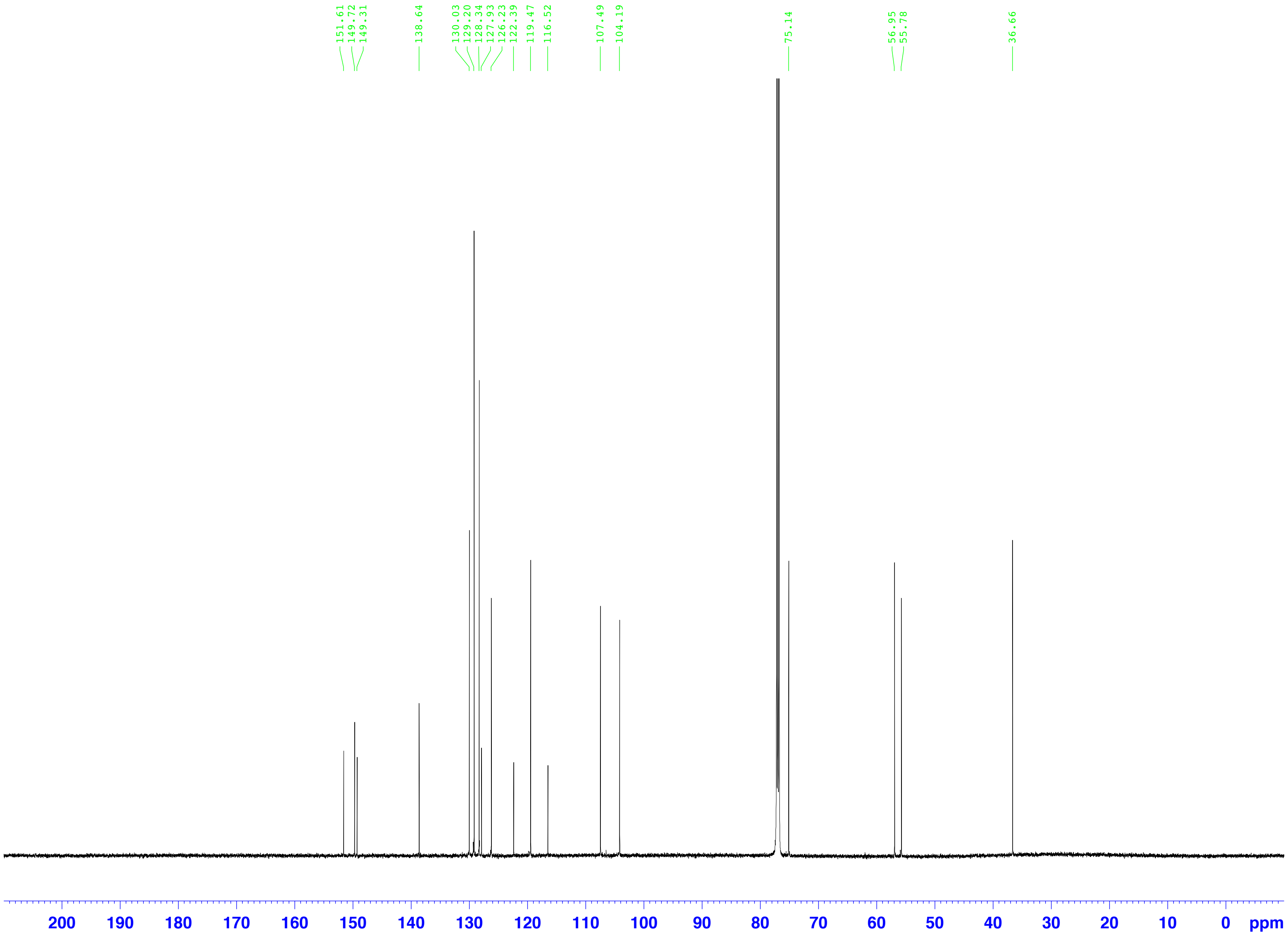


```
Current Data Parameters
NAME            YA1-1769-1
EXPNO                   11
PROCNO                   1

F2 - Acquisition Parameters
Date_             20200216
Time                  3.01
INSTRUM              spect
PROBHD    5 mm CPPBBO BB
PULPROG            zgpg30
TD                   65536
SOLVENT              CDCl3
NS                    3600
DS                       4
SWH             36057.691 Hz
FIDRES           0.550197 Hz
AQ              0.9087659 sec
RG                  175.56
DW                  13.867 use
DE                   18.00 use
TE                   298.2 K
D1              2.00000000 sec
D11             0.03000000 sec
TD0                      1

======== CHANNEL f1 =======
SFO1          150.9178981 MHz
NUC1                   13C
P1                   10.00 use
PLW1           80.00000000 W

======== CHANNEL f2 =======
SFO2          600.1324005 MHz
NUC2                    1H
CPDPRG[2           waltz16
PCPD2                70.00 use
PLW2           13.43999958 W
PLW12           0.61714000 W
PLW13           0.31042001 W

F2 - Processing parameters
SI                   32768
SF            150.9028132 MHz
WDW                     EM
SSB       0
LB                    1.00 Hz
GB        0
PC                    1.40
```

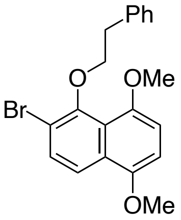

13k

^{1}H NMR (600 MHz, $CDCl_3$)

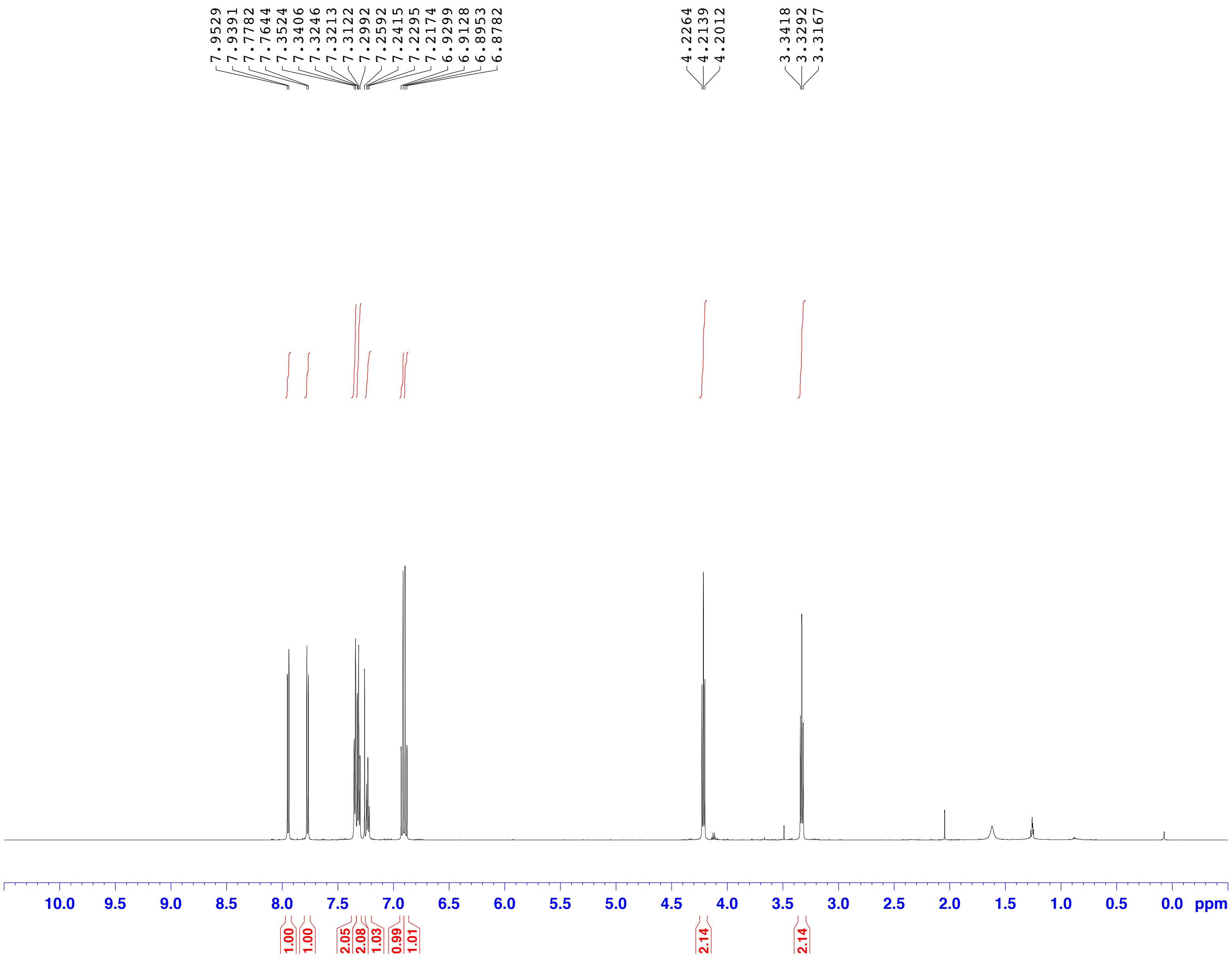


```
Current Data Parameters
NAME      YA1-1771-crude
EXPNO                 10
PROCNO                 1

F2 - Acquisition Parameters
Date_           20200217
Time               11.51
INSTRUM            spect
PROBHD    5 mm CPPBBO BB
PULPROG             zg30
TD                 65536
SOLVENT            CDCl3
NS                    16
DS                     2
SWH            12019.230 Hz
FIDRES          0.183399 Hz
AQ             2.7262976 sec
RG                 31.94
DW                41.600 use
DE                 10.00 use
TE                 298.1 K
D1            1.00000000 sec
TD0                    1

======== CHANNEL f1 =======
SFO1         600.1337060 MHz
NUC1                  1H
P1                 12.00 use
PLW1         21.00000000 W

F2 - Processing parameters
SI                 65536
SF           600.1300153 MHz
WDW                   EM
SSB       0
LB                  0.30 Hz
GB        0
PC                  1.00
```

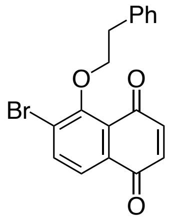

9k
(crude)

^{13}C NMR (150 MHz, $CDCl_3$)

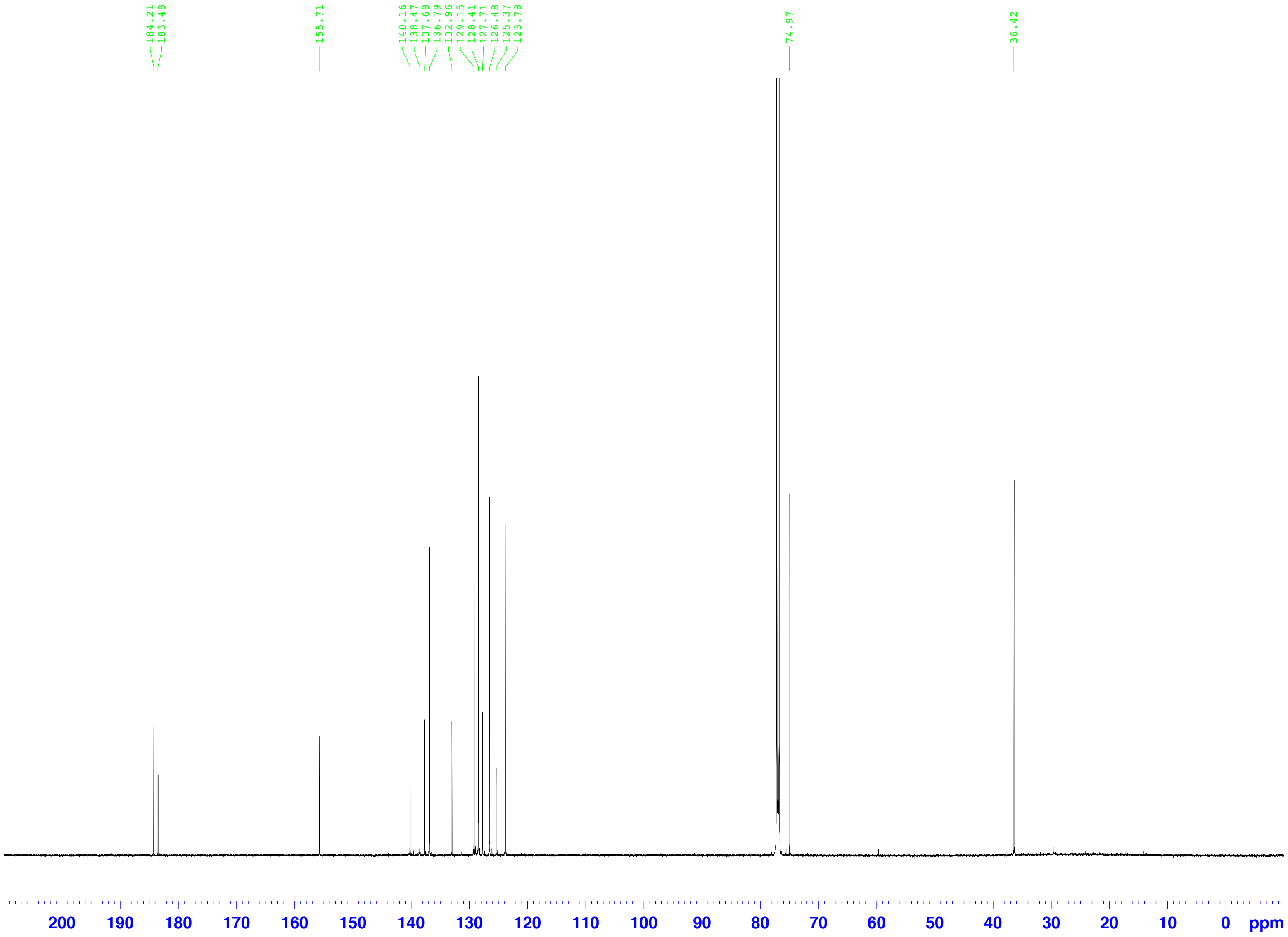


```
Current Data Parameters
NAME        YA1-1860-crude
EXPNO                   11
PROCNO                   1

F2 - Acquisition Parameters
Date_             20201122
Time                  0.01
INSTRUM              spect
PROBHD    5 mm CPPBBO BB
PULPROG            zgpg30
TD                   65536
SOLVENT              CDCl3
NS                    2400
DS                       4
SWH             36057.691 Hz
FIDRES           0.550197 Hz
AQ              0.9087659 sec
RG                  175.56
DW                  13.867 use
DE                   18.00 use
TE                   298.2 K
D1              2.00000000 sec
D11             0.03000000 sec
TD0                      1

======== CHANNEL f1 =======
SFO1          150.9178981 MHz
NUC1                   13C
P1                   10.00 use
PLW1           80.00000000 W

======== CHANNEL f2 =======
SFO2          600.1324005 MHz
NUC2                    1H
CPDPRG[2           waltz16
PCPD2                70.00 use
PLW2           13.43999958 W
PLW12           0.61714000 W
PLW13           0.31042001 W

F2 - Processing parameters
SI                   32768
SF            150.9028135 MHz
WDW                     EM
SSB      0
LB                    1.00 Hz
GB       0
PC                    1.40
```

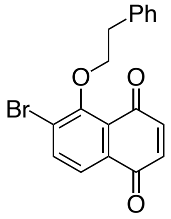

9k
(crude)

^{1}H NMR (600 MHz, $CDCl_3$)

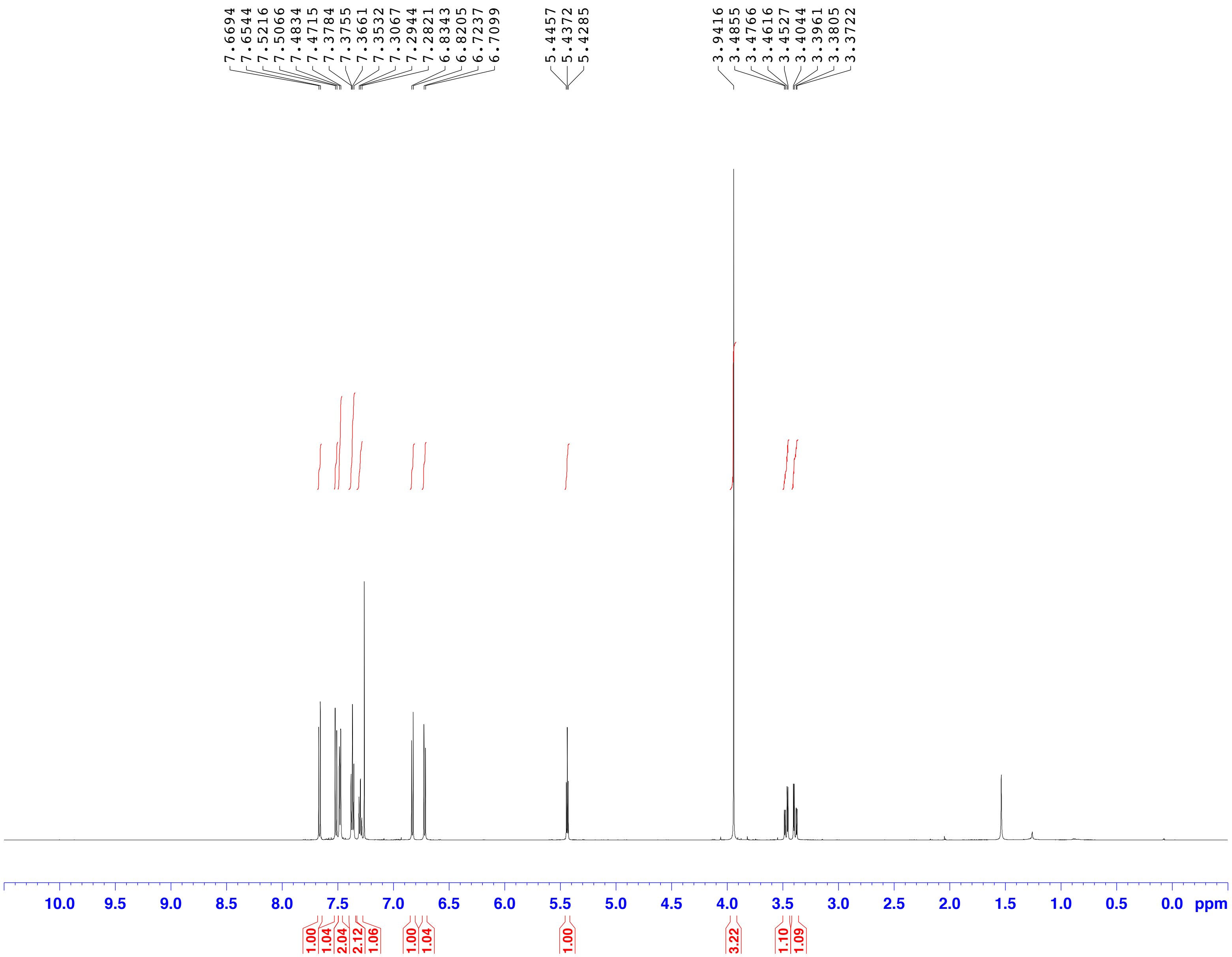

Current Data Parameters
NAME YA1-1772-a
EXPNO 10
PROCNO 1

F2 - Acquisition Parameters
Date_ 20200219
Time 13.50
INSTRUM spect
PROBHD 5 mm CPPBBO BB
PULPROG zg30
TD 65536
SOLVENT CDCl3
NS 16
DS 2
SWH 12019.230 Hz
FIDRES 0.183399 Hz
AQ 2.7262976 sec
RG 31.94
DW 41.600 use
DE 10.00 use
TE 298.2 K
D1 1.00000000 sec
TD0 1

======== CHANNEL f1 =======
SFO1 600.1337060 MHz
NUC1 1H
P1 12.00 use
PLW1 21.00000000 W

F2 - Processing parameters
SI 65536
SF 600.1300150 MHz
WDW EM
SSB 0
LB 0.30 Hz
GB 0
PC 1.00

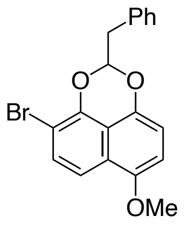

11k

^{13}C NMR (150 MHz, $CDCl_3$)

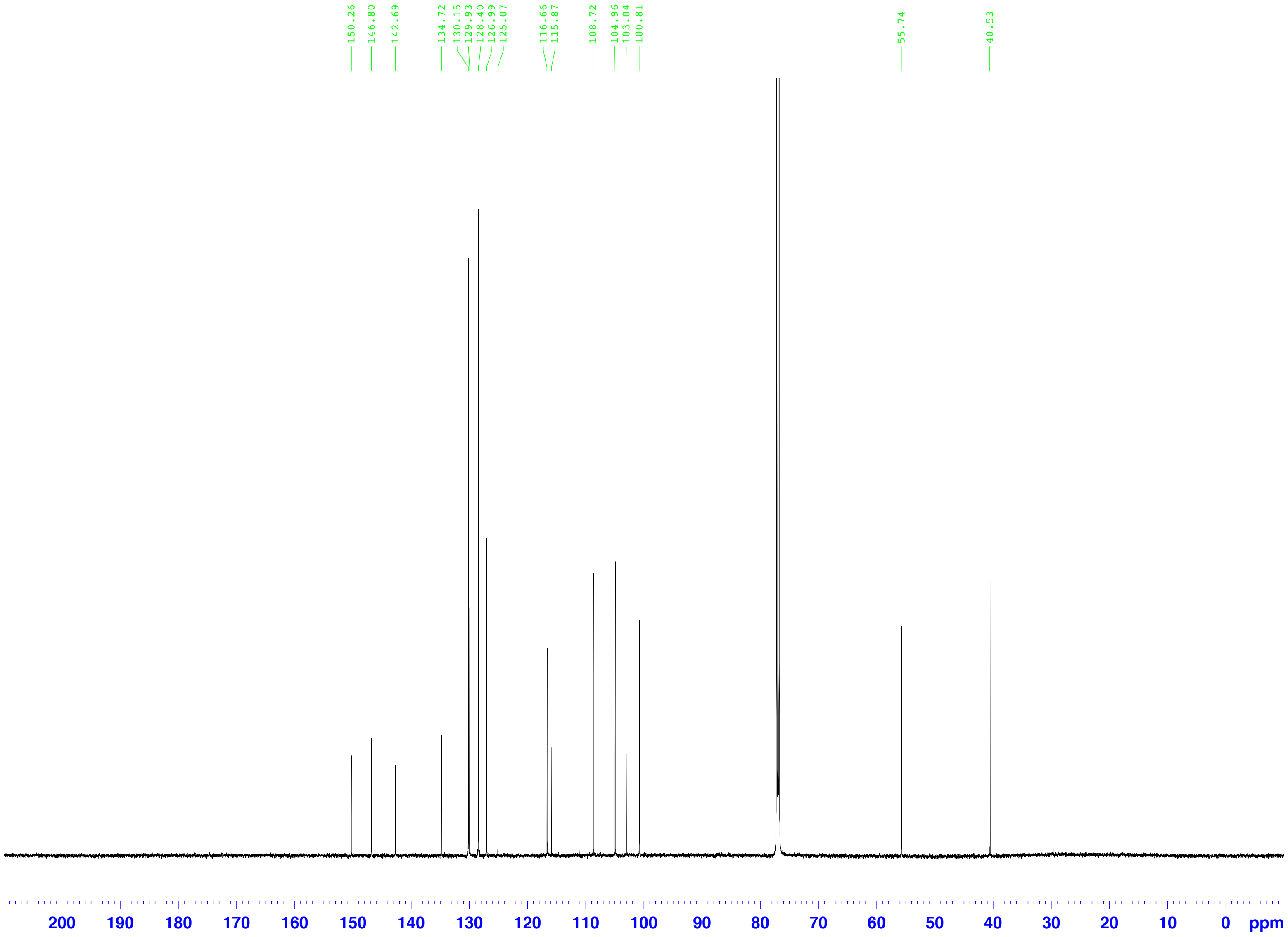

BRUKER

```
Current Data Parameters
NAME              YA1-1772-a
EXPNO                     11
PROCNO                     1

F2 - Acquisition Parameters
Date_               20200220
Time                    2.31
INSTRUM                spect
PROBHD   5 mm CPPBBO BB
PULPROG              zgpg30
TD                     65536
SOLVENT                CDCl3
NS                      3000
DS                         4
SWH              36057.691 Hz
FIDRES            0.550197 Hz
AQ               0.9087659 sec
RG                    175.56
DW                    13.867 use
DE                     18.00 use
TE                     298.2 K
D1              2.00000000 sec
D11             0.03000000 sec
TD0                        1

======== CHANNEL f1 =======
SFO1          150.9178981 MHz
NUC1                     13C
P1                     10.00 use
PLW1           80.00000000 W

======== CHANNEL f2 =======
SFO2          600.1324005 MHz
NUC2                      1H
CPDPRG[2             waltz16
PCPD2                  70.00 use
PLW2           13.43999958 W
PLW12           0.61714000 W
PLW13           0.31042001 W

F2 - Processing parameters
SI                     32768
SF            150.9028124 MHz
WDW                       EM
SSB      0
LB                      1.00 Hz
GB       0
PC                      1.40
```

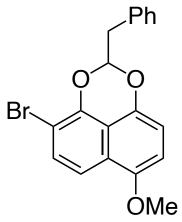

11k

^1H NMR (600 MHz, $CDCl_3$)

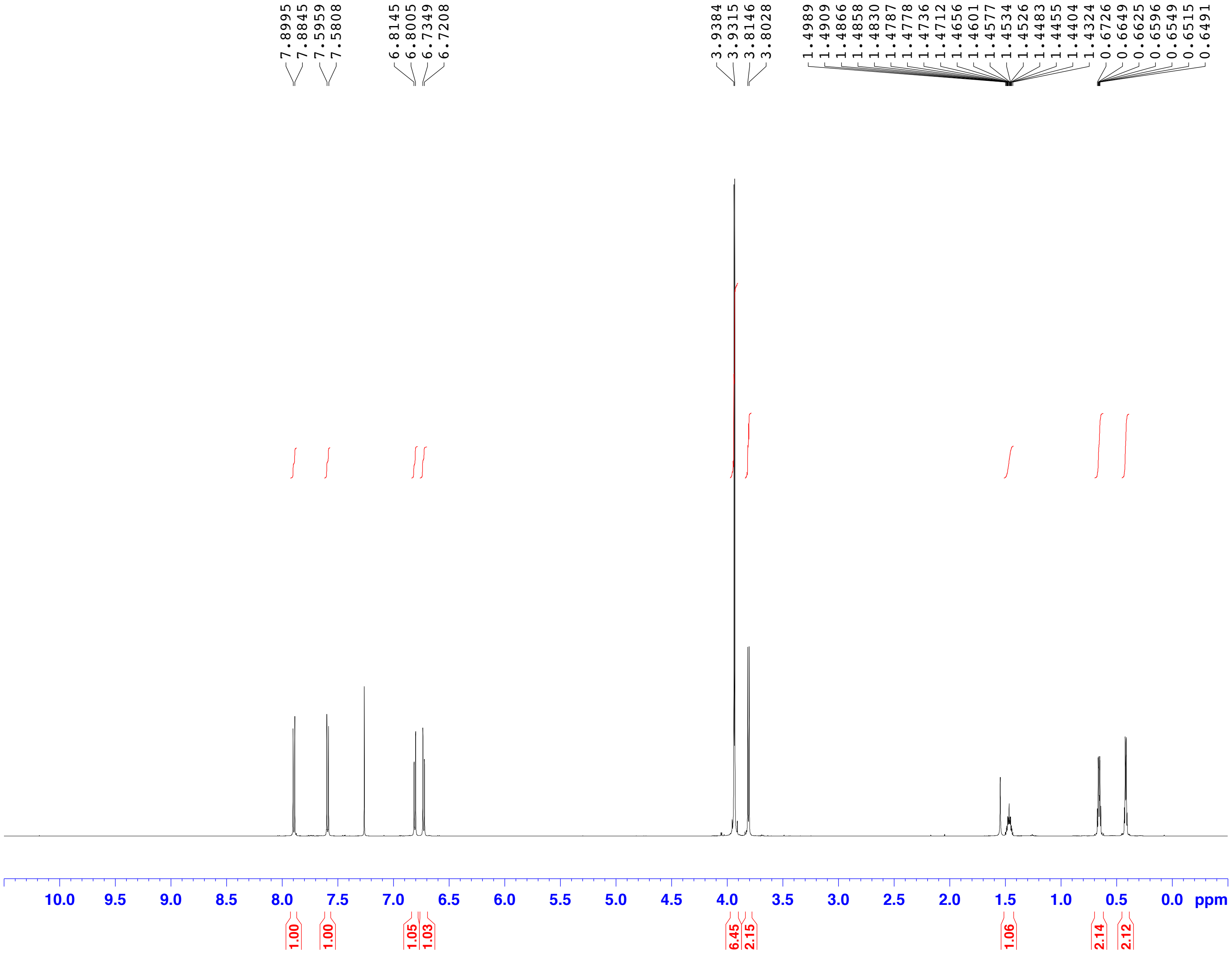

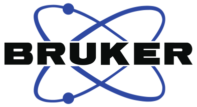

Current Data Parameters
NAME YA1-1773-2
EXPNO 30
PROCNO 1

F2 - Acquisition Parameters
Date_ 20200220
Time 17.34
INSTRUM spect
PROBHD 5 mm CPPBBO BB
PULPROG zg30
TD 65536
SOLVENT CDCl3
NS 16
DS 2
SWH 12019.230 Hz
FIDRES 0.183399 Hz
AQ 2.7262976 sec
RG 31.94
DW 41.600 use
DE 10.00 use
TE 298.0 K
D1 1.00000000 sec
TD0 1

======== CHANNEL f1 =======
SFO1 600.1337060 MHz
NUC1 1H
P1 12.00 use
PLW1 21.00000000 W

F2 - Processing parameters
SI 65536
SF 600.1300150 MHz
WDW EM
SSB 0
LB 0.30 Hz
GB 0
PC 1.00

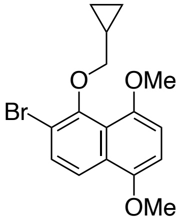

13l

^{13}C NMR (150 MHz, CDCl$_3$)

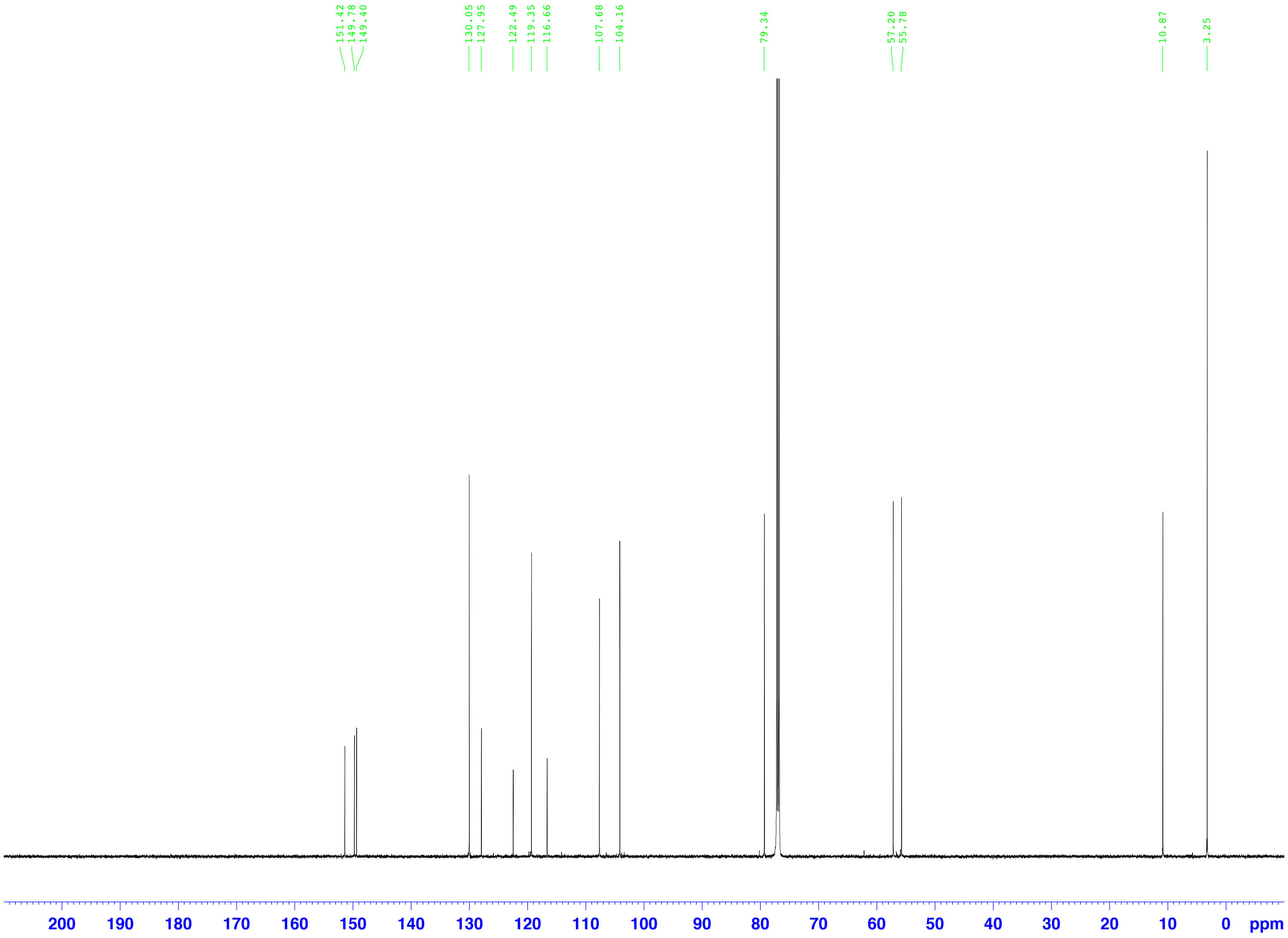

BRUKER

```
Current Data Parameters
NAME              YA1-1773-2
EXPNO                     21
PROCNO                     1

F2 - Acquisition Parameters
Date_             20200221
Time                  6.32
INSTRUM              spect
PROBHD    5 mm CPPBBO BB
PULPROG            zgpg30
TD                  65536
SOLVENT             CDCl3
NS                   3000
DS                      4
SWH            36057.691 Hz
FIDRES          0.550197 Hz
AQ             0.9087659 sec
RG                 175.56
DW                 13.867 use
DE                  18.00 use
TE                  298.2 K
D1             2.00000000 sec
D11            0.03000000 sec
TD0                     1

======== CHANNEL f1 =======
SFO1        150.9178981 MHz
NUC1                    13C
P1                    10.00 use
PLW1        80.00000000 W

======== CHANNEL f2 =======
SFO2        600.1324005 MHz
NUC2                     1H
CPDPRG[2            waltz16
PCPD2                 70.00 use
PLW2        13.43999958 W
PLW12        0.61714000 W
PLW13        0.31042001 W

F2 - Processing parameters
SI                  32768
SF          150.9028129 MHz
WDW                    EM
SSB      0
LB                   1.00 Hz
GB       0
PC                   1.40
```

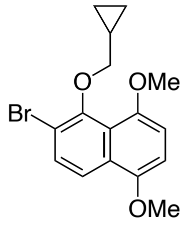

13l

^1H NMR (600 MHz, $CDCl_3$)

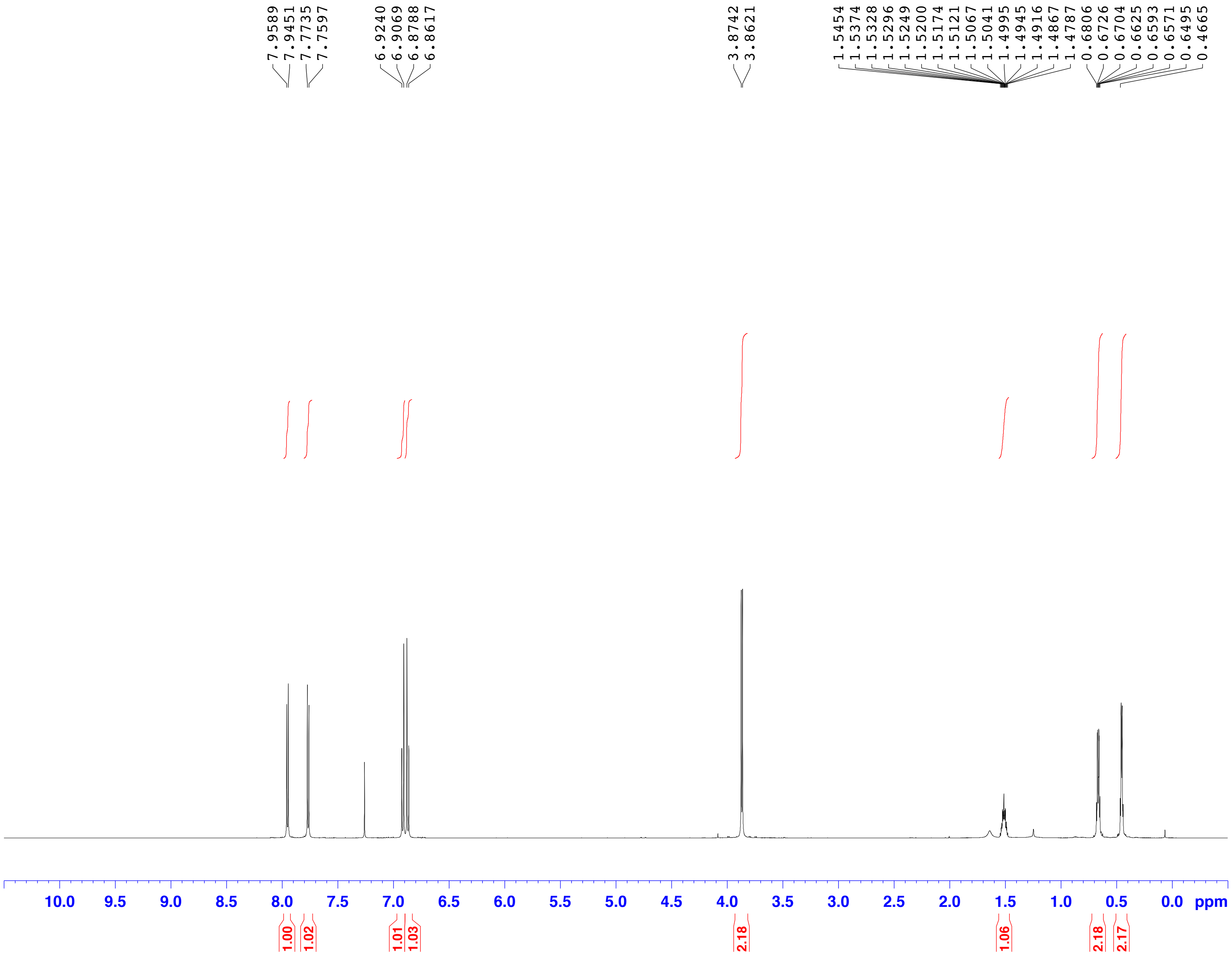

Current Data Parameters
NAME YA1-1867-crude
EXPNO 10
PROCNO 1

F2 - Acquisition Parameters
Date_ 20201209
Time 19.48
INSTRUM spect
PROBHD 5 mm CPPBBO BB
PULPROG zg30
TD 65536
SOLVENT CDCl3
NS 16
DS 2
SWH 12019.230 Hz
FIDRES 0.183399 Hz
AQ 2.7262976 sec
RG 17.5
DW 41.600 use
DE 10.00 use
TE 298.2 K
D1 1.00000000 sec
TD0 1

======== CHANNEL f1 =======
SFO1 600.1337060 MHz
NUC1 1H
P1 12.00 use
PLW1 21.00000000 W

F2 - Processing parameters
SI 65536
SF 600.1300148 MHz
WDW EM
SSB 0
LB 0.30 Hz
GB 0
PC 1.00

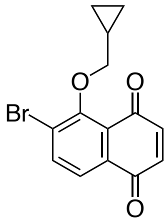

9l
(crude)

^{13}C NMR (150 MHz, $CDCl_3$)

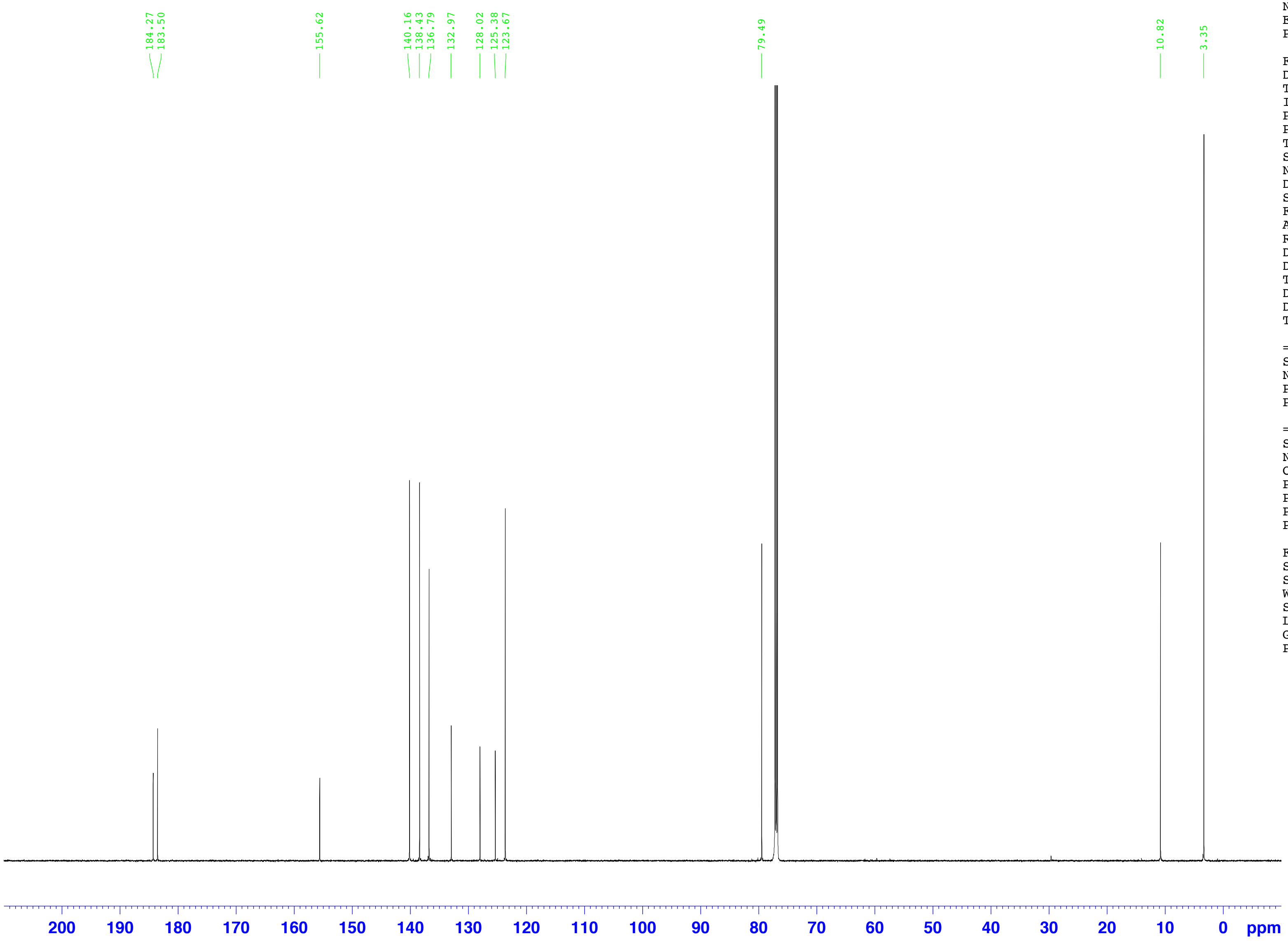

Current Data Parameters
NAME YA1-1867-crude
EXPNO 11
PROCNO 1

F2 - Acquisition Parameters
Date_ 20201210
Time 0.04
INSTRUM spect
PROBHD 5 mm CPPBBO BB
PULPROG zgpg30
TD 65536
SOLVENT CDCl3
NS 2400
DS 4
SWH 36057.691 Hz
FIDRES 0.550197 Hz
AQ 0.9087659 sec
RG 175.56
DW 13.867 use
DE 18.00 use
TE 298.2 K
D1 2.00000000 sec
D11 0.03000000 sec
TD0 1

======== CHANNEL f1 =======
SFO1 150.9178981 MHz
NUC1 13C
P1 10.00 use
PLW1 80.00000000 W

======== CHANNEL f2 =======
SFO2 600.1324005 MHz
NUC2 1H
CPDPRG[2 waltz16
PCPD2 70.00 use
PLW2 13.43999958 W
PLW12 0.61714000 W
PLW13 0.31042001 W

F2 - Processing parameters
SI 32768
SF 150.9028142 MHz
WDW EM
SSB 0
LB 1.00 Hz
GB 0
PC 1.40

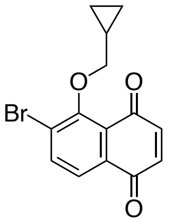

9l
(crude)

^{1}H NMR (600 MHz, $CDCl_3$)

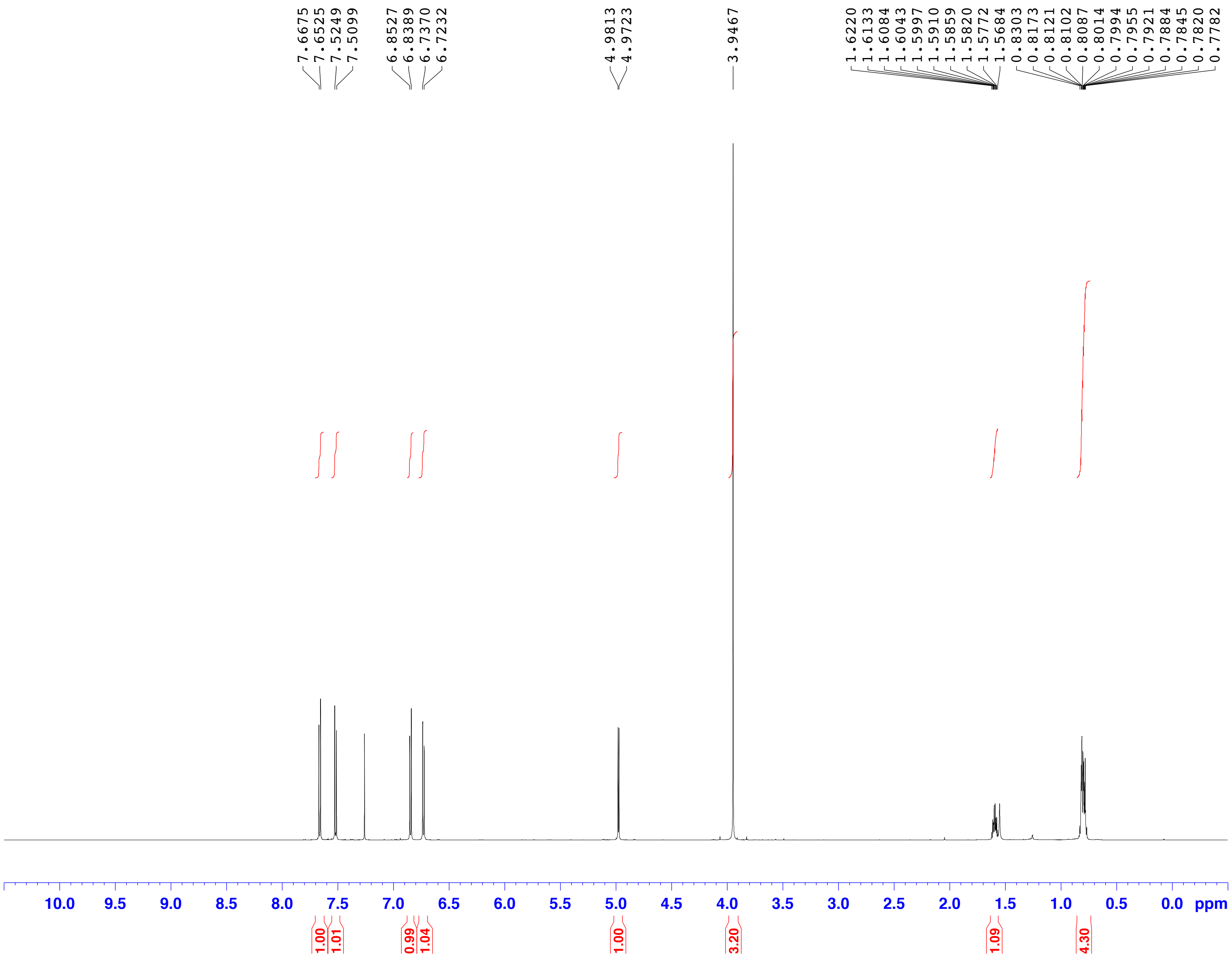


```
Current Data Parameters
NAME             YA1-1776-c
EXPNO                    10
PROCNO                    1

F2 - Acquisition Parameters
Date_              20200222
Time                  16.50
INSTRUM               spect
PROBHD    5 mm CPPBBO BB
PULPROG                zg30
TD                    65536
SOLVENT               CDCl3
NS                       16
DS                        2
SWH               12019.230 Hz
FIDRES             0.183399 Hz
AQ                2.7262976 sec
RG                     17.5
DW                   41.600 use
DE                    10.00 use
TE                    298.2 K
D1               1.00000000 sec
TD0                       1

======== CHANNEL f1 =======
SFO1            600.1337060 MHz
NUC1                     1H
P1                    12.00 use
PLW1            21.00000000 W

F2 - Processing parameters
SI                    65536
SF              600.1300149 MHz
WDW                      EM
SSB       0
LB                     0.30 Hz
GB        0
PC                     1.00
```

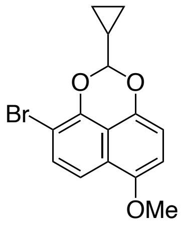

11l

^{13}C NMR (150 MHz, $CDCl_3$)

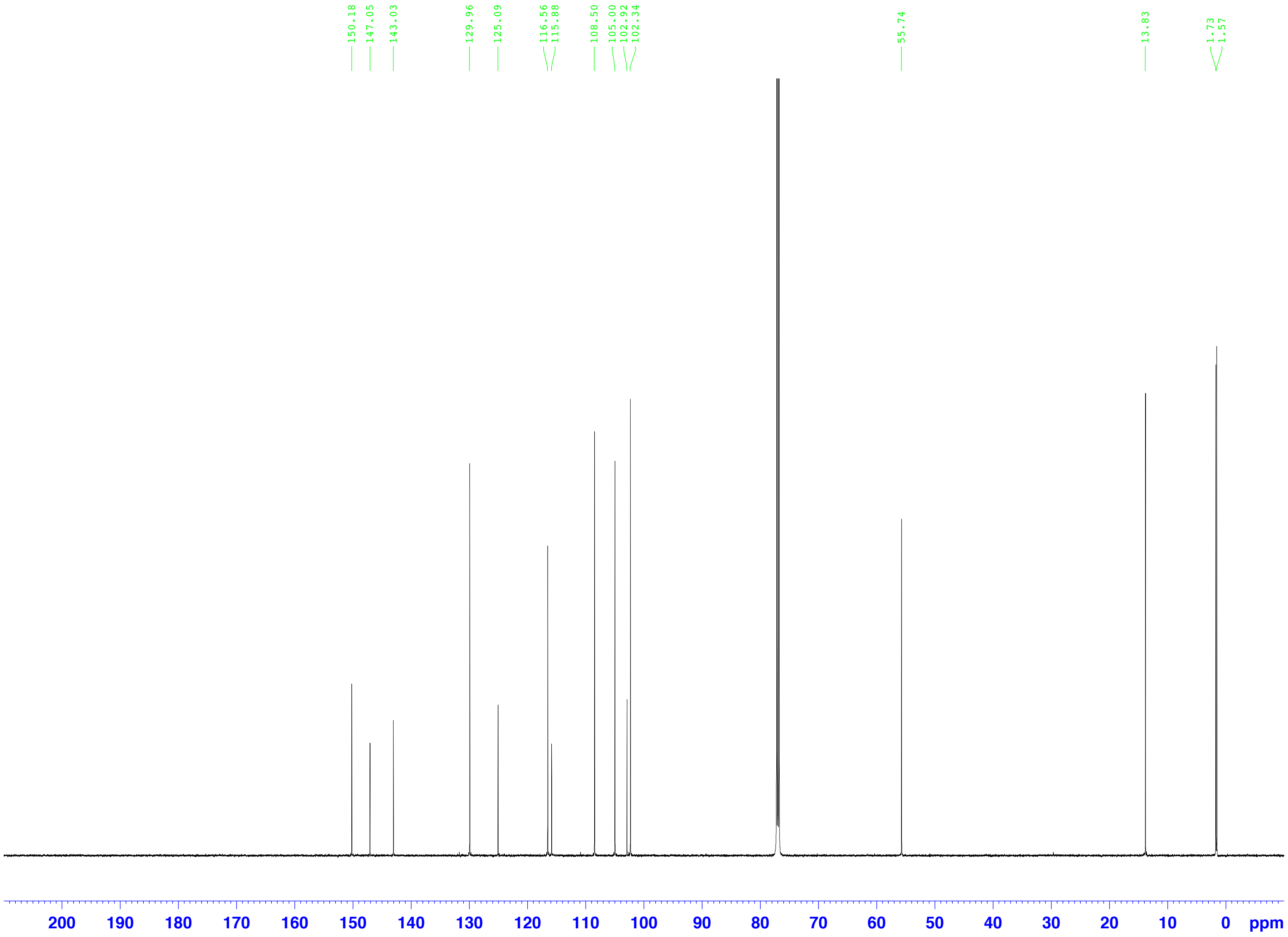


```
Current Data Parameters
NAME              YA1-1776-c
EXPNO                     11
PROCNO                     1

F2 - Acquisition Parameters
Date_               20200225
Time                    2.31
INSTRUM                spect
PROBHD    5 mm CPPBBO BB
PULPROG              zgpg30
TD                     65536
SOLVENT                CDCl3
NS                      3000
DS                         4
SWH               36057.691 Hz
FIDRES             0.550197 Hz
AQ                0.9087659 sec
RG                    175.56
DW                    13.867 use
DE                     18.00 use
TE                     298.2 K
D1                2.00000000 sec
D11               0.03000000 sec
TD0                        1

======== CHANNEL f1 =======
SFO1            150.9178981 MHz
NUC1                     13C
P1                     10.00 use
PLW1             80.00000000 W

======== CHANNEL f2 =======
SFO2            600.1324005 MHz
NUC2                      1H
CPDPRG[2             waltz16
PCPD2                  70.00 use
PLW2             13.43999958 W
PLW12             0.61714000 W
PLW13             0.31042001 W

F2 - Processing parameters
SI                     32768
SF              150.9028137 MHz
WDW                       EM
SSB       0
LB                      1.00 Hz
GB        0
PC                      1.40
```

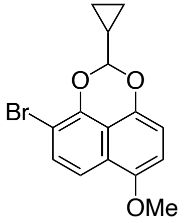

11l

^{1}H NMR (600 MHz, $CDCl_3$)

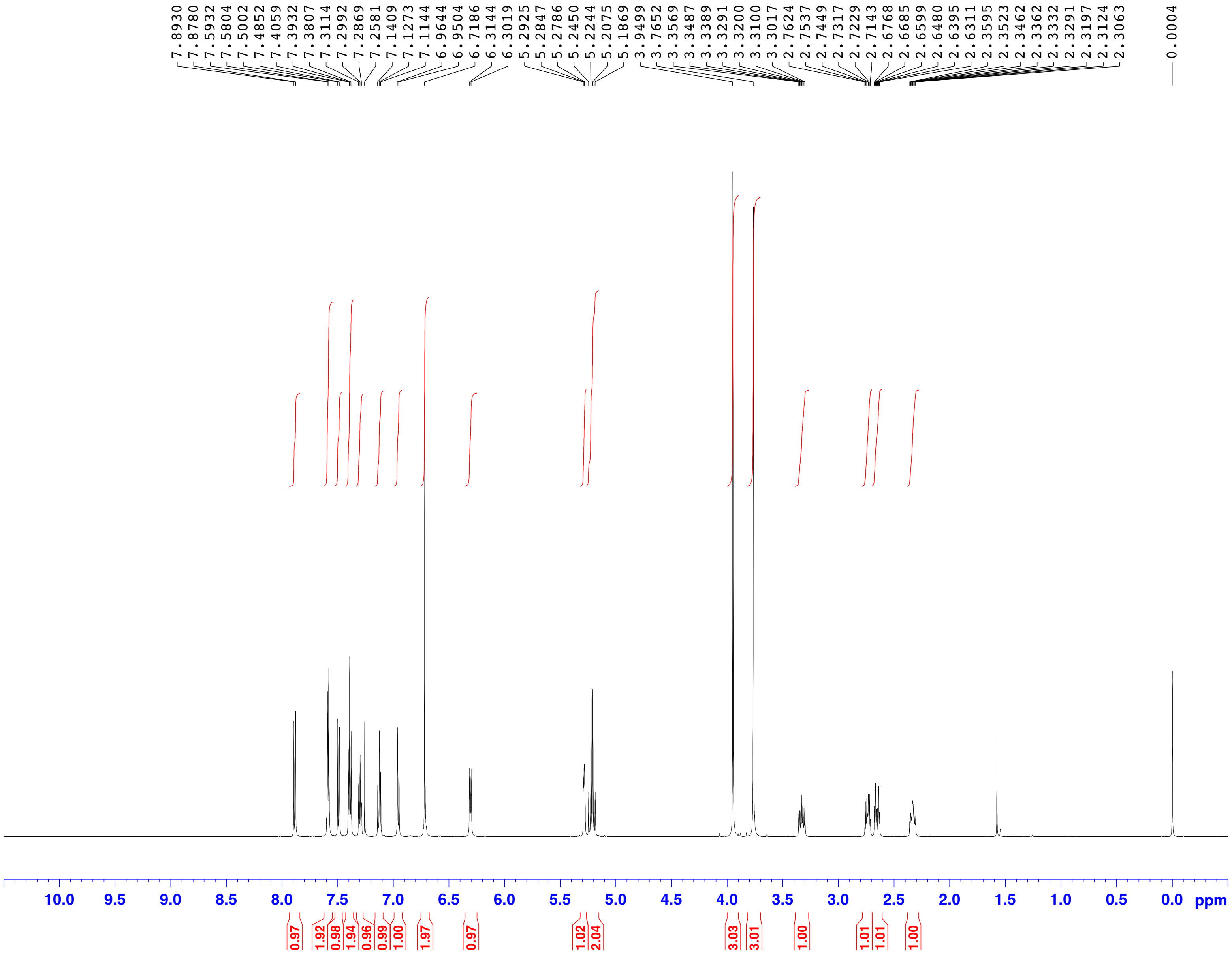

Current Data Parameters
NAME DO1-294-recryst
EXPNO 10
PROCNO 1

F2 - Acquisition Parameters
Date_ 20220208
Time 16.45
INSTRUM spect
PROBHD 5 mm CPPBBO BB
PULPROG zg30
TD 65536
SOLVENT CDCl3
NS 16
DS 2
SWH 12019.230 Hz
FIDRES 0.183399 Hz
AQ 2.7262976 sec
RG 31.94
DW 41.600 usec
DE 10.00 usec
TE 298.2 K
D1 1.00000000 sec
TD0 1

======== CHANNEL f1 ========
SFO1 600.1337060 MHz
NUC1 1H
P1 12.00 usec
PLW1 21.00000000 W

F2 - Processing parameters
SI 65536
SF 600.1300160 MHz
WDW EM
SSB 0
LB 0.30 Hz
GB 0
PC 1.00

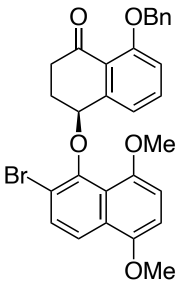

(*S*)-**20**

^{13}C NMR (150 MHz, $CDCl_3$)

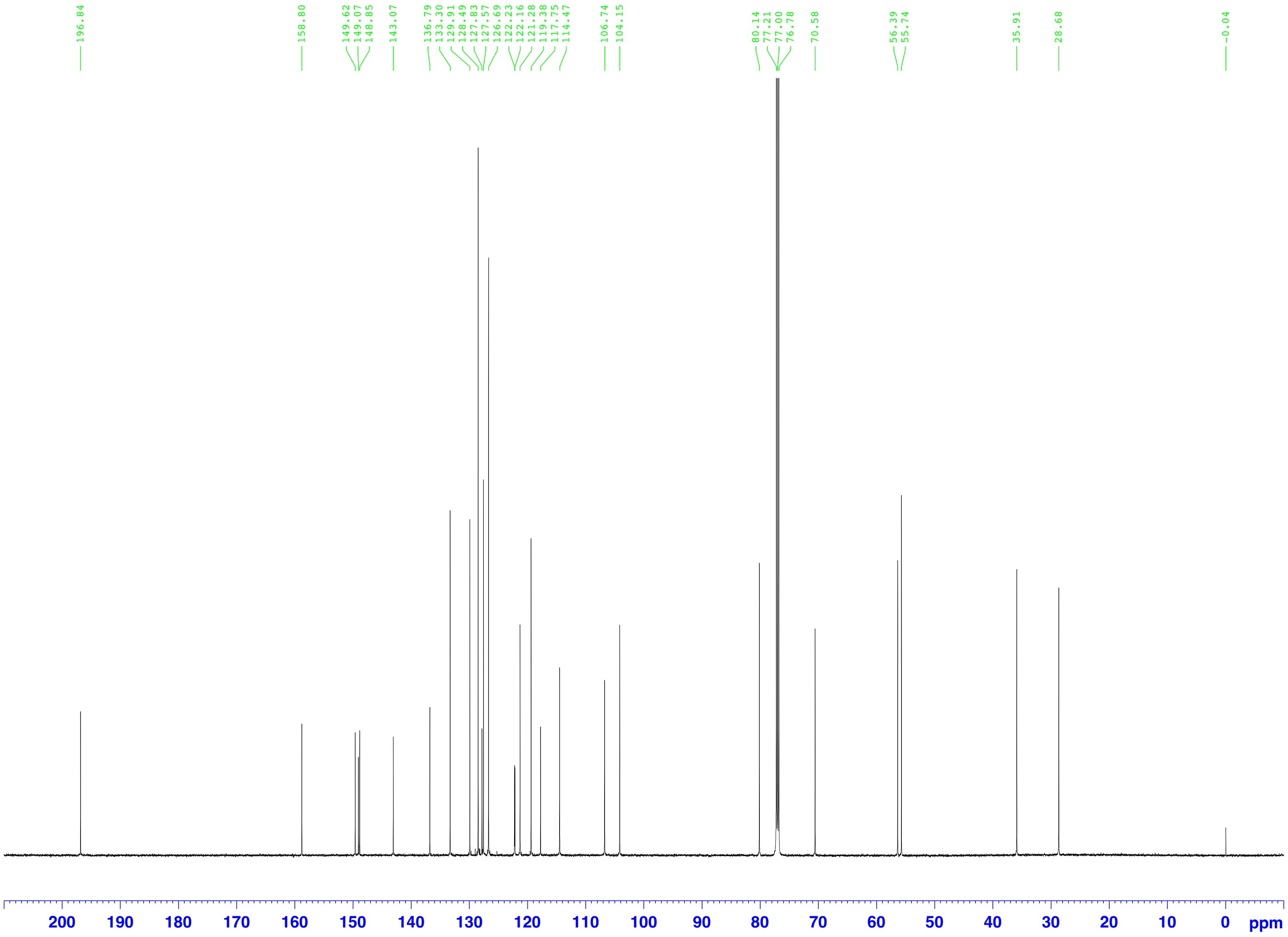

Current Data Parameters
NAME DO1-50-1st-data
EXPNO 12
PROCNO 1

F2 - Acquisition Parameters
Date_ 20210114
Time 5.00
INSTRUM spect
PROBHD 5 mm CPPBBO BB
PULPROG zgpg30
TD 65536
SOLVENT CDCl3
NS 1024
DS 4
SWH 36057.691 Hz
FIDRES 0.550197 Hz
AQ 0.9087659 sec
RG 175.56
DW 13.867 usec
DE 18.00 usec
TE 298.2 K
D1 2.00000000 sec
D11 0.03000000 sec
TD0 1

======== CHANNEL f1 ========
SFO1 150.9178981 MHz
NUC1 13C
P1 10.00 usec
PLW1 80.00000000 W

======== CHANNEL f2 ========
SFO2 600.1324005 MHz
NUC2 1H
CPDPRG[2 waltz16
PCPD2 70.00 usec
PLW2 13.43999958 W
PLW12 0.61714000 W
PLW13 0.31042001 W

F2 - Processing parameters
SI 32768
SF 150.9028179 MHz
WDW EM
SSB 0
LB 1.00 Hz
GB 0
PC 1.40

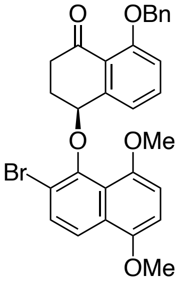

(*S*)-**20**

^{1}H NMR (600 MHz, $CDCl_3$)

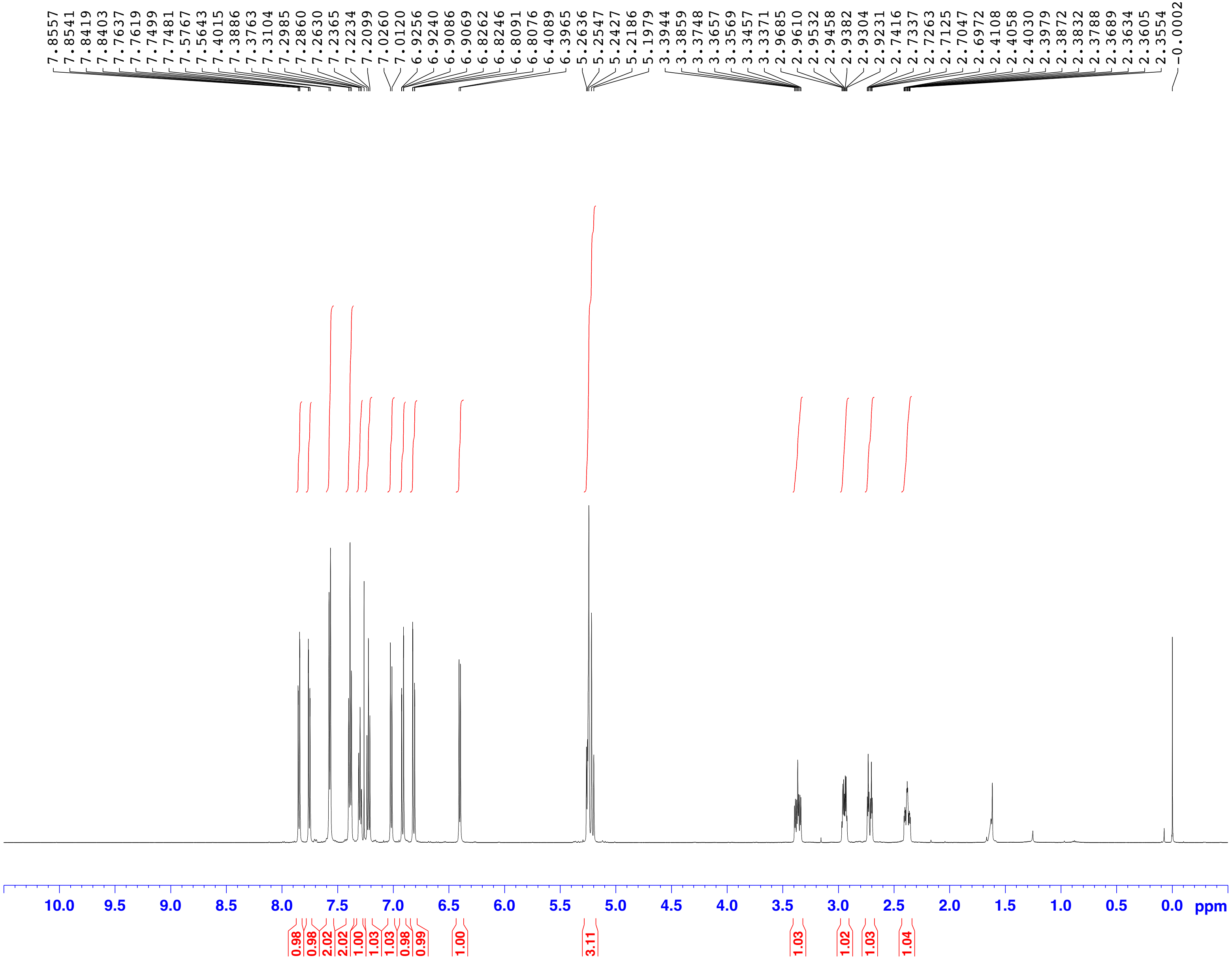

BRUKER

Current Data Parameters
NAME DO1-291-ptlc
EXPNO 10
PROCNO 1

F2 - Acquisition Parameters
Date_ 20220126
Time 20.04
INSTRUM spect
PROBHD 5 mm CPPBBO BB
PULPROG zg30
TD 65536
SOLVENT CDCl3
NS 16
DS 2
SWH 12019.230 Hz
FIDRES 0.183399 Hz
AQ 2.7262976 sec
RG 17.5
DW 41.600 usec
DE 10.00 usec
TE 298.1 K
D1 1.00000000 sec
TD0 1

======== CHANNEL f1 ========
SFO1 600.1337060 MHz
NUC1 1H
P1 12.00 usec
PLW1 21.00000000 W

F2 - Processing parameters
SI 65536
SF 600.1300126 MHz
WDW EM
SSB 0
LB 0.30 Hz
GB 0
PC 1.00

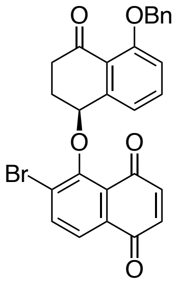

(*S*)-**17**

^{13}C NMR (150 MHz, $CDCl_3$)

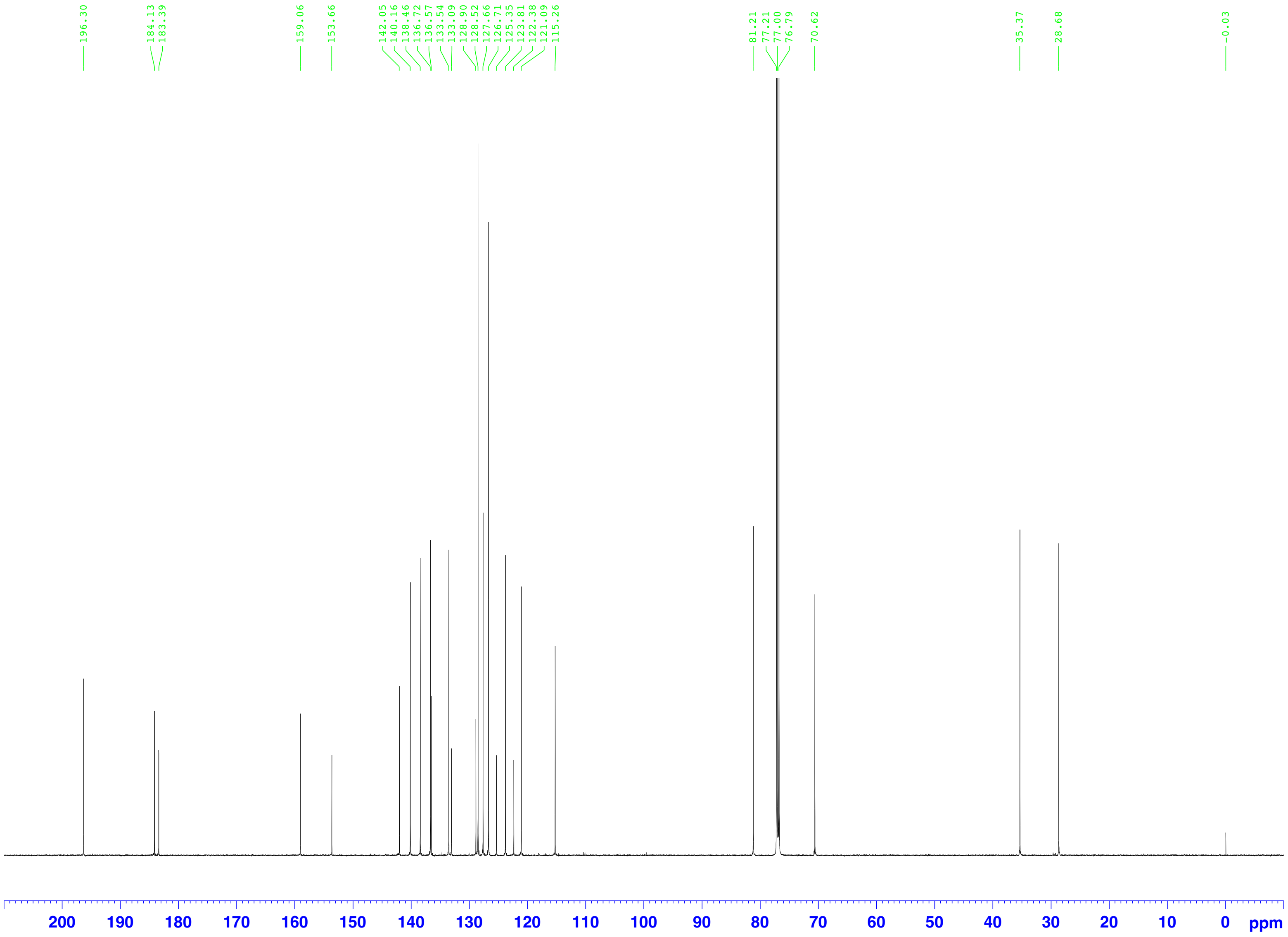


```
Current Data Parameters
NAME          DO1-291-ptlc
EXPNO                   12
PROCNO                   1

F2 - Acquisition Parameters
Date_             20220127
Time                  4.58
INSTRUM              spect
PROBHD     5 mm CPPBBO BB
PULPROG            zgpg30
TD                   65536
SOLVENT              CDCl3
NS                    4096
DS                       4
SWH              36057.691 Hz
FIDRES            0.550197 Hz
AQ               0.9087659 sec
RG                  175.56
DW                  13.867 usec
DE                   18.00 usec
TE                   298.1 K
D1              2.00000000 sec
D11             0.03000000 sec
TD0                      1

======== CHANNEL f1 ========
SFO1        150.9178981 MHz
NUC1                   13C
P1                   10.00 usec
PLW1           80.00000000 W

======== CHANNEL f2 ========
SFO2        600.1324005 MHz
NUC2                    1H
CPDPRG[2           waltz16
PCPD2                70.00 usec
PLW2           13.43999958 W
PLW12           0.61714000 W
PLW13           0.31042001 W

F2 - Processing parameters
SI                   32768
SF          150.9028163 MHz
WDW                     EM
SSB                      0
LB                    1.00 Hz
GB                       0
PC                    1.40
```

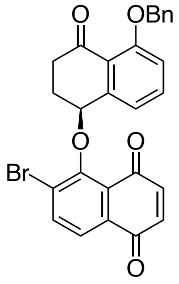

(*S*)-**17**

^{1}H NMR (600 MHz, $CDCl_3$)

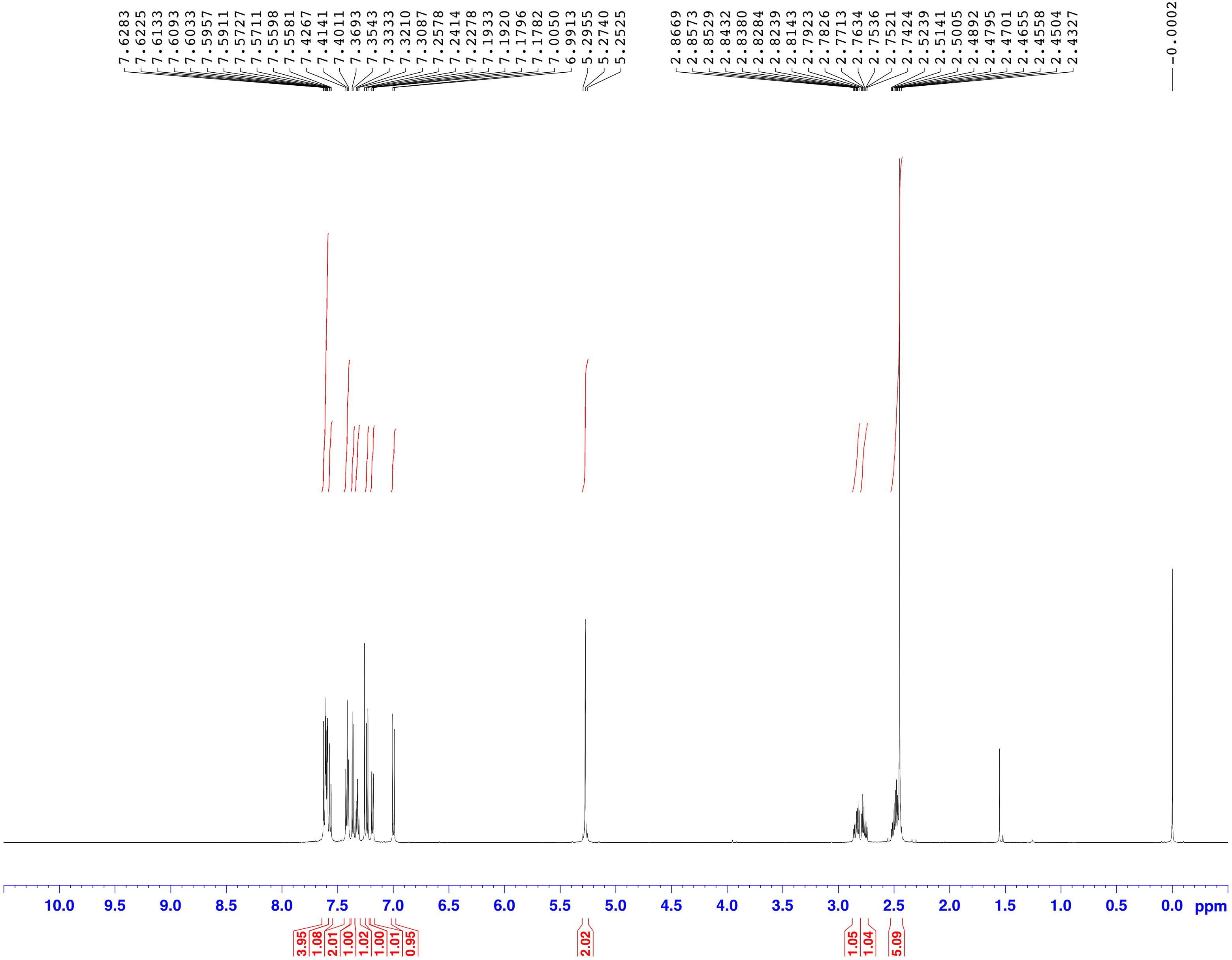


```
Current Data Parameters
NAME          DO1-59-ptlc
EXPNO                  30
PROCNO                  1

F2 - Acquisition Parameters
Date_            20210204
Time                19.16
INSTRUM             spect
PROBHD   5 mm CPPBBO BB
PULPROG              zg30
TD                  65536
SOLVENT             CDCl3
NS                     16
DS                      2
SWH             12019.230 Hz
FIDRES           0.183399 Hz
AQ              2.7262976 sec
RG                  31.94
DW                 41.600 usec
DE                  10.00 usec
TE                  298.2 K
D1             1.00000000 sec
TD0                     1

======== CHANNEL f1 ========
SFO1          600.1337060 MHz
NUC1                   1H
P1                  12.00 usec
PLW1          21.00000000 W

F2 - Processing parameters
SI                  65536
SF            600.1300163 MHz
WDW                    EM
SSB                     0
LB                   0.30 Hz
GB                      0
PC                   1.00
```

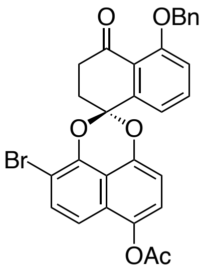

(*S*)-**22**

^{13}C NMR (150 MHz, $CDCl_3$)

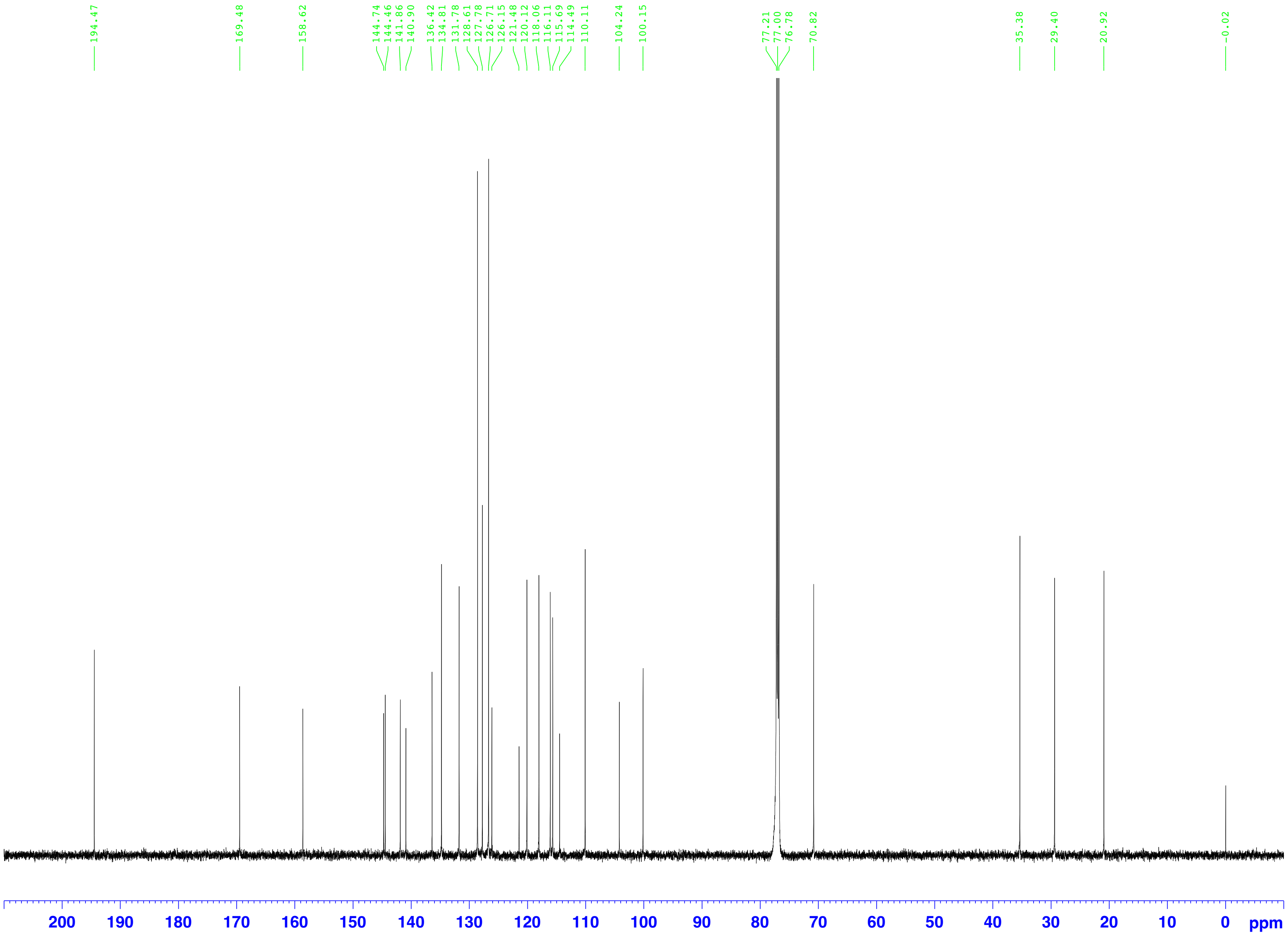


```
Current Data Parameters
NAME          DO1-59-ptlc
EXPNO                  33
PROCNO                  1

F2 - Acquisition Parameters
Date_            20210205
Time                 3.00
INSTRUM             spect
PROBHD    5 mm CPPBBO BB
PULPROG            zgpg30
TD                  65536
SOLVENT             CDCl3
NS                   1024
DS                      4
SWH             36057.691 Hz
FIDRES           0.550197 Hz
AQ              0.9087659 sec
RG                 175.56
DW                 13.867 usec
DE                  18.00 usec
TE                  298.2 K
D1             2.00000000 sec
D11            0.03000000 sec
TD0                     1

======== CHANNEL f1 ========
SFO1          150.9178981 MHz
NUC1                  13C
P1                  10.00 usec
PLW1          80.00000000 W

======== CHANNEL f2 ========
SFO2          600.1324005 MHz
NUC2                   1H
CPDPRG[2          waltz16
PCPD2               70.00 usec
PLW2          13.43999958 W
PLW12          0.61714000 W
PLW13          0.31042001 W

F2 - Processing parameters
SI                  32768
SF            150.9028140 MHz
WDW                    EM
SSB                     0
LB                   1.00 Hz
GB                      0
PC                   1.40
```

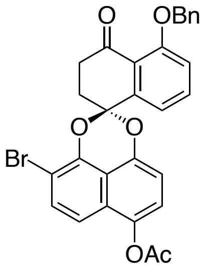

(*S*)-**22**

^{1}H NMR (600 MHz, $CDCl_3$)

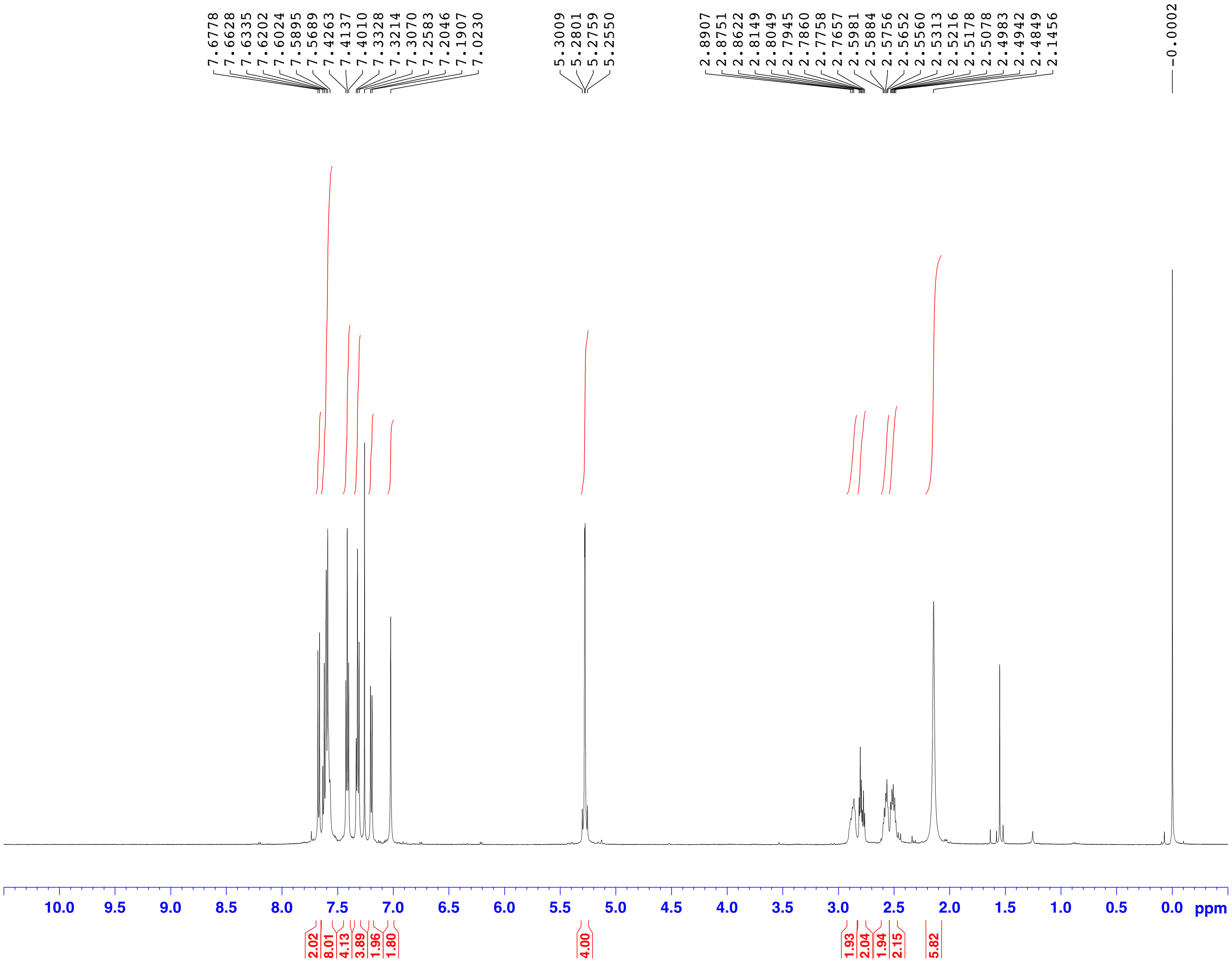


```
Current Data Parameters
NAME      DO1-59-ptlc-bp
EXPNO                 10
PROCNO                 1

F2 - Acquisition Parameters
Date_           20210204
Time               19.20
INSTRUM            spect
PROBHD   5 mm CPPBBO BB
PULPROG             zg30
TD                 65536
SOLVENT            CDCl3
NS                    16
DS                     2
SWH            12019.230 Hz
FIDRES          0.183399 Hz
AQ             2.7262976 sec
RG                 31.94
DW                41.600 usec
DE                 10.00 usec
TE                 298.2 K
D1            1.00000000 sec
TD0                    1

======== CHANNEL f1 ========
SFO1         600.1337060 MHz
NUC1                  1H
P1                 12.00 usec
PLW1         21.00000000 W

F2 - Processing parameters
SI                 65536
SF           600.1300160 MHz
WDW                   EM
SSB                    0
LB                  0.30 Hz
GB                     0
PC                  1.00
```

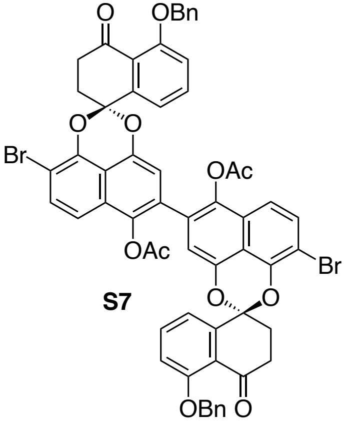

S7

^{13}C NMR (150 MHz, $CDCl_3$)

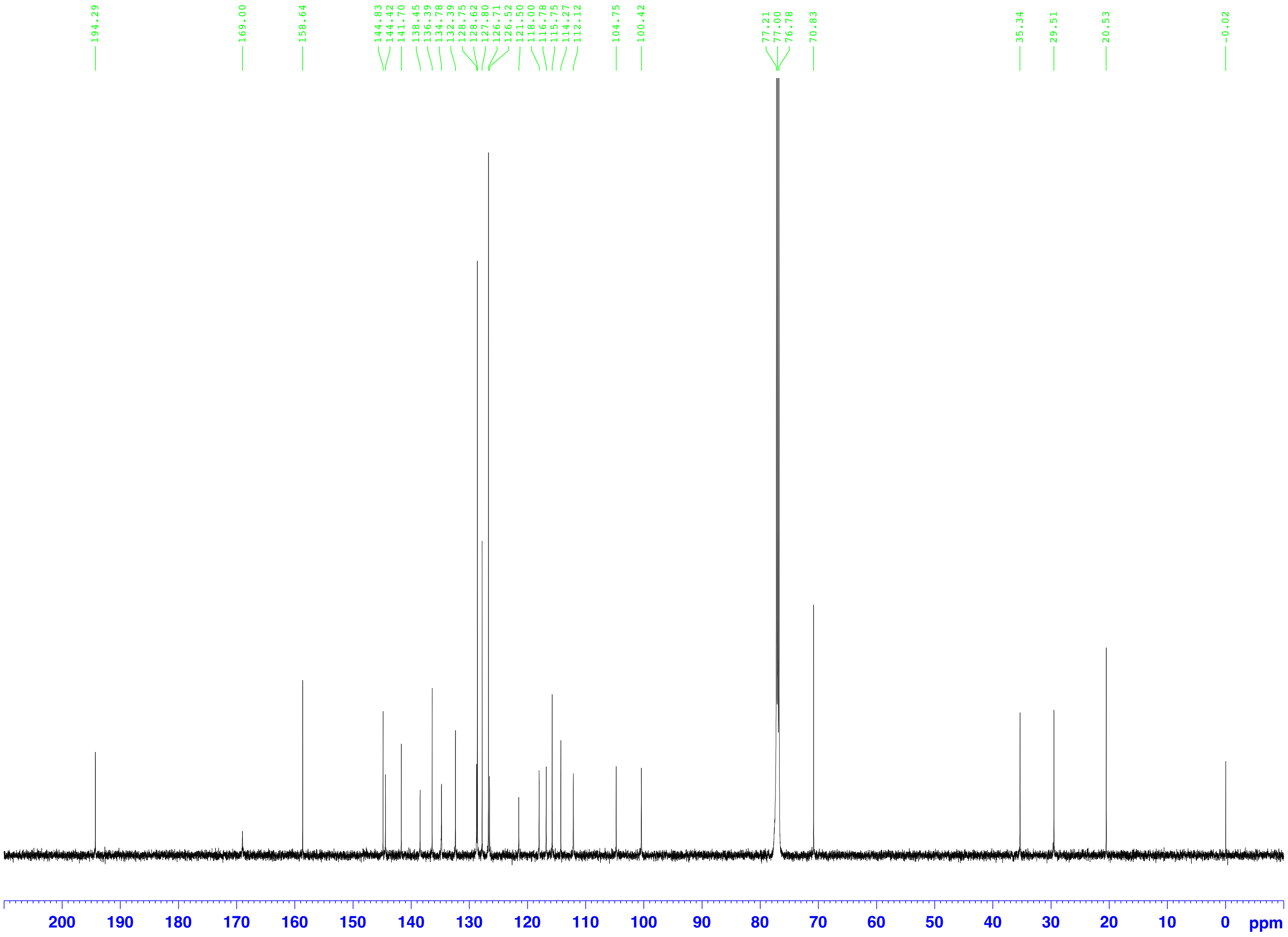


```
Current Data Parameters
NAME      DO1-59-ptlc-bp
EXPNO                 13
PROCNO                 1

F2 - Acquisition Parameters
Date_           20210205
Time                5.51
INSTRUM            spect
PROBHD    5 mm CPPBBO BB
PULPROG          zgpg30
TD                 65536
SOLVENT            CDCl3
NS                  1024
DS                     4
SWH           36057.691 Hz
FIDRES         0.550197 Hz
AQ            0.9087659 sec
RG               175.56
DW               13.867 usec
DE                18.00 usec
TE                298.2 K
D1           2.00000000 sec
D11          0.03000000 sec
TD0                    1

======== CHANNEL f1 ========
SFO1        150.9178981 MHz
NUC1                 13C
P1                 10.00 usec
PLW1         80.00000000 W

======== CHANNEL f2 ========
SFO2        600.1324005 MHz
NUC2                  1H
CPDPRG[2         waltz16
PCPD2              70.00 usec
PLW2         13.43999958 W
PLW12         0.61714000 W
PLW13         0.31042001 W

F2 - Processing parameters
SI                 32768
SF          150.9028136 MHz
WDW                   EM
SSB                    0
LB                  1.00 Hz
GB                     0
PC                  1.40
```

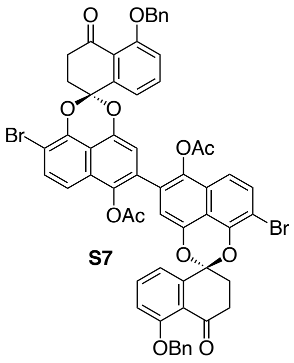

S7

HSQC

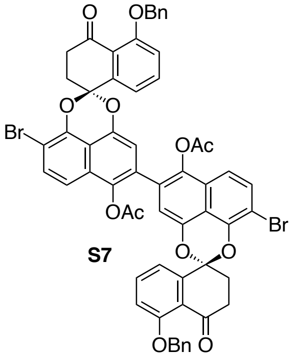

S7

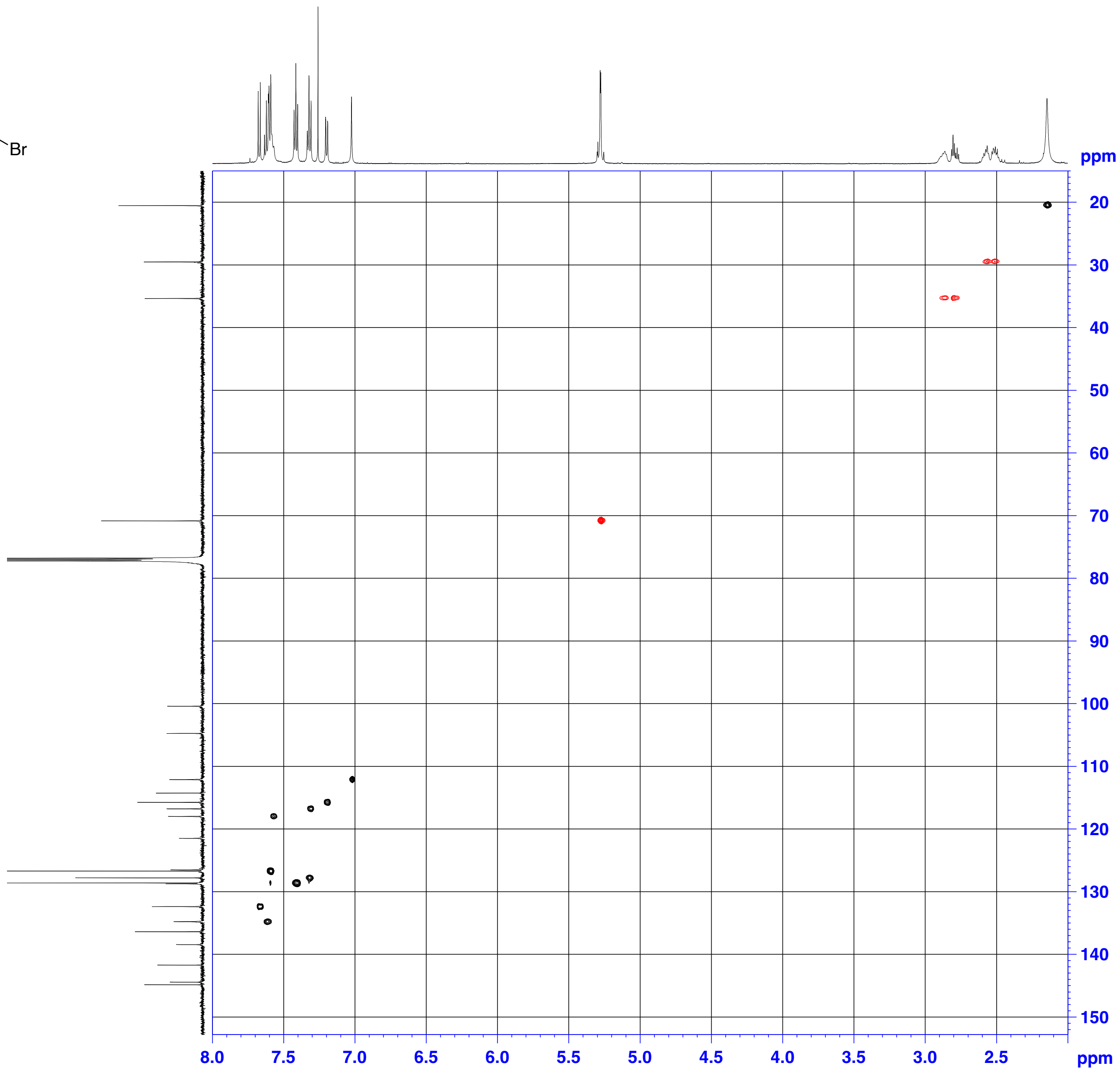


```
Current Data Parameters
NAME      DO1-59-ptlc-bp
EXPNO                 15
PROCNO                 1

F2 - Acquisition Parameters
Date_           20210205
Time                6.08
INSTRUM            spect
PROBHD   5 mm CPPBBO BB
PULPROG  hsqcedetgpsisp2.3
TD                  2048
SOLVENT            CDCl3
NS                     4
DS                    32
SWH             7211.539 Hz
FIDRES          3.521259 Hz
AQ             0.1419947 sec
RG                175.56
DW                69.333 usec
DE                 10.00 usec
TE                 298.2 K
CNST2        145.0000000
CNST17        -0.5000000
D0            0.00000300 sec
D1            2.00000000 sec
D4            0.00172414 sec
D11           0.03000000 sec
D16           0.00020000 sec
D21           0.00360000 sec
D24           0.00089000 sec
IN0           0.00002000 sec

======== CHANNEL f1 ========
SFO1         600.1328224 MHz
NUC1                  1H
P1                 12.00 usec
P2                 24.00 usec
P28                    0 usec
PLW1         21.00000000 W

======== CHANNEL f2 ========
SFO2         150.9133710 MHz
NUC2                 13C
CPDPRG[2            garp
P3                 10.00 usec
P14               500.00 usec
P24              2000.00 usec
P31              1730.00 usec
PCPD2              60.00 usec
PLW0                   0 W
PLW2         80.00000000 W
PLW12         2.22219992 W
SPNAM[3] Crp60,0.5,20.1
SPOAL3             0.500
SPOFFS3                0 Hz
SPW3         12.22299957 W
SPNAM[7]    Crp60comp.4
SPOAL7             0.500
SPOFFS7                0 Hz
SPW7         12.22299957 W
SPNAM[18  Crp60_xfilt.2
SPOAL18            0.500
SPOFFS18               0 Hz
SPW18         3.53270006 W

====== GRADIENT CHANNEL =====
GPNAM[1]     SMSQ10.100
GPNAM[2]     SMSQ10.100
GPNAM[3]     SMSQ10.100
GPNAM[4]     SMSQ10.100
GPZ1               80.00 %
GPZ2               20.10 %
GPZ3               11.00 %
GPZ4               -5.00 %
P16              1000.00 usec
P19               600.00 usec

F1 - Acquisition parameters
TD                   256
SFO1            150.9134 MHz
FIDRES        195.312500 Hz
SW               165.658 ppm
FnMODE     Echo-Antiecho

F2 - Processing parameters
SI                  1024
SF           600.1300161 MHz
WDW                QSINE
SSB                    2
LB                     0 Hz
GB                     0
PC                  1.40

F1 - Processing parameters
SI                  1024
MC2       echo-antiecho
SF           150.9028135 MHz
WDW                QSINE
SSB                    2
LB                     0 Hz
GB                     0
```

HMBC

key HMBC correlations

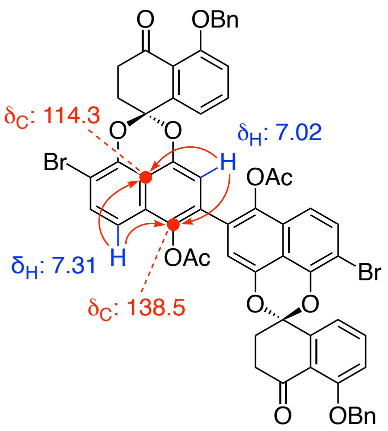

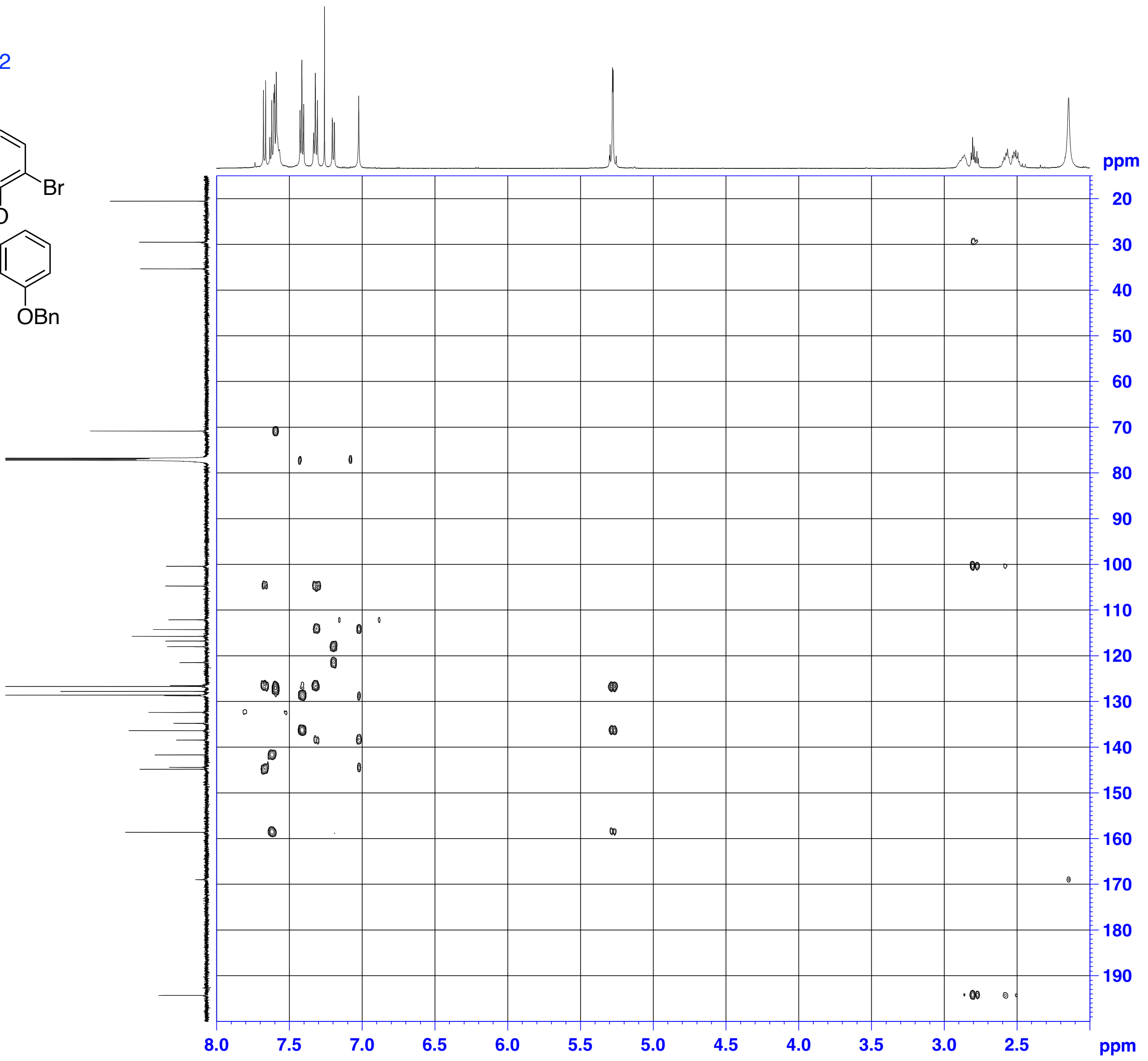

^{1}H NMR (600 MHz, $CDCl_3$)

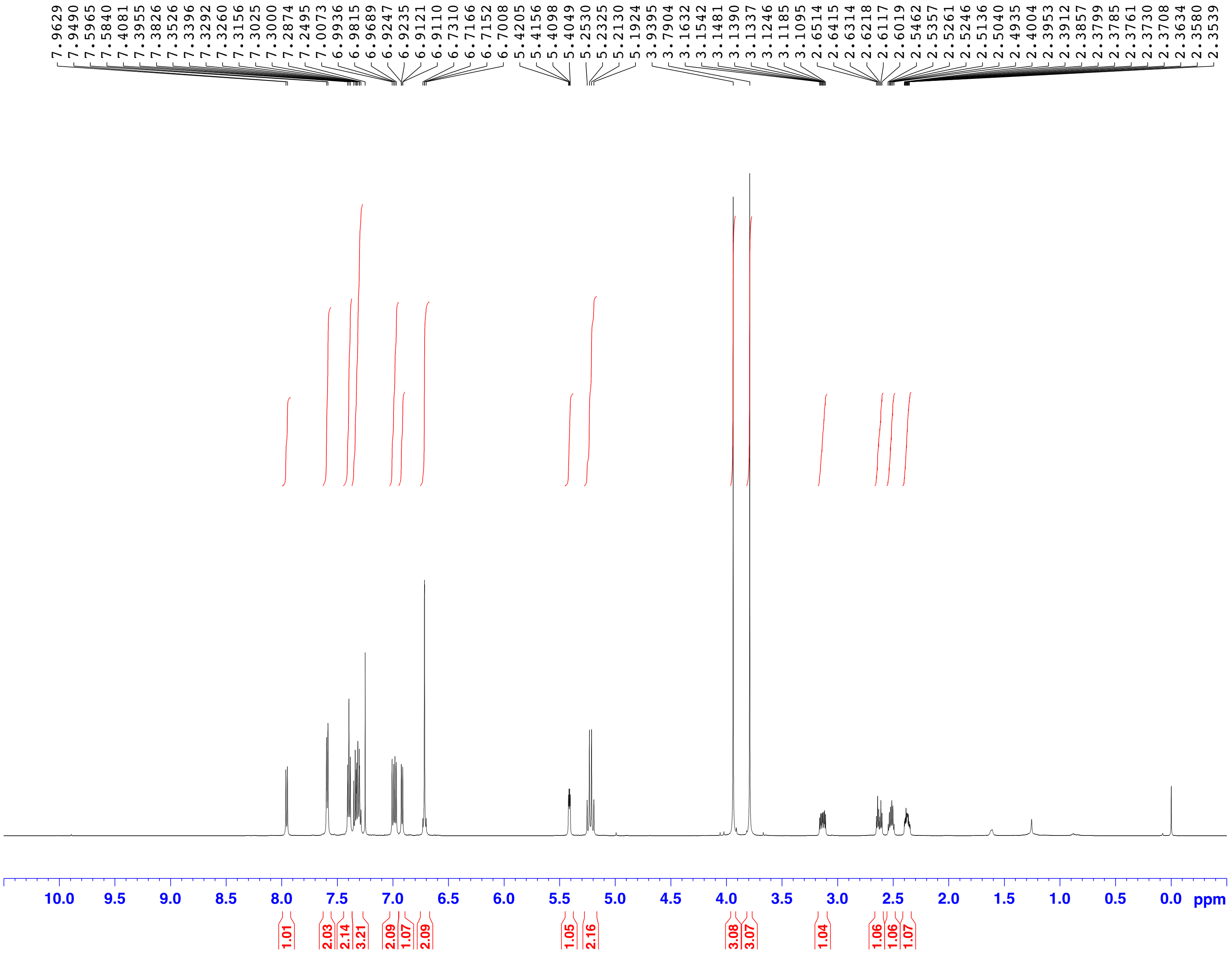

BRUKER

```
Current Data Parameters
NAME        DO1-57-column2-1
EXPNO                     20
PROCNO                     1

F2 - Acquisition Parameters
Date_               20210128
Time                   18.38
INSTRUM                spect
PROBHD     5 mm CPPBBO BB
PULPROG                 zg30
TD                     65536
SOLVENT                CDCl3
NS                        16
DS                         2
SWH                12019.230 Hz
FIDRES              0.183399 Hz
AQ                 2.7262976 sec
RG                      17.5
DW                    41.600 usec
DE                     10.00 usec
TE                     298.2 K
D1                1.00000000 sec
TD0                        1

======== CHANNEL f1 ========
SFO1             600.1337060 MHz
NUC1                      1H
P1                     12.00 usec
PLW1             21.00000000 W

F2 - Processing parameters
SI                     65536
SF               600.1300213 MHz
WDW                       EM
SSB                        0
LB                      0.30 Hz
GB                         0
PC                      1.00
```

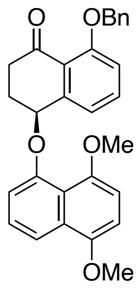

S8

^{13}C NMR (150 MHz, $CDCl_3$)

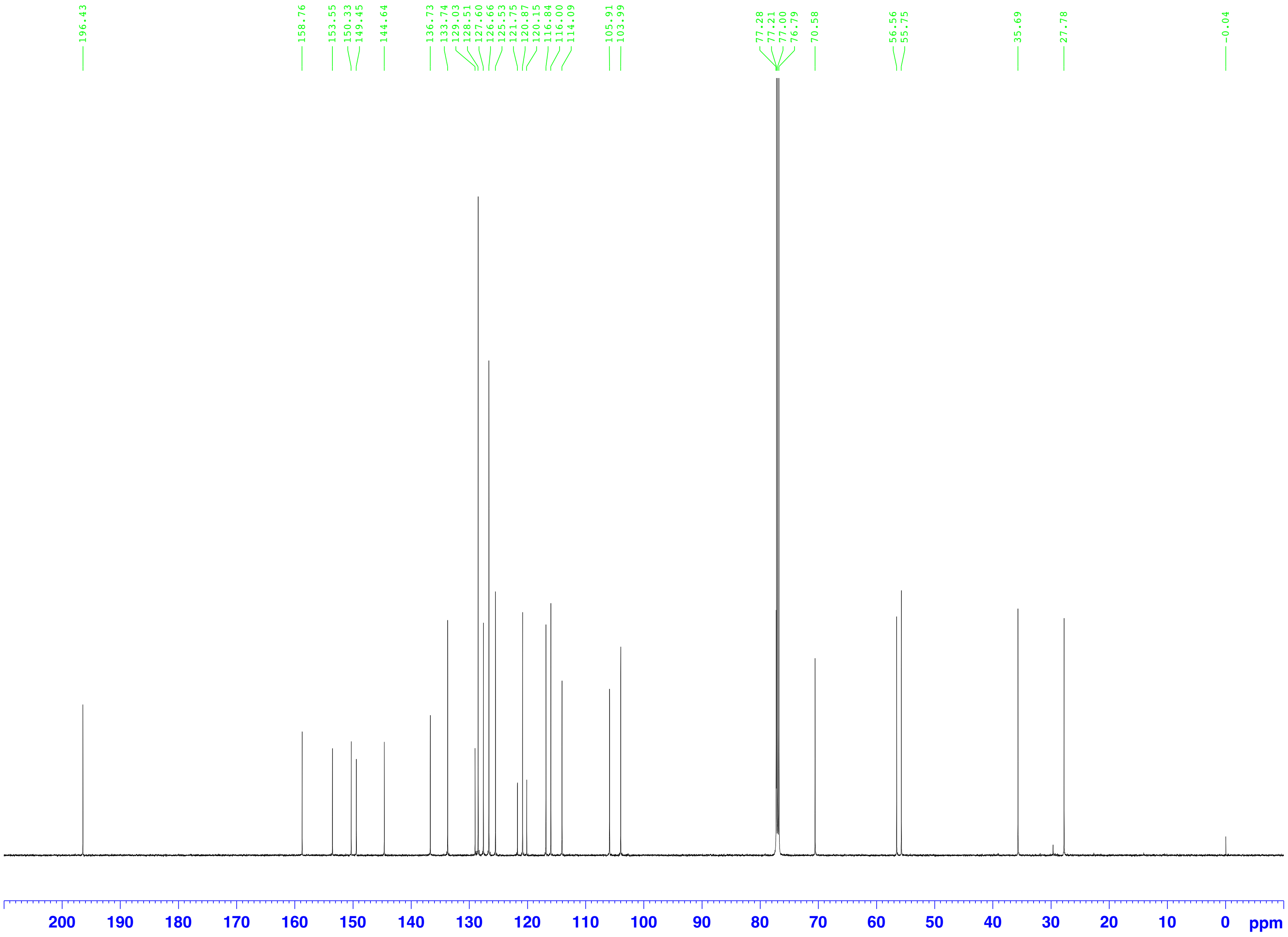


```
Current Data Parameters
NAME      DO1-57-column2-1
EXPNO                   23
PROCNO                   1

F2 - Acquisition Parameters
Date_             20210129
Time                  3.02
INSTRUM              spect
PROBHD     5 mm CPPBBO BB
PULPROG            zgpg30
TD                   65536
SOLVENT              CDCl3
NS                    1024
DS                       4
SWH              36057.691 Hz
FIDRES            0.550197 Hz
AQ               0.9087659 sec
RG                  175.56
DW                  13.867 usec
DE                   18.00 usec
TE                   298.2 K
D1              2.00000000 sec
D11             0.03000000 sec
TD0                      1

======== CHANNEL f1 ========
SFO1          150.9178981 MHz
NUC1                   13C
P1                   10.00 usec
PLW1           80.00000000 W

======== CHANNEL f2 ========
SFO2          600.1324005 MHz
NUC2                    1H
CPDPRG[2           waltz16
PCPD2                70.00 usec
PLW2           13.43999958 W
PLW12           0.61714000 W
PLW13           0.31042001 W

F2 - Processing parameters
SI                   32768
SF            150.9028186 MHz
WDW                     EM
SSB                      0
LB                    1.00 Hz
GB                       0
PC                    1.40
```

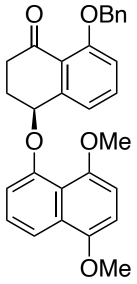

S8

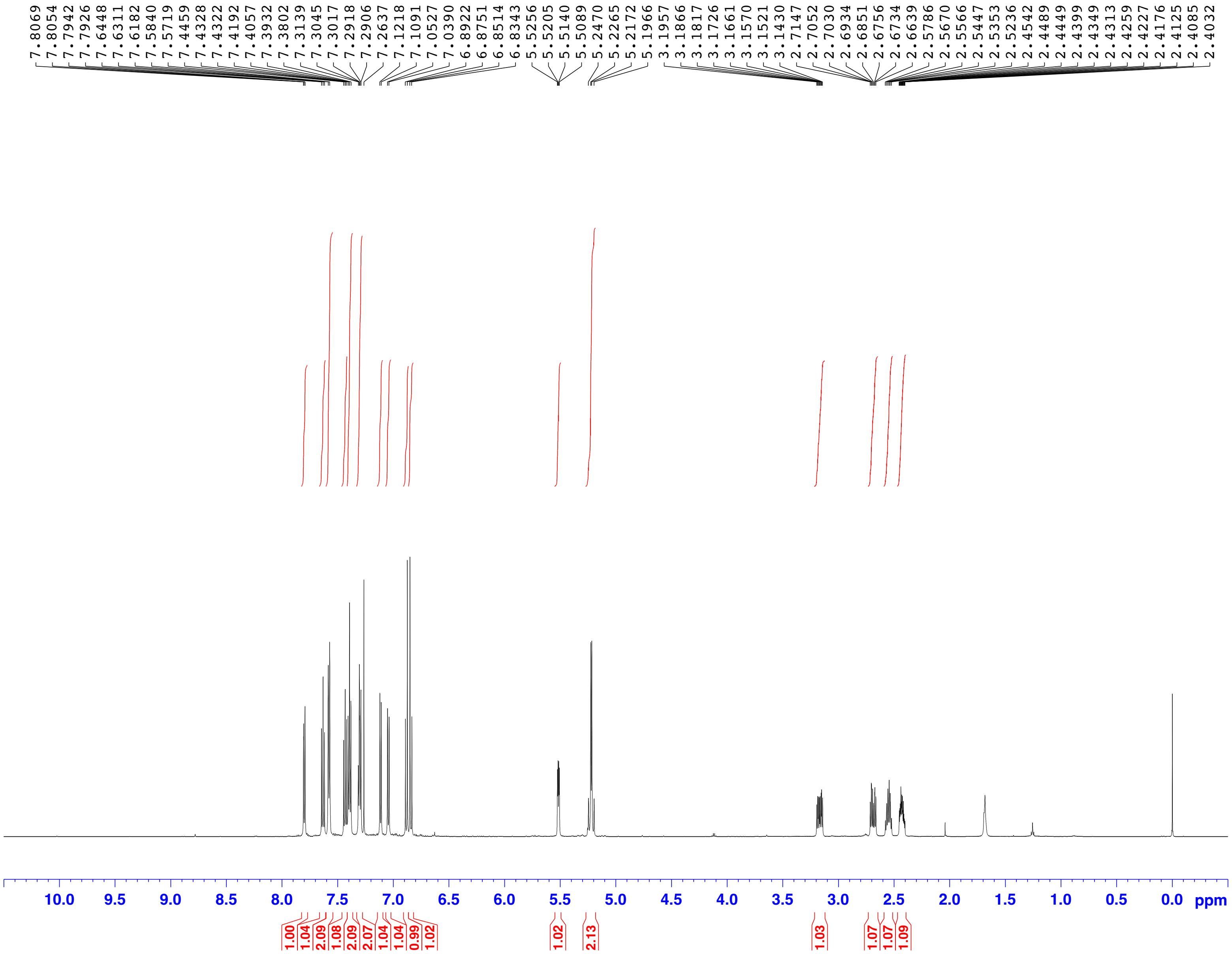

Current Data Parameters
NAME DO1-361-ptlc
EXPNO 10
PROCNO 1

F2 - Acquisition Parameters
Date_ 20220527
Time 19.35
INSTRUM spect
PROBHD 5 mm CPPBBO BB
PULPROG zg30
TD 65536
SOLVENT CDCl3
NS 16
DS 2
SWH 12019.230 Hz
FIDRES 0.183399 Hz
AQ 2.7262976 sec
RG 17.5
DW 41.600 usec
DE 10.00 usec
TE 298.1 K
D1 1.00000000 sec
TD0 1

======== CHANNEL f1 ========
SFO1 600.1337060 MHz
NUC1 1H
P1 12.00 usec
PLW1 21.00000000 W

F2 - Processing parameters
SI 65536
SF 600.1300120 MHz
WDW EM
SSB 0
LB 0.30 Hz
GB 0
PC 1.00

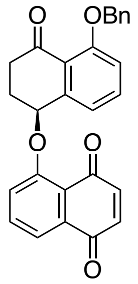

S9

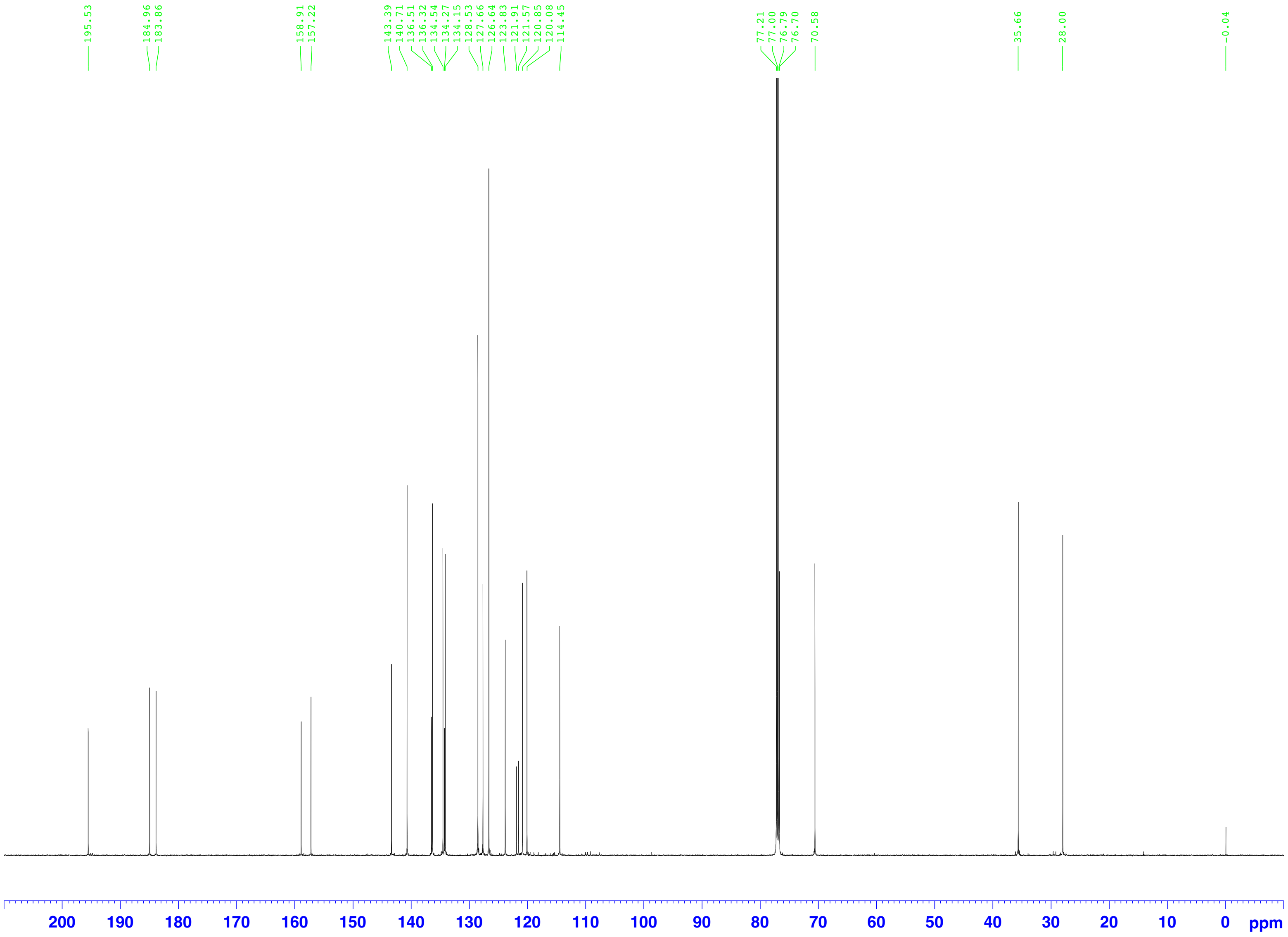


```
Current Data Parameters
NAME          DO1-361-ptlc
EXPNO                   11
PROCNO                   1

F2 - Acquisition Parameters
Date_             20220528
Time                  1.27
INSTRUM              spect
PROBHD    5 mm CPPBBO BB
PULPROG            zgpg30
TD                  65536
SOLVENT             CDCl3
NS                   4096
DS                      4
SWH             36057.691 Hz
FIDRES           0.550197 Hz
AQ              0.9087659 sec
RG                 175.56
DW                 13.867 usec
DE                  18.00 usec
TE                  298.2 K
D1             2.00000000 sec
D11            0.03000000 sec
TD0                     1

======== CHANNEL f1 ========
SFO1          150.9178981 MHz
NUC1                  13C
P1                  10.00 usec
PLW1          80.00000000 W

======== CHANNEL f2 ========
SFO2          600.1324005 MHz
NUC2                   1H
CPDPRG[2           waltz16
PCPD2               70.00 usec
PLW2          13.43999958 W
PLW12          0.61714000 W
PLW13          0.31042001 W

F2 - Processing parameters
SI                  32768
SF            150.9028179 MHz
WDW                    EM
SSB                     0
LB                   1.00 Hz
GB                      0
PC                   1.40
```

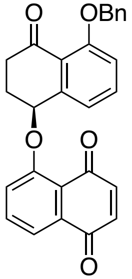

S9

^{1}H NMR (600 MHz, $CDCl_3$)

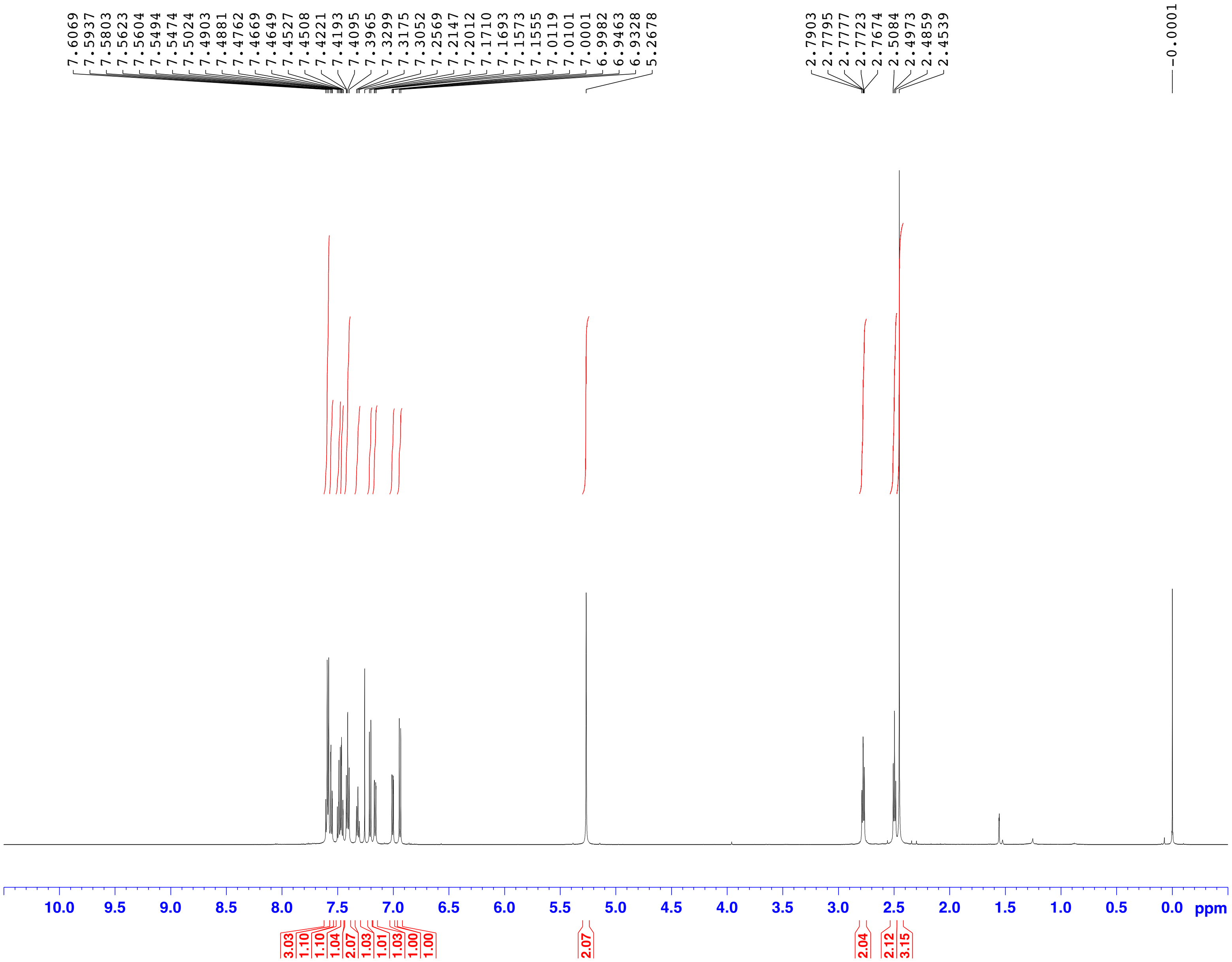


```
Current Data Parameters
NAME          DO1-61-ptlc-1
EXPNO                    20
PROCNO                    1

F2 - Acquisition Parameters
Date_              20210204
Time                  19.24
INSTRUM               spect
PROBHD   5 mm CPPBBO BB
PULPROG                zg30
TD                    65536
SOLVENT               CDCl3
NS                       16
DS                        2
SWH               12019.230 Hz
FIDRES             0.183399 Hz
AQ                2.7262976 sec
RG                    31.94
DW                   41.600 usec
DE                    10.00 usec
TE                    298.2 K
D1               1.00000000 sec
TD0                       1

======== CHANNEL f1 ========
SFO1            600.1337060 MHz
NUC1                     1H
P1                    12.00 usec
PLW1            21.00000000 W

F2 - Processing parameters
SI                    65536
SF              600.1300171 MHz
WDW                      EM
SSB                       0
LB                     0.30 Hz
GB                        0
PC                     1.00
```

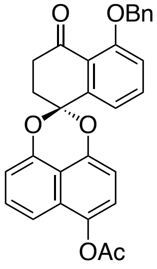

S11

^{13}C NMR (150 MHz, $CDCl_3$)

C13CPD CDCl3 {Z:\data} ogawa 59

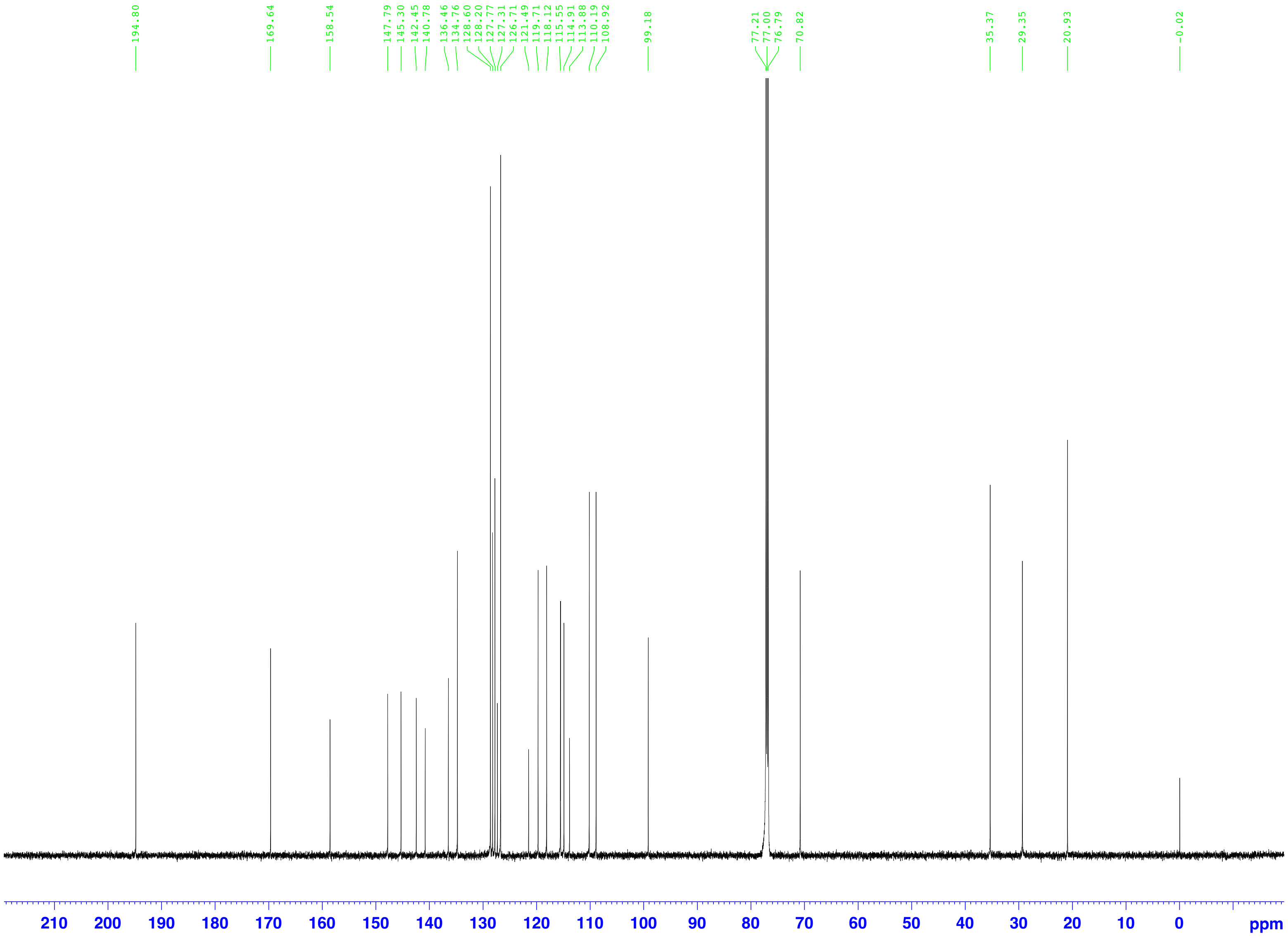


```
Current Data Parameters
NAME        DO1-61-ptlc-1
EXPNO                  23
PROCNO                  1

F2 - Acquisition Parameters
Date_            20210205
Time                21.31
INSTRUM             spect
PROBHD   5 mm CPPBBO BB
PULPROG           zgpg30
TD                 65536
SOLVENT            CDCl3
NS                  1024
DS                     4
SWH            36057.691 Hz
FIDRES          0.550197 Hz
AQ             0.9087659 sec
RG                175.56
DW                13.867 usec
DE                 18.00 usec
TE                 298.2 K
D1            2.00000000 sec
D11           0.03000000 sec
TD0                    1

======== CHANNEL f1 ========
SFO1         150.9178981 MHz
NUC1                 13C
P1                 10.00 usec
PLW1         80.00000000 W

======== CHANNEL f2 ========
SFO2         600.1324005 MHz
NUC2                  1H
CPDPRG[2          waltz16
PCPD2              70.00 usec
PLW2         13.43999958 W
PLW12         0.61714000 W
PLW13         0.31042001 W

F2 - Processing parameters
SI                 32768
SF           150.9028142 MHz
WDW                   EM
SSB                    0
LB                  1.00 Hz
GB                     0
PC                  1.40
```

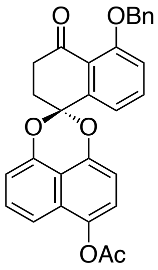

S11

^{1}H NMR (600 MHz, $CDCl_3$)

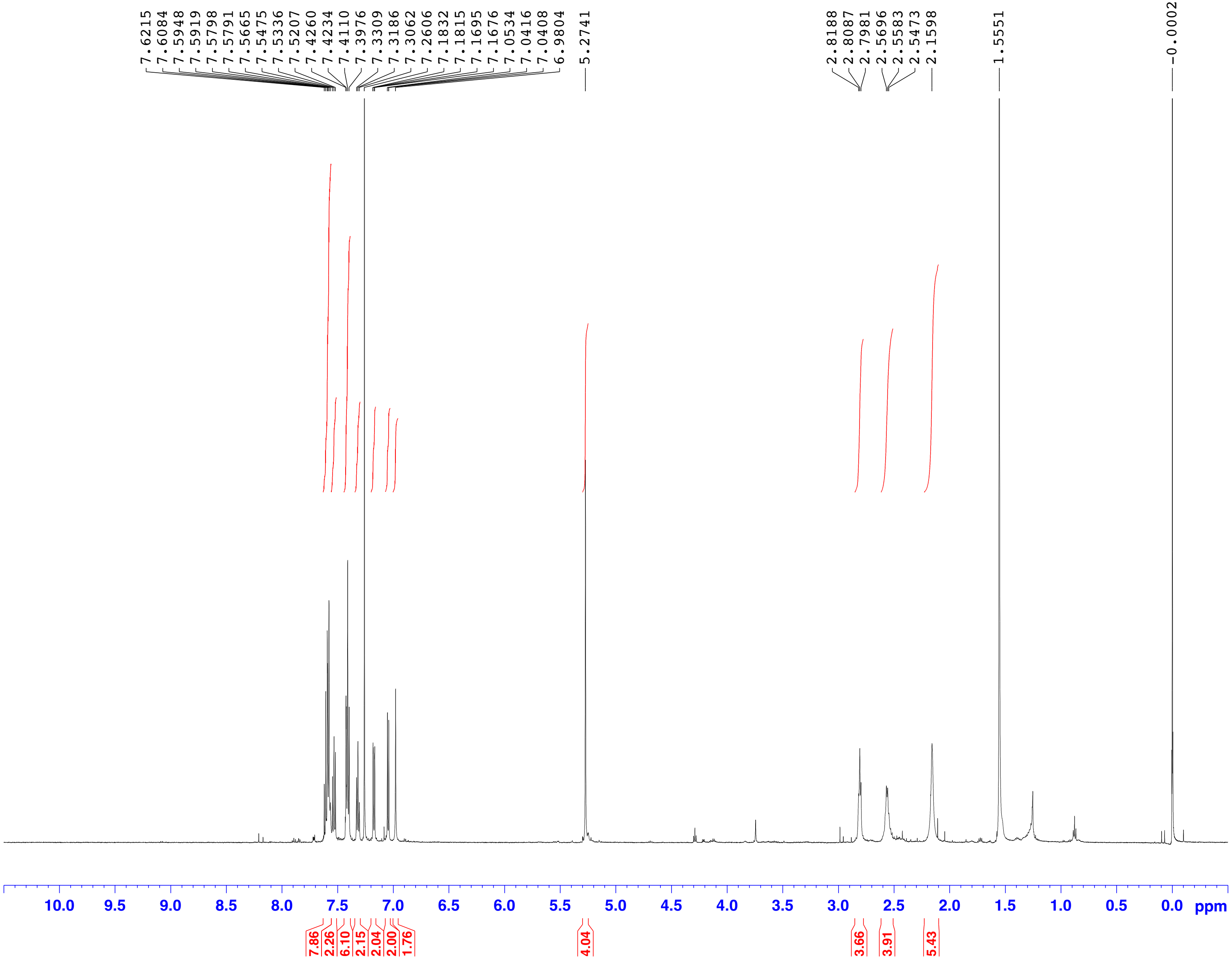


```
Current Data Parameters
NAME          DO1-362-ptlc
EXPNO                   40
PROCNO                   1

F2 - Acquisition Parameters
Date_             20220607
Time                 17.52
INSTRUM              spect
PROBHD    5 mm CPPBBO BB
PULPROG               zg30
TD                   65536
SOLVENT              CDCl3
NS                      16
DS                       2
SWH             12019.230 Hz
FIDRES           0.183399 Hz
AQ              2.7262976 sec
RG                   31.94
DW                  41.600 usec
DE                   10.00 usec
TE                   298.0 K
D1             1.00000000 sec
TD0                      1

======== CHANNEL f1 ========
SFO1         600.1337060 MHz
NUC1                    1H
P1                   12.00 usec
PLW1          21.00000000 W

F2 - Processing parameters
SI                   65536
SF           600.1300139 MHz
WDW                     EM
SSB                      0
LB                    0.30 Hz
GB                       0
PC                    1.00
```

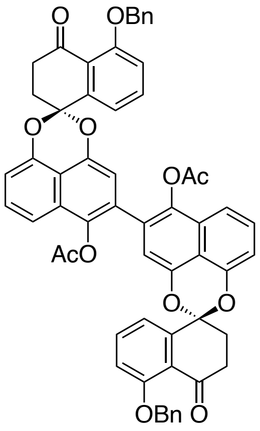

S12

^{13}C NMR (150 MHz, $CDCl_3$)

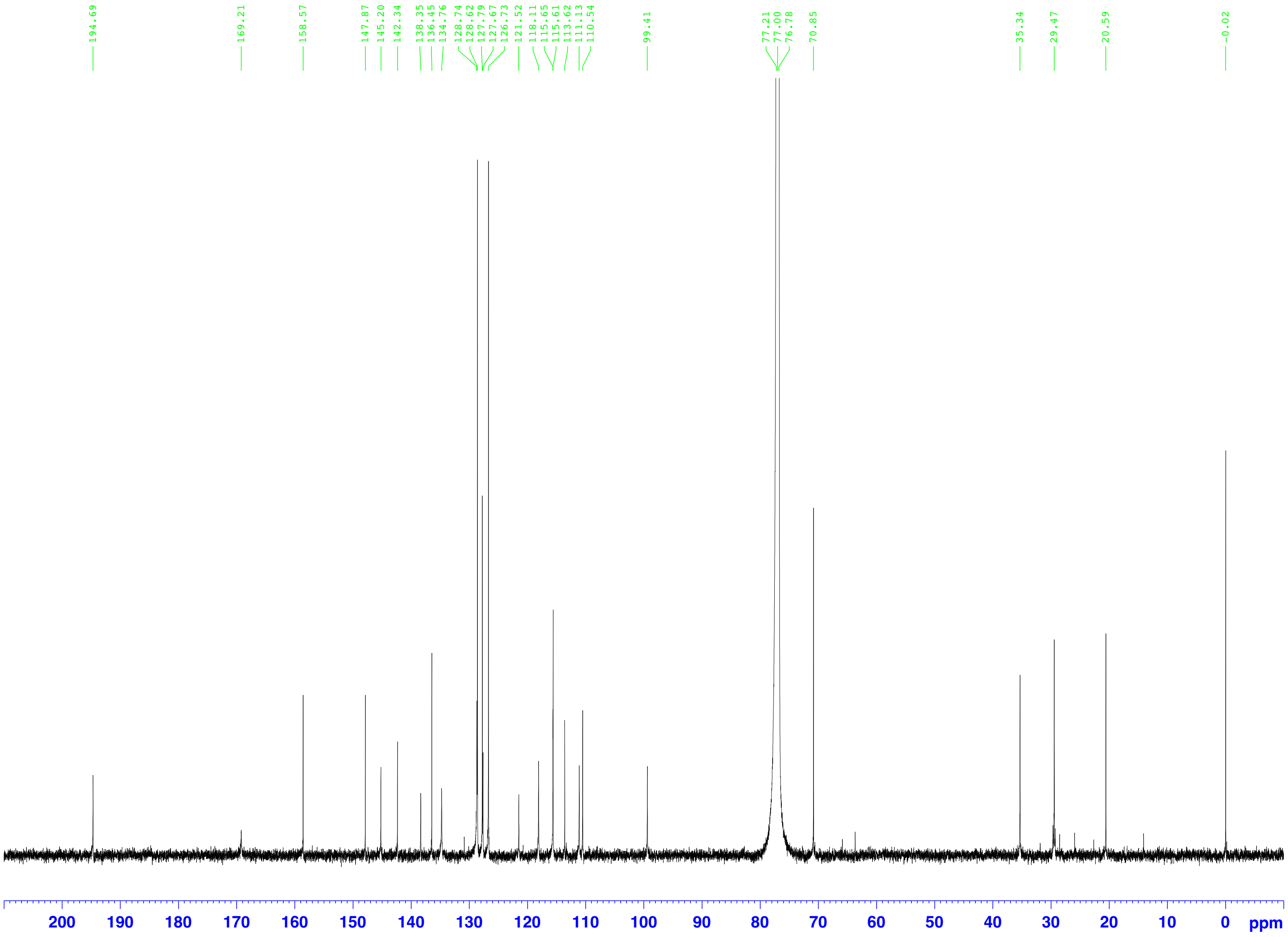


```
Current Data Parameters
NAME          DO1-362-ptlc
EXPNO                   80
PROCNO                   1

F2 - Acquisition Parameters
Date_             20220613
Time                  1.06
INSTRUM              spect
PROBHD    5 mm CPPBBO BB
PULPROG            zgpg30
TD                   65536
SOLVENT              CDCl3
NS                   32768
DS                       4
SWH             36057.691 Hz
FIDRES           0.550197 Hz
AQ              0.9087659 sec
RG                  175.56
DW                  13.867 usec
DE                   18.00 usec
TE                   298.0 K
D1              2.00000000 sec
D11             0.03000000 sec
TD0                      1

======== CHANNEL f1 ========
SFO1        150.9178981 MHz
NUC1                   13C
P1                   10.00 usec
PLW1          80.00000000 W

======== CHANNEL f2 ========
SFO2        600.1324005 MHz
NUC2                    1H
CPDPRG[2           waltz16
PCPD2                70.00 usec
PLW2          13.43999958 W
PLW12          0.61714000 W
PLW13          0.31042001 W

F2 - Processing parameters
SI                   32768
SF          150.9028118 MHz
WDW                     EM
SSB                      0
LB                    1.00 Hz
GB                       0
PC                    1.40
```

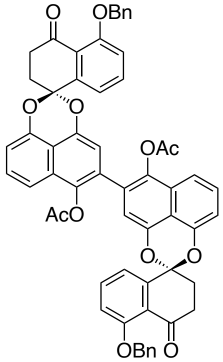

S12

¹H NMR (600 MHz, $CDCl_3$)

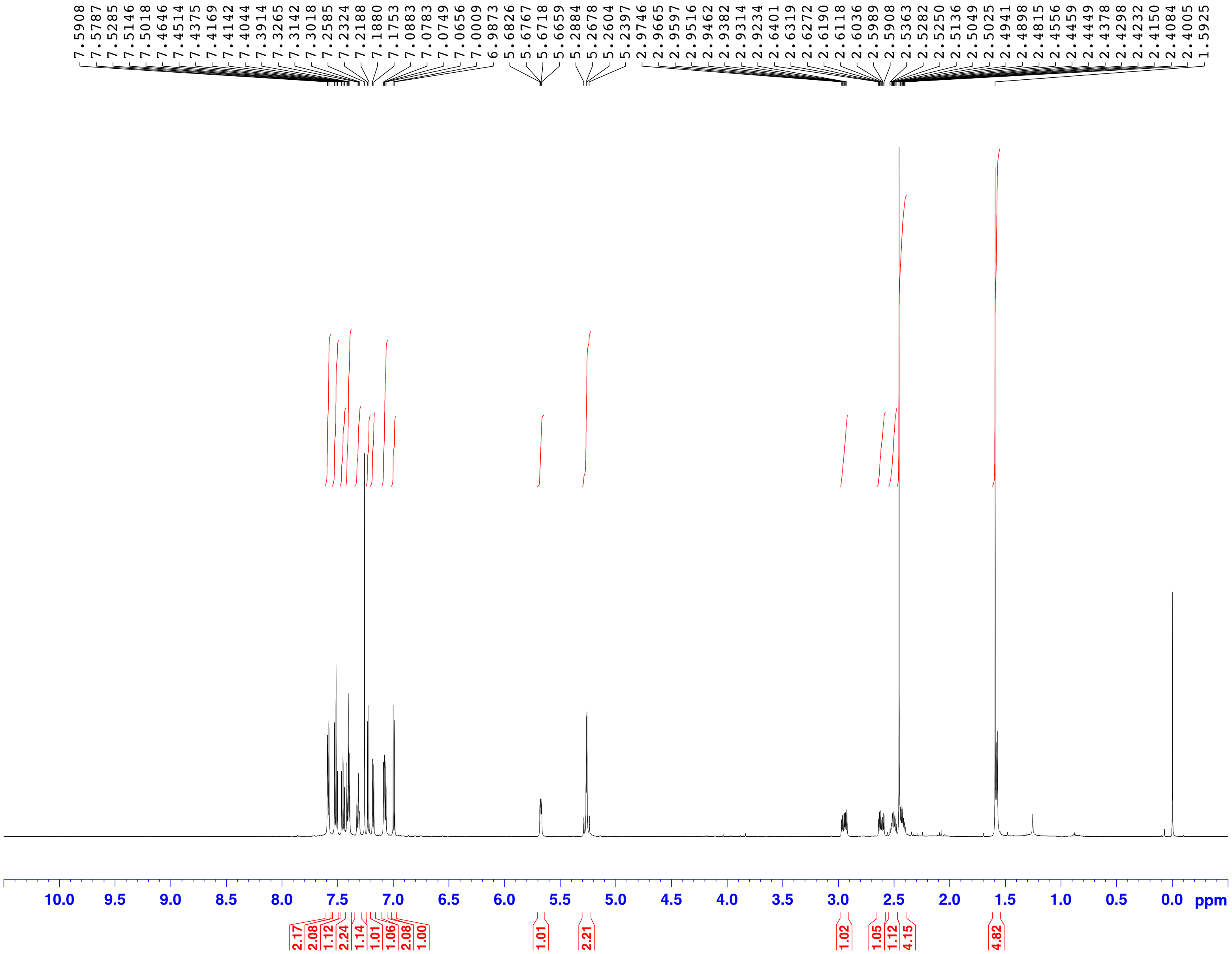

BRUKER

```
Current Data Parameters
NAME           DO1-362-ptlc
EXPNO                    60
PROCNO                    1

F2 - Acquisition Parameters
Date_              20220607
Time                  20.19
INSTRUM               spect
PROBHD    5 mm CPPBBO BB
PULPROG                zg30
TD                    65536
SOLVENT               CDCl3
NS                       16
DS                        2
SWH               12019.230 Hz
FIDRES             0.183399 Hz
AQ                2.7262976 sec
RG                    31.94
DW                   41.600 usec
DE                    10.00 usec
TE                    298.0 K
D1               1.00000000 sec
TD0                       1

======== CHANNEL f1 ========
SFO1            600.1337060 MHz
NUC1                     1H
P1                    12.00 usec
PLW1            21.00000000 W

F2 - Processing parameters
SI                    65536
SF              600.1300152 MHz
WDW                      EM
SSB                       0
LB                     0.30 Hz
GB                        0
PC                     1.00
```

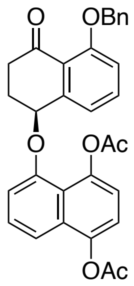

S13

^{13}C NMR (150 MHz, $CDCl_3$)

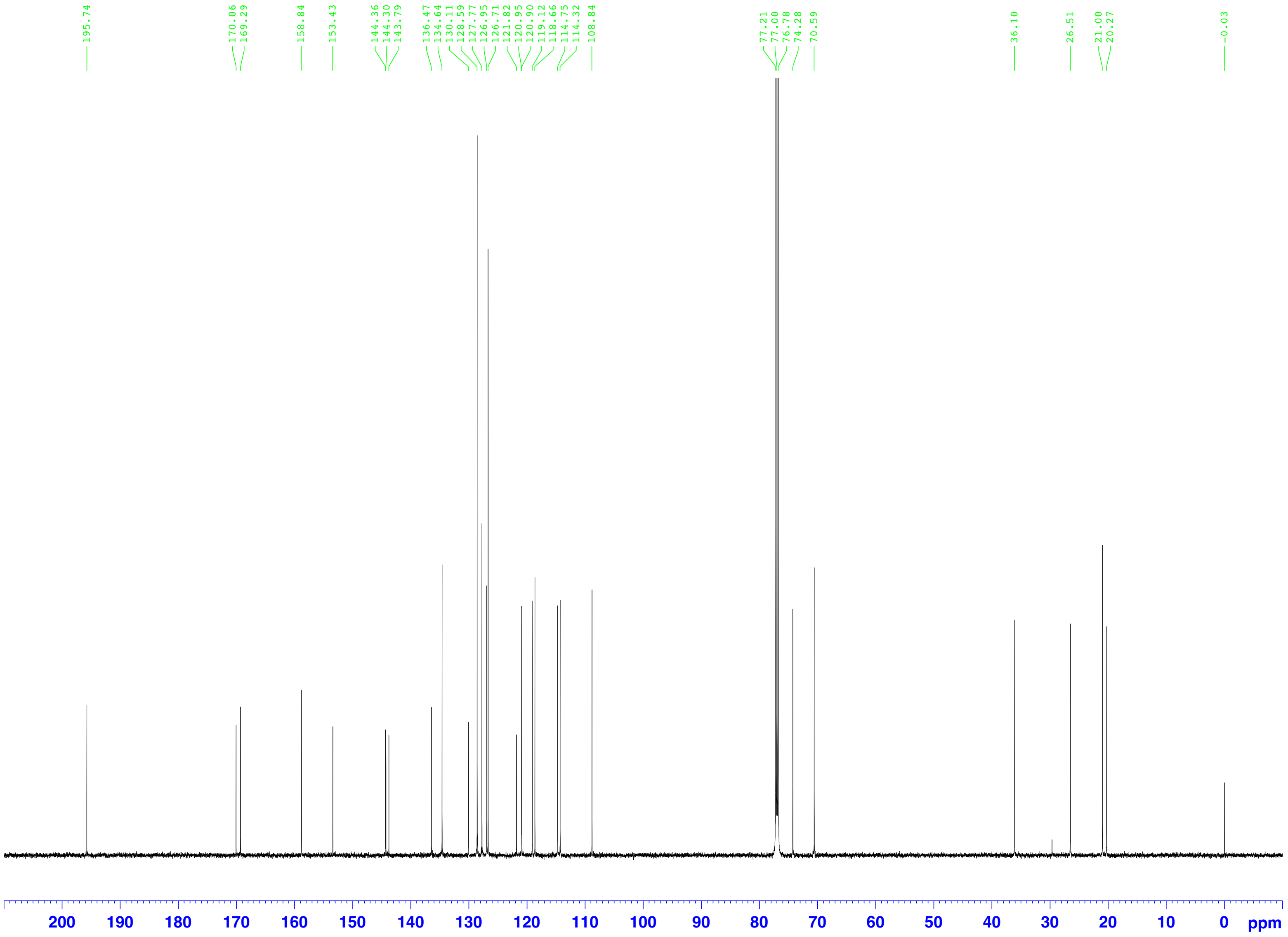


```
Current Data Parameters
NAME          DO1-362-ptlc
EXPNO                   61
PROCNO                   1

F2 - Acquisition Parameters
Date_             20220608
Time                  7.05
INSTRUM              spect
PROBHD     5 mm CPPBBO BB
PULPROG            zgpg30
TD                   65536
SOLVENT              CDCl3
NS                    2048
DS                       4
SWH             36057.691 Hz
FIDRES           0.550197 Hz
AQ              0.9087659 sec
RG                  175.56
DW                  13.867 usec
DE                   18.00 usec
TE                   297.9 K
D1             2.00000000 sec
D11            0.03000000 sec
TD0                      1

======== CHANNEL f1 ========
SFO1          150.9178981 MHz
NUC1                   13C
P1                   10.00 usec
PLW1           80.00000000 W

======== CHANNEL f2 ========
SFO2          600.1324005 MHz
NUC2                    1H
CPDPRG[2           waltz16
PCPD2                70.00 usec
PLW2           13.43999958 W
PLW12           0.61714000 W
PLW13           0.31042001 W

F2 - Processing parameters
SI                   32768
SF            150.9028142 MHz
WDW                     EM
SSB                      0
LB                    1.00 Hz
GB                       0
PC                    1.40
```

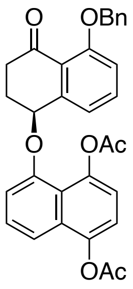

S13

^{1}H NMR (600 MHz, $CDCl_3$)

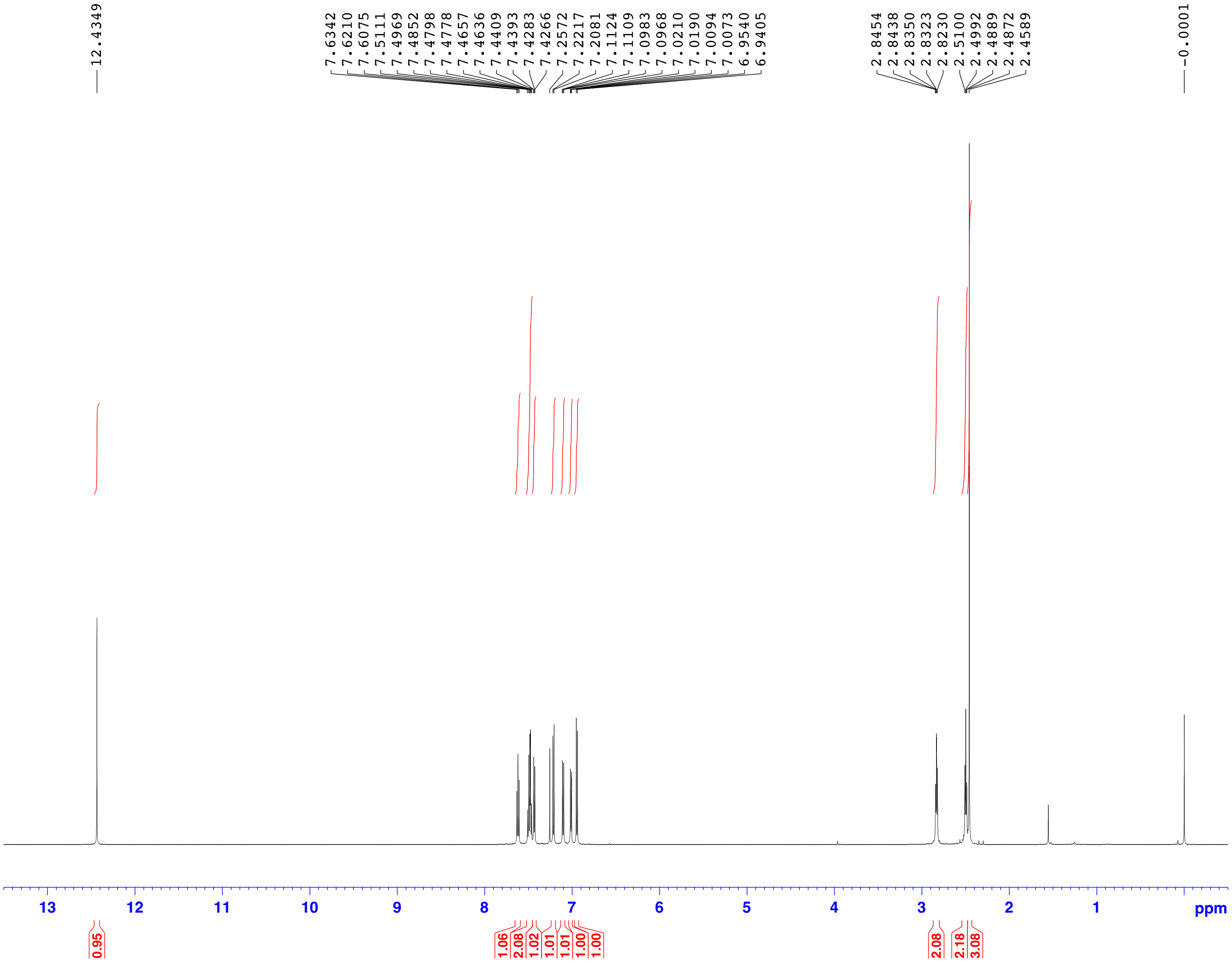


```
Current Data Parameters
NAME        DO1-62-ptlc-2
EXPNO                  10
PROCNO                  1

F2 - Acquisition Parameters
Date_           20210204
Time               13.26
INSTRUM            spect
PROBHD   5 mm CPPBBO BB
PULPROG             zg30
TD                 65536
SOLVENT            CDCl3
NS                    16
DS                     2
SWH            12019.230 Hz
FIDRES          0.183399 Hz
AQ             2.7262976 sec
RG                 31.94
DW                41.600 usec
DE                 10.00 usec
TE                 298.1 K
D1            1.00000000 sec
TD0                    1

======== CHANNEL f1 ========
SFO1         600.1337060 MHz
NUC1                  1H
P1                 12.00 usec
PLW1         21.00000000 W

F2 - Processing parameters
SI                 65536
SF           600.1300166 MHz
WDW                   EM
SSB                    0
LB                  0.30 Hz
GB                     0
PC                  1.00
```

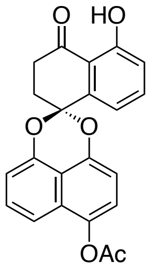

16

^{13}C NMR (150 MHz, $CDCl_3$)

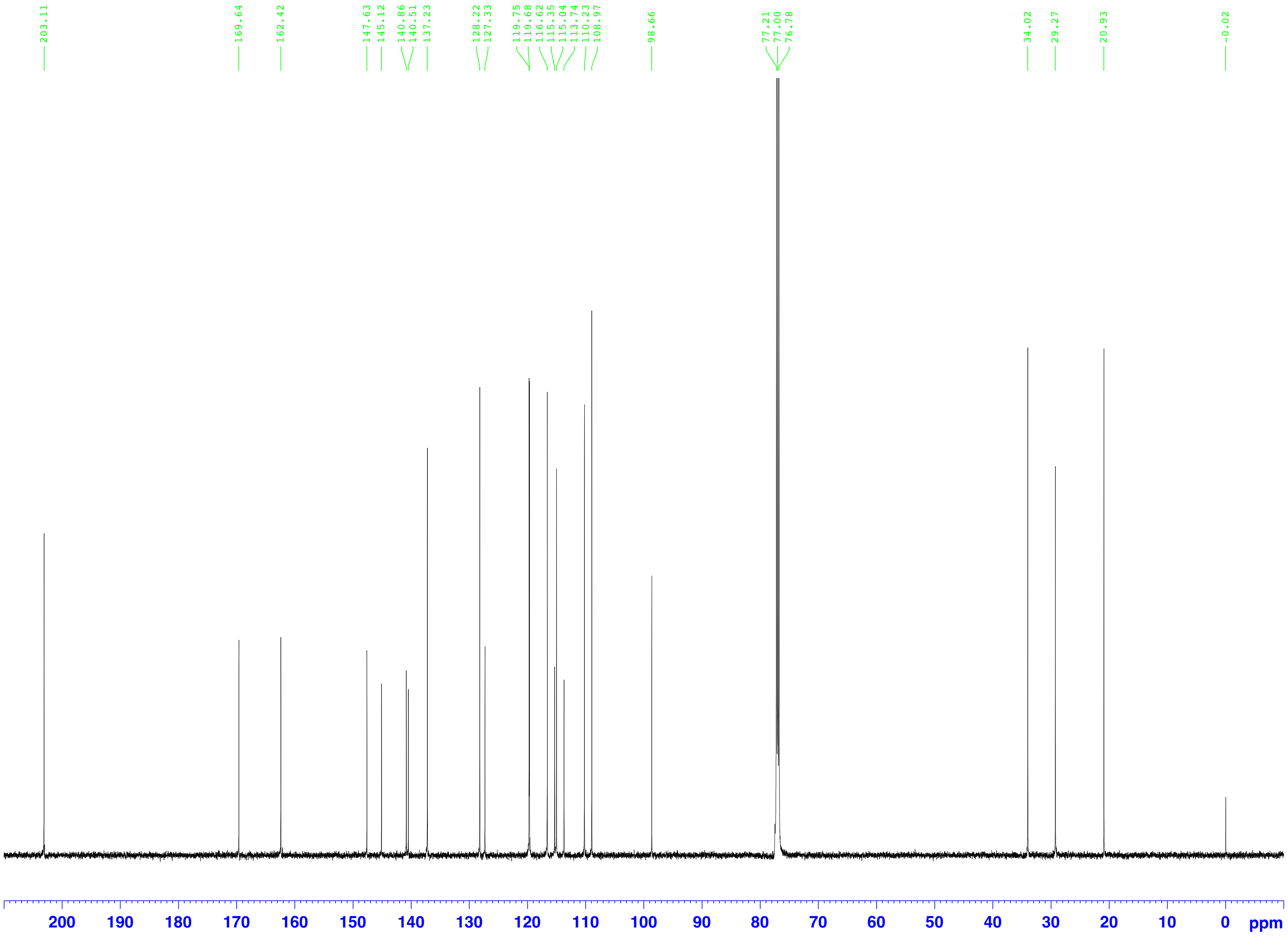

Current Data Parameters
NAME DO1-62-ptlc-2
EXPNO 13
PROCNO 1

F2 - Acquisition Parameters
Date_ 20210205
Time 0.01
INSTRUM spect
PROBHD 5 mm CPPBBO BB
PULPROG zgpg30
TD 65536
SOLVENT CDCl3
NS 1024
DS 4
SWH 36057.691 Hz
FIDRES 0.550197 Hz
AQ 0.9087659 sec
RG 175.56
DW 13.867 usec
DE 18.00 usec
TE 298.1 K
D1 2.00000000 sec
D11 0.03000000 sec
TD0 1

======== CHANNEL f1 ========
SFO1 150.9178981 MHz
NUC1 13C
P1 10.00 usec
PLW1 80.00000000 W

======== CHANNEL f2 ========
SFO2 600.1324005 MHz
NUC2 1H
CPDPRG[2 waltz16
PCPD2 70.00 usec
PLW2 13.43999958 W
PLW12 0.61714000 W
PLW13 0.31042001 W

F2 - Processing parameters
SI 32768
SF 150.9028145 MHz
WDW EM
SSB 0
LB 1.00 Hz
GB 0
PC 1.40

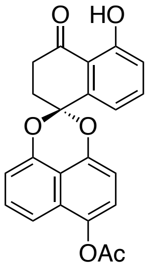

16

^{1}H NMR (600 MHz, $CDCl_3$)

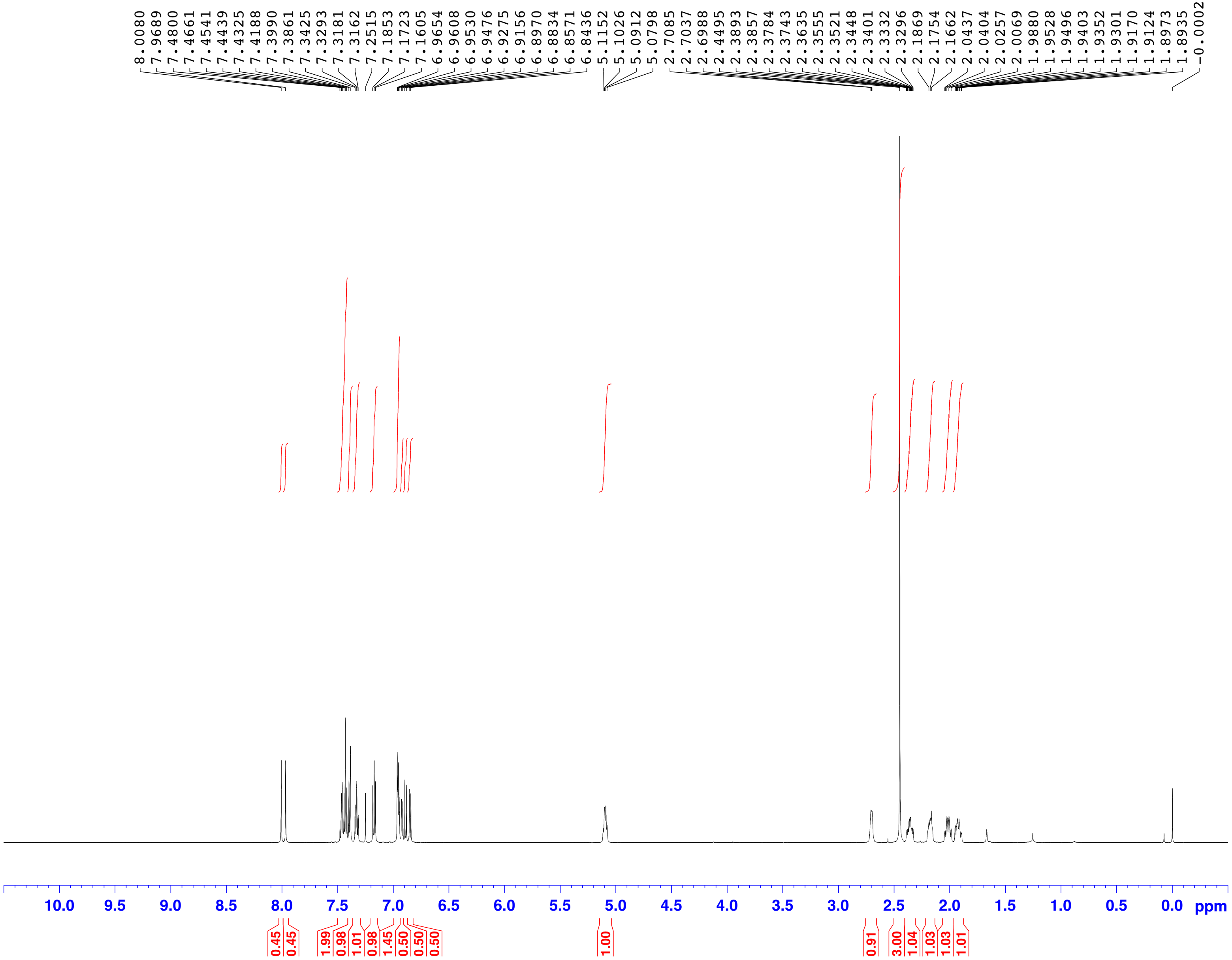

Current Data Parameters
NAME DO1-255-ptlc
EXPNO 21
PROCNO 1

F2 - Acquisition Parameters
Date_ 20220123
Time 20.02
INSTRUM spect
PROBHD 5 mm CPPBBO BB
PULPROG zg30
TD 65536
SOLVENT CDCl3
NS 16
DS 2
SWH 12019.230 Hz
FIDRES 0.183399 Hz
AQ 2.7262976 sec
RG 31.94
DW 41.600 usec
DE 10.00 usec
TE 298.2 K
D1 1.00000000 sec
TD0 1

======== CHANNEL f1 ========
SFO1 600.1337060 MHz
NUC1 1H
P1 12.00 usec
PLW1 21.00000000 W

F2 - Processing parameters
SI 65536
SF 600.1300196 MHz
WDW EM
SSB 0
LB 0.30 Hz
GB 0
PC 1.00

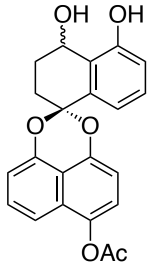

S14

dr = 1:1

^{13}C NMR (150 MHz, CDCl$_3$)

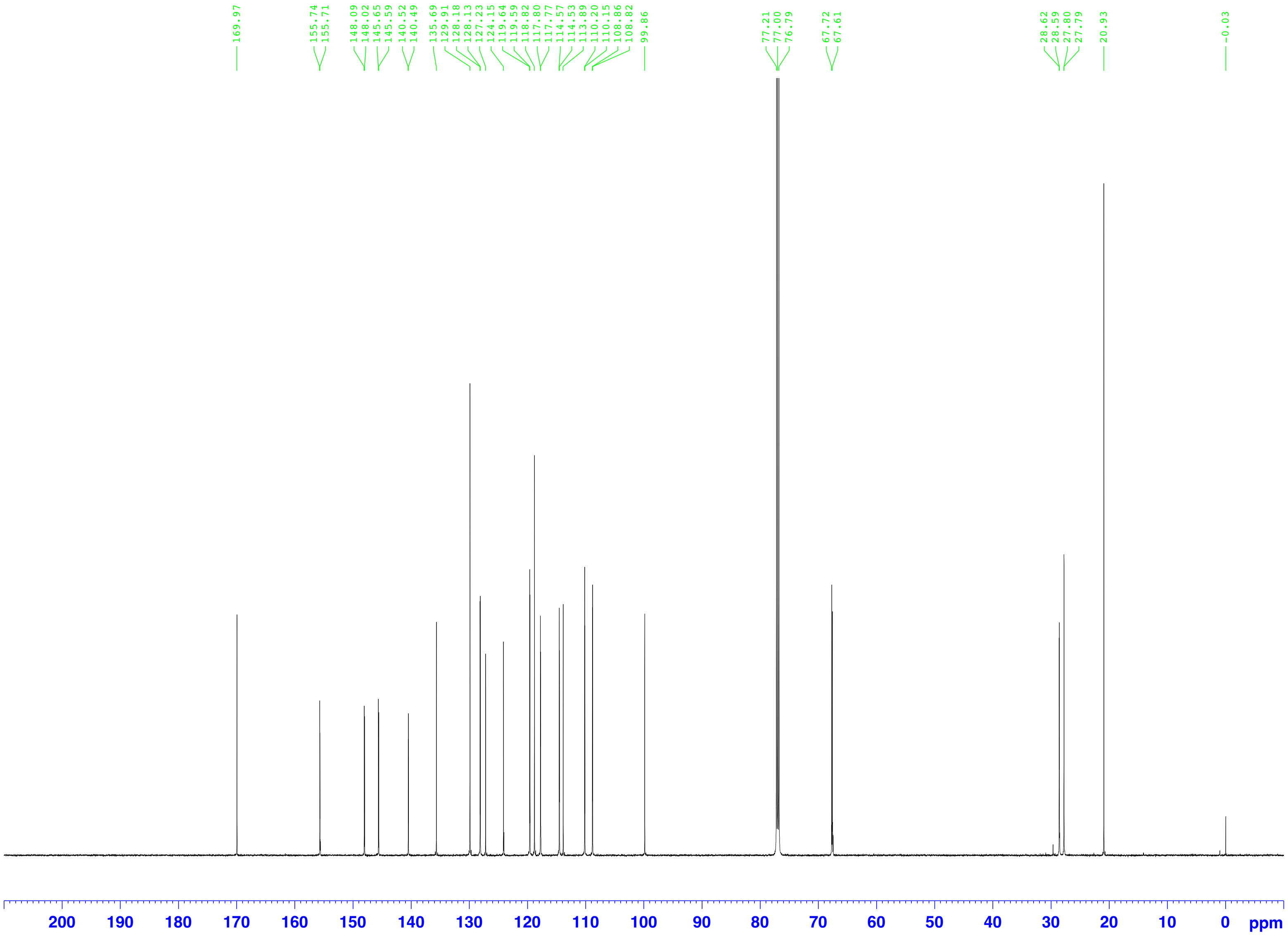

BRUKER

```
Current Data Parameters
NAME          DO1-255-ptlc
EXPNO                   22
PROCNO                   1

F2 - Acquisition Parameters
Date_             20220123
Time                 23.26
INSTRUM              spect
PROBHD    5 mm CPPBBO BB
PULPROG            zgpg30
TD                   65536
SOLVENT              CDCl3
NS                    4096
DS                       4
SWH             36057.691 Hz
FIDRES           0.550197 Hz
AQ              0.9087659 sec
RG                  175.56
DW                  13.867 usec
DE                   18.00 usec
TE                   298.2 K
D1              2.00000000 sec
D11             0.03000000 sec
TD0                      1

======== CHANNEL f1 ========
SFO1          150.9178981 MHz
NUC1                   13C
P1                   10.00 usec
PLW1           80.00000000 W

======== CHANNEL f2 ========
SFO2          600.1324005 MHz
NUC2                    1H
CPDPRG[2           waltz16
PCPD2                70.00 usec
PLW2           13.43999958 W
PLW12           0.61714000 W
PLW13           0.31042001 W

F2 - Processing parameters
SI                   32768
SF            150.9028165 MHz
WDW                     EM
SSB                      0
LB                    1.00 Hz
GB                       0
PC                    1.40
```

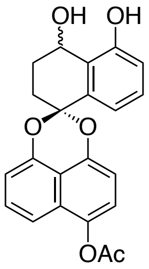

S14

dr = 1:1

^{1}H NMR (600 MHz, $CDCl_3$)

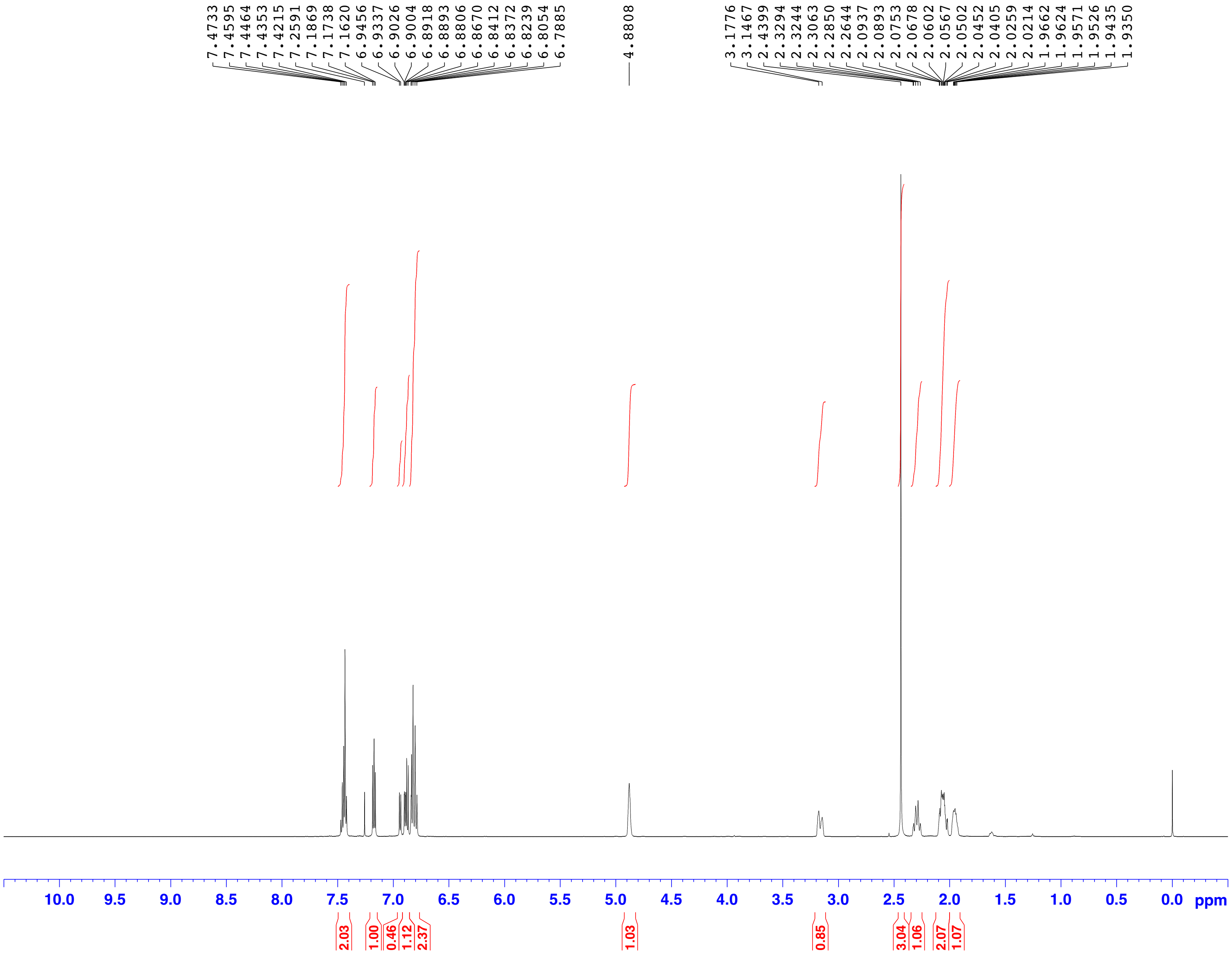


```
Current Data Parameters
NAME      DO1-290-autocolumn
EXPNO                   40
PROCNO                   1

F2 - Acquisition Parameters
Date_             20220121
Time                 20.02
INSTRUM              spect
PROBHD     5 mm CPPBBO BB
PULPROG               zg30
TD                   65536
SOLVENT              CDCl3
NS                      16
DS                       2
SWH              12019.230 Hz
FIDRES            0.183399 Hz
AQ               2.7262976 sec
RG                    17.5
DW                  41.600 usec
DE                   10.00 usec
TE                   298.0 K
D1              1.00000000 sec
TD0                      1

======== CHANNEL f1 ========
SFO1           600.1337060 MHz
NUC1                    1H
P1                   12.00 usec
PLW1           21.00000000 W

F2 - Processing parameters
SI                   65536
SF             600.1300150 MHz
WDW                     EM
SSB                      0
LB                    0.30 Hz
GB                       0
PC                    1.00
```

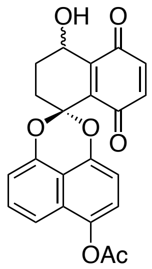

23

dr = 1:1

^{13}C NMR (150 MHz, $CDCl_3$)

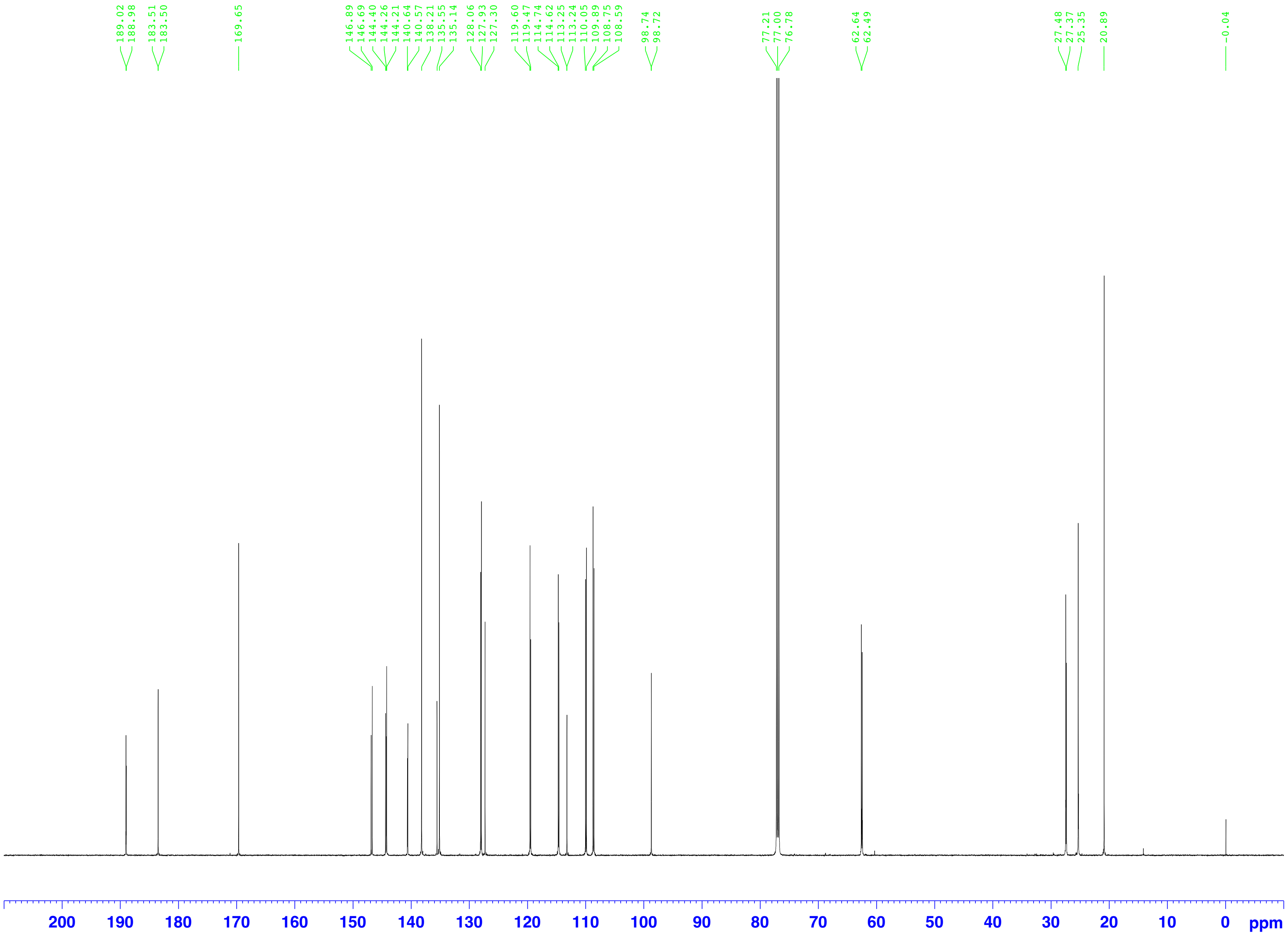


```
Current Data Parameters
NAME      DO1-290-autocolumn
EXPNO                     42
PROCNO                     1

F2 - Acquisition Parameters
Date_               20220123
Time                   17.31
INSTRUM                spect
PROBHD     5 mm CPPBBO BB
PULPROG              zgpg30
TD                     65536
SOLVENT                CDCl3
NS                      4096
DS                         4
SWH               36057.691 Hz
FIDRES             0.550197 Hz
AQ                0.9087659 sec
RG                    175.56
DW                    13.867 usec
DE                     18.00 usec
TE                     298.2 K
D1                2.00000000 sec
D11               0.03000000 sec
TD0                        1

======== CHANNEL f1 ========
SFO1             150.9178981 MHz
NUC1                     13C
P1                     10.00 usec
PLW1             80.00000000 W

======== CHANNEL f2 ========
SFO2             600.1324005 MHz
NUC2                      1H
CPDPRG[2             waltz16
PCPD2                  70.00 usec
PLW2             13.43999958 W
PLW12             0.61714000 W
PLW13             0.31042001 W

F2 - Processing parameters
SI                     32768
SF               150.9028187 MHz
WDW                       EM
SSB                        0
LB                      1.00 Hz
GB                         0
PC                      1.40
```

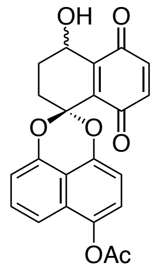

23

dr = 1:1

^{1}H NMR (600 MHz, $CDCl_3$)

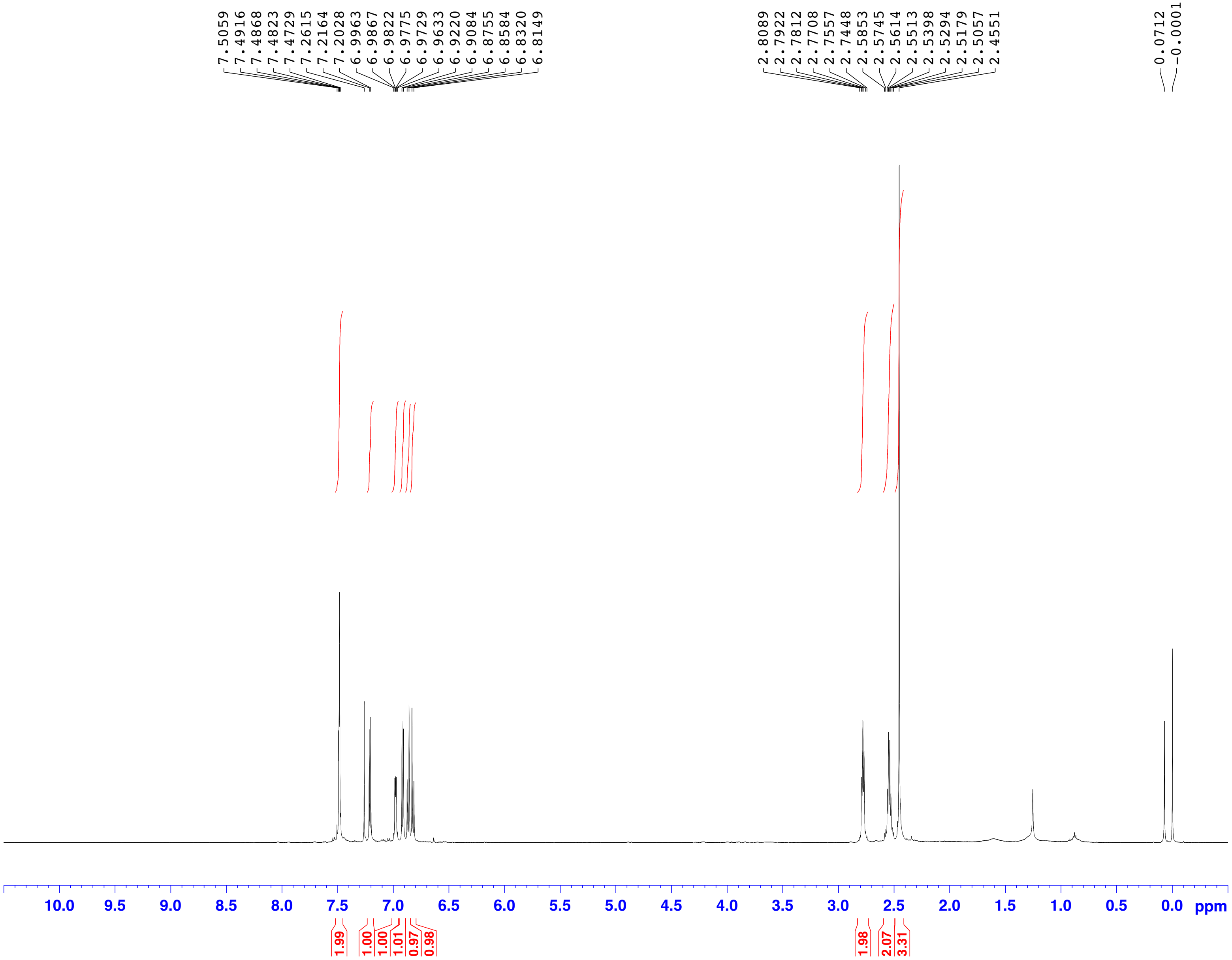


```
Current Data Parameters
NAME        DO1-303-crude
EXPNO                  20
PROCNO                  1

F2 - Acquisition Parameters
Date_            20220203
Time                19.31
INSTRUM             spect
PROBHD    5 mm CPPBBO BB
PULPROG              zg30
TD                  65536
SOLVENT             CDCl3
NS                     16
DS                      2
SWH             12019.230 Hz
FIDRES           0.183399 Hz
AQ              2.7262976 sec
RG                   17.5
DW                 41.600 usec
DE                  10.00 usec
TE                  298.1 K
D1             1.00000000 sec
TD0                     1

======== CHANNEL f1 ========
SFO1          600.1337060 MHz
NUC1                   1H
P1                  12.00 usec
PLW1          21.00000000 W

F2 - Processing parameters
SI                  65536
SF            600.1300135 MHz
WDW                    EM
SSB                     0
LB                   0.30 Hz
GB                      0
PC                   1.00
```

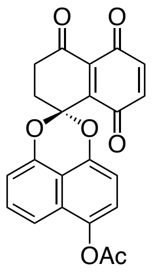

24
(crude)

^{13}C NMR (150 MHz, $CDCl_3$)

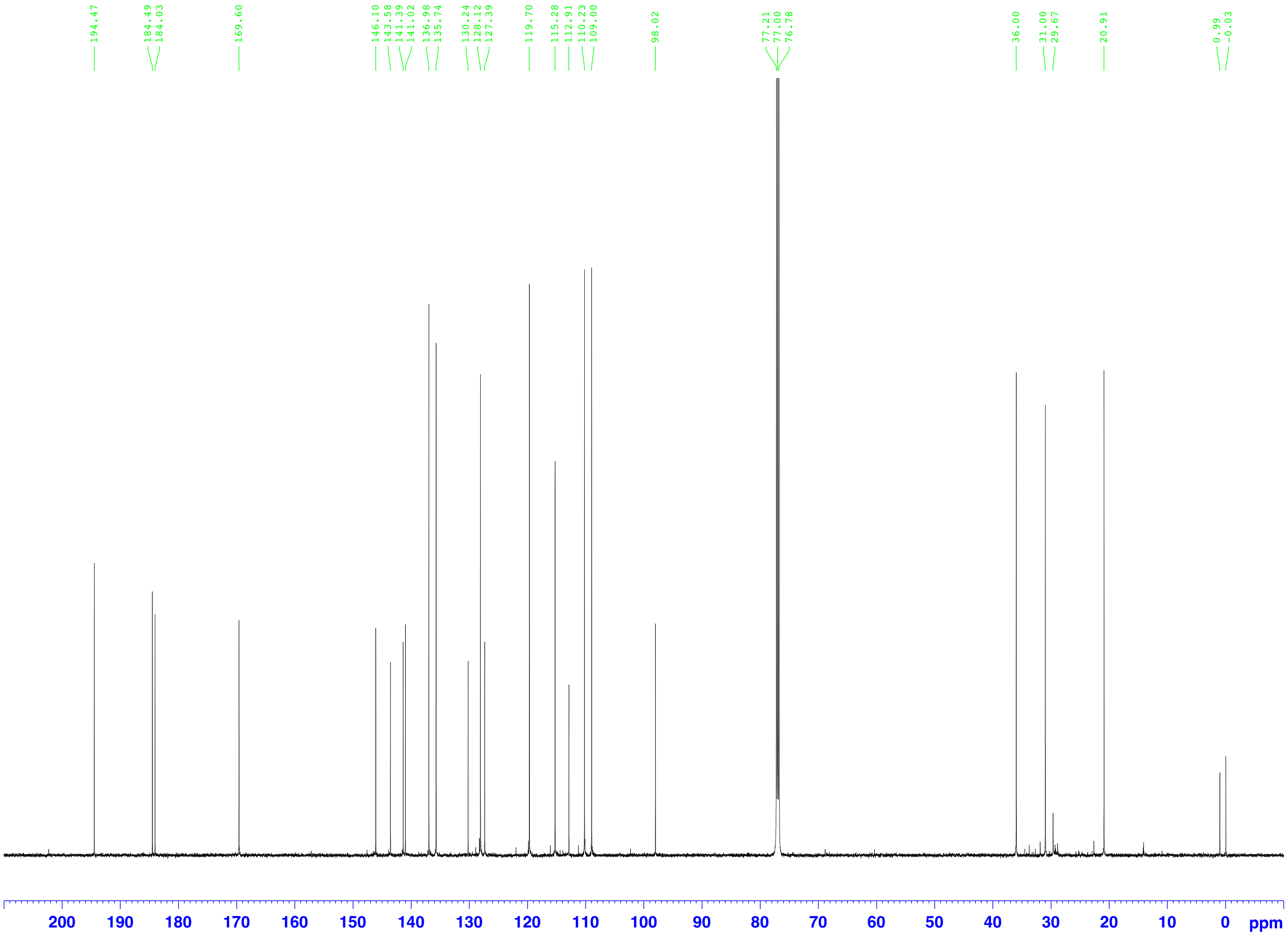


```
Current Data Parameters
NAME          DO1-303-crude
EXPNO                    31
PROCNO                    1

F2 - Acquisition Parameters
Date_              20220204
Time                   1.29
INSTRUM               spect
PROBHD    5 mm CPPBBO BB
PULPROG             zgpg30
TD                    65536
SOLVENT               CDCl3
NS                     4096
DS                        4
SWH              36057.691 Hz
FIDRES            0.550197 Hz
AQ               0.9087659 sec
RG                   175.56
DW                   13.867 usec
DE                    18.00 usec
TE                    298.2 K
D1               2.00000000 sec
D11              0.03000000 sec
TD0                       1

======== CHANNEL f1 ========
SFO1           150.9178981 MHz
NUC1                    13C
P1                    10.00 usec
PLW1           80.00000000 W

======== CHANNEL f2 ========
SFO2           600.1324005 MHz
NUC2                     1H
CPDPRG[2            waltz16
PCPD2                 70.00 usec
PLW2           13.43999958 W
PLW12           0.61714000 W
PLW13           0.31042001 W

F2 - Processing parameters
SI                    32768
SF             150.9028144 MHz
WDW                      EM
SSB                       0
LB                     1.00 Hz
GB                        0
PC                     1.40
```

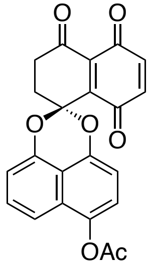

24
(crude)

^{1}H NMR (600 MHz, $CDCl_3$)

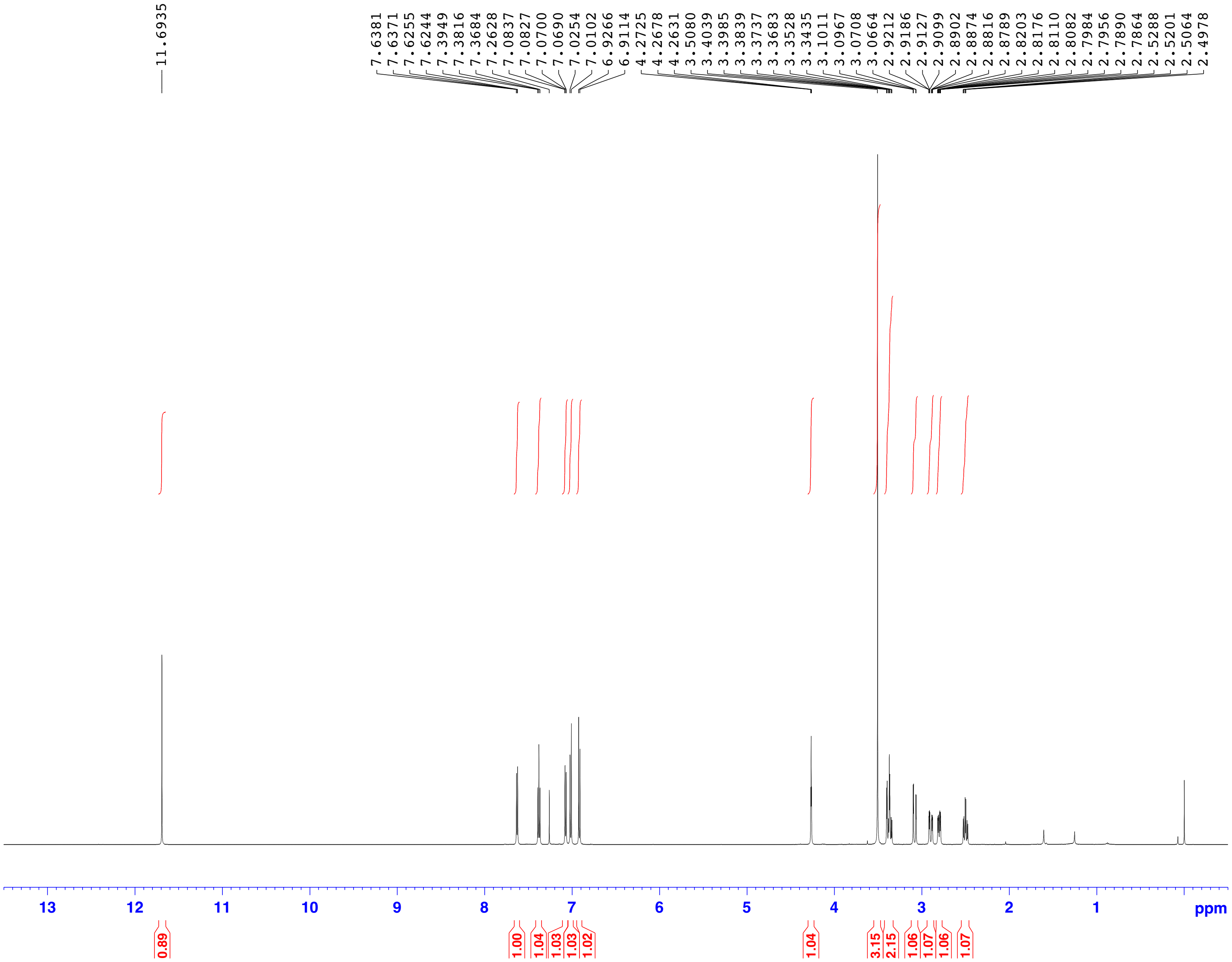

Current Data Parameters
NAME DO1-287-ptlc
EXPNO 10
PROCNO 1

F2 - Acquisition Parameters
Date_ 20220120
Time 20.04
INSTRUM spect
PROBHD 5 mm CPPBBO BB
PULPROG zg30
TD 65536
SOLVENT CDCl3
NS 16
DS 2
SWH 12019.230 Hz
FIDRES 0.183399 Hz
AQ 2.7262976 sec
RG 17.5
DW 41.600 usec
DE 10.00 usec
TE 298.1 K
D1 1.00000000 sec
TD0 1

======== CHANNEL f1 ========
SFO1 600.1337060 MHz
NUC1 1H
P1 12.00 usec
PLW1 21.00000000 W

F2 - Processing parameters
SI 65536
SF 600.1300126 MHz
WDW EM
SSB 0
LB 0.30 Hz
GB 0
PC 1.00

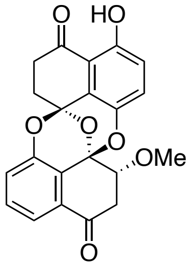

preussomerin EG_3 (**5**)

^{13}C NMR (150 MHz, $CDCl_3$)

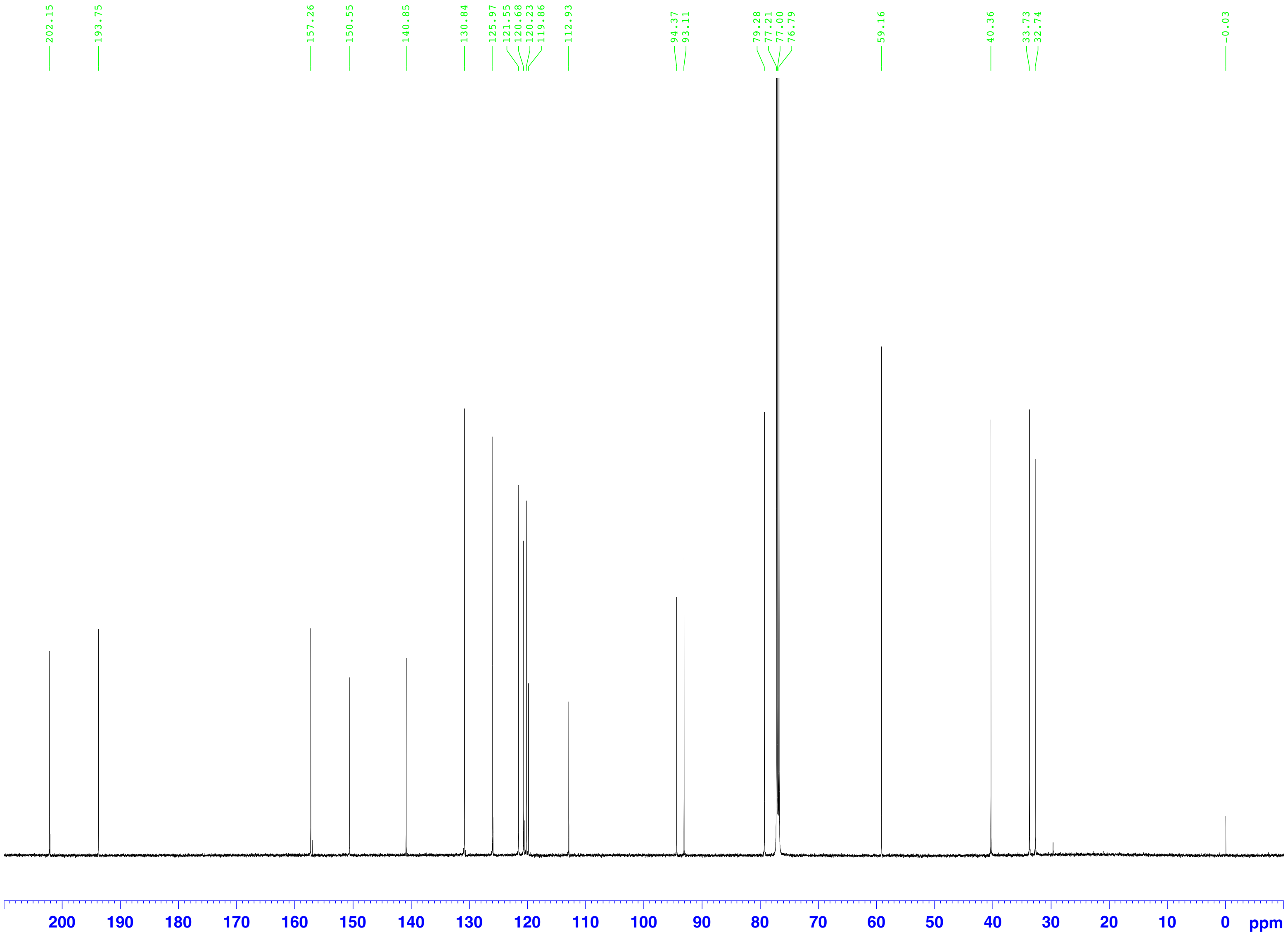


```
Current Data Parameters
NAME        DO1-287-ptlc
EXPNO                 12
PROCNO                 1

F2 - Acquisition Parameters
Date_           20220121
Time                1.27
INSTRUM            spect
PROBHD   5 mm CPPBBO BB
PULPROG          zgpg30
TD                 65536
SOLVENT            CDCl3
NS                  4096
DS                     4
SWH           36057.691 Hz
FIDRES         0.550197 Hz
AQ            0.9087659 sec
RG               175.56
DW               13.867 usec
DE                18.00 usec
TE                298.2 K
D1           2.00000000 sec
D11          0.03000000 sec
TD0                    1

======== CHANNEL f1 ========
SFO1        150.9178981 MHz
NUC1                 13C
P1                 10.00 usec
PLW1        80.00000000 W

======== CHANNEL f2 ========
SFO2        600.1324005 MHz
NUC2                  1H
CPDPRG[2         waltz16
PCPD2              70.00 usec
PLW2         13.43999958 W
PLW12         0.61714000 W
PLW13         0.31042001 W

F2 - Processing parameters
SI                 32768
SF          150.9028150 MHz
WDW                   EM
SSB                    0
LB                  1.00 Hz
GB                     0
PC                  1.40
```

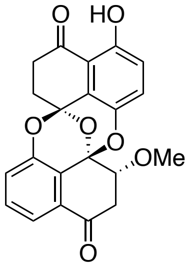

preussomerin EG_3 (**5**)

^{1}H NMR (600 MHz, $CDCl_3$)

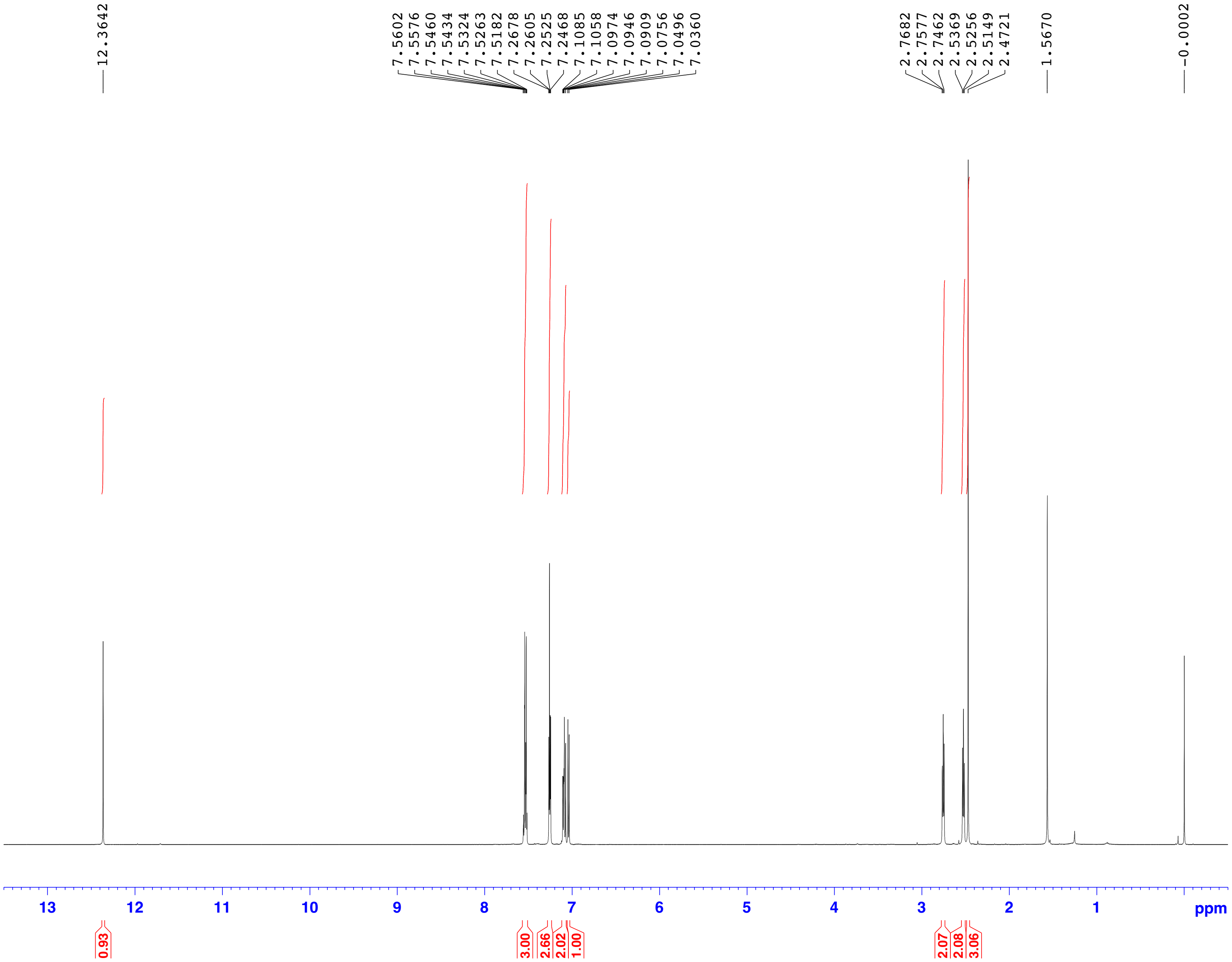


```
Current Data Parameters
NAME          DO1-342-ptlc
EXPNO                   10
PROCNO                   1

F2 - Acquisition Parameters
Date_             20220427
Time                 21.16
INSTRUM              spect
PROBHD   5 mm CPPBBO BB
PULPROG               zg30
TD                   65536
SOLVENT              CDCl3
NS                      16
DS                       2
SWH              12019.230 Hz
FIDRES            0.183399 Hz
AQ               2.7262976 sec
RG                   31.94
DW                  41.600 usec
DE                   10.00 usec
TE                   298.1 K
D1              1.00000000 sec
TD0                      1

======== CHANNEL f1 ========
SFO1           600.1337060 MHz
NUC1                    1H
P1                   12.00 usec
PLW1           21.00000000 W

F2 - Processing parameters
SI                   65536
SF             600.1300141 MHz
WDW                     EM
SSB                      0
LB                    0.30 Hz
GB                       0
PC                    0.10
```

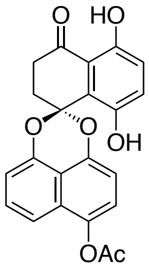

26

^{13}C NMR (150 MHz, $CDCl_3$)

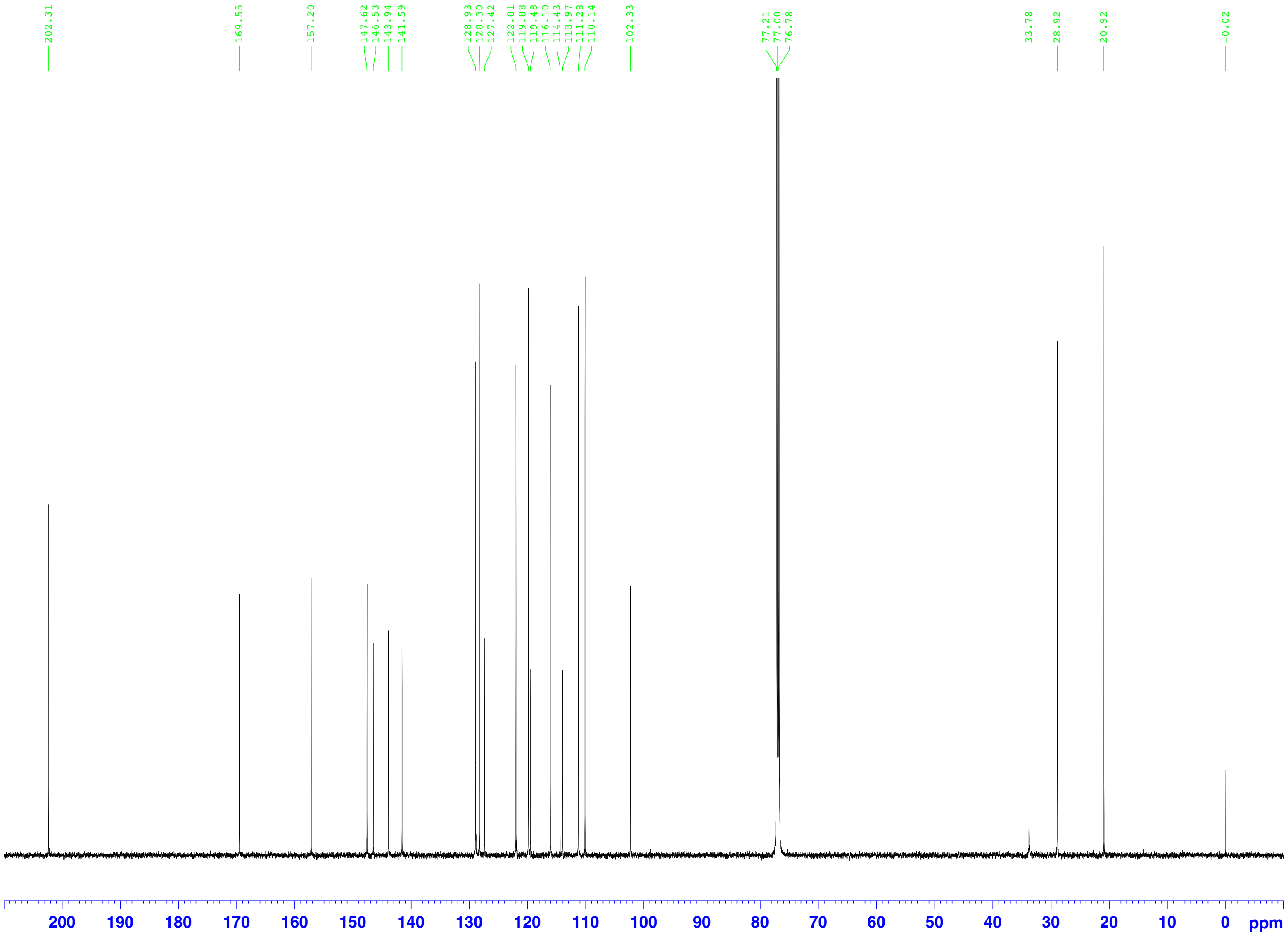

BRUKER

Current Data Parameters
NAME DO1-342-ptlc
EXPNO 21
PROCNO 1

F2 - Acquisition Parameters
Date_ 20220428
Time 3.05
INSTRUM spect
PROBHD 5 mm CPPBBO BB
PULPROG zgpg30
TD 65536
SOLVENT CDCl3
NS 6000
DS 4
SWH 36057.691 Hz
FIDRES 0.550197 Hz
AQ 0.9087659 sec
RG 175.56
DW 13.867 usec
DE 18.00 usec
TE 298.1 K
D1 2.00000000 sec
D11 0.03000000 sec
TD0 1

======== CHANNEL f1 ========
SFO1 150.9178981 MHz
NUC1 13C
P1 10.00 usec
PLW1 80.00000000 W

======== CHANNEL f2 ========
SFO2 600.1324005 MHz
NUC2 1H
CPDPRG[2 waltz16
PCPD2 70.00 usec
PLW2 13.43999958 W
PLW12 0.61714000 W
PLW13 0.31042001 W

F2 - Processing parameters
SI 32768
SF 150.9028131 MHz
WDW EM
SSB 0
LB 1.00 Hz
GB 0
PC 1.40

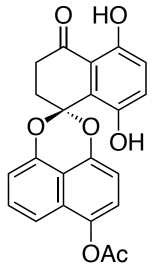

26

^{1}H NMR (600 MHz, acetone-d_6)

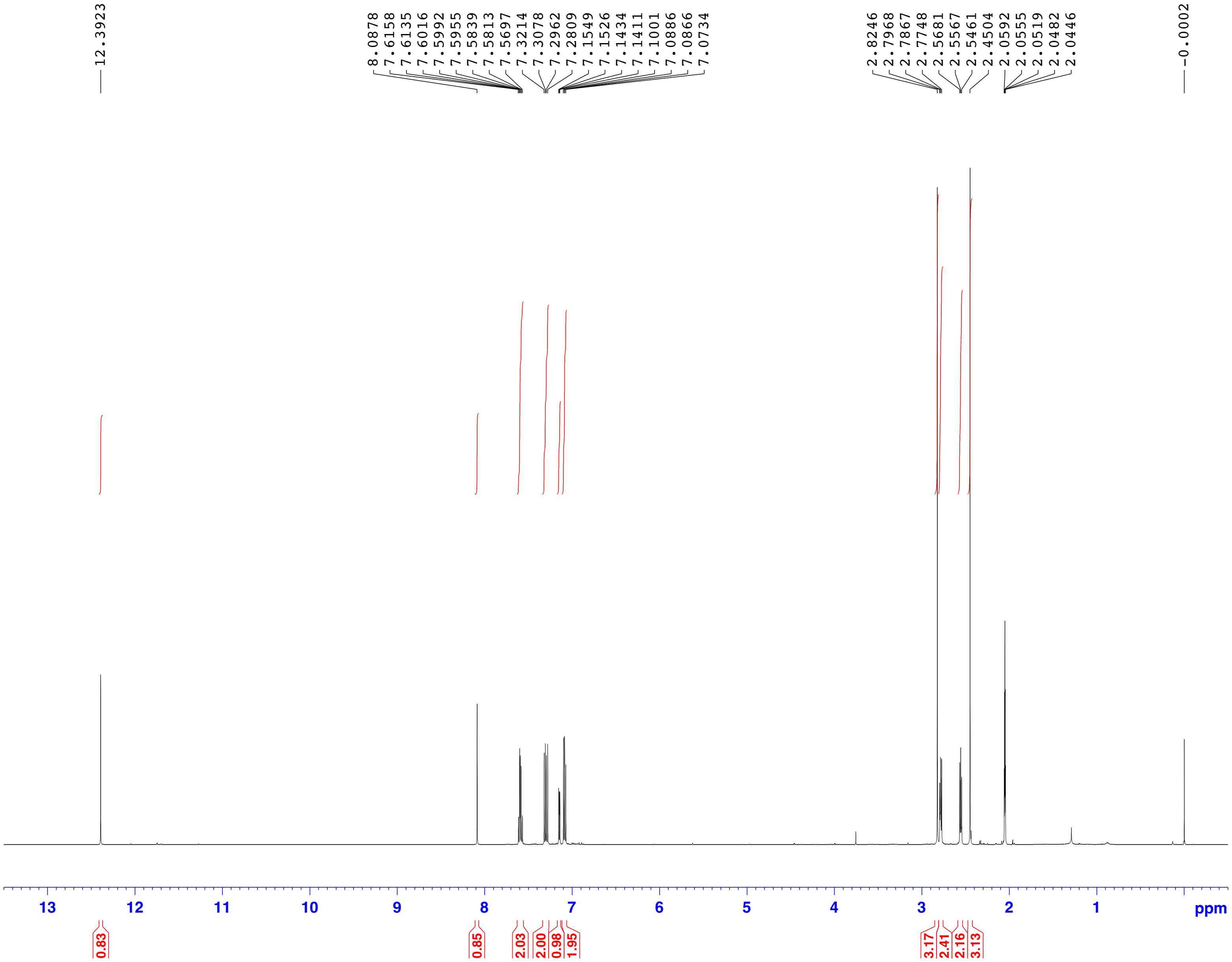


```
Current Data Parameters
NAME          DO1-342-ptlc
EXPNO                   31
PROCNO                   1

F2 - Acquisition Parameters
Date_             20220429
Time                  2.33
INSTRUM              spect
PROBHD    5 mm CPPBBO BB
PULPROG               zg30
TD                   65536
SOLVENT            Acetone
NS                      16
DS                       2
SWH              12019.230 Hz
FIDRES            0.183399 Hz
AQ               2.7262976 sec
RG                   31.94
DW                  41.600 usec
DE                   10.00 usec
TE                   298.1 K
D1              1.00000000 sec
TD0                      1

======== CHANNEL f1 ========
SFO1           600.1337060 MHz
NUC1                    1H
P1                   12.00 usec
PLW1           21.00000000 W

F2 - Processing parameters
SI                   65536
SF             600.1300091 MHz
WDW                     EM
SSB                      0
LB                    0.30 Hz
GB                       0
PC                    1.00
```

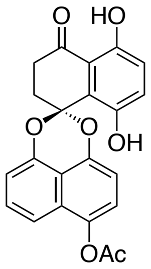

26

^{13}C NMR (150 MHz, acetone-d_6)

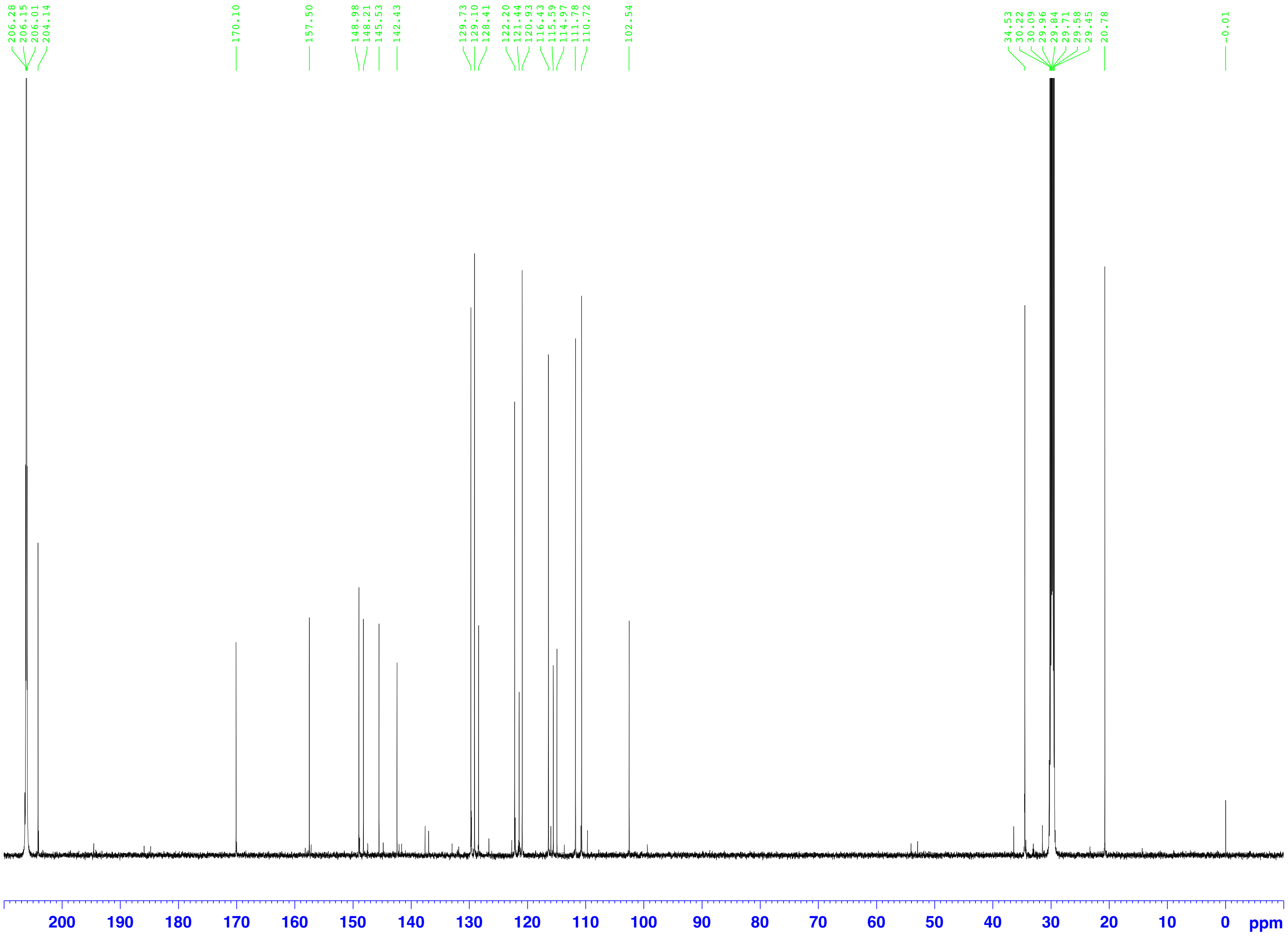


```
Current Data Parameters
NAME          DO1-342-ptlc
EXPNO                   32
PROCNO                   1

F2 - Acquisition Parameters
Date_             20220429
Time                  7.31
INSTRUM              spect
PROBHD     5 mm CPPBBO BB
PULPROG            zgpg30
TD                   65536
SOLVENT            Acetone
NS                    6000
DS                       4
SWH              36057.691 Hz
FIDRES            0.550197 Hz
AQ               0.9087659 sec
RG                  175.56
DW                  13.867 usec
DE                   18.00 usec
TE                   298.1 K
D1              2.00000000 sec
D11             0.03000000 sec
TD0                      1

======== CHANNEL f1 ========
SFO1        150.9178981 MHz
NUC1                   13C
P1                   10.00 usec
PLW1           80.00000000 W

======== CHANNEL f2 ========
SFO2        600.1324005 MHz
NUC2                    1H
CPDPRG[2           waltz16
PCPD2                70.00 usec
PLW2           13.43999958 W
PLW12           0.61714000 W
PLW13           0.31042001 W

F2 - Processing parameters
SI                   32768
SF          150.9026735 MHz
WDW                     EM
SSB                      0
LB                    1.00 Hz
GB                       0
PC                    1.40
```

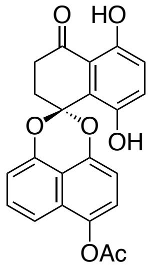

26

^{1}H NMR (600 MHz, $CDCl_3$)

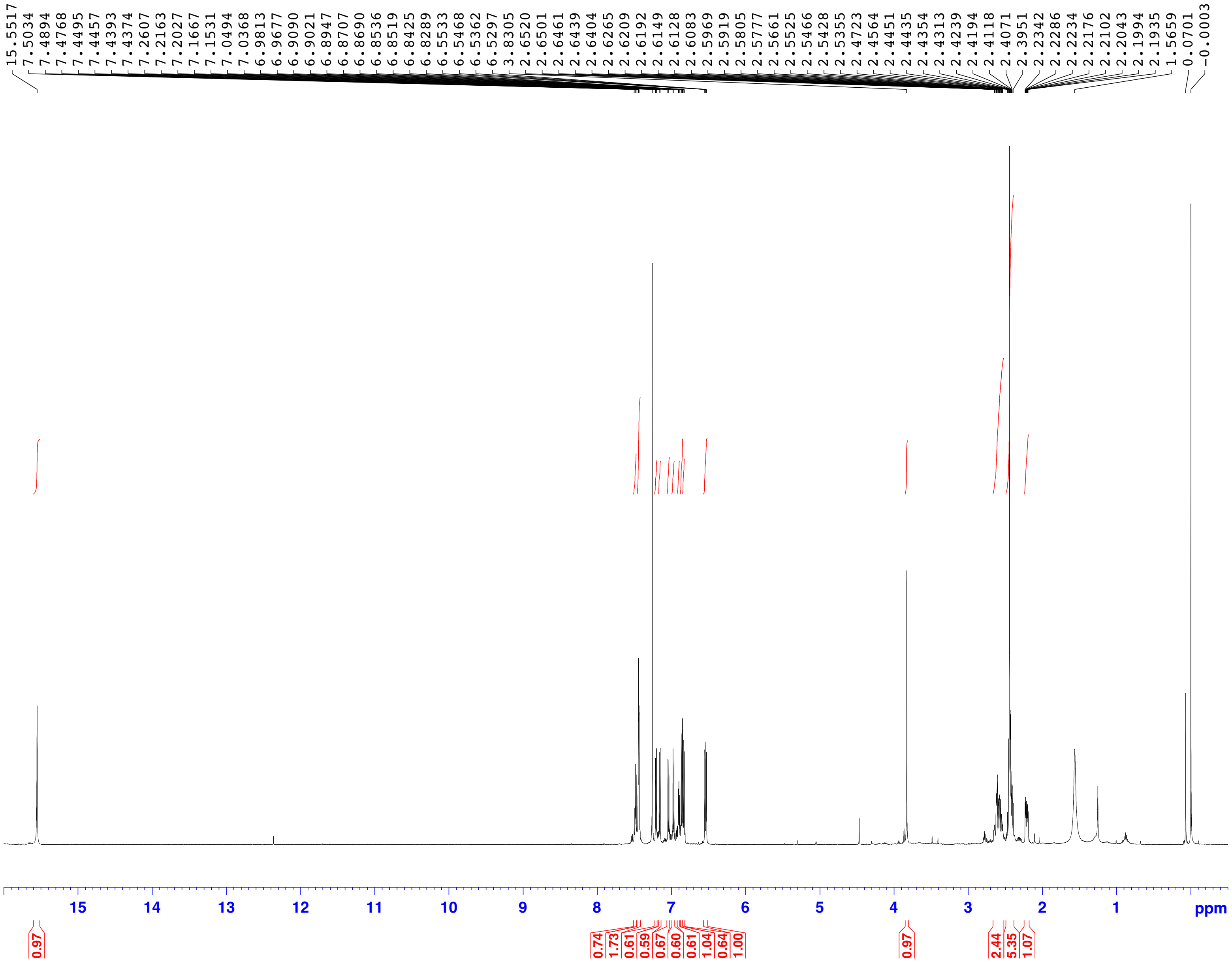

BRUKER

```
Current Data Parameters
NAME          DO1-318-ptlc2
EXPNO                    10
PROCNO                    1

F2 - Acquisition Parameters
Date_              20220405
Time                  18.36
INSTRUM               spect
PROBHD    5 mm CPPBBO BB
PULPROG                zg30
TD                    65536
SOLVENT               CDCl3
NS                       16
DS                        2
SWH               12019.230 Hz
FIDRES             0.183399 Hz
AQ                2.7262976 sec
RG                    31.94
DW                   41.600 usec
DE                    10.00 usec
TE                    298.1 K
D1               1.00000000 sec
TD0                       1

======== CHANNEL f1 ========
SFO1            600.1337060 MHz
NUC1                     1H
P1                    12.00 usec
PLW1            21.00000000 W

F2 - Processing parameters
SI                    65536
SF              600.1300138 MHz
WDW                      EM
SSB                       0
LB                     0.30 Hz
GB                        0
PC                        0
```

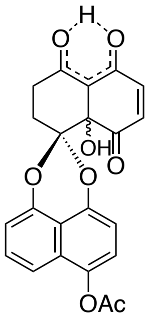

29
(dr = 1:1)

^{13}C NMR (150 MHz, $CDCl_3$)

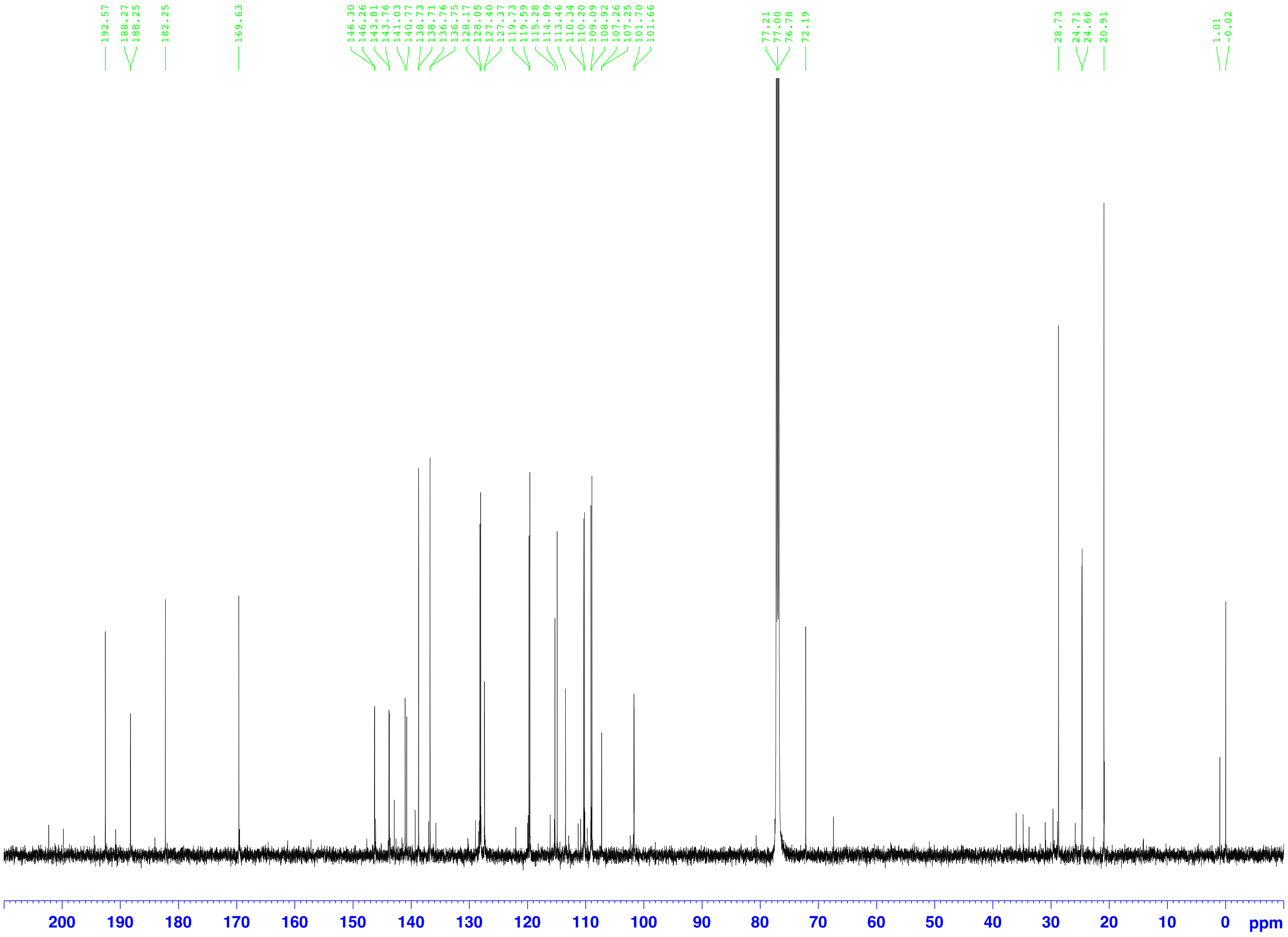


```
Current Data Parameters
NAME        DO1-318-ptlc2
EXPNO                  12
PROCNO                  1

F2 - Acquisition Parameters
Date_            20220406
Time                 7.09
INSTRUM             spect
PROBHD     5 mm CPPBBO BB
PULPROG            zgpg30
TD                  65536
SOLVENT             CDCl3
NS                   4096
DS                      4
SWH             36057.691 Hz
FIDRES           0.550197 Hz
AQ              0.9087659 sec
RG                 175.56
DW                 13.867 usec
DE                  18.00 usec
TE                  298.1 K
D1             2.00000000 sec
D11            0.03000000 sec
TD0                     1

======== CHANNEL f1 ========
SFO1          150.9178981 MHz
NUC1                  13C
P1                  10.00 usec
PLW1          80.00000000 W

======== CHANNEL f2 ========
SFO2          600.1324005 MHz
NUC2                   1H
CPDPRG[2          waltz16
PCPD2               70.00 usec
PLW2          13.43999958 W
PLW12          0.61714000 W
PLW13          0.31042001 W

F2 - Processing parameters
SI                  32768
SF            150.9028124 MHz
WDW                    EM
SSB                     0
LB                   1.00 Hz
GB                      0
PC                   1.40
```

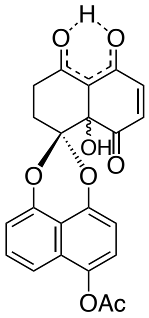

29
(dr = 1:1)

HSQC

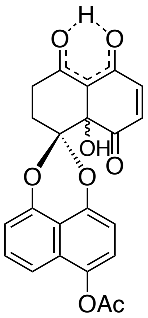

29
(dr = 1:1)

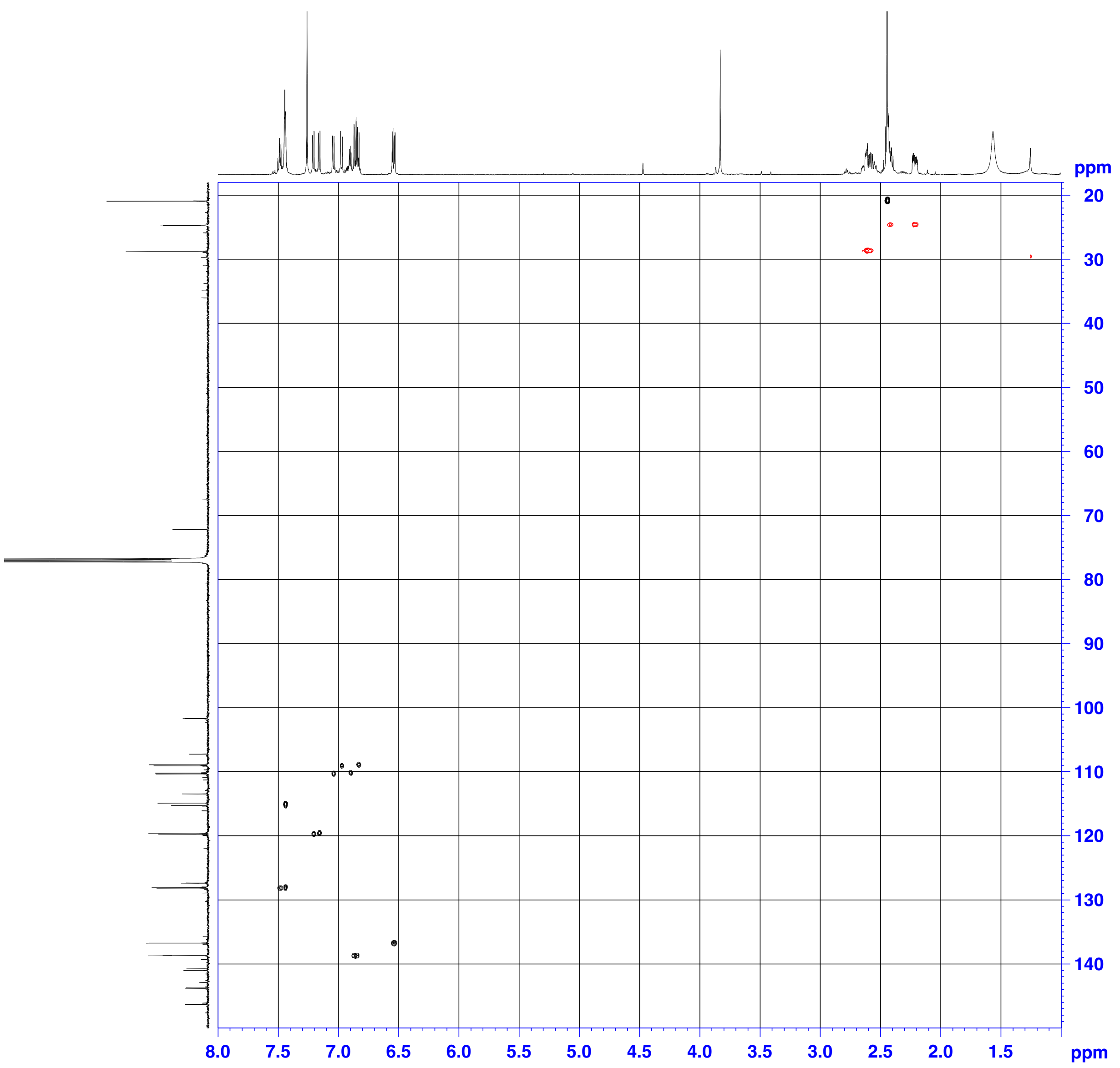


```
Current Data Parameters
NAME       DO1-318-ptlc2
EXPNO                 15
PROCNO                 1

F2 - Acquisition Parameters
Date_           20220406
Time                8.15
INSTRUM            spect
PROBHD   5 mm CPPBBO BB
PULPROG  hsqcedetgpsisp2.3
TD                  2048
SOLVENT            CDCl3
NS                     4
DS                    32
SWH             7211.539 Hz
FIDRES          3.521259 Hz
AQ             0.1419947 sec
RG                175.56
DW                69.333 usec
DE                 10.00 usec
TE                 298.1 K
CNST2        145.0000000
CNST17        -0.5000000
D0            0.00000300 sec
D1            2.00000000 sec
D4            0.00172414 sec
D11           0.03000000 sec
D16           0.00020000 sec
D21           0.00360000 sec
D24           0.00089000 sec
IN0           0.00002000 sec

======== CHANNEL f1 ========
SFO1         600.1328224 MHz
NUC1                  1H
P1                 12.00 usec
P2                 24.00 usec
P28                    0 usec
PLW1         21.00000000 W

======== CHANNEL f2 ========
SFO2         150.9133710 MHz
NUC2                 13C
CPDPRG[2            garp
P3                 10.00 usec
P14               500.00 usec
P24              2000.00 usec
P31              1730.00 usec
PCPD2              60.00 usec
PLW0                   0 W
PLW2         80.00000000 W
PLW12         2.22219992 W
SPNAM[3] Crp60,0.5,20.1
SPOAL3             0.500
SPOFFS3                0 Hz
SPW3         12.22299957 W
SPNAM[7]    Crp60comp.4
SPOAL7             0.500
SPOFFS7                0 Hz
SPW7         12.22299957 W
SPNAM[18  Crp60_xfilt.2
SPOAL18            0.500
SPOFFS18               0 Hz
SPW18         3.53270006 W

====== GRADIENT CHANNEL =====
GPNAM[1]      SMSQ10.100
GPNAM[2]      SMSQ10.100
GPNAM[3]      SMSQ10.100
GPNAM[4]      SMSQ10.100
GPZ1               80.00 %
GPZ2               20.10 %
GPZ3               11.00 %
GPZ4               -5.00 %
P16              1000.00 usec
P19               600.00 usec

F1 - Acquisition parameters
TD                   256
SFO1            150.9134 MHz
FIDRES        195.312500 Hz
SW               165.658 ppm
FnMODE      Echo-Antiecho

F2 - Processing parameters
SI                  1024
SF           600.1300138 MHz
WDW                QSINE
SSB                    2
LB                     0 Hz
GB                     0
PC                  1.40

F1 - Processing parameters
SI                  1024
MC2          echo-antiecho
SF           150.9028124 MHz
WDW                QSINE
SSB                    2
LB                     0 Hz
GB                     0
```

HMBC

key HMBC correlations

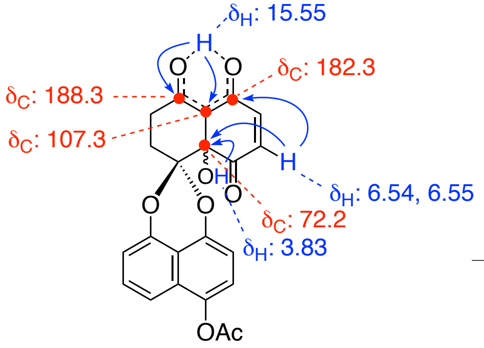

29
(dr = 1:1)

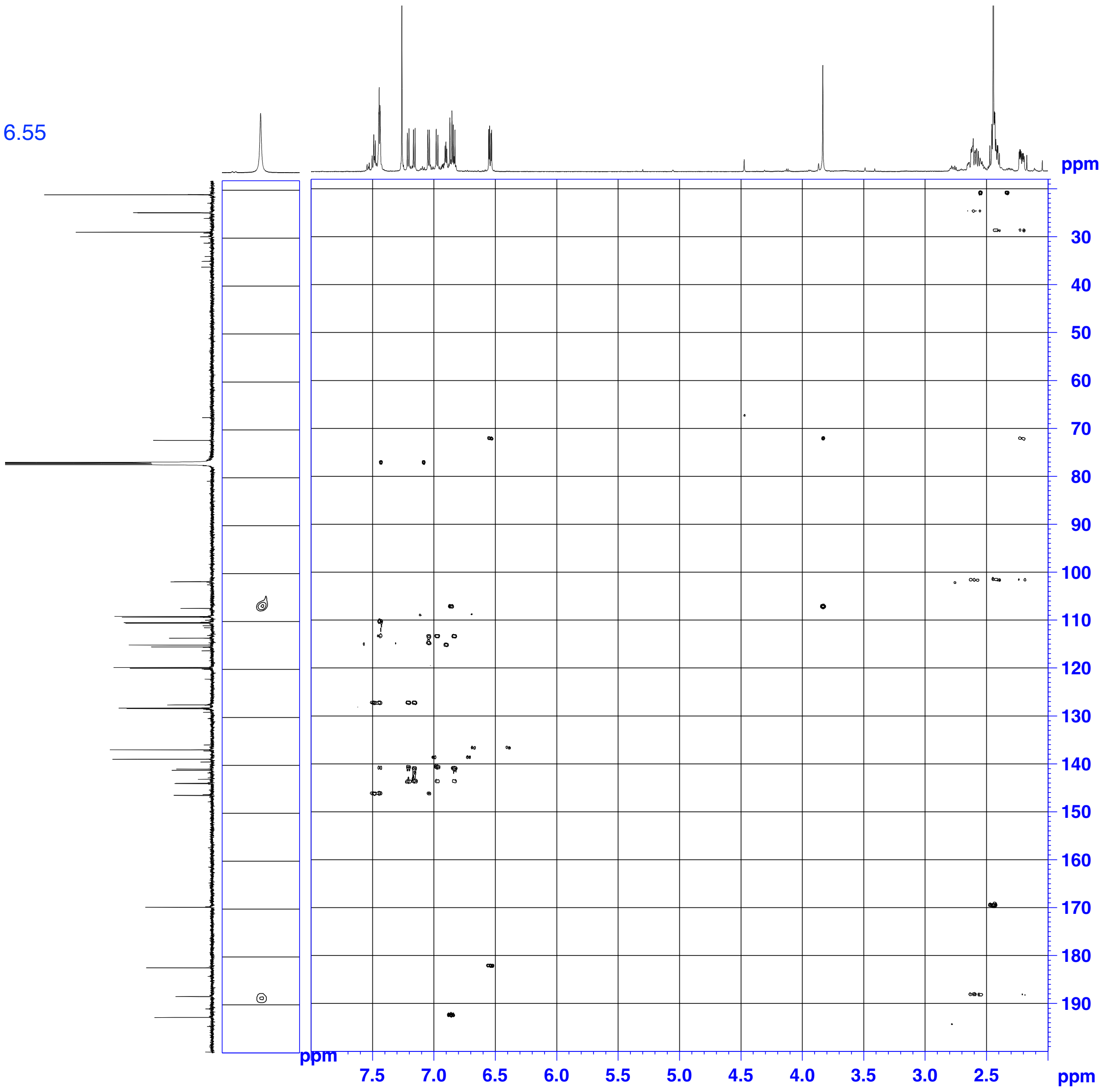


```
Current Data Parameters
NAME      DO1-318-ptlc2
EXPNO                14
PROCNO                1

F2 - Acquisition Parameters
Date_          20220406
Time               7.15
INSTRUM           spect
PROBHD   5 mm CPPBBO BB
PULPROG     hmbcgplpndqf
TD                 2048
SOLVENT           CDCl3
NS                   16
DS                   16
SWH            5263.158 Hz
FIDRES         2.569901 Hz
AQ            0.1945600 sec
RG               175.56
DW               95.000 usec
DE                10.00 usec
TE                298.2 K
CNST2       145.0000000
CNST13       10.0000000
D0           0.00000300 sec
D1           1.43651199 sec
D2           0.00344828 sec
D6           0.05000000 sec
D16          0.00020000 sec
IN0          0.00001490 sec

======== CHANNEL f1 ========
SFO1        600.1322670 MHz
NUC1                 1H
P1                12.00 usec
P2                24.00 usec
PLW1        21.00000000 W

======== CHANNEL f2 ========
SFO2        150.9178741 MHz
NUC2                13C
P3                10.00 usec
PLW2        80.00000000 W

====== GRADIENT CHANNEL =====
GPNAM[1]      SMSQ10.100
GPNAM[2]      SMSQ10.100
GPNAM[3]      SMSQ10.100
GPZ1              50.00 %
GPZ2              30.00 %
GPZ3              40.10 %
P16             1000.00 usec

F1 - Acquisition parameters
TD                  128
SFO1           150.9179 MHz
FIDRES       524.328857 Hz
SW              222.353 ppm
FnMODE               QF

F2 - Processing parameters
SI                 2048
SF          600.1300138 MHz
WDW                SINE
SSB                   0
LB                    0 Hz
GB                    0
PC                 1.40

F1 - Processing parameters
SI                 1024
MC2                  QF
SF          150.9028124 MHz
WDW                SINE
SSB                   0
LB                    0 Hz
GB                    0
```

^{1}H NMR (600 MHz, $CDCl_3$)

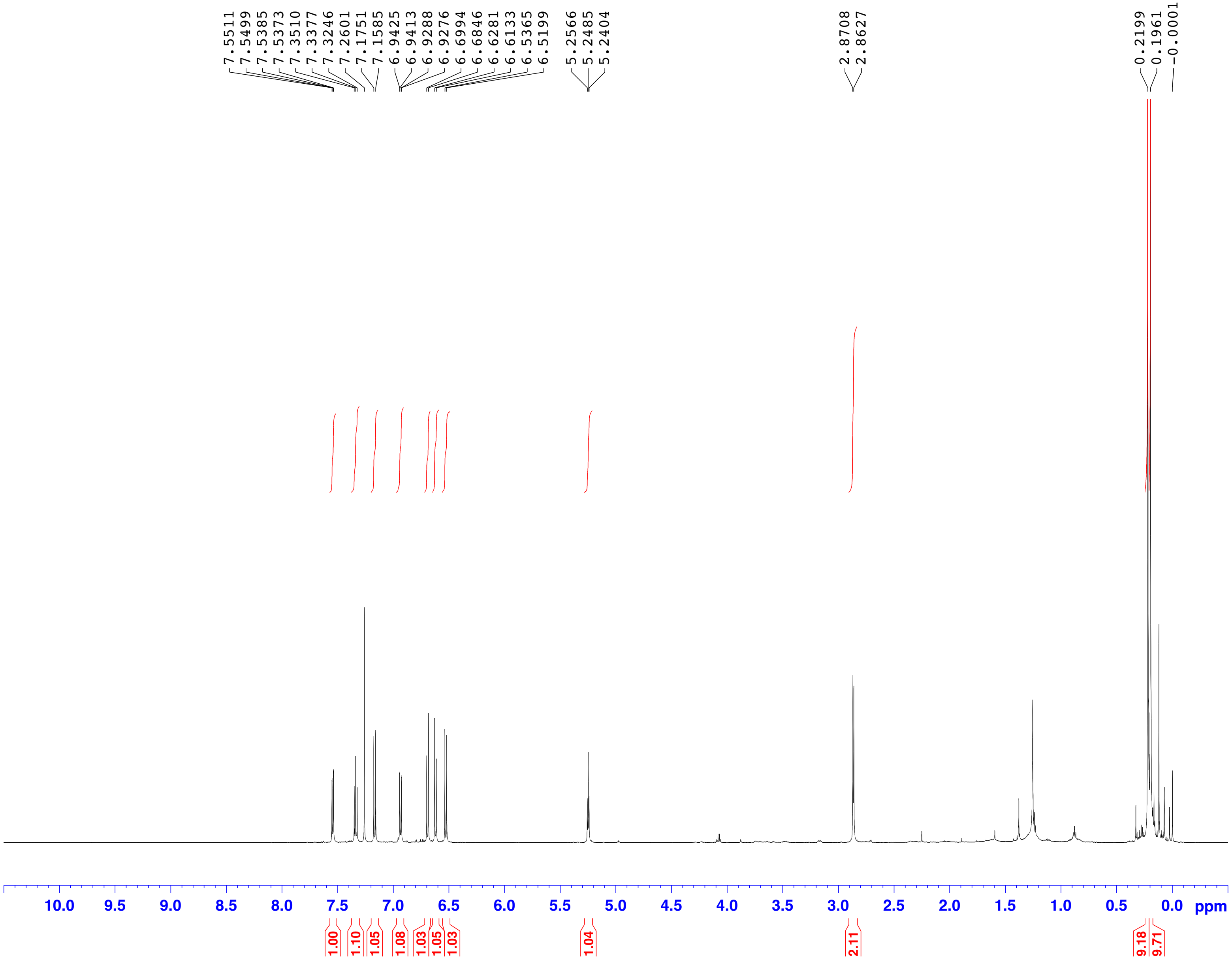

BRUKER

```
Current Data Parameters
NAME        DO1-307-crude
EXPNO                  20
PROCNO                  1

F2 - Acquisition Parameters
Date_            20220207
Time                21.05
INSTRUM             spect
PROBHD    5 mm CPPBBO BB
PULPROG              zg30
TD                  65536
SOLVENT             CDCl3
NS                     16
DS                      2
SWH             12019.230 Hz
FIDRES           0.183399 Hz
AQ              2.7262976 sec
RG                   17.5
DW                 41.600 usec
DE                  10.00 usec
TE                  298.1 K
D1             1.00000000 sec
TD0                     1

======== CHANNEL f1 ========
SFO1          600.1337060 MHz
NUC1                   1H
P1                  12.00 usec
PLW1          21.00000000 W

F2 - Processing parameters
SI                  65536
SF            600.1300143 MHz
WDW                    EM
SSB                     0
LB                   0.30 Hz
GB                      0
PC                   1.00
```

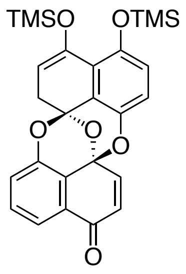

30
(crude)

^{13}C NMR (150 MHz, $CDCl_3$)

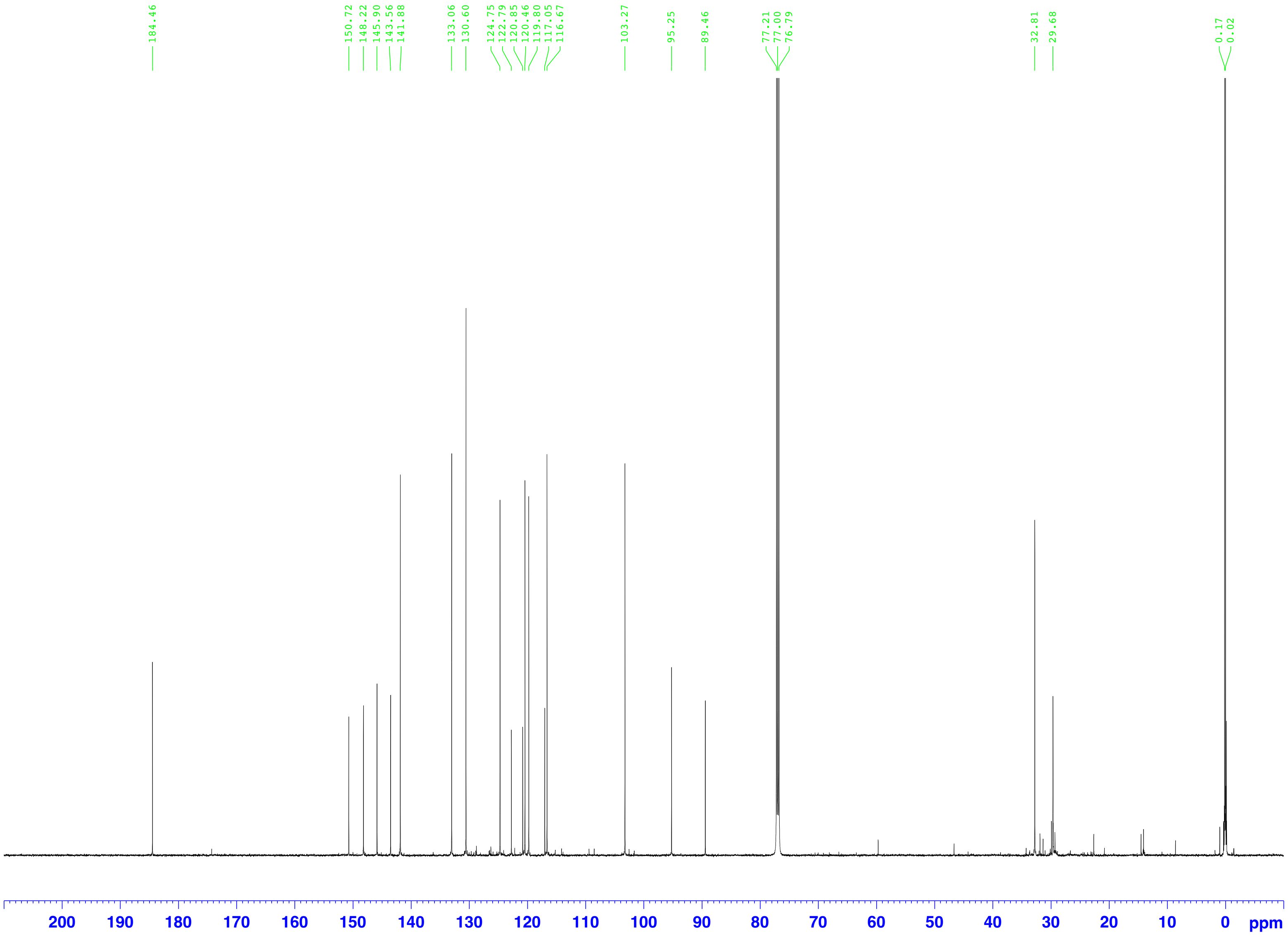

Current Data Parameters
NAME DO1-307-crude
EXPNO 41
PROCNO 1

F2 - Acquisition Parameters
Date_ 20220208
Time 1.27
INSTRUM spect
PROBHD 5 mm CPPBBO BB
PULPROG zgpg30
TD 65536
SOLVENT CDCl3
NS 4096
DS 4
SWH 36057.691 Hz
FIDRES 0.550197 Hz
AQ 0.9087659 sec
RG 175.56
DW 13.867 usec
DE 18.00 usec
TE 298.1 K
D1 2.00000000 sec
D11 0.03000000 sec
TD0 1

======== CHANNEL f1 ========
SFO1 150.9178981 MHz
NUC1 13C
P1 10.00 usec
PLW1 80.00000000 W

======== CHANNEL f2 ========
SFO2 600.1324005 MHz
NUC2 1H
CPDPRG[2 waltz16
PCPD2 70.00 usec
PLW2 13.43999958 W
PLW12 0.61714000 W
PLW13 0.31042001 W

F2 - Processing parameters
SI 32768
SF 150.9028130 MHz
WDW EM
SSB 0
LB 1.00 Hz
GB 0
PC 1.40

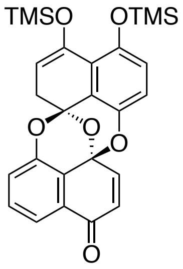

30
(crude)

^{1}H NMR (600 MHz, $CDCl_3$)

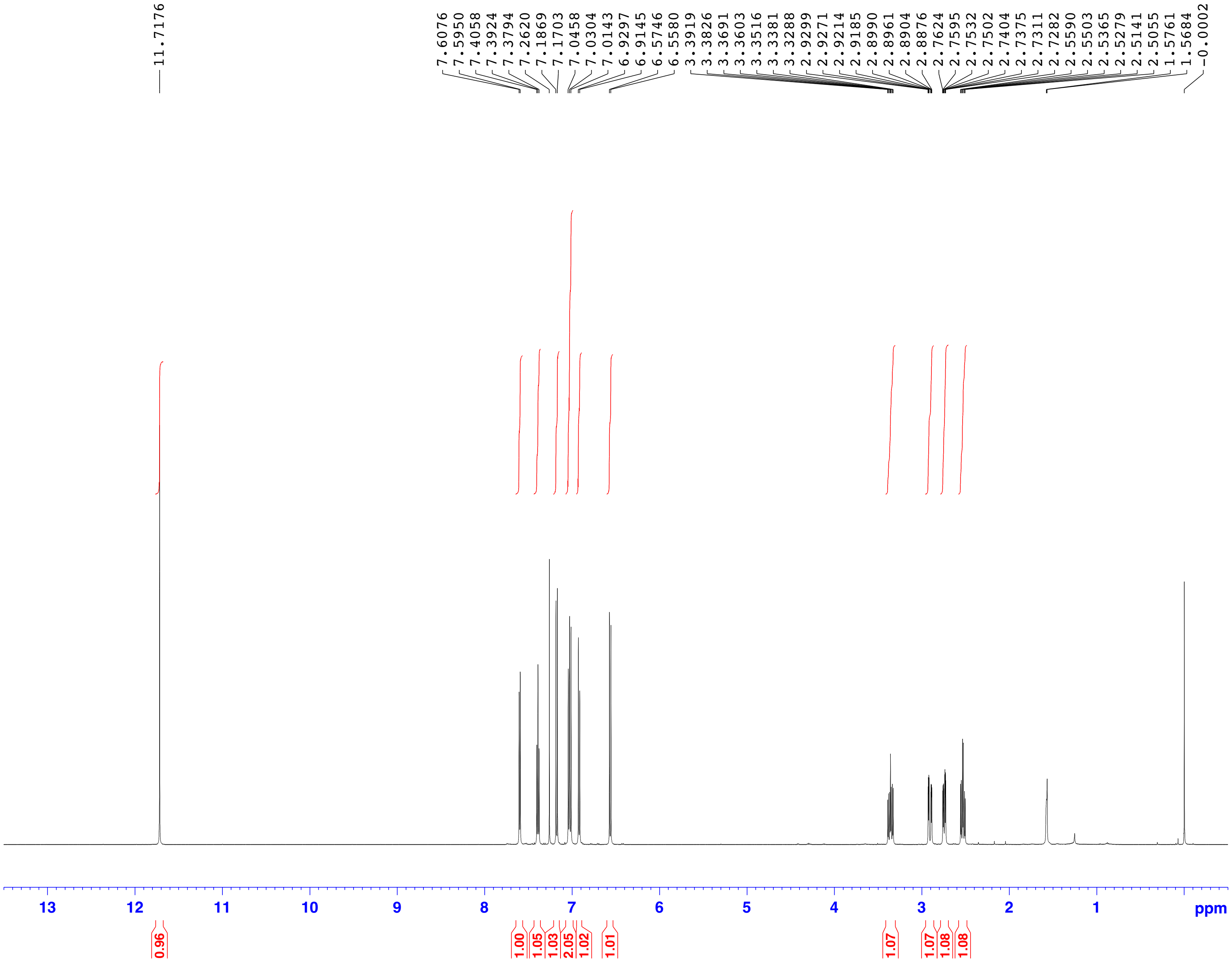

BRUKER

Current Data Parameters
NAME DO1-246-ptlc
EXPNO 10
PROCNO 1

F2 - Acquisition Parameters
Date_ 20211124
Time 22.02
INSTRUM spect
PROBHD 5 mm CPPBBO BB
PULPROG zg30
TD 65536
SOLVENT CDCl3
NS 16
DS 2
SWH 12019.230 Hz
FIDRES 0.183399 Hz
AQ 2.7262976 sec
RG 31.94
DW 41.600 usec
DE 10.00 usec
TE 298.0 K
D1 1.00000000 sec
TD0 1

======== CHANNEL f1 ========
SFO1 600.1337060 MHz
NUC1 1H
P1 12.00 usec
PLW1 21.00000000 W

F2 - Processing parameters
SI 65536
SF 600.1300131 MHz
WDW EM
SSB 0
LB 0.30 Hz
GB 0
PC 1.00

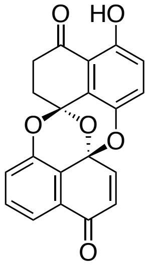

preussomerin EG_1 (**3**)

^{13}C NMR (150 MHz, $CDCl_3$)

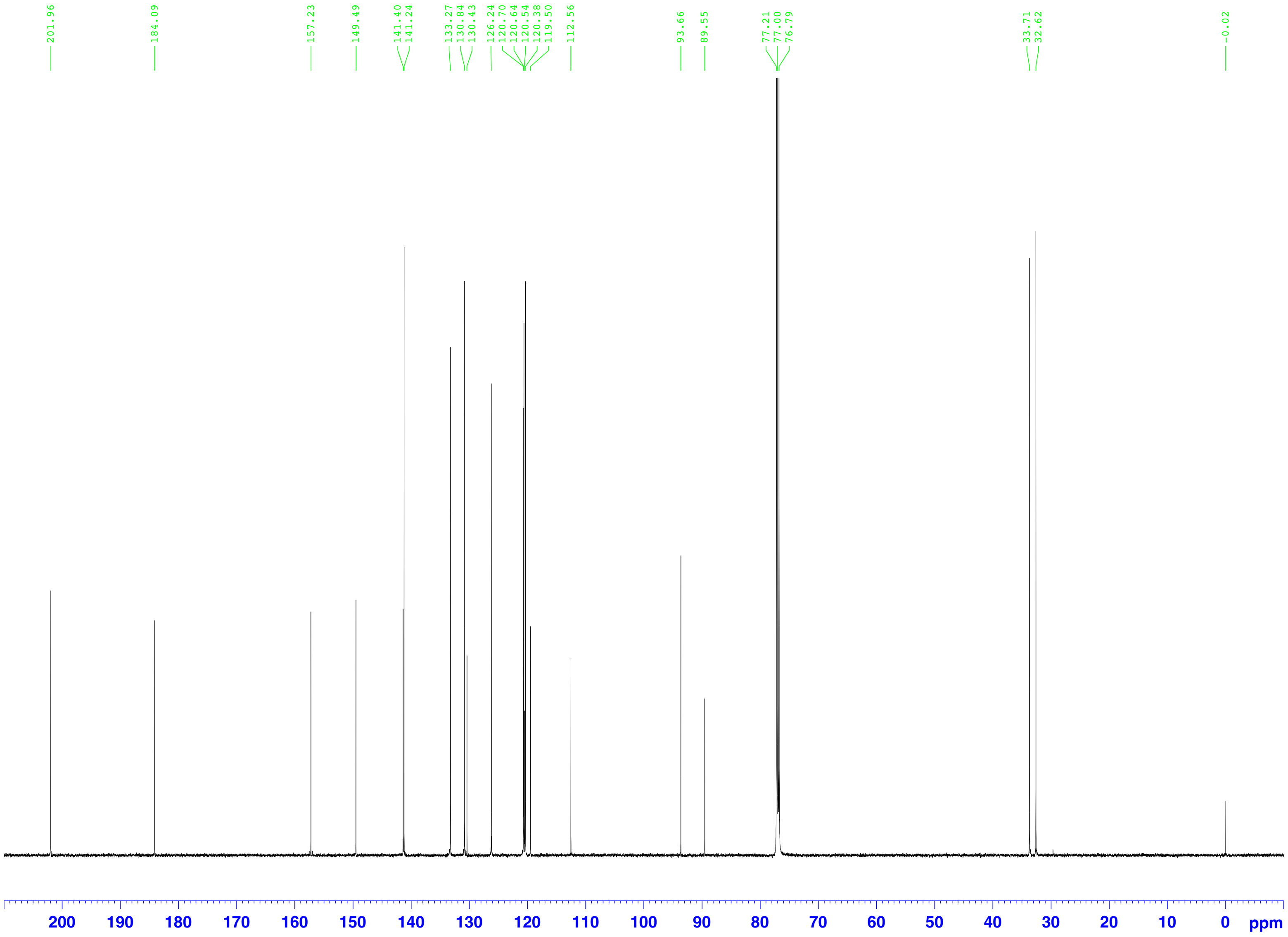


```
Current Data Parameters
NAME          DO1-246-ptlc
EXPNO                   11
PROCNO                   1

F2 - Acquisition Parameters
Date_             20211125
Time                  9.43
INSTRUM              spect
PROBHD    5 mm CPPBBO BB
PULPROG            zgpg30
TD                   65536
SOLVENT              CDCl3
NS                    4096
DS                       4
SWH              36057.691 Hz
FIDRES            0.550197 Hz
AQ               0.9087659 sec
RG                  175.56
DW                  13.867 usec
DE                   18.00 usec
TE                   298.1 K
D1              2.00000000 sec
D11             0.03000000 sec
TD0                      1

======== CHANNEL f1 ========
SFO1          150.9178981 MHz
NUC1                   13C
P1                   10.00 usec
PLW1           80.00000000 W

======== CHANNEL f2 ========
SFO2          600.1324005 MHz
NUC2                    1H
CPDPRG[2           waltz16
PCPD2                70.00 usec
PLW2           13.43999958 W
PLW12           0.61714000 W
PLW13           0.31042001 W

F2 - Processing parameters
SI                   32768
SF            150.9028130 MHz
WDW                     EM
SSB                      0
LB                    1.00 Hz
GB                       0
PC                    1.40
```

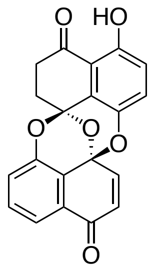

preussomerin EG_1 (**3**)

HSQC

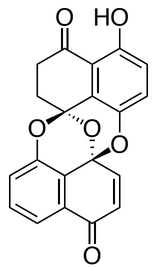

preussomerin EG_1 (**3**)

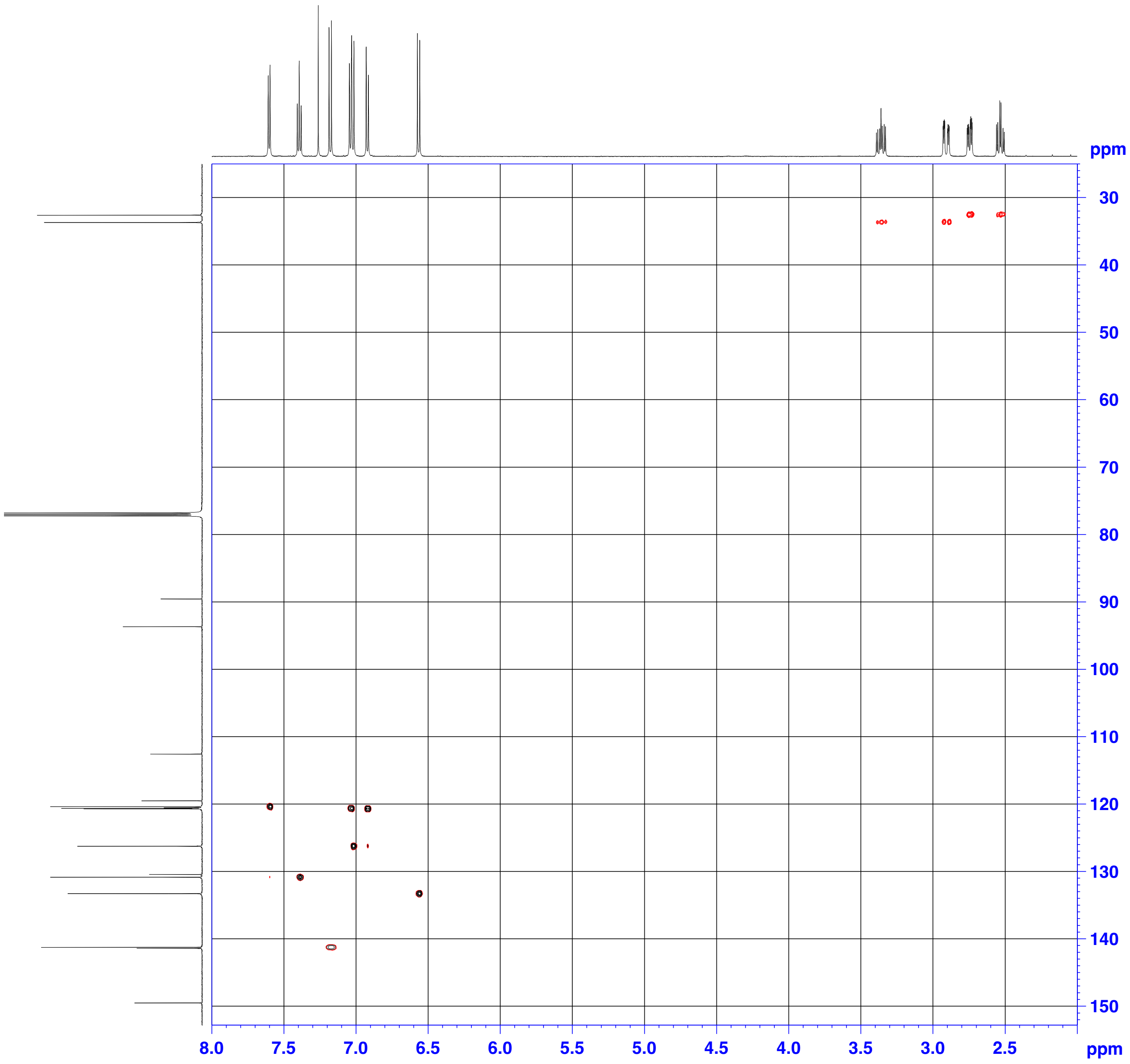


```
Current Data Parameters
NAME        DO1-246-ptlc
EXPNO                 14
PROCNO                 1

F2 - Acquisition Parameters
Date_           20211124
Time               22.25
INSTRUM            spect
PROBHD   5 mm CPPBBO BB
PULPROG  hsqcedetgpsisp2.3
TD                  2048
SOLVENT            CDCl3
NS                     4
DS                    32
SWH             7211.539 Hz
FIDRES          3.521259 Hz
AQ             0.1419947 sec
RG                175.56
DW                69.333 usec
DE                 10.00 usec
TE                 298.1 K
CNST2        145.0000000
CNST17        -0.5000000
D0            0.00000300 sec
D1            2.00000000 sec
D4            0.00172414 sec
D11           0.03000000 sec
D16           0.00020000 sec
D21           0.00360000 sec
D24           0.00089000 sec
IN0           0.00002000 sec

======== CHANNEL f1 ========
SFO1         600.1328224 MHz
NUC1                  1H
P1                 12.00 usec
P2                 24.00 usec
P28                    0 usec
PLW1         21.00000000 W

======== CHANNEL f2 ========
SFO2         150.9133710 MHz
NUC2                 13C
CPDPRG[2            garp
P3                 10.00 usec
P14               500.00 usec
P24              2000.00 usec
P31              1730.00 usec
PCPD2              60.00 usec
PLW0                   0 W
PLW2         80.00000000 W
PLW12         2.22219992 W
SPNAM[3] Crp60,0.5,20.1
SPOAL3             0.500
SPOFFS3                0 Hz
SPW3         12.22299957 W
SPNAM[7]     Crp60comp.4
SPOAL7             0.500
SPOFFS7                0 Hz
SPW7         12.22299957 W
SPNAM[18  Crp60_xfilt.2
SPOAL18            0.500
SPOFFS18               0 Hz
SPW18         3.53270006 W

====== GRADIENT CHANNEL =====
GPNAM[1]      SMSQ10.100
GPNAM[2]      SMSQ10.100
GPNAM[3]      SMSQ10.100
GPNAM[4]      SMSQ10.100
GPZ1               80.00 %
GPZ2               20.10 %
GPZ3               11.00 %
GPZ4               -5.00 %
P16              1000.00 usec
P19               600.00 usec

F1 - Acquisition parameters
TD                   256
SFO1            150.9134 MHz
FIDRES        195.312500 Hz
SW               165.658 ppm
FnMODE      Echo-Antiecho

F2 - Processing parameters
SI                  1024
SF           600.1300131 MHz
WDW                QSINE
SSB                    2
LB                     0 Hz
GB                     0
PC                  1.40

F1 - Processing parameters
SI                  1024
MC2          echo-antiecho
SF           150.9028130 MHz
WDW                QSINE
SSB                    2
LB                     0 Hz
GB                     0
```

HMBC

key HMBC correlations

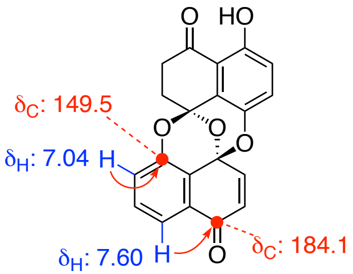

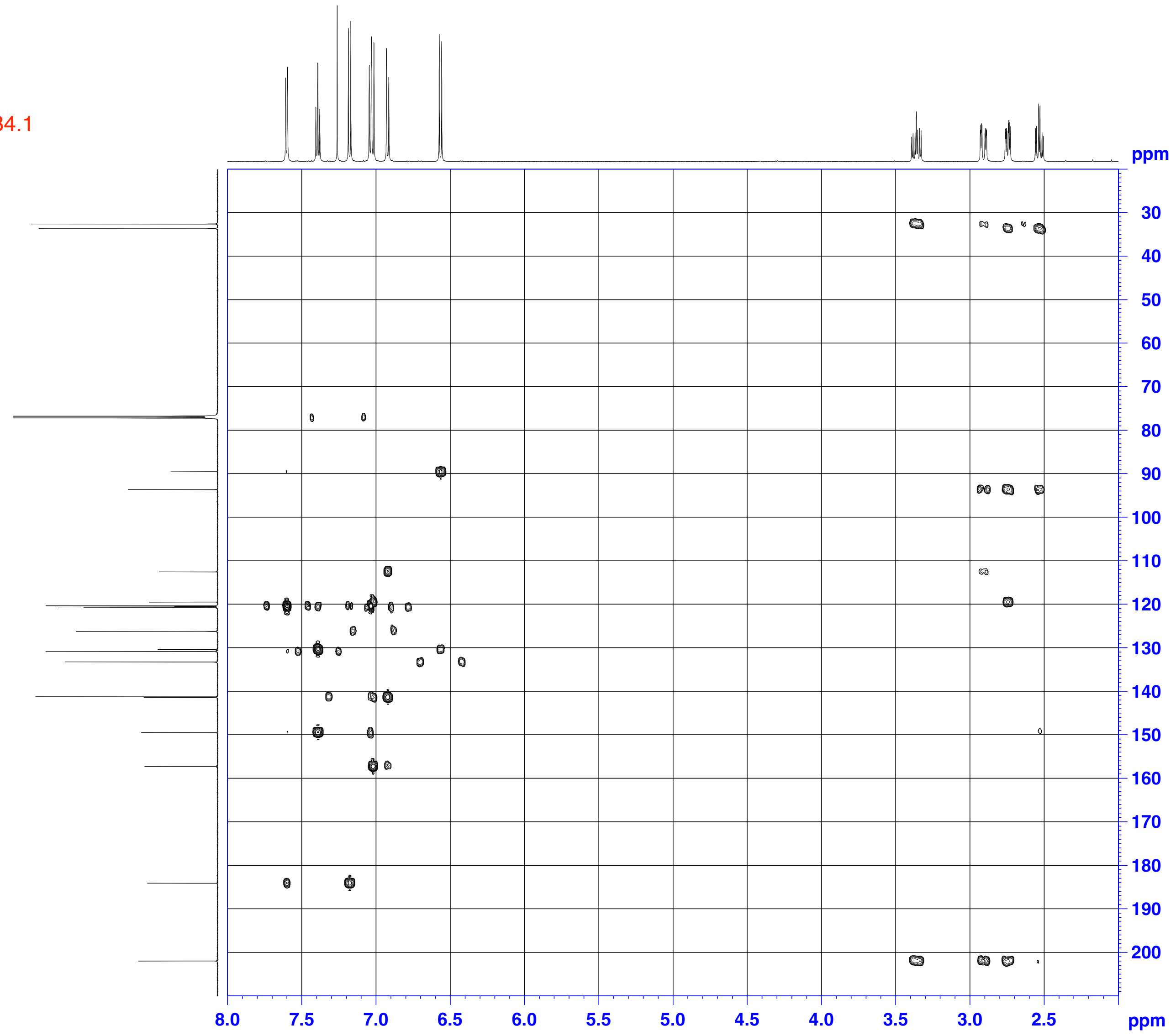


```
Current Data Parameters
NAME          DO1-246-ptlc
EXPNO                   13
PROCNO                   1

F2 - Acquisition Parameters
Date_             20211124
Time                 22.09
INSTRUM              spect
PROBHD    5 mm CPPBBO BB
PULPROG      hmbcgplpndqf
TD                    2048
SOLVENT              CDCl3
NS                       4
DS                      16
SWH               7462.687 Hz
FIDRES            3.643890 Hz
AQ               0.1372160 sec
RG                  175.56
DW                  67.000 usec
DE                   10.00 usec
TE                   298.1 K
CNST2          145.0000000
CNST13          10.0000000
D0              0.00000300 sec
D1              1.49385595 sec
D2              0.00344828 sec
D6              0.05000000 sec
D16             0.00020000 sec
IN0             0.00001490 sec

======== CHANNEL f1 ========
SFO1           600.1337929 MHz
NUC1                    1H
P1                   12.00 usec
P2                   24.00 usec
PLW1           21.00000000 W

======== CHANNEL f2 ========
SFO2           150.9178741 MHz
NUC2                   13C
P3                   10.00 usec
PLW2           80.00000000 W

====== GRADIENT CHANNEL =====
GPNAM[1]       SMSQ10.100
GPNAM[2]       SMSQ10.100
GPNAM[3]       SMSQ10.100
GPZ1                 50.00 %
GPZ2                 30.00 %
GPZ3                 40.10 %
P16                1000.00 usec

F1 - Acquisition parameters
TD                     128
SFO1              150.9179 MHz
FIDRES          524.328857 Hz
SW                 222.353 ppm
FnMODE                  QF

F2 - Processing parameters
SI                    2048
SF             600.1300131 MHz
WDW                   SINE
SSB                      0
LB                       0 Hz
GB                       0
PC                    1.40

F1 - Processing parameters
SI                    1024
MC2                     QF
SF             150.9028130 MHz
WDW                   SINE
SSB                      0
LB                       0 Hz
GB                       0
```

^1H NMR (600 MHz, $CDCl_3$)

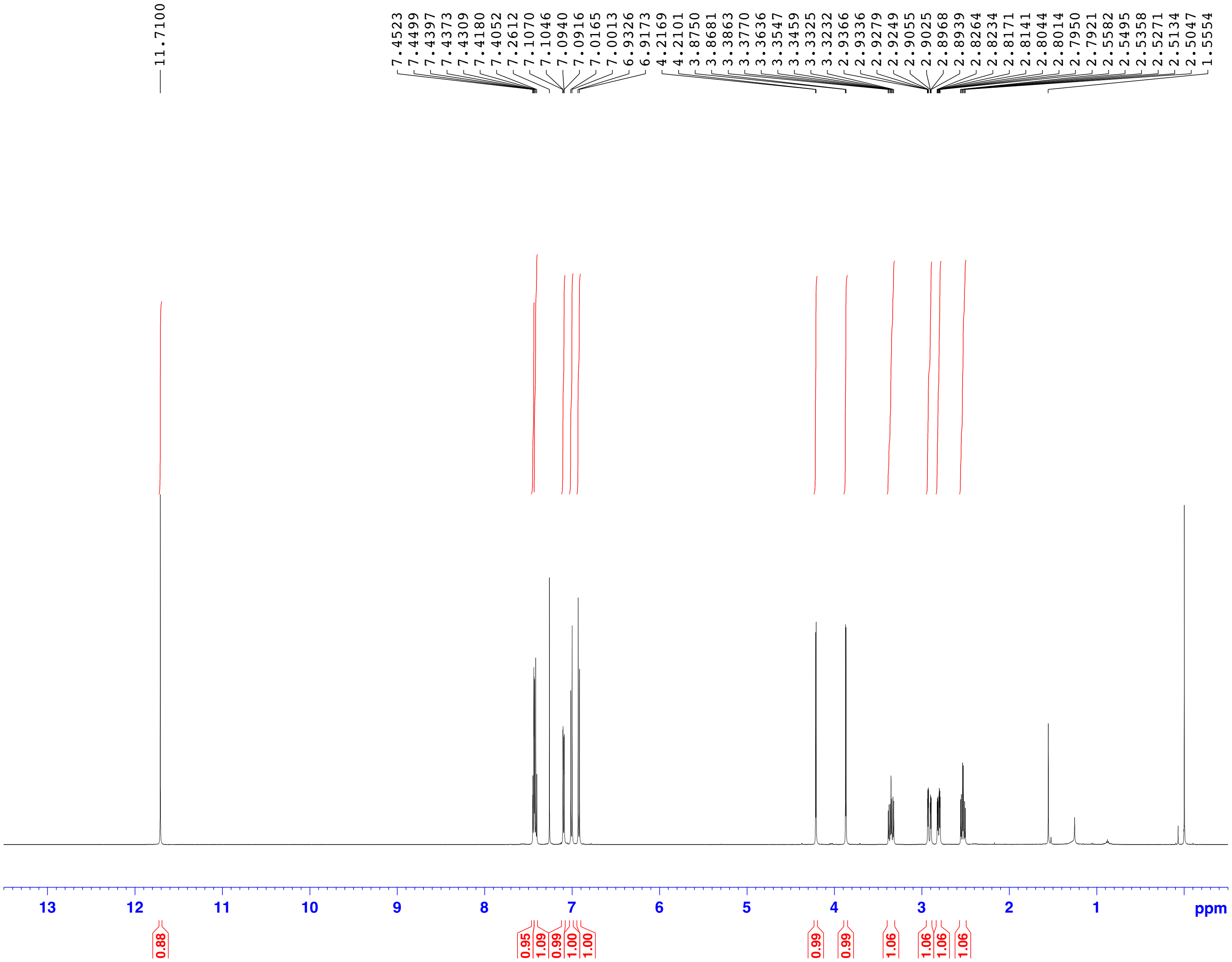

Current Data Parameters
NAME DO1-314-ptlc
EXPNO 50
PROCNO 1

F2 - Acquisition Parameters
Date_ 20220222
Time 19.13
INSTRUM spect
PROBHD 5 mm CPPBBO BB
PULPROG zg30
TD 65536
SOLVENT CDCl3
NS 16
DS 2
SWH 12019.230 Hz
FIDRES 0.183399 Hz
AQ 2.7262976 sec
RG 31.94
DW 41.600 usec
DE 10.00 usec
TE 298.2 K
D1 1.00000000 sec
TD0 1

======== CHANNEL f1 ========
SFO1 600.1337060 MHz
NUC1 1H
P1 12.00 usec
PLW1 21.00000000 W

F2 - Processing parameters
SI 65536
SF 600.1300137 MHz
WDW EM
SSB 0
LB 0.30 Hz
GB 0
PC 1.00

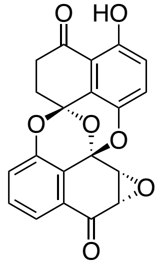

31

^{13}C NMR (150 MHz, $CDCl_3$)

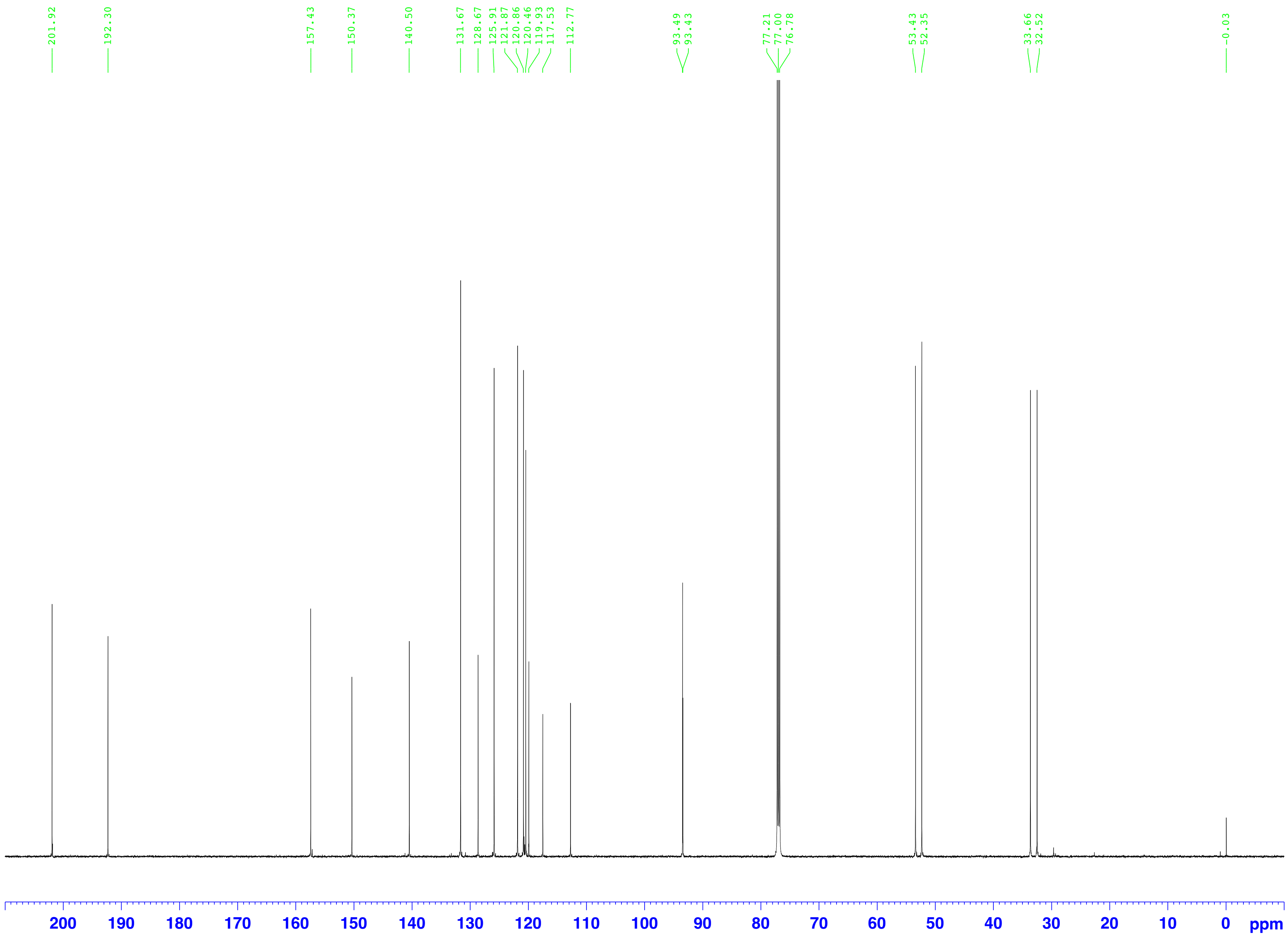


```
Current Data Parameters
NAME        DO1-297-ptlc
EXPNO                 42
PROCNO                 1

F2 - Acquisition Parameters
Date_           20220202
Time                1.28
INSTRUM            spect
PROBHD    5 mm CPPBBO BB
PULPROG          zgpg30
TD                 65536
SOLVENT            CDCl3
NS                  4096
DS                     4
SWH           36057.691 Hz
FIDRES         0.550197 Hz
AQ            0.9087659 sec
RG               175.56
DW               13.867 usec
DE                18.00 usec
TE                298.1 K
D1           2.00000000 sec
D11          0.03000000 sec
TD0                    1

======== CHANNEL f1 ========
SFO1        150.9178981 MHz
NUC1                13C
P1                10.00 usec
PLW1        80.00000000 W

======== CHANNEL f2 ========
SFO2        600.1324005 MHz
NUC2                 1H
CPDPRG[2         waltz16
PCPD2             70.00 usec
PLW2        13.43999958 W
PLW12        0.61714000 W
PLW13        0.31042001 W

F2 - Processing parameters
SI                 32768
SF          150.9028144 MHz
WDW                   EM
SSB                    0
LB                  1.00 Hz
GB                     0
PC                  1.40
```

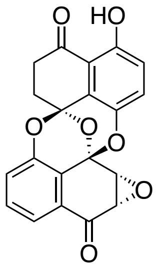

31

^{1}H NMR (600 MHz, $CDCl_3$)

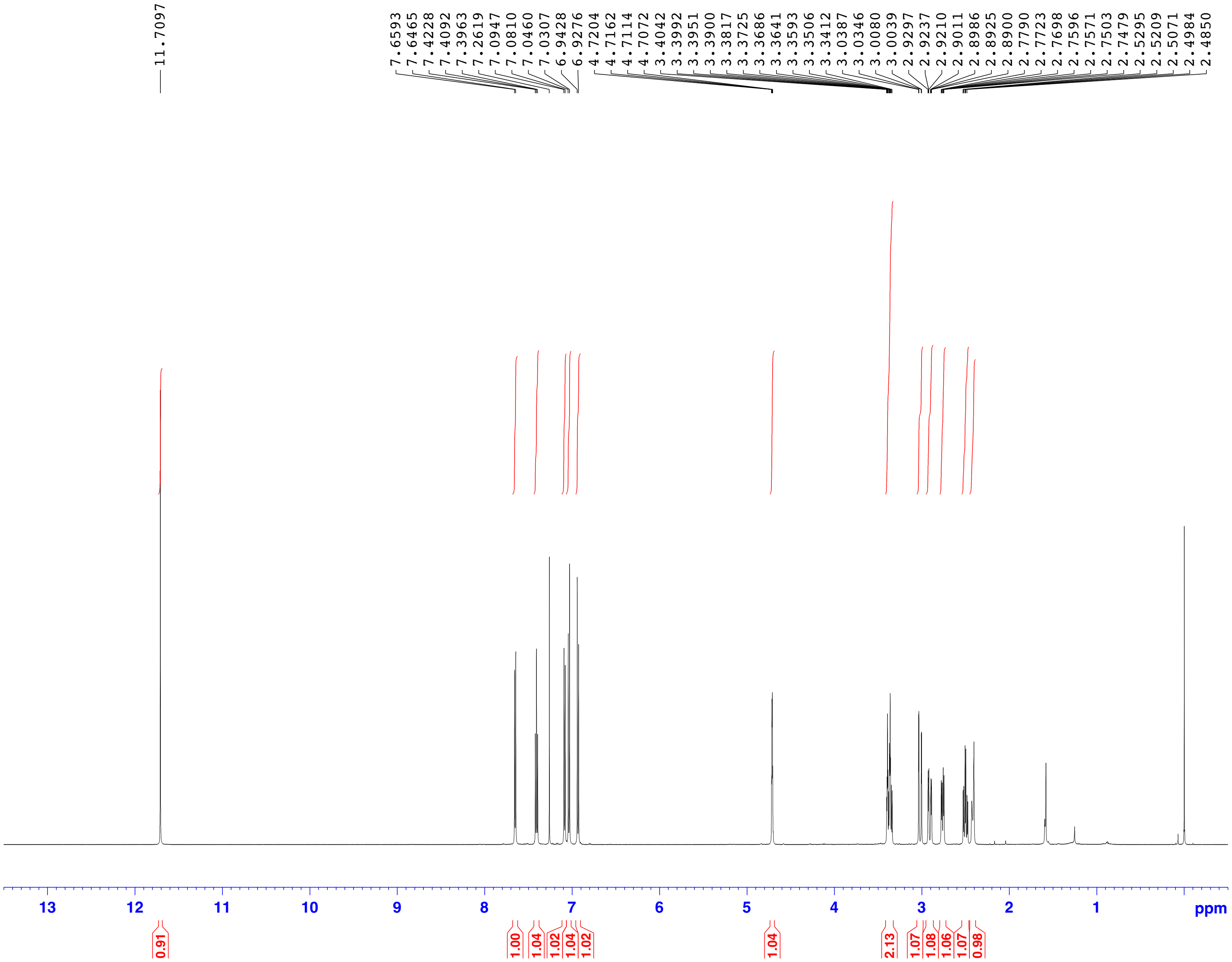

BRUKER

```
Current Data Parameters
NAME          DO1-285-ptlc
EXPNO                   32
PROCNO                   1

F2 - Acquisition Parameters
Date_             20220118
Time                  1.13
INSTRUM              spect
PROBHD    5 mm CPPBBO BB
PULPROG               zg30
TD                   65536
SOLVENT              CDCl3
NS                      16
DS                       2
SWH              12019.230 Hz
FIDRES            0.183399 Hz
AQ               2.7262976 sec
RG                   31.94
DW                  41.600 usec
DE                   10.00 usec
TE                   298.2 K
D1              1.00000000 sec
TD0                      1

======== CHANNEL f1 ========
SFO1           600.1337060 MHz
NUC1                    1H
P1                   12.00 usec
PLW1           21.00000000 W

F2 - Processing parameters
SI                   65536
SF             600.1300131 MHz
WDW                     EM
SSB                      0
LB                    0.30 Hz
GB                       0
PC                    1.00
```

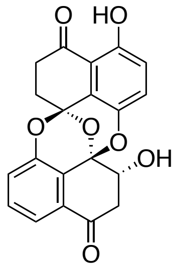

preussomerin EG_2 (**4**)

^{13}C NMR (150 MHz, $CDCl_3$)

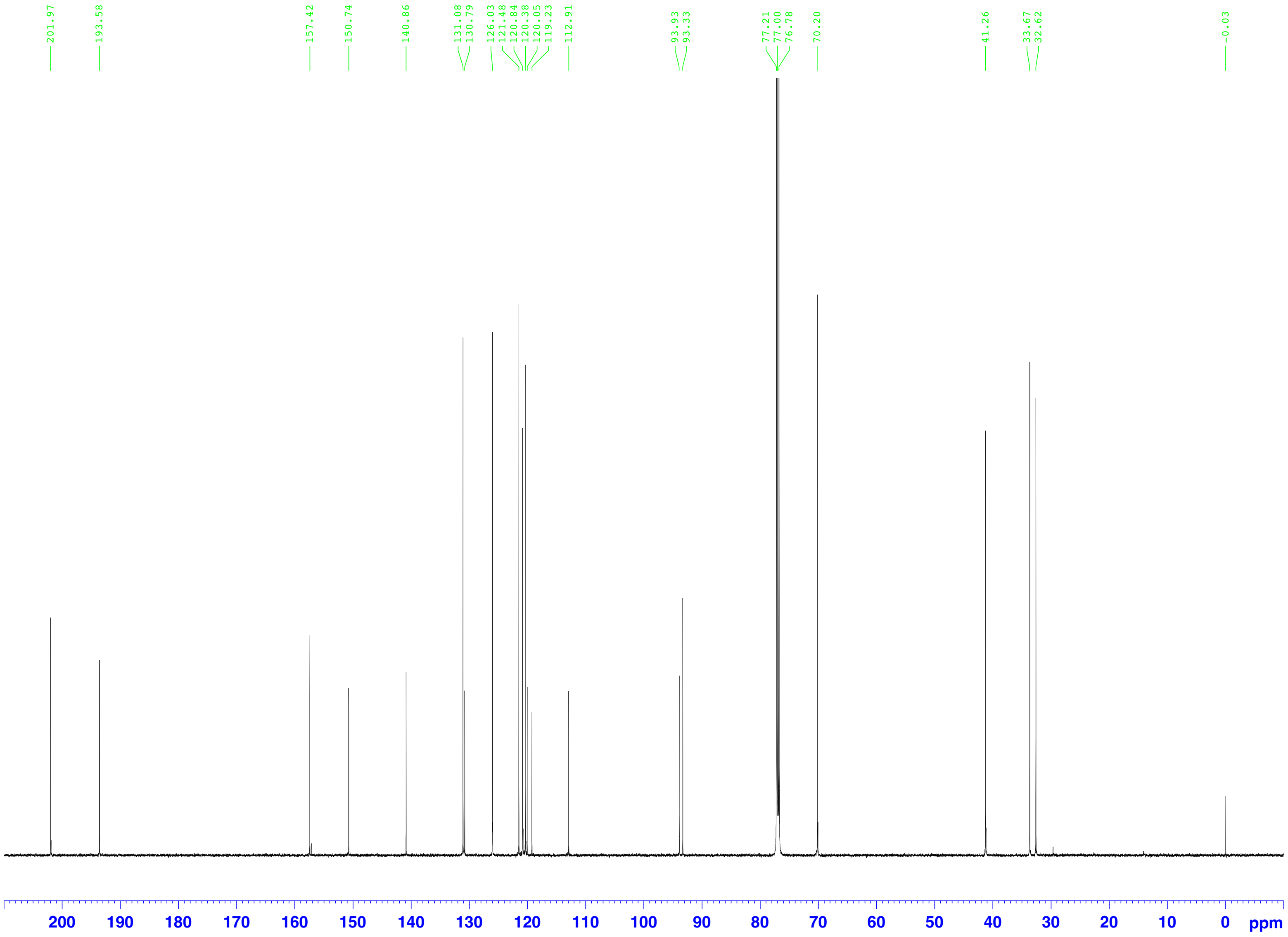


```
Current Data Parameters
NAME          DO1-285-ptlc
EXPNO                   34
PROCNO                   1

F2 - Acquisition Parameters
Date_             20220118
Time                  4.42
INSTRUM              spect
PROBHD    5 mm CPPBBO BB
PULPROG            zgpg30
TD                  65536
SOLVENT             CDCl3
NS                   4096
DS                      4
SWH             36057.691 Hz
FIDRES           0.550197 Hz
AQ              0.9087659 sec
RG                 175.56
DW                 13.867 usec
DE                  18.00 usec
TE                  298.1 K
D1             2.00000000 sec
D11            0.03000000 sec
TD0                     1

======== CHANNEL f1 ========
SFO1          150.9178981 MHz
NUC1                  13C
P1                  10.00 usec
PLW1          80.00000000 W

======== CHANNEL f2 ========
SFO2          600.1324005 MHz
NUC2                   1H
CPDPRG[2           waltz16
PCPD2               70.00 usec
PLW2          13.43999958 W
PLW12          0.61714000 W
PLW13          0.31042001 W

F2 - Processing parameters
SI                  32768
SF            150.9028138 MHz
WDW                    EM
SSB                     0
LB                   1.00 Hz
GB                      0
PC                   1.40
```

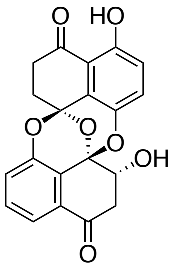

preussomerin EG_2 (**4**)
